# Proceedings of Réanimation 2017, the French Intensive Care Society International Congress

**DOI:** 10.1186/s13613-016-0224-7

**Published:** 2017-01-10

**Authors:** Chtara Kamilia, Kais Regaieg, Najeh Baccouch, Hedi Chelly, Mabrouk Bahloul, Mounir Bouaziz, Ali Jendoubi, Ahmed Abbes, Houda Belhaouane, Oussama Nasri, Layla Jenzri, Salma Ghedira, Mohamed Houissa, Kamal Belkadi, Youness Harti, Afak Nsiri, Khalid Khaleq, Driss Hamoudi, Rachid Harrar, Camille Thieffry, Frédéric Wallet, Erika Parmentier-Decrucq, Raphaël Favory, Daniel Mathieu, Julien Poissy, Thomas Lafon, Philippe Vignon, Emmanuelle Begot, Alexandra Appert, Mathilde Hadj, Paul Claverie, Morgan Matt, Olivier Barraud, Bruno François, Amira Jamoussi, Amira Ben Jazia, Takoua Marhbène, Dhouha Lakhdhar, Jalila Ben Khelil, Mohamed Besbes, Julien Goutay, Caroline Blazejewski, Isabelle Joly-Durand, Isabelle Pirlet, Marie Pierre Weillaert, Sebastien Beague, Soufi Aziz, Reda Hafiane, Khalid Hattabi, Mohamed Aziz Bouhouri, Driss Hammoudi, Abdelaziz Fadil, Rachid Al Harrar, Khalid Zerouali, Fatma Kaaniche Medhioub, Rania Allela, Najla Ben Algia, Samar Cherif, Mohamed Taoufik Slaoui, Souhail Boubia, Y. Hafiani, A. Khaoudi, R. Cherkab, W. Elallam, C. Elkettani, L. Barrou, M. Ridaii, Rihi El Mehdi, Caroline Schimpf, Assaf Mizrahi, Benoît Pilmis, Alban Le Monnier, Kelly Tiercelet, Mélanie Cherin, Cédric Bruel, Francois Philippart, Sébastien Bailly, Jc Lucet, Alain Lepape, François L’hériteau, Martine Aupée, Caroline Bervas, Sandrine Boussat, Anne Berger-Carbonne, Anaïs Machut, Anne Savey, Jean-François Timsit, REA‑RAISIN Study group, OUTCOMEREA study group, Keyvan Razazi, Jérémy Rosman, Nicolas de Prost, Guillaume Carteaux, Chloe Jansen, Jean Winoc Decousser, Christian Brun-Buisson, Armand Mekontso Dessap, Aymen M’rad, Zouhour Ouali, Manel Barghouth, Achille Kouatchet, Rafael Mahieu, Emmanuel Weiss, David Schnell, Jean-Ralph Zahar, Margaux Artiguenave, Paktoris-Papine Sophie, Florence Espinasse, Faten El Sayed, Aurélien Dinh, Cyril Charron, Guillaume Geri, Antoine Vieillard-Baron, Xavier Repessé, Hatem Kallel, Claire Mayence, Stéphanie Houcke, Pascal Guegueniat, Didier Hommel, Kaouther Dhifaoui, Zied Hajjej, Amira Fatnassi, Walid Sellami, Iheb Labbene, Mustapha Ferjani, Fahmi Dachraoui, Sabrine Nakkaa, Abdelwaheb M’ghirbi, Ali Adhieb, Dhouha Ben Braiek, Kmar Hraiech, Ali Ousji, Islem Ouanes, Hammouda Zaineb, Saousen Ben Abdallah, Lamia Ouanes-Besbes, Fekri Abroug, Simon Klein, Mattéo Miquet, Jean-Marc Thouret, Vincent Peigne, Jean-Louis Daban, Mathieu Boutonnet, Bernard Lenoir, Takoua Merhbene, Celine Derreumaux, Thierry Seguin, Jean-Marie Conil, Charlotte Kelway, Valery Blasco, Cyril Nafati, Karim Harti, Laurent Reydellet, Jacques Albanese, Narjess Ben Aicha, Khaoula Meddeb, Ahmed Khedher, Jihene Ayachi, Nesrine Fraj, Nesrine Sma, Imed Chouchene, Mohamed Boussarsar, Soumaya Ben Yedder, Walid Samoud, Bousselmi Radhouene, Bousselmi Mariem, Asma Ammar, Asma Ben Cheikh, Hend Ben Lakhal, Messaouda Khelfa, Yamina Hamdaoui, Nabiha Bouafia, Timothée Trampont, Thomas Daix, Vincent Legarçon, Henri Hani Karam, Nicolas Pichon, Fatma Essafi, Nasreddine Foudhaili, Hafedh Thabet, Youssef Blel, Nozha Brahmi, Hanane Ezzouine, Mahmoud Kerrous, Saad El Haoui, Soufiane Ahdil, Abdellatif Benslama, Khalid Abidi, Tarek Dendane, Ssouni Oussama, Jihane Belayachi, Naoufal Madani, Redouane Abouqal, Amine Ali Zeggwagh, Hatem Ghadhoune, Anis Chaari, Guissouma Jihene, Hend Allouche, Insaf Trabelsi, Habib Brahmi, Mohamed Samet, Hatem El Ghord, Ben Sik Ali Habiba, Nouira Hajer, Najla Tilouch, Sondes Yaakoubi, Oussama Jaoued, Rim Gharbi, Mohamed Fekih Hassen, Souheil Elatrous, Julien Arcizet, Bertrand Leroy, Caroline Abdulmalack, Catherine Renzullo, Maël Hamet, Jean-Marc Doise, Jérôme Coutet, Chaigar Mohammed Cheikh, Zakaria Quechar, Magalie Joris, Dimitri Titeca Beauport, Loay Kontar, Delphine Lebon, Bérengère Gruson, Michel Slama, Jean-Pierre Marolleau, Julien Maizel, Julie Gorham, Lieveke Ameye, Thierry Berghmans, Marianne Paesmans, Jean-Paul Sculier, Anne-Pascale Meert, Max Guillot, Marie-Pierre Ledoux, Thierry Braun, Quentin Maestraggi, Baptiste Michard, Vincent Castelain, Raoul Herbrecht, Francis Schneider, Severine Couffin, David Lobo, Nicolas Mongardon, Gilles Dhonneur, Roman Mounier, Pierrick Le Borgne, Sophie Couraud, Jean-Etienne Herbrecht, Alexandra Boivin, François Lefebvre, Pascal Bilbault, Setti-Aouicha Zelmat, Djamila-Djahida Batouche, Fatima Mazour, Belkacem Chaffi, Nadia Benatta, Ali Habiba Sik, I. Talik, Maxime Perrier, Eliane Gouteix, Claude Koubi, Annabelle Escavy, Victoria Guilbaut, Jean-Philippe Fosse, Rahma Ben Jazia, Ahmed Abdelghani, Pierre-Julien Cungi, Julien Bordes, Cédric Nguyen, Candice Pierrou, Maximilien Cruc, Alain Benois, Frédéric Duprez, Thierry Bonus, Grégory Cuvelier, Sandra Ollieuz, Sharam Machayekhi, Frédéric Paciorkowski, Gregory Reychler, Remi Coudroy, Arnaud W. Thille, Xavier Drouot, Véronique Diaz, Jean-Claude Meurice, René Robert, Olfa Turki, Hmida Chokri Ben, Mona Assefi, Romain Deransy, Hélène Brisson, Antoine Monsel, Filomena Conti, Olivier Scatton, Olivier Langeron, Hassen Ben Ghezala, Salah Snouda, Chiekh Imen Ben, Moez Kaddour, Anwar Armel, Lafrikh Youness, Bensaid Abdelhak, Miloudi Youssef, Al Harrar Najib, Amouzoun Mustapha, Mtioui Noufel, Zamd Mohamed, El Khayat Salma, Medkouri Ghizlane, Benghanam Mohamed, Ramdani Benyounes, Florent Montini, Sébastien Moschietto, Emilien Gregoire, Guillaume Claisse, Julien Guiot, Philippe Morimont, Jean-Marie Krzesinski, Christophe Mariat, Bernard Lambermont, Etienne Cavalier, Pierre Delanaye, Soumia Benbernou, Sofiane Ilies, Abdelkader Azza, Khalida Bouyacoub, Meriem Louail, Houria Mokhtari-Djebli, Romain Arrestier, Fabrice Daviaud, Xavier Laborne Francois, Elsa Brocas, Gérald Choukroun, Oscar Peñuelas, José-Angel Lorente, Pablo Cardinal-Fernandez, José-Maria Rodriguez, José-Antonio Aramburu, Andres Esteban, Fernando Frutos-Vivar, Laurent Bitker, Nicolas Costes, Didier Le Bars, Franck Lavenne, Mojgan Devouassoux, Jean-Christophe Richard, Malika Mechati, Marc Gainnier, Laurent Papazian, Christophe Guervilly, Aude Garnero, Jean Michel Arnal, Hadrien Roze, Jean Christophe Richard, Benjamin Repusseau, Antoine Dewitte, Olivier Joannes-Boyau, Alexandre Ouattara, Nadia Harbouze, A. M. Amine, A. G. Olandzobo, Alexandre Herbland, Marie Richard, Nicolas Girard, Lucile Lambron, Olivier Lesieur, Sarah Wainschtein, Sidonie Hubert, Albane Hugues, Marc Tran, Philippe Bouillard, Vlad Loteanu, Maxime Leloup, Alexandra Laurent, Florent Lheureux, Alessia Prestifilippo, Martin Delgado Maria Cruz, Rigal Romain, Massimo Antonelli, Torra Lluis Blanch, Franck Bonnetain, Maria Grazzia-Bocci, Jordi Mancebo, Emmanuel Samain, Hebert Paul, Gilles Capellier, Taissa Zavgorodniaia, Marion Soichot, Isabelle Malissin, Sebastian Voicu, Pierre Garçon, Antoine Goury, Lamia Kerdjana, Nicolas Deye, Emmanuel Bourgogne, Bruno Megarbane, Olfa Mejri, Marwa Ben Hmida, Salma Tannous, Lucie Chevillard, Laurence Labat, Patricia Risede, Hana Fredj, Maxime Léger, Marion Brunet, Gaël Le Roux, David Boels, Nicolas Lerolle, Souaad Farah, Hélène Amiel-Niemann, Nathalie Kubis, Xavier Declèves, Nicoals Peyraux, Frederic Baud, Micaela Serafini, Jean-Claude Alvarez, Annette Heinzelman, Mathieu Jozwiak, Sandrine Millasseau, Jean-Louis Teboul, Jean-Emmanuel Alphonsine, François Depret, Nathalie Richard, Pierre Attal, Christian Richard, Xavier Monnet, Denis Chemla, Salma Jerbi, Wafa Khedhiri, Hatem Necib, Paolo Scarfo, Charles Chevalier, Michael Piagnerelli, Alexandre Lafont, Antoine Galy, Claire Mancia, Amel Zerhouni, Kheira Tabeliouna, Ali Gaja, Bassem Hamrouni, Abir Malouch, Sami Fourati, Rihab Messaoud, Youssef Zarrouki, Amra Ziadi, Manal Rhezali, Zahira Zouizra, Drissi Boumzebra, Mohamed Abdennasser Samkaoui, Jennifer Brunet, Bertrand Canoville, Pierre Verrier, Calin Ivascau, Amélie Seguin, Xavier Valette, Damien Du Cheyron, Cedric Daubin, Wulfran Bougouin, Nadia Aissaoui, Lionel Lamhaut, Daniel Jost, Carole Maupain, Frankie Beganton, Adrien Bouglé, Florence Dumas, Eloi Marijon, Xavier Jouven, Alain Cariou, Sudden Death Expertise Center, Florent Poirson, Ulriikka Chaput, Thomas Beeken, Leclerc Maxime, Oueslati Haikel, Dominique Vodovar, Jonathan Chelly, Philippe Marteau, Richard Chocron, Philippe Juvin, Thomas Loeb, Frederic Adnet, Eric Lecarpentier, Antoine Riviere, Bertand De Cagny, Thierry Soupison, Elodie Privat, Joséphine Escutnaire, Cyrielle Dumont, Valentine Baert, Christian Vilhelm, Hervé Hubert, Stéphane Leteurtre, Marion Fresco, Michael Bubenheim, Gaetan Beduneau, Dorothée Carpentier, Steven Grange, Elise Artaud-Macari, Benoit Misset, Fabienne Tamion, Christophe Girault, Guillaume Dumas, Sylvie Chevret, Virginie Lemiale, Djamel Mokart, Julien Mayaux, Frédéric Pène, Martine Nyunga, Pierre Perez, Anne-Sophie Moreau, Fabrice Bruneel, François Vincent, Kada Klouche, Jean Reignier, Antoine Rabbat, Elie Azoulay, Jean-Pierre Frat, Stéphanie Ragot, Jean-Michel Constantin, Gwenael Prat, Alain Mercat, Thierry Boulain, Alexandre Demoule, Jérôme Devaquet, Saad Nseir, Julien Charpentier, Laurent Argaud, Pascal Beuret, Jean-Damien Ricard, Christelle Teiten, Nicolas Marjanovic, Nicola Palamin, Erwan L’Her, Arthur Bailly, Julie Boisramé-Helms, Benoit Champigneulle, Toufik Kamel, Emmanuelle Mercier, Aurélie Le Thuaut, Jean-Baptiste Lascarrou, Amélie Rolle, Audrey De Jong, Gérald Chanques, Samir Jaber, Geoffroy Hariri, Jean-Luc Baudel, Vincent Dubée, Gabriel Preda, Simon Bourcier, Jeremie Joffre, Naïke Bigé, Hafid Ait-Oufella, Eric Maury, Houda Mater, Hamid Merdji, David Grimaldi, Christophe Rousseau, Jean-Paul Mira, Jean-Daniel Chiche, Ines Sedghiani, A. Benabderrahim, Dhekra Hamdi, Asma Jendoubi, Mohamed Ali Cherif, Youssef Zied El Hechmi, Jerbi Zouheir, François Bagate, Radhwen Bousselmi, Frédérique Schortgen, Pierre Asfar, Emmanuel Guérot, Grelon Fabien, Nadia Anguel, Lasocki Sigismond, Henry-Lagarrigue Matthieu, Frédéric Gonzalez, Legay François, Christophe Guitton, Maleka Schenck, Doise Jean-Marc, Didier Dreyfuss, Peter Radermacher, for the HYPER2S Investigators and REVA research network, Antoine Frère, Laurent Martin-Lefèvre, Gwenhaël Colin, Maud Fiancette, Matthieu Henry-Laguarrigue, Jean-Claude Lacherade, Christine Lebert, Isabelle Vinatier, Aihem Yehia, Aurélie Joret, Nicolas Menunier-Beillard, Dalila Benzekri-Lefevre, Arnaud Desachy, Fréderic Bellec, Gaëtan Plantefève, Jean-Pierre Quenot, Ferhat Meziani, Elsa Tavernier, Stephan Ehrmann, Clinical Research in Intensive Care and Sepsis (CRICS network), Nicolas Chudeau, Tommy Raveau, Valérie Moal, Pascal Houillier, Emmanuelle Rouve, Karim Lakhal, Charlotte Salmon Gandonnière, Youenn Jouan, Laetitia Bodet-Contentin, Adrien Balmier, Jonathan Messika, Cubrea network, Etienne De Montmollin, Victorine Pouyet, Benjamin Sztrymf, Abirami Thiagarajah, Damien Roux, Marc Pineton De Chambrun, Charles-Edouard Luyt, François Beloncle, Nathalie Zapella, Stanislas Ledochowsky, Nicolas Terzi, Jean-Marc Mazou, Romain Sonneville, Sylvie Paulus, Yannick Fedun, Mickael Landais, Jean-Herlé Raphalen, Alain Combes, Zahir Amoura, Aemilia Jacquemin, Felipe Guerrero, Bertrand Marcheix, Nicolas Hernandez, Olivier Fourcade, Bernard Georges, Clément Delmas, Sarah Makoudi, Audrey Genton, Rémy Bernard, Guillaume Lebreton, Julien Amour, Charlotte Mazet, Fanny Bounes, Gurbuz Murat, Laure Cronier, Guillaume Robin, Caroline Biendel, Stein Silva, Samia Boubeche, Caroline Abriou, Véronique Wurtz, Vincent Scherrer, Nathalie Rey, Gioia Gastaldi, Benoit Veber, Fabien Doguet, Arnaud Gay, Bertrand Dureuil, Emmanuel Besnier, Antoine Rouget, Guillaume Gantois, Eric Magalhaes, Ruben Wanono, Roland Smonig, Mathilde Lermuzeaux, Jordane Lebut, Andremont Olivier, Claire Dupuis, Aguila Radjou, Bruno Mourvillier, Mathilde Neuville, Marie Pia D’ortho, Lila Bouadma, Anny Rouvel-Tallec, Marika Rudler, Nicolas Weiss, Vincent Perlbarg, Damien Galanaud, Dominique Thabut, Brain Liver Pitié-Salpêtrière Study Group (BLIPS), Emna Rachdi, Ghada Mhamdi, Ahlem Trifi, Rim Abdelmalek, Sami Abdellatif, Foued Daly, Rochdi Nasri, Hanene Tiouiri, Salah Ben Lakhal, Geoffroy Rousseau, Romain Asmolov, Leslie Grammatico-Guillon, Adrien Auvet, Said Laribi, Denis Garot, Pierre François Dequin, Antoine Guillon, Jean-Louis Fergé, Gwénolé Abgrall, Ronan Hinault, Shazima Vally, Benoit Roze, Agathe Chaplain, Cyrille Chabartier, Anne-Charlotte Savidan, Sabia Marie, Andre Cabie, Dabor Resiere, Ruddy Valentino, Hossein Mehdaoui, Lucas Benarous, Marième Soda-Diop, Fouad Bouzana, Gilles Perrin, Jeremy Bourenne, Béatrice Eon, Dominique Lambert, Agnes Trebuchon, Géraldine Poncelet, Fleur Le Bourgeois, Levy Michael, Guillot Camille, Jérôme Naudin, Anna Deho, Stéphane Dauger, Michaël Sauthier, Krystale Bergeron-Gallant, Guillaume Emeriaud, Philippe Jouvet, Nicolas Tiebergien, Matthias Jacquet-Lagrèze, Jean-Luc Fellahi, Florent Baudin, Sandrine Essouri, Etienne Javouhey, Claude Guérin, Marie Lampin, Ouardia Mamouri, Patrick Devos, Yasemin Karaca-Altintas, Matthieu Vinchon, David Brossier, Redha Eltaani, Sonia Teyssedre, Meyet Sabine, Jean-Christophe Bouchut, Olivier Peguet, Lucie Petitdemange, Anne Sophie Guilbert, Nabil Tabet Aoul, Zakaria Addou, Nabil Aouffen, Benqqa Anas, Samira Kalouch, Khalid Yaqini, Aziz Chlilek, Rchi Abdou, Perrine Gravellier, Julie Chantreuil, Nadine Travers, Antoine Listrat, Claire Le Reun, Geraldine Favrais, Zoe Coppere, Stéphane Blanot, Juliette Montmayeur, Régis Bronchard, Stephane Rolando, Gilles Orliaguet, Pierre-Louis Leger, Jérôme Rambaud, Emilie Thueux, Alexandra De Larrard, Véronique Berthelot, Julien Denot, Marie Reymond, Alain Amblard, Sarah Morin-Zorman, Etienne Lengliné, Claire Pichereau, Eric Mariotte, Canet Emmanuel, Julien Poujade, Guillaume Trumpff, Ralf Janssen-Langenstein, Marie-Line Harlay, Noorah Zaid, Nawel Ait-Ammar, Christine Bonnal, Jean-Claude Merle, Francoise Botterel, Eric Levesque, Zakaria Riad, Mehdi Mezidi, Hodane Yonis, Mylène Aublanc, Sophie Perinel-Ragey, Floriane Lissonde, Aurore Louf-Durier, Romain Tapponnier, Bruno Louis, Jean-Marie Forel, Magali Bisbal, Samuel Lehingue, Romain Rambaud, Mélanie Adda, Sami Hraiech, Elisa Marchi, Antoine Roch, Vincent Guerin, Sacha Rozencwajg, Matthieu Schmidt, Guillaume Hekimian, Nicolas Bréchot, Jean Louis Trouillet, Sébastien Besset, Guillaume Franchineau, Ania Nieszkowska, Leprince Pascal, Maud Loiselle, Chemam Sarah, Dangers Laurence, Thomas Guillemette, Alice Jacquens, Sebastien Kerever, Bertrand Guidet, Philippe Aegerter, Vincent Das, Muriel Fartoukh, Jan Hayon, Mathieu Desmard, Jean-Pierre Fulgencio, Benjamin Zuber, A. Soufi, K. Khaleq, D. Hamoudi, Charlotte Garret, Matthieu Peron, Emmanuel Coron, Cédric Bretonnière, Etienne Audureau, Winters Audrey, Duvoux Christophe, Jacquelinet Christian, Azoulay Daniel, Feray Cyrille, Wissal Aissaoui, Kawtar Rghioui, Wafae Haddad, Houcine Barrou, Anna Carteaux-Taeib, Renato Lupinacci, Gilles Manceau, Florence Jeune, Christophe Tresallet, Sahar Habacha, Ines Fathallah, Aymen Zoubli, Rafaa Aloui, Nadia Kouraichi, Emilie Jouet, Julie Badin, Brice Fermier, Marc Feller, Mathieu Serie, Jérôme Pillot, William Marie, Chloé Gisbert-Mora, Camille Vinclair, Pierre Lesbordes, Pascal Mathieu, Fabienne De Brabant, Emmanuel Muller, Marie-Aline Robaux, Mikhael Giabicani, Antoine Marchalot, Stéphanie Gelinotte, Pierre Louis Declercq, Jean-Pierre Eraldi, François Bougerol, Nicolas Meunier-Beillard, Hervé Devilliers, Jean-Philippe Rigaud, Camille Verrière, Fanny Ardisson, Nancy Kentish-Barnes, Gwenaëlle Jacq, Akli Chermak, Alexandre Lautrette, Matthieu Legrand, Alexis Soummer, Guillaume Thiery, Alice Cottereau, Emmanuel Canet, Marie Caujolle, Jérôme Allyn, Dorothée Valance, Caroline Brulliard, Olivier Martinet, Julien Jabot, Thomas Gallas, David Vandroux, Nicolas Allou, Arthur Durand, Rémi Nevière, Florian Delguste, Eric Boulanger, Sebastien Preau, Ruste Martin, Hélène Cochet, Jean Pierre Ponthus, Virginie Amilien, Martial Tchir, Elise Barsam, Mohsen Ayoub, Jean Francois Georger, Izaute Guillame, Julie Assaraf, Simona Tripon, Maxime Mallet, Guilaume Barbara, Guillaume Louis, Stéphane Gaudry, Nicolas Barbarot, Angéline Jamet, Hervé Outin, Sébastien Gibot, Pierre-Edouard Bollaert, Mathilde Holleville, Stéphane Legriel, Anne Laure Chateauneuf, Sébastien Cavelot, Jean-Denis Moyer, Jean Pierre Bedos, Philippe Merle, Aurelie Laine, De Sa Natalie, Mathieu Cornuault, Jérome Libot, Karim Asehnoune, Bertrand Rozec, Jacques Dantal, Michel Videcoq, Thècle Degroote, Emmanuelle Jaillette, Farid Zerimech, Balduyck Malika, Jean-François Llitjos, Marlène Amara, Guillaume Lacave, Béatrice Pangon, José Mavinga, Joseph Nsiala Makunza, M. E. Mafuta, Yves Yanga, Amisi Eric, Jp Ilunga, Ma Kilembe, Fanny Alby-Laurent, Julie Toubiana, Amel Mokline, Achraf Laajili, Helmi Amri, Imene Rahmani, Nidhal Mensi, Lazheri Gharsallah, Sofiene Tlaili, Bahija Gasri, Rym Hammouda, Amen Allah Messadi, Pierre-Antoine Allain, Nathallie Gault, Catherine Paugam-Burtz, Arnaud Foucrier, Bassem Chatbri, Yousra Bourbiaa, Lamia Thabet, Arthur Neuschwander, Looten Vincent, Jennifer Beck, Chhor Vibol, Yavchitz Amelie, Matthieu Resche-Rigon, Jean MantzRomain Pirracchio, Côme Bureau, Maxens Decavèle, Sébastien Campion, Roukia Ainsouya, Marie-Cécile Niérat, Hélène Prodanovic, Mathieu Raux, Thomas Similowski, Bruno-Pierre Dubé, Suela Demiri, Martin Dres, Faten May, Hervé Quintard, Ilias Kounis, Faouzi Saliba, Stephane André, Marc Boudon, Philippe Ichai, Aline Younes, Lionel Nakad, Audrey Coilly, Teresa Antonini, Rodolphe Sobesky, Eleonora De Martin, Didier Samuel, Noemie Hubert, Mai-Anh Nay, Johann Auchabie, Bruno Giraudeau, Reignier Jean, Michaël Darmon, Stephane Ruckly, Maïté Garrouste-Orgeas, Elisabeth Gratia, Dany Goldgran-Toledano, Samir Jamali, Anne Sylvie Dumenil, Carole Schwebel, Laurent Brisard, Philippe Bizouarn, Thierry Lepoivre, Johanna Nicolet, Jean Christophe Rigal, Jean Christian Roussel, Cherifa Cheurfa, Julien Abily, Thomas Lescot, Isaline Page, Stéphanie Warnier, Monique Nys, Anne-Françoise Rousseau, Pierre Damas, Fabrice Uhel, Mathieu Lesouhaitier, Murielle Grégoire, Baptiste Gaudriot, Arnaud Gacouin, Yves Le Tulzo, Erwan Flecher, Karin Tarte, Jean-Marc Tadié, Quentin Georges, M. Soares, Kyeongman Jeon, Sandra Oeyen, Chin Kook Rhee, Pascale Gruber, Marlies Ostermann, Quentin Hill, Peter Depuydt, Christelle Ferra, Alice Muller, Bourmaud Aurelie, Christopher Niles, Fabien Herbert, Sylviane Pied, Séverine Loridant, Nadine François, Anne Bignon, Boualem Sendid, Caroline Lemaitre, Celine Dupre, Aymen Zayene, Lucie Portier, Nathalie De Freitas Caires, Philippe Lassalle, Aymeric Le Neindre, Pascal Selot, Daniel Ferreiro, Maria Bonarek, Stépahen Henriot, Julie Rodriguez, Mara Taddei, Mauro Di Bari, Cheryl Hickmann, Diego Castanares-Zapatero, Louise Deldicque, Peter Van Den Bergh, Gilles Caty, Jean Roeseler, Marc Francaux, Pierre-François Laterre, Bastien Dupuis, Sharam Machayeckhi, Celine Sarfati, Alex Moore, Paula Mendialdua, Emilie Rodet, Catherine Pilorge, Francois Stephan, Saida Rezaiguia-Delclaux, Jonathan Dugernier, Michel Hesse, Thibaud Jumetz, Emilie Bialais, Virginie Depoortere, Jean Bernard Michotte, Xavier Wittebole, François Jamar

**Affiliations:** 1 Réanimation polyvalente, Faculté de médecine de Sfax, Sfax, Tunisia; 2 Réanimation polyvalente, CHU Habib Bourguiba, Sfax, Tunisia; 3 ICU, CHU Habib Bourguiba, Sfax, Tunisia; 4 Anesthesia and Intensive Care, Charles Nicolle Teaching Hospital, Tunis, Tunisia; 5 Intensive care, Charles Nicolle Hospital, Tunis, Tunisia; 6 Anesthésie réanimation, CHU Ibn Rochd Casa, Casablanca, Morocco; 7 Reanimation des urgences chirurgicale, Chu Ibn Rochd, Casablanca, Morocco; 8 Service de réanimation des urgences chirurgicales, CHU IBN ROCHD de Casablanca, Casablanca, Morocco; 9 Service d’accueil des urgences, Chu Ibn Rochd, Casablanca, Morocco; 10 Pôle de réanimation, hôpital salengro, C.H.R.U. - Lille, Avenue Oscar Lambret, Lille, France; 11 Centre de biologie pathologie génétique, Centre Hospitalier Régional Universitaire de Lille, Lille, France; 12 Inserm cic 1435/urgences/samu, Centre Hospitalier Universitaire de Limoges, Limoges, France; 13 Services urgences adultes/samu-smur/inserm cic1435, C.H.U de Limoges, Limoges, France; 14 Service de réanimation polyvalente, Centre Hospitalier Universitaire de Limoges, Limoges, France; 15 Inserm cic 1435/réanimation polyvalente, C.H.U de Limoges, Limoges, France; 16 Urgences/samu, Centre Hospitalier Universitaire de Limoges, Limoges, France; 17 Service urgences adultes/samu-smur, C.H.U de Limoges, Limoges, France; 18 Service de maladies infectieuses, Centre Hospitalier Universitaire de Limoges, Limoges, France; 19 Bactériologie-virologie-hygiène/umr-s 1092, Centre Hospitalier Universitaire de Limoges, Limoges, France; 20 Medical icu, Hospital Abderrahmen Mami De Pneumo-Phtisiologie, Ariana, Tunisia; 21 Réanimation médicale, Hôpital Abderrahmen Mami, Ariana, Tunisia; 22 Department of intensive care and toxicology, Centre d’Assistance Médicale Urgente, Tunis, Tunisia; 23 Service de réanimation médicale, Centre d’assistance médicale-urgente, Tunis, Tunisia; 24 Reanimation, Centre Hôspitalier Henri Duffaut, Avignon, France; 25 Interne en anesthésie réanimation, C.H. Régional Universitaire de Lille (CHRU de Lille), Lille, France; 26 Réanimation polyvalente, Hospital Center De Dunkerque, Dunkerque, France; 27 Equipe opérationnelle d’hygiene, Hospital Center De Dunkerque, Dunkerque, France; 28 Service de chirurgie digestive, Hospital Center De Dunkerque, Dunkerque, France; 29 Laboratoire, Hospital Center De Dunkerque, Dunkerque, France; 30 Service des urgences viscérales, CHU Ibn Rochd de Casablanca, Casablanca, Morocco; 31 Service de microbiologie, CHU Ibn Rochd de Casablanca, Casablanca, Morocco; 32 Faculté de médecine de Sfax, Sfax, Tunisia; 33 Hopital régional mahres, Faculté de médecine de Sfax, Sfax, Tunisia; 34 Intensive care, Hopital régional Gafsa, Sfax, Tunisia; 35 Intensive care, Hopital régional mahres, Sfax, Tunisia; 36 Anesthesia service surgical resuscitation, Chu ibn rochd, Casablanca, Morocco; 37 Thoracic surgery, Chu ibn rochd, Casablanca, Morocco; 38 Anesthesiology and Intensive Care Department, University Teaching Hospital Ibn Rushd-Casablanca, Casablanca, Morocco; 39 Intensive Care Unit, IBN Rochd, Casablanca, Morocco; 40 Réanimation, Groupe Hospitalier Paris Saint-Joseph, Paris, France; 41 Unité de microbiologie clinique et dosages des anti-infectieux, Groupe Hospitalier Paris Saint-Joseph, Paris, France; 42 Réanimation polyvalente adulte, Centre Hospitalier Intercommunal André Grégoire, Montreuil, France; 43 Iame team 5, INSERM UMR 1137, Paris, France; 44 Hygiène hospitalière, Hôpital Bichat-Claude Bernard (AP-HP), Paris, France; 45 Réanimation, Hospices Civils De Lyon, Lyon, France; 46 Médecine interne, Hôpital Bichat-Claude Bernard (AP-HP), Paris, France; 47 Hygiène hospitalière, C.H.U de Rennes, Rennes, France; 48 Pharmacie, CHU - Hôpitaux de Bordeaux, Bordeaux, France; 49 Réanimation, CHRU Nancy, Nancy, France; 50 Dgos, Ministère des Affaires sociales et de la Santé, Paris, France; 51 Cclin sud est, Hospices Civils De Lyon, Lyon, France; 52 Cclin, Hospices Civils De Lyon, Lyon, France; 53 Réanimation médicale et infectieuse, Hôpital Bichat-Claude Bernard, Paris, France; 54 Department of Intensive Care Medicine and Infectious Diseases, Hôpital Bichat-Claude Bernard-APHP, Paris, France; 55 Réanimation Médicale, Hôpital Henri Mondor, Créteil, France; 56 Cepi, Hospital Henri Mondor, Créteil, France; 57 Microbiologie, Hôpital Henri Mondor, Créteil, France; 58 Department of Biology, Centre d’Assistance Médicale Urgente, Tunis, Tunisia; 59 Service de Réanimation médicale et Médecine hyperbare, Centre Hospitalier Universitaire d’Angers, Angers, France; 60 Réanimation médicale, Centre Hospitalier Universitaire d’Angers, Angers, France; 61 Département d’anesthésie-réanimation, Hôpital Beaujon, Boulevard du Général Leclerc, Clichy, France; 62 Anesthesiology and Critical Care, Hospital Center university Beaujon (AP-HP), Clichy, France; 63 Réanimation médicale, CHU de Strasbourg, Strasbourg, France; 64 Laboratoire de bacteriologie-virologie-hygiene, Hôpital Avicenne, Bobigny, France; 65 Réanimation médico-chirurgicale, Assistance Publique - Hôpitaux de ParisHôpital Ambroise Paré, Boulogne-Billancourt, France; 66 Equipe opérationnelle d’hygiène hospitalière, Assistance Publique - Hôpitaux de Paris, Hôpital Ambroise Paré, Boulogne-Billancourt, France; 67 Service de microbiologie, Assistance Publique - Hôpitaux de Paris, Hôpital Ambroise Paré, Boulogne-Billancourt, France; 68 Equipe mobile de microbiologie, Assistance Publique - Hôpitaux de Paris, Hôpital Ambroise Paré, Boulogne-Billancourt, France; 69 Service de réanimation médicale, Hôpital Cochin, Paris, France; 70 Intensive Care Unit, Hospital, Cayenne, French Guiana; 71 Department of Critical Care Medicine and Anesthesiology, Military Hospital of Tunis, Tunis, Tunisia; 72 Réanimation polyvalente, CHU Fatouma Bourguiba, Monastir, Tunisia; 73 Réanimation, Centre Hospitalier Métropole-Savoie, Chambéry, France; 74 Réanimation, Hôpital d’Instruction des Armées Percy, Clamart, France; 75 Département d’anesthésie-réanimation, Hôpital d’Instruction des Armées Percy, Clamart, France; 76 Réanimation respiratoire, Hôpital Abderrahmen Mami de pneumo-phtisiologie, Ariana, Tunisia; 77 Réanimation polyvalente, Hopital Rangueil, Avenue du Professeur Jean Poulhes, Toulouse, France; 78 RPPFHopital de la Timone, Marseille, France; 79 Réanimation médicale, CHU Farhat Hached, Sousse, Tunisia; 80 Réanimation médicale, CHU Farhat Hached. Research Laboratory N° LR14ES05. Faculty of Medicine, Sousse, Tunisia; 81 Medical Intensive Care Unit, Farhat Hached Hospital. Research Laboratory N° LR14ES05. Faculty of Medicine, Sousse, Tunisia; 82 Hospital Hygiene Unit, Farhat Hached Hospital, Sousse, Tunisia; 83 Service de réanimation, Centre d’assistance médicale-urgente, Tunis, Tunisia; 84 Department of emergency, Centre d’Assistance Médicale Urgente, Tunis, Tunisia; 85 Anesthesiology and Intensive Care Department, University Teaching Hospital Ibn Rushd-Casablanca, Casablanca, Morocco; 86 Medical Intensive Care Unit, Mohamed V University Hopital Ibn Sina, Rabat, Morocco; 87 Service des urgences médicales hospitalières - ibn sina – université mohamed v – rabat, Hopital Ibn Sina, Rabat, Morocco; 88 Réanimation médicale bizerte, Faculté de médecine de Tunis, Bizerte, Tunisia; 89 Intensive Care Unit, King Hamad University Hospital, Muharraq, Bahrain; 90 Réanimation Médicale, EPS Taher Sfar Mahdia, Mahdia, Tunisia; 91 Pharmacy Unit, C.H. Chalon sur Saône William Morey, Chalon-sur-Saône, France; 92 Intensive Care Unit, C.H. Chalon sur Saône William Morey, Chalon-sur-Saône, France; 93 Anesthésie réanimation, Chu Ibn Rochd, Casablanca, Morocco; 94 Réanimation médicale, Centre Hospitalier Universitaire, Amiens, France; 95 Hématologie clinique et thérapie cellulaire, Centre Hospitalier Universitaire, Amiens, France; 96 Intensive Care and Thoracic Oncology, Institute Jules Bordet, Brussel, Belgium; 97 Data Centre, Institute Jules Bordet, Brussels, Belgium; 98 Réanimation médicale, C.H.R.U. Hôpitaux Universitaires Strasbourg, Strasbourg, France; 99 Département d’oncologie et d’hématologie, C.H.R.U. Hôpitaux Universitaires Strasbourg, Strasbourg, France; 100 Service de réanimation médicale, Hôpital de Hautepierre du C.H.R.U, Avenue Molière, Strasbourg, France; 101 Service de réanimation médicale, Hôpital de Hautepierre, Hôpitaux Universitaires de Strasbourg, Strasbourg, France; 102 Hautepierre réanimation médicale, C.H.R.U. Hôpitaux Universitaires Strasbourg, Strasbourg, France; 103 Surgical Intensive Care, Hospital Henri Mondor, Créteil, France; 104 Anesthesia and surgical intensive care, Hospital Henri Mondor, Créteil, France; 105 Anesthesia and Intensive Care Medicine, CHU Henri Mondor, Créteil, France; 106 Service d’accueil des urgences, Hôpitaux Universitaires de Strasbourg, Strasbourg, France; 107 Département d’information médicale, C.H.R.U. Hôpitaux Universitaires Strasbourg, Strasbourg, France; 108 Réanimation, Etablissement hospitalier spécialisé 1er novembre, Oran, Algeria; 109 Anesthésie réanimation chirurgicale, EHU 1er Novembre, Oran, Algeria; 110 Réanimation pédiatrique, Centre Hospitalier et Universitaire d’Oran, Oran, Algeria; 111 Anesthesie -réanimation chirurgicale, EHS 1er Novembre, oran, Algeria; 112 Service de gynéco-obstétrique, EHS 1er Novembre, Oran, Algeria; 113 Cardiologie, Centre Hospitalier et Universitaire d’Oran, Oran, Algeria; 114 Réanimation surveillance continue, Hôpital Privé Gériatrique Les Sources, Nice, France; 115 Pneumologie, CHU Farhat Hached, Sousse, Tunisia; 116 Intensive Care Unit and Anesthesiology, Hôpital d’Instruction des Armées Sainte-Anne, Toulon, France; 117 Intensive Care Unit and Anesthesiology, Hôpital d’Instruction des Armées Laveran, Marseille, France; 118 Anesthésie réanimation, Hôpital Médico Chirurgical Bouffard, Djibouti, Djibouti; 119 ICU, C.H. Epicura Hornu, Hornu, Belgium; 120 Laboratoire de l’effort et du mouvement, Condorcet, Tournai, Belgium; 121 Irec, pôle de pneumologie, ucl, Cliniques Universitaires Saint Luc, Bruxelles, Belgium; 122 Service de pneumologie, Cliniques Universitaires Saint Luc, Bruxelles, Belgium; 123 Service de Réanimation médicale, CHU de Poitiers, Poitiers, France; 124 Neurophysiology, CHU de Poitiers, Poitiers, France; 125 Pneumologie, CHU de Poitiers, Poitiers, France; 126 SFAX, CHU HABIB BOURGUIBA, Sfax, Tunisia; 127 Réanimation chirurgicale polyvalente, Groupe Hospitalier Pitié-Salpêtrière, Paris, France; 128 Hépato-gastro-entérologie et médecine de la transplantation, Groupe Hospitalier Pitié-Salpêtrière, Paris, France; 129 Chirurgie digestivehépato-bilio-pancréatique et transplantation hépatique, Groupe Hospitalier Pitié-Salpêtrière, Paris, France; 130 Réanimation Médicale, Hôpital Henri Mondor, Avenue du Maréchal de Lattre de Tassigny, Créteil, France; 131 Réanimation Médicale, Hopital regional zaghouan, Faculté de médecine de Tunis, Zaghouan, Tunisia; 132 Teaching Department of Emergency and Intensive Care, Regional hospital of Zaghouan, Zaghouan, Tunisia; 133 Département d’anesthésie réanimation, CHU Ibn Rochd Casa, Casablanca, Morocco; 134 Anesthésie réanimation, Hopital 20 Aout CHU IBN Rochd, Casablanca, Morocco; 135 Néphrologie hémodialyse et transplantation rénale, CHU Ibn Rochd, Casablanca, Morocco; 136 Nephrology, C.H.U de Liège - Sart Tilman, Liège, Belgium; 137 Nephrology, Hospital Center University De Saint-Étienne, Saint-Priest-en-Jarez, France; 138 Medical Intensive Care, C.H.U de Liège - Sart Tilman, Liège, Belgium; 139 Néphrologie, Centre Hospitalier Universitaire de Saint-Étienne, Saint-Étienne, France; 140 Biologie cliniqueC.H.U de Liège - Sart Tilman, Liège, Belgium; 141 C.H.U de Liège - Sart Tilman, Liège, Belgium; 142 URGENCES MEDICALES CHUORAN, Faculté de médecine d’Oran, Oran, Algeria; 143 Emergencies, CHU ORAN, Oran, Algeria; 144 Urgences medicaleschu oran, oran, Algeria; 145 Urgences Médicales et réanimation, Centre Hospitalier et Universitaire d’Oran, Oran, Algeria; 146 Réanimation polyvalente, C.H. Sud Francilien, Corbeil-Essonnes, France; 147 Samu, C.H. Sud Francilien, Corbeil-Essonnes, France; 148 Departamento de cuidados intensivos, Hospital Universitario de Getafe, Getafe, Spain; 149 Departamento de anatomía patología, Hospital Universitario de Getafe, Getafe, Spain; 150 Réanimation médicale, Hospital Croix-Rousse, Lyon, France; 151 Positron emission tomography department, CERMEP, Bron, France; 152 Service d’anatomo-pathologie, Hospital Croix-Rousse, Lyon, France; 153 Reanimation des urgences et medicale, CHU la Timone 2, Marseille, France; 154 Réanimation des détresses respiratoires et infections sévères, Hôpital Nord APHM, Toulon, France; 155 Service de réanimation des urgences et médicale, Hôpital Timone Adulte, Marseille, France; 156 Réanimation des détresses respiratoires et infections sévères, Hôpital Nord APHM, Marseille, France; 157 Service de réanimation-détresses respiratoires et infections sévères, Hôpital Nord, Marseille, France; 158 Réanimation polyvalente, Hôpital Sainte Musse, Toulon, France; 159 Sar 2, réanimation médico-chirurgicale magellan, CHU de Bordeaux, Bordeaux, France; 160 Departement des urgences, C.H. Annecy Genevois, Annecy, France; 161 Réanimation, Centre Hospitalier la Rochelle, La Rochelle, France; 162 Réanimation, Groupement Hospitalier La Rochelle Ré Aunis, La Rochelle, France; 163 Clinical Psychology Department, Centre Hospitalier la Rochelle, La Rochelle, France; 164 Clinical Research Unit, Centre Hospitalier la Rochelle, La Rochelle, France; 165 Médecine interne, Groupe Hospitalier Paris Saint-Joseph, Paris, France; 166 Psychology, University of Bourgogne Franche-Comté, Besançon, France; 167 Icu, Policlinico A. Gemelli, Rome, Italy; 168 Icu, Hospital de Sabadell, Madrid, Spain; 169 Icu, CHUM, Montreal, Canada; 170 Icu, Policlinico Universitario A. Gemelli, Università Cattolica Del Sacro Cuore, Rome, Italy; 171 Unité de méthodologie et de qualité de vie en cancérologie, CHRU de Besançon, Boulevard Alexandre Fleming, Besançon, France; 172 Réanimation polyvalente, Hospitat Sant Pau, Barcelone, Spain; 173 Réanimation chirurgicale, CHU de Besançon, Besançon, France; 174 Réanimation Médicale, CHU de Besançon, Besançon, France; 175 Department of Medical and Toxicological Critical Care, Lariboisière Hospital, Paris, France; 176 Laboratory of Toxicology, Lariboisière Hospital, Paris, France; 177 Réanimation médicale et toxicologique, Hôpital Lariboisière (AP-HP), Paris Cedex 10, France; 178 Service de Réanimation Médicale et Toxicologique, CHU Lariboisière, Paris, France; 179 Inserm u1144, Paris-Descartes University, Paris, France; 180 Centre Antipoison, C.H.U. d’Angers, Angers, France; 181 Department of Physiological Investigations, Lariboisière Hospital, Paris, France; 182 Médecins Sans Frontières, Geneva, Switzerland; 183 Réanimation polyvalente, SAMU de Paris, Paris, France; 184 Assistance Publique Hôpitaux de Paris, Paris, France; 185 Médecins Sans Frontières, 2, rue saint-sabin, Paris, France; 186 Service de réanimation médicaleinserm umr s_999, université paris-sudHôpital de bicêtre, hôpitaux universitaires paris-sud, Assistance publique – Hôpitaux de Paris, Le Kremlin-Bicêtre, France; 187 ALAM Medical, Vincennes, France; 188 Otolaryngology-Head and Neck Surgery, Shaare-Zedek Medical Center and Hebrew University Medical School, Jerusalem, Israel; 189 Réanimation médicale, CHU de Bicêtre, Le Kremlin-Bicêtre, France; 190 Service de physiologieinserm umr s_999, université paris-sudHôpital de bicêtre, hôpitaux universitaires paris-sud, Assistance publique – Hôpitaux de Paris, Le Kremlin-Bicêtre, France; 191 Internal Medicine, Hospital André Vésale, Montigny-le-Tilleul, Belgium; 192 Biologie clinique, Hôpital Civil Marie Curie, Charleroi, Belgium; 193 Réanimation polyvalente, Hôpital Civil Charleroi, Charleroi, Belgium; 194 Reanimation, EHS CANASTEL, Oran, Algeria; 195 Medical Imaging Department, Charles Nicolle Teaching Hospital, Tunis, Tunisia; 196 Anesthesiology and Intensive Care, Caddi Ayyad University Mohammed VI Teaching Hospital, Marrakesh, Morocco; 197 Cardio Vascular Surgery, Caddi Ayyad University Mohammed VI Teaching Hospital, Marrakesh, Morocco; 198 Anesthesiology, Centre Hospitalier Universitaire de Caen, Caen, France; 199 Medical Intensive Care, Centre Hospitalier Universitaire de Caen, Caen, France; 200 Thoracic and Cardiovascular Surgery, Centre Hospitalier Universitaire de Caen, Caen, France; 201 Réanimation médicale, Centre Hospitalier Universitaire de Caen, Caen, France; 202 Cardiologie, Hôpital Européen Georges-Pompidou, Rue Leblanc, Paris, France; 203 Réanimation médicale, Hopital Europeen Georges-Pompidou, Paris, France; 204 Réanimation adulte, Hôpital Necker - Enfants Malades, Paris, France; 205 B.s.p.p., Paris, France; 206 Cardiologie, Pitié-Salpêtrière Hospital, Paris, France; 207 Paris descartesInserm U970, Paris, France; 208 Département d’anesthésie et de réanimation, Hôpital Universitaire La Pitié-Salpêtrière, Paris, France; 209 Service d’accueil des urgences, Hôpital Cochin, Paris, France; 210 Cardiologie, Hôpital Européen Georges-Pompidou, Paris, France; 211 Hépato-gastro-entérologie, Hôpital Saint-Antoine, Paris, France; 212 Urgences, Hôpital Européen Georges-Pompidou (AP-HP), Paris, France; 213 Samu92, Hôpital Raymond-Poincaré (AP-HP), Garches, France; 214 Samu-smur 93, Hôpital Avicenne, Bobigny, France; 215 Samu94, Hôpital Henri Mondor, Créteil, France; 216 Pediatric Intensive Care Unit, Hôpital Jeanne de Flandre PARKING, Lille, France; 217 French National out-of-Hospital Cardiac Arrest Registry (réac), Lille, France; 218 Public Health Department, Université Lille - Faculté de Médecine Henri Warembourg, Loos, France; 219 Public Health Department, Université Lille - Faculté de Médecine Henri Warembourg, Lille, France; 220 Réanimation pédiatrique, Centre Hospitalier Régional Universitaire de Lille, Lille, France; 221 Service de réanimation pédiatrique, CHRU de Lille, Lille, France; 222 Medical Intensive Care Unit, Hospital Center University Hospital, Rouen, France; 223 Department of Clinical Research Support, Biostatistics Unit, Hospital Center University Hospital, Rouen, France; 224 Réanimation médicale, Centre Hospitalier Universitaire Rouen, Rouen, France; 225 Intensive Care, Hospital Center University Rouen, Rouen, France; 226 Réanimation médicale, Hôpital Saint-Louis, Paris, France; 227 Service de biostatistique et information médicale, Hôpital Saint-Louis, Paris, France; 228 Réanimation, Institut Paoli-Calmettes, Marseille, France; 229 Réanimation médicale, Hôpital Pitié-Salpêtrière, Paris, France; 230 Unité de réanimation et de surveillance continueservice de pneumologie et réanimation médicale, Pitié-Salpêtrière Hospital, Paris, France; 231 Service de pneumologie et réanimation médicale, Hôpital Pitié-Salpêtrière, Paris, France; 232 Réanimation polyvalente, Centre Hospitalier de Roubaix, Roubaix, France; 233 Réanimation polyvalente, C.H.U. de Nancy, Nancy, France; 234 59C.H. Régional Universitaire de Lille (CHRU de Lille), Lille, France; 235 Service de réanimation médicale, C.H. Régional Universitaire de Lille, Lille, France; 236 Centre de réanimation, C.H. Régional Universitaire de Lille (CHRU de Lille), Lille, France; 237 Service de réanimation médico-chirurgicale, Centre Hospitalier de Versailles, Le Chesnay, France; 238 Réanimation polyvalente, Groupe Hospitalier Intercommunal Le Raincy-Montfermeil, Montfermeil, France; 239 Service de réanimation, CHU Lapeyronie, Montpellier, France; 240 Réanimation, Centre Hospitalier Départemental - site de La Roche-sur-Yon, La Roche-sur-Yon, France; 241 Réanimation médicale, Chu Nantes, Nantes, France; 242 Service de réanimation médicale, CHU Hôtel-Dieu Nantes, Nantes, France; 243 Réanimation pneumologique, Hôpital Cochin, Paris, France; 244 Département de biostatistiques et d’epidémiologie, CHU de Poitiers, Poitiers, France; 245 Réanimation adulte, C.H.U. Estaing, Clermont-Ferrand, France; 246 Réanimation médicale, CHRU de Brest, Brest, France; 247 Réanimation médicale polyvalente, Hôpital de La Source, CHR Orléans, Orléans, France; 248 Service de pneumologie et réanimation médicale, Groupe Hospitalier Pitié-Salpêtrière, Paris, France; 249 Réanimation médicale, inserm umr_s 1158 neurophysiologie respiratoire expérimentale et clinique, Pitié-Salpêtrière Hospital, Paris, France; 250 Hospital Foch, 92151 Suresnes, France; 251 Centre de Réanimation, Centre Hospitalier Régional Universitaire de Lille, Lille, France; 252 Service de réanimation médicale, Hospices Civils de Lyon - Groupement Hospitalier Edouard Herriot, Lyon, France; 253 Réanimation, Centre Hospitalier Général de Roanne, Roanne, France; 254 Service de Réanimation Médico-Chirurgicale, CHU Louis Mourier, Colombes, France; 255 Urgences adultes, Hospital Center Regional University, Brest, France; 256 Urgences, CHU de Poitiers, Poitiers, France; 257 Laboratoire des usages, Irt B-Com, Plouzané, France; 258 Réanimation ctcv transplantation thoracique, CHU de Nantes - Hôpital Nord Laennec, Saint-Herblain, France; 259 Réanimation, CHU de Strasbourg, Strasbourg, France; 260 Réanimation médicale, Nouvel Hôpital Civil, Hôpitaux Universitaires de Strasbourg, Strasbourg, France; 261 Service de réanimation polyvalente, Hôpital Européen Georges-Pompidou (AP-HP), Paris, France; 262 Réanimation polyvalente, Centre Hospitalier Régional Universitaire de Tours, Tours, France; 263 Biostatistiques, C.H.U. Hôtel Dieu, Nantes, France; 264 Unité de recherche clinique, Centre Hospitalier Départemental - site de La Roche-sur-Yon, La Roche-sur-Yon, France; 265 Réanimation polyvalente, Centre Hospitalier Départemental - site de La Roche-sur-Yon, La Roche-sur-Yon, France; 266 DAR B, Hôpital Saint Eloi, Montpellier, France; 267 Réanimation médicale, Hôpital Saint-Antoine, Paris, France; 268 Service de reanimation médicale, Hôpital Saint-Antoine, Paris, France; 269 Unité 1016, Institut National de la Santé et de la Recherche Médicale, 2rue méchain, 75014 Paris, France; 270 Service de réanimation polyvalente, Centre Hospitalier de Versailles, Le Chesnay, France; 271 Service de réanimation médicale, Hôpital Cochin, Assistance Publique Hôpitaux de Paris, Paris, France; 272 Emergency and Intensive Care Department, Hôpital Babib Thameur, Tunis, Tunisia; 273 Réanimation, C.H.U. d’Angers, Angers, France; 274 Réanimation, C.H. - Le Mans, Le Mans, France; 275 Réanimation chirurgicale, C.H.U. d’Angers, Angers, France; 276 Réanimation polyvalente, Hospital Center Departmental De Vendée, La Roche-sur-Yon, France; 277 Réanimation medico-chirurgicale, Hopital avicenne, Bobigny, France; 278 Réanimation, C.H. de Saint Brieuc, Saint-Brieuc, France; 279 Réanimation médicale, C.H.U. Hôtel Dieu, Nantes, France; 280 Réanimation, C.H. Chalon sur Saône William Morey, Chalon-sur-Saône, France; 281 Réanimation polyvalente, Hôpital Louis-Mourier (AP-HP), Colombes cedex, France; 282 Inserm, iame, umr 1137, Université Paris Diderot, Sorbonne Paris Cité, Paris, France; 283 Institut für anästhesiologische pathophysiologie und verfahrensentwicklung, Universitätsklinikum Ulm, Ulm, Germany; 284 Réanimation polyvalente, CHD de Vendée, La Roche-sur-Yon, France; 285 Réanimation polyvalente, CHRU de Tours, Tours, France; 286 Réanimation médicale, CHU de Dijon, Dijon, France; 287 Réanimation médicale polyvalente, CHR d’Orléans, Orléans, France; 288 Réanimation polyvalente, CH D’Angoulême, Angoulême, France; 289 Réanimation, CH de Montauban, Montauban, France; 290 Réanimation polyvalente, CH Victor Dupouy, Argenteuil, France; 291 Intensive Care, Chu Dijon, Dijon, France; 292 Inserm cic 1415, CHRU de Tours, Tours, France; 293 Réanimation polyvalente, CHRU Hôpitaux de Tours, Tours, France; 294 Réanimation médico-chirurgicale, C.H. - Le Mans, Le Mans, France; 295 Laboratoire de biochimie, C.H.U. d’Angers, Angers, France; 296 Département de physiologie et radio-isotopes, Hôpital Européen Georges-Pompidou (AP-HP), Paris, France; 297 Réanimation polyvalente, CHU Bretonneau, Tours, France; 298 Service de réanimation chirurgicale, Hôpital Guillaume et René Laënnec, CHU de Nantes, Nantes, France; 299 Réanimation médico-chirurgicale, Hôpital Louis-Mourier - APHP, Colombes, France; 300 Service de réanimation médico-chirurgicale, CHU Louis Mourier, Colombes, France; 301 Réanimation, C.H. Général Saint Denis hôpital Delafontaine, Saint-Denis, France; 302 Réanimation médico-chirurgicale, René Dubos, Pontoise, France; 303 Réanimation polyvalente, Hôpital Antoine Béclère, Clamart, France; 304 Réanimation médico-chirurgicale, René Dobus, Pontoise, France; 305 Service de réanimation médicale, Groupe Hospitalier La Pitié-Salpêtrière, Institut de Cardiométabolisme et Nutrition, Assistance Publique Hôpitaux de Paris, Paris, France; 306 Service de réanimation médicale, Groupe Hospitalier Pitié-Salpêtrière, Institut de Cardiométabolisme et Nutrition, Paris, France; 307 Département de réanimation médicale et de médecine hyperbare, C.H.U. d’Angers, Angers, France; 308 Service de réanimation médicale, C.H. Lyon Sud, Pierre-Bénite, France; 309 Service de réanimation médicale, Clinique de Réanimation Médicale, Grenoble, France; 310 Service de réanimation polyvalente, C.H. de Dax - Côte d’Argent, Dax, France; 311 Service de réanimation médicale et infectieuse, Hôpital Bichat-Claude Bernard, Assistance Publique Hôpitaux de Paris, Paris, France; 312 Service de réanimation chirurgicale, Hopital Louis Pradel, Bron, France; 313 Service de réanimation polyvalente, Centre Hospitalier Bretagne Atlantique, Vannes, France; 314 Service de réanimation adulte, Hôpital Necker, Assistance Publique Hôpitaux de Paris, Paris, France; 315 Service de médecine interne, Groupe Hospitalier La Pitié-Salpêtrière, Institut IE3M, Assistance Publique Hôpitaux de Paris, Paris, France; 316 Laboratoire d’hémostase, Hopital Rangueil, Toulouse, France; 317 Chirurgie cardio-vasculaire, Hopital Rangueil, Toulouse, France; 318 Réanimation purpan, CHU Toulouse, Toulouse, France; 319 Cardiologie, Hopital Rangueil, Toulouse, France; 320 Service de chirurgie thoracique et cardiovasculaire, Groupe Hospitalier Pitié Salpêtrière, Paris, France; 321 CHU Toulouse, Toulouse, France; 322 Cardiology, Hopital Rangueil, Toulouse, France; 323 Réanimation, Centre Hospitalier Universitaire Toulouse, Toulouse, France; 324 Department of Anesthesia and Critical Care, Chu-Hôpitaux De Rouen, Rouen, France; 325 Department of Anesthesia and Critical Care, Centre Hospitalier Universitaire Rouen, Rouen, France; 326 Reanimation chirurgicale, Centre Hospitalier Universitaire Rouen, Rouen, France; 327 Department of Cardiac Surgery, Chu-Hôpitaux De Rouen, Rouen, France; 328 Anesthésie, Centre Hospitalier Universitaire Rouen, Rouen, France; 329 Physiology, Hôpital Bichat-Claude Bernard-AP-HP, Paris, France; 330 Réanimation Médicale et Infectieuse, GH Bichat Claude Bernard, Paris, France; 331 Hepatology and Gastroenterology, Pitié-Salpêtrière Hospital, Paris, France; 332 Unité de réanimation neurologique, Hôpital Pitié-Salpêtrière, Paris, France; 333 Bioinformatics and Biostatistics Platform, ICM Institut du Cerveau et de la Moelle épinière, Paris, France; 334 Neurological Icu, Pitié-Salpêtrière Hospital, Paris, France; 335 Brain Liver Pitié-Salpêtrière Study Group (BLIPS), Hôpital Pitié-Salpêtrière, Paris, France; 336 Réanimation médicale, Centre hospitalier universitaire la Rabta, Tunis, Tunisia; 337 Maladies infectieuses, Centre Hospitalier universitaire la Rabta, Tunis, Tunisia; 338 Département de médecine d’urgence, Hospital Trousseau, Chambray-lès-Tours, France; 339 Service d’information médicaleepidémiologie et economie de la santé, CHRU Hôpitaux de Tours, Tours, France; 340 Intensive Care Unit, C.H.U La Meynard, Fort de France, Martinique; 341 Department of infectious & tropical diseases, C.H.U La Meynard, Fort de France, Martinique; 342 13, Hôpital Timone adulte, Marseille, France; 343 Service de neurophysiologie clinique, Hôpital Timone Adulte, Marseille, France; 344 Pédiatrie, Hôpital Robert-Debré (AP-HP), Paris, France; 345 Réanimation et surveillance continue pédiatriques, Hôpital Robert Debré, Paris, France; 346 Réanimation et surveillance continue pédiatriques, CHU Robert Debré, Paris, France; 347 Paediatric Intensive Care Unit, CHU Sainte-JustineChemin de la Côte-Sainte-Catherine, Montreal, QC Canada; 348 Anesthésie réanimation, Hôpital Louis Pradel, Bron, France; 349 Réanimation pédiatrique, Hôpital Femme Mère Enfant, Bron, Lyon, France; 350 Réanimation pédiatrique hfme, Hospices civils de Lyon, Lyon, France; 351 Réanimation médicale, Hôpital de la Croix-Rousse, Lyon, France; 352 Service de pédiatrie, CH d’Armentières, Armentières, France; 353 Service de biostatistiques, CHRU Lille, Lille, France; 354 Service de neurochirurgie pédiatrique, CHRU de Lille, Lille, France; 355 69, Hôpital Femme Mère Enfant, Bron, France; 356 01, Hospital Center Fleyriat, Bourg-en-Bresse, France; 357 69, Edouard Herriot Hospital, Lyon, France; 358 67, University De Strasbourg - Campus Médecine, Strasbourg, France; 359 Réanimation pédiatrique canastel, Faculté de médecine d’Oran, Oran, Algeria; 360 Réanimation pédiatrique de Canastel d’oran, Departement de medecine d’Oran Algerie, Oran, Algeria; 361 Anesthésie réanimation pédiatrique, Etablissement hospitalier spécialisé en pédiatrie Canastel, Oran, Algeria; 362 Service de réanimation pédiatrique, Chu Ibn Rochd, Casablanca, Morocco; 363 Réanimation pédiatrique, Hôpital Clocheville, Centre Hospitalier Universitaire Tours, Tours, France; 364 Chirurgie pédiatrique de la tête et du cou, Hôpital Clocheville, Centre Hospitalier Universitaire Tours, Tours, France; 365 Fondation Ophtalmologique Adolphe de Rothschild, Paris, France; 366 Réanimation neurochirurgicale pédiatrique, Hospital Necker, Paris, France; 367 Agence de Biomédecine, Saint-Denis, France; 368 Service de régulation et d’appui, Agence biomedecine, Malakoff, France; 369 réanimation néonatale et pédiatrique, Hopital pour enfants Trousseau, Paris, France; 370 Saint Louis, Paris, France; 371 Hématologie, Hôpital Saint Louis, Paris, France; 372 Service de réanimation médicale et infectieuse, Hôpital Bichat-Claude Bernard-APHP, Paris, France; 373 Réanimation médicale, Saint Louis, Paris, France; 374 Service de réanimation médicale, Hôpital Saint-Louis (AP-HP), Paris, France; 375 Anesthésie-réanimation, C.H.R.U. Hôpitaux Universitaires Strasbourg, Strasbourg, France; 376 Unité de parasitologie-mycologie, département de virologie, bactériologie-hygiène, parasitologie, Hopital henri mondor, Créteil, France; 377 Inserm, u955, equipe 13, équipe biomécanique cellulaire et respiratoire, Université Paris-Est Créteil - Faculté de médecine, Créteil, France; 378 Reanimation, Institute Paoli-Calmettes, Marseille, France; 379 Réanimation médicale, Hôpital Bichat-Claude Bernard (AP-HP), Paris, France; 380 Réanimation médicale, Pitié-Salpêtrière Hospital, Paris, France; 381 Département d’anesthésie réanimation, GH St Louis/Lariboisière AP-HP, Paris, France; 382 Urc, Hospital Ambroise Paré, Boulogne-Billancourt, France; 383 Réanimation médico-chirurgicale, Hôpital Tenon, Paris, France; 384 Réanimation polyvalente, CHI Poissy-St Germain, Poissy, France; 385 Réanimation polyvalente, Centre Hospitalier Sud Francilien, Boulevard Jean Jaurès, Corbeil-Essonnes, France; 386 Intensive Care Unit, Hospital Center De Versailles, Le Chesnay, France; 387 Hépato-gastroentérologie, CHU Hôtel-Dieu Nantes, Nantes, France; 388 Service de santé publique, ap-hp hôpital henri mondor ea7376 cepia, université paris est crétei, ap-hp hôpital henri mondor, Créteil, France; 389 Upres ea 2415 département de biostatistiques, Université de Montpellier, Montpellier, France; 390 Service d’hépatologie, Hôpital Henri Mondor, Créteil, France; 391 Aence de la biomédecine, Agence de la biomédecine, Saint-Denis, France; 392 Service de chirurgie digestive et transplantation hépatique, Hôpital Henri Mondor, Créteil, France; 393 Chirurgie digestive, Groupe Hospitalier Pitié-Salpêtrière, Paris, France; 394 Chirurgie digestive et hépato-bilaire, Groupe Hospitalier Pitié-Salpêtrière, Paris, France; 395 Intensive Care Unit, Regional Hospital of Ben Arous, Tunis, Tunisia; 396 Departement of Intensive Care and Toxicology, Centre d’Assistance Médicale Urgente, Tunis, Tunisia; 397 41C.H. de Blois, Blois, France; 398 Réanimation polyvalente - usc, Centre Hospitalier de la Côte Basque, Bayonne, France; 399 Intensive Care, Dieppe General Hospital, Dieppe, France; 400 Umr 7366 cnrs, University of Burgundy, Dijon, France; 401 Internal Medicine, Chu Dijon, Dijon, France; 402 Hôpital Saint-Louis (AP-HP), Paris, France; 403 Réanimation médicale, Assistance Publique Hôpitaux de Paris, Hôpital Saint Louis, Paris, France; 404 Réanimation, C.H. de Versailles, Le Chesnay, France; 405 Réanimation polyvalente, CH sud Essonne, Paris, France; 406 Réanimation médicale, CHU Gabriel-Montpied, Clermont-Ferrand, France; 407 Anesthésie réanimation et traitement chirurgical des grands brûlés, APHP - Hôpital Saint-Louis, Lariboisière, Fernand-Widal, Paris, France; 408 Réanimation, Hospital Foch, Suresnes, France; 409 Réanimation - Grands Brulés, CHU Pointe à Pitre - Abymes, Pointe a Pitre, Guadeloupe France; 410 Réanimation polyvalente, CHU La Réunion, Saint-Denis, France; 411 Nord, Hôpital Roger Salengro, Lille, France; 412 Inserm u995 equipe 4, Université Lille 2, Lille, France; 413 Réanimation médicale, Centre Hospitalier Régional Universitaire de Lille, Lille, France; 414 Réanimation polyvalente - surveillance continue, Ctre Hospitalier Intercommunal de Villeneuve Saint Georges, Lucie et Raymond AUBRAC, Villeneuve-Saint-Georges, France; 415 Anesthésie réanimation, Hopital Louis Pradel, Bron, France; 416 Unité de soins intensifs d’hépatogastroentérologie, Pitié-Salpêtrière Hospital, Paris, France; 417 Unité de soins intensifs d’hépatogastroentérologie, Hôpital Pitié-Salpêtrière, Paris, France; 418 Unité de Soins Intensifs. Service d’Hépato-gastro-entérologie du Pr Poynard, CHU Pitié Salpétrière, Paris, France; 419 Réanimation médicale, Hôpital central, , C.H.U. de Nancy, Nancy, France; 420 Réanimation polyvalente, Hôpital de Mercy, Ars Laquenexy, France; 421 Service de réanimation polyvalente, Centre hospitalier, Saint Brieuc, France; 422 Service de Réanimation Médico-Chirurgicale, CHI de Poissy/Saint-Germain-en-Laye - Site Poissy, Poissy, France; 423 Explorations fonctionelles du système nerveux adulte, Centre Hospitalier Universitaire, Amiens, France; 424 Nord, C.H. Régional Universitaire de Lille (CHRU de Lille), Lille, France; 425 Nord, C.H. de Valenciennes, Valenciennes, France; 426 Coordination prélèvements organes, CHU Hôtel-Dieu Nantes, Nantes, France; 427 Réanimation chirurgicale, C.H.U. Hôtel Dieu, Nantes, France; 428 Néphrologie-transplantation rénale, CHU Hôtel-Dieu Nantes, Nantes, France; 429 Laboratoire de biochimie et biologie moléculaire, Centre Hospitalier Régional Universitaire de Lille, Lille, France; 430 Hospital Center De Versailles, 78150 Le Chesnay, France; 431 Anesthesia and Reanimation, Cliniques universitaires de kinshasa, Kinshasa, Democratic Republic of the Congo; 432 Anesthésie-Réanimation, Hôpital Privé d’Athis-Mons - Site Caron, Athis-Mons, France; 433 Scool of Public Health, Université de Kinshasa, Batiment administratif, Kinshasa, Democratic Republic of the Congo; 434 Burn Care Department, Trauma and Burn Center, Tunis, Tunisia; 435 Unité d’épidémiologie et recherche clinique, Hospital Center University Beaujon (AP-HP), Clichy, France; 436 Biology Department, Trauma Center, Ben Arous, Tunisia; 437 Anesthésie réanimation, Hôpital Européen Georges-Pompidou (AP-HP), Paris, France; 438 Biostatistiques, Hôpital Saint-Louis (AP-HP), Paris, France; 439 Keenan Research Center, St Michael’s Hospital, Toronto, Canada; 440 Inserm umr_s 1158 “neurophysiologie respiratoire expérimentale et clinique”, Pitié-Salpêtrière Hospital, Paris, France; 441 Département d’anesthésie-réanimation et urm_s 1158, Hôpital Universitaire Pitié-Salpêtrière, Paris, France; 442 Réanimation polyvalente, Hôpital Saint-Roch, Nice, France; 443 Centre hépato-billiaire, APHP Hôpital Paul-Brousse, Villejuif, France; 444 Réanimation, C.H.U de Caen, Caen, France; 445 Unité de biostatistiques, Hôpital Bretonneau, Tours, France; 446 Réanimation Médicale, CHU Saint-Etienne - Hôpital Nord, Saint-Étienne, France; 447 Reanimation, Hôpital Bichat-Claude Bernard (AP-HP), Paris, France; 448 Réanimation, Fondation Hopital Saint Joseph, Paris, France; 449 Réanimation polyvalente, Centre Hospitalier Général, Gonesse, France; 450 Réanimation médicale, Centre Hospitalier Sud Essonne, Dourdan, France; 451 Réanimation chirurgicale, Hôpital Antoine Béclère, Clamart, France; 452 Réanimation médicale, C.H.U. Grenoble, Grenoble, France; 453 Chirurgie ctcv transplantation thoracique, CHU de Nantes - Hôpital Nord Laennec, Saint-Herblain, France; 454 Anesthésie réanimation, Centre Hospitalier Universitaire Rouen, Rouen, France; 455 Réanimation chirurgicale digestive, Hôpital Saint-Antoine, Paris, France; 456 Réanimation urgence et médicale pr auffray, Hôpital de la Timone, Marseille, France; 457 Intensive care, CHU Sart-Tilman, Liège, Belgium; 458 Réanimation médicale, Centre Hospitalier Universitaire de Rennes, Rennes, France; 459 Biosit and inserm u917, faculte de medecine, Université rennes 1, Centre Hospitalier Universitaire de Rennes, Rennes, France; 460 Anesthésie-réanimation, Centre Hospitalier Universitaire de Rennes, Rennes, France; 461 Service de chirurgie thoracique, cardiaque et vasculaire, Centre Hospitalier Universitaire, Rennes, France; 462 Biosit and inserm u917, faculte de medecine, Universite rennes 1, Centre Hospitalier Universitaire de Rennes, Rennes, France; 463 Post-graduation Program, D’Or Institute for Research and Education, Rio de Janeiro, Brazil; 464 Department of Critical Care Medicine and Division of Pulmonary and Critical Care Medicine, Sungkyunkwan University, Seoul, Republic of Korea; 465 Department of Intensive Care, Ghent University hospital, Ghent, Belgium; 466 Division of Pulmonary, Allergy and Critical Care Medicine, Seoul St. Mary’s Hospital, Seoul, Republic of Korea; 467 Intensive care, anaesthesia, and surgery, The Royal Marsden Hospital, London, UK; 468 Nephrology and Intensive Care, St Thomas’ Hospital, London, UK; 469 Hematology, Leeds Teaching Hospital, Leeds, UK; 470 Department of Intensive Care, Ghent University Hospital, Ghent, Belgium; 471 Hematology, Catalan Institute of Oncology, Barcelona, Spain; 472 Department of critical care medicine and pulmonary medicine, Hospital de Clinicas de Porto Alegre, Porto Alegre, Brazil; 473 Département de santé publique, Institut de Cancérologie de la Loire Lucien Neuwirth, Saint-Priest-en-Jarez, France; 474 Centre d’infection et d’immunité de lille equipe 4 - basic and clinical immunity of parasitic di, Institut Pasteur de Lille, Rue du Professeur Calmette, Lille, France; 475 Centre de biologie et pathologie génétique, laboratoire de mycologie et parasitologie, Centre Hospitalier Régional Universitaire de Lille, Lille, France; 476 Service de réanimation chirurgicale, Hôpital huriez, Centre Hospitalier Régional Universitaire de Lille, Lille, France; 477 Inserm u995-2, Universite Lille - Droit et Santé, Lille, France; 478 Inserm u1019 e13, Institute Pasteur De Lille, Lille, France; 479 LUNGINNOV, 59000 Lille, France; 480 Physiotherapy, Hôpital Forcilles - Fondation Cognacq-Jay, Férolles-Attilly, France; 481 Department of Healthy Profession, Intensive Care Unit, Division of Cardiology, Careggi University Hospital, Firenze, Italy; 482 Division of Geriatric Cardiology and Medicine, Research Unit of Medicine of Ageing, Careggi University Hospital, Firenze, Italy; 483 Intensive Care Unit, Cliniques universitaires Saint-Luc, Université catholique de Louvain, Brusseles, Belgium; 484 Institute of Neuroscience, Université catholique de Louvain, Louvain-la-Neuve, Belgium; 485 Neuromuscular reference centrer, Cliniques universitaires Saint-Luc, Université catholique de Louvain, Brussels, Belgium; 486 Department of Physical Medicine and Rehabilitation, Cliniques universitaires Saint-Luc, Université catholique de Louvain, Brussels, Belgium; 487 Sevice des soins intensifs, Cliniques Universitaires Saint-Luc, Bruxelles, Belgium; 488 Icu, C.H. Epicura, Hornu, Belgium; 489 Physiotherapy Department, Surgical Center Marie Lannelongue, Le Plessis-Robinson, France; 490 Réanimation adulte, Surgical Center Marie Lannelongue, Le Plessis-Robinson, France; 491 Médecine nucléaire, Cliniques Universitaires Saint-Luc, Brussels, Belgium; 492 Respiratory, Haute École de Santé Vaud, Lausanne, Switzerland; 493 Service de médecine nucléaire, Cliniques Universitaires Saint-Luc, Woluwe-Saint-Lambert, Belgium

## .

### .

#### P144 Post traumatic cerebral thrombophlebitis: prospective study about 15 cases

##### Chtara Kamilia^1^, Kais Regaieg^2^, Olfa Turki^1^, Najeh Baccouch^2^, Hedi Chelly^1^, Mabrouk Bahloul^1^, Mounir Bouaziz^1^

###### ^1^Réanimation polyvalente, Faculté de médecine de Sfax, Sfax, Tunisia; ^2^Réanimation polyvalente, CHU Habib Bourguiba, Sfax, Tunisia

####### **Correspondence:** Chtara Kamilia - kamilia.chtaraelaoud@gmail.com

*Annals of Intensive Care* 2017, **7**(**Suppl 1**):P144

**Introduction** Head injury is a rare but possible etiology of cerebral thrombophlebitis. The diagnosis should be considered especially in front of open head injuries extended to venous sinuses. The MR angiography is the gold standard for early diagnosis.

**Patients and methods** This is a descriptive prospective study of all trauma patients hospitalized in the intensive care unit of the University Hospital Habib Bourguiba Sfax over a period of 6 years between January 2010 and June 2016 and in whom the diagnosis of cerebral venous thrombophlebitis has been confirmed by angiography CT or MR angiography.

**Results** During the period study, 15 patients were included. The median age of patients was 29 [17–49] years. All patients were male, victims of poly trauma following an accident of traffic. In admission, SAPSII was 31 [24–52] and SOFA was 4 [2–8]. We have noted the presence of a serious head injury in 15 patients, extended open skull fractures of the venous sinus in 9 patients. A related chest trauma was present in 12 patients and abdominal trauma in 4 patients, trauma of the pelvis and/or members were present in 7 patients. All patients underwent mechanical ventilation. The diagnosis of cerebral venous thrombosis was confirmed by cerebral angiography CT in 9 patients and cerebral MR angiography in 6 patients. 7 patients have presented secondary pulmonary embolism. All patients did not show a contraindication against anticoagulation at diagnosis of thrombophlebitis. The thrombophilia (antithrombin III, protein C and S, homocysteine, and antiphospholipid, gene mutation factors II and V) as well as for anti-neutrophil cytoplasmic antibodies were negative in all patients. The outcome was favorable in 13 patients. Two patients were died due to a state of refractory septic shock.

**Discussion** Post traumatic cerebral thrombophlebitis is a rare thrombotic vascular disease. It must be mentioned especially with presence of extensive skull fractures in open sinuses. Venous MR angiography is the gold standard. The treatment is based on anticoagulation curative dose. Its prescription can be complicated in these cases associated with traumatic intracranial hemorrhage.

**Conclusion** Head injury is a rare but possible etiology of cerebral thrombophlebitis. Other prospective studies are needed to better understand the path physiology and the prognosis of these thromboses.

**Competing interests** None.

#### P145 Pain measurement in mechanically ventilated patients with traumatic brain injury: behavioral pain tools versus analgesia/nociception index—preliminary results

##### Ali Jendoubi^1^, Ahmed Abbes,^1^, Houda Belhaouane,^1^, Oussama Nasri,^1^, Layla Jenzri,^1^, Salma Ghedira^2^, Mohamed Houissa^2^

###### ^1^Anesthesia and Intensive Care, Charles Nicolle Teaching Hospital, Tunis, Tunisia; ^2^ Intensive care, Charles Nicolle Hospital, Tunis, Tunisia

####### **Correspondence:** Ali Jendoubi - jendoubi_ali@yahoo.fr

*Annals of Intensive Care* 2017, **7**(**Suppl 1**):P145

**Introduction** Pain is highly prevalent in critically ill trauma patients especially those with a traumatic brain injury (TBI). Behavioral pain tools such as the Behavioral pain scale (BPS), and critical care pain observation tool (CPOT) are recommended for sedated non-communicative patients. The analgesia nociception index (ANI) assesses the relative parasympathetic tone as a surrogate for antinociception/nociception balance in sedated patients. The primary aim is to evaluate the effectiveness of ANI in detecting pain in TBI patients. The secondary aim was to evaluate the impact of Norepinephrine use on ANI effectiveness, and to determine the correlation between ANI and BPS.

**Patients and methods** We performed a prospective observational study in 21 deeply sedated TBI patients. Exclusion criteria were non-sinus cardiac rhythm; presence of pacemaker; atropine or isoprenaline treatment; neuromuscular blocking agents and major cognitive impairment. HR, blood pressure and ANI were continuously recorded using the Physiodoloris^®^ device at rest (T1), during (T2) and after the end (T3) of the painful stimulus (tracheal suctioning).

**Fig. 1:**
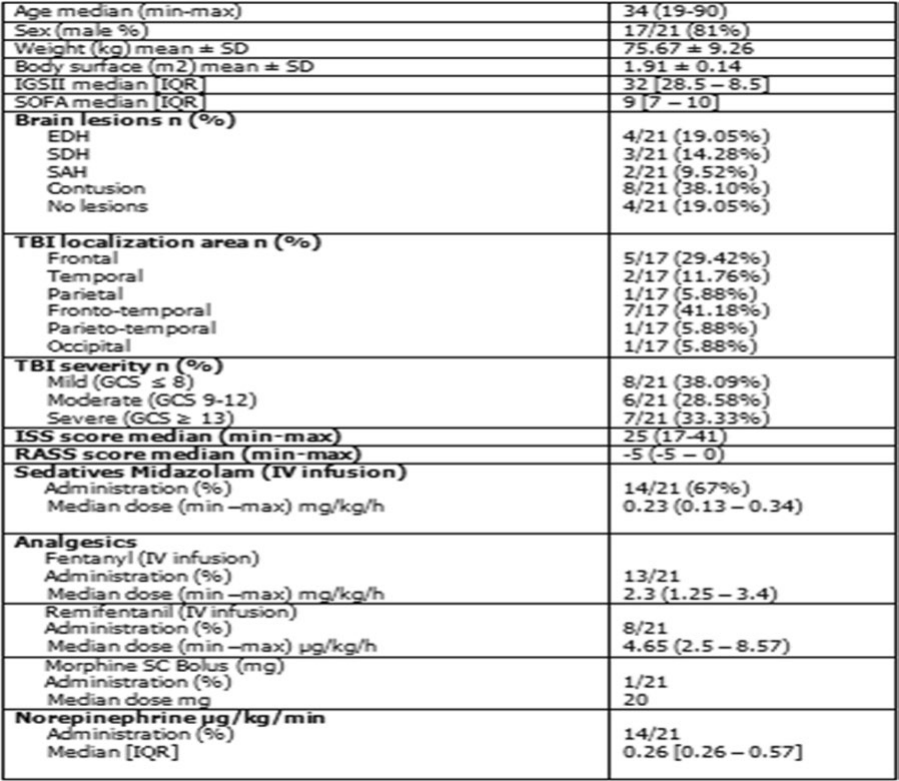
Baseline demographic and clinical characteristics. Values are expressed as mean ± standard deviation (SD); n (%) or median [interquartile range]. *EDH* extradural haemorrhage *SDH* subdural haemorrhage, *SAH* subarachnoid hemorrhage

**Results** In total, 100 observations were scored. Patients’ *characteristics* were *resumed* in Fig. 1. ANI was significantly lower at T2 (Med (min–max) 54.5 (22–100)) compared with T1 (90.5 (50–100), p < 0.0001) and T3 (82 (36–100), p < 0.0001). Similar results were found in the subgroups of patients with (65 measurements) or without (35) Norepinephrine. During procedure, A negative linear relationship was observed between ANI and BPS (r^2^ = −0.469, p < 0.001). At the threshold of 50, the sensitivity and specificity of ANI to detect patients with BPS ≥ 5 were 73 and 62%, respectively with a negative predictive value of 86%.

**Discussion**

**Conclusion** ANI is effective in detecting pain in deeply sedated critically ill TBI patients, including those patients treated with Norepinephrine.

**Competing interests** None.

#### P146 The prognosis of cervical spine trauma in elderly subjects in surgical intensive care

##### Kamal Belkadi^1^, Ma Bouhouri^2^, Youness Harti^3^, Afak Nsiri^2^, Khalid Khaleq^4^, Driss Hamoudi^2^, Rachid Harrar^2^

###### ^1^Anesthesie reanimation, chu ibn rochd, Casablanca, Morocco; ^2^Reanimation des urgences chirurgicale, chu ibn rochd, Casablanca, Morocco; ^3^Anesthésie réanimation, CHU Ibn Rochd Casa, Casablanca, Morocco; ^4^Service d’accueil des urgences, Chu Ibn Rochd, Casablanca, Morocco

####### **Correspondence:** Kamal Belkadi - kamal.belkadi@gmail.com

*Annals of Intensive Care* 2017, **7**(**Suppl 1**):P146

**Introduction** The aim of our study was to assess the prognostic factors of cervical spine trauma in elderly subjects admitted in the surgical intensive care unit.

**Patients and methods** We conducted a retrospective, and single-center study over 16 years (January 2000–January 2016) in Ibn Rochd hospital, we included all patients aged over 65 years with isolated cervical spine trauma, operated and non-operated, admitted in surgical intensive care, the death risk factors were searched by uni and multivariate analysis.

**Results** 198 patients were collected, the average age was 69.4 ± 3.9 years, with a male predominance 70.2%, the main causes were road accidents (50.5%) and fall (34.3%), 68.2% had a complete form (Frankel A), 75.7% were operated. The death rate in our study was 55%. The prognostic factors in univariate analysis were: hypertension, heart disease, fall injury, surgical delay >10 h; independent factors of death in multivariate analysis: heart disease and fall injury.

**Conclusion** The cervical spine trauma in elderly patients hospitalized in intensive care unit is poor prognosis.

**Competing interests** None.

#### P147 Interest of the urine antigen testing for *Legionella pneumophila* in the management of severe acute pneumonia: practice survey and analysis of performance in intensive care unit

##### Camille Thieffry^1^, Frédéric Wallet^2^, Erika Parmentier-Decrucq^1^, Raphaël Favory^1^, Daniel Mathieu^1^, Julien Poissy^1^

###### ^1^Pôle de réanimation, hôpital salengro, C.H.R.U. - Lille, Avenue Oscar Lambret, Lille, France, Lille, France; ^2^ Centre de biologie pathologie génétique, Centre Hospitalier Régional Universitaire de Lille, Lille, France

####### **Correspondence:** Julien Poissy - julien_poissy@hotmail.fr

*Annals of Intensive Care* 2017, **7**(**Suppl 1**):P147

**Introduction** Legionnaire’s disease is a rare but severe acute pneumonia with a difficult definitive diagnosis. Legionella’s urine antigen testing is a quick, sensitive and specific test, widely prescribed in the case of an acute and severe community-acquired pneumonia. However, its overall contribution to the diagnosis of atypical pneumonia remains unknown in daily practice. The aims of this study were to evaluate the usefulness of this test in a “real life” utilization and to identify potential clinical markers of legionnaire’s disease in order to optimize its prescription. Legionnaire’s disease is a rare but severe acute pneumonia with a difficult definitive diagnosis. Legionella’s urine antigen testing is a quick, sensitive and specific test, widely prescribed in the case of an acute and severe community-acquired pneumonia. However, its overall contribution to the diagnosis of atypical pneumonia remains unknown in daily practice. The aims of this study were to evaluate the usefulness of this test in a “real life” utilization and to identify potential clinical markers of legionnaire’s disease in order to optimize its prescription.

**Patients and methods** We conducted a retrospective, monocentric and observational study. All the prescriptions of the urine antigen testing where monitored in the intensive care department and the emergency room admitting severe patients, in our University Teaching Hospital, from January 1st 2013 to December 31st 2015. Qualitative variables were compared by a Fischer’s exact test, and quantitative variables by a Mann–Whitney test. All tests were bilateral, and p ≤ 0.05 was considered as significant. ROC curves were determined for the variables of interest.

**Results** During the period of the survey, 1142 urinary tests were performed in 1002 patients. Three tests were positive for 0.26% of patients. Only 569 patients suffered from an actual pneumonia. Other patients suffered mostly from acute bronchitis (137 cases), and exacerbation of chronic obstructive pulmonary disease (123 cases). The characteristics of the 3 patients suffering from legionnaire’s disease differed significantly compared to the other kinds of acute pneumonia for: the need for invasive mechanical ventilation (100 vs 34.96% of patients; p = 0.043), PaO_2_/FiO_2_ ratio (107.5 vs 274.5; p = 0.0107), duration of mechanical ventilation (27.5 vs 2.5 days; p = 0.0062), natremia (129.5 vs 138 mmol/l; p = 0.0125), Creatine Phospho-Kinase level (16,125 vs 106UI; p = 0.0225) and Serum Glutamat Oxalacetat Transaminase (251 vs 35UI; p = 0.0157). We determined ROC curves for these last biological variables. Natremia: better threshold = 131, Se/Sp = 100/84.2%, AUC = 0.92. SGOT: better threshold = 191, Se/Sp = 100/89.6%, AUC = 0.9. CPK: better threshold 195, Se/Sp = 100/66.5, AUC = 0.88.

**Discussion** Urine antigen testing for Legionnaire’s disease appears to be over-prescribed in many cases with a very poor level of positivity in our cohort. However, it could be limited to patients exhibiting a pneumonia, and among them to patients with the classical biological perturbations described in this disease, which have excellent diagnostic performance. This targeted strategy would present an important benefit in term of costs.

**Conclusion** Our results show that a better selection in the patients who could benefit of this test is mandatory. Simples markers in routine testings could help the clinician to adapt his prescription, optimizing this test’s efficiency.

**Competing interests** None.

#### P148 Staphylococcal community-acquired urinary tract infection in the emergency department: a sign for acute infective endocarditis?

##### Thomas Lafon^1^, Philippe Vignon^2^, Emmanuelle Begot^2^, Alexandra Appert^3^, Mathilde Hadj^3^, Paul Claverie^3^, Morgan Matt^4^, Olivier Barraud^5^, Bruno François^6^

###### ^1^Inserm cic 1435/urgences/samu, Centre Hospitalier Universitaire de Limoges, Limoges, France; ^2^Service de réanimation polyvalente, Centre Hospitalier Universitaire de Limoges, Limoges, France; ^3^Urgences/samu, Centre Hospitalier Universitaire de Limoges, Limoges, France; ^4^Service de maladies infectieuses, Centre Hospitalier Universitaire de Limoges, Limoges, France; ^5^Bactériologie-virologie-hygiène/umr-s 1092, Centre Hospitalier Universitaire de Limoges, Limoges, France; ^6^Inserm cic1435/service de réanimation polyvalente, Centre Hospitalier Universitaire de Limoges, Limoges, France

####### **Correspondence:** Thomas Lafon - thomas.lafon@chu-limoges.fr

*Annals of Intensive Care* 2017, **7**(**Suppl 1**):P148

**Introduction** Urinary tract infection is a frequent cause of admission at the Emergency Department (ED). Most prevalent bacteria are usually gram-negative bacilli and *Staphylococcus aureus* (*Sa*) is rarely evidenced (2.5%) except in hospital-acquired infections due to urinary catheter [1]. Bacteriuria can be observed in *Sa* infective endocarditis (IE) because of the metastatic properties of *Sa*. We hypothesized that presence of *Sa* in the urine could be related to Staphylococcal bacteremia associated with unsuspected IE and not only the expression of a “usual” urinary tract infection.

**Patients and methods** This is a descriptive single-center study conducted over a 10 year-period in the Teaching Hospital of Limoges. All patients admitted to the ED with *Sa* (both MSSA and MRSA) isolated from their urine cultures were retrospectively analyzed. Data were collected from the database of the microbiology department and the patient medical charts. We secondarily searched if a *Sa* IE had been documented in patients with *Sa* isolated from their blood cultures in order to establish a link between IE and presence of *Sa* in the urine. We used modified Dukes criteria as diagnostic criteria of IE [2].

**Results** Between 2005 and 2015, 420,000 patients were admitted in the ED. Out of the 204 records analyzed, 174 patients whose urine culture grew *Sa* were excluded because they had a urinary catheter (n = 75) or sterile blood cultures (n = 99). Finally, 30 patients were studied (17 men; median age: 73 years; diabetes: n = 7; mitral valvular disease: n = 2, aortic valvular disease: n = 2) (Table 1). Reasons for admission were markedly heterogeneous and fever accounted for 14 cases. Echocardiography was performed in 25 patients with a median delay of 6.5 days (range: 0–23 days) and IE was confirmed in 21 of them. Only three cases of IE have been diagnosed in the ED because of a fever and valvular murmur (n = 2) but no patient was admitted to the ED for IE suspicion. Other initially suspected diagnoses were prostatitis, pneumonia or appendicitis and not related to any type of infection in 26% of the cases. The origin of *Sa* bacteremia was cutaneous in 67% of the cases. During the hospital stay, 70% of the patients presented secondary sites of *Sa* infection in addition to the urinary tract (arthritis, splenic abcess, cerebral hematoma). Among 21 patients presenting with an IE, 8 died within 7 days, and total hospital mortality reached 50%.

**Table 1:** Patients characteristics

Characteristics	n (%)
Median age (year)	63
Heart murmur	5 (24)
Congenital heart valve defect	19 (90)
Mitral localization	19 (90)
Abuse (drug, alcohol…)	4 (19)
Microbiology	
MSSA	20 (95)
MRSA	1 (5)
Origin	
Skin	14 (67)
Unknown	7 (33)
Complications	
Central nervous system	9 (43)
Visceral abscesses	2 (9.5)
Septic arthritis	9 (43)
Skin and eye petechiae	9 (43)
Death	8 (38)

**Conclusion** This case series suggests that IE should be ruled out when *Sa* bacteriuria is evidenced, irrespective of the clinical presentation. This could question the reality of isolated community-acquired urinary tract infections due to *Sa*.

**Competing interests** None.

**References**

1. Li JS, Sexton DJ, Mick N, Nettles R, Fowler VG, Ryan T, et al. Proposed modifications to the Duke criteria for the diagnosis of infective endocarditis. Clin Infect Dis Off Publ Infect Dis Soc Am. 2000;30(4):633–8.
2. Ekkelenkamp MB, Verhoef J, Bonten MJ. Quantifying the relationship between *Staphylococcus aureus* bacteremia and *S. aureus* bacteriuria: a retrospective analysis in a tertiary care hospital. Clin Infect Dis Off Publ Infect Dis Soc Am. 2007;44(11):1457–9.

#### P149 The infectious tricuspid endocarditis in ICU: clinical features, management and outcome

##### Amira Jamoussi^1^, Amira Ben Jazia^1^, Takoua Marhbène^1^, Dhouha Lakhdhar^1^, Jalila Ben Khelil^1^, Mohamed Besbes^1^

###### ^1^Medical icu, Hospital Abderrahmen Mami De Pneumo-Phtisiologie, Ariana, Tunisia

####### **Correspondence:** Amira Jamoussi - dr.amira.jamoussi@gmail.com

*Annals of Intensive Care* 2017, **7**(**Suppl 1**):P149

**Introduction** Since the outbreak scourge of intravenous drug addiction in Tunisia, we are witnessing the emergence of cases of infectious tricuspid endocarditis (ITE). This pathology should be studied because it requires specific medical and surgical management. The aim of the study was to describe the clinical features, management and outcome of ITE in intensive care unit.

**Patients and methods** This was a retrospective study from January 2009 to December 2014. We enrolled patients who were hospitalized in intensive care unit and had ITE. We recorded baseline characteristics, management and outcome.

**Results** During the study period, we collected 10 cases of ITE making an incidence rate of 3 cases for 1000 patient admissions. They were divided into 8 men and 2 women. The median age was of 37.5 years. The main reasons of ICU admission was acute respiratory failure (80%), among them 5 required mechanical ventilation. Hemodynamic failure was present in 3 cases.

The median SAPS II was of 33 [19–90]. The median APACHE II was of 17 [7–53]. Different contributing factors were identified: intravenous drug abuse (6 patients), a central venous catheter (1 patient) and a pacemaker (1 patient).

All patients underwent transesophageal echocardiography showing one or several vegetations on native tricuspid valve. No associated left endocarditis was found.

Blood cultures were positive in 8 cases of which 5 contained 2 different micro-organisms. The identified micro-organisms were: *Meticillin Resistant Staphylococcus aureus* (n = 6), *Meticillin Sensitive Staphylococcus aureus* (n = 3), *coagulase*-*negative staphylococcus* (n = 2), *Enterobacter cloacae* (n = 1), and *candida famata* (n = 1). Occurring complications were hospital-acquired infections (n = 5), septic pulmonary embolism (n = 4), withdrawal syndrome (5 cases), acute renal failure (n = 2) and atrioventricular block (n = 2).

Medical treatment consisted of a double antibiotic treatment. Surgical treatment was required in 7 patients: tricuspid valve replacement by bioprosthesis (6 cases) and valvuloplasty (1 case).

The average length of stay was of 31.3 days [2–56]. ITE had recurred on bioprotheses in two patients after intravenous drug resumption; they underwent surgery again and one of them died.

In hospital mortality was of 30%. The outcome was favorable in 7 patients.

**Conclusion** The ITE in ICU is a severe disease with frequent complications and in hospital mortality reaches 30%. The most frequent incriminated micro-organism is *Meticillin Resistant Staphylococcus aureus.* It often requires medical and surgical treatment. Intravenous drug addiction remains the most common cause and worsens the prognosis by the risk of recurrence.

**Competing interests** None.

#### P150 Microbiological mapping of community-acquired intra-abdominal infections (IAI) and indicator of local antibiotherapy appropriateness with French national guidelines

##### Julien Goutay^1^, Caroline Blazejewski^2^, Isabelle Joly-Durand^3^, Isabelle Pirlet^4^, Marie Pierre Weillaert^5^, Sebastien Beague^2^

###### ^1^Interne en anesthésie réanimation, C.H. Régional Universitaire de Lille (CHRU de Lille), Lille, France; ^2^Réanimation polyvalente, Hospital Center De Dunkerque, Dunkerque, France; ^3^Equipe opérationnelle d’hygiene, Hospital Center De Dunkerque, Dunkerque, France; ^4^Service de chirurgie digestive, Hospital Center De Dunkerque, Dunkerque, France; ^5^Laboratoire, Hospital Center De Dunkerque, Dunkerque, France

####### **Correspondence:** Julien Goutay - julien.goutay@gmail.com

*Annals of Intensive Care* 2017, **7**(**Suppl 1**):P150

**Introduction** French guidelines for community-acquired IAI underline the importance of establishing antibiotherapy protocols based on regular analysis of microbiological data; and of systematic site infection cultures to determine microbial sensitivity to antibiotics. Our study describes microbial population involved in our community-acquired IAI and defines an annual follow-up indicator of probabilistic antibiotherapy inadequacy to microbial sensitivity.

**Materials and methods** We conducted a retrospective, monocentric, observational study from January the 1st 2014 to December the 31st 2015. All community-acquired IAI in adults were included. Exclusion criteria were: cirrhosis and peritoneal dialysis. Initial probabilistic antibiotherapy and total antibiotherapy duration were left to the discretion of the physician in charge. Results of intra-abdominal cultures (IAC) were analyzed. Three microbial groups were defined: (A) A-group: wild-type bacteria strains treated with adequat antibiotherapy; (B) B-group: antibiotic resistant bacteria treated with adequat antibiotherapy; (C) C-group: multi-drug resistant bacteria treated with inadequat antibiotherapy. A Chi square analysis was performed on SPSS software (IBM).

**Results** 98 community-acquired IAI were included: 54 (55%) had positive IAC with 133 bacteria; 34 (35%) didn’t have intra-abdominal swab; 10 (10%) had sterile cultures. Predominant strains were Gram-negative bacteria (76/133 (57%)). More represented bacteria were *Escherichia Coli* (50/133 (38%)) and *Bacteroïdes fragilis* (16/133 (12%)). 127/133 (95%) bacteria belong to A- and B-groups. B-group importance increased significantly between 2015 and 2016. C-group characteristics were comparable over the 2 years. Initial antibiotherapy was inadequate with French guidelines in 38/98 (39%) cases and with microbial antibiotic susceptibility in 6/133 (4.5%) cases (C-group). Average antibiotherapy duration was 11.3 days. Antibiotic treatment duration was too long according to French guidelines for 65/98 (67%) patients.

**Discussion** Our microbial population in community-acquired IAI is similar to national studies with a lowest resistance rate (C-group under 10%). Probabilistic antibiotherapy proposed by French guidelines is appropriated to our microbial ecology. Antibiotherapy duration is unconformed with guidelines in 67% cases. Peroperative swabs are frequently missing (35%). Non-compliance with French guidelines highlights the importance to formalize our local procedure. This formalization at any stage (surgical, medical and biological cares) seems essential to improve our standard of care. C-group rate could be used as a real-time feedback to adapt our protocol continuously.

**Conclusion** The goal of our study is to improve local standard of care by offering a formalization of community-acquired IAI management procedure. C-group rate seems to be a good follow-up indicator of probabilistic antibiotherapy inadequacy to microbial sensitivity, allowing an optimization of our protocol in real-time.

**Competing interests** None.

**Reference**

1. Montravers et al. Recommandations Formalisées d’Experts «Prise en charge des infections intra-abdominales», Octobre 2015.

#### P151 The resumption of peritonitis in surgical intensive care unit

##### Ma Bouhouri^1^, Kamal Belkadi^2^, Soufi Aziz^1^, Khalid Khaleq^3^, Afak Nsiri^1^, Driss Hamoudi^1^, Rachid Harrar^1^

###### ^1^Reanimation des urgences chirurgicale, chu ibn rochd, Casablanca, Morocco; ^2^Anesthesie reanimation, chu ibn rochd, Casablanca, Morocco; ^3^Service d’accueil des urgences, Chu Ibn Rochd, Casablanca, Morocco

####### **Correspondence:** Kamal Belkadi - kamal.belkadi@gmail.com

*Annals of Intensive Care* 2017, **7**(**Suppl 1**):P151

**Introduction** The resumption of peritonitis is a serious complication of abdominal and pelvic surgery. It’s a medical and surgical emergency, the prognosis depends on the speed, the quality of the care, and the underlying terrain and etiology.

**Patients and methods** We conducted a descriptive analytic retrospective study over a period of 5 years (January 2011–June 2016) 60 cases of peritonitis hospitalized in surgical intensive care unit.

**Results** The average age of our patients was 44.36 years with a sex ratio of 1.5 (36H/24F). The most frequent risk factors were: factors relating to the ground, and factors related to the initial peritonitis.

Clinical signs were dominated by fever (75%), abdominal pain (52%). The period of the average recovery was 8.2 days. The decision of the surgical revision was based on a clinical, biological and radiological criteria.

40 patients in our series, 67% of cases were taken on clinical and biological criteria while 15 patients 25% were taken on radiological criteria. In 8% of the remaining cases, the potential severity of the clinical and biological state in association with an inconclusive ultrasound, led to reoperation.

The therapeutic treatment was based on a perioperative resuscitation, treatment of organ failure, empirical antibiotic therapy and by midline laparotomy surgery. Bacteriological samples performed intraoperatively allowed to have the following bacteriological profile: predominance of BGN (79%) dominated by *E. coli* (28%) followed by *Klebsiella pneumoniae* (21%), Acinetobacter and *Enterococcus baumanii* (12%). The multimicrobien character was found in 55%. The *E. coli*–*Klebsiella pneumoniae* association was the most frequent (37%).

The anastomotic dehiscence was the direct cause of the most common surgical revision found intraoperative (62%). The average hospital stay was 8 days. The mortality rate was 61%. The main prognostic factors in our study emerged in the univariate analysis were: kidney failure, the number of organ failure, a TP <50% the needs of ventilation and the use of catecholamines.

**Discussion** Mortality is variable depending on the studies, between 25 and 60%.

**Conclusion** The diagnosis often difficult. Only effective and early therapeutic management reduces mortality remains high in recent years despite the various advances in the field of surgery and reanimation.

**Competing interests** None.

#### P152 Prognostic factors in intra abdominal sepsis: a prospective study

##### Reda Hafiane^1^, Khalid Khaleq^1^, Khalid Hattabi^2^, Mohamed Aziz Bouhouri^1^, Afak Nsiri^1^, Driss Hammoudi^1^, Abdelaziz Fadil^2^, Rachid Al Harrar^1^

###### ^1^Service de réanimation des urgences chirurgicales, CHU IBN ROCHD de Casablanca, casablanca, Morocco; ^2^Service des urgences viscérales, CHU Ibn Rochd de casablanca, Casablanca, Morocco

####### **Correspondence:** Reda Hafiane - hafiane.reda89@gmail.com

*Annals of Intensive Care* 2017, **7**(**Suppl 1**):P152

**Introduction** Intra- abdominal sepsis represents a life threatening condition. Its manifestations are non specific and can quickly lead to multi organ failure if not treated correctly. Patient’s assessment is essential in order to adjust the therapy. The aim of our study is to highlight the prognostic factors in this situation.

**Patients and methods** It’s a prospective observational study performed during 6 months (02/2016–08/2016) in visceral emergency operating rooms.

Inclusion criteria: adults admitted with intra-abdominal infectious disease diagnosed with biological and radiological means.

Studied parameters: demographic data, co morbidities, number of organ failure, type of anesthesia, intraoperative incidents and evolution. Results were analyzed using SPSS software, prognostic factors were extracted with univariate then multivariate analysis. Significant results were noted.

**Results** During this period, we admitted 302 patients, the mean age: 41.14 ± 17 years. Male predominance was noticed in our population: 69.9%. The mortality rate was: 13.2%.

The main prognostic factors were reported in the attached Table 2.

**Table 2:** Main prognostic factors in intra abdominal sepsis

Variable	Hazard ratio unadjusted	p	Hazard ratio adjusted	p
Age	1.047	0.001	1.032	0.009
Sex	3.25	0.001	0.068	0.47
High hemoglobin	0.68	0.01	0.33	0.52
creatinine	1.11	0.001	1.16	0.001
Pesence of clammy skin	14.74	0.001	77	0.05
Urea	37.34	0.01	37	0.25
Hemodynamic instability	26	0.001	1.27	0.834
Use of vasoactive drugs	29	0.001	34	0.001
Operating time	1.018	0.0001	1.014	0.001

**Discussion** Many significant prognostic factors were identified: Age, existence of hemodynamic failure with renal involvement, long operative time and the use of vasoactive drugs.

A high hemoglobin level at the admission was a protective factor.

The presence of respiratory distress, the sex and the presence of yeasts were not significant factors in our study.

**Conclusion** Intra abdominal sepsis is causing quickly a multi organ dysfunction syndrome leading to death. Therefore, our priority is to stop this sepsis with the help of the surgeon and the efficient use of antibiotics.

**Competing interests** None.

#### P153 Community acquired intra abdominal sepsis: concerning 302 cases

##### Reda Hafiane^1^, Khalid Khaleq^1^, Khalid Hattabi^2^, Mohamed Aziz Bouhouri^1^, Afak Nsiri^1^, Driss Hammoudi^1^, Khalid Zerouali^3^, Abdelaziz Fadil^2^, Rachid Al Harrar^1^

###### ^1^Service de réanimation des urgences chirurgicales, CHU IBN ROCHD de Casablanca, casablanca, Morocco; ^2^ Service des urgences viscérales, CHU Ibn Rochd de casablanca, Casablanca, Morocco; ^3^Service de microbiologie, CHU Ibn Rochd de casablanca, Casablanca, Morocco

####### **Correspondence:** Reda Hafiane - hafiane.reda89@gmail.com

*Annals of Intensive Care* 2017, **7**(**Suppl 1**):P153

**Introduction** Intra abdominal sepsis is a dangerous condition causing a high mortality rate even with surgery and post operative care improvement.

The aim of the study is to assess the bacteriological and epidemiological profile of this population.

**Patients and methods** It’s a prospective observational study performed during 6 months (02/2016–08/2016) in visceral emergency operating rooms.

Inclusion criteria: adults admitted with intra-abdominal infectious disease diagnosed with biological and radiological means.

Exclusion criteria: post operative peritonitis and deceased patients before their admittance.

Studied parameters: demographic data (gender, age…), the time management, co morbidities, number of organ failure, intraoperative incidents and postoperative evolution.

**Results** During this period, we admitted 302 patients, the mean age: 41.14 ± 17 years. Male predominance was noticed in our population: 69.9%.

Main emergencies are reported in the attached Table 3. Concerning the bacteriological profile: we had 121 positive samplings. Enterobacterias were the most frequent strain. *E coli* was predominant: 43%, *Enterococcus faecalis*: 25%, *Streptococcus (viridians* and *acidominimus):* 14%, we had 1 case of *Acinetobacter baumanii* resistant to imipenem. 6 cases of yeasts were found (C*andida albicans*).

**Table 3:** Main emergencies with epidemiological profile

Disease	Number (n)	Sex ratio (M/F)	Age (years)	Time management (days)	Number of organ failure (n)	APACHE II score	Number of deaths (n)
Peptic perforation peritonitis	48	47	42.5	1.54	1	10	1
Appendicitis	100	1.43	31.7	2.95	0	7	0
intestinal perforation peritonitis	18	0.63	36	5.6	3	19	6
Biliary peritonitis	10	0.43	62.2	7.2	2	16	4
Post traumatic peritonitis	6	6	25	3.1	3	15	2
Necrotizing fasciitis	28	13	55.9	20	0	12	0

Antibiotic use was: Ampicilline: 44%, Ceftriaxone 41%, metronidazole: 76% and tazocilline: 4%.

**Discussion** In our context, community acquired intra abdominal sepsis leads to a high death rate. We noticed relevant parameters: a late time management, high gravity scores, some surgical procedures not directed by supervisors. Therefore, we have to establish therapeutic protocols tailored to each disease in order to improve patients’ management and help to reduce the mortality rate.

**Conclusion** Early diagnosis and care for intra abdominal sepsis represent a major way to prevent complications. Bacteriological proof is necessary to adjust post operative antibiotherapy.

**Competing interests** None.

#### P154 Conformity of antibiotic prescribing in emergency room

##### Fatma Kaaniche Medhioub^1^, Rania Allela^2^, Najla Ben Algia^3^, Samar Cherif^4^

###### ^1^Faculté de médecine de Sfax, Sfax, Tunisia; ^2^Hopital régional mahres, Faculté de médecine de Sfax, Sfax, Tunisia; ^3^Intensive care, hopital régional Gafsa, Sfax, Tunisia; ^4^Intensive care, hopital régional mahres, Sfax, Tunisia

####### **Correspondence:** Fatma Kaaniche Medhioub - fatma_kaaniche@yahoo.fr

*Annals of Intensive Care* 2017, **7**(**Suppl 1**):P154

**Introduction** The development of bacterial resistance is a major public health problem due to unreasonable use of antibiotics. The introduction of appropriate antibiotic therapy has a positive impact on patient survival and a significant economic impact. The objective of this study is to evaluate the compliance of antibiotics prescribed in emergencies.

**Patients and methods** Prospective study conducted on 1 year (01/01/2015–31/12/2015). We have included patients admitted to the emergency with hyperthermia (>38°), hypothermia (<36°) or two criteria of systemic inflammatory response syndrome. Were collected in the emergency department: history, presence of prior antibiotic therapy, demographic and clinical characteristics at admission, prescription of antibiotics in emergencies and its modalities and the discharge diagnosis. During hospitalization were collected: the introduction, modification or discontinuation of the antibiotic, the reasons for this change and certainty diagnosis. The compliance analysis of antibiotic therapy was performed by an expert group (two infectiologists, a bacteriologist and an emergency doctor) with regard to the current recommendations. Two groups were defined and compared: group of patients receiving complies antibiotic therapy and group with antibiotics considered improper. The criteria associated with non-compliance were sought.

**Results** Four hundred and twenty-two patients were enrolled. The final diagnosis retained an infectious etiology in 356 patients (84%). The mean age was 62.4 ± 18 years. Blood cultures were taken in 370 cases (87.7%). Infectious sites were most often lung (52%) and urine (32%). Severe sepsis was diagnosed in 14 patients (3.3%). A complies prescription was found in 335 patients (79.4%). Antibiotic therapy was started in 302 patients (71.5%) at the emergency and classified complies with 234 (55.4%). Among the 68 patients (16.1%) with an illegal antibiotic, it was continued in 24 (35.3%) during hospitalization. Among 120 patients (28.4%) did not receive antibiotics, this attitude was classified complies in 115 patients (95.8%). Non-compliance was related to the presence of antibiotics in the last 3 months and the presence of renal failure.

**Conclusion** Particular attention should be paid to the antibiotic prescription in patients subject to prior exposure to these. Dose adjustments should be respected in cases of renal failure. Regular evaluation of the antibiotic prescription in the emergency is necessary.

**Competing interests** None.

#### P155 Pulmonary resections’ bacterial cartography

##### Mohamed Taoufik Slaoui^1^, Souhail Boubia^2^, Y. Hafiani^1^, A. Khaoudi^1^, R. Cherkab^1^, W. Elallam^1^, C. Elkettani^1^, L. Barrou.^1^, M. Ridaii^2^

###### ^1^Anesthesia service surgical resuscitation, chu ibn rochd, Casablanca, Morocco; ^2^ Thoracic surgery, chu ibn rochd, Casablanca, Morocco

####### **Correspondence:** Mohamed Taoufik Slaoui - dr.t.slaoui@gmail.com

*Annals of Intensive Care* 2017, **7**(**Suppl 1**):P155

**Introduction** The study of the bacterial cartography in thoracic surgery is extremely important for the treatment of post-operative infections due to the severity of the underlying pathology, the fragility of patients after surgery in addition to the choice of the empiric antibiotic therapy.

**Materials and methods** We led a prospective study following all the patients who underwent a pulmonary resection surgery for a period of 7 months from January to July 2016, jointly with the microbiology department, CHU Ibn Rochd, Casablanca. The bronchial secretions were collected by a protected distal bronchial sample using a (Combicath) after the intubation.

**Results** During the period of the study, 92 patients underwent a pulmonary resection, 65% for a neoplastic pathology.

The medium age was 43 years ±8 and 58% of our sample were male. 48% of our patients had smoking habits and 16 of them had pulmonary tuberculosis, 12 had repeated respiratory infections. The antibiotics used in pre-operative: 58% of beta-lactams; 22% of fluoroquinolones; 5% of macrolides.

Moreover, 60% of our patients were classified ASA1.

Of the 92 obtained samples, 22 were positive (23.9%). The most frequently observed germs were the *Acinetobacter baumannii* (8.7%), *Pseudomonas aeruginosa* (6.5%), *Klebsiella pneumoniae* (4.3%), *Staphylococcus aureus* (4.3%). The *Acinetobacter baumannii* was the most resistant germ (60% sensibility to carbapenem).

These patients were followed until their D30 after surgery, 12 of them developed a post-operative pneumonitis with 4 cases of multi-resistant *Acinetobacter Baumanii*, 2 of which deceased.

**Conclusion** Pneumonitis after pulmonary resection are common and severe that’s why it is necessary to establish a global prevention strategy mainly based on general patricians and pneumologists’ awareness concerning the choice of the prescribed antibiotics, in order to avoid the spread of multi-resistant germs.

**Competing interests** None.

#### P156 The *Acinetobacter baumannii* (AB) in the severe burns

##### Rihi El Mehdi^1^

###### ^1^Intensive care unit, IBN ROCHD, Casablanca, Morocco

####### **Correspondence:** Rihi El Mehdi - mehdi_44@hotmail.fr

*Annals of Intensive Care* 2017, **7**(**Suppl 1**):P156

**Introduction** Infection is a major cause of morbidity and mortality in burned. The bacterial ecology varies among centers. Despite the progress in the management of severe burned, mortality remains very high. The aim of this study is to establish the pathogenic profile of AB in this population.

**Materials and methods** Single-center retrospective study of 7 months, including any serious burned hospitalized for more than 48 h in intensive care, and who benefited from bacteriological samples during his stay.

Infectivity was retained on a range of clinical and biological arguments (CDC criteria) changed). They excluded all burned died for non-infectious causes, and patients with isolated settlement.

**Results** Sixty-two (72) patients were infected by the AB during our study period. The sex ratio (M/F) was 1.7 and the mean age was 39 ± 23 years. Nosocomial pneumonia was present in 61.11% of cases. Urinary tract infection was present in 18.05% of cases. Bacteremia was present in 12.5% of cases. Skin infection was present 8.33% of cases. The resistance profile was marked by 100% of cases of resistance to third-generation cephalosporins (C3G), 88% of cases of resistance to fluoro-quinolones (FQ), 74% of cases of resistance to imipenem and 64, 28% of cases of resistance to tigecycline.

**Conclusion** The incidence of infection with *Acinetobacter baumannii* in our unit remains high compared to that of intensive care units. Colonization and infection by the AB are significantly associated with increased length of stay, and mortality, and given the gravity of hospitalized patients, failure to comply with hygiene and abusive use of antibiotic prophylaxis.

**Competing interests** None.

**Reference**

1. Coignard B, Lepoutre A, Desenclos JC. Lessons learned from implementing a mandatory notification of hospital acquired infections in France [cited June 11, 2006]. Lyon, France.

#### P157 Clinical impact of extended-spectrum β-lactamase producing Enterobacteriaceae colonization on pneumonia in ICU

##### Caroline Schimpf^1^, Assaf Mizrahi^2^, Benoît Pilmis^2^, Alban Le Monnier^2^, Kelly Tiercelet^1^, Mélanie Cherin^3^, Cédric Bruel^1^, Francois Philippart^1^

###### ^1^Réanimation, Groupe Hospitalier Paris Saint-Joseph, Paris, France; ^2^Unité de microbiologie clinique et dosages des anti-infectieux, Groupe Hospitalier Paris Saint-Joseph, Paris, France; ^3^Réanimation polyvalente adulte, Centre Hospitalier Intercommunal André Grégoire, Montreuil, France

####### **Correspondence:** Francois Philippart - fphilippart@gmail.com

*Annals of Intensive Care* 2017, **7**(**Suppl 1**):P157

**Introduction** ESBL are enzymes mostly found in *Enterobacteriaceae* and confer resistance to all beta lactams antibiotics except cefoxitin and carbapenems. Recently, a significant increase in the rate of ESBL-related infections in ICU makes difficult the choice of empiric antibiotic therapy, especially in patients colonized by extended-spectrum β-lactamase producing Enterobacteriaceae (ESBLe) [1]. Notably, very few data are currently available regarding the role of ESBLe colonization on further pneumonia involving the same bacteria [2]. The aim of our study was to describe the incidence of ESBLe infections among ESBLe-colonized ICU patients.

**Patients and methods** This study was conducted retrospectively from January 1st 2011 to May 1st 2016, in our intensive care department. All admitted ESBLe-colonized patients who develop an infection during their ICU stay have been included in the study. The only exclusion criterion was an antibiotic treatment for an ESBLe infection at ICU admission.

**Results** During the period of the study, 386 stays were associated with an ESBLe colonization in 384 patients. 148 infections were diagnosed in patients colonized by ESBLe, among which 78 pneumonias. In 18 cases (23%) the ESBLe was involved in the pulmonary infection (PN-ESBLe+) and was the only responsible bacterium in 66% of cases. The ESBLe was the same in screening and pneumonia in 15 cases (83%). The PN-ESBLe+ was associated with septic shock in 9 (50%) cases, acute respiratory distress syndrome in 2 (11%) cases and neurologic failure in 7 (39%) cases. Episodes were ventilator-associated pneumonia in 56% (10 cases) of PN-ESBLe+ and 52% (31 cases) of PN-ESLBe-. The most common pathogens involved were *Escherichia coli*, *Klebsiella pneumoniae*, and *Enterobacter cloacae* in both groups. Comparing groups (PN-ESBLe+ or PN-ESLBe−), only the notion of prior antibiotic therapy within 30 days (OR 3.9 [1.07–18.3]; p = 0.03) and colonization by ESBL *Klebsiella pneumoniae* (OR 4.04 [1.02–16.1]; p = 0.04) were more frequent in PN-ESBLe+. At least one empiric antibiotic was effective on the ESBLe in 83% of cases. In vitro antibiotic susceptibility tests demonstrate 100% efficiency of the association of piperacillin/tazobactam and amikacin on ESBLe involved in pneumonia.

Mortality at day 28 was 24% for PN-ESBLe+ and 44% for PN-ESBLe−. Hospital mortality was 53 and 38% respectively (p = NS).

Among 70 extra-pulmonary infections, ESLBe take part in 30 (43%) cases. The involvement of ESBLe was significantly lower in pneumonia than in other infections (p = 0.01).

**Discussion** Due to the single center character of our study, results cannot be extrapolated to the whole ICU population. Nevertheless, the observed incidence of colonizing ESBLe in our study is close enough from others studies. This point consolidates reflection about ICU pneumonia empiric treatment.

**Conclusion** The involvement of colonizing-ESBLe in ICU pneumonia is rare in our population and significantly lower than in other infections. Identified risk factors for PN-ESBLe + are a prior antibiotic therapy within 30 days and colonization with *K. pneumoniae*. Alternative associations to carbapenem remain efficient in all cases of pneumonia in our ICU and should probably be kept in mind.

**Competing interests** None.

**References**

1. Bretonniere C, Leone M, Milesi C, Allaouchiche B, Armand-Lefevre L, Baldesi O, et al. Strategies to reduce curative antibiotic therapy in intensive care units (adult and paediatric). Intensive Care Med 2015;41(7):1181–96.
2. Depuydt PO, Vandijck DM, Bekaert MA, Decruyenaere JM, Blot SI, Vogelaers DP, et al. Determinants and impact of multidrug antibiotic resistance in pathogens causing ventilator-associated-pneumonia. Crit Care 2008;12(6):R142.

#### P158 Risk factors of resistance for Gram negative bacilli responsible for ICU: acquired bacteremia—analysis of a large French ICU network

##### Sébastien Bailly^1^, Jc Lucet^2^, Alain Lepape^3^, François L’hériteau^4^, Martine Aupée^5^, Caroline Bervas^6^, Sandrine Boussat^7^, Anne Berger-Carbonne^8^, Anaïs Machut^9^, Anne Savey^10^, Jean-François Timsit^11^, REA-RAISIN Study group

###### ^1^Iame team 5, INSERM UMR 1137, Paris, France; ^2^Hygiène hospitalière, Hôpital Bichat-Claude Bernard (AP-HP), Paris, France; ^3^Réanimation, Hospices Civils De Lyon, Lyon, France; ^4^Médecine interne, Hôpital Bichat-Claude Bernard (AP-HP), Paris, France; ^5^Hygiène hospitalière, C.H.U de Rennes, Rennes, France; ^6^Pharmacie, CHU - Hôpitaux de Bordeaux, Bordeaux, France; ^7^Réanimation, CHRU Nancy, Nancy, France; ^8^Dgos, Ministère des Affaires sociales et de la Santé, Paris, France; ^9^Cclin sud est, Hospices Civils De Lyon, Lyon, France; ^10^Cclin, Hospices Civils De Lyon, Lyon, France; ^11^ Réanimation médicale et infectieuse, Hôpital Bichat-Claude Bernard, Paris, France

####### **Correspondence:** Sébastien Bailly - sbailly@chu-grenoble.fr

*Annals of Intensive Care* 2017, **7**(**Suppl 1**):P158

**Introduction** Immediate adequate treatment of ICU-acquired Gram negative bacilli (GNB) bloodstream infections (BSI) improves patients’ prognosis. Risk factors of resistance of GNB-BSIs should be better assessed.

**Materials and methods** Data from a large French national ICU network were explored during a 10-year period (2005–2014). Patients with a GNB-BSI were included and were divided into two groups according to the resistance (R) profile (BSI due to a R isolate or not). The following three groups were considered: (1) all GNB-BSI including *Pseudomonas* spp., *Acinetobacter* spp., *Stenotrophomonas* spp. and *Enterobacteriacae* (Eb) for which the following R were considered: ticarcillin (*Pseudomonas* spp., *Acinetobacter* spp., *Stenotrophomonas* spp.); ceftazidime (cefta) (*P. aeruginosa* (PA), *Acinetobacter* spp., *Stenotrophomonas* spp.), third generation cephalosporin (3GC) (Eb) and imipenem (all GNB, during the period 2011–2014 only), (2) PA cefta R from 2005 to 2014 and (3) Eb species resistant to 3GC from 2005 to 2014. Univariable hierarchical logistic models with two levels (random center and region effects) were used to select variables associated with resistance using a *p* value threshold of 0.2. Selected variables were further introduced in multivariable analyses using a hierarchical model with two random effects.

**Results** From 265,035 patients admitted in an annual median of 158 French ICUs, 9553 experienced an ICU-acquired (>48 h.) BSI, 5062 (53%) BSI due to GNB, including 1764 (35%) BSI due to R isolates. PA was identified in 1167 (23%) BSIs (480 (41%) R) and Eb in 3298 (65%) BSIs (1226 (34%) R). The median annual incidences of R GNB BSIs/10,000 ICU patients were: 68 for all R GNB BSI, 41 for Eb 3GC-R BSIs and 9.2 for PA cefta-R BSI. There was a significant increase of annual incidence for all GNB R and Eb 3GC-R BSI.

**Fig. 2:**
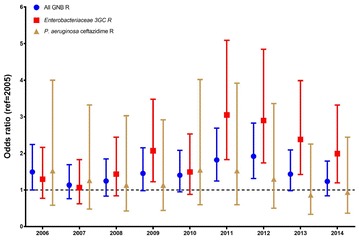
Evolution of the risk to have a BSI due to a resistant strain according to the year of ICU admission

**Fig. 3:**
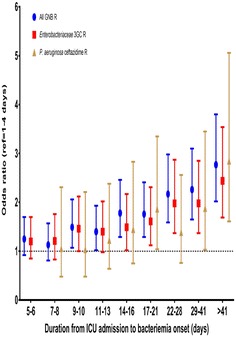
Days from ICU admission to infection

Independent factors associated with all R GNB BSI were: 1) ICU variables: percentage of patients with an immunosuppression other than neutropenia (7.9–14%: OR 1.23; 95% CI, [1.04–1.46]; >14%: 1.31 [1.09–1.57]); percentage of resistant GNB the previous year (55–66%: 1.87 [1.59–2.2]; >66%: 2.93 [2.43–3.53]) and 2) patient-variables: antimicrobial therapy at ICU admission (1.79 [1.55–2.08]); presence of an invasive device (CVC or intubation) (1.99 [1.25–3.16]) before infection; and one protective factor: trauma at ICU admission (0.76 [0.65–0.89]). The year effect was significant both for all R GNB and 3GC-R Eb but not for R PA. This effect was more pronounced for 3GC-R Eb, with an increase in the risk of R from 2005 to 2014 (Fig. 2). The duration from ICU admission to infection was the main risk factor of R for all BGN and sub-groups (Eb and PA): the probability of having a BSI due to a R strain increased with the time in ICU before infection (Fig. 3). ICU-based random effect remains significant indicating major impact of local epidemiology.

**Limitation** The absence of information about antibiotic consumption may partly explain the remaining significant center random effect in the final models.

**Conclusion** The duration from ICU admission to BSI was a main risk factor for a resistant isolate in GNB BSI. Resistance rates increased over time, especially for 3GC-R Eb and were highly dependent of local previous epidemiology.

**Competing interests** None.

#### P159 Sepsis at ICU admission due to extended-spectrum β-lactamase producing enterobacteriaceae among colonized patients: prevalence, risk factors and prognosis

##### Keyvan Razazi^1^, Jérémy Rosman^1^, Nicolas de Prost^1^, Guillaume Carteaux^1^, Chloe Jansen^2^, Jean Winoc Decousser^3^, Christian Brun-Buisson^1^, Armand Mekontso Dessap^1^

###### ^1^Réanimation Médicale, Hôpital Henri Mondor, Créteil, France; ^2^Cepi, Hospital Henri Mondor, Créteil, France; ^3^Microbiologie, Hôpital Henri Mondor, Créteil, France

####### **Correspondence:** Jérémy Rosman - jeremy.rosman@gmail.com

*Annals of Intensive Care* 2017, **7**(**Suppl 1**):P159

**Introduction** Prevalence of Extended-spectrum beta-lactamase-producing Enterobacteriaceae (ESBL-PE) carriers dramatically increases all over the world with a spread to the community. The increasing prevalence of ESBL-PE carriage at Intensive Care Unit (ICU) admission raises important questions on empiric therapy strategies in patients presenting with infection, which may include the use of a carbapenem as first-line therapy. Data on ESBL-PE sepsis at ICU admission among colonized patients are lacking.

**Patients and methods** We prospectively assessed between 2009 and 2015 the prevalence, risk factors and prognosis of ESBL-PE sepsis among ESBL-PE carriers at ICU admission. The following data were collected: demographic characteristics, which included sex, age, simplified acute physiology score (SAPS II), location before ICU admission, antibiotic exposure, surgery during the previous year, presence of underlying disease, Charlson comorbidity index, presence of indwelling devices and outcomes.

**Results** A total of 597 patients had ESBL-PE carriage detected at admission, corresponding to 9.5% of admitted patients. Among these patients, 325 patients had sepsis at ICU admission. Fifty patients (15.4%) had ESBL-PE related sepsis at ICU admission. ESBL-PE infection included 23 (46%) urinary tract infections, 14 (28%) pulmonary infections, 9 (18%) abdominal infections and 4 (8%) other infections. All but two ESBL-PE pneumonia cases were hospital-acquired (86%) while community-acquired ESBL-PE urinary tract infection was not uncommon (12/36 = 33%).

By multivariable analysis, prior urinary tract disease [OR 3.0 (1.1–8.0)], hospital-acquired sepsis at admission [OR 2.9 (1.4–5.7)], treatment with fluoroquinolone within the past 3 months [OR 2.8 (1.2–6.4)] past ESBL-PE infection [OR 2.8 (1.2–6.5)] were independent predictive factors for ESBL-PE sepsis at admission, whereas a pulmonary source of sepsis [OR 0.30 (0.15–0.61)] was protective. The final model showed a good calibration (chi^2^ = 3.7, p = 0.45) and discrimination (area under the curve = 0.85). Patients with ESBL-PE related sepsis had more often septic shock and bacteraemia at admission. ESBL-PE related sepsis was also more often associated with more frequent inadequate empirical therapy (68 vs 87%, p < 0.001). However, mortality did not differ significantly between patients with ESBL-PE infection and others (20 vs 22%).

**Conclusion** At ICU admission, ESBL-PE related sepsis was relatively infrequent among colonized patients. Our predictive factors for ESBL-PE may help choosing empiric therapy for sepsis among ESBL-PE carriers at ICU admission. The study did not show a significant association between ESBL-PE infection at admission and mortality.

**Competing interests** None.

**Reference**

1. Goulenok T, Ferroni A, Bille E, Lécuyer H, Join-Lambert O, Descamps P, Nassif X, Zahar JR. Risk factors for developing ESBL *E. coli*: can clinicians predict infection in patients with prior colonization? Hosp Infect. 2013;84(4):294–9.

#### P160 Carbapenemase-producing Enterobacteriaceae: experience of a Tunisian intensive care unit

##### Amira Ben Jazia^1^, A. M’rad^1^, Zouhour Ouali^2^, Manel Barghouth^1^, Y Blel^1^, N Brahmi^1^

###### ^1^Department of intensive care and toxicology, Centre d’Assistance Médicale Urgente, Tunis, Tunisia; ^2^Department of biology, Centre d’Assistance Médicale Urgente, Tunis, Tunisia

####### **Correspondence:** A. M’rad - mrad.aymen@gmail.com

*Annals of Intensive Care* 2017, **7**(**Suppl 1**):P160

**Introduction** Carbapenemase-producing Enterobacteriaceae (CPEc) are increasingly reported worldwide and constitutes a real challenge antibiotic for clinicians to preserve the bacterial ecology. Its incidence has remarkably increased in our intensive care unit during the last 5 years.

This work aims to focus on the impact of CPEc increasing in our intensive care unit.

**Patients and methods** A retrospective and descriptive study conducted in a Tunisian intensive care unit, including all hospitalized patients infected by an Enterobacteriaceae. We have determined annual incidence of CPEc from January 2008 to December 2015.

**Results** One thousand two hundred and three episodes of Enterobacteriaceae infections were eligible in 748 patients (324 male/424 female) aged between 14 and 88 years.

The global prevalence of CPEc across study period was 4.48% (54/1203).The prevalence of CPEc in each site was respectively of 23% in hemocultures, 18% in coproculture, 12% in catheters, 3.3% in respiratory tract, and 3% in urinary tract.

**Fig. 4:**
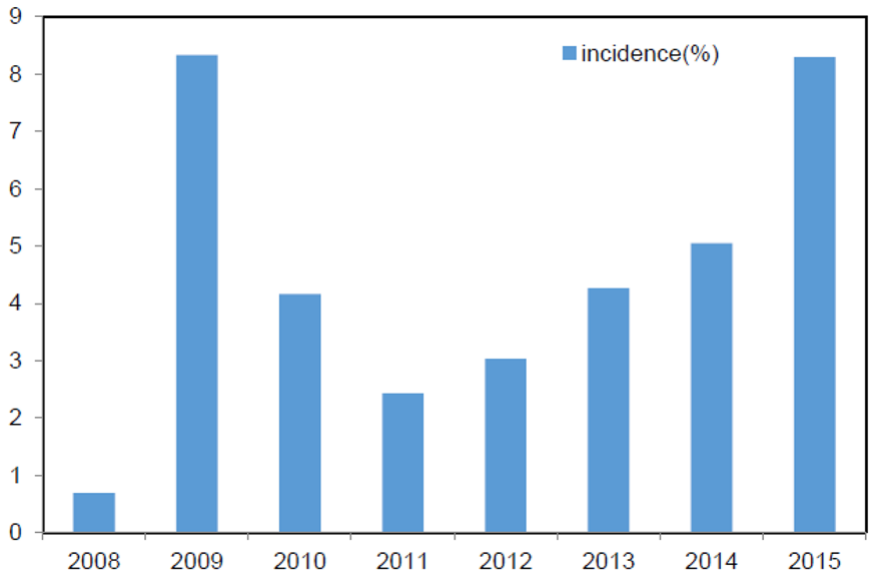
CPEc increasing from 2011 to 2015

The overall incidence of (CPEc) increased from 0.69% (1/144) in 2008 to 7.77% (15/193) in 2015 (Fig. 4).

**Conclusion**

Our study confirms the rapid spread of CPEc in Tunisian hospital and the urgent need for a well-structured and coordinated national surveillance plan in order to limit their dissemination.

**Competing interests** None.

#### P162 Extended spectrum beta lactamase producing enterobacteriacae (ESBL-PE) infections in ICU

##### Achille Kouatchet^1^, Rafael Mahieu^2^, Emmanuel Weiss^3^, David Schnell^4^, Jean-Ralph Zahar^5^

###### ^1^Service de Réanimation médicale et Médecine hyperbare, Centre Hospitalier Universitaire d’Angers, Angers, France; ^2^Réanimation médicale, Centre Hospitalier Universitaire d’Angers, Angers, France; ^3^Département d’anesthésie-réanimation, Hôpital Beaujon, Boulevard du Général Leclerc, Clichy, France, Clichy, France; ^4^Réanimation médicale, CHU de Strasbourg, Strasbourg, France; ^5^Laboratoire de bacteriologie-virologie-hygiene, Hôpital Avicenne, Bobigny, France

####### **Correspondence:** Achille Kouatchet - ackouatchet@chu-angers.fr

*Annals of Intensive Care* 2017, **7**(**Suppl 1**):P162

**Introduction** The ESBL spread has a major consequence in term of antibiotic choices. Carbapenem antibiotic are regarded as the most effective treatment. However numbers of authors suggest that alternatives antibiotics (i.e. noncarbapenems) could be used in ESBL-PE infections. There are some conflicting data regarding the use of alternatives in case of ESBL-PE infections. Moreover as far as we know, there are no data in ICU.

**Objectives** the aim of this study was to describe ESBL-PE infections in ICU and therapeutic options chosen in these specific situations.

**Patients and methods** Prospective multicentric observational cohort study conducted in volunteers ICU. All consecutive patients hospitalized in ICU with ESBL-PE infection according to CDC definitions were included. Severity of illness was defines according to bone criteria, SAPS II and SOFA. Demographic datas, empirical and definitive antibiotic therapy (ET and DT), clinical evolution, and outcome were recorded. In vitro antimicrobial susceptibility testing was performed by the disk diffusion method or the Vitek 2 system according to the guidelines of the Antibiogram Committee of the French Microbiologic society.

**Results** During the study period 146 patients with ESBL-PE infection met eligibility criteria with respectively a median age and SAPS II score of 63 (51–74) and 50 (38–70). The median SOFA Score at first day of antibiotic therapy and ICU admission were 7 (4–11) and 7 (5–11) respectively. The most frequent site of infection were respiratory tract (45%), urinary tract (20%) and abdominal (17%). The most frequent isolated species were: Escherichia coli (43%), Klebsiella sp (37%) and Enterobacter sp (18%). Respectively 50, 23 and 27% patients had septic shock, severe sepsis and sepsis according to Bone criteria.

Among ESBL-PE, 98.6% were carbapenem and 46.5 were BLBI sensitive. Among the whole population, 47 (48%) patients received a carbapenems as ET. 66 (68%) received a DT with carbapenems and 31 (32%) patients received an alternative DT. The most frequent reasons for maintaining carbapenems as DT were: Antibiotic susceptibility tests (38% of cases), severity level (33% of cases) immunosuppression (8% of cases). The Median length of ICU stay after infection was respectively 12 (6–27) and 11 (7–16) days for carbapenems and alternatives DT (p = 0.1). The D28 mortality was 24% for patients with carbapenems DT and 24% for patients with alternatives DT (p = 0.02).

Surprisingly, there were no differences between the 2 groups (carbapenems vs alternatives) in term of severity.

**Conclusion** Alternatives are frequently used for ESBL-PE infections in ICU. In our cohort 31 (32%) patients received antibiotics other than carbapenems regardless of the severity.

**Competing interests** None.

#### P163 Extended-spectrum beta-lactamase-producing enterobacteriaceae cross-transmission in the absence of private room in intensive care unit

##### Margaux Artiguenave^1^, Paktoris-Papine Sophie^1^, Florence Espinasse^2^, Faten El Sayed^3^, Aurélien Dinh^4^, Cyril Charron^1^, Guillaume Geri^5^, Antoine Vieillard-Baron^1^, Xavier Repessé^1^

###### ^1^Réanimation médico-chirurgicale, Assistance Publique - Hôpitaux de Paris, Hôpital Ambroise Paré, Boulogne-Billancourt, France; ^2^Equipe opérationnelle d’hygiène hospitalière, Assistance Publique - Hôpitaux de Paris, Hôpital Ambroise Paré, Boulogne-Billancourt, France; ^3^Service de microbiologie, Assistance Publique - Hôpitaux de Paris, Hôpital Ambroise Paré, Boulogne-Billancourt, France; ^4^Equipe mobile de microbiologie, Assistance Publique - Hôpitaux de Paris, Hôpital Ambroise Paré, Boulogne-Billancourt, France; ^5^Réanimation Médicale, Hôpital Cochin, Paris, France

####### **Correspondence:** Xavier Repessé - xavier.repesse@aphp.fr

*Annals of Intensive Care* 2017, **7**(**Suppl 1**):P163

**Introduction** Multidrug micro-organisms are responsible for longer hospitalisations and poorer outcomes in intensive care unit (ICU). The transmission of extended-spectrum beta-lactamase producing *enterobacteriaceae* (ESBL-PE) is prevented by the application of additional contact precautions, mainly relying on isolation in a private room and hand hygiene with waterless alcohol-based solution. Contact isolation cannot be achieved in our 12-bed ICU only composed of two twin bedrooms. We aimed at reporting the ESBL-PE acquisition in this peculiar architectural form of ICU and at studying the impact of twin bedrooms on ESBL-PE cross-transmission.

**Patients and methods** An observational and non-interventional study was prospectively conducted in the 12-bed ICU of a university hospital Ambroise Paré (Boulogne-Billancourt, France). Inclusion criteria were: (1) adult patients and (2) a period of hospitalisation allowing the patient to be nursed by at least two paramedical teams. Characteristics of patients at admission (age, sex, SAPSII) and clinical data during hospital stay (duration of mechanical ventilation, duration of ICU stay, outcome) were prospectively collected. Microbiological data concerning ESBL-PE imported and acquired carriage were monitored by rectal swabs collected at admission and once weekly every Monday for the whole duration of the ICU stay. ESBL imported carriage was defined as a first screening positive for ESBL whereas ESBL acquired carriage as a negative first screening at admission followed by at least one positive rectal swan. Mechanistic of a potential cross-transmission was studied following a three-step process consisting in (1) identifying patients considered as possible ESBL sources (index patients) for transmission, (2) classifying each ESBL strain according to the CTXm 1 and 9 groups and (3) diagnosing potential cross-transmission by gene sequencing of remaining cases of possible transmission.

**Results** From June 2014 to April 2015, 550 patients were admitted in the ICU, among which 470 followed the inclusion criteria. The rate of ESBL colonization at admission was 13.2% (n = 62), mainly with *Escherichia coli*. Two hundred and twenty-one non-colonized patients were screened at least twice. The incidence of ESBL acquisition was 4.1% (9 patients on 221), also mainly with *Escherichia coli*. Mortality did not differ between ESBL carriers and non-carriers. In univariate analysis, ESBL acquisition was associated with the Injury Global Score II (IGSII) and the Sequential Organ Failure Assessment (SOFA) at admission, the need for catecholamine and the ICU length of stay (LOS). In multivariate analysis, ICU LOS and IGSII at admission were the strongest risk factor for ESBL acquisition. The nine ESBL-acquired carriers had one to three index patients defined as a patient hospitalized who shared at least 1 day hospitalization. The CTXm grouping of the ESBL strains excluded a cross-transmission for 4 patients. The gene sequencing did it for 3 others and confirmed a cross-transmission in only two patients (0.8%). The cross-transmission emanated from the same source of a CTXm 1 ESBL-producing *E. coli*. This patient shared 1 day in a different unit with the first acquired carrier and 2 days in the same unit with the other. No case of cross-transmission in the same room was observed.

**Conclusion** The rate of 13.2% of ESBL carriage on admission was comparable to other rates in French ICUs (15%). Despite the absence of contact isolation, the incidence of ESBL acquisition was 4.1% which is actually lower than transmission rates previously published in other ICUs. A cross-transmission concerned two ESBL-acquired carriers only and resulted from the same index patient during short shared hospitalizations of 1 day in a different unit and 2 days in the same unit. Our results question whether the contact isolation in private rooms plays a major role for the prevention of ESBL cross-transmission in ICU, although the external validity of our results could be questionable.

**Competing interests** None.

#### P164 Prevalence of colonization with extended spectrum B-lactamase producing bacteria and subsequent ICU acquired infection in French Guiana

##### Hatem Kallel^1^, Claire Mayence^1^, Stéphanie Houcke^1^, Pascal Guegueniat^1^, Didier Hommel^1^

###### ^1^Intensive care unit, Hospital, Cayenne, French Guiana

####### **Correspondence:** Hatem Kallel - kallelhat@yahoo.fr

*Annals of Intensive Care* 2017, **7**(**Suppl 1**):P164

**Introduction** Bacterial resistance to antibiotics is a common problem worldwide. In South America, this prevalence is reported to be the highest in the world. However, in French Guyana, there is no data on the epidemiology of colonization and infection caused by extended spectrum B-lactamase producing enterobacteriaceae (ESBL-PE). We conducted this study to investigate the prevalence of colonization with ESBL-PE and subsequent ICU acquired infection in French Guiana.

**Materials and methods** A 24 months (January 2014 to December 2015) observational study in a 14 beds ICU in a general hospital. Our unit, is the sole and the referral one of all French Guiana department.

**Results** Over the study period, 670 patients were admitted to ICU and 603 of them (90%) were hospitalized more than 48 h. The mean occupancy rate was 82.5 ± 20.6% and the mean colonization index (with ESBL-PE) was de 37 ± 18.1%. The mean age was 43.4 ± 21.1 years. The sex-Ratio (M/F) was 1.3. The mean IGS II calculated at admission to ICU was 44.6 ± 24.2. The most recorded organ failures at admission to ICU were respiratory and hemodynamic ones (56.7 and 37.2% respectively). At admission to ICU, 44.2% of patients presented active infection and 57.3% received antibiotics. Multidrug resistant (MDR) bacteria carriage was found in 88 patients (13.4%) at ICU admission and was acquired in ICU in 89 other patients (13.4%). The most isolated MDR bacteria at admission were ESBL producing *E coli* and *K. pneumoniae*. However, the most isolated MDR bacteria during ICU stay were ESBL producing *K. pneumoniae* and *E cloacae*. During the ICU stay, 98 patients (14.6%) had presented 147 episodes of ICU acquired infections (ICU-AI). Over the 177 patients carrying MDR bacteria, 159 (89.8%) carried ESBL-PE and 66 developed ICU-AI. ESBL-PE caused 21.2, 37.5, 20, and 66.7% of 1st, 2nd, 3rd and 4th ICU-AI episodes respectively. Statistical analysis didn’t show any link between ESBL-PE carriage and a first episode of ICU-AI caused by ESBL-PE.

**Conclusion** Our study show a high prevalence of ESBL-PE bacteria carriage at admission in our ICU. ESBL-PE carriage was not associated to higher prevalence of ICU-AI caused by the same microorganism. This finding can help to reduce the inappropriate use of carbapenems in such conditions.

**Competing interests** None.

#### P165 On-line hemofiltration versus conventional hemofiltration in septic shock patients: clinical safety and effectiveness

##### Kaouther Dhifaoui^1^, Zied Hajjej^1^, Amira Fatnassi^1^, Walid Sellami^1^, Iheb Labbene^1^, Mustapha Ferjani^1^

###### ^1^Department of critical care medicine and anesthesiology, Military Hospital of Tunis, Tunisia, Tunis, Tunisia

####### **Correspondence:** Zied Hajjej - hajjej_zied@hotmail.com

*Annals of Intensive Care* 2017, **7**(**Suppl 1**):P165

**Introduction** The implementation of hemofiltration (HF) as a renal replacement therapy in septic shock patients requires the supply of large quantities of replacement solutions. These solutions are either industrially prepared in autoclaved expensive plastic bags (conventional hemofiltration, CHF) or continuously provided in unlimited amounts at the dialysis machine directly from the water treatment plant to form the replacing solutions (on-line hemofiltration, OLHF).The aim of our study was to evaluate the safety and effectiveness of on-line hemofiltration compared to conventional hemofiltration in septic shock patients.

**Patients and methods** The investigative protocol was approved by the Institutional Ethics Authorities and all patients or their legally authorized representatives provided written informed consent. It was a prospective, randomized, clinical study, including septic shock patients with acute renal failure. Patients were randomized to receive either on-line hemofiltration (n = 8) or conventional hemofiltration (n = 25) for renal replacement therapy during 4 days. Hemodynamic monitoring was conducted by conventional devises, including: electrocardiogram and a radial arterial catheter for invasive arterial pressure every 6 h during period study. We collected serum samples also every 6 h (urea, potassium and sodium levels, troponin, hemoglobin, platelets, C-reactive protein and lactates).

**Results** The evolution of heart rate (HR), mean arterial pressure (MAP), biological markers were comparable between the two groups over time except a significant decrease in MAP in the OLHF group compared to CHF group only at H6 (P = 0.008) and H12 (P = 0.015) and a significant decrease in C-reactive protein level in the OLHF group at H48 (P = 0.02).

**Conclusion** On-line hemofiltration seems to be a safe and reliable method of renal replacement therapy in septic shock patients. It may be associated with attenuated pro-inflammatory cytokine profile (C-reactive protein).

**Competing interests** None.

#### P166 Usefulness of biological testing during renal replacement therapy in ICU patients

##### Fahmi Dachraoui^1^, Sabrine Nakkaa^1^, Abdelwaheb M’ghirbi^1^, Ali Adhieb^1^, Dhouha Ben Braiek^1^, Kmar Hraiech^1^, Ali Ousji^1^, Islem Ouanes^1^, Hammouda Zaineb^1^, Saousen Ben Abdallah^1^, Lamia Ouanes-Besbes^1^, Fekri Abroug^1^

###### ^1^Réanimation polyvalente, CHU Fatouma Bourguiba, Monastir, Tunisia

####### **Correspondence:** Fahmi Dachraoui - dachraoui.fahmi@gmail.com

*Annals of Intensive Care* 2017, **7**(**Suppl 1**):P166

**Introduction** Clinical and biological monitoring of efficacy and safety of RRT sessions is thought useful and in many ICUs biological testing at mid RRT session and at its end is routinely performed. The aim of the present study is to evaluate the impact of laboratory tests performed during RRT session on clinical decision making and treatment alteration.

**Patients and methods** Retrospective study including all consecutive patients hospitalized in the medical ICU of the University Hospital Monastir, Tunisia between January 2015 and September 2016, requiring intermittent hemodialysis performed in the ICU. For each patient we collected demographic characteristics (age, sex, SAPS III, diagnosis, comorbidities), indication of the RRT, clinical and biological parameters before, during and at the end of RRT session. Based on the patient monitoring records during each RRT session we identified therapeutic interventions started before the end of the RRT session in the light of the results of laboratory tests performed during the session (usually in the middle the session): infusion of glucose, potassium, transfusion, extension of the session.

**Results** During the study period, 370 patients were admitted to the ICU. Of these 24 patients required acute hemodialysis. The median age of these patients were 54 years (IQR = 31), 53% of them were female. The main comorbidities were hypertension, diabetes, chronic renal failure (CRF) with preserved diuresis, respectively in 55, 38 and 36%. ¾ of the patients included were in septic shock and had median SAPS III score of 92 (IQR = 34). Anuria, pulmonary oedema, hyperkalemia, and acidosis indicated RRT sessions respectively in 47.1, 24.5, 8.8, 9.8 and 9.8%. Results of laboratory tests performed during RRT sessions prompted a specific attitude in the following rates: infusion of glucose in 35.7%, addition of potassium in 22%, and extension of the session duration 15.2%.

**Conclusion** The practice of laboratory tests during the RRT sessions seems useful since it could impact clinical decision making in more than one-third of sessions.

**Competing interests** None.

#### P167 Feasibility of regional citrate anticoagulation for membrane-based therapeutic plasma exchange in ICU

##### Simon Klein^1^, Mattéo Miquet^1^, Jean-Marc Thouret^1^, Vincent Peigne^1^

###### ^1^Réanimation, Centre Hospitalier Métropole-Savoie, Chambéry, France

####### **Correspondence:** Vincent Peigne - vincentpeigne@yahoo.fr

*Annals of Intensive Care* 2017, **7**(**Suppl 1**):P167

**Introduction** Therapeutic plasma exchange (TPE) is crucial for the management of auto-immune diseases like thrombotic thrombocytopenic purpura or myasthenia gravis. TPE is performed either by centrifugation, with specific machines which are not routinely available in ICUs, or by using specific plasma separation membranes with widely spread in ICUs hemofiltration machines. Regional citrate anticoagulation for TPE is well established with centrifugation but has been seldom described for membrane TPE. We are reporting the experience of our ICU in this field.

**Patients and methods** Retrospective study including all patients who received TPE with citrate regional anticoagulation between 2013 and 2016 in an 18-bed ICU. TPE is performed solely in the ICU in our institution.

**Results** 26 patients were included. TPE was required for thrombotic microangiopathy (13 patients), vasculitis (6 patients), hyperviscosity syndrome (2 patients), Guillain–Barré syndrome (2 cases) and others (3 patients). Mean SAPS2 score was 32 [standard deviation (SD) 16.6]. 281 TPE were performed, with a mean number of 10.5 (SD 11.5; range 2–57) TPE per patients. Coagulation of the circuit of TPE occurred in 10 (38%) patients. Coagulation of the circuit occurred in 10.3% (29/281) of the TPE. Minor adverse events have been reported in two patients: one had a rash during the first TPE (no recurrence during the 56 next TPEs) and the other had paresthesia during the first two TPEs (the calcium infusion was increased and there had been no recurrence during the 25 next TPEs). No serious adverse events related to citrate were observed.

**Conclusion** Regional anticoagulation with citrate allowed us to perform TPE in 26 patients, without significant adverse events. The rate of circuit coagulation was 10.3% per TPE.

**Competing interests** None.

#### P168 Modelization of the cost-effectiveness of anti-thrombin to reduce the incidence of membrane thrombosis during continuous hemofiltration

##### Vincent Peigne^1^, Jean-Louis Daban^2^, Mathieu Boutonnet^2^, Bernard Lenoir^3^

###### ^1^Réanimation, Centre hospitalier Métropole Savoie, Chambéry, France; ^2^Réanimation, Hôpital d’Instruction des Armées Percy, Clamart, France; ^3^Département d’anesthésie-réanimation, Hôpital d’Instruction des Armées Percy, Clamart, France

####### **Correspondence:** Vincent Peigne - vincentpeigne@yahoo.fr

*Annals of Intensive Care* 2017, **7**(**Suppl 1**):P168

**Introduction** A reduced incidence of membrane thrombosis after injection of Anti-thrombin (AT) has been reported in septic patients with acquired deficit in AT undergoing continuous hemofiltration. As this strategy was routinely performed in our unit until 2012, we investigated its cost-effectiveness.

**Patients and methods** Data about the use of hemofiltration, the consumption of AT and hemofiltration devices during 2011 (period with routine use of AT) and 2012 (period with use of AT only if a membrane thrombosis occurred) were extracted from the administrative database of the institution. A decisional tree was built to modelize the impact of AT on the consumption of hemofiltration devices and blood products. The decisional tree took into account the probability of membrane thrombosis with and without AT and the probability of transfusion after membrane thrombosis. Costs were obtained from the pharmacy of the institution (AT, hemofiltration devices) and from the literature (blood products).

**Results** During 2011, 77 days of hemofiltration were performed, with the use of 45 doses of AT (23,202€) and 76 hemofiltration devices (11,632€). During 2012, 76 (−1%) days of hemofiltration were performed, with the use of 5 (−89%) doses of AT (2578€) and 85 (+10%) hemofiltration devices (13,443€). The mean cost of 1 day of hemofiltration decreased from 449€ to 211€ with the diminution of the use of AT.

According to the decisional tree, AT was almost never cost-effective. The only circumstances associated with a benefit for the use of AT was the association of a probability of thrombosis with AT inferior to 0.1, of a probability of thrombosis without AT equal 1, of a probability of transfusion after thrombosis equal 1 and a cost of transfusion of 424€. In these extremely favorable circumstances, AT could decrease the daily cost of hemofiltration of 2.22–19.30€.

**Discussion** The model has several limits: the losses of utility related to transfusion and to interruption of hemofiltration due to thrombosis were not taken into account; the cost of AT measurement was not estimated; the work load of changing a membrane and of transfusion after membrane thrombosis was not analyzed.

**Conclusion** Our results suggest that anti-thrombin is not cost-effective to reduce the costs of hemofiltration related to membrane thrombosis.

**Competing interests** None.

#### P169 Vascular access sites for acute renal replacement in intensive care unit

##### Amira Ben Jazia^1^, Amira Jamoussi^2^, Takoua Merhbene^3^, Dhouha Lakhdhar^4^, Jalila Ben Khelil^2^, Mohamed Besbes^2^

###### ^1^Medical ICU, Hospital Abderrahmen Mami De Pneumo-Phtisiologie, Ariana, Tunisia; ^2^Réanimation médicale, Hôpital Abderrahmen Mami, Ariana, Tunisia; ^3^Réanimation respiratoire, Hôpital Abderrahmen Mami de pneumo-phtisiologie, Ariana, Tunisia; ^4^Service de réanimation médicale, Centre d’assistance médicale-urgente, Tunis, Tunisia

####### **Correspondence:** Amira Ben Jazia - amira26juillet@yahoo.fr

*Annals of Intensive Care* 2017, **7**(**Suppl 1**):P169

**Introduction** Several temporary venous catheterizations are sometimes required for acute renal replacement therapy (RRT) in the intensive care unit (ICU). This study compares catheterizations in the femoral and jugular veins in terms of patient safety.

**Materials and methods** This was a descriptive retrospective review of dialysis sessions (DS) records monitoring performed in patients older than 17 years hospitalized in medical intensive care unit between April 2011 and December 2015.

A study of dialysis catheter, was conducted in critically ill adults requiring RRT was performed.

Catheter insertion site, catheter age and urea reduction ratio (URR) were analyzed.

**Results** URRs were analyzed from 330 dialysis sessions (*n* = 64 patients). The mean rate of URRs was 52.8 ± 12.4. Only 31.4% of dialysis sessions (DS) were efficient with URR ≥ 60.

This study analyzed 64 patients who underwent two different sites of catheterization: the femoral and jugular site.

The mean age of cathéters was 1.613 days.

No significant difference (*P* = 0.18) in the efficiency of (DS) was detected between sessions performed through femoral (*n* = 225; 68%) and jugular (*n* = 105; 32%) dialysis catheters.

**Conclusion** Femoral and internal jugular acute vascular access sites are both acceptable for RRT therapy in the ICU. The effectiveness of (DS) in the ICU is low (31.4%). An analysis of predictive factors of inefficiency of (DS) is expected to improve our results.

**Competing interests** None.

**Reference**

1. Ridel C, Baldea MC, Rondeaua E, Vinsonneaub C. La dose de dialyse en réanimation: existe-t-il vraiment un idéal? Dose of dialysis in intensive care unit.

#### P170 Renal replacement therapy protocol with regional citrate anticoagulation: observational study of efficacy with a new post-filter ionized calcemia target

##### Celine Derreumaux^1^, Thierry Seguin^1^, Jean-Marie Conil^1^

###### ^1^Réanimation polyvalente, Hopital Rangueil, Toulouse, France

####### **Correspondence:** Celine Derreumaux - celine.derreumaux@gmail.com

*Annals of Intensive Care* 2017, **7**(**Suppl 1**):P170

**Introduction** In Intensive Care Unit (ICU), some patients suffering from acute kidney injury need renal replacement therapy (RRT). It requires the circuit anticoagulation, this could be done by a regional citrate method. Today, this is a recommended approach for the everyday care, even if the technique isn’t widespread yet [1]. The ionized calcemia dosing through the filter (“post-filter” ionized-calcemia) is used to monitor the technique efficacy, with a target of 0.25–0.35 mmol/L showing a good filter anticoagulation.

The objective of our study was the assessment of efficacy and safety of our regional citrate anticoagulation protocol, with a less restrictive post-filter ionized calcemia target (0.3–0.6 mmol/L). The main goal was the analysis of the circuit lifespan, considering a lifespan above 24 h, as well as the search of some clinical and biological factors affecting the technique efficacy. Moreover, we analyzed the side effects incidence of the protocol (hypernatremia, metabolic alcalosis), and their consequences. The study received the scientific ethical agreement of University Hospital of Toulouse, and is registered with number 18-0214.

**Patients and methods** 57 patients, admitted to one of the two University Hospital ICUs of Toulouse, needing a continuous RRT method, without any need for systemic heparin anticoagulation, and without severe hepatocellular failure, were included in the study. 103 filters included over a 1-year period were analyzed.

**Results** Results show a mean filter lifespan of 48 h, with a lifespan above 24 h for 85.4% of all filters. Coagulation was the cessation reason for 29.1% of filters, most of them before 24 h of the filter use. A value of post-filter ionized calcemia at day 1 below 0.54 mmol/L was the main factor influencing a filter lifespan above 24 h. An age older than 51 and a SAPS II severity score below 80 were other factors conditioning a filter lifespan of more than 24 h. Side effects of citrate were rare and didn’t have any clinical impact among our patients.

**Discussion** These results suggest that citrate used for anticoagulation in RRT could have an additional anti inflammatory effect through the induced hypocalcemia, as well as an energetic gain which could lead to a renal protection against ischemia–reperfusion mechanism [2]. Moreover, these results call into question the need of post-filter ionized calcemia dosing for the monitoring of citrate anticoagulation efficacy, since the method safety is monitored by the total-to-ionized calcium ratio.

**Conclusion** During continuous RRT in ICU, a regional citrate anticoagulation protocol with a non-restrictive post-filter ionized calcemia target seems to be efficient and could reduce side effects. These results need to be confirmed with a randomised control study.

**Competing interests** None.

**References**

1. KDIGO Clinical Practice Guideline for Acute Kidney Injury. Kidney international Supplements, 2012.
2. Oudemans-van Straaten, HM et al. Citrate anticoagulation for continuous venovenous hemofiltration. Crit Care Med, 2009;37(2):545–52.

#### P171 Impact of the use of an oXiris filter versus an AN69ST filter on the duration of hemofiltration in intensive care

##### Charlotte Kelway^1^, Valery Blasco^1^, Cyril Nafati^1^, Karim Harti^1^, Laurent Reydellet^1^, Jacques Albanese^1^

###### ^1^RPPF, Hopital de la Timone, Marseille, France

####### **Correspondence:** Charlotte Kelway - chakel@hotmail.com

*Annals of Intensive Care* 2017, **7**(**Suppl 1**):P171

**Introduction** Continuous veno-venous haemofiltration (CVVH) is used to treat acute kidney injury in critically ill patients. To optimize its efficiency, CVVH requires effective anticoagulation. Systemic anticoagulation with standard heparin, the most used, can lead to major bleeding complications. Hemofilters that are able to adsorb heparin molecules on their surface such as AN69ST and oXiris membranes represent an alternative. The objective of this study was to compare these two types of filters in terms of duration, efficiency, dysfunctions and cost.

**Materials and methods** From October 2012 to May 2014, we conducted a retrospective, observational, and non-interventional study. All patients admitted in the intensive care unit needing CVVH were included. The primary endpoint was the filter lifespan: AN69ST versus oXiris. The secondary endpoint was the filter efficiency (urea reduction ratio: URR). The main analysis did not consider the anticoagulation type. We conducted a subgroup analysis taking into account the use or not of an anticoagulation.

**Results** 181 sessions in 93 patients were carried out using 386 filters representing 10,706 h of treatment. The mean AN69ST filter lifespan was 27 ± 20 h and 28 ± 22 h for oXiris filters (*p* > 0.05). There is no significant difference in terms of duration between the two filters. The subgroup analysis taking into consideration the use or not of anticoagulation did not show any difference either. The mean URR was 48 ± 23% in the AN69ST group and 44 ± 25% in the oXiris group (*p* > 0.05). Concerning the dysfunctions, there were no significant difference between the two filters. One hundred and seventy-six AN69ST filters were used for a total cost of 24,288 euros. Two hundred and ten oXiris filters were used for a total cost of 39,060 euros.

**Conclusion** The AN69ST and oXiris lifespans are not significantly different. They were as efficient in terms of blood epuration and had as many dysfunctions. The use of an oXiris filter rather than an AN69ST to extend the circuit’s lifespan in the same clinical conditions is not justified considering the extra cost generated.

**Competing interests** None.

**References**

1. Zhonghua Wei Zhong Bing Ji Jiu Yi Xue 2015;27(5);343–8.
2. Intensive Care Med 2012; 38(11):1818–25.
3. PLoS One 2014;9(5):e97187.

#### P172 Effects of early use of diuretics in patients at risk of acute renal failure and oliguria

##### Narjess Ben Aicha^1^, Khaoula Meddeb^1^, Ahmed Khedher^1^, Jihene Ayachi^1^, Nesrine Fraj^1^, Nesrine Sma^1^, Imed Chouchene^1^, Mohamed Boussarsar^2^

###### ^1^Réanimation médicale, CHU Farhat Hached, Sousse, Tunisia; ^2^Réanimation médicale, CHU Farhat Hached. Research Laboratory N° LR14ES05. Faculty of Medicine, Sousse, Tunisia

####### **Correspondence:** Mohamed Boussarsar - hamadi.boussarsar@gmail.com

*Annals of Intensive Care* 2017, **7**(**Suppl 1**):P172

**Introduction** Because oliguria is a poor prognostic sign in patients with acute renal failure (ARF), diuretics are often used to increase urine output in patients with or at risk of ARF. From a pathophysiological point of view there are several reasons to expect that loop diuretics could have a beneficial effect on renal function. However, a review of literature shows that the use of loop diuretics in patients with ARF has been associated with inconclusive results despite the theoretical benefits [1].

To assess the adjunctive effect of diuretics, to alter the progression to kidney injury or failure, in patients at risk for acute renal failure.

**Patients and methods** This is a retrospective chart review of consecutive patients who developed ARF with oliguria in the intensive care unit. Chart abstractors were well trained residents. Two chart reviewers (senior intensivists) studied all the charts. An explicit protocol was used to precise all needed definitions. Uniform handling of data was ensured especially for conflicting, missing or unknown data. Oliguria was defined as urine output lower than 0.5 ml/kg/h for at least 3 h.

RIFLE score was assessed before and after urinary output normalisation. Therapeutic intervention to optimize pre-renal perfusion was described. Mean arterial blood pressure (MBP) before and after therapeutic initiation, oliguria duration, delay from oliguria onset to diuretic administration, delay from diuretic administration to urinary output normalisation were measured.

**Results** 23 patients were studied over a 2 years period. They were 63 [24, 87] median (IQR) aged, with diabetes mellitus, 22%; hypertension, 47.8%; cardiac failure, 32% and chronic respiratory failure, 43%. Chronic and obstructive kidney diseases were excluded. Median SAPS II was 37 [23, 75]. 80% were on mechanical ventilation.

RIFLE score before diuretics administration was assessed at oliguria onset as (patients without risk, zero; R, 69%; I, 17%; F, 10%; L, 4%; E, zero). Fluid resuscitation after oliguria onset was administered in 77% and vasopressors in 80%. Median (IQR) delay from oliguria onset to diuretic administration was 5 [0.5, 22] h while optimization of pre-renal hemodynamic disturbances was already achieved.

The median (IQR) MBP before and after therapeutic intervention was respectively, 74 [46, 100] and 95 [69, 110] mmHg. Median (IQR) delay from initiation of therapeutic intervention and MBP improvement was 1.5 [0, 3] h. The delay from diuretic administration to urinary output normalization was 3 [0.5, 27] h.

**Fig. 5:**
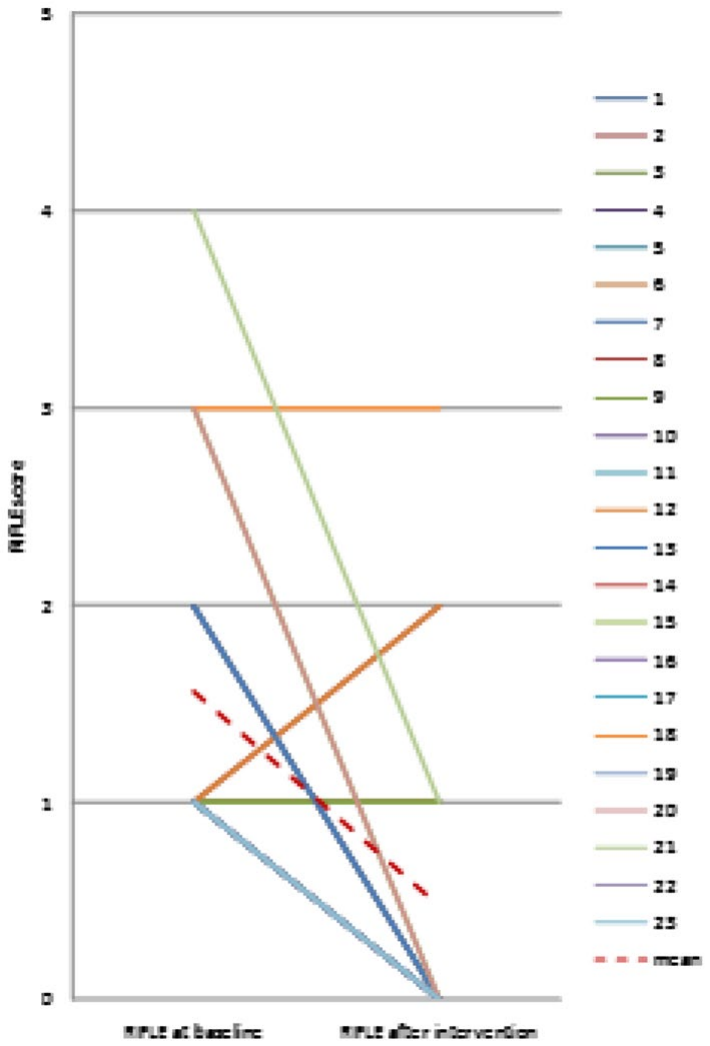
Progression of RIFLE score classes respectively from baseline to after therapeutic intervention

After resumption of diuresis, RIFLE score was assessed as (patients without risk, 74%; R, 17%; I, 8%; F, 1% L, zero; E, zero) (Fig. 5). Increased serum creatinine level, above 1.5 fold normal range, was observed only in 6 (26%) patients.

**Conclusion** Rapid optimization of pre-renal hemodynamic disturbances associated with short delay administration of diuretics could significantly alter the progression to kidney injury or failure in at risk acute renal failure ICU patients.

**Competing interests** None.

**Reference**

1. Ho KM, Power BM. Benefits and risks of furosemide in acute kidney injury. Anaesthesia. 2010;65(3):283–93. doi:10.1111/j.1365-2044.2009.06228.x. Epub 2010 Jan 19.

#### P173 Epidemiology and risk factors of *Acinetobacter baumannii* ventilator associated pneumonia

##### Walid Sellami^1^, Zied Hajjej^1^, Soumaya Ben Yedder^2^, Walid Samoud^1^, Bousselmi Radhouene^1^, Bousselmi Mariem^3^, Iheb Labbene^1^, Mustapha Ferjani^1^

###### ^1^Department of critical care medicine and anesthesiology, Military Hospital of Tunis, Tunisia, Tunis, Tunisia; ^2^Department of critical care medicine and anesthesiology, Military hospital of tunis, tunisia, Tunis, Tunisia, Tunisia; ^3^Department of critical care medicine and anesthesiology, Military Hospital of Tunis, Tunisia, tunis, Tunisia

####### **Correspondence:** Walid Sellami - drsellamiwalid@yahoo.fr

*Annals of Intensive Care* 2017, **7**(**Suppl 1**):P173

**Introduction** The ventilator associated pneumonia (VAP) is a common and severe complication of assisted ventilation. It’s the leading cause of nosocomial infections in intensive care unit and remain responsible for a high morbidity and mortality because of the emergence of multi-drug resistant (MDR) bacterial agent such us *Acinetobacter baumannii* (AB). The aim of this study was to determine the incidence, risk factors and prognosis of AB VAP.

**Patients and methods** Retrospective study extending over a 5 year period (January 2010–January 2016) that included all patients over 18 years and ventilated more than 48 h and developing AB VAP. Patients were divided into two groups: one consisting of patients who developed VAP to AB and the second developed VAP to another bacterial pathogen.

**Results** One hundred and forty patients developed VAP. The incidence rate of AB VAP was 15.3% with a density of incidence of 20.3 per 1000 ventilator days. Age, male gender, the time between hospitalization and mechanical ventilation and the medical pathology were risk factors for developing AB VAP. AB was resistant to ceftazidime in 100%, to imipenem in 65%, tobramycin in 70% and netilmycin in 35.3%, rifampin in 85% with a sensitivity to colistin in 100% of cases. The resistance of this germ to imipenem increased from 35% in 2010 to 88.5% in 2016. The evolution of patients with AB VAP developed frequently septic shock compared to other patients (44 vs 19.3%; p = 0.038). The AB VAP mortality was higher (50 vs 33%; p = 0.03).

**Conclusion** The increasing incidence of multi-drug resistant AB VAP is responsible for a high morbidity and mortality. So we need to identify risk factors and to strengthen the means of prevention of hand contamination and cross transmission during invasive procedures.

**Competing interests** None.

#### P174 Incidence and risk factors of central line associated bloodstream infections and its risk factors in a Tunisian medical intensive care unit

##### Nesrine Sma^1^, Asma Ammar^2^, Khaoula Meddeb^1^, Asma Ben Cheikh^2^, Hend Ben Lakhal^1^, Jihene Ayachi^1^, Ahmed Khedher^1^, Nesrine Fraj^1^, Messaouda Khelfa^1^, Yamina Hamdaoui^1^, Imed Chouchene^1^, Nabiha Bouafia^2^, Mohamed Boussarsar^3^

###### ^1^Réanimation médicale, CHU Farhat Hached, Sousse, Tunisia; ^2^Hospital hygiene unit, Farhat Hached Hospital, Sousse, Tunisia; ^3^Medical intensive care unit, Farhat Hached Hospital. Research Laboratory N° LR14ES05. Faculty of Medicine, Sousse, Tunisia

####### **Correspondence:** Mohamed Boussarsar - hamadi.boussarsar@gmail.com

*Annals of Intensive Care* 2017, **7**(**Suppl 1**):P174

**Introduction** Central line associated bloodstream infections (CLABSI) are among the serious hospital-acquired infections. The aim of this study is to determine the incidence of CLABSI, the pathogens and the risk factors that play a role in the development of BSI among patients followed in a Tunisian medical intensive care unit.

**Patients and methods** All patients admitted for more than 48 h were included in the study over a 1-year period in an 8-bed medical ICU. The enrollment was based on clinical and laboratory diagnosis of BSI. Blood samples were collected from catheter hub of all patients for culture, followed by identification and antibiotic sensitivity testing of the isolates. For all subjects, age, sex, underlying diseases, SAPS II score, ICU length of stay, invasive procedures and their durations (mechanical ventilation, central catheterization, urinary catheterization) were recorded. Risk factors were evaluated by a multivariate logistic regression model.

**Results** Among a total of 237 admissions from September 15th 2015 to September 15th 2016, 163 (68.7) patients were eligible. One hundred twenty-five (76) patients had a central line. A total of 27 episodes of CLABSI were assessed in 23 (18.4) patients. The mean SPASII of patients with CLABSI was 33 ± 15.4. Their mean CHARLSON index was 1.8 ± 1.7, median duration of catheterization was 4 [1.5–7] days and 8 (34.8) had more than one catheterization attempt. The rate of CLABSI was 19.2/1000 catheter.days. Gram positive bacteremia was determined in 13% of BSI patients. Of these isolates, 3 were Staphylococci. Gram negative bacteremia was determined in 35% of these isolates, 4 were *Acinetobacter baumannii*, 3 were Klebsiella pneumonia and 1 was Proteus mirabilis and in 56% of cases BSI was diagnoses clinically. A univariate analysis identified ventilator-associated pneumonia, sedation, and longer interval between onset of CLABSIs and the duration of catheterization as risk factors of CLABSIs. In multivariate analysis, the independent factors of CLABSI which are the duration of catheterization (OR, 1.06; 95% CI, [1.003–1.139]; p = 0.042) and catheterization attempt number (OR, 1.99; 95% CI, [1.18–3.37]; p = 0.01). Thirteen (56.5) patients developed septic shock and they all died.

**Discussion** The rate of CLABSI in our ICU (19.2/1000 catheter.days) was higher compared with the mean rate of CLABSI in ICU reported by the NNIS system surveillance for 2004, which is 3.9/1000 catheter.days [1]. Duration of catheterization, frequent manipulation of catheter, catheter location, catheter type, underlying diseases, suppression of immune system, and types of fluids administered through the catheter are significant risk factors in development of BSIs [2]. In our study both duration of catheterization and number of attempts are independent factors for CLABSI.

**Conclusion** In a monocenter cohort, CLABSI had a moderate density rate but are associated with poor outcome. Identifying the risk factors is necessary to find solutions for this major health problem.

**Competing interests** None.

**References**

1. National Nosocomial Infections Surveillance (NNIS) system report, data summary from January 1992 through June 2004, issued October 2004. Am J Infect Control 2004;32:470–85.
2. Öztürk F, Gündeş S, Işık C. Prospective evaluation of the risk factors, etiology and the antimicrobial susceptibilities of the isolates in nosocomial bacteriemic patients. Mikrobiyol Bul.2008;42:17–27.

#### P175 Is prehospital endobronchial intubation a risk factor for subsequent ventilator associated pneumonia?

##### Timothée Trampont^1^, Thomas Lafon^2^, Thomas Daix^3^, Vincent Legarçon^1^, Paul Claverie^1^, Henri Hani Karam^1^, Nicolas Pichon^3^, Philippe Vignon^3^, Bruno François^3^

###### ^1^Service urgences adultes/samu-smur, C.H.U de Limoges, Limoges, France; ^2^Services urgences adultes/samu-smur/inserm cic1435, C.H.U de Limoges, Limoges, France; ^3^Inserm cic 1435/réanimation polyvalente, C.H.U de Limoges, Limoges, France

####### **Correspondence:** Timothée Trampont - timothee.trampont@orange.fr

*Annals of Intensive Care* 2017, **7**(**Suppl 1**):P175

**Introduction** According to some studies, field-intubated patients have 1.5–3 times greater risk of ventilator associated pneumonia (VAP). Endobronchial intubation (EI) can be unrecognized by the physicians and may result in complications such as atelectasis which in turn could increase the risk of VAP. The aim of our study was to confirm this hypothesis.

**Patients and methods** This monocentric retrospective study included all consecutive patients >18 years who underwent an out-of-hospital tracheal intubation before their admission to the intensive care unit (ICU) between January 2012 and December 2015. Exclusion criteria were suspected aspiration or pneumonia on admission, patients who died within the first 5 days of ICU stay, extubation in less than 48 h and underlying disease making radiological interpretation difficult for VAP diagnosis. VAP were divided into early onset (<7 days) and late onset (≥7 days) events and were independently diagnosed by two experienced intensivists who had no access to the initial chest X-ray performed to check the position of the tracheal tube, based on the Clinical Pulmonary Infection Score. Onset of ventilator associated tracheobronchitis (VAT) was also noted. Inadvertent endobronchial intubation was determined by another independent physician based on the interpretation of admission chest X-ray.

**Fig. 6:**
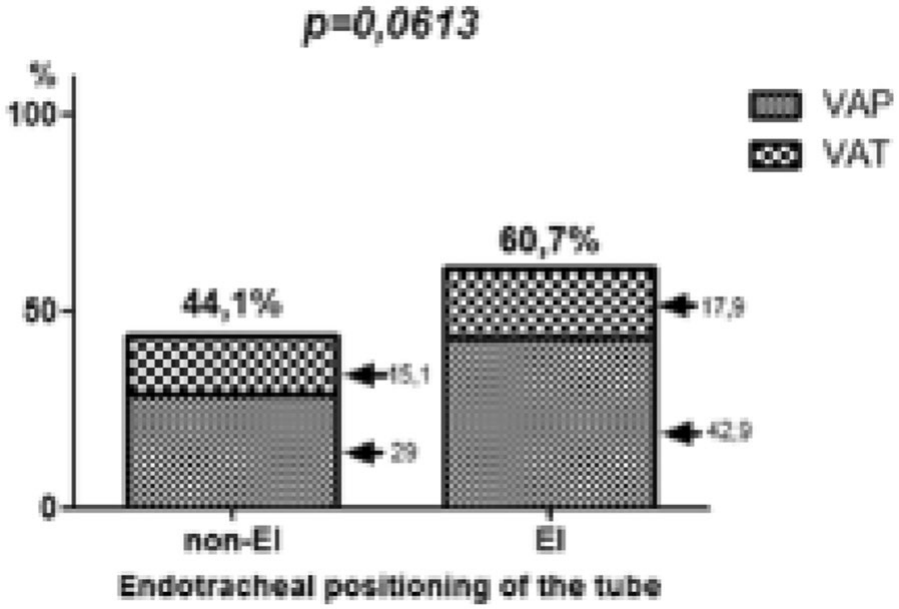
Rate of early-onset VAP + VAT depending of the position of the tube

**Results** 397 patients were intubated out-of-hospital. Of the 284 patients excluded, 104 had an extubation in less than 48 h, 114 were died within the first 5 days, 22 had a suspicion of pneumonia, 28 a suspicion of aspiration and 8 an underlying disease making radiological interpretation difficult. Of the 121 patients included, 28 (23.1%) had an EI upon admission. No significant difference was observed between the EI and non-EI group for gender, age, SAPS2, comorbidities and diagnostic category (cardiorespiratory arrest, trauma, coma and cardiorespiratory failure). Early-onset VAP were diagnosed in 43% in the EI group and in 29% of non-EI patients (p = 0.085). Adding early onset VAT, the respiratory infection rate was 61% in the EI group and 44% in the non-EI group (p = 0.061) (Fig. 6). Late-onset VAP were observed in 8.6% in the non-EI group and 7.1% in the EI group, without difference between groups (p = 0.403). There was no inter-group difference in the duration of ventilation, duration of ICU stay and ICU mortality. *Staphyloccocus aureus* was the most prevalent pathogen in patients with early-onset VAP (23.1%, only one strain was methicillin-resistant).

**Conclusion** This study found a high rate of inadvertent prehospital endobronchial intubation with a higher incidence of early-onset VAP. These results support the implementation of specific procedures to decrease the incidence of EI.

**Competing interests** None.

**References**

1. Bissinger U, Lenz G, Kuhn W. Unrecognized endobronchial intubation of emergency patients. Annals of Emergency Medicine. 1989;18(8):853–55.
2. Sitzwohl C, Langheinrich A, Schober A, et al. Endobronchial intubation detected by insertion depth of endotracheal tube, bilateral auscultation, or observation of chest movements: randomised trial. BMJ. 2010;341(nov091):c5943–c5943.

#### P176 Early versus late-onset ventilator-associated pneumonia: causative pathogens and resistance profiles

##### Hend Ben Lakhal^1^, Aymen M’rad^1^, Fatma Essafi^1^, Nasreddine Foudhaili^1^, Hafedh Thabet^2^, Youssef Blel^1^, Nozha Brahmi^1^

###### ^1^Department of intensive care and toxicology, Centre d’Assistance Médicale Urgente, Tunis, Tunisia; ^2^Service de réanimation, centre d’assistance médicale-urgente, Tunis, Tunisia

####### **Correspondence:** A M’rad - mrad.aymen@gmail.com

*Annals of Intensive Care* 2017, **7**(**Suppl 1**):P176

**Introduction** Ventilator-associated pneumonia (VAP) is associated with increased hospital stay and high morbidity and mortality in critically ill patients. The classic dichotomy between early and late onset VAP is no longer helpful available. The aims of this study were to determine the incidence of multidrug-resistant pathogens in the first episodes of VAP and to assess potential differences in bacterial profiles of subjects with early-onset versus late-onset VAP.

**Patients and methods** Retrospective cohort study over a period of 18 months including all patients who had a first episode of VAP confirmed by positive culture. Subjects were distributed into 2 groups according to the number of intubation days: early-onset VAP (<5 days) or late-onset VAP (≥5 days).The primary endpoint was the nature of causative pathogens and their resistance profiles.

**Results** Sixty patients were included, 29 men and 31 women. The average age was 38 ± 16 years. The IGS 2 at admission was 40.5 [32; 44] APACHE 19 [15; 22]. Monomicrobial infections were diagnosed in of 46 patients (77%).Two different bacteria were isolated in 14 cases (13%). *A. baumannii* was the most frequently isolated in 53% (n = 32) of patients; followed by *P. aeruginosa* in 37% (n = 22), *Enterobacteriaceae* in 28% (n = 17) and *S. aureus* in 5% (n = 3). The isolated bacteria were multidrug-resistant in most cases (58/60). The VAP group comprised 36 episodes (60%) of early-onset VAP and 24 episodes (40%) of late-onset VAP. *A. baumannii* was isolated in 47% of early VAP (n = 17) versus 62% of late VAP (n = 15) (p = NS), *P. aeruginosa* in 36% of early VAP (n = 13) versus 37% of late VAP (n = 9) (p = NS) and *Enterobacteriaceae* in 30% of early VAP (n = 11) versus 25% of late VAP (n = 6) (p = NS). For the resistance profile of the different pathogens isolated, there was no difference between early and late onset VAP.

**Conclusion** According to new data from the literature, there were no microbiological differences in the prevalence of potential multidrug-resistant pathogens or in their resistance profiles associated with early-onset versus late-onset VAP.

**Competing interests** None.

#### P177 Nosocomial infection in the sever burns

##### Rihi El Mehdi^1^

###### ^1^Intensive care unit, IBN ROCHD, Casablanca, Morocco

####### **Correspondence:** Rihi El Mehdi - mehdi_44@hotmail.fr

*Annals of Intensive Care* 2017, **7**(**Suppl 1**):P177

**Introduction** The bacterial nosocomial infection is a major cause of morbidity and mortality in burned. The bacterial ecology in an ICU has a major impact in terms of morbidity and mortality, particularly in the center of burned or length of stay of patients is increased compared to a general intensive care.

**Materials and methods** We conducted an observational study spread over 7 months in ICU for severe burned burnt including any who have spent more than 48 h with nosocomial infection (modified CDC criteria), and in which all biological and bacteriological samples were taken. The different types of infections studied were: skin, urinary, lung and bloodstream infections. They excluded all patients belatedly supported or having stayed in other healthcare facilities.

**Results** One hundred twenty (120) patients showed nosocomial infection during this period. The sex ratio (M/F) was 1.7 and the mean age was 39 ± 23 years. Bacteremia was present in 44.84% of cases, followed by the urinary tract infection that was present in 21.21% of cases, followed by the cutaneous infection in 10.30% of cases, and last pulmonary infection in 9% of cases. Infection was polymicrobial in 14.5% of cases. The main bacteria identified were: *Acinetobacter baumanii* (43.45%) of which 74% is resistant to imipenem, Enterobacteriaceae (31.5%), *Pseudomonas aeruginosa* (24%) of which 83.25% is resistant to ceftazidime and 68.2% is resistant to imipenem, Enterococcus (16%) and *Staphylococcus Aureus* (14.29%).

**Conclusion** The incidence of nosocomial infection is very high compared to literature. The rate of resistance to common antibiotics is very high. A drastic management of antibiotics in our context, the selection of patients and the frequent use in the operating room for skincare allow a better management of these patients.

**Competing interests** None.

**Reference**

1. Wurtz R, Karajovic M, Dacumos E, Jovanovic B, Hanumadass M. Nosocomial infections in a burn intensive care unit. Burns. 1995;21:181–84.

#### P178 Ventilator acquired pneumonia: diagnosis treatment and bacterial ecology in a Moroccan intensive care unit

##### Hanane Ezzouine^1^, Mahmoud Kerrous^1^, Saad El Haoui^1^, Soufiane Ahdil^1^, Abdellatif Benslama^1^

###### ^1^Anesthesiology and intensive care department, University Teaching Hospital IBN Rushd-Casablanca, Casablanca, Morocco

####### **Correspondence:** Hanane Ezzouine - ezzouinehanane@yahoo.fr

*Annals of Intensive Care* 2017, **7**(**Suppl 1**):P178

**Introduction** The management of ventilator acquired pneumonia is a diagnostic and therapeutic challenge. Antibiotic therapy is a key link. The objective of this work is to study the epidemiological, clinical patients who developed VAP during their stay in the Medical Intensive Care Unit of the CHU Ibn Rushd in Casablanca and are features of the bacterial ecology of VAP during the 2015.

**Patients and methods** It is a retrospective descriptive study and analytical spread over 1 year, from January 2015 to December 2015. Were included all patients with pneumonia Ventilator, with or without bacteraemia, after hospitalization for more than 48 h. Were collected clinical, biological, radiological, bacteriological and scalable patients included.

**Results** The average age of the patients was 42.19 years with a sex ratio (M/F) 1.47 APACHE II score average was 16.4, the average SAPSII was 31.12 and the average was 2.39 SOFA. 86.5% of patients were intubated on admission in intensive care. 3.5% were intubated within less than 5 days notice after admission and the average time to onset of VAP was 3.76 days. The VAP were early in 68.4% of cases. The average hospital stay of patients being 19.63 days. 73.68% in the sample used in our patients was bronchial aspiration. The most offending germs are 27.2% *Acinetobacter baumannii*, *Pseudomonas aeruginosa* 16.3%. 100% of patients received empirical antibiotic therapy chosen according to the ecology of the service. 77.4% died. 43.8% of deaths were directly related to VAP.

**Conclusion** Ventilator acquired pneumonia is main problem in our ICU. The bacteriological ecology must be usually known. In our unit *Acinetobacter baumanii* is the main germ associated.

**Competing interests** None.

#### P179 Determinants and prognostic factors of *Acinetobacter baumannii* ventilator-associated pneumonia

##### Khalid Abidi^1^, Tarek Dendane^1^, Ssouni Oussama^1^, Jihane Belayachi^2^, Naoufal Madani^2^, Redouane Abouqal^2^, Amine Ali Zeggwagh^1^

###### ^1^Medical intensive care unit, Mohamed V University Hopital Ibn Sina, Rabat, Morocco; ^2^Service des urgences médicales hospitalières - ibn sina – université mohamed v – rabat, Hopital Ibn Sina, Rabat, Morocco

####### **Correspondence:** Tarek Dendane - tdendane@hotmail.com

*Annals of Intensive Care* 2017, **7**(**Suppl 1**):P179

**Introduction** *Acinetobacter baumannii* (AB) ventilator-associated pneumonia (VAP) is common in critically ill patients. The aims of this study were to describing the epidemiological characteristics of AB-VAP, to identify risk factors for acquisition and factors predictive of a poor outcome.

**Materials and methods** A retrospective-prospective study was conducted at the Medical Intensive Care Unit of the University Hospital Ibn Sina, Rabat-Morocco from January 2013 to December 2015. They were included in the study that all patients developed VAP with identified germ. For identification of risk factors of acquisition of AB VAP, two groups of patients were compared: patients with AB VAP versus patients with VAP caused by other germs. To identify factors associated with mortality, two other groups were compared: Survivors versus died.

**Results** 122 patients presented VAP among which 60 were caused by *Acinetobacter baumannii.* Among isolates of AB, 8.3% were drug susceptible, and 16.7% were multidrug-resistant while 75% were extensively drug-resistant. They were Independent risk factors for acquisition of AB VAP in multivariate analysis: the presence of a central venous catheter before the occurrence of VAP, duration of prior hospitalization ≥4 days and ICU duration of stay ≥5 days. The mortality rate of AB VAP was 85%. The independent risk factors for poor outcome in multivariate analysis were: duration of antibiotic treatment >7 days, the reintubation and the presence of a previous hospitalization.

**Discussion** Our data were similar to those of the literature with a high incidence of VAP due to the AB (49%) and a high rate of resistance to this bacterium particularly to carbapenems. However, and compared to the literature, the VAP AB were responsible for a death rate much higher (85%).

**Conclusion** Our data were similar to those of the literature with a high incidence of VAP due to the AB (49%) and a high rate of resistance to this bacterium particularly to carbapenems. However, and compared to the literature, the VAP AB were responsible for a death rate much higher (85%).

**Competing interests** None.

#### P180 Ventilator-associated pneumonia in the elderly: a study of the prognosis

##### Hatem Ghadhoune^1^, Anis Chaari^2^, Guissouma Jihene^1^, Hend Allouche^1^, Insaf Trabelsi^1^, Habib Brahmi^1^, Mohamed Samet^1^, Hatem El Ghord^1^

###### ^1^Réanimation médicale bizerte, Faculté de médecine de Tunis, Bizerte, Tunisia; ^2^Intensive care unit, King Hamad University Hospital, Muharraq, Bahrain

####### **Correspondence:** Hatem Ghadhoune - ghadhoune@yahoo.fr

*Annals of Intensive Care* 2017, **7**(**Suppl 1**):P180

**Introduction** Ventilator-associated pneumonia (VAP) is common in critically-ill patients. In fact, 10–20% of patients requiring invasive mechanical ventilation develop this complication. The onset of VAP has been reported to be associated with increased mortality. However, data related to critically-ill elderly patients are scarce. The aim of this study is to assess the prognostic impact of VAP in critically-ill elderly patients.

**Patients and methods** Mono-center, retrospective study conducted from 01/012014 to 30/09/2015. All old patients (age ≥65 years) requiring mechanical ventilation were included. Two groups were compared: Patients who developed VAP (VAP (+) group) and those who did not develop VAP (VAP (−) group).

**Results** During the study period, 86 patients were included. The causes of admission in the intensive care unit (ICU) were shock (n = 30), acute respiratory failure (n = 41) and disturbed level of consciousness (n = 15). Diabetes mellitus, hypertension and chronic obstructive pulmonary disease were the most common comorbidities (44.2, 32.6 and 40.7% respectively). Mean age was 73.3 ± 6.5 years. Sex-ratio (M/F) was 1.8. Mean APACHE(II) score was 24 ± 9. The mean duration of mechanical ventilation was 10 ± 15 days. Thirty patients (34.9%) developed VAP. ICU-mortality was significantly higher in the VAP (+) group (90 vs 55.4%; p = 0.001). Multivariate analysis identified two independent factors predicting ICU mortality: Shock on admission (OR = 22.7, CI 95% [5.1–101.2], p < 0.001) and VAP (OR = 5.7, CI 95% [1.2–28.1], p = 0.033).

**Conclusion** VAP is common in critically-ill elderly patients and is associated with worse outcome. Therefore, preventing its onset is of paramount importance.

**Competing interests** None.

**Reference**

1. Magill SS, Li Q, Gross C, Dudeck M, Allen-Bridson K, Edwards JR. Crit Care Med. 2016 Aug 10. [Epub ahead of print].

#### P181 Epidemiologic characterization and prognosis factors of *Acinetobacter baumannii* ventilator-associated pneumonia

##### Ben Sik Ali Habiba^1^, Nouira Hajer^2^, Najla Tilouch^2^, Sondes Yaakoubi^2^, Oussama Jaoued^2^, Rim Gharbi^2^, Mohamed Fekih Hassen^2^, Souheil Elatrous^2^

###### ^1^Réanimation médicale, EP taher sfar, Mahdia, Tunisia; ^2^Réanimation médicale, EPS Taher Sfar Mahdia, Mahdia, Tunisia

####### **Correspondence:** Mohamed Fekih Hassen - mohamed.fekihhassen@rns.tn

*Annals of Intensive Care* 2017, **7**(**Suppl 1**):P181

**Introduction** Ventilator associated pneumonia (VAP) is the most frequent nosocomial infection in critically ill patients. It is associated with high mortality, prolonged mechanical ventilation, length of stay, and increased health-care costs. Among pathogens responsible of VAP, *Acinetobacter baumannii* which is characterized by its ability to spread in the hospital environment and to acquire resistance leading sometimes to therapeutic impasses is associated with a particularly high mortality reaching 30–75%.

**Objective** To describe the epidemiological characteristics of *A. baumannii* VAP, to determine their prognosis and identify factors associated with mortality.

**Patients and methods** It is a monocentric observational study conducted over a period of 13 years in a Tunisian intensive care unit (ICU) including mechanical ventilated patients for more than 48 h with confirmed *A. baumannii* VAP.

**Results** One hundred and twenty-three patients were included in the study*. A. baumannii* was responsible for 31% of VAP in our ICU. The VAP were late in 59% of cases. More than 90% of isolates pathogens were resistant to ticarcillin, piperacillin, piperacillin–tazobactam, ceftazidime and ciprofloxacin. Sixty percent of germs were sensitive to imipenem. Resistance to imipenem has increased consistently from 0% at the beginning of the study to 88% in 2015. All pathogens were susceptible to colistin. *A. baumannii VAP* was complicated by septic shock in 63% of cases. The median duration of mechanical ventilation and of ICU stay were 17 (IQR: 11–25) and 25 days (IQR: 17–41) respectively. The use of parenteral nutrition was the only factor associated with the occurrence of *A. baumannii* VAP resistant to imipenem (odds ratio 2.27, 95% CI [1.07–4.80], p = 0.033). ICU mortality was 45%. It was higher in patients with *A. baumannii* VAP resistant to imipenem (55 vs 39%, p > 0.05). In the multivariate analysis, the age, the use of renal replacement therapy and the occurrence of VAP relapse have been identified as factors associated with mortality.

**Conclusion** *A. baumannii* resistance to imipenem became threatening. The use of parenteral nutrition was the only factor associated with the occurrence of *A. baumannii* VAP resistant to imipenem. The choice of empiric antimicrobial for VAP caused by this pathogen must take in consideration the epidemiologic data of each country and each ICU. *A. baumannii* VAP was associated with high mortality. The age, the use of renal replacement therapy and the occurrence of VAP relapse have been identified as predictive of poor outcome.

**Competing interests** None.

#### P182 Admission in intensive care unit for severe adverse drug event: what finding?

##### Julien Arcizet^1^, Bertrand Leroy^1^, Caroline Abdulmalack^2^, Catherine Renzullo^1^, Maël Hamet^2^, Jean-Marc Doise^2^, Jérôme Coutet^1^

###### ^1^Pharmacy unit, C.H. Chalon sur Saône William Morey, Chalon-sur-Saône, France; ^2^Intensive care unit, C.H. Chalon sur Saône William Morey, Chalon-sur-Saône, France

####### **Correspondence:** Julien Arcizet - julien.arcizet@ch-chalon71.fr

*Annals of Intensive Care* 2017, **7**(**Suppl 1**):P182

**Introduction** Adverse drug events (ADE) remain a serious public health problem. They represent between 0.16 and 15.7% of hospital admissions and between 0.37 and 27.4% of intensive care unit (ICU) admissions. They are defined as any injury related to a drug, and include both adverse drug reactions, expected or not, but also underuse, overuse and misuse, unintended or undesired, preventable or not. Indeed, mortality from iatrogenic event would rise between 2.0 and 28.1%, whereas these ADE that resulted in ICU hospitalization could be prevented in 17.5–85.7% of cases. These unplanned admissions overload ICU, limit access to health care for other patients and have serious economic consequences for the health system. It is therefore necessary to study these ADE to know their main causes and attempt to find a solution to avoid them.

The main objectives of our study were to clinically and pharmaceutically analyze and stratify the different ADE leading to hospitalization in our ICU.

**Patients and methods** This is a monocentric prospective study, between June 2014 to January 2016, in medico-surgery ICU. From all admissions, we had included patients admitted in our hospital for involuntary ADE (plausible, likely and very likely causal). We had collected clinical aspects (Failure mode, IGSII score, mortality in ICU) and pharmaceutical aspect (number of drug, offending drugs) at daily medical staff meeting.

**Results** On 1545 admissions, 154 patients were hospitalized for unintended ADE. The average age was 70 years old [26; 95], with a men/women ratio equal to 1.8. The clinical severity IGSII score found was 51 [13; 120]. Average length of stay in ICU was 5.5 days [1; 28] on average in this unit. The main reasons of admission were: hematologic failure (in particular hemorrhagic) (29.9%), metabolic failure (19.5%), renal failure (11.0%), neurological failure (11.0%) and sepsis (10.4%). Respiratory, hepatic, hemodynamic failures and hypersensitivity reactions represented respectively less than 10% of cases. 34 patients (22%) included died during their stays in ICU. On average, 7.6 drugs were found in the usual treatment of the patient. 32.5% of this population had a known cognitive disorder and 62% of them self-management of their treatment. The main drugs involved were: furosemide (16.9%), metformin (13.0%), perindopril (9.1%), lysine acetylsalicylate (8.4%), warfarin (8.4%) and fluindione (7.8%). The most common drug families involved were: drugs of the cardiovascular system (33.8% of cases), anticoagulants and antiplatelet agents (31.8%), antidiabetics (16.9%) and psychotropic (13.6%).

**Conclusion** Hospitalizations in ICU for ADE are still too common despite their preventability for most cases. Many patients with known cognitive disorder manage their treatment themselves and this is probably one of the reasons of iatrogenic events. Anticoagulants and antiplatelet agents, by side effects, misuse, underuse or overuse are very often involved. The onset of kidney failure from dehydration and the continuation of nephrotoxic and antidiabetic treatment also remain one of the most common causes. Consequently, it is necessary to continue and develop primary, secondary and tertiary prevention strategies to prevent their appearance, to limit their consequences and to reduce recidivism.

**Competing interests** None.

#### P183 Prolonged intensive care unit stay: prognostic factors

##### Chaigar Mohammed Cheikh^1^, Zakaria Quechar^1^, Hanane Ezzouine^1^, Abdellatif Benslama^1^

###### ^1^Anesthesiology and intensive care department, UNIVERSITY TEACHING HOSPITAL IBN RUSHD-CASABLANCA, Casablanca, Morocco

####### **Correspondence:** Chaigar Mohammed Cheikh - chaigarmed@gmail.com

*Annals of Intensive Care* 2017, **7**(**Suppl 1**):P183

**Introduction** Intensive care unit (ICU) is usually identified as a place of acute care, concentrated over a short period. For many reasons, a prolonged stay in the ICU has a pejorative connotation for the intensivist physician. The aim of our study is to describe the epidemiological, clinical, paraclinical profile of patients hospitalized for a long time in ICU (over 15 days) and to identify the main prognostic factors and those that can predict the duration of stay in ICU.

**Patients and methods** We conducted a retrospective study, over a period of 5 years and 6 months (January 2010 to June 2015), enrolling patients whose length of stay was greater than or equal to 15 days in medical ICU of the UH Ibn Rochd of Casablanca. Statistical analysis was performed using SPSS 21.0.

**Results** We enrolled 151 patients witch correspond to 8.2% of all admissions. The sex ratio was 1.6, the average age was (43.2 ± 18.5 years). The majority of patients (75%) were transferred from the emergency department. Medical pathology was the main motive of admission (82.1% of cases). Means of severity scores were calculated as follows: APACHE III (56.2 ± 22.9), IGS III (40 ± 9.6), OSF (0.6 ± 0.7). The average length of stay was (42.2 ± 59.7 days). The incidence of nosocomial infection was 82.8%, the average day of onset was 9.34 ± 8.7 days. Pneumonia, bacteremia and vascular catheter infections were the main sites, Gram-Negative Bacilli were the most frequently identified, dominated by *Acinetobacter baumanii* (27.9%). Mechanical ventilation (91.4%) and vascular catheterization (84.1%) are the most used invasive devices. Antibiotics (92.1%), sedation (91.4%) and vasopressors (60.9%) were the main administrated treatments. The outcome was favorable in 37.7% of cases. Hemodynamic instability (64.2%) and respiratory complications (61.6%) were the complications most frequently observed, septic shock occurred in 49% of cases. Mortality rate was 55%. In univariate analysis, the variables that have emerged as risk factors of mortality were: sex, length of hospitalization, severity scores (APACHE III, IGS III, OSF), the Charlson comorbidity score adjusted to age, traumatic pathology, the occurrence of nosocomial infection, septic shock, hemodynamic instability, neurological worsening, use of vasopressors, and tracheostomy. In multivariate analysis: nosocomial infection (p = 0.04), hemodynamic worsening (p = 0.03), use of vasopressors (p < 0.01) and antibiotics (p < 0.01) appeared to be risk factors of mortality.

**Conclusion** Although patients hospitalized in ICU for more than 15 days are few, they represent a serious problem of care and an important part of the activity of intensive care (bed occupancy, care costs).

**Competing interests** None.

#### P184 Admissions and readmissions to the intensive care unit of patients with hematologic malignancies: a 5 years retrospective study

##### Magalie Joris^1^, Dimitri Titeca Beauport^1^, Loay Kontar^1^, Delphine Lebon^2^, Bérengère Gruson^2^, Michel Slama^1^, Jean-Pierre Marolleau^2^, Julien Maizel^1^

###### ^1^Réanimation médicale, Centre Hospitalier Universitaire, Amiens, France; ^2^Hématologie clinique et thérapie cellulaire, Centre Hospitalier Universitaire, Amiens, France

####### **Correspondence:** Magalie Joris - joris.magalie@chu-amiens.fr

*Annals of Intensive Care* 2017, **7**(**Suppl 1**):P184

**Introduction** Despite an improvement in prognosis of patients with hematologic malignancies for the last decade, mortality of such patients admitted to the intensive care unit (ICU) remains high. Yet, it seems that a first ICU stay does not modify prognosis of the malignancy. Until now, there is no data on readmission in the ICU of such patients and its effect on short and long term prognosis impact.

**Patients and methods** This retrospective, single-center study conducted on a 5 years period in the medical ICU from our university hospital included 265 patients with hematological malignancies admitted for a first stay. Objectives were to evaluate the ICU, day 28 and 6 months mortality, to identify prognostic factors associated with mortality within uni- and multivariate analysis, to evaluate readmission rate within the 60 days after discharge, to indentify the admission risk factors associated with ICU readmission and the prognosis factors associated with mortality during the second ICU stay.

**Results** The mean age was 58.6 ± 14.8 years, with a male–female ratio of 1.55. The most represented malignancies were acute leukemia (40.8%) and non-Hodgkin lymphomas (26.8%); 16.2% were hematopoietic stem cell transplant recipients. 54% of patients had newly diagnosed malignancy, 20.8% were in complete remission (CR), 11.7% had stable disease or partial remission and 13.6% had progressive disease. 46.4% of patients presented with severe neutropenia at the time of ICU admission. The main life-threatening complications precipitating ICU admission were acute respiratory failure for 43.8%, sepsis for 51.7%, acute kidney injury for 14%, neurological disturbance for 18.1% and preventing tumor lysis syndrome for 15.8%. 11.3% presented with hemophagocytic lymphohistiocytosis (HLH). 34.3% of patients received non-invasive ventilation, 46.8% mechanical ventilation (MV), 54.3% needed vasoactive drugs administration and 40.8% had renal replacement therapy. ICU, day 28 and 6 months mortality were 39.8, 46.4 and 63.4% respectively. By multivariate analysis poor performance status, IGS II, HLH, MV and anti-fungal administration were associated with increased ICU mortality, infections with Pseudomonas were associated with higher day 28 mortality. Catheter related infections were associated with better ICU survival and CR was associated with lower day 28 mortality. 38 of 132 (28.9%) candidate patients for ICU readmission after a first stay were readmitted within the 60 days following discharge. Median overall survival was lower in readmitted versus non readmitted patients. 6 months mortality was 73.8% for readmitted versus 13.8% for no readmitted patients (p < 0.0001). The second ICU stay mortality was 60.5% and 6 month mortality was 78.9%. By multivariate analysis, only MV was associated with prognosis. The 6 months mortality rate of patients who survived to the second ICU stay was significantly higher than the patients who survived to the first admission but were not readmitted (46.7 vs 13.8%, p = 0.0007).

**Conclusion** Main features, short and long term mortality and prognostic factors associated with ICU admission are in lines with previous studies. Early readmission rate was high with a negative impact on survival. Despite admission in the ICU of patients with hematologic malignancies seems not to affect long term prognosis, early readmission seems to have a pejorative impact on the course of the malignancy.

**Competing interests** None.

**Reference**

1. Azoulay E. Outcomes of critically ill patients with hematologic malignancies: prospective multicenter data from France and Belgium—a groupe de recherche respiratoire en réanimation onco-hématologique study. J Clin Oncol. 2013;31:2810–18.

#### P185 Prognosis of lung cancer patients admitted to ICU

##### Julie Gorham^1^, Lieveke Ameye,^2^, Thierry Berghmans,^1^, Marianne Paesmans^2^, Jean-Paul Sculier,^1^, Anne-Pascale Meert^1^

###### ^1^Intensive Care and Thoracic Oncology, Institute Jules Bordet, Brussel, Belgium; ^2^Data centre, Institute Jules Bordet, Brussel, Belgium

####### **Correspondence:** Anne-Pascale Meert - secret.sculier@bordet.be

*Annals of Intensive Care* 2017, **7**(**Suppl 1**):P185

**Introduction** Lung cancer is among all types of cancer, the most common solid tumour admitted in intensive care [1]. Recent studies showed that the prognosis of patients with lung cancer during intensive care unit (ICU) stay has improved [2]. The aim of our study was to determine the causes of ICU admission of lung cancer patients, their prognosis and to identify factors predicting hospital mortality and survival after hospital discharge.

**Patients and methods** We conducted a retrospective study including all patients with lung cancer admitted for a medical or surgical complication in the intensive care unit of a cancer hospital between September 1, 2008 and December 31, 2013. mbol’ > [1]. Recent studies showed that the prognosis of patients with lung cancer during intensive care unit (ICU) stay has improved [2]. The aim of our study was to determine the causes of ICU admission of lung cancer patients, their prognosis and to identify factors predicting hospital mortality and survival after hospital discharge.

**Results** During this period, 212 ICU admissions occurred in 180 patients with lung cancer. The majority of them were men (64%), had non small cell lung cancer (80%) and metastases at the time of admission (81%). 54% received an antineoplastic therapy during the month preceding the ICU admission. 47 patients (26%) died during hospitalisation with 36 deaths in the intensive care unit and 11 in the hospital ward after ICU discharge. The three main reasons of admissions were: cardiovascular problems (32%), respiratory failure (29%) and neurological (16%) complications. The SAPS II score (OR 1.07; 95% CI 1.04–1.11) as continuous covariate and the presence of respiratory complications (OR 4.00; 95% CI 1.76–9.07) were the 2 factors independently affecting hospital mortality in multivariate analysis. Median overall survival since ICU admission was 3.7 months (95% CI, 2.8–4.4). Median overall survival for patients discharged alive from ICU was 4.8 months (95% CI, 3.9–5.6 months). Considering patients discharged alive from the hospital, only the presence of metastases was a statistically independent prognostic factor in multivariate analysis (HR 2.3; 95% CI 1.44–3.65).

**Discussion** The prognosis of patients with lung cancer admitted in ICU improved probably due to a better selection of these patients eligible for intensive care. As already observed in general cancer patients’ population, prognosis factors for hospital mortality are related to the acute complications but survival after hospital discharge is dependent from the cancer stage.

**Conclusion** Lung cancer patients are admitted in critical care in over half of the cases for cardiovascular and respiratory complications. The hospital mortality rate of these patients admitted in ICU was 26%. However survival after hospital discharge remains low and dependent of the cancer metastatic status. ICU admission should be considered for patients with lung cancer.

**Competing interests** None.

**References**

1. Kress JP, Christenson J, Pohlman AS et al. Outcomes of critically ill cancer patients in a university hospital setting. Am J Respir Crit Care Med. 1999;160:1957–61.
2. Adam AK, Soubani AO. Outcome and prognostic factors of lung cancer patients admitted to the medical intensive care unit. Eur Respir J 2008;31:47–53.

#### P186 Treatment intensity may not predict prognosis for patients admitted in ICU with relapsed acute myeloid leukemia

##### Max Guillot^1^, Marie-Pierre Ledoux^2^, Thierry Braun^1^, Quentin Maestraggi^1^, Baptiste Michard^1^, Vincent Castelain^1^, Raoul Herbrecht^2^, Francis Schneider^1^

###### ^1^Réanimation médicale, C.H.R.U. Hôpitaux Universitaires Strasbourg, Strasbourg, France; ^2^Département d’oncologie et d’hématologie, C.H.R.U. Hôpitaux Universitaires Strasbourg, Strasbourg, France

####### **Correspondence:** Max Guillot - max.guillot@me.com

*Annals of Intensive Care* 2017, **7**(**Suppl 1**):P186

**Introduction** Admission of cancer patients with poor prognosis in intensive care units (ICU), like acute myeloid leukemia (AML) resistant to the first course of induction chemotherapy, continue to be controversial. The ICU trial may be an alternative in this case to ICU refusal. The ICU trial is a full-code ICU admission followed by reappraisal of the level of care. The objective of this study was to find variables available at ICU admission and at day 3 in order to predict prognosis of critically ill medical patients with relapsed acute myeloid leukemia.

**Patients and methods** Retrospective monocentric study of consecutive patients with a relapsed AML admitted to the 30 beds medical ICU of an academic hospital. We evaluated hematological treatments, organs supports and mortality in ICU.

**Results** Between 2002 and 2014, 24 patients with relapsed AML were admitted in the ICU. At admission, patients were 54 years old, IGS 2: 64 ± 24, Lactates: 4.9 mmol/L (±4.7). Eight patients underwent bone marrow transplant (BMT). Five patients had graft-versus-host disease (GVHD). 12 patients were admitted for septic shock, 7 patients for acute respiratory failure, 2 for cardiac arrests, 1 for coma, 1 for acute kidney injury and 1 for hemorrhagic shock. BMT was significantly associated with higher mortality [Odds ratio (0R) 13.0 (95% confidence interval (95% CI) 1.7–99, 43)—p: 0.02]. 7 BMT patients died in ICU. Neutropenia [OR 0.33 (95% CI 0.05–1.87)—p: 0.4] and GVHD (OR 2.0 [95% CI 0.07–51.6)—p: 1.0] were not able to predict mortality in ICU. The first day in ICU: 15 patients were under mechanical ventilation, 17 patients need vasopressor perfusion and 3 patients dialysis. Mortality in ICU was 37%. 4 patients died from acute illness before day 3. Among the 24 patients admitted in ICU, none of the life-sustaining interventions at admission were associated with mortality: invasive mechanical ventilation [OR 9.14 (95% CI 0.9–92.4) - p: 0.08], vasopressor perfusion [OR 6.2 (95% CI 0.6–62.2)—p: 0.18] and renal replacement therapy [OR 4.8 (95% CI 0.3–65.8)—p: 0.27]. On day 3, life supports were not associated with higher mortality: invasive mechanical ventilation [OR 3.7 (95% CI 0.32–41.1)—p: 0.35], vasopressor perfusion [OR 2.0 (95% CI 0.27–14.7)—p: 0.64] and renal replacement therapy [OR 1.2 (95% CI 0.08–16.45)—p: 1.0].

**Conclusion** Mortality in ICU was 37% in patients with relapsed AML. In fact, temporary full-code ICU management in patients with relapsed AML seems to be appropriate. None of the life-sustaining interventions at admission and on day 3 were able to predict survival. An ICU trial of 3 days might not be enough to appraise precisely the outcome. Bone marrow transplant was associated with a high mortality in our study. In case of relapsed AML with BMT, ICU management is still challenging.

**Competing interests** None.

**Reference**

1. Azoulay E, Soares M, Darmon M, Benoit D, Pastores S, Afessa B. Intensive care of the cancer patient: recent achievements and remaining challenges. Ann Intensive Care. 2011;1(1):5.

#### P187 Mortality analysis of the chronically critically ill patients: an epidemiological prospective study

##### Severine Couffin^1^, David Lobo^2^, Nicolas de Prost^3^, Nicolas Mongardon^4^, Gilles Dhonneur^5^, Roman Mounier^2^

###### ^1^Surgical intensive care, Hospital Henri Mondor, Créteil, France; ^2^Anesthesia and surgical intensive care, Hospital Henri Mondor, Créteil, France; ^3^Réanimation Médicale, Hôpital Henri Mondor, Créteil, France; ^4^Service de réanimation médicale, Hôpital Cochin, Paris, France; ^5^Anesthesia and intensive care medicine, CHU Henri Mondor, Créteil, France

####### **Correspondence:** Severine Couffin - scouffin@gmail.com

*Annals of Intensive Care* 2017, **7**(**Suppl 1**):P187

**Introduction** The growing population of chronically critically-ill patients has a poor prognosis despite all the resources mobilised [1]. Our primary objective was to analyse the prognostic value of different definitions used to describe them. Our secondary objective was to look for early clinical and biological factors that could be associated with the in-hospital mortality.

**Patients and methods** We conducted an epidemiological prospective study in 3 intensive care units (neurosurgical, cardiosurgical and medical) of a large French teaching hospital (Henri Mondor, Créteil). We included all the patients hospitalized for at least 7 days. We tested 5 definitions: the prolonged mechanical ventilation, the definition taken up by Kahn et al. [2], the prolonged length of stay, the persistent critical illness and the persistent inflammation-immunosuppression and catabolism syndrome. Two biological examinations were performed: upon entering the study and 1 week later. The study endpoint was the in-hospital mortality.

**Results** Thirty patients were included between April and July 2016. Among them, only 40% matched the definition of prolonged mechanical ventilation, which is still the most used in the literature. Further, it was not associated with the mortality, but the prolonged length of stay was, with 59% of these patients, that did not survive to their hospital stay. Other parameters that were significantly different between the patients who died and those who survived were an advanced age, an elevated IGS II score at hospital admission, an elevated SOFA score at study entry, a late healthcare-associated infection and several biological variables: a high C reactive protein, low albumin and prealbumin and a poor percent of monocytes expressing HLA-DR, all measured at day 7.

**Conclusion** The in-hospital mortality of chronically critically-ill is still high. A prolonged length of stay is the only definition who may be helpful to identify the patients with the poorest outcome. Among the early factors associated with mortality, we found a late healthcare-associated infection and a low percent of monocytes expressing HLA-DR, pointing to the value of studying the immune system of these patients.

**Competing interests** None.

**References**

1. Nelson JE. Chronic critical illness. Am J Respir Crit Care Med. 2010; 182(4):446–54.
2. Kahn JM. The epidemiology of chronic critical illness in the United States. Crit Care Med. 2015;43(2):282–7.

#### P188 Clinical characteristics and outcome of nonagenarians and centenarians in a medical ICU

##### Pierrick Le Borgne^1^, Sophie Couraud^1^, Jean-Etienne Herbrecht^2^, Quentin Maestraggi^2^, Alexandra Boivin^2^, François Lefebvre^3^, Pascal Bilbault^1^, Francis Schneider^2^

###### ^1^Service d’accueil des urgences, Hôpitaux Universitaires de Strasbourg, Strasbourg, France; ^2^Réanimation médicale, C.H.R.U. Hôpitaux Universitaires Strasbourg, Strasbourg, France; ^3^Département d’information médicale, C.H.R.U. Hôpitaux Universitaires Strasbourg, Strasbourg, France

####### **Correspondence:** Pierrick Le Borgne - pierrick_med@yahoo.fr

*Annals of Intensive Care* 2017, **7**(**Suppl 1**):P188

**Introduction** As a result of demographic transition, the proportion of «very elderly» (≥90 years) patients is increasing worldwide and more of these patients are nowadays admitted to intensive care units (ICU). Among physicians the discussion about appropriateness of these ICU admissions still remains controversial mostly due to questionable outcome, limited resources and costs. The aim of the study was to determine and evaluate the clinical characteristics and outcome in a very old population admitted to a medical ICU in an urban teaching hospital.

**Patients and methods** We present here a monocentric, retrospective and observational study. We reviewed the charts of all patients (≥90 years) admitted to a medical ICU between 2000 and 2015 (16 years). We collected epidemiological, clinical and biological parameters and all therapeutic measures during the ICU stay. A long-term survival follow-up was also performed. Two hundred eighty-four patients were included for statistical analysis. Multivariate Cox regression was also performed to identify risk factors for 28-day outcome.

**Results** A total of 284 patients were included, which represented 1.8% of admissions to the ICU during the period of the study. The mean age was 92.6 ± 2.1 years, the sex ratio was 0.41. Most of patients (41%) were admitted from the Emergency Department. 20% of these admitted patients suffered of previous dementia. The mean Charlson comorbidity score was 7.7 ± 1.7 and the mean McCabe score was 1.33 ± 0.5. The admission diagnosis in the ICU was mainly respiratory distress (51%), septic shock (11%), cardiac arrest (10%) and coma (8%). The mean SAPS-II score within 24 h of ICU admission was 55.9 ± 21.7. Half of these patients required support by mechanical ventilation (mean duration 7.3 days) and vasoactive drugs and 6% of patients received renal replacement. ICU and in-hospital mortality rates were 38 and 44% respectively. Overall survival at 6 months after hospital discharge was 33%. Multivariate regression revealed necessity of catecholamines and mechanical ventilation as independent risk factors and urinary sepsis as protective factor for 28-day outcome. *In fine*, for 34% of these patients, a limitation of active treatment was decided (on average after 2 days of stay). For all others there was no justification for limiting care because of a well-established treatment plan (with family, GP, ICU team).

**Conclusion** The proportion of elderly patients remains low, but they are increasingly being treated in intensive care units. Nevertheless, the in-hospital mortality is high compared to the average mortality in our ICU over the same period (20%). The prognosis is often not as poor as initially perceived by physicians. The indication for ICU treatment in our study was mostly justified; in the setting of consistent patient care and good clinical practice. It remains therefore appropriate to discuss every single ICU admission of elderly patients without any restriction related to age. Thus, the ongoing cluster-randomized trial of ICU admissions for the elderly patients (ICE-CUB 2 study) is deeply awaited to confirm or not these results [1].

**Keywords** Intensive care; prognosis; outcome; elderly patients; over 90-years old.

**Competing interests** None.

**Reference**

1. Boumendil A, Woimant M, Quenot J-P, Rooryck F-X, Makhlouf F, Yordanov Y, et al. Designing and conducting a cluster-randomized trial of ICU admission for the elderly patients: the ICE-CUB 2 study. Ann Intensive Care. 2016;6(1):74.

#### P189 The hemorrhage postpartum: inventory

##### Setti-Aouicha Zelmat^1^, Djamila-Djahida Batouche^2^, Fatima Mazour^3^, Belkacem Chaffi^4^, Nadia Benatta^5^

###### ^1^Réanimation, etablissement hospitalier spécialisé 1er novembre, oran, Algeria; ^2^Réanimation pédiatrique, Centre Hospitalier et Universitaire d’Oran, Oran, Algeria; ^3^ Anesthesie -réanimation chirurgicale, EHS 1er Novembre, oran, Algeria; ^4^Service de gynéco-obstétrique, EHS 1er Novembre, oran, Algeria; ^5^Cardiologie, Centre Hospitalier et Universitaire d’Oran, Oran, Algeria

####### **Correspondence:** Setti-Aouicha Zelmat - settiaouichazelmat@yahoo.fr

*Annals of Intensive Care* 2017, **7**(**Suppl 1**):P189

**Introduction** Regardless of the route of delivery, the postpartum hemorrhage (PPH) is defined as blood loss ≥500 ml after childbirth, and severe PPH as blood loss ≥1000 ml. PPH is the leading cause of maternal mortality in Africa. The aim of this prospective study was to assess the quality of the initial management of PPH in Algeria in Oran EHU and to determine the factors of care with the severity of this complication.

**Patients and methods** We conducted a prospective cohort study between April 2014 and September 2014 at the EHU ORAN. All women who delivered vaginally and showed HPP including the suspected cause was uterine atony were included. The severe PPH was defined as bleeding that required invasive surgical treatment (hysterectomy, arterial ligation), a transfusion, a transfer to an intensive care unit or death of the patient. The quality of care was evaluated using objective criteria defined by a delay of diagnosis and care and mortality.

**Results** Among the 466 women who delivered vaginally during the study period, 23 had a PPH, link with uterine atony alleged at diagnosis, 18 of which presented signs of severity. In 41% of cases, the delay in diagnosis of PPH was less than 30 min; 70% of women received oxytocin within 10 min after diagnosis. The tranexanique acid was used in 1 case. The examination of the cervix, uterine exploration and uterine massage was performed in 67, 99 and 97%, respectively. The failure of first line treatment involved 24% of patients. Among them, the time between the diagnosis of PPH and administration of blood derivatives was greater than 1 h in a third of cases. The administration of oxytocin delay exceeds 10 min multiplied by 2.5 the risk of severe PPH. However we had 2 deaths in our series.

**Discussion** In our study the optimal period of care was not adequate, obtaining blood derivatives in our institution remains among the factors aggravating Among the main risk factors for PPH, uterine atony was the main source of complication. Bleeding postpartum aggravated in our two patients has led to the deaths from late diagnosis and care that was not optimal. These hemorrhages PP is the leading cause of mortality: 21% of obstetric deaths (25% in the confidential survey 1996–1997) [1].

A hysterectomy was indicated after failure to conservative treatment. The death rate is estimated at 8% following a disorder complicated hemostasis of disseminated intravascular coagulation (DIC). In some series, the mortality rate is estimated between 2 and 4% [2].

**Conclusion** The management of PPH in obstetrics gynecology service The EHU Oran was not optimal. The issue of timing of diagnosis and initial treatment is crucial. Solutions must be sought locally to ensure the administration of essential medicines in time, especially the injection of oxytocin within 10 min after diagnosis.

**Competing interests** None.

**References**

1. Bouvier-Colle MH, Deneux C, Szego E, et al. Estimation de la mortalité maternelle en France: une nouvelle méthode. J Gynecol Obstet Biol Reproduction, 2004;33:421–9.
2. Rossi AC, Lee RH, Chmait RH. Emergency postpartum hys-terectomy for uncontrolled postpartum bleeding. ObstetGynecol 2010;115:637—44.

#### P190 Evolution of the management and prognosis of patients admitted in intensive care unit for exacerbation of chronic obstructive pulmonary disease

##### Ali Habiba Sik^1^, I Talik^1^, Najla Tilouch^1^, Sondes Yaakoubi^1^, Rim Gharbi^1^, Oussama Jaoued^1^, Mohamed Fekih Hassen^1^, Souheil Elatrous^1^

###### ^1^Réanimation Médicale, EPS Taher Sfar Mahdia, Mahdia, Tunisia

####### **Correspondence:** Mohamed Fekih Hassen mohamed.fekihhassen@rns.tn

*Annals of Intensive Care* 2017, **7**(**Suppl 1**):P190

**Introduction** Chronic obstructive pulmonary disease (COPD) is a common pathology that would represent the third cause of death worldwide by 2020. Its evolution is interspersed with episodes of acute exacerbations (AECOPD) that may indicate an admission in intensive care unit in the most.

**Objective** To study the evolution of management modalities of patients admitted in our intensive care unit for AECOPD, to determine their prognosis and to identify factors associated with mortality.

**Patients and methods** It is a retrospective, monocentric study, performed in a Tunisian intensive care unit (ICU) over a period of 10 years. We including all patients admitted in ICU for AECOPD. Parameters collected were demographic features, comorbidities, regular treatment, dyspnea assessed by the MRC scale, initial clinical severity reflected by SAPS II and APACHE II scores, modalities and ICU admission deadlines, initial arterial blood gas analysis, management of patients in the ICU (ventilation modalities, prescription of antibiotics, use of vasoactive drugs) and their outcomes (incidence of nosocomial infections and their sites, length of stay and ICU mortality).

**Results** A total of 512 patients, which represents 17.5% of all hospitalizations, with mean age of 72 years (IQR: 66–77) were admitted for AECOPD during the study period. The mean SAPS II and APACHE II were respectively 32 (IQR: 24–45) and 18 (IQR: 14–24). Of these, 60% were ventilated with NIV whose overall failure rate was 48% with a significant decrease between the beginning and the end of the study (94 vs 31% p = 0.001). Sixty-four percent of patients received antibiotics at admission. The prescription rate of antibiotics has decreased significantly over the years from 82 to 36%. The incidence of nosocomial infections was 18%. It remained steady between 11 and 27%. Their sites were pulmonary in 83% of cases. ICU mortality was 16%. In multivariate analysis, ICU admission deadlines, NIV failure and the use of vasoactive drugs were identified as factors associated with mortality.

**Conclusion** Our study showed the importance of AECOPD in the activity of our ICU. The management of these patients has evolved over the years, which was reflected by the significant decrease in the prescription of antibiotics and the enhancement of NIV success rate. This result could be attributed to the combination of several factors: precocious management of patients, experience of the healthcare team and the use of efficient ventilators. ICU admission deadlines, NIV failure and the use of vasoactive drugs were identified as factors associated with mortality.

**Competing interests** None.

#### P191 Music therapy improves the tolerance of non-invasive ventilation during its introduction

##### Maxime Perrier^1^, Eliane Gouteix^1^, Claude Koubi^1^, Annabelle Escavy^1^, Victoria Guilbaut^1^, Jean-Philippe Fosse^1^

###### ^1^Réanimation surveillance continue, Hôpital Privé Gériatrique Les Sources, Nice, France

####### **Correspondence:** Jean-Philippe Fosse - janfilipfos@gmail.com

*Annals of Intensive Care* 2017, **7**(**Suppl 1**):P191

**Introduction** Aim. Investigate the effect of music therapy on the tolerance of non-invasive ventilation (NIV) during its introduction.

**Patients and methods** *Type of study* Prospective, randomized, single blind, monocentric.

Thirty patients who needed NIV introduction were included.

They were divided into two groups (15 with music and 15 without music) and randomized by block of 4.

The patients were all equipped with headphones and half of them received a 60 min session of music therapy of their choice, and the other half had to keep the headphones on without music.

The main outcome measure was the number of non-programmed interventions by the caregivers during the hour.

Patient’s and caregiver’s feeling were evaluated at the end of the session by a semi-quantitative scale:

- +2 very difficult; +1 difficult; 0 normal; −1 easy; −2 very easy

Pulse rate, systolic and diastolic blood pressure, respiratory rate, pain, peripheral oxygen saturation, arterial blood gas parameters, Glasgow Coma score and Richmond scale were reported before and after the session.

**Results** Music therapy allowed a significant reduction of the number of non-programmed interventions during the hour (0.6 ± 0.74 against 1.73 ± 1.62; p < 0.05).

The patient’s and the caregiver’s feeling of the session was better under music therapy (−1.07 ± 0.88 and −1.07 ± 1.03 against 0.13 ± 0.83 and 0.07 ± 1.03; p < 0.05).

The other parameters were not statistically significant.

**Discussion** Our study showed that music therapy allows a better tolerance of the NIV’s introduction in a quantitative and a qualitative way.

**Conclusion** Music therapy allows a 65.3% reduction in the number of non-programmed interventions by the caregivers, during the first hour of the NIV’s introduction, and a better feeling of the session.

**Competing interests** None.

**References**

1. Bradt J, Dileo C. Music interventions for mechanically ventilated patients. In: The cochrane collaboration. Wiley. 2014.
2. Jaber S, Bahloul H, Guétin S, Chanques G, Sebbane M, Eledjam J–J. Effets de la musicothérapie en réanimation hors sédation chez des patients en cours de sevrage ventilatoire versus des patients non ventilés. Annales Françaises d’Anesthésie et de Réanimation. 2007;26:30–38.

#### P192 Non-invasive ventilation in acute exacerbations of Obesity-Hypoventilation Syndrome (AE/OHS)

##### Jihene Ayachi^1^, Ahmed Khedher^1^, Rahma Ben Jazia^2^, Khaoula Meddeb^1^, Ahmed Abdelghani^2^, Imed Chouchene^1^, Mohamed Boussarsar^3^

###### ^1^Réanimation médicale, CHU Farhat Hached, Sousse, Tunisia; ^2^Pneumologie, CHU Farhat Hached, Sousse, Tunisia; ^3^Réanimation médicale, CHU Farhat Hached. Research Laboratory N° LR14ES05. Faculty of Medicine, Sousse, Tunisia

####### **Correspondence:** Mohamed Boussarsar - hamadi.boussarsar@gmail.com

*Annals of Intensive Care* 2017, **7**(**Suppl 1**):P192

**Introduction** Although NIV is effective in acute hypercapnic COPD-related respiratory failure, its efficacy in AE/OHS has been less demonstrated.

The aim of the study was to evaluate efficacy of NIV in AE/OHS and to identify factors associated with poor prognosis in non-invasive-ventilated AE/OHS patients.

**Patients and methods** A retrospective analysis of all consecutive patients admitted to ICU for AE/OHS. Clinical, ABG’s and outcome characteristics were collected. Factors associated with poor prognosis were identified.

**Results** One hundred patients were included over a 13 years period. 44 patients underwent NIV. They were 66.6 ± 12.6 years aged; BMI, 40.6 ± 7.7 kg/m^2^; SAPSII, 29 ± 13; pH, 7.33 ± 0.08; pCO_2_, 69 ± 22 mmHg. They were scored with grade II encephalopathy score on admission. Mean duration of NIV was 5.1 ± 4.4 days. 14 (32%) patients failed NIV and were intubated with a delay of 85.6 ± 156.7 h. 11 (25%) died and length of stay was 10.7 ± 9.5 days. Four factors were significantly associated with mortality, mMRC, (47 vs 14%; p = 0.02); encephalopathy score, (60 vs 15%; p = 0.008); NIV failure, (64 vs 7%; p = 0.0001); inotropic agents, (58 vs 12.5%; p = 0.004).

**Conclusion** NIV in AE/OHS demonstrates rather efficient. However delay of intubation seems to be of poor prognostic value.

**Competing interests** None.

#### P193 Oxygenotherapy with an oxygen concentrator in intensive care unit: a prospective study

##### Pierre-Julien Cungi^1^, Julien Bordes^1^, Cédric Nguyen^1^, Candice Pierrou^2^, Maximilien Cruc^1^, Alain Benois^3^, Eric Meaudre^1^

###### ^1^Intensive care unit and anesthesiology, Hôpital d’Instruction des Armées Sainte-Anne, Toulon, France; ^2^Intensive care unit and anesthesiology, Hôpital d’Instruction des Armées Laveran, Marseille, France; ^3^Anesthésie réanimation, Hôpital Médico Chirurgical Bouffard, Djibouti, Djibouti

####### **Correspondence:** Pierre-Julien Cungi - pjcungi@gmail.com

*Annals of Intensive Care* 2017, **7**(**Suppl 1**):P193

**Introduction** Oxygen therapy is an essential issue for the French Military Health Service (FMHS). Wounded soldiers are severe trauma patients often burnt and suffering from haemorrhagic shock. They need all along their management oxygen therapy. The theatres of external operations are isolated with limited resources. Their supply is difficult. Currently, 50% of the trauma are intubated. Thirty-three percent of the patient admitted in intensive care suffers from acute respiratory distress syndrome (ARDS). The FMHS chose oxygen concentrator as oxygen source in addition to oxygen pressurized bottles. Their supply can be uncertain in conflict areas. Insufficient data are available concerning the use of oxygen concentrator in intensive care unit.

The primary endpoint was to determine over the total duration of oxygen therapy, the number of days on which the use of pressurized oxygen was needed for patients oxygenated by oxygen concentrator. The secondary endpoints were to identify when pressurized oxygen was needed, describe the characteristics of the population with oxygen therapy and estimate the oxygen quantity economised thanks to the use of oxygen concentrator.

**Materials and methods** The study took place in the forward surgical unit of Bouffard. It’s a French role 3 located in Djibouti Republic in Africa. All patients over 15 admitted in the intensive care and needing oxygen therapy were included. All the patients were oxygenated with an oxygen concentrator. The oxygen concentrators used were Sequaltm Integra 10 OM, that could deliver up to 10 l/min of normobaric oxygen. The ventilator used were Pulmonetictm LTV 1000 and 1200.

**Results** Thirty-six patients were included over the 6 months’ study period. Sixty percent of the patients were men with an average age of 38 (15–90). Sixty percent of them were medical admissions, 22.9% were trauma and 17.1% were surgical admissions. Eight persons died which represented 22.9% of the patients. The mean SAPS II was 38.8. The mean length of stay in intensive care was 9 days (0–44). The mean time of mechanical ventilation was 6 (1–35) days. Among the patients, 76.5% were intubated. Eight patients (22.8%) needed noninvasive ventilation, for six (17%) of them it was after extubation. Two hundred and fifty-one days represents the total number of days of oxygen therapy divided into 142 days of invasive ventilation, 15 days of noninvasive ventilation and 94 days of oxygen mask. The use of pressurized oxygen was necessary 19 times over the 251 days of oxygen therapy which represents 7.5% of the total time. The causes of its use were in ten cases (52.6%) criteria of severe ARDS, in six cases an emergency intubation and in three cases a transfer. One dysfunction of an oxygen concentrator happened during our study. The oxygen concentrator produced 1024 m3 of oxygen over the study period, which represents 104 oxygen pressurized bottles of 50 litres. This enabled an economy of 10,000 euros.

**Conclusion** It is safe to use oxygen concentrator to take care of critically ill patients in limited resources environment. The use of pressurized oxygen is still compulsory in two situations: in case of electricity failure and in case of high FiO_2_ (above 60%). Oxygen concentrators are sufficient in 92.5% of the time. They enable to deliver oxygen any time which is essential when supply is uncertain in conflict areas.

**Competing interests** None.

#### P194 Evaluation of fractional delivered oxygen between nasal cannula and nasal oxygen catheter

##### Frédéric Duprez^1^, Thierry Bonus^1^, Grégory Cuvelier^2^, Sandra Ollieuz^1^, Sharam Machayekhi^1^, Frédéric Paciorkowski^1^, Gregory Reychler^3^

###### ^1^ICU, C.H. Epicura Hornu, Hornu, Belgium; ^2^Laboratoire de l’effort et du mouvement, Condorcet, Tournai, Belgium; ^3^Irec, pôle de pneumologie, ucl, Cliniques Universitaires Saint Luc, Bruxelles, Belgium

####### **Correspondence:** Frédéric Duprez - dtamedical@hotmail.com

*Annals of Intensive Care* 2017, **7**(**Suppl 1**):P194

**Introduction** Oxygen therapy is the main supportive treatment of hypoxia. Nasal cannula (NC) and nasal oxygen catheter (NOC) were used to administer oxygen therapy in hypoxia. Few studies have examined the difference in fractional delivered oxygen (FDO2) between these two systems. The aim of our study was to compare the difference of FDO2 between NC and NOC.

**Materials and methods** On a bench study, a two-compartment model of adult lung (Dual Test Lung DTL, Michigan Instrument) was connected to a Servo i^®^ Ventilator. The ventilator was set in volume-controlled mode. Three minute ventilation (MV: 6/9/12 l/min at Ti/Ttot = 0.33) and two oxygen flow rate (OFR: 2 and 4 l/min) were analyzed. OFR was analyzed with a thermal mass flow meter Vogtlyn™ Red Y. The compliance of the artificial lung was set to 70 ml/cmH_2_O and the resistance set to 5 cmH_2_O/l s^−1^. The FDO2 and MV measurements were made using an iWorx^®^ acquisition system (GA207 gas analyzer and analog/digital IX/228 s) and LabScribe II^®^ software. To simulate the anatomic dead space of the nasopharynx (±50 ml for an adult) we have used a 15 cm length corrugated tubing ISO 22 mm (CT22) at the level of inflow of DTL. NC was introduced at the entry of the CT22 while the NOC was introduced totally into the CT22. Statistical: ANOVA on ranks followed by Student–Newman–Keuls.

**Results**

**Conclusion** In oxygen therapy, with NC or NOC, for a Ti/Ttot = 0.33, FDO2 is influenced by MV, OFR and oxygen system delivery. For the same level of OFR and system delivery, when MV increases, FDO2 decreases (see Table 4). For the same MV and level of OFR, FDO2 was more efficient with NOC than NC. The differences of FDO2 between NOC and NC decrease with increasing MV. The FDO2 fluctuations according to the value of the MV are greater with the NOC to 4 L/min.

**Table 4:** FDO2 comparison between NC and NOC at different OFR and MV

VE(L/min)	NC2 L/min	NOC2 L/min	NC 4 L/min	NOC4 L/min
6	31% (0.5)	37% (0.5)	38% (0.6)	43% (0.5)
9	29% (0.7)	34% (0.6)	34% (0.5)	39% (0.7)
12	26% (0.6)	30% (0.6)	30% (0.7)	34% (0.5)

ANOVA on ranks: p < 0.05, except between: NOC2 (VE 9 L/min) and NOC4 (VE12 L/min)/NC2 (VE 9 L/min) and NOC2 (VE12 L/min)/NOC2 (VE12 L/min) and NC4 (VE12 L/min)/NC4 (VE 9 L/min) and NOC4 (VE 9 L/min)/NOC2 (VE 9 L/min) and NC4 (VE 9 L/min)

In clinical situation, NOC is less used than the NC. Compared to the NC, NOC is an alternative to increase the FDO2 with the same OFR. NOC is more efficient than NC because during expiratory time, anatomical dead space it fills with O_2_, which increases the FDO2. However, if the respiratory frequency increases then expiratory time decreases, filling with O_2_ decreases which reduces FDO2. Note that NOC may become uncomfortable at OFR greater than 5 L/min.

**Competing interests** None.

**Reference**

1. Tiep BL, Nicotra B. Evaluation of a low-flow oxygen-conserving nasal cannula. Am Rev Respir Dis. 1984;130(3):500–2.

#### P195 Variability of fractional delivered oxygen (FDO2) with nasal cannula

##### Frédéric Duprez^1^, Thierry Bonus^1^, Grégory Cuvelier^2^, Sharam Machayekhi^1^, Sandra Ollieuz^1^, Gregory Reychler^3^

###### ^1^ICU, C.H. Epicura Hornu, Hornu, Belgium; ^2^Laboratoire de l’effort et du mouvement, Condorcet, Tournai, Belgium; ^3^ Irec, pôle de pneumologie, ucl, Cliniques Universitaires Saint Luc, Bruxelles, Belgium

####### **Correspondence:** Frédéric Duprez - dtamedical@hotmail.com

*Annals of Intensive Care* 2017, **7**(**Suppl 1**):P195

**Introduction** Nasal Cannula (NC) is an option to deliver oxygen therapy. According to American Thoracic Society (ATS), standard NC delivers a fractional delivered oxygen (FDO2) of 24–40% at supply oxygen flows ranging from 1 to 5 L/min. An equation was proposed by ATS to predict oxygen delivery: FDO2 = 20% + (4 * O2 L/min). Moreover, for ATS, FDO2 is also influenced by respiratory frequency (Rf), tidal volume (Vt) and ratio Ti/Ttot. However, the equation of ATS does not take into account these parameters. Our hypothesis is that these parameters can significantly affect the FDO2. The aim of this study was to determine the effect of Rf, Vt and Ti/Ttot on FDO2.

**Materials and methods** The study was conducted on bench with NC connected to a two compartment adult lung model (Dual Test Lung^®^) (DTL) controlled by a Maquet Servo I^®^ ventilator. One oxygen flow rate (OFR) (5 L/min) and 3 min ventilation (MV: 6/9/12 L/min) with two Ti/Ttot (0.33 and 0.25) were investigated. All settings of MV were generated by modifying Rf (10–40 CPM) and Vt (0.3 and 0.6 L). Inspiratory flows rate (IFR) obtained with settings range from 18 to 48 L/min. OFR was analyzed by a thermal mass flow meter Vogtlyn™ Red Y. FDO2 and MV measurements were made using a iWorx^®^ acquisition system (GA207 gas analyzer) and LabScribe II^®^ software. Compliance of DTL was set to: 0.07 L/cmH_2_O and resistance to: 5 cmH_2_O/L s^−1^. Statistical: ANOVA repeated measures followed by Newman Keuls method.

**Results** FDO2 comparisons between: Ti/Ttot 0.33 and 0.25 and three MV: 6–9–12 L/min at OFR: 5 L/min.

**Conclusion** IFR and OFR are the main determinants of FDO2. Equation of ATS is correct when IFR is equal to 18 L/min.

When IFR is different of this value, Equation of ATS is not appropriate.

In our experiment, with an OFR of 5L/min, when IFR = 18 L/min (MV = 6 L/min and Ti/Ttot = 0.33), the FDO2 is equal to 41% (±1%) (see Table 5). To this value of IFR, the FDO2 is in accordance with the formula of ATS, but when IFR increase beyond 18 L/min, the FDO2 decrease and the formula is not in accordance with ATS. This can be explain because during inspiratory phase, air room (Fractional oxygen = 0.21) entry in airway mixes with OFR (FO2 = 1), which modifies the FDO2. In this case, when IFR increase then FDO2 decrease and vice versa. Medical and paramedical staff must be aware that with patients who receive OFR by nasal cannula, any change of OFR and/or inspiratory flow changes the FDO2. In this case, for maintain the same FDO2, it is necessary that modify the value of OFR.

**Table 5:** FDO2 comparisons between diffferent TI/Ttot and MV at OFR 5 L/min

MV (L/min)	Ti/Ttot = 0.33				Ti/Ttot = 0.25			
	RfxVt	FDO2	RfxVt	FDO2	RfxVt	FDO2	RfxVt	FDO2
6	10 × 0.6	41% (a)	20 × 0.3	42% (d)	10 × 0.6	36% (g)	20 × 0.3	37% (j)
9	15 × 0.6	36% (b)	30 × 0.3	35% (e)	15 × 0.6	32% (h)	30 × 0.3	32% (k)
12	20 × 0.6	31% (c)	40 × 0.3	30% (f)	20 × 0.6	30% (i)	40 × 0.3	29% (l)

*Rf* respiratory frequency (in CPM), *Vt* tidal volume (in Liter)

ANOVA RM results: p < 0.05. No statistical difference are found between: (a–d)/(b–e)/(c–f)/(g–j)/(h–k)/(i–l)/(b–j)/(b–g)/(f–k)/(c–k)/(f–h)/(f–l)/(f–i)/(c–i)/(c–l)/(e–g)

**Competing interests** None.

**Reference**

1. Wagstaff T, Soni N. Performance of six types of oxygen delivery devices at varying respiratory rates. Anaesthesia, 2007;62: 492–503.

#### P196 How to assess FiO_2_ delivered under oxygen mask in clinical practice?

##### Remi Coudroy^1^, Arnaud W Thille^1^, Xavier Drouot^2^, Véronique Diaz^2^, Jean-Claude Meurice^3^, René Robert^1^, Jean-Pierre Frat^1^, the FLORALI study group

###### ^1^Réanimation médicale, CHU de Poitiers, Poitiers, France; ^2^Neurophysiology, CHU de Poitiers, Poitiers, France; ^3^Pneumologie, CHU de Poitiers, Poitiers, France

####### **Correspondence:** - Remi Coudroy remi.coudroy@chu-poitiers.fr

*Annals of Intensive Care* 2017, **7**(**Suppl 1**):P196

**Introduction** The actual FiO_2_ delivered under oxygen mask in patients with acute respiratory failure and the factors that may influence the FiO_2_ are poorly known. In clinical practice, different methods including formula or conversion tables based on oxygen flow can be used to estimate delivered FiO_2_. We aimed to assess first the factors influencing measured values of FiO_2_, and second the best method to estimate FiO_2_ in patients breathing under oxygen mask.

**Patients and methods** We included ICU patients admitted for acute hypoxemic respiratory failure from a previous prospective trial [1] in whom FiO_2_ was measured under oxygen mask using a portable oxygen analyzer. We collected demographic variables and respiratory parameters that may influence measured FiO_2_. Low FiO_2_ was defined according to the median measured FiO_2_.

For each patient, measured FiO_2_ was compared to “Calc + 3%” formula (FiO_2_ = oxygen flow in liters per minute × 0.03 + 0.21) to “Calc + 4%” formula (FiO_2_ = oxygen flow in liters per minute × 0.04 + 0.21), and to a conversion table [2]. A ± 10% limit of agreement for each estimation method was arbitrarily considered acceptable.

**Results** Among the 265 patients included, median measured FiO_2_ was 65% [60–73]. After adjustment on oxygen flow, the three variables independently associated with low measured FiO_2_ using multivariate analysis were patient’s height, a low PaCO_2_, and a respiratory rate greater than 30 breaths/min.

Using paired analysis, each estimation methods differed significantly from measured FiO_2_ (p < 0.0001 for each). Values outside the limits of agreement accounted for 55% of cases for the Calc + 3% formula, 69% for the Calc + 4% formula, and 94% for the conversion table (p < 0.0001).

**Conclusion** Independently from oxygen flow, the 3 major physiologic variables associated with low FiO_2_ delivered under mask were tallness, high respiratory rate and low PaCO_2_. None of the tested methods estimated accurately measured FiO_2_ in patients with acute respiratory failure breathing oxygen through a mask.

**Competing interests** None.

**References**

1. Frat JP, Thille AW, Mercat A, Girault C, Ragot S, Perbet S, et al. High-flow oxygen through nasal cannula in acute hypoxemic respiratory failure. N Engl J Med. 2015;372(23):2185–96.
2. Vincent JL, Rello J, Marshall J, Silva E, Anzueto A, Martin CD, et al. International study of the prevalence and outcomes of infection in intensive care units. JAMA. 2009;302(21):2323–9.

#### P197 Hyperglycemia in ICU: incidence and impact prognosis

##### Olfa Turki^1^, Mabrouk Bahloul^2^, Kais Regaieg^3^, Chtara Kamilia^2^, Hmida Chokri Ben^2^, Hedi Chelly^2^, Mounir Bouaziz^2^

###### ^1^SFAX, CHU HABIB BOURGUIBA, Sfax, Tunisia; ^2^Réanimation polyvalente, Faculté de médecine de Sfax, Sfax, Tunisia; ^3^ICU, CHU Habib Bourguiba, Sfax, Tunisia

####### **Correspondence:** Olfa Turki - olfa.turki.rea@gmail.com

*Annals of Intensive Care* 2017, **7**(**Suppl 1**):P197

**Introduction** Acute hyperglycemia is common in intensive care. It was associated with poor prognosis and increased mortality.

The purpose of our study is to investigate the frequency of hyperglycemia in our ICU, to determine the main causes of high blood sugar and to analyze the impact of this hyperglycemia.

**Patients and methods** Our study is prospective during 3 months. It was conducted in the intensive care unit of the University Hospital Habib Bourguiba Sfax-Tunisia. Were included in our study all patients admitted to the service during the period of the study. For each patient included were collected from the ICU admission, clinical and biological data.

**Results** During the study period, 194 patients were hospitalized in our ICU and the diagnosis of hyperglycemia (>8 mmol/l) was admitted in 93 patients (48%). The comparison between patients who developed hyperglycemia and those free hyperglycemia group showed that, the patients of the first group were significantly older (p < 0.001). Additionally, hyperglycemic patients had more medical history including history of diabetes (p < 0.001), a higher SAPS II (p < 0.05), a more significant frequency of active infections (p < 0.05). Moreover, the presence of hyperglycemia was associated with shock (p < 0.05) and respiratory distress (p < 0.05).

Their evolution was marked by the significantly higher frequency of infectious complications (p < 0.05), thromboembolic complications (p < 0.05) and acute renal failure (p < 0.05). The average duration of mechanical ventilation and the length of stay were also significantly prolonged in hyperglycemia group patients (p < 0.05 for both).

Finally, the presence of hyperglycemia was significantly associated with a higher mortality rate.

**Conclusion** We concluded that hyperglycemia is correlated with poor prognosis of morbidity and mortality. But strict glycemic control remain controversial. Thus, further studies on this subject will be recommended to define the exact place of glycemic control in intensive care.

**Competing interests** None.

#### P198 Acute kidney injury following orthotopic liver transplant: impact of preservation solutions as a risk factor

##### Mona Assefi^1^, Romain Deransy^1^, Hélène Brisson^1^, Antoine Monsel^1^, Filomena Conti^2^, Olivier Scatton^3^, Olivier Langeron^1^

###### ^1^Réanimation chirurgicale polyvalente, Groupe Hospitalier Pitié-Salpêtrière, Paris, France; ^2^Hépato-gastro-entérologie et médecine de la transplantation, Groupe Hospitalier Pitié-Salpêtrière, Paris, France; ^3^Chirurgie digestive, hépato-bilio-pancréatique et transplantation hépatique, Groupe Hospitalier Pitié-Salpêtrière, Paris, France

####### **Correspondence:** Mona Assefi - monaassefi@hotmail.com

*Annals of Intensive Care* 2017, **7**(**Suppl 1**):P198

**Introduction** Acute kidney injury (AKI) is a common complication after orthotopic liver transplantation that can increase morbidity and mortality rates. Identification of AKI risk factors is important to prevent renal failure. The composition of preservation solution for organ transplants limiting ischemia and reperfusion injuries, may play an important role in the development of AKI and has never been studied before. The aim of this study was first to evaluate the impact of four preservation solutions (SCOT, Solutions de Conservations des Organes et Tissus; UW, University of Wisconsin; Celsior; IGL-1) on the occurrence of early AKI after liver transplant, second to identify perioperative risk factors transplant, and third to determine urine biochemical profiles.

**Materials and methods** In this prospective observational study, we analysed clinical and laboratory data, during the preoperative, intraoperative and postoperative periods, from 168 liver transplant recipients, between February 2009 and June 2012. AKI was defined by KDIGO criteria [1].

**Fig. 7:**
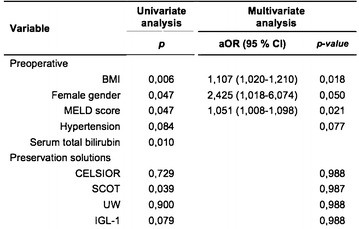
See text for description

**Results** AKI was reported in 86 patients (51.2%) in the 7 days after orthotopic liver transplant. In univariate analysis, SCOT was a risk factor for development of AKI. There was no difference with the other preservation solutions. The other risk factors for AKI occurrence were: MELD score, female gender, Body Mass Index (BMI) and preoperative serum total bilirubin. After fitting a forward stepwise regression model, the type of preservation solution was not anymore an independent risk factor for development of AKI, unlike MELD score, female gender and BMI (p ≤ 0.05). The 1-year mortality, duration of mechanical ventilation, intensive care unit (ICU) and hospital length of stays were significantly increased among patients who developed AKI. Urine biochemistry profiles, although disturbed by the use of diuretics, could highlight an early inadequate renal perfusion after liver transplantation. See Fig. 7.

**Discussion** In this study, a high incidence of post-liver transplant AKI in ICU during the first week after surgery was noted and risk factors of AKI were identified. However, this study failed to demonstrate an impact of preservation solution on AKI occurrence after liver transplant. Risk-factors (MELD, female gender and BMI) are only related to the patient characteristics. Our results should be interpreted with caution given the small sample size, and the monocentric character of the study.

**Conclusion** Solution for organ preservation has no impact on AKI occurrence in ICU within 7 days following orthotopic liver transplant. Because of many factors that can influence AKI, a new study with largest sample size is necessary to demonstrate or eliminate a possible impact of preservation solution.

**Competing interests** None.

**Reference**

1. KDIGO. Kidney Int Suppl. 2012;2:7–24.

#### P199 Management of acute kidney injury and application of RIFLE criteria in a Tunisian medical intensive care unit

##### Hassen Ben Ghezala^1^, Salah Snouda^2^, Chiekh Imen Ben^3^, Moez Kaddour^2^

###### ^1^Réanimation Médicale, Hôpital Henri Mondor, Avenue du Maréchal de Lattre de Tassigny, Créteil, France; ^2^Réanimation Médicale, Hopital regional zaghouan, faculté de médecine de Tunis, Zaghouan, Tunisia; ^3^Teaching department of emergency and intensive care, Regional hospital of Zaghouan, Zaghouan, Tunisia

####### **Correspondence:** Hassen Ben Ghezala - hassen.ghezala@gmail.com

*Annals of Intensive Care* 2017, **7**(**Suppl 1**):P199

**Introduction** Despite the advances made in recent years in the definition and classification of acute renal failure (ARF), epidemiological data remain varied and imprecise. These data basically vary with the population studied and the pathologies that are responsible of the ARF can be particularly complex and intricated especially in emergency and intensive care units (ICU).The aim of our study was to describe the clinical, etiologic, and therapeutic and outcome of ARF, to apply the RIFLE classification (Bellomo and col, Crit Care 2004) to our population of kidney failure patients admitted to intensive care, and assess its relevance in terms of hospital mortality risk.

**Materials and methods** We conducted a retrospective analysis over a period of 48 months (January 2011–December 2014), of records of patients who experienced ARF. The ARF is defined according to the RIFLE classification. We picked up all the clinical and laboratory data, the need for renal replacement therapy, the complications and the disease progression.

**Results** During this study period, 82 out of 1269 patients admitted to our ICU were hospitalized for ARF. They were classified into RIFLE R (30 patients or 37%); RIFLE I (15 patients or 18%) and RIFLE F (37 patients or 45%). The initial reason for visiting the emergency was asthenia (41%), followed by disorders of consciousness and dyspnea. The average age of our patients was 67 ± 13 years with a sex ratio of 2.9. Most found risk factors were hypertension (50%), diabetes (39%) and heart disease (35%). The ARF was essentially functional (56%). The main origin was septic (28%) followed by hypovolemia. Renal replacement therapy was required by 34 patients (41%).

Overall mortality in our population was 45%. Most RIFLE F patients died (62%). Among the risk factors associated with a severe prognosis, we definitely include the RIFLE classification (OR 4.2; CI 95% [2.66–6.65]). The other factors associated with mortality were SAPS II score (OR 6.45; CI 95% [2.77–7.32]) and use of vasopressor agents (OR 3.34; CI 95% [2.07–5.41]).

**Conclusion** ARF is a serious pathology burdened with a heavy morbidity and mortality. It is essentially functional and its main causes are sepsis and hypovolemia. RIFLE classification can predict morality in our Tunisian critically ill patients.

**Competing interests** None.

#### P200 Death rate risk factors of acute renal failure in intensive care department

##### Anwar Armel^1^, Lafrikh Youness^1^, Bensaid Abdelhak^2^, Miloudi Youssef^2^, Al Harrar Najib^2^, Amouzoun Mustapha^3^, Mtioui Noufel^3^, Zamd Mohamed^3^, El Khayat Salma^3^, Medkouri Ghizlane^3^, Benghanam Mohamed^3^, Ramdani Benyounes^3^

###### ^1^Anesthésie réanimation, CHU Ibn Rochd, Casablanca, Morocco; ^2^Anesthésie réanimation, Hopital 20 Aout CHU IBN Rochd, Casablanca, Morocco; ^3^Néphrologie hémodialyse et transplantation rénale, CHU Ibn Rochd, Casablanca, Morocco

####### **Correspondence:** Anwar Armel - armelanwar@gmail.com

*Annals of Intensive Care* 2017, **7**(**Suppl 1**):P200

**Introduction** Acute renal failure (ARF/IRA) is a frequent complication in intensive care unit. Despite of many technical innovations, mortality remains important.

Our study’s aim is to establish the different mortality risk factors ARF/IRA in intensive care.

**Materials and methods** It is a descriptive cross-sectional study interesting 76 patients admitted at the ICU of the 20 August UHC Ibn Rochd, who showed out (ARF/IRA) according to RIFLE classification, on admission or have developed it during hospitalization. The study was made over a period of 18 months.

It excluded all patients with chronic or pre-terminal renal failure.

Statistical analysis used the epi-info test with significance level P < 0.05.

**Results** During the study period, 423 patients were enrolled, 76 of which have completed our inclusion criteria, that is an incidence of 17.96%. Patients average age was 54.57 ± 19.78 years with a male predominance. Antecedents most found are diabetes (43.4%) and high blood pressure (28.94%). Most of the cases were in class F (65.6% of the cases). Classes I and R are 21.9 and 12.5% respectively. Death rate was 64.47%, septic shock was death’s cause in 22.36% of them.

Mortality risk factors selected were age, the disease’s medical background, diabetes or pre-renal failure history, presence of hemodynamic failure, RIFLE stage F and organic nature of the acute renal failure.

**Conclusion** Acute renal failure occurrence in intensive care is a critical step with a very derogatory prognosis significance. A better understanding of its risk factors and prognosis is basic for more effective management.

**Competing interests** None.

#### P201 Early management of tumor lysis syndrome in intensive care unit

##### Dhouha Lakhdhar^1^, Florent Montini^1^, Sébastien Moschietto^1^

###### ^1^Reanimation, Centre Hôspitalier Henri Duffaut, Avignon, France

####### **Correspondence:** Dhouha Lakhdhar - lakdardoha@gmail.com

*Annals of Intensive Care* 2017, **7**(**Suppl 1**):P201

**Introduction** Tumor lysis syndrome (TLS) occur with tumor breakdown usually by response to chemotherapy. It may also occur spontaneously. The rapid destruction of malignant cells release intracellular content into the extracellular compartment, inducing metabolic and electrolytic imbalances, which result in acute kidney injury. Early prophylactic therapy is essential to ovoid the occurence of TLS and prevent life threatening complications.

The aim of this study was to prove the value of the early management and monitoring of patients with high risk of TLS in intensive care units (ICU).

**Patients and methods** This was a mono-centric, descriptive and retrospective study. During a 3 year period, from January 2013 to June 2016, case notes of fifteen patients with hematologic malignancies were reviewed. They were admitted in intensive care unit for chemotherapy induction for patients with high risk of TLS, or for established TLS. We collected informations regarding clinical and biological presentation, treatment and outcome.

**Results** Fifteen patients were included. The median age was 65 [45–73] years, with IGS II score at the admission 40 [31–51]. Mainly male (87%). The major part had acute myeloid leukemia and highly aggressive lymphoma. Twelve patients were admitted in ICU for chemotherapy induction. Four patients had spontaneous TLS and three patients had TLS after chemotherapy started in oncology department. Concerning patients who had acute myeloid leukemia the median rate of white blood cells count was 84,000 [15,000–166,000] white blood cells per microliter. Seven patients had tumoral renal infiltration. The median CKD-epi score was 77 [23.9–95.6] mL/min/1.73 m^2^. According to KDICO score, five patients were in stage G1, five were in stage G4, four were in stage G2 and one in stage G3a. At the time of admission, seven patients had hyperphosphatemia, nine had hypocalcemia, five had hyperuricemia, and only one had hyperkalemia. Chemotherapy was started in ICU for twelve patients. The median hydration amount was 2.5 [1–3] l the first 48 h. The aggressive IV hydration was monitored with daily heart ultrasound examination and urine output. Recombinant urate oxidase (rasburicase) was given to all patients, the number of doses depended on urecemia levels. Six patients needed only one dose of rasburicase. Seven patients had renal replacement therapy (RRT) and it lasted 48 [48–72] h. The RRT was prophylactic in four cases started when phophatemia was more than 2 mmol/L, and therapeutic for renal failure and established TLS in three cases. The median duration stay in ICU was 5 [4–7] j. Thirteen patients left the ICU without major metabolic dysfunction. Two patients deceased due to infectious complications.

**Discussion** Monitoring of electrolytes was done on average, three times a day which is hard to do in onco-hematology unit. The early use of rasburicase and the aggressive IV hydration helped to prevent TLS for seven patients. The aggressive IV hydration was made according to echocardiography data and close monitoring of vital signs and urine output which has allowed to avoid volume overload and acute pulmonary edema. The early prophylactic RRT prevented renal failure and metabolic complications.

**Conclusion** Early management of TLS in ICU can prevent TLS and most of its serious complications and should be considered in TLS prophylaxis recommendations.

**Competing interests** None.

#### P202 The added value of plasma or urinary NGAL concentration in clinical practice

##### Emilien Gregoire^1^, Guillaume Claisse^2^, Julien Guiot^3^, Philippe Morimont^3^, Jean-Marie Krzesinski^1^, Christophe Mariat^4^, Bernard Lambermont^3^, Etienne Cavalier^5^, Pierre Delanaye^6^

###### ^1^Nephrology, C.H.U de Liège - Sart Tilman, Liège, Belgium; ^2^Nephrology, Hospital Center University De Saint-Étienne, Saint-Priest-en-Jarez, France; ^3^Medical intensive care, C.H.U de Liège - Sart Tilman, Liège, Belgium; ^4^Néphrologie, Centre Hospitalier Universitaire de Saint-Étienne, Saint-Étienne, France; ^5^Biologie clinique, C.H.U de Liège - Sart Tilman, Liège, Belgium; ^6^C.H.U de Liège - Sart Tilman, Liège, Belgium

####### **Correspondence:** Pierre Delanaye - pierre_delanaye@yahoo.fr

*Annals of Intensive Care* 2017, **7**(**Suppl 1**):P202

**Introduction** Plasma and urinary NGAL concentrations have been proposed for the early diagnosis of acute kidney injury (AKI). However, the added value of these parameters on simple usual clinical data (such as baseline serum creatinine and/or diuresis) has been questioned [1].

**Patients and methods** We measured both urinary and plasma NGAL concentration (Bioporto, Gentofte, Denmark on Roche Cobas 6000) in 98 patients admitted to the medical ICU of an Academic Hospital. The measurement was done in the first 24 h after ICU admission. Renal transplanted and dialysis patients were excluded. AKI was defined according to serum creatinine criteria of the KDIGO guidelines (1.5–1.9 times baseline or ≥0.3 mg/dL increase).

**Results** Three patients were excluded from the analysis because of early (in the first 24 h) death. For the 95 patients, median [IQR] age was 64 years [20], mean (SD) SAPSII score was 46 (17), 39% were septic, median baseline (=at admission in ICU) serum creatinine was 9.7 [8.4] mg/dL, and median first 24 h diuresis was 1432 [1340] mL. ICU mortality was 14.7%. Prevalence of AKI stage 1 was 21%. Both urinary (expressed as the ratio of NGAL on urinary creatinine) and plasma NGAL were predictive of AKI Stage 1. Predictive value of plasmatic measurements was higher than the urinary one (AUC of 0.627 and 0.758, respectively, p = 0.0273 between AUC), but not higher than either baseline serum creatinine (AUC = 0.737) or 24 h diuresis (AUC = 0.735). Backward multivariate regression showed that plasma NGAL concentration was associated with serum creatinine, CRP and albumin, whereas urinary NGAL was associated with leucocyturia and baseline creatinine.

**Discussion** Previous positive studies with NGAL did not compare the performance of this costly biomarker with simple usual clinical parameters to predict AKI. Moreover, several parameters were associated with NGAL concentrations with a high risk of collinearity (CRP) and/or false positive results (leucocyturia).

**Conclusion** Our data do not support any added value of NGAL concentration over baseline serum creatinine or urine output to predict AKI.

**Competing interests** None.

**Reference**

1. Vanmassenhove J. Urinary and serum biomarkers for the diagnosis of acute kidney injury: an in-depth review of the literature. Nephrol Dial Transplant, 2013;28:254–273.

#### P203 Interest of the resistivity index in the detection of renal aggression in intensive care

##### Soumia Benbernou^1^, Sofiane Ilies^1^, Abdelkader Azza^2^, Khalida Bouyacoub^3^, Meriem Louail^4^, Houria Mokhtari-Djebli^5^

###### ^1^URGENCES MEDICALES CHUORAN, Faculté de médecine d’Oran, Oran, Algeria; ^2^Emergencies, CHU ORAN, Oran, Algeria; ^3^Urgences medicales, Chu Oran, oran, Algeria; ^4^Anesthésie réanimation chirurgicale, EHU 1er Novembre, Oran, Algeria; ^5^Urgences Médicales et réanimation, Centre Hospitalier et Universitaire d’Oran, Oran, Algeria

####### **Correspondence:** Soumia Benbernou - gsoumia@hotmail.com

*Annals of Intensive Care* 2017, **7**(**Suppl 1**):P203

**Introduction** Acute renal failure (ARF) is a common entity in intensive care, concern that the heavy morbidity and mortality it is associated [1].

Early diagnosis of this entity remains difficult, neither diuresis and creatinine are early parameters in the diagnosis of ARF. The kidney is an organ that suffers long to become faulty, the priority is to recognize renal aggression and to achieve a therapeutic allowing reversibility of the infringement.

A number of markers have been developed for the diagnosis of the IRA but costs remain high not allowing their routine use. The measurement of resistance index with the renal Doppler could be a solution for the diagnosis of aggression and also of the etiology.

**Patients and methods** Performed prospective study in the intensive care unit of emergency room from January 2016 to June 2016. Bringing together ninety-two patients.

The material used, PHILIPS brand with cardiac ultrasound probe of 3.5 MHz.

Three patient groups are made selon their renal resistive index, group A with IR < 0.75, group B, 0.75 < IR < 0.90, and Group C with IR^3^ to 0.90.

**Results** Patients in group A number of 33 patients showed no impairment of renal function. Patients in group B at number 41 which are supported effectively do not progress to the group C.

Five patients progressed to group C.

Patients in group C at number eighteen, lives with a higher resistance index of 0.90 or required an extra renal purification.

Patients in group C are classified as risk or failure check the RIFLE classification, eleven have a persistent hyper hydration seven with persistent hypovolemia.

The elevation of creatinine was seen later within 48 h after the IR >90.

**Discussion** In our series the resistance index has a value of early diagnosis of renal prognosis aggression in the occurrence and development of renal failure. Renal doppler associated with a strictly applied standardized protocol achieves the two goals of monitoring who aid in the diagnosis and guide treatment. Although the recommendations of experts to this tool provides that it should probably not use the resistance index measured by renal doppler to diagnose or treat an IRA (Grade 2) [2]. Identifying the cause of kidney aggression is a prerequisite before any therapeutic action. Hypovolemia and soda hydro overload are the causes principales. Excess filling hyper intra thoracic pressure and hypoxia are the main causes of kidney congestion.

**Conclusion** Doppler is an early renal medium in the diagnosis of renal aggression. A larger series could assert this observation.

**Competing interests** None.

**References**

1. Demiselle J. Diagnostic strategies for renal impairment in the intensive care unit. Réanimation. 2015;24:625–35.
2. Recommendations formalized expert Acute renal failure in the perioperative and intensive care (excluding extrarenal purification techniques). Anesth Reanim. 2016;2:184–205.

#### P204 Peri-partum thrombotic microangiopathic syndrome: diagnosis and treatment in intensive care unit, a single center retrospective study

##### Romain Arrestier^1^, Fabrice Daviaud^1^, Xavier Laborne Francois^2^, Elsa Brocas^1^, Gérald Choukroun^1^

###### ^1^Réanimation polyvalente, C.H. Sud Francilien, Corbeil-Essonnes, France; ^2^Samu, C.H. Sud Francilien, Corbeil-Essonnes, France

####### **Correspondence:** Romain Arrestier - arrestier_romain@yahoo.fr

*Annals of Intensive Care* 2017, **7**(**Suppl 1**):P204

**Introduction** Peri-partum thrombotic microangiopathic syndrome (PP-TMA) is a frequent situation in intensive care unit, mainly with HELLP syndrome. Other syndromes as Thrombotic Thrombocytopenic Syndrome (PTT) and atypical Hemolytic and Uremic Syndrome (aHUS), need specific treatments. When PP-TMA is diagnosed there are no clear guidelines about the right treatment to start after foetal extraction. Plasma exchange (PE) should be used to treat PTT. The prognosis of patients is improved when this technique is started early in the course of the disease but several days are necessary to obtain results of diagnosis tests. The aim of our study was to evaluate characteristics of the patients with PP-TMA who were treated by PE in ICU and to define the characteristics associated with the beginning of PE.

**Patients and methods** We conducted a retrospective analysis of all the women with PP-TMA admitted in ICU between 2012 and 2016, in a hospital with a level 3 maternity with about 5000 deliveries a year and the possibility to perform plasmatic exchanges. We have differentiated 2 groups, with PE and without PE.

**Results** 38 patients were admitted in our unit with PP-TMA. 33 were treated without PE and 5 with PE. 32/33 patients in the no PE group had a diagnosis of HELLP and 4/5 in the other group. PE group had a greater IGS2 score (21.62 [8–84] vs 34 [13–52]), had significantly more pre-eclampsia, 4/5 (80%) versus 7/33 (21%) p = 0.0187. PE were started at an average of 1.6 days after foetal extraction, and with an average of 5 sessions. Patients of the PE group had significantly lower nadir of hemoglobin but also lower hemoglobin level at day 2 and day 5. Nadir of platelets count was also lower and level remain lower at days 1, 2, 3 and 5. Acute kidney injury (using Kdigo classification) was more frequent with a higher rate of dialysis in ICU, in the PE group (4/5 (80%) vs 3/33 (9%) p = 0.0022) with a more frequent need for dialysis at the exit of ICU. Proteinuria was significantly higher in the PE group (862.8 mg/mmol vs 308.41 mg/mmol, p = 0.0134). ADAMTS13 dosage was done only in patients with PE. We find a diminution of ADAMTS13 activity (before PE) with an average of 53% [40–70] in this group. There was no death, and adverse effects were not significantly different.

**Discussion** This study shows that PE was used when diagnosis was uncertain in the most severe form of PP-TMA. Low hemoglobin, low platelets, acute kidney injury and high level of proteinuria are the main factors associated with the decision to begin PE. This technique was safe and not associated with major adverse events. Several studies show that there are physiopathological crossovers between diseases associated with PP-TMA, for example low ADAMTS13 activity in HELLP or mutation in alternative complement pathway which induced HELLP. Moreover, studies and case reports show a benefit of PE in HELLP syndrome. Our study did not find significant difference in adverse events (maybe due to a lack of power), but this is another argument to discuss PE in the management of PP-TMA in severe patients. The main limits of our study are that none of the patients who had a plasmatic exchange had a diagnosis of PTT and that diagnosis tests were not performed in all patients with PP-TMA (complements level, ADAMTS13…).

**Conclusion** PP-TMA treated with PE has lower hemoglobin, lower platelets, higher rate of kidney injury and proteinuria than those treated without PE. No difference were found for adverse events. Begining of PE should be discussed for management of a PP-TMA without amelioration after fœtal extraction.

**Competing interests** None.

**Reference**

1. Fakhouri F, Vercel C, Frémeaux-Bacchi V. Obstetric nephrology: AKI and thrombotic microangiopathies in pregnancy. Clin J Am Soc Nephrol. 2012;7(12):2100–6.

#### P205 Which patients with acute respiratory distress syndrome (ARDS) have diffuse alveolar damage?

##### Arnaud W Thille^1^, Oscar Peñuelas^2^, José-Angel Lorente^2^, Pablo Cardinal-Fernandez^2^, José-Maria Rodriguez^3^, José-Antonio Aramburu^3^, Andres Esteban^2^, Fernando Frutos-Vivar^2^

###### ^1^Réanimation Médicale, CHU de Poitiers, Poitiers, France; ^2^Departamento de cuidados intensivos, Hospital Universitario de Getafe, Getafe, Spain; ^3^Departamento de anatomía patología, Hospital Universitario de Getafe, Getafe, Spain

####### **Correspondence:** aw.thille@gmail.com Arnaud W Thille

*Annals of Intensive Care* 2017, **7**(**Suppl 1**):P205

**Introduction** Diffuse alveolar damage (DAD) is the typical histological feature of acute respiratory distress syndrome (ARDS). However, in a previous study including 356 patients with criteria for ARDS, we found that only 45% of them had DAD at autopsy exanimation [1]. It has been shown that patients with ARDS and DAD on open lung biopsy had higher mortality than those without DAD [2]. Thus, we aimed to identify markers associated with DAD in patients with ARDS.

**Patients and methods** We included the 356 patients who met criteria for ARDS at time of death in our large database of clinical autopsies [1]. We assessed the proportion of DAD according to the severity of ARDS including the degree of hypoxemia and the 4 ancillary variables from the Berlin definition: use of high levels of positive end-expiratory pressure (PEEP at least 10 cmH_2_O), radiographic severity (3 or 4 quadrants on chest radiograph), altered respiratory system compliance (≤40 mL/cmH_2_O), and large dead space defined as a corrected expired volume per minute (≥10 L/min).

**Results** DAD was associated with all the severity markers above-mentioned using univariate analysis. After multivariable logistic regression, the three markers independently associated with presence of DAD were the gender with an odds ratio (OR) of 1.76 [1.08–2.86] for females (p = 0.02), the degree of hypoxemia with an OR of 3.88 [1.51–9.94] for moderate and 7.22 [2.83–18.3] for severe ARDS (p < 0.01 for both), and the radiographic severity with an OR of 3.56 [1.88–6.72] (p < 0.01) for patients with diffuse opacities involving the 4 quadrants. DAD was found in 67% of patients with PaO_2_/FiO_2_ ≤ 100 mmHg and diffuse opacities.

**Conclusion** DAD was significantly more frequent in females. In addition to the severity of hypoxemia, diffuse infiltrates involving the 4 quadrants was a significant marker of DAD.

**Competing interests** None.

**References**

1. Thille AW, Esteban A, Fernandez-Segoviano P, Rodriguez JM, Aramburu JA, Penuelas O, Cortes-Puch I, Cardinal-Fernandez P, Lorente JA, Frutos-Vivar F. Comparison of the Berlin definition for acute respiratory distress syndrome with autopsy. Am J Respir Crit Care Med. 2013;187:761–67.
2. Cardinal-Fernandez P, Bajwa EK, Dominguez-Calvo A, Menendez JM, Papazian L, Thompson BT. The presence of diffuse alveolar damage on open lung biopsy is associated with mortality in patients with acute respiratory distress syndrome: a systematic review and meta-analysis. Chest. 2016;149:1155–64.

#### P206 Functional imaging of lung macrophage inflammation during high-volume ventilation using PET/CT

##### Laurent Bitker^1^, Nicolas Costes^2^, Didier Le Bars^2^, Franck Lavenne^2^, Mojgan Devouassoux^3^, Jean-Christophe Richard^1^

###### ^1^Réanimation médicale, Hospital Croix-Rousse, Lyon, France; ^2^Positron emission tomography department, CERMEP, Bron, France; ^3^Service d’anatomo-pathologie, Hospital Croix-Rousse, Lyon, France

####### **Correspondence:** Laurent Bitker - laurent.bitker@chu-lyon.fr

*Annals of Intensive Care* 2017, **7**(**Suppl 1**):P206

**Introduction** Ventilation Induced Lung Injury (VILI) is responsible for an increased mortality in ARDS [1]. Mechanical ventilation may trigger an inflammatory response, comprising alveolar macrophage activation and recruitment, which may be specifically, repeatedly and spatially assessed by functional imaging techniques such as Positron Emission Tomography combined with Computerized Tomography (PET/CT) [2]. 11C-PK11195 is a PET radiotracer with potential to quantify macrophage inflammation. We aim to assess its performance to detect lung macrophage recruitment in an experimental high-volume VILI model.

**Materials and methods** VILI was performed in 5 anesthetized pigs under neuromuscular blockade by rapidly increasing the tidal volume (Vt) to obtain a transpulmonary pressure (TPP) between 35 and 40 cmH_2_O under zero end-expiratory pressure. PET/CT acquisitions were performed before (T1) and after 4 h of high-volume ventilation (T2), and image-derived measurements were realized on the whole lungs, and regionally on 6 distinct lung regions (divided along the anteroposterior and the cephalocaudal axes). 11C-PK11195 lung uptake was estimated using the Standardized Uptake Value (SUV), normalized to the CT-derived tissue fraction in the region of interest (ROI). Mechanical lung aggression was estimated by CT-derived dynamic and static strains, and tidal alveolar hyperinflation (expressed as a fraction of the tidal variation in the ROI volume). After euthanasia, alveolar damage and macrophage recruitment were assessed in the 6 lung regions, using semi-quantitative scores.

**Results** Between T1 and T2, VT and TPP significantly increased from 6.0 ± 0.1 to 49.4 ± 2.9 ml/kg and 8.6 ± 1.5 to 37.8 ± 4.4 cmH_2_O, respectively. SUV on the whole lung significantly increased from 1.8 ± 0.6 to 3.0 ± 0.5 between T1 and T2 and dynamic strain from 0.36 ± 0 to 2.1 ± 0.2, whereas static strain did not significantly vary. Tidal alveolar hyperinflation significantly increased from 18 ± 4 to 68 ± 3% on the whole lung between T1 and T2. Regionally, dynamic strain, and tidal alveolar hyperinflation significantly differed between regions, as well as between T1 and T2. Regional SUV differed between T1 and T2 but not between regions. Regional static strain did not differ between regions, nor between T1 and T2. In multivariate analysis, regional SUV was independently and significantly associated with dynamic strain and tidal alveolar hyperinflation. Histologic analysis showed significant regional differences in alveolar damage but not in macrophage recruitment. SUV was positively associated with macrophage recruitment but not with alveolar damage.

**Discussion** In this experimental VILI model, 11C-PK11195 SUV was significantly increased after 4 h of injurious ventilation, and was significantly and positively associated with high-volume CT-derived mechanical parameters, such as dynamic strain and tidal alveolar hyperinflation. The radiotracer’s specificity for macrophages is confirmed by the SUV significant association with macrophage recruitment and the lack of association with alveolar inflammatory edema.

**Conclusion** 11C-PK11195 is a macrophage-specific PET radiotracer, with potential to dynamically and specifically assess alveolar macrophage inflammation induced by high-volume ventilation.

**Competing interests** Research founded by the French society of intensive care medicine (SRLF) and La Fondation pour la Recherche Médicale (DEA20140630499).

**References**

1. ARDS Network. Ventilation with lower tidal volumes as compared with traditional tidal volumes for acute lung injury and the acute respiratory distress syndrome. N Engl J Med. 2000;342(18):1301–8.
2. Frank JA, Wray CM, McAuley DF, Schwendener R, Matthay MA. Alveolar macrophages contribute to alveolar barrier dysfunction in ventilator-induced lung injury. Am J Physiol Lung Cell Mol Physiol. 2006;291(6):L1191–8.

#### P208 Reverse triggering on mild and severe ARDS patients

##### Malika Mechati^1^, Marc Gainnier^1^, Laurent Papazian^1^, Christophe Guervilly^1^

###### ^1^Reanimation des urgences et medicale, CHU la Timone 2, Marseille, France

####### **Correspondence:** Malika Mechati - malikamechati@hotmail.fr

*Annals of Intensive Care* 2017, **7**(**Suppl 1**):P208

**Introduction** The reverse triggering (RT) is the term used to name the contractions reflexes of the muscle diaphragmatic provoked (“triggered”) by the periodic insufflations, delivered by the ventilator, at sedated patients under mechanical ventilation [1]. The RT constitutes a new form of patient–ventilator interaction clinically difficult to detect and little known. The RT could have potential implications during the management of acute respiratory distress syndrome (ARDS). At present, the management of severe ARDS consists among others, on the use of an early and systematic perfusion of neuromuscular blockade agents (NMBA) during a 48 h’ period, continuation to the ACURASYS essay which showed a reduction of the mortality in the group of the severe ARDS patient receiving NMBA. The reason of the beneficial effect of curare is not perfectly known. It is possible that the phenomenon of RT is a mechanism implied in the deleterious role of the mechanical ventilation during ARDS. The abolition of this phenomenon by NMBA could explain the beneficial effect of NMBA in ARDS [2]. The objective was to look for the phenomenon of RT in two groups of ARDS patients: a group receiving NMBA and a group not receiving NMBA.

**Patients and methods** Physiological observational and comparative study in intensive care units. We record continuous signals of airflow, airway pressure, and esophageal pressure during 24 h of consecutives patients with ARDS criteria and PaO_2_/FiO_2_ ratio ≤150 at a positive end-expiratory pressure (PEEP) of 5 cmH_2_O evolving for less than 48 h under mechanical ventilation.

**Fig. 8:**
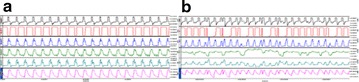
Recording of continuous signals of airflow, airway pressure, esophageal pressure, transpulmonary pressure and tidal volume. **a** NMBA group; **b** unless NMBA group

**Results** Recording of esophageal pressure of 21 consecutives moderate to severe ARDS patients were blinded analyzed (group NMBA n = 11; group unless NMBA n = 10). Any phenomenon of RT was observed in the group of mild ARDS patients receiving NMBA (Fig. 8a). We confirmed the existence of RT on 3 patients of 10 in the group of mild ARDS who not receiving NMBA (*p* = 0.05) (Fig 8b).

**Discussion** One of the main limits was the quality of the collection of the signal of esophageal pressure. The monitoring of esophageal pressure is technically difficult, and can d influence the quality of the signal and the reliability of the results.

**Conclusion** This study confirms the existence of the phenomenon of reverse triggering among deeply sedated patients not receiving NMBA with a 30% incidence. More research is needed to determine if the reverse triggering is a risk factor independent from VILI, associated with the bad prognosis of severe SDRA patients and, if a strategy of early treatment based on NMBA, could improve the prognosis of reached patients.

**Competing interests** None.

**References**

1. Akoumianaki E. Mechanical ventilation-induced reverse-triggered breaths: a frequently unrecognized form of neuromechanical coupling. Chest. 2013;143(4):927–38.
2. Papazian L. Neuromuscular blockers in early acute respiratory distress syndrome. N Engl J Med. 2010;363(12):1107–16.

#### P210 Airway and transpulmonary driving pressure selected by INTELLiVENT-ASV after recruitment in ARDS patients

##### Aude Garnero^1^, Jean Michel Arnal^1^

###### ^1^Réanimation polyvalente, Hôpital Sainte Musse, Toulon, France

####### **Correspondence:** Aude Garnero) - aude.garnero@gmail.com

*Annals of Intensive Care* 2017, **7**(**Suppl 1**):P210

**Introduction** In acute respiratory distress syndrome (ARDS), airway driving pressure (ΔPAW = Plateau pressure (PPLAT) minus total PEEP) reflects the strain applied to the respiratory system. ΔPAW below 15 cmH_2_O is associated with increased survival [1]. However, transpulmonary driving pressure (ΔPTP) is a better assessment of the strain applied to the lung. Treatment strategy leading to ΔPTP below 8 cmH_2_O seems associated with increased survival [2]. INTELLiVENT-ASV is a fully closed loop ventilation mode that adjusts minute volume according to PETCO2 and select tidal volume according to respiratory mechanics. This prospective physiological study aimed to verify if the combination of an optimal recruitment strategy and a closed loop ventilation mode, namely INTELLiVENT-ASV could achieve a lung protective ventilation. Thus ΔPAW and ΔPTP selected by INTELLiVENT-ASV after recruitment were measured in moderate and severe ARDS patients.

**Patients and methods** The study was conducted in the general ICU of Hopital Sainte Musse, Toulon, France. Eligible participants were adults aged 18 or over, with early onset moderate or severe ARDS, invasively ventilated for less than 24 h at the time of inclusion. Exclusion criteria were contraindication to insert nasogastric tube, bronchopleural fistula, emphysema, pneumothorax, increased intracranial pressure, acute corpulmonale, hemodynamic instability, pregnancy. Patients were sedated and ventilated in controlled mode. An esophageal balloon was inserted and position was controlled by an occlusion test. A 10 s sustained inflation recruitment maneuver was performed targeting a transpulmonary pressure at 27 cmH_2_0 during the maneuver. Then a decremental PEEP trial was performed to determine the level of PEEP associated with end-expiratory transpulmonary pressure (PTP TE) at 1–2 cmH_2_O. At this level, airway and esophageal pressure (PESO) were measured at end-inspiration and end-expiration using 5 s end-inspiratory and end-expiratory occlusions, respectively. Transpulmonary pressure was calculated as airway minus esophageal pressure at end-inspiration and end-expiration. ΔPTP was calculated as the difference between PTP TI and PTP TE.

**Results** Fourteen patients were included between February 2016 and August 2016, 8 moderate and 6 severe ARDS, 13 were paralyzed. Sex ratio = 11/3, Age = 59 ± 18 years, SAPS II = 56 ± 22, BMI = 26 ± 5.

For the total respiratory system: PEEP TOT = 17 ± 4 cmH_2_O, P PLAT = 26 ± 5 cmH_2_O, ΔPAW = 9 ± 1 cmH_2_O, VT = 6.0 ± 1.0 mL/KgPBW, P PEAK = 29 ± 5 cmH_2_O, CRS = 48 ± 15 ml/cmH_2_O.

For the chest wall: PESO TI = 18 ± 5 cmH_2_O, PESO TE = 15 ± 5 cmH_2_O.

For the lungs: PTP TI = 8 ± 3 cmH_2_O, PTP TE= 2 ± 2 cmH_2_O, ΔPTP = 6 ± 2 cmH_2_O, CL = 81 ± 39 ml/cmH_2_O.

**Conclusion** After a recruitment maneuver and a PEEP set to achieve positive PTP TE, INTELLiVENT-ASV selected low and safe ΔPAW and ΔPTP in moderate to severe ARDS patients. This strategy seems lung protective.

**Competing interests** None.

**References**

1. Amato MB. Driving pressure and survival in the acute respiratory distress syndrome. N Engl J Med. 2015;372:747–55.
2. Baedorf Kassis E. Mortality and pulmonary mechanics in relation to respiratory system and transpulmonary driving pressures in ARDS. Intensive Care Med. 2016;42:1206–13.

#### P211 BIPAP-APRV airway pressure release ventilation with spontaneous ventilation in ARDS patients under VV-ECMO

##### Hadrien Roze^1^, Jean Christophe Richard^2^, Benjamin Repusseau^1^, Antoine Dewitte^1^, Olivier Joannes-Boyau^1^, Alexandre Ouattara^1^

###### ^1^Sar 2, réanimation médico-chirurgicale magellan, CHU de Bordeaux, Bordeaux, France; ^2^Departement des urgences, C.H. Annecy Genevois, Annecy, France

####### **Correspondence:** Hadrien Roze - hadrien.roze@chu-bordeaux.fr

*Annals of Intensive Care* 2017, **7**(**Suppl 1**):P211

**Introduction** Because gas exchanges under ECMO are partially controlled independently of mechanical ventilation (MV), opposite strategies can be considered from fully controlled MV to early spontaneous ventilation (SV). To limit the risks associated with fully controlled ventilation, we changed our MV strategy by choosing a mode facilitating SV: BiPAP-APRV. This study in severe patients compared two consecutive strategies with or without early SV under ECMO.

**Patients and methods** Fully controlled MV used pressure assist control ventilation (PAC) without SV whereas SV was promoted with BiPAP-APRV using the same settings of controlled ventilation plus 40% of SV. In both group high inspiratory pressure was 24cmH20, PEEP was around 12 cmH_2_O with a driving pressure strickly below 14cmH20 and respiratory frequency was 12 cycles/min.

**Fig. 9:**
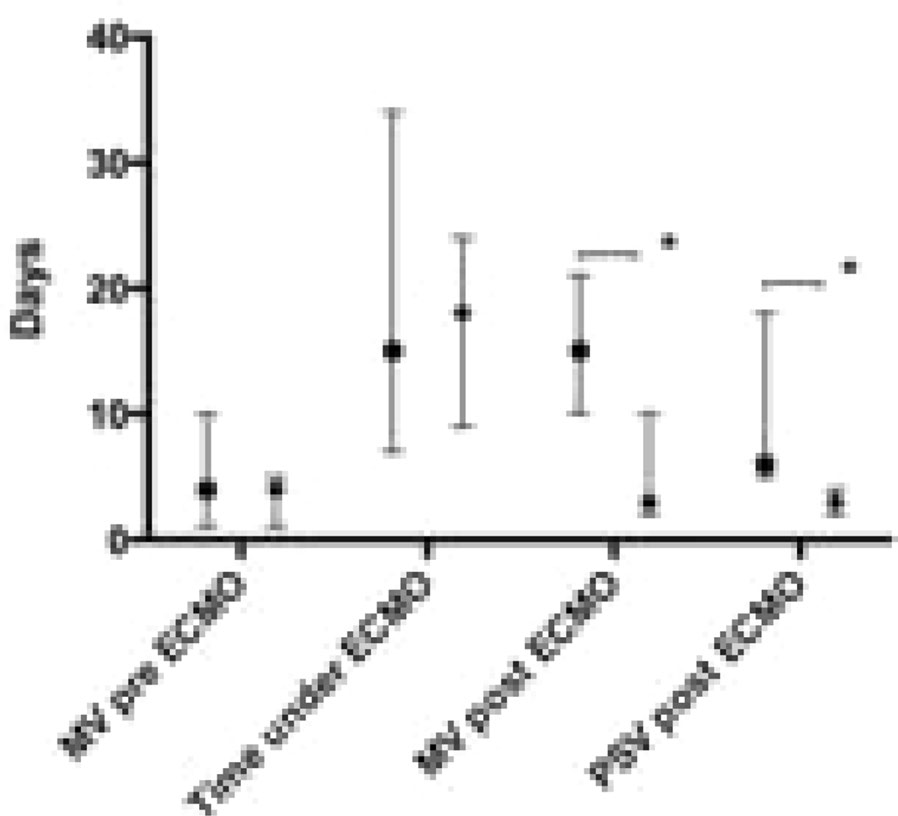
Circles are PAC group, rhombus are APRV group. *MV* mechanical ventilation, *PSV* pressure support ventilation. Data are presented as median (IQR), Comparison between the 2 groups at each time Mann–Whithney Test, *p < 0.05

**Results** 14 similar patients were analysed, 7 in each group. Patients in BIPAP-APRV spent 78% (40–88) of their time with SV under ECMO, this time was inversely correlated with weaning time under pressure support ventilation after ECMO removal, R2 0.54, p = 0.04. Duration of sedation was similar in both groups. At SV initiation with APRV Sedation Agitation Scale was 1 (1–2). In the 500 cycles recorded, controlled and spontaneous VT were 1.6 (1.3–2.7) and 1.2 (1.1–1.5) ml kg^−1^ PBW respectively (p < 0.0001) and the % of SV was 39 (30–48) %. Patients spent the same time under ECMO in both groups but the weaning time under pressure support ventilation (PSV) after ECMO removal had a significant median reduction of 3 days in the BIPAP-APRV group, p = 0.03 (Fig. 9).

**Discussion**

We reported the feasibility of a protocol based on BIPAP-APRV aiming at resuming SV as soon as possible in ARDS patients under ECMO. The occurrence of spontaneous inspiratory efforts in ARDS patients can major variability of transpulmonary pressure and as result jeopardise VT and driving pressure control. This might be an issue if protective ventilation is not guaranteed anymore. VT with BiPAP-APRV remains within safe range when the ratio of spontaneous minute ventilation to total minute ventilation is between 30 and 40% [1].

BiPAP-APRV is more efficient than PSV to increase lung aeration in patients with ARDS [1]. Recruitment of dependent region is more likely to achieve if SV is not supported by synchronized positive airway pressure as during BiPAP-APRV [2]. Our strategy targeting a percentage of SV between 30 and 40% with high PEEP could be viewed as a compromise in order to promote SV and protective ventilation at the same time.

**Conclusion** Protective ventilation combined with SV under ECMO by using a specific protocol based on BiPAP-APRV is feasible and safe. It may facilitate weaning and thus reduce the time under MV after ECMO. To what extend this beneficial effect is directly due to the presence of SV deserve further investigations.

**Competing interests** None.

**References**

1. Intensive Care Med. 2013;39:2003–10.
2. Anesth Analg. 2009;109:1892–1900.

#### P213 Evolution of family refusal to organ donation from subject in a state of brain death

##### Nadia Harbouze^1^, A. M. Amine^1^, A. G. Olandzobo^1^, R. Cherkab^1^, C. Elkettani^1^, L. Barrou^1^

###### ^1^Anesthesiology and intensive care departmen, UNIVERSITY TEACHING HOSPITAL IBN RUSHD-CASABLANCA, Casablanca, Morocco

####### **Correspondence:** Nadia Harbouze - nadialina_@hotmail.com

*Annals of Intensive Care* 2017, **7**(**Suppl 1**):P213

**Introduction** Since the first transplant from a patient in a state of brain death conducted in 2010 at the University Teaching Hospital IBN RUSHD of Casablanca, the number of transplants has increased. However, it is still inadequate meet the growing needs of organs. The refusal of families remains the main obstacle to the developpement of organ transplantation in Morocco. The aim of our study is to monitor and analyse the evolution of family refusal to organ donation in a brain dead patient.

**Patients and methods** This is a retrospective and comparative study from August until December 2014.The data were collected from records of brain dead patients candidates for organ donation at the 7 intensive care units on IBN RUSHED Hospital. The coordination registers were also studied. A questionnaire was distributed to families who refused organ donation to investigate the causes of the refusal.

**Results** During this period, 79 patients with brain death have been identified and 78 families had been approached. 61 families (78%) refused organ donation. The main causes of refusal were: fear of body mutilation (35%), lack of will (24%) and religious causes in 28% of cases.

The refusal rate for families decreased from 85% in 2010 to 50% in 2014. Only 2 patients experienced cardiac arrest before transplantation. During this period, 22 cornea transplants from brain-dead patient were conducted with 20 kidney transplants and two liver transplants.

**Discussion** The evolution of the refusal of families saw a decline through awareness and communication campaigns for organ donation.

**Conclusion** Improvements to our health care system must be proposed including strengthening detection of potential donors and relationships with the donor’s family and effective communication policy.

**Competing interests** None.

**Reference**

1. Le Nobin J, et al. Organ donation, reasons for family refusal: a retrospective study in a French organ harvesting center. Progrès en Urologie. 2014;24(5):282–87.

#### P215 Thank-you letters from relatives in the ICU: a qualitative analysis with regards to the patient–relatives–caregiver relationship

##### Alexandre Herbland^1^, Marie Richard^2^, Nicolas Girard^3^, Lucile Lambron^1^, Olivier Lesieur^1^

###### ^1^Réanimation, Centre Hospitalier la Rochelle, La Rochelle, France; ^2^Clinical psychology department, Centre Hospitalier la Rochelle, La Rochelle, France; ^3^Clinical research unit, Centre Hospitalier la Rochelle, La Rochelle, France

####### **Correspondence:** Alexandre Herbland - alexandre.herbland@orange.fr

*Annals of Intensive Care* 2017, **7**(**Suppl 1**):P215

**Introduction** In the ICU, three major actors are involved in the caring relationship: patient, relatives and caregivers. Acting as spontaneous testimonials of the lived experience, thank-you letters from relatives may be considered by ICU teams as a source of original information which could help in improving care for critically ill patients and families. This study aimed to investigate the qualitative content of thank-you letters from relatives of patients who stayed in the ICU. Specifically, our research questions were, with regards to the letters’ content, (1) how is the caring relationship tackled and characterized by relatives? (2) to what extent does this relationship impact their experience of ICU?

**Materials and methods** The study took place in a 16-beds ICU during a 6-month period. The research team consisted in a care assistant, a nurse (also clinical research associate), a psychologist (not working in the ICU) and an intensivist. The corpus consisted in twenty thank-you letters received in the ICU. We conducted a qualitative study according to the thematic inductive approach. The process of coding was intended to create established meaningful patterns.

**Results** Two main themes emerged as specific determinants of the caring relationship: (1) the temporality, comprising the time dedicated to the patients and their family, the time spent with the ICU team, the striking time corresponding to significant events for relatives needed to be shared with the staff, the extension of the link with caregivers by evocating a new life after ICU stay, the writing time as a counter-gift to the caregivers; (2) the caregivers behaviour, including human skills detailed in many core values (kindness, availability, devotion, attention, goodwill, sensitivity) psychological support, emotional sharing, capabilities to give informations. Relatives feel to be “at the center of all attention” in the same way as their loved ones.

Through the narration of ICU experience, the caring relationship is characterized as follows: (1) the caregiver becomes a close person with an equal relationship (feelings of friendship, emotional closeness); (2) the ICU team becomes a new family (contrasting with the poor living environment of ICUs); (3) the relative becomes a caregiver (with appropriation of medical terms or speaking of his loved one as a patient); (4) the caregiver is seen as a “super-hero” through an asymmetrical relationship with an overstatement of personal dedication and investment of the staff members (abnegation, vocation, involvement).

The caring relationship impacts relatives’ experience of intensive care in several ways: (1) relatives are deeply touched by caregivers’ human behavior, emotional support being a source of solace and resilience in particular for bereaved families; (2) relatives express the idea that taking care of humans is not a valued and rewarded task and the emerging awareness of hospital realities and difficulties of work in the ICU; (3) the most striking transformational change in relatives is the perception of their own vulnerability and humanity, leading them to exhibit an outward-looking attitude (for example filling out their organ-donation card), and encouraging the ICU caregivers to continue their missions for the others.

**Conclusion** Thank-you letters provide both encouraging and informative messages for ICU teams about relational care for patients and families notably the indivisibility of the families and their critically ill loved ones. The relatives’ experience of the ICU appears strongly influenced by the caring relationship in the way they express an authentic revelation of their own humanity and altruistic thoughts. The thematic content of thank-you letters questions determinants and fundamental values at stake in the patient–relatives–caregivers relationship.

**Competing interests** None.

#### P216 Advance directives and tomorrow’s doctors: perception and potential use

##### Sarah Wainschtein^1^, Sidonie Hubert^1^, Albane Hugues^1^, Marc Tran^2^, Kelly Tiercelet^2^, Mélanie Cherin^3^, Cédric Bruel^2^, Francois Philippart^2^

###### ^1^Médecine interne, Groupe Hospitalier Paris Saint-Joseph, Paris, France; ^2^Réanimation, Groupe Hospitalier Paris Saint-Joseph, Paris, France; ^3^Réanimation polyvalente adulte, Centre Hospitalier Intercommunal André Grégoire, Montreuil, France

####### **Correspondence:** Francois Philippart - fphilippart@gmail.com

*Annals of Intensive Care* 2017, **7**(**Suppl 1**):P216

**Introduction** Far from medical paternalism, the doctor-patient relationship has now evolved to respect “the autonomy and patients’ rights”. Changing behavior has been gradual, while the law offered the patient the freedom to consent to care and then of expressing their wishes regarding the therapeutic intensity they would benefit, in critical situations where consent would not be possible, through advance directives (AD) [1]. Their use is of paramount interest for intensivist in many critical situations. Unfortunately, the use of AD remains marginal because of the unfamiliarity of patients with their use and an appropriation default by clinicians [2]. The aim of our study was to investigate the perspective of the coming family physician generation on advances directives.

**Patients and methods** Population of interest was general practitioner fellow (GPF) from class of 2012 to 2014. We built an online questionnaire survey about knowledge and the place they want to give to AD in their forthcoming daily clinical activity. This questionnaire was sent to GPF emails obtained by universities, unions and via the official mailing lists of different regionals classes provided by the first contacted.

Descriptive analysis of quantitative data was expressed as mean and standard deviation, qualitative data in number and percentage. The comparison of continuous variables was performed by the Student t-test and the comparison of categorical variables by a Chi2 test. Analyzes were conducted on BiostaTGV website and Microsoft Excel^®^.

**Results** 2310 GPF answered the survey, mainly from Ile de France (n = 218), Toulouse (n = 199) and Lille (n = 169). For GPF the majority of patients do not know the AD (81.6%) and 54% think that those who know do not know how to use it.

88.4% of GPF think writing AD by patients requires better information. According to them, the information should concern the support offered in the ICU (77.1%), the use of mechanical ventilation (58.7%), dialysis (48.2%) and the evolution of patients after hospitalization in ICU (50.6%). Nevertheless information on the prognosis of chronic diseases or organ failure seems interesting for only 37 and 20.7% of them respectively.

89.8% of GPF wish to propose the drafting of AD to their patients. However, only 60.1% of them are willing to suggest AD to patients with cancer or hematologic malignancies, 56.2% to patients with neurological and/or degenerative disorders, 20.5% to elderly patients.

**Discussion** Despite the low proportion of the population we think these observations to be of interest because we probably selected the GPF the most interested in AD as the participation was not mandatory.

**Conclusion** A large majority of young of future general practitioner is willing to be involved in the implementation of AD with their patients, however the target population remains very limited, considering that half of them do not want to discuss AD with patients suffering from diseases potentially associated with ICU admission or therapeutic intensity discussion.

**Competing interests** None.

**References**

1. Leonetti J. LOI n° 2005–370 du 22 avril 2005 relative aux droits des malades et à la fin de vie. In; 2005.
2. Comité consultatif national d’ethique C. Fin de vie, autonomie de la personne, volonté de mourir. 2013.

#### P217 Reducing futility in the ICU: practice makes perfect

##### Olivier Lesieur^1^, Philippe Bouillard^1^, Nicolas Girard^1^, Vlad Loteanu^1^, Alexandre Herbland^1^, Maxime Leloup^1^

###### ^1^Réanimation, Groupement Hospitalier La Rochelle Ré Aunis, La Rochelle, France

####### **Correspondence:** Olivier Lesieur - olivier.lesieur@gmail.com

*Annals of Intensive Care* 2017, **7**(**Suppl 1**):P217

**Introduction** Substantial variability exists between countries, institutions, and physicians in the decision to withhold or withdraw (WhWd) life-sustaining treatment in critically ill patients. Moreover practices and skills may co-evolve over time. This epidemiological study aims to compare the procedural features of foregoing treatments between two separate periods at a 4-year interval (2012 vs 2016).

**Patients and methods** For each of the two periods considered, the characteristics and outcome of patients qualified for a WhWd procedure were collected in a single 16-bed ICU.

**Results** A total of 199 patients (70 ± 13 years, SAPS II 67 ± 22, sex ratio M/F 2.2) were qualified for a WhWd procedure in the past (84 patients) and current (115 patients) periods of the study. Differences (CI 95%) or Odds ratio (CI 95%) are given in Table 6 only in case of significant difference observed.

**Table 6:** characteristics and outcome of patients admitted over the study periods (2012 vs 2016)

Study period ICU admissions	2012 N = 596	2016 N = 403	Odds ratio (CI 95%)
Length of the period (months)	12	8	
Mean Age (yrs.)	65	65	
Mean SAPS II	54	53	
Mean LOS (days)	10.5	9.5	
Mortality rate (%)	28	29	
Sex ratio M/F	1.6	1.8	
Rate of patients qualified for a withholding or withdrawal (WhWd) procedure (%)	14	29	2.4 (1.8–3.3)
Brain deceased patients (N)	25	19	
Circulatory deceased patients (N)	143	97	
Rate of WhWd patients among circulatory deceased patients (%)	53	87	5.7 (2.9–11.1)
Rate of WhWd patients ultimately discharged alive from the ICU (%)	10	27	3.5 (1.5–8.1)
Rate of unexpected deaths^a^ among circulatory deceased patients (%)	47	13	0.18 (0.09–0.34)

*SAPS* Simplified Acute Physiology Score, *LOS* length of stay, *WhWd* withholding or withdrawal

^a^Unexpected death: i.e. without previous do-not-resuscitate order

**Discussion** At a 4-year interval, the proportion of patients dying in the ICU with a previous instruction “not to resuscitate” substantially increases, such as that of patients discharged alive after a formal WhWd decision (p < 0.0001). Correlatively, the rate of unexpected deaths declines. The overall death rate remains unchanged. The decrease in average length of stay is not statistically significant (p = 0.22).

**Conclusion** By anticipating unavoidable deaths, our efforts to withhold or withdraw hopeless treatments in critically ill patients have resulted in a lower rate of failed resuscitations without any change in global mortality.

**Competing interests** None.

#### P218 The specificity of stress factors in intensive care units (ICU)

##### Alexandra Laurent^1^, Florent Lheureux^1^, Alessia Prestifilippo^2^, Martin Delgado Maria Cruz^3^, Rigal Romain^4^, Massimo Antonelli^5^, Torra Lluis Blanch^3^, Franck Bonnetain^6^, Maria Grazzia-Bocci^3^, Jordi Mancebo^7^, Emmanuel Samain^8^, Hebert Paul^4^, Gilles Capellier^9^

###### ^1^Psychology, University of Bourgogne Franche-Comté, Besançon, France; ^2^Icu, Policlinico A. Gemelli, Rome, Italy; ^3^Icu, Hospital de Sabadell, Madrid, Spain; ^4^ICU, CHUM, Montréal, Canada; ^5^ICU, Policlinico Universitario A. Gemelli, Università Cattolica Del Sacro Cuore, Rome, Italy; ^6^Unité de méthodologie et de qualité de vie en cancérologie, CHRU de Besançon, Boulevard Alexandre Fleming, Besançon, France, Besançon, France; ^7^Réanimation polyvalente, Hospitat Sant Pau, Barcelone, Barcelone, Spain; ^8^Réanimation chirurgicale, CHU de Besançon, Besançon, France; ^9^Réanimation Médicale, CHU de Besançon, Besançon, France

####### **Correspondence:** Alexandra Laurent - alexandra.laurent@univ-fcomte.fr

*Annals of Intensive Care* 2017, **7**(**Suppl 1**):P218

**Introduction** Many studies have ever been conducted on the stress factors in health care settings. These studies have led to the development of stress scales intended for physicians and nurses (Bonneterre, 2008). However, the serious pathological and unpredictable states of patients in ICU require specific work and confront ICU professionals with specific stress factors. In this context, the question is to know if ICUs need a specific tool to identified stress factors.

**Patients and methods** This study aims to identify stress factors to the ICU and compare with the stress factors of the scale used in the international studies (Nursing Stress Scale (NSS), Nursing Work Index and Derivates, Karasek’s Questionnaire, Effort-Reward Imbalance Questionnaire, Job stress scale, The Source of Occupational Stress Scale, Health Professions Stress Inventory…).

This study was conducted in adult intensive care units in public or private hospitals in four countries: Canada, France, Italy, Spain. In each country, 40 health care professionals were solicited for an exploratory interview about the sources of stress in the work environment: 10 senior physicians, 10 residents, 10 experienced nurses (with more than 2 years of experience in the service) and 10 inexperienced nurses (with less than 2 years of experience in the service). All the interview transcripts were analysed using an inductive coding approach.

**Results** One hundred and sixty professionals (80 physicians and 80 nurses) were included in the study. Eight themes emerged from the analysis, and they concerned the stress linked to (1) patient (2) care, (3) team, (4) family, (5) institutional context, (6) environment, (7) organizational context, (8) individual dimensions. In each theme, sub-themes have been identified and determine more precisely the difficulties at work.

**Discussion** Our findings emphasize the complexity of work in ICUs and show the specifics factors not taken into account in the generic stress scales such as stress in relation with family relationships, the end of life decisions and inequity of health care.

**Conclusion** The specific stress scale should allow to better identified stress in ICU and to develop measures of prevention and support and training programs.

**Competing interests** None.

**Reference**

1. Bonneterre V, Liaudy S, Chatellier G, Lang T, de Gaudemaris R. Reliability, validity, and health issues arising from questionnaires used to measure Psychosocial and Organizational Work Factors (POWFs) among hospital nurses: a critical review. J Nurs Measur. 2008;16(3):207–30.

#### P219 Anxiety and depression in critial care staff of the university hospital of Monastir, Tunisia

##### Fahmi Dachraoui^1^, Abdelwaheb M’ghirbi^1^, Ali Adhieb^1^, Sabrine Nakkaa^1^, Kmar Hraiech^1^, Ali Ousji^1^, Dhouha Ben Braiek^1^, Saousen Ben Abdallah^1^, Hammouda Zaineb^1^, Islem Ouanes^1^, Lamia Ouanes-Besbes^1^, Fekri Abroug^1^

###### ^1^Réanimation polyvalente, CHU Fatouma Bourguiba, Monastir, Tunisia

####### **Correspondence:** Fahmi Dachraoui - dachraoui.fahmi@gmail.com

*Annals of Intensive Care* 2017, **7**(**Suppl 1**):P219

**Introduction** Intensive care units (ICU) is a place where caregivers face many constraints that can affect their physical and mental health due to the use of specific care and strong emotional charge related to patient death and pain of the families. The aim of the present study is to detect anxiety disorders and/or depression among staff working in ICUs.

**Materials and methods** On September 2016, a questionnaire was distributed to staff (medical and paramedical) operating in 5 ICUs in the university hospital Fattouma Bourguiba Monastir, Tunisia (1 medical ICU, 1 surgical ICU, 2 cardiologic CCUs and 1 nephrologic intermediate care unit). This questionnaire included demographic data of participants (age, sex, marital status, length of service, psychiatric history, consumption of anxiolytic and/or antidepressant) and the Hospital Anxiety and Depression Scale (HAD: scale composed by 14 items to screen the anxiety (A) and/or depression (D) among hospital staff).

**Results** During the study period, 106 participants completed the questionnaire (82%), 58% of them were women, the median age was 32 years ± 8.4. Forty-nine participants were doctors (the majority of them residents: 41/49). 12.3% of participants (all paramedics) worked on night shift, seniority of more than a year in the ICU was found in 59% of participants. 53.8% of staff interviewed were married and only 14.2% of them reported consumption of anxiolytics and/or antidepressants. 65.9 and 63.2% of the participants had respectively symptoms suggesting anxiety and depression. The median HAD score was 19 (IQR = 8); The medical function seems to be significantly associated with the occurrence of symptoms of anxiety and depression compared to paramedics, however the type of ICU (medical/surgical ICUs vs cordiologic/nephrologic ICUs) does not appear to be related to the occurrence of symptoms of anxiety or depression (Table 7).

**Table 7:** Relationship between the type of ICU, the function and the occurrence of symptoms of anxiety or depression in ICUs

	Anxiety, n (%)	Depression, n (%)
Medical/surgical versus cardiologic/nephrologic ICU	36 (70%) versus 36 (65.4%); p = 0.54	40 (78%) versus 37 (67.2%); p = 0.13
Medical versus Paramédical	47 (83.9%) versus 25 (51%); p = 0.001	46 (82%) versus 20 (41%); p < 0.001

**Conclusion** Anxiety and depression are common symptoms among caregivers in ICUs. Improved conditions of work in these units should be a target to avoid Burn Out Syndrome.

**Competing interests** None.

#### P220 Life-threatening 3,4-methylenedioxy-methamphetamine (MDMA) poisoning: clinical features and prognostic value of MDMA and its major metabolite concentrations on admission

##### Taissa Zavgorodniaia^1^, Marion Soichot^2^, Isabelle Malissin^1^, Sebastian Voicu^1^, Pierre Garçon^1^, Antoine Goury^1^, Lamia Kerdjana^1^, Nicolas Deye^1^, Emmanuel Bourgogne^2^, Bruno Megarbane^1^

###### ^1^Department of medical and toxicological critical care, Lariboisière Hospital, Paris, France; ^2^Laboratory of toxicology, Lariboisière Hospital, Paris, France

####### **Correspondence:** Bruno Megarbane - bruno.megarbane@aphp.fr

*Annals of Intensive Care* 2017, **7**(**Suppl 1**):P220

**Introduction** The 3, 4-methylenedioxy-methamphetamine (called MDMA) is a widely consumed recreational amphetamine derivate. The objectives of our study were to describe the features of life-threatening MDMA poisonings admitted to the intensive care unit (ICU) and to investigate the prognosticators on admission including the plasma concentrations of MDMA and its main active metabolite, the 3, 4-methyldioxyamphétamine (MDA).

**Patients and methods** We conducted a retrospective single centre observational study including all MDMA-poisoned patients admitted to the ICU from 2007 to 2016. Plasma MDMA and MDA concentrations were determined using high-performance liquid chromatography coupled to mass spectrometry. Comparisons were performed using Chi-2 and Mann–Whitney tests. Pharmacokinetics was modeled using PKsolver^®^ software and the usual parameters calculated.

**Results** Twenty patients (16M/4F; age: 25 years [21; 36] (median [25; 75 percentiles]; poly-intoxications: 80%) were included. On admission, patients presented marked consciousness impairment (Glasgow Score: 3 [3; 14]), hyperthermia (45%; 37.6 °C [37.3; 39.4].), serotonin syndrome (35%) and seizures (25%). Three patients (15%) experienced pre-hospital cardiac arrest. Management mainly consisted in the administration of adequate supportive care: massive fluid repletion (60%), mechanical ventilation (55%; duration: 1 day [1; 4]), sedative drugs (50%), external cooling (40%), catecholamines (35%), defibrillation (20%), massive transfusions (20%) and arterio-venous ECMO (20%). Cyproheptadine, the antidote of the serotonin syndrome, was administered to 20% of the patients. During the ICU stay, the following complications were observed including aspiration pneumonia (40%), cardiovascular failure (30%), disseminated intravascular coagulation (30%), acute renal failure (30%), liver failure (30%), rhabdomyolysis (25%), hemorrhages (20%), ventilator-acquired pneumonia (20%) and nerve/vessel compressions (10%). Five patients (25%) died in the ICU and among the survivors, one patient developed significant neurological sequellae. Based on a univariate analysis, pre-hospital cardiac arrest onset (p = 0.009), massive hyperlactatemia (p = 0.01) and marked coagulation disturbances (p = 0.004) on ICU admission were associated to death. Plasma MDMA (638 ng/mL [167; 989]) and MDA concentrations (25 ng/mL [17; 39]) on admission did not significantly differ according to the final outcome. The pharmacokinetic analysis showed that all the patients were in the elimination phase when admitted to the ICU, with prolonged MDMA and MDA half-lives in relation to the liver and renal failures and possibly to the elevated ingested doses of MDMA too.

**Conclusion** MDMA poisoning may be life-threatening and even fatal despite optimal ICU management. Strengthening the information among the youth about the dangers of MDMA use still remains a public health necessity.

**Competing interests** None.

#### P221 Emergency carbon monoxide poisoning: clinical presentation and outcomes

##### Fatma Essafi^1^, Aymen M’rad^1^, Olfa Mejri^1^, Marwa Ben Hmida^1^, Hafedh Thabet^2^, Youssef Blel^1^, Nozha Brahmi^1^

###### ^1^Department of intensive care and toxicology, Centre d’Assistance Médicale Urgente, Tunis, Tunisia; ^2^Department of emergency, Centre d’Assistance Médicale Urgente, Tunis, Tunisia

####### **Correspondence:** A. M’rad - mrad.aymen@gmail.com

*Annals of Intensive Care* 2017, **7**(**Suppl 1**):P221

**Introduction** Carbon monoxide (CO) poisoning is one of the common causes of poisoning specially in the cold season, which leads to a significant morbidity and mortality.

**Patients and methods** We retrospectively reviewed the medical data of patients who presented to the toxicology emergency department with CO poisoning during January 2015 to March 2016. We analyzed patients’ characteristics, management, and outcomes.

**Results** A total of six hundred and sixty-six patients (523 female and 143 male), aged of 34 ± 14 years, were included; poisoning occurred between December and February in 75% of cases, secondary to an indoor heating system exposure in the majority of cases (89%). The estimated duration of exposure was 2.5 ± 2 h [0.5–13 h], with a mean carboxyhaemoglobin (COHb) level on arrival at 18.6 ± 17%.

Neurological changes were the most presenting symptoms including headache (n = 652, 98%), dizziness (n = 272, 40%), seizure (n = 15, 2.3%) and loss of consciousness (n = 81, 12.2%). Digestive disorders involving vomiting and nausea were observed in 32.1% (n = 214). One woman without cardiovascular risk factors developed non ST-segment elevation myocardial infarction complicated by lung edema. The majority of patients (n = 601, 90%) received normobaric oxygen during 6 h (n = 535) and 12 h (n = 66). Hyperbaric oxygen therapy was administered at 2.5 ATA during 1 h to 65 patients for neurological changes (n = 24), pregnancy (n = 22) and elevated COHb ≥ 25% (n = 19). Mechanical ventilation was required for 5 patients, and admission into intensive care unit in 39 patients (6%). Death occurred in 5 cases (0.7%).

**Conclusion** The carbon monoxide poisoning is a common reason for emergency department visits in winter. The physician should be aware of the serious neurological and cardiovascular complications, if symptomatic treatment and oxygen therapy regimens were not respected.

**Competing interests** None.

#### P222 Neuro-respiratory toxicity of baclofen in the rat: study of the concentrations/effects relationships and role of GABAergic receptors

##### Salma Tannous^1^, Lucie Chevillard^1^, Laurence Labat^1^, Patricia Risede^1^, Isabelle Malissin^2^, Bruno Megarbane^2^

###### ^1^Inserm u1144, Paris-Descartes University, Paris, France; ^2^Department of medical and toxicological critical care, Lariboisière Hospital, Paris, France

####### **Correspondence:** Bruno Megarbane - bruno.megarbane@aphp.fr

*Annals of Intensive Care* 2017, **7**(**Suppl 1**):P222

**Introduction** Baclofen, a GABA-B receptor agonist is used as muscle relaxant agent and recently for the treatment of alcohol dependence. The number of poisonings has significantly increased since this new indication. Clinical presentation of poisoning mainly includes sedation, hypotonia, respiratory depression and seizures. To characterize the neurorespiratory toxicity of this molecule at high doses, we aimed at investigating alterations in Sprague–Dawley rat ventilation and brain electrical activity after baclofen administration and studied their reversal by GABA-receptor antagonists.

**Materials and methods** Rat ventilation was investigated using plethysmography and arterial blood gas analysis while brain electrical activity was studied using EEG with one implanted frontal electrode. Three baclofen doses were used including 43.5 mg/kg (30% lethal dose-50%), 72.5 mg/kg (50%) and 116 mg/kg (80%). Baclofen concentrations were obtained using HPLC-MSMS assay. We modeled baclofen pharmacokinetics and analyzed the doses/effects and effects/concentrations relationships.

**Results** Baclofen induced early-onset and prolonged dose-dependent sedation (p = 0.0002), hypothermia (p = 0.004), EEG and respiratory depression (0.001) characterized by significant increase in the inspiratory (p = 0.0001) and expiratory times (p = 0.02). Significant increase in PaCO_2_ and decrease in arterial pH and PaO_2_ were observed at 116 mg/kg (p = 0.001), peaking at 240 min. EEG showed signal slowing, burst-suppression aspects and spikes peaking at 5–6 h post-injection without normalization at the end of the experiment at 24 h. We did reverse baclofen-induced decrease in tidal volume with saclofen (a GABA-B receptor antagonist) and interestingly no alteration of baclofen-induced respiratory depression was observed with flumazenil (a GABA-A receptor antagonist). Pharmacokinetic parameters of baclofen were obtained at the three doses and were dose-dependent. Significant but non-linear relationships were observed between baclofen-induced effects and concentrations.

**Conclusion** Baclofen causes dose-dependent neurorespiratory toxicity in rats. However, due to increased poisonings, its safety profile at high doses remains to be established in humans.

**Competing interests** None.

#### P223 Baclofen poisoning: an epidemiological retrospective study in intensive care unit

##### Messaouda Khelfa^1^, A M’rad^1^, Hana Fredj^1^, Youssef Blel^1^, Nozha Brahmi^1^

###### ^1^Department of intensive care and toxicology, Centre d’Assistance Médicale Urgente, Tunis, Tunisia

####### **Correspondence:** Youssef Blel - blelyoussef@yahoo.fr

*Annals of Intensive Care* 2017, **7**(**Suppl 1**):P223

**Introduction** Baclofen is commonly used as treatment of spasticity, and recently prescribed in treatment of alcohol withdrawal. It has spasmolytic action resulting from antagonistic activity at presynaptic GABA receptors of the spinal cord. Because it’s widely prescription, it became one of the principal agents of poisoning. We present a retrospective study that aims to report the clinical outcomes and management of twenty cases of baclofen poisoning requiring ICU admission.

**Patients and methods** Retrospective review of medical observations of patients admitted to our intensive care unit for baclofen poisoning.

**Results** Between January 2013 and June 2016, 20 patients were enrolled in this study. Mean age was 28.2 ± 11 years. Sex ratio was 0.53. Mean APACHE II scale was 8.7 ± 4. Mean IGSII scale was 22.35 ± 11. Poisoning was deliberate in 100% of cases. Mean ingested dose was 448.5 ± 349 mg. The majority of patients presented to the emergency room at 4.8 ± 5 h after ingestion. Digestive decontamination was performed in 9.6% (n = 2) of patients.

Clinical presentation was dominated by neurological symptoms; including coma (n = 15), hypotonia (n = 5), hyporeflexia (n = 5), agitation (n = 5), seizures (n = 3) and delirium in 1 case. Hemodynamic manifestations included bradycardia in 12 patients, three of them required atropine infusion. One patient presented with hypotension responding to vascular resuscitation.

Sixteen cases required mechanical ventilation. Aspiration pneumonia was noted in 6 cases. Mean duration of ventilation was 36.6 h ± 27. Mean hospital length of stay was 62 h ± 25. Complications included ventilation associated pneumonia in one case and moderate rhabdomyolysis in 3 cases. All patients evolved favorably. There is no correlation between coma and assumed ingested dose.

**Conclusion** Baclofen overdose causes mainly neurological effects and except for bradycardia cardiovascular effects were uncommon. Prognosis is good if full supportive care is administered properly.

**Competing interests** None.

#### P224 Baclofen overdose: a retrospective multicentric study

##### Maxime Léger^1^, Marion Brunet^2^, Gaël Le Roux^2^, David Boels^2^, Nicolas Lerolle^1^

###### ^1^Réanimation médicale, C.H.U. d’Angers, Angers, France; ^2^Centre antipoison, C.H.U. d’Angers, Angers, France

####### **Correspondence:** Maxime Léger - mxmleger@gmail.com

*Annals of Intensive Care* 2017, **7**(**Suppl 1**):P224

**Introduction** The lack of an effective treatment for the maintenance of abstinence from alcohol has led physicians to take an interest in baclofen. Beyond efficacy, safety of baclofen, prescribed in high doses, is a concern, especially in case of drug overdose. Indeed, patients with chronic alcohol abuse frequently develop psychiatric disorders, and are at risk of voluntary drug intoxications. Thus, we set up a retrospective study to describe morbidity and mortality associated with baclofen overdose.

**Patients and methods** A case was defined as exposure to baclofen in self-harm attempt with or without symptoms between January 2008 and December 2015. This study was based on data collected by the Poison Control Center (PCC) of Angers University Hospital during telephone responses to toxicological exposure and during patient follow-up, supplemented by reports from clinical staff in hospital. The mortality rate of baclofen poisoning cases was compared to the 31,859 non-baclofen voluntary drug poisoning cases declared to the PCC of Angers University Hospital over the same period. Baclofen elimination half-lives were calculated in patients with multiple samplings.

**Results** 190 cases (median age: 40 years; gender (female): 46.8%) of voluntary intoxications were reported, including two deaths diagnosed at first medical assessment. Over years, number of baclofen poisoning grew up from 8 cases in 2008 to 91 cases in 2015. 111 patients (59%) had GCS ≤12 at admission and 77 had GCS >12 (41%). Eighty patients required mechanical ventilation (42.6%). Neurological severity (GCS ≤ 12 or >12) was significantly associated with higher suspected ingested dose (400 [IQR 190–685 mg] vs 120 [IQR 80–300 mg], p < 0.0001) and higher baclofen blood concentration (3.24 mg/l [min–max = 0.05–14.82 mg/l] vs. 0.32 mg/l [min–max = 0–1.66 mg/l], p < 0.001). Seizures were frequently observed in patients with GCS < 12 (n = 24, 22% vs. n = 2, 3%, in patients with GCS >12, p < 0.001). Three patients died in the hospital (hospital mortality rate 1.6%, total mortality rate 2.6%). Non-baclofen cases had lower rate of endotracheal intubation (n = 1833, 6%, p < 0.001 for comparison) and mortality rate (n = 299, 0.1%, p = 0.02 for comparison).

Spontaneous baclofen elimination half-life was calculated in six patients with normal serum creatinine and was 5.57 h [min–max = 3.2–8.3 h]. In the five patients who underwent dialysis for toxicological reason without evidence of renal failure, baclofen half-life was 3.46 h [min–max = 2.4–4.43 h]. Comparison between these two data showed a significant difference (p = 0.03).

**Conclusion** Baclofen, prescribed in high doses, may lead to severe intoxications: self-poisonings frequently require endotracheal intubation and are associated with an increased risk of death. Dialysis decreases baclofen elimination half-time but clinical relevance of this difference could not be determined.

**Competing interests** None.

#### P225 Baclofen poisoning in the intensive care unit: clinical features and investigation of the relationships between the toxic encephalopathy and the plasma baclofen concentration

##### Souaad Farah^1^, Lucie Chevillard^2^, Hélène Amiel-Niemann^3^, Laurence Labat^2^, Isabelle Malissin^1^, Nathalie Kubis^3^, Xavier Declèves^2^, Bruno Megarbane^1^

###### ^1^Department of medical and toxicological critical care, Lariboisière Hospital, Paris, France; ^2^Inserm u1144, Paris-Descartes University, Paris, France; ^3^Department of physiological investigations, Lariboisière Hospital, Paris, France

####### **Correspondence:** Bruno Megarbane - bruno.megarbane@aphp.fr

*Annals of Intensive Care* 2017, **7**(**Suppl 1**):P225

**Introduction** Baclofen, a GABA-B receptor-agonist with muscle relaxant properties established since 1974, has been recently used at elevated doses to treat dependence to ethanol. The number of prescriptions has exponentially increased without an exact evaluation of its toxicity. We aimed to describe acute baclofen poisoning requiring intensive care unit (ICU) admission and study the relationships between the toxic encephalopathy and the plasma baclofen concentration.

**Patients and methods** We conducted a single-centre retrospective study including all baclofen-poisoned patients admitted to the ICU in 2013–2016. When requested by the clinical situation, repeated electroencephalograms and measurements of the plasma baclofen concentrations were performed. Toxic EEG encephalopathy on a scale of zero to five was graded according to the international rating system (Markand, 1984). Plasma baclofen concentration was determined using liquid chromatography coupled to mass spectrometry in tandem developed with a Quantum Ultra apparatus (Thermo Fisher Scientific) and electrospray source ionization in positive mode (limit of quantification: 5 ng/mL). Linear regression and Chi-2 or Mann–Whitney tests were used as requested for subgroup comparisons. Baclofen pharmacokinetics and the relationships between the toxic encephalopathy and the plasma baclofen concentration were modeled using WinNonlin software v.5.3 (Pharsight Corporation, CA).

**Results** Twenty-eight patients (17 M/11F; age: 41 years [32; 49] (median [25th; 75th percentiles]) were included. Poisoning was mainly multidrug ingestion (92%) by suicidal attempt (63%) occurring in chronic alcoholic patients (71%). The presumed ingested dose of baclofen was 210 mg [109; 460] and the initial plasma concentration 1425 ng/mL [206; 2298]. Poisoning features included coma (Glasgow coma score: 3 [3; 11]) with hypotonia (46%), seizures (39%), abnormal pupil diameter (75%), sinus bradycardia (39%), aspiration pneumonia (21%) and EEG disturbances (43%) including the presence of burst suppressions, spikes, spike-waves, background slowing and even prolonged isoelectric trace in one severely poisoned patient. Management was supportive including mechanical ventilation (54%) which duration was related to the plasma concentration of baclofen (r2 = 0.67). One patient was dialyzed. No patient died. Baclofen half-lives of elimination (7 h [4; 9]) were closed to the observed values reported at therapeutic doses. The relationship between baclofen-induced encephalopathy as a function of the baclofen concentrations was described using a sigmoidal Emax model.

**Conclusion** Baclofen poisoning may be life-threatening. Toxic encephalopathy is well-described with EEG and its grade correlated to the baclofen concentration. Prescribers should be aware of the dangers of baclofen which benefits to treat dependence to alcohol are still lacking.

**Competing interests** None.

#### P226 Outbreak of facio-troncular dystonia in central Africa due to counterfeatured drug with haloperidol

##### Nicoals Peyraux^1^, Frederic Baud^2^, Micaela Serafini^1^, Jean-Claude Alvarez^3^, Annette Heinzelman^4^

###### ^1^Geneva, Médecins Sans Frontières, Genève, Switzerland; ^2^SAMU de Paris, Réanimation polyvalente, Paris, France; ^3^Paris, Assistance Publique Hôpitaux de Paris, Paris, France; ^4^2, rue saint-sabin, Médecins Sans Frontières, Paris, France

####### **Correspondence:** Frederic Baud - baud.frederic@wanadoo.fr

*Annals of Intensive Care* 2017, **7**(**Suppl 1**):P226

**Introduction** During week 52, 2014, in the northeast of the Democratic Republic of Congo, an African area included in the “Meningitis belt of Africa” and in which malaria is endemic, patients with suspected but atypical meningitis were reported. In January 2015, Médecins sans Frontières (MSF) Geneva was approached by the Ministry of Health to support the outbreak.

**Patients and methods** At the scene, data were collected on individual chart file records. Thereafter, at MoH and MSF case-management sites, information for each patient was recorded in a standardized Excel line-list. Patients’ demographic characteristics, clinical features, and discharge were recorded in an epidemiological study. At per request of the attending physician, cerebrospinal fluid was analysed for evidence of meningitis and blood for malaria. Owing to the atypical clinical features, on the advice of the MSF referent toxicologist, urine and medicine samples were collected in symptomatic patients. Toxicological analysis included liquid chromatography-mass spectrometry and high performance liquid chromatography/UV spectrometry. The study was approved by the Ethical Committee of the Ministery of Health of the Republic Democratic du Congo, including treatment of adults and children with specific antidote.

**Results** Initial examination suggested that an illness other than bacterial meningitis was the cause of patients’ complaints. First hypothesis was meningitis receiving uncomplete dosage regimen of antibiotics. Thereafter owing to apparent loss of consciousness with abnormal eyes movements, non-tonico-clonic seizures were considered meanwhile. The ratio of individuals less 5 y-o to those equal to and greater was 33/67%. The male to female ratio was 46/53%. The mean duration of hospitalisation was 3.4 ± 0.8 days (extremes 1–10 days). Extrapyramidal syndrome predominant on the upper part of the body was noted by paediatrician neurologists who suggested considering a genetic disease. However, signs and symptoms were present in people from different families in different areas at the same time. The definitive diagnosis made on pictures and videos of children and adults and was facio-troncular dystonia resulting from drug-induced adverse effect. Four urine samples were collected in children and sent to a toxicological laboratory in France. All urine samples were positive for haloperidol meanwhile the other causes of facio-troncular dystonia were excluded, including other neuroleptics, metoclopramide, antidepressants, amodiaquine, anti-histaminic drugs, anti-epileptics, and cocaine. From January to August 2015, 1021 hospitalisations were recorded in 925 patients. Looking for the source of haloperidol showed that tablets sold as ‘diazepam’ and consumed by symptomatic patients contained haloperidol as the sole active pharmaceutical ingredient, suggesting that this large outbreak was due to haloperidol toxicity from falsified diazepam. Initial treatment was diazepam to relieve severe facio-troncular dystonia which was efficient but resulted in long-lasting sedation more especially in children. A dosage regimen using bipéridène administered by intravenous and oral route was refined to prevent adverse effects related to this anticholinergic agent used in children. The complete reversal of the facio-troncular dystonia was the antidotal evidence supporting the toxicological diagnostic. The mortality rate was less than 5% meanwhile the direct causal relationship with ADR is questionable. An epidemiological study, including toxicological analysis in controls in ongoing. Indeed, facio-troncular dystonia induced by haloperidol does not result from a drug overdose but is an ADR occurring in about 20% of patients treated with haloperidol. WHO is involved in the inquiry related to this counterfeature involving different countries. The cause of the error is presently under investigation.

**Discussion** This outbreak emphasizes the need to consider toxicity resulting from counterfeatured medicines when facing collective atypical signs and symptoms in countries with unrestricted access to medication with limited control of qualities of the medicinal drugs.

**Conclusion** Counterfeatured medicinal drug may result not only in poor efficacy but also in onset of unexpected outbreak of unknown diseases that should suggest a toxic origin.

**Competing interests** None.

#### P227 Dosage regimen of biperiden to treat haloperidol-induced severe facio-troncular dystonic syndrome in children

##### Frederic Baud^1^, Nicoals Peyraux^2^, Micaela Serafini^2^, Annette Heinzelman^3^

###### ^1^SAMU de ParisRéanimation polyvalente, Paris, France; ^2^Geneva, Médecins Sans Frontières, Genève, Switzerland; ^3^2, rue saint-sabin, Médecins Sans Frontières, Paris, France

####### **Correspondence:** Frederic Baud - baud.frederic@wanadoo.fr

*Annals of Intensive Care* 2017, **7**(**Suppl 1**):P227

**Introduction** In late 2014-early 2015, Médecins Sans Frontières (MSF) had to face an outbreak of severe facio-troncular dystonic syndrome (FTDS) in North-East Congo. This outbreak resulted from counterfeature of pills sold as diazepam. Toxicological analysis revealed one pill contained about 10 mg of haloperidol. FTDS induced by haloperidol does not result from a drug overdose but is an adverse drug reaction (ADR) occurring in about 20% of patients treated with haloperidol. Nine-hundred and twenty-five individuals were admitted in MSF structures for 1021 FTDS. The ratio of individuals less than 5 y-o and equal to or greater of age was 33/67%, including 35 (3.4%) of children less than 1 y-o. Initial treatment was based on diazepam which relieved FTDS but resulted in long-lasting sedation, preventing given any drug by the oral route. Owing to the definitive diagnosis, a shift to the use of a more specific antidote was chosen. Biperiden was selected as existing in the intravenous and oral form in the Swiss pharmacopea. The study was approved by the Ethical Committee of the Ministery of Health of the Republic Democratic du Congo.

**Patients and methods** As a whole, biperiden was used in 223 cases (84% of the total). Treated children presented with severe dystonia as evidenced by inability to cooperate and to swallow. Verbal informed consent was obtained from relatives. The dosage regimen to treat drug-induced dystonic syndrome in the Swiss pharmacopea is as follows: for parenteral use in children, intravenously or intramuscularly: 0.040 mg/kg or 1.2 mg/m^2^ BSA every 30, according to response and tolerance; a maximum of four doses per day should be used. The internal MSF recommendations for biperiden use in children were 0.01–0.05 mg/kg of body weight that might be repeated four times a day. Initially, biperiden administration was administered under medical supervision by the MSF referent at the scene.

**Results** There was no pediatric preparation of biperiden. Accordingly, the adult preparation was used in children. The preparation contained 5 mg of biperiden in one milliter of solvent. The initial planned dose for children of 1 y-o and less and those up to 5 y-o were 1 and 2 mg, respectively. The 5 mg (1 ml) of biperiden was diluted in 4 ml of saline resulting in a final dilution of 1 mg/ml. Six children were treated according this dosage regimen. However, the one 1 mg dose was either of limited efficacy while being associated in others of signs suggestive of ADR, including agitation, heart rate greater than 160 b/min, the upper limit for children aged of 1 y-o and less. Two children greater than 1 y-o presented severe abnormal behavior resulting in an attempt at escape. Owing to question about safety, the dosage regimen was changed, as follows: 5 mg (1 ml) of biperiden was diluted with 9 ml of saline resulting in a final dilution of 0.5 mg/ml. An initial dose of 0.5 mg was administered intravenously as a bolus dose. The effects were looked for over 15 min. In the absence of improvement in facial dystonia, a second bolus dose of 0.5 mg was administered, a third dose could be considered 15 min later if the FTDS did not resume. The cumulative initial dose should not be greater than 2 mg. In addition to the reversal of facial dystonia, the therapeutic effect of biperiden included the return of swallowing to normal allowing to give further doses of biperiden by the oral route for three days. The first oral dose was administered no less than 12 h after the last initial dose at a dose equal to the efficient initial cumulative dose. The following doses were halved every 12 h. No ADR related to biperiden were reported using this dosage regimen. The mean duration of hospitalisation was 3.4 ± 0.8 days.

**Discussion** The bioavailability of biperiden by the oral route is equal to 33%. Accordingly, the corresponding intravenous dose should be divided by a factor three. Dosage regimen of anticholinergic drugs in children are poorly documented. The dosage regimen recommended by the pharmacopea resulted in frequent and severe ADR. Titration of biperiden resulted in efficient and safe dosage.

**Conclusion** When biperiden administration is required by intravenous route in children of 5 y-o and less, biperiden should be administered intravenously and titred using bolus dose of 0.5 mg till the therapeutic effect is obtained.

**Competing interests** None.

#### P228 Acute poisoning with rodenticides in the intensive care unit

##### Hassen Ben Ghezala^1^, Salah Snouda^2^, Moez Kaddour^2^, Chiekh Imen Ben^3^

###### ^1^Réanimation Médicale, Hôpital Henri Mondor, Avenue du Maréchal de Lattre de Tassigny, Créteil, France, Créteil, France; ^2^Réanimation Médicale, Hopital regional zaghouan, faculté de médecine de Tunis, Zaghouan, Tunisia; ^3^Teaching department of emergency and intensive care, Regional hospital of Zaghouan, Zaghouan, Tunisia

####### **Correspondence:** Hassen Ben Ghezala - hassen.ghezala@gmail.com

*Annals of Intensive Care* 2017, **7**(**Suppl 1**):P228

**Introduction** Severe poisoning by rodenticides is frequent. It represents nearly 30% of patients admitted to the new intensive care unit (ICU) of the region. That is why we decided to perform this study. The aim of this work was to describe the epidemiology, clinical features and management of all patients admitted to our unit for acute poisoning with rodenticides.

**Patients and methods** It was a retrospective study performed in the year 2013 from January to December. The study included all patients admitted in the ICU for rodenticide poisoning.

**Results** 32 patients were enrolled in the study. Our patients were young with a mean age of 30 ± 2 years. Poisoning was more common in females (n = 24; 75%). The mean delay between rodenticide poisoning and first medical contact was about 2 ± 2 h in the cases where this information. Most of our patients (91%) attended the emergency department of Zaghouan with a non-medical transportation. It was a suicide attempt in most cases (62%) and an accidental poisoning in 32% of patients. The most frequent cause of poisoning in our study was organophosphorus pesticide (n = 24; 75%). The second cause was alpha-chloralose poisoning with seven cases (22%). One patient ingested accidentally an anticoagulant rodenticide. Most of patients had ingested (oral route) the rat poison (n = 23; 78%). Clinical examination found normal vital signs in ten cases (31%). Nine patients (28%) had a shock, eight patients (25%) had an acute metabolic disorder and five patients (16%) had acute respiratory failure or were comatose. All patients enrolled in the study were admitted in the ICU for a period of clinical observation of 24 h. Stomach pumping (gastric lavage) was performed in 30 patients (93%). An antidote which was atropine was needed in twelve patients. Three patients (9%) who ingested alpha-chloralose needed intubation and mechanical ventilation. All patients had a good outcome and were discharged from ICU and from hospital. The mean ICU length of stay was 2 ± 3 days.

**Conclusion** This is the first study of acute poisoning with rodenticides admitted in the new ICU. The results of our study were similar to those published in recent literature. Cases of acute poisoning with rodenticides reported in this work were not severe.

**Competing interests** None.

#### P229 Hemodynamic correlates of the left ventricular mean ejection pressure: a carotid tonometric study

##### Mathieu Jozwiak^1^, Sandrine Millasseau^2^, Jean-Louis Teboul^1^, Jean-Emmanuel Alphonsine^1^, François Depret^1^, Nathalie Richard^2^, Pierre Attal^3^, Christian Richard^1^, Xavier Monnet^1^, Denis Chemla^4^

###### ^1^Service de réanimation médicale, inserm umr s_999, université paris-sud, Hôpital de bicêtre, hôpitaux universitaires paris-sud, Assistance publique – Hôpitaux de Paris, Le Kremlin-Bicêtre, France; ^2^Alam medical, ALAM Medical, Vincennes, France; ^3^Otolaryngology-head and neck surgery, Shaare-Zedek Medical Center and Hebrew University Medical School, Jerusalem, Israel; ^4^Service de physiologie, inserm umr s_999, université paris-sud, Hôpital de bicêtre, hôpitaux universitaires paris-sud, Assistance publique – Hôpitaux de Paris, Le Kremlin-Bicêtre, France

####### **Correspondence:** Mathieu Jozwiak - mathieu.jozwiak@aphp.fr

*Annals of Intensive Care* 2017, **7**(**Suppl 1**):P229

**Introduction** The systemic arterial load imposed to the left ventricle (LV) is a major determinant of normal/abnormal cardiovascular function. The LV mean ejection pressure (LVMEP) is the best estimate of load faced by the LV throughout ejection. The contribution of the steady and pulsatile blood pressure (BP) component of arterial load to LVMEP is debated. We studied the hemodynamic correlates of LVMEP using carotid tonometry. Intensive care unit patients equipped with an indwelling catheter were studied, thus allowing precise calibration of the tonometer.

**Patients and methods** Carotid tonometry (Complior Analyse^®^ ALAM Medical, France) was prospectively performed on 28 hemodynamically stable, spontaneously breathing patients (12F, mean age ± SD = 64 ± 18 years). Carotid waveforms were calibrated from diastolic BP and time-averaged mean BP invasively obtained at the radial (n = 18) and femoral (n = 10) artery. All patients were free of aortic stenosis. LVMEP was the area under the systolic part of the carotid pressure waveform divided by ejection time.

**Results** LVMEP (111 ± 17 mmHg) was strongly related to central systolic BP (126 ± 21 mmHg; r^2^ = 0.97) and was also related to mean BP (r^2^ = 0.82), peripheral systolic BP (r^2^ = 0.83), peripheral (r^2^ = 0.35) and central (r^2^ = 0.50) pulse pressure (each P < 0.05). The LVEMP was not related to age, heart rate and stroke volume. Systolic pulse wave amplification ratio from carotid to periphery was 1.07 ± 0.08.

**Conclusion** LVMEP was most strongly related to central systolic BP, which combines the influences of the steady and pulsatile components of central arterial load (r^2^ = 0.97). LVMEP was less strongly related to peripheral systolic BP, which may be less informative given variable systolic pulse wave amplification across patients.

**Competing interests** None.

#### P230 Prognostic value of high-sensitivity troponin I in patients with septic shock

##### Ali Jendoubi^1^, Ahmed Abbes^1^, Salma Jerbi^1^, Wafa Khedhiri^1^, Hatem Necib^1^, Salma Ghedira^2^, Mohamed Houissa^2^

###### ^1^Anesthesia and Intensive Care, Charles Nicolle Teaching Hospital, Tunis, Tunisia; ^2^Intensive care, Charles Nicolle Hospital, Tunis, Tunisia

####### **Correspondence:** Ali Jendoubi - jendoubi_ali@yahoo.fr

*Annals of Intensive Care* 2017, **7**(**Suppl 1**):P230

**Introduction** Myocardial dysfunction is one of the main predictors of poor outcome in septic patients, with mortality rates next to 70%. Many pathological findings were found in the sepsis induced cardiomyopathy including myocardial ischemia, alterations in microcirculation and *proinflammatory cytokines.*

The aim of this study was to assess the prognostic value of a recently developed highly sensitive cardiac troponin I (hsTnI) assay in patients with septic shock.

**Patients and methods** We performed a prospective observational study in septic shock ICU patients within 72 h of admission. Exclusion criteria were age >18 years; pregnancy; post-cardiac arrest and brain-dead. HsTnI was measured soon after admission and 12, 24, 48 and 72 h after. Patients were subjected to transthoracic echocardiography (TTE) at study inclusion and regular biochemical and hemodynamic assessments were performed. Pearson’s Chi square and Fisher’s Exact tests were used. P < 0.05 was considered significant.

**Results** Thirty-three patients (M/F = 25/8) with septic shock were included in the study. Significant differences in demographic and biochemical (laboratory and metabolic) parameters, severity scores, life-support therapies (vasopressors, ventilation), and length of ICU stay were compared between survivors and non-survivors at 28 days. 15 patients (45.45%) showed an elevated HsTnI on admission time. The level of HsTnI at 72 h was significantly higher in non-survivors (n = 12) than survivors (n = 21) (median 69.6 [16.7–118.8] vs 9.6 [4.2–21.4] ng/L, P = 0.049). HsTnI level elevation was mainly correlated with lactate serum and hemodynamic parameters. Besides, the clearance of creatinine was significantly lower in non-survivors patients at 28 days (median 48.15 [27.5–121.5] vs 110 [90–135] ml/min, P = 0.022). Right heart abnormalities existed in non-survival patients but were not associated with elevated hsTnI. *Tricuspid Annular Peak* Systolic *Velocity* was significantly lower in non survivors than survivors (median 16 [13–16.1] vs 17 [15.75–24] cm/s, P = 0.042).

**Conclusion** Circulating hs-cTnI is present in patients with septic shock. A rise of HsTnI may be an indicator of poor outcome. Also, right heart functional abnormalities exist in patients with septic shock.

**Competing interests** None.

#### P231 Evolution of the right distribution width as a pronostic marker during the differents state of shock

##### Paolo Scarfo^1^, Charles Chevalier^2^, Michael Piagnerelli^3^

###### ^1^Internal medicine, Hospital André Vésale, Montigny-le-Tilleul, Belgium; ^2^Biologie clinique, Hôpital Civil Marie Curie, Charleroi, Belgium; ^3^Réanimation polyvalente, Hôpital Civil Charleroi, Charleroi, Belgium

####### **Correspondence:** Paolo Scarfo - paoloscarfo@hotmail.com

*Annals of Intensive Care* 2017, **7**(**Suppl 1**):P231

**Introduction** Right distribution Width (RDW) has been recently proposed as a pronostic factor in different pathologic situations and especially to the septic patients who stay in ICU. Some works substantiate the relationship between an alteration of the red blood cell rheology during the septic shock and a severe state of the disease. No one has studied the RDW between the differents shocks yet.

We are going to determinate the relationship between RDW and APACHE II score, mortality rate in the Intensive Care Unit (ICU), at the hospital, at the day 30 and 90.

**Materials and methods** We investigated those parameters near 228 patients who were admitted at the ICU and needed norepinephrine between the first of March and the 31st of December.

They were stratified in différent groups: septic shock n = 101, cardiogenic shock n = 100, hemorragic shock n = 20 and obstructive shock n = 7.

**Results** We did not observe any correlation between the RDW and the ICU mortality, hospital mortality and at the day 30 and 90. Only a poor significant correlation has been found between the cardiogenic shock and the mortality rate: at the hospital (p = 0.01), at day 30 (p = 0.01) and at the day 90 (p = 0.05) but not in the ICU (p = 0.89). The Receiver Operating Characteristics (ROC) curves do not show significant differences between RDW, APACHE II score and ICU mortality rate or intra hospital.

The sample of the hemorrhagic shock and obstructive shock was not usable for this calculation. Compared to other studies which were focused on the septic shock where the mortality was approximately 20%, we determinated a mortality rate near 50%.

**Conclusion** The delta of the RDW D3/D1 did not present any correlation with the mortality rate. In our study, the RDW in the different kind of shocks do not look like to be a good predictive marker of the mortality, except for the patients included in the cardiogenic shock where a poor significant correlation could be highlighted.

**Competing interests** None.

**References**

1. Antonelou MH, Kriebardis AG, Papassideri IS. Aging and death signalling in mature red cells: from basic science to transfusion practice. Blood Transfus. 2010;8(Suppl 3):s39–47.
2. Demir A, Yarali N, Fisgin T, Duru F, Kara A. Most reliable indices in differentiation between thalassemia trait and iron deficiency anemia. Pediatr Int. 2002;44(6):612–6.

#### P232 Prognostic factors in patients hospitalized in the intensive care unit for complicated acute myocardial infarction

##### Alexandre Lafont^1^, Antoine Galy^2^, Thomas Daix^3^, Claire Mancia^2^, Nicolas Pichon^2^, Bruno François^3^, Antoine Vieillard-Baron^1^, Philippe Vignon^3^

###### ^1^Réanimation médico-chirurgicale, Assistance Publique - Hôpitaux de Paris, Hôpital Ambroise Paré, Boulogne-Billancourt, France; ^2^Service de réanimation polyvalente, Centre Hospitalier Universitaire de Limoges, Limoges, France; ^3^Inserm cic 1435/réanimation polyvalente, C.H.U de Limoges, Limoges, France

####### **Correspondence:** Philippe Vignon - sarah.demai@chu-limoges.fr

*Annals of Intensive Care* 2017, **7**(**Suppl 1**):P232

**Introduction** Prognostic factors in patients admitted to the Intensive Care Unit (ICU) with a complicated Acute Myocardial Infarction (AMI) have been scarcely described. The aim of our study was to determine independent factors of hospital death in this specific population.

**Patients and methods** This two-center retrospective study included all consecutive patients who were admitted to the ICU between January 2000 and December 2015 for a complicated AMI. Exclusion criteria were an inaugural cardiac arrest revealing AMI, a peri-operative AMI, and patients transferred to the ICU after emergency coronary artery bypass grafting (CABG) for AMI. Patients with ventricular arrhythmias responsible for transient circulatory arrest during their management, with rapid recovery of normal consciousness were studied. In each patient, comorbidities, SAPS2 score, SOFA score upon ICU admission, and AMI complications including cardiogenic shock, transient circulatory arrest, pulmonary edema and mechanical complications (i.e., ruptured papillary muscle, ventricular septal defect, tamponade) were recorded. Multivariate regression analysis was performed to determine independent factors of hospital death.

**Results** Among 358 patients admitted to the ICU with complicated AMI, 91 were excluded for inaugural cardiac arrest with sustained coma requiring therapeutic hypothermia (n = 60), emergency CABG for AMI (n = 17), or AMI after surgery (n = 43). Finally, 267 patients (1.4% of all admissions) were studied (180 men; mean age: 67 ± 12 years; SAPS2: 57 ± 21; SOFA: 8 ± 4), 82% of them being ventilated. AMI complications were: cardiogenic shock (n = 191), pulmonary edema (n = 124), transient circulatory arrest (n = 60), and mechanical complication (n = 29). Prompt coronary angiography was performed in 215 patients (81%) and angioplasty in 167 of them (78%). Coronary angiography was not performed in 52 patients for hemodynamic instability preventing patient transportation (n = 40) or expired time of potential revascularization (n = 12). Only two of these patients had cardiogenic shock. Hospital mortality reached 46% (n = 122). Non survivors were older, had higher severity scores, were more frequently under mechanical ventilation and catecholamines, had lower ejection fraction (27 ± 13 vs 37 ± 12%: p = 0.0004), and exhibited higher lactate (8.1 ± 5.1 vs 4.6 ± 3.3 mmol/l: p = 0.0001) and creatinine levels (178 ± 122 µmol/l vs 136 ± 103 µmol/l: p = 0.0027) than survivors. Independent prognostic factors were: SAPS2 score (OR 1.13 per point [95% CI 1.05–1.22; p = 0.002), serum creatinine >150 µmol/l upon admission (OR 1.82; 95% CI 1.0–3.32; p = 0.05), and cardiogenic shock (OR 3.37; 95% CI 1.72–3.32; p = 0.0004).

**Conclusion** Cardiogenic shock was the most frequent complication of AMI who led to ICU admission, whereas mechanical complications are rare at the era of early coronary reperfusion strategies. In addition to severity score, serum creatinine and cardiogenic shock appeared as independent factors of hospital death.

**Competing interests** None.

#### P233 Place in the thrombolysis embolism pulmonary high risk

##### Nadia Benatta^1^, Djamila-Djahida Batouche^2^, Amel Zerhouni^3^, Kheira Tabeliouna^2^, Setti-Aouicha Zelmat^4^

###### ^1^cardiologie, Centre Hospitalier et Universitaire d’Oran, Oran, Algeria; ^2^Réanimation pédiatrique, Centre Hospitalier et Universitaire d’Oran, Oran, Algeria; ^3^Reanimation, EHS CANASTEL, Oran, Algeria; ^4^Anesthésie réanimation chirurgicale, EHU 1er Novembre, Oran, Algeria

####### **Correspondence:** Djamila-Djahida Batouche - khedidjabatouche@yahoo.fr

*Annals of Intensive Care* 2017, **7**(**Suppl 1**):P233

**Introduction** Pulmonary embolism (PE) in high-risk is a partial or total obliteration of the pulmonary arterial network by a fibrin-clot cruoric more than 50%, the management requires a rapid reduction of pulmonary arterial resistance and right ventricular post load through rapid revascularization by thrombolysis. Our aim is to determine the value of thrombolysis in pulmonary embolism and describe the clinical, paraclinical and outcome pulmonary embolism at high risk.

**Patients and methods** This is a descriptive study of 20 cases of pulmonary embolism at high risk admitted to the cardiology department to CHU Oran between 2008 and 2014. Signs of gravity of (PE) comprising: syncope, circulatory collapse, cardiogenic shock or acute pulmonary sonographic sign of heart. It was confirmed in chest CT. All patients received thrombolysis using the protocol accelerated by two types of molecules: streptokinase or actilyse.

**Results** The sex ratio was 0.11; mean age 44 years, ranging from 20 to 80 years; Risk factors were dominated by contraception was 35% and the postoperative 30% the clinical picture was dominated by cardiogenic shock in 75% of cases. 20% cardiovascular collapse and syncope in 5%; Doppler echo all patients had signs of dysfunction of the right ventricle represented by the dilatation of the right cavities and pulmonary hypertension. The CTA found a (PE) bilateral in 70% right in 20%. Thrombolysis using actilyse in 12 patients and streptokinase in 8 cases.

The outcome was favorable in 16 patients; with two cases that are complicated by chronic pulmonary heart and the death of 2 patients with cancer.

**Discussion** The female predominance is explained by the increase of risk factors hormonal contraception, whose first generation combination hormonal. Our patient had a high probability with clinical signs of severity based on the score WELLS [1]. This diagnosis was confirmed by chest CT; which shows the vascular bed obstruction degree with a very good sensitivity and specificity.

The suspect patients with severe PE and that presented signs of acute pulmonary heart ultrasound have effectively (PE). The indication of thrombolysis was chosen on hemodynamic criteria; success is found in 80% of patients with improved hemodynamics dice the early hours. This success is explained by the role of thrombolytic in lysis clot to obtain pulmonary arterial revascularization; and reduce pulmonary arterial resistance and the right ventricular afterload which accelerates the healing of right heart failure and improvement of pulmonary capillary volume. The 02 cases who developed a chronic pulmonary heart; it was done immediately a right ventricular dysfunction with pulmonary arterial outset of very high pressures suggestive that the embolism occurred on an already pathological right heart. No cases of massive bleeding were noted in our series.

**Conclusion** Severe pulmonary embolism is burdened with high mortality; diagnosis is based on the stratification of risk score, was facilitated by the non-invasive strategies that articlent around the Doppler echocardiography and CT angiography; thrombolysis can reduce the high mortality related to severe pulmonary embolism.

**Competing interests** None.

**Reference**

1. Meyer G, Sanchez O. Stratification du risque dans l’embolie pulmonaire aiguë.Realites Cardiologiques • N° 219 - Cahier 1 • Septembre 2006.

#### P234 Hypertensive peaks of diabetics in the emergency

##### Fatma Kaaniche Medhioub^1^, Najla Ben Algia^2^, Rania Allela^3^, Samar Cherif^4^

###### ^1^Faculté de médecine de Sfax, Sfax, Tunisia; ^2^Intensive care, hopital régional Gafsa, Sfax, Tunisia; ^3^Hopital régional mahres, Faculté de médecine de Sfax, Sfax, Tunisia; ^4^Intensive care, hopital régional mahres, Sfax, Tunisia

####### **Correspondence:** Fatma Kaaniche Medhioub - fatma_kaaniche@yahoo.fr

*Annals of Intensive Care* 2017, **7**(**Suppl 1**):P234

**Introduction** Hypertension is a frequent motif for admission to emergencies. The diabetic is increasingly exposed to this risk [1]. The objective of this study is to evaluate the proportion of diabetic patients presenting to the emergency department with high blood pressure (BP) and to identify their epidemiological and clinical characteristics.

**Patients and methods** Prospective study conducted over a period of 3-month (1/01/2016–31/03/2016). After measuring vital signs in the emergency, patients included had a systolic BP ≥ 140 mmHg and/or diastolic BP ≥ 90 mmHg with diabetes. A BP control was performed 30 min after the inclusion. If hypertension persists, patients were admitted to cardiology or general medicine department.

**Results** We included 75 patients. Thirty-eight patients (50.6%) had persistent hypertension. The most common background were: dyslipidemia, coronary disease and kidney failure associated with diabetes and hypertension. The average age of patients was 58 ± 13 years. The sex ratio was 2.8. The average duration of diabetes was 8 ± 3.5 years. It was a type 2 diabetes in 64% of cases and type 1 in 36% of cases. Among the degenerative complications: coronary disease in 14.7%, arterial disease of the limbs (10.7%), stroke (5.3%), retinopathy (5.3%) and nephropathy (4%). All patients had at least one cardiovascular risk factor. Hypertension was classified: grade I (33%), grade II (45%), grade III (22%). The IEC was the antihypertensive most prescribed in our series with a monotherapy in 56% of cases, 24% in bitherapy and 20% in triple therapy.

**Conclusion** Hypertension is a disease frequently associated with diabetes. It worsens the prognosis of diabetics from where the need for optimal control of blood pressure and management of other risk factors.

**Competing interests** None.

**Reference**

1. Norman RCC, Guanmin Ch. Canadian efforts to prevent and control hypertension. Can J Cardiol. 2014;26:14C–17C.

#### P236 The value of Doppler ultrasound parameters of portal vein and hepatic artery in the prediction of liver dysfunction in patients with septic shock

##### Ali Jendoubi^1^, Ali Gaja^2^, Bassem Hamrouni^3^, Abir Malouch^1^, Sami Fourati^1^, Salma Jerbi^1^, Rihab Messaoud^1^, Salma Ghedira^3^, Mohamed Houissa^3^

###### ^1^Anesthesia and Intensive Care, Charles Nicolle Teaching Hospital, Tunis, Tunisia; ^2^Medical imaging department, Charles Nicolle Teaching Hospital, Tunis, Tunisia; ^3^Intensive care, Charles Nicolle Hospital, Tunis, Tunisia

####### **Correspondence:** Ali Jendoubi - jendoubi_ali@yahoo.fr

*Annals of Intensive Care* 2017, **7**(**Suppl 1**):P236

**Introduction** Sepsis associated liver dysfunction (SLD) is usually attributed to systemic and/or microcirculatory disturbance. Hypoxic hepatitis, also known as shock liver or ischemic hepatitis, is a life threatening event associated with high morbidity and mortality. Doppler ultrasonography is a non invasive method to measure Doppler hepatic hemodynamic parameters. The primary objective of this study was to assess the accuracy of the hepatic hemodynamic parameters (portal venous blood flow PVBF and resistance index of the hepatic artery HARI) in predicting SLD in septic shock patients. The secondary aims were to identify factors associated with SLD, investigate the effects of volume expansion (VE) on systemic and intrahepatic hemodynamics and to assess the intra- and interoperator reproducibility. We also analyzed 28-day mortality.

**Patients and methods** In a prospective design, we included 30 consecutive patients with septic shock (24 males; median age: 36.5 years) admitted to the ICU with septic shock in Charles Nicolle Hospital of Tunis from February to July 2015. All patients were resuscitated following the Surviving Sepsis Campaign guidelines. We measured systemic hemodynamic variables (Mean arterial pressure (MAP), and cardiac index (CI)) and performed hepatic Doppler before and after volume expansion. We measured PVBF and computed the HARI. We recorded the liver function tests (ALT, AST and Bilirubin) for 48 h. SLD was defined as an increase in serum Bilirubin ≥ 20 µmol/l (Hepatic SOFA ≥ 1). Accuracy of the hepatic hemodynamic parameters to predict SLD was measured by the area under the ROC curve. P < 0.05 was taken to indicate statistical significance.

**Results** The median SOFA score at T0 was 8 points and the median IGS2 score was 38 points. The sources of infection were as follows: the lungs (n = 19), the abdomen (n = 4) and the urinary tract (n = 3). The incidence of SLD in our cohort was 33.3% (n = 10). There was no significant difference between “SLD group” and “No-SLD group” in all hepatic hemodynamic parameters especially the PVBF and the HARI. Lactate levels were significantly higher in patients with SLD (median 3.55 vs. 0.85 mmol/l). Similarly, the platelet count was significantly lower in the “SLD group” [mean (± SD) 149.2 ± 103.9 (109/l) vs. 242.8 ± 104.1 (109/l); p = 0.039]. There was no difference in duration of mechanical ventilation, ICU length of stay and 28-day mortality between the 2 groups.

The PVBF was significantly lower in patients who died before D28 (median: 558 vs. 826 l/min in the survivors; p = 0.014).

Volume expansion caused a significant increase in CI, mean hepatic artery velocity and the PVBF.

The intra- and interoperator reproducibility was good to excellent for the systolic and mean velocities of the hepatic artery, portal vein diameter and the PVBF.

**Conclusion** Our results don’t support the hypothesis that the hepatic sonography is predictive of SLD in septic shock. Our pilot study showed higher lactate levels and hematologic SOFA in SLD group. The PVBF was significantly lower in patients who died before D28. More experience will be necessary to define the ultimate role of Doppler ultrasonography in the evaluation of hepatic perfusion in patients with septic shock.

**Competing interests** None.

#### P237 Postoperative care and risk factors after multiple valve surgery for advanced rheumatic heart disease in 150 patients

##### Youssef Zarrouki^1^, Amra Ziadi^1^, Manal Rhezali^1^, Zahira Zouizra^2^, Drissi Boumzebra^2^, Mohamed Abdennasser Samkaoui^1^

###### ^1^Anesthesiology and intensive care, caddi ayyad university Mohammed VI teaching hospital, Marrakesh, Morocco; ^2^Cardio vascular surgery, caddi ayyad university Mohammed VI teaching hospital, Marrakesh, Morocco

####### **Correspondence:** Youssef Zarrouki - zarroukiyoussef@hotmail.fr

*Annals of Intensive Care* 2017, **7**(**Suppl 1**):P237

**Introduction** Early surgery is the current trend for management of patients with valvular disease. That said many of them, particularly from developing countries, are still operated at a very advanced stage of disease. Despite improvements in myocardial protection and surgical techniques, postoperative care after multiple valve surgery (MVS) for advanced rheumatic heart disease (RHD) remains to be a clinical challenge. We conducted a study to determine postoperative complications and morbidity-mortality risk factors in this subgroup of patients.

**Patients and methods** Through a retrospective study over a period of 8 years, from June 2007 to October 2015, patients admitted in intensive care after aortic and mitral valve surgery combined with tricuspid repair for RHD, were included if at least one the following criteria is found. Criteria for selecting patients: exertional dyspnea grade III or IV of the NYHA; cardiomegaly; low left ventricular ejection fraction (LVEF); severe pulmonary hypertension (PHT) and or right ventricular dysfunction; deterioration of more than one vital function. Preoperative clinical and ultracardiosonographic features are assessed. Surgical difficulties are evaluated, as well as risk factors for occurrence of complications and causes of death.

**Results** 150 patients (out of 1025 admitted during the same period) are eligible to be included. Patients clinical profile and ultrasonographic data are summarized in Tables 8 and 9.

**Table 8:** Patients clinical profile

Age	37 years (21–64 years)
Gender	F: 81 (54%), M:69 (46%)
Medical history	Systemic hypertension: 21 (14%); diabetes: 18 (12%); Stroke: 17 (11.33%); acute lower limb ischemia: 4%; recurrent heart failure: 97 patients(64.6%); Hepatic dysfunction: 15 (10%); renal insufficiency: 18 (12%); infective endocarditis: 17 (11.33%); Prior mitral valve surgery: 9 (6%)
Symptoms	Exertional dyspnea III–IV: 123 (82%)
chest x-ray	Cardiomégaly with CTR > 0.7: 128 (85%)
ECG	Atrial fibrillation: 135 (90%)

**Table 9:** preoperative ultracardiosonographic data

Cause	Exclusively rheumatic advanced multiple valvulopathy
Impact of the Valvular disease	PHT: 70 mmHg (60–120 mmHg);RV dilatation with moderate to severe dysfunction: 68 (45.33%) with moderate to severe functional tricuspid regurgitation; LVEF: 42% (22–65%); Left Atrium dilatation: 54.6% + thrombus 12%; Ascending aortic aneurysm:2%

The surgical procedure consisted mostly in a double mitral and aortic valve replacement with tricuspid valve repair. Additional procedures are realized in 59 patients (39%).

The average ICU length of stay is 7 days (2–30 days) with a mean mechanical ventilation duration of 48 h (1–10 days) and an inotropic support in 100% of patients. Additive therapies like nitric oxyde is used in 35 patients and sildenafil in 42.

Early postoperative complications occured in 63 patients: low cardiac output Sd in 12 cases and acute right sided heart failure in 7 patients. Bleeding with redo in 10 patients. Infections are noted in 19 patients with 7 cases of septic shock. Acute kidney failure requiring dialysis in 5 patients. Dysarythmias and transient atrioventricular block are observed in 23 patients and serious neurologic events in 5 patients.

In hospital mortality is 18.6% (28 patients). Deceased patients profile is listed in Table 10.

**Table 10:** Deceased patients profile

Age	44 years old (30–64 years)
Symptoms	Exertional Dyspnea grade III- VI;HF renal insufficiency; Liver dysfunction; ascites; acute lung edema and refractory HF
Preoperative echocardiography findings	Severe PHT: 9 patients; severe mitral regurgitation caused by infective endocarditis with severe LV dysfunction: 1 case; Massive Ao regurgitation with severe LV dysfunction: 2 cases

Causes of death are: severe LV dysfunction (5 patients); severe RV dysfunction (15 patients); severe pneumonia (3 patients); septic chock (2 patients); stroke (1 patient); intraventricular hemorrhage (1 patient); fulminant hepatitis (1 patient).

**Discussion** In the European Registry of the Euro Heart Survey, patients with very advanced heart valvular disease accounted for 8% of all valvular heart disease. In our series, they represent 22%.

**Conclusion** The operative mortality was typically very high, but new surgical technologies and intensive care standards should improve outcome. Generally the postoperative mortality is very significantly lower than spontaneous mortality. Thus the indication of surgery should not be rejected in this subgroup of patients.

**Competing interests** None.

## .

### E-posters

#### S1 Extracorporeal life support for refractory cardiac arrest: a 10-year study

##### Jennifer Brunet^1^, Bertrand Canoville^2^, Pierre Verrier^1^, Aurélie Joret^2^, Calin Ivascau^3^, Amélie Seguin^2^, Xavier Valette^2^, Damien Du Cheyron^2^, Cedric Daubin^2^

###### ^1^Anesthesiology, Centre Hospitalier Universitaire de Caen, Caen, France; ^2^Medical intensive care, Centre Hospitalier Universitaire de Caen, Caen, France; ^3^Thoracic and cardiovascular surgery, Centre Hospitalier Universitaire de Caen, Caen, France

####### **Correspondence:** Cedric Daubin - daubin-c@chu-caen.fr

*Annals of Intensive Care* 2017, **7**(**Suppl 1**):S1

**Introduction** We aimed to identify factors associated with ICU survival among selected patients receiving Extra Corporeal Life Support (ECLS) for in-hospital or out-of-hospital refractory cardiac arrest.

**Patients and methods** All consecutive patients treated with ECLS for refractory cardiac arrest in the Caen University Hospital in northwestern France over the last decade (2005–2015) were included in a retrospective cohort study.

**Results** Sixty-two patients were included: 25 with out-of-hospital refractory cardiac arrest and 37 with in-hospital refractory arrest. The initial rhythms was shockable rhythm in 25 (40%) cases. At ECLS initiation, the mean no flow was 0.8 ± 3.9 min and mean low flow (time between the time of refractory cardiac arrest and time at which an ECLS flow was provided) was 71 ± 37 min. The mean ECLS flow rate was 4.17 ± 1.31 L/min. Initial blood test results were: arterial pH = 7.04 ± 0.20 and plasma lactate = 12.8 ± 5.9 mmol/L.

Eleven (18%) patients survived (4/30 (13%) acute coronary syndrome, 4/8 (50%) severe poisoning due to drug intoxication, 1/4 (25%) dilated cardiomyopathy, and 2/20 (10%) others). Survival was lower for patients with out-of-hospital refractory cardiac arrest, 2 of 25 (8%), than for patients with in-hospital refractory cardiac arrest, 9 of 37 (24%), respectively, *p* = 0.17. As expected, out-of-hospital refractory cardiac arrest was associated with a more prolonged low flow (89 ± 40 min vs 60 ± 30 min, *p* < 0.01) and a more profound acidosis (pH 6.9 ± 0.2 vs 7.1 ± 0.2, *p* = 0.01 and arterial lactate 14.7 ± 6.1 vs 11 ± 5, *p* = 0.05).

In univariate analysis, survival was lower for patient with refractory cardiac arrest unrelated to drug intoxication, 13 vs 50%, respectively, *p* = 0.03. In addition, mortality was associated with arterial pH (7.01 ± 0.2 vs 7.14 ± 0.15, *p* = 0.025) and low flow (75 ± 40 vs 55 ± 12 min, *p* = 0.005).In multivariate analysis, two factors was associated with survival: drug intoxication as the reason for ECLS [Adjusted Odds Ratio, 8.34; 95% CI, 1.25–71; *p* = 0.01], and low flow [Adjusted Odds Ratio, 0.98; 95% CI, 0.95–1; *p* = 0.14].

**Conclusion** In a highly selected group of critically ill patients with refractory cardiac arrest, the potential beneficial effect of ECLS could be due only to its clinical impact on reversible causes of circulatory failure (i.e. severe drug intoxication in our cohort). Further studies are needed to clarify whether the use of ECLS could be considered as a disproportionate tool, specifically in patients with out-of-hospital refractory cardiac arrest due to acute coronary syndrome or associated with prolonged low flow or a profound acidosis.

**Competing interests** None.

#### S2 Post-cardiac arrest shock treated with veno-arterial extracorporeal membrane oxygenation: an observational study and propensity-score analysis

##### Wulfran Bougouin^1^, Nadia Aissaoui^2^, Alain Combes^3^, Nicolas Deye^4^, Lionel Lamhaut^5^, Daniel Jost^6^, Carole Maupain^7^, Frankie Beganton,^8^, Adrien Bouglé(), Florence Dumas^9^, Eloi Marijon^1^, Xavier Jouven^10^, Alain Cariou^11^, Sudden Death Expertise Center

###### ^1^Cardiologie, Hôpital Européen Georges-Pompidou, Rue Leblanc, Paris, France, Paris, France; ^2^Réanimation médicale, Hopital Europeen Georges-Pompidou, Paris, France; ^3^Service de Réanimation Médicale, Groupe Hospitalier Pitié Salpêtrière, Paris, France; ^4^Réanimation Médicale et Toxicologique, Hôpital Lariboisière, Paris, France; ^5^Réanimation adulte, Hôpital Necker - Enfants Malades, Paris, France; ^6^Bspp, B.s.p.p., Paris, France; ^7^Cardiologie, Pitié-Salpêtrière Hospital, Paris, France; ^8^Paris descartes, Inserm U970, Paris, France; ^9^Service d’accueil des urgences, Hôpital Cochin, Paris, France; ^10^Cardiologie, Hôpital Européen Georges-Pompidou, Paris, France; ^11^Réanimation Médicale, Hôpital Cochin, Paris, France

####### **Correspondence:** Wulfran Bougouin - wulfran.bougouin@gmail.com

*Annals of Intensive Care* 2017, **7**(**Suppl 1**):S2

**Introduction** Post-cardiac arrest (CA) shock is a mixed shock, including vasoplegia and myocardial dysfunction. Several authors reported the reversibility of post-CA myocardial dysfunction, described as a myocardial stunning. This complication often requires inotropic support, mostly dobutamine, combined with vasoactive drugs. However, these treatments are sometimes insufficient to control the circulatory failure. Veno-arterial extracorporeal membrane oxygenation (VA-ECMO) has been proposed in the most severe cases but the level of evidence is very low. We assessed characteristics, outcome and prognostic factors of patients treated with VA-ECMO for post-CA shock.

**Patients and methods** Using a large regional registry, we focused on all CA admitted in ICU. Post-CA shock was defined as the need for continuous norepinephrine or epinephrine infusion to maintain mean arterial pressure above 60 mmHg for more than 6 h following ROSC, despite adequate fluid loading. Among patients who developed a post-CA shock, prognostic was compared according to VA-ECMO use, using logistic regression and propensity score. Specific prognostic factors were identified among VA-ECMO patients.

**Fig. 10:**
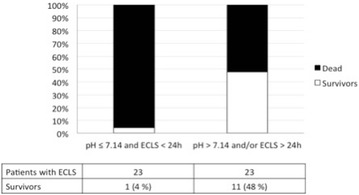
See text for description

**Results** Among 2988 patients admitted after CA, 1489 developed a post-CA shock, and were included. They were mostly male (68%), with mean age 63 years (SD = 15). 52 patients (3.5%) were treated with VA-ECMO, mostly patients with ischemic cause of CA (67%). Among patients with post-CA shock, 312 (21%) were discharged alive (25% in VA-ECMO group, 21% in control group, P = 0.45). After adjustment for pre-hospital and in-hospital factors, survival did not differ among patients treated with VA-ECMO (OR for survival = 0.9, 95% CI 0.4–2.3, P = 0.84). Results were consistent after propensity-score matching. Among patients treated with VA-ECMO, initial arterial pH (OR 1.7 per 0.1 increase, 95% CI 1.0–2.8, P = 0.04) and implantation of VA-ECMO over 24 h after ROSC (OR 20.0, 95% CI 1.4–277.3, P = 0.03) were associated with survival (Fig. 10).

**Conclusion** Post-CA shock is frequent and is associated with a high mortality rate. When used in selected patients, we observed that VA-ECMO may be an appropriate treatment.

**Competing interests** None.

#### S4 Esophageal cooling after cardiac arrest

##### Antoine Goury^1^, Florent Poirson^1^, Ulriikka Chaput^2^, Pierre Garçon^1^, Thomas Beeken^1^, Lamia Kerdjana^1^, Sebastian Voicu^1^, Isabelle Malissin^1^, Leclerc Maxime^1^, Oueslati Haikel^1^, Dominique Vodovar^1^, Jonathan Chelly^1^, Philippe Marteau^2^, Bruno Megarbane^1^, Nicolas Deye^3^

###### ^1^Réanimation médicale et toxicologique, Hôpital Lariboisière (AP-HP), Paris Cedex 10, France; ^2^Hépato-gastro-entérologie, Hôpital Saint-Antoine, Paris, France; ^3^Réanimation Médicale et Toxicologique, Hôpital Lariboisière (AP-HP), Paris, France

####### **Correspondence:** Antoine Goury - gouryantoine@gmail.com

*Annals of Intensive Care* 2017, **7**(**Suppl 1**):S4

**Introduction** Targeted temperature management (TTM) between 32 °C and 36 °C is recommended in comatose patients successfully resuscitated after out-of-hospital cardiac arrest (OHCA). Advanced cooling methods of TTM seem the most effective. This prospective, monocentric, observational, open clinical study aimed to assess the feasibility and safety of a new esophageal device to perform TTM in such patients.

**Patients and methods** The Esophageal Cooling Device (ECD^®^, Advanced Cooling Therapy, USA) is a semi-invasive multichambered silicone heat exchanger device, placed in the esophagus to provide highly efficient heat transfer, replacing the usual standard gastric tube. ECD is connected to a heat exchanger console Medi-therm III^®^ (Gamida, France) performing the temperature’s automatic feedback control. Main inclusion criteria were: OHCA from presumed cardiac cause needing TTM, delay between CA and return of spontaneous circulation (ROSC) <60 min, delay between ROSC and inclusion <240 min. Main exclusion criteria were: known esophageal disease, unstable ROSC and hemodynamic condition, severe cardiac conductive disorder requiring pacing. After achieving sustained ROSC and performing coronarography ± CT-scan, ECD was inserted as soon as possible. A target temperature (TT) of 33 °C was maintained for at least 24 h followed by a controlled rewarming (rate <0.5 °C/h). No cold fluids had to be used during TTM. Normothermia was maintained during the first 3 days after CA. Esophagogastroduodenoscopy was performed systematically after removal of ECD.

**Results** 18 patients were enrolled in the study and 17 completed the study (median [IQR 25–75], age: 60yo [54–65], SAPSII: 69 [61–75], time to ROSC: 20 min [14–30], shockable rhythm 53%). Time to reach the TT was 15 h [10–17], with a mean cooling rate to reach TT of 0.26 °C/h [0.19–0.36]. The average time spend in the 32–34 °C-target range was 26 h [21–28]. All patients reached the 32–34 °C-target range (3 patients did not reach 33 °C). The temperature deviation out of the 33 °C-TT during the maintenance phase was 0.1 °C [0.03–0.20]. Five patients (36%) presented an episode of minor deviation (0.5°–1 °C), and one patient (6%) a deviation >1 °C versus the TT. Rewarming time was 16 h [12–20] and the mean rewarming rate was 0.20 °C/h [0.18–0.22]. Average time between introduction and removed of the ECD was 50 h (42–56). Among the 16 esogastroduodenoscopy performed, 9 (56%) were strictly normal. Endoscopy showed minor gastric injuries in 6 patients (38%). Within these patients, 3 (19%) also presented minor esophageal injuries. Esogastric injuries characteristics were mostly similar to usual orogastric probe injuries. One patient (6%) experienced a serious ulcerous esophagitis mimicking a peptic esophagitis, not firmly related to the ECD. No patients necessitated hemostatic local procedure and no significant gastrointestinal bleeding was observed. Eight patients (47%) were alive at D90, including 5 patients (29%) with a Cerebral Performance Category score of 1. This compares favorably to outcomes from previous studies.

**Conclusion** ECD seems an interesting and safe semi-invasive method of cooling in OHCA patients treated with 33 °C-TTM. Although it seems slower than more invasive devices to reach 33 °C, ECD was able to strictly maintained the TT within the maintenance phase of TTM. Further studies will be necessary to define the exact place of this new device within the cooling strategy in patients necessitating a precise TTM-strategy.

**Competing interests** None.

**References**

1. Monsieurs KG, Nolan JP, Bossaert LL, Greif R, Maconochie IK, Nikolaou NI, et al. European Resuscitation Council Guidelines for Resuscitation 2015: Section 1. executive summary. Resuscitation. 2015;95:1–80.
2. Kudenchuk PJ, Sandroni C, Drinhaus HR, Böttiger BW, Cariou A, Sunde K, et al. Breakthrough in cardiac arrest: reports from the 4th Paris international conference. Ann Intensive Care. 2015;5(1):22.

#### S5 Are characteristics of receiving hospitals associated with outcome after cardiac arrest? Insights from the Great Paris registry

##### Richard Chocron^1^, Wulfran Bougouin^2^, Frankie Beganton,^3^, Philippe Juvin,^1^, Thomas Loeb,^4^, Frederic Adnet^5^, Eric Lecarpentier,^6^, Lionel Lamhaut^7^, Daniel Jost,^8^, Xavier Jouven^9^, Eloi Marijon^10^, Alain Cariou^2^, Florence Dumas^11^

###### ^1^Urgences, Hôpital Européen Georges-Pompidou (AP-HP), Paris, France; ^2^Réanimation Médicale, Hôpital Cochin, Paris, France; ^3^Paris descartes, Inserm U970, Paris, France; ^4^Samu92, Hôpital Raymond-Poincaré (AP-HP), Garches, France; ^5^Samu-smur 93, Hôpital Avicenne, Bobigny, France; ^6^Samu94, Hôpital Henri Mondor, Créteil, France; ^7^Réanimation adulte, Hôpital Necker - Enfants Malades, Paris, France; ^8^Bspp, B.s.p.p., Paris, France; ^9^Cardiologie, Hôpital Européen Georges-Pompidou, Paris, France; ^10^Cardiologie, Hôpital Européen Georges-Pompidou, Rue Leblanc, Paris, France, Paris, France; ^11^Service d’accueil des urgences, Hôpital Cochin, Paris, France

####### **Correspondence:** Florence Dumas - florence.dumas@aphp.fr

*Annals of Intensive Care* 2017, **7**(**Suppl 1**):S5

**Introduction** Since post-cardiac arrest care might influence the outcome, characteristics of receiving hospitals should be integrated in survival evaluation of patients transported in hospital. We aimed at assessing the influence of care level center on survival at discharge in a regional registry of out-of-hospital cardiac arrest (OHCA).

**Materials and methods** We prospectively collected Utstein and in-hospital data for all non-traumatic OHCA patients, in whom a successful Return of Spontaneous Circulation (ROSC) had been obtained, from a large metropolitan area (Great Paris). Receiving hospitals were categorized in 3 groups (A, B, C) depending on their respective characteristics (annual volumes, 24/7 catheterization availability and temperature management use). We compared patients’ characteristics in the 3 groups and performed a multivariable logistic regression using discharge survival at endpoint.

**Results** During the study period (May2011–Dec2013), 1476 patients were admitted in 48 hospitals (917 in group A, 428 in group B and 91 in group C). Overall survival rate at discharge was 433/1436 (30%). Patients’ baseline characteristics significantly differed, as hospitals from group A treated younger patients and more frequent shockable rhythms (p < 0.001). Unadjusted survival rate differed significantly among the 3 groups of hospitals (respectively 34, 25 and 15.4% for A, B, C, p < 0.01). However in multivariable analysis, the category of hospital was no longer associated with survival.

**Conclusion** In this population-based study, characteristics of receiving hospitals are not associated with survival rate at discharge. This could result from the strategy used for triage, which aims in matching patients’ characteristics and resources.

**Competing interests** None.

#### S6 Can urinary [TIMP-2]*[IGFBP7] predict early acute kidney injury after cardiac arrest?

##### Dimitri Titeca Beauport^1^, Magalie Joris^1^, Loay Kontar^1^, Antoine Riviere^1^, Bertand De Cagny^1^, Thierry Soupison^1^, Michel Slama^1^, Julien Maizel^1^

###### ^1^Réanimation médicale, Centre Hospitalier Universitaire, Amiens, France

####### **Correspondence:** Dimitri Titeca Beauport - titeca.dimitri@chu-amiens.fr

*Annals of Intensive Care* 2017, **7**(**Suppl 1**):S6

**Introduction** Acute kidney injury (AKI) commonly occurs after cardiac arrest and is associated with an increased mortality and a delayed awaking. Early recognition of AKI remains challenging, given that serum creatinine increases belatedly after aggression. The aim of this study was to test the hypothesis that urinary [TIMP-2]*[IGFBP7] can predict early AKI after cardiac arrest.

**Materials and methods** We conducted a monocentric prospective study from August 01, 2015 to August 31, 2016. Urinary [TIMP-2]*[IGFBP7] was measured within the 6 h following the cardiac arrest. Were excluded moribund, anuric, early RRT indication, CKD stage 4–5 and renal transplant recipients. The primary end point was to assess the predictive value of urinary [TIMP-2]*[IGFBP7], to identify early AKI defined by a KDIGO stage 3 within the first 48 h of observation.

**Results** Among forty patients analyzed, 17 (42.5%) reached KDIGO stage 3. An urinary [TIMP-2]*[IGFBP7] above 1.13 can predict KDIGO stage 3 with a sensitivity of 76.5% (50.1–93.2), specificity of 91.3% (72.0–98.9) and AUROC at 0.87 (0.73–0.96). Baseline serum creatinine above 81 µmol/l and baseline urinary output ≤0.62 ml/kg/h achieved respectively AUROC at 0.72 and 0.78. Pairwise comparisons of ROC curves were non-significant, between these 3 parameters.

**Conclusion** Early urinary [TIMP-2]*[IGFBP7] dosage achieved good performance to detect patients with high risk of AKI after cardiac arrest. However, in the limits of this study, its performances were not statistically different from baseline serum creatinine or 6 first hours urinary output.

**Competing interests** None.

#### S7 Out-of-hospital cardiac arrest between prepubescent, pubescent and adults patients: data from a national French registry (RÉAC)

##### Elodie Privat^1^, Joséphine Escutnaire^2^, Cyrielle Dumont^3^, Valentine Baert^2^, Christian Vilhelm^4^, Hervé Hubert^4^, Stéphane Leteurtre^5^

###### ^1^Pediatric Intensive Care Unit, Hôpital Jeanne de Flandre PARKING, Lille, France; ^2^French national out-of-hospital cardiac arrest registry (réac), French national out-of-hospital cardiac arrest registry (réac), Lille, France; ^3^Public health department, Université Lille 2 - Faculté de Médecine Henri Warembourg, Loos, France; ^4^Public health department, Université Lille 2 - Faculté de Médecine Henri Warembourg, Lille, France; ^5^Réanimation pédiatrique, Centre Hospitalier Régional Universitaire de Lille, Lille, France

####### **Correspondence:** Elodie Privat - elodie.privat@hotmail.fr

*Annals of Intensive Care* 2017, **7**(**Suppl 1**):S7

**Introduction** Out-of-hospital cardiac arrests (OHCA) are an absolute urgency and have a very poor prognosis. Pediatric guidelines differ from adult guidelines for cardiac arrest management. Since 2005, adult guidelines apply from the onset of puberty. The main objective was to describe the epidemiological characteristics and outcome of OHCA victims while taking puberty into account. The secondary objective was to determine the prognostic factors for survival at D30.

**Materials and methods** All patients less than 65 years of age, victims of OHCA between July 1, 2011 and September 1, 2015 care by a Mobile Emergency and Resuscitation Service (SMUR) participating in French National Cardiac Arrest registry (RéAC) were included. Patients were split into 3 groups: Prepubescent patients (named “Children”: Girls 0–9 years, Boys 0–11 years), Pubescent patients (named “Adolescents”: Girls from 10 to 17 years and Boys from 12 to 17 years) and “Adults” (men and women 18–64 years). The “Adolescents” group was consecutively compared to the “Children” group and to the “Adults” group.

**Results** 644 children, 256 adolescents and 16,566 adults under the age of 65 have been included. OHCA in Adolescents occurred more often on public roads (30%) or in public places (15%) and were more often traumatic (46%) than those in children and adults. Respiratory causes were more frequent in children (27%) than in adolescents (18%) and adults patients (18%). The proportion of shockable rhythm increased with age (3, 7 and 10% for children, adolescents and adults respectively). Survival at D30 was greater in adolescents (12%) than in children (7%) and adults (8%) (p = 0.01 and p = 0.02 respectively). In the 3 studied groups, initial shockable rhythm was a survival factor at D30 (respectively OR 18.97, 95% CI [3.84–93.78], OR 29.51, 95% CI [8.02–108.64] and OR 14.11, 95% CI [11.39–17.48] for children, adolescents and adults). Other risk factors are described in Table 11.

**Table 11:** Risk factors of survival at day 30

	Odds Ratio	IC 95%	p
*Children*			
Initial rhythm			
No shockable	Ref.		
Shockable	18.97	[3.84–93.78]	<10–3
Resuscitation duration			
>30 min	Ref.		
≤30 min	16.81	[3.91–72.21]	<10–3
*Adolescent*			
Low flow			
>15 min	Ref.		
≤15 min	45.65	[5.77–361.27]	<10–3
Initial rhythm			
No shockable	Ref.		
Shockable	29.51	[8.02–108.64]	<10–3
*Adults*			
No flow			
>10 min	Ref.		
<5 min	1.27	[1.03–1.56]	0.03
5–10 min	1.51	[1.18–1.93]	<10–3
Initial rhythm			
No shockable	Ref.		
Shockable	14.11	[11.39–17.48]	<10–3
Resuscitation duration			
>30 min	Ref.		
≤30 min	3.03	[2.29–4.02]	< 10–3

*Ref.* reference

**Conclusion** Adolescents had better survival at D30 than the 2 others groups. Adolescents and adults had shockable rhythm more often than children. Moreover, respiratory failure was less frequent in adolescent and adults patients compared to children. Puberty seems to be a good limit to differentiate pediatric patients with OHCA.

**Competing interests** None.

#### S8 Non-invasive ventilation in clinical practice: a 8-year experience in a French medical intensive care unit

##### Marion Fresco^1^, Michael Bubenheim^2^, Gaetan Beduneau^3^, Dorothée Carpentier^3^, Steven Grange^3^, Elise Artaud-Macari^3^, Benoit Misset^1^, Fabienne Tamion^4^, Christophe Girault^3^

###### ^1^Medical intensive care unit, Hospital Center University Hospital, Rouen, France; ^2^Department of clinical research support, biostatistics unit, Hospital Center University Hospital, Rouen, France; ^3^Réanimation médicale, Centre Hospitalier Universitaire Rouen, Rouen, France; ^4^Réanimation médicale, Hospital Center University Rouen, Rouen, France

####### **Correspondence:** Marion Fresco - fresco.marion@gmail.com

*Annals of Intensive Care* 2017, **7**(**Suppl 1**):S8

**Introduction** Non-invasive ventilation (NIV) is an effective alternative to endotracheal mechanical ventilation (MV) in the management of acute respiratory failure (ARF) patients. Nevertheless, it can be still difficult to assess its real feasibility, application and outcome in daily clinical practice. Therefore, we report our clinical experience with routine use of NIV since the last national recommendations (2006). Our aims were to evaluate the clinical efficacy and outcome of NIV, and to identify predictive factors for NIV failure based on a daily use.

**Patients and methods** We conducted an observational retrospective single-center cohort study by reviewing all medical records from January 2006 to December 2013 in our 22-bed medical intensive care unit (ICU). Eligible patients were those having received NIV during their ICU stay. Two groups were defined according to the indication of NIV: NIV for hypoxemic or hypercapnic ARF (ARF-NIV), and NIV used in the post-extubation period for weaning, prevention or treatment of post-extubation ARF (post-extubation NIV).The main evaluation criteria were the incidence of NIV use, success/failure rate of NIV and risk factors for NIV failure in each group. NIV failure was defined as the need for stopping NIV whatever the reason (intubation, intolerance, death) within 3 days after its initiation.

**Results** Of the 5031 MV performed in 8 years, 979 (19.5%) NIV trials were included: 805 (82.2%) for ARF-NIV (399 hypoxemic, 406 hypercapnic) and 174 (17.8%) for post-extubation NIV. The main causes for ARF were pulmonary infections (35.5%) and acute cardiogenic pulmonary edema (24.3%). The overall incidence of NIV use increased from 25.5% of ARF in 2006–36.4% in 2013 (maximum of 59.0% in 2012). The failure rate was 23.6% for the ARF-NIV group (27.8% for hypoxemic ARF, 19.5% for hypercapnic) and 14.9% for the post-extubation NIV group (p = 0.0009). Efficacy of NIV was similar despite an increased severity of patients (SAPSII) during the study period. The median length of ICU stay was 5 days (2; 9), and was longer in the post-extubation NIV group (11 days (6; 19)) than in the ARF-NIV (5 days (3; 9) for hypoxemic ARF, 4 (2; 6) for hypercapnic) (p < 0.001). The overall ICU mortality was 11.0% (15.8% in hypoxemic group, 8.1% in hypercapnic group, and 6.9% in post-extubation NIV group) (p = 0.0005).

In multivariate analysis, the main risk factors for ARF-NIV failure were: SAPS II on admission (p < 0.0001), absence of cardiologic history (p = 0.0274) and the cause of ARF (p = 0.0016) with a higher failure rate for pulmonary infections than acute cardiogenic pulmonary edema (OR 2.94, p = 0.0004). For post-extubation NIV, the only independent risk factor for failure was normocapnia before NIV initiation (p = 0.0011).

**Conclusion** Our large longitudinal study demonstrates the feasibility and efficacy of NIV applied in daily clinical practice. Provided it is performed in a suitable environment by an experienced team, NIV should be considered as a first-line ventilatory treatment in various etiologies of ARF and a very useful ventilatory support in the post-extubation period. Nevertheless, risk factors for NIV failure should be known by ICU clinicians, hypoxemic ARF remaining the more difficult indication to manage with NIV.

**Competing interests** None.

**References**

1. Demoule A, Chevret S, Carlucci A, Kouatchet A, Jaber S, Meziani F, et al. Changing use of noninvasive ventilation in critically ill patients: trends over 15 years in francophone countries. Intensive Care Med. 2016;42:82–92.
2. Schnell D, Timsit J-F, Darmon M, Vesin A, Goldgran-Toledano D, Dumenil A-S, et al. Noninvasive mechanical ventilation in acute respiratory failure: trends in use and outcomes. Intensive Care Med. 2014;40(4):582–91.

#### S9 Initial ventilation strategy and risk for intubation in immunocompromised patients with acute respiratory failure

##### Guillaume Dumas^1^, Sylvie Chevret^2^, Virginie Lemiale^1^, Djamel Mokart^3^, Julien Mayaux^4^, Frédéric Pène^5^, Achille Kouatchet^6^, Martine Nyunga^7^, Pierre Perez^8^, Anne-Sophie Moreau^9^, Fabrice Bruneel^10^, François Vincent^11^, Loay Kontar^12^, Kada Klouche^13^, Laurent Papazian^14^, Jean Reignier^15^, Antoine Rabbat^16^, Elie Azoulay^1^, Groupe de Recherche Respiratoire en Réanimation Onco-Hématologique (Grrr-OH)

###### ^1^Réanimation médicale, Hôpital Saint-Louis, Paris, France; ^2^Service de biostatistique et information médicale, Hôpital Saint-Louis, Paris, France; ^3^Réanimation, Institut Paoli-Calmettes, Marseille, France; ^4^Réanimation médicale, Hôpital Pitié-Salpêtrière, Paris, France; ^5^Réanimation Médicale, Hôpital Cochin, Paris, France; ^6^Service de Réanimation médicale et Médecine hyperbare, Centre Hospitalier Universitaire d’Angers, Angers, France; ^7^Réanimation polyvalente, Centre Hospitalier de Roubaix, Roubaix, France; ^8^Réanimation polyvalente, C.H.U. de Nancy, Nancy, France; ^9^59, C.H. Régional Universitaire de Lille (CHRU de Lille), Lille, France; ^10^Réanimation médico-chirurgicale, Centre Hospitalier de Versailles, Le Chesnay, France; ^11^Réanimation polyvalente, Groupe Hospitalier Intercommunal Le Raincy-Montfermeil, Montfermeil, France; ^12^Réanimation médicale, Centre Hospitalier Universitaire, Amiens, France; ^13^Service de réanimation, CHU Lapeyronie, Montpellier, France; ^14^Service de réanimation-détresses respiratoires et infections sévères, Hôpital Nord, Marseille, France; ^15^Réanimation, Centre Hospitalier Départemental - site de La Roche-sur-Yon, La Roche-sur-Yon, France; ^16^Réanimation pneumologique, Hôpital Cochin, Paris, France

####### **Correspondence:** Guillaume Dumas - dumas.guillaume1@gmail.com

*Annals of Intensive Care* 2017, **7**(**Suppl 1**):S9

**Introduction** Acute respiratory failure (ARF) is the leading cause for ICU admission in immunocompromised patients. In these patients, oxygenation strategy is of major interest to avoid the need for mechanical ventilation (MV), which is associated with high mortality rates. In that setting, use of non-invasive ventilation (NIV) and oxygen therapy with High Flow Nasal cannula (HFNC) could be interesting alone or in association, but data about initial ventilation strategy in immunocompromised patients are controversial.

**Patients and methods** To assess how initial oxygenation strategy actually influences the risk of MV on the coming day within the three first days of ICU stay. The study end-point was the need for MV on the coming day. We restricted analyses to these first three ICU days given, based on our own experience, most of MV was expected to occur by then.

We performed a post hoc analysis combining three prospective studies of critically ill immunocompromised patients (two randomized control trials, the IVNICTUS and the MINIMAX studies and one prospective cohort, the TRIAL-OH study). We only considered patients with ARF and a delay between ICU admission and study inclusion less than 48 h. We excluded patients who required invasive MV within the first day, those with an ICU stay less than 1 day and those with acute pulmonary edema diagnosis at ICU admission.

In order to estimate and compare the causal effect of daily respiratory management strategy on the probability of intubation in the coming day, we computed inverse probability of treatment weights (IPTW) using propensity-score, defined as the probability of actual treatment selection conditionally on observed covariates. To handle confounding in such dynamic regimens, we considered marginal structural models (MSM), which have been proposed to estimate the causal effect of a time-dependent exposure when time-dependent covariates that can be affected by the previous treatment are present. Two treatment exposure models were considered: NIV versus oxygen therapy regardless the device (model 1) and HFNC alone, NIV alone versus NIV + HFNC versus standard oxygen therapy alone (model 2).

**Results** 847 patients were included in the study. In model 1, there was no difference between NIV and oxygen groups on MV whatever the landmark time. In model 2, while the unweighted OR for intubation at day 1 was significantly higher in the NIV group (OR 2.05, 95%CI 1.29–3.29) and HFNC group (OR 2.85, 95%CI 1.37–5.67) than those in the standard oxygen alone group, these differences disappeared in the weighted samples. Using MSM, no effect of the oxygenation strategy on MV was found, regardless of the oxygenation devices but the landmark time was associated with a reduced occurrence of MV.

**Conclusion** We found no evidence of any significant difference from several oxygenation strategies on mechanical ventilation probability during the first 3 days of ICU in a large cohort of immunocompromised patients with ARF.

**Competing interests** None.

#### S10 Acute hypoxemic respiratory failure: which patients need intubation?

##### Jean-Pierre Frat^1^, Remi Coudroy^1^, Stéphanie Ragot^2^, Jean-Michel Constantin^3^, Gwenael Prat^4^, Alain Mercat^5^, Thierry Boulain^6^, Christophe Girault^7^, Alexandre Demoule^8^, Jérôme Devaquet,^9^, Saad Nseir^10^, Julien Charpentier^11^, Keyvan Razazi^12^, Laurent Argaud^13^, Pascal Beuret^14^, Jean-Damien Ricard^15^, René Robert^16^, Arnaud W Thille^1^

###### ^1^Réanimation Médicale, CHU de Poitiers, Poitiers, France; ^2^Département de biostatistiques et d’epidémiologie, CHU de Poitiers, Poitiers, France; ^3^Réanimation adulte, C.H.U. Estaing, Clermont-Ferrand, France; ^4^Réanimation médicale, CHRU de Brest, Brest, France; ^5^Service de réanimation médicale et médecine hyperbare, Centre Hospitalier Universitaire d’Angers, Angers, France; ^6^Réanimation médicale polyvalente, Hôpital de La Source, CHR Orléans, Orléans, France; ^7^Réanimation Médicale, Centre Hospitalier Universitaire Rouen, Rouen, France; ^8^Service de pneumologie et réanimation médicale, Groupe Hospitalier Pitié-Salpêtrière, Paris, France; ^9^92151, Hospital Foch, Suresnes, France; ^10^Centre de Réanimation, Centre Hospitalier Régional Universitaire de Lille, Lille, France; ^11^Réanimation Médicale, Hôpital Cochin, Paris, France; ^12^Réanimation Médicale, Hôpital Henri Mondor, Créteil, France; ^13^Réanimation Médicale, Hospices Civils de Lyon - Groupement Hospitalier Edouard Herriot, Lyon, France; ^14^Réanimation, Centre Hospitalier Général de Roanne, Roanne, France; ^15^Service de Réanimation Médico-Chirurgicale, CHU Louis Mourier, Colombes, France; ^16^Service de Réanimation médicale, CHU de Poitiers, Poitiers, France

####### **Correspondence:** Jean-Pierre Frat - jean-pierre.frat@chu-poitiers.fr

*Annals of Intensive Care* 2017, **7**(**Suppl 1**):S10

**Introduction** The role of noninvasive ventilation (NIV) is debated in the management of patients with acute hypoxemic respiratory failure. A recent study showed that patients treated with high-flow nasal cannulae oxygen therapy (HFNC) had lower intubation and mortality rates than those treated by the association of HFNC with NIV (1). High tidal volumes (VT) delivered with NIV may be associated with an increased risk of intubation (2). We aimed to identify risk factors associated to intubation, in hypoxemic patients with acute respiratory failure and especially the role of VT under NIV.

**Patients and methods** This is an ancillary study from a multicenter, randomized, controlled trial including patients with acute hypoxemic respiratory failure (FLORALI-study). We focused on only patients with moderate or severe hypoxemia (PaO_2_:FiO_2_ ratio ≤200 mmHg) and we excluded those with mild hypoxemia. The criteria for intubation were predetermined including worsened or persisted respiratory failure, impairment of neurologic status and hemodynamic instability.

**Results** After adjustment on the oxygenation strategy, the two factors independently associated with intubation were the presence of bilateral pulmonary infiltrates at admission (OR 2.33, 1.10–4.94, p = 0.03) and the respiratory rate (OR 1.04, 1.0–1.09, p = 0.05).

In patients treated with NIV, a tidal volume exceeding 9 ml/kg 1 h after NIV initiation was the only variable independently associated with intubation (OR 2.92, 1.05–8.10, p = 0.04).

In patients treated with HFNC, the respiratory rate was the only variable independently associated with intubation (OR 1.1, 1.02–1.18, p = 0.02). Patients with a respiratory rate ≥30 breath/min had a significantly increased risk of intubation (OR 3.30, 1.09–9.97, p = 0.03).

**Conclusion** Bilateral pulmonary infiltrates and a high respiratory rate were the two major factors of intubation in patients with acute hypoxemic respiratory failure. A high tidal volume (≥9 ml/kg) under NIV was associated with an increased risk of intubation.

**Competing interests** Consulting activities with Fisher & Paykel.

**References**

1. Frat JP, Thille AW, Mercat A, et al. High-flow oxygen through nasal cannula in acute hypoxemic respiratory failure. N Engl J Med. 2015;372(23):2185–96.
2. Carteaux G, Millan-Guilarte T, De Prost N, et al. Failure of noninvasive ventilation for de novo acute hypoxemic respiratory failure: role of tidal volume. Crit Care Med. 2016;44(2):282–90.

#### S11 Stress evaluation during simulated endotracheal intubation: evaluation of different variables and physiological parameters

##### Christelle Teiten^1^, Nicolas Marjanovic^2^, Nicola Palamin^3^, Erwan L’Her^4^

###### ^1^Urgences adultes, Hospital Center Regional University, Brest, France; ^2^Urgences, CHU de Poitiers, Poitiers, France; ^3^Laboratoire des usages, Irt B-Com, Plouzané, France; ^4^Réanimation médicale, CHRU de Brest, Brest, France

####### **Correspondence:** Erwan L’Her - erwan.lher@univ-brest.fr

*Annals of Intensive Care* 2017, **7**(**Suppl 1**):S11

**Introduction** Simulation is a powerful tool for technical skills teaching. No data exists on how to improve teaching and evaluating the potential impact of stress. Main objective of our study was to validate stress measurement parameters during two simulated intubation conditions.

**Materials and methods** 26 physicians were recruited (10 juniors/16 seniors) and equipped with various biometric sensors. All simulation sequences were videorecorded and physicians also weared eye-tracking glasses. Salivar cortisol measurements were performed after each sequence. Two situations were randomly tested, one in standard and easy intubation conditions, one under difficult conditions presumed to induce stress (monitoring alarms, mannikin lying on the floor, difficult intubation). Autoevaluations of video recordings and mental workload (MWL) were performed retrospectively.

**Fig. 11:**
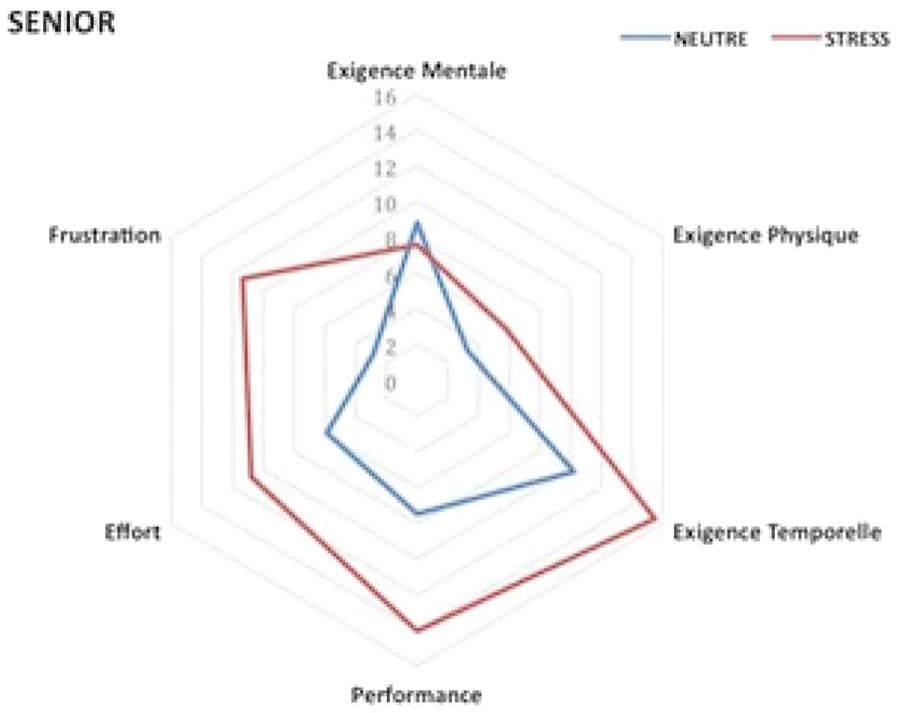
See text for description

**Results** Few differences were depicted in-between junior and seniors. Physiological parameters modifications and a heart rate variability decrease were observed under the difficult intubation conditions (SDNN for juniors = 41.2 ± 20.9 vs 29.7 ± 12.4 ms; p = 0.015). Stress was demonstrated to increase MWL (Nasa-TLX = 39 ± 18 vs. 63 ± 15; p = 0.001), frustration being its determinant component (p = 0.033) (Fig. 11). Video-recordings depicted significant stress occurence under the difficult conditions, whatever the operator’s experience.

**Conclusion** Stress is an important component of success during intubation. Simulation conditions enables to reproduce its occurence, using different types of tools, from physiological parameters to heart rate variability and psychocognitive tests. Future research is required to evaluate the impact of these parameters on teaching.

**Competing interests** None.

#### S12 Preoxygenation for intubation in the ICU: an ancillary study of the MACMAN Trial

##### Arthur Bailly^1^, Julie Boisramé-Helms^2^, Benoit Champigneulle^3^, Toufik Kamel^4^, Emmanuelle Mercier^5^, Jean-Damien Ricard^6^, Virginie Lemiale^7^, Aurélie Le Thuaut^8^, Jean Reignier^9^, Jean-Baptiste Lascarrou^1^, Clinical Research &, Intensive Care Sepsis (CRICS)

###### ^1^Réanimation, Centre Hospitalier Départemental - site de La Roche-sur-Yon, La Roche-sur-Yon, France; ^2^Réanimation, CHU de Strasbourg, Strasbourg, France; ^3^Service de réanimation polyvalente, Hôpital Européen Georges-Pompidou (AP-HP), Paris, France; ^4^Réanimation médicale polyvalente, Hôpital de La Source, CHR Orléans, Orléans, France; ^5^réanimation polyvalente, Centre Hospitalier Régional Universitaire de Tours, Tours, France; ^6^Service de Réanimation Médico-Chirurgicale, CHU Louis Mourier, Colombes, France; ^7^Réanimation médicale, Hôpital Saint-Louis, Paris, France; ^8^Biostatistiques, C.H.U. Hôtel Dieu, Nantes, France; ^9^Réanimation médicale, Chu Nantes, Nantes, France

####### **Correspondence:** Jean-Baptiste Lascarrou - jean-baptiste.lascarrou@chd-vendee.fr

*Annals of Intensive Care* 2017, **7**(**Suppl 1**):S12

**Introduction** In ICU, intubation is a high risk procedure associated with high morbidity. Despite procedure’s improvement with systematic application of fluid loading, early use of vasopressors and checklist use, morbidity remains high. Preoxygenation and intubation are the 2 main determinants of complication occurrence. McGrath Mac failed to improve first pass laryngoscopy success during intubation in ICU as compare to Macintosh laryngoscope. A predefined post hoc analysis of the MACMAN study was performed to evaluate quality of preoxygenation with 4 devices allowed: bag valve mask, non-invasive ventilation (NIV), high flow nasal cannula (HFNC), and non-re-breather mask (NRM).

**Patients and methods** MACMAN was a multicentre, open-label, randomized controlled superiority trial. Consecutive patients requiring intubation were randomly allocated to either the McGRATH MAC videolaryngoscope or the Macintosh laryngoscope, with stratification by centre and operator experience. An only inclusion criterion was: “Patients must be admitted to an ICU and require mechanical ventilation through an endotracheal tube”. Patients were excluded if: contraindication to orotracheal intubation (e.g., unstable spinal lesion); insufficient time to include and randomize the patient (e.g., because of cardiac arrest); age <18 years; pregnant or breastfeeding woman; correctional facility inmate; patient under guardianship; patient without health insurance; refusal of the patient or next of kin to participate in the study; previous enrolment in a clinical randomized trial with intubation as the primary end point (including previous inclusion in the present trial). Post-hoc analysis was performed to assess occurrence of SpO_2_ <90% during intubation procedure between 4 groups of preoxygenation: BVM (at a minimum flow of 15 L/min, NIV (100% FiO_2_), HFNC (at a minimum flow of 60 L/min, with 100% FiO_2_), and NRM (at a minimum flow of 15 L/min). Between-groups difference in desaturation occurrence was adjusted for baseline covariates significantly associated with the group membership (*P* < 0.2). Multivariate analysis of the occurrence of a desaturation (<90%) was performed using logistic regression. Bag-valve mask was considered reference.

**Results** Baseline characteristics were showed in Table 12. Groups were similar at baseline except for PaO_2_/FiO_2_ ratio. In univariate analysis, age (P = 0.08), SAPS2 (*P* = 0.02), PaO_2_/FiO_2_ ratio (*P* = 0.03),SpO2 (*P* = 0.001) and method of preoxygenation (*P* = 0.08) were associated with occurrence of desaturation below 90%. In multivariate analysis, SpO2 at randomization and method of preoxygenation were only predictors of desaturation below 90%. BVM and HRM were associated with similar risk of desaturation occurrence whereas NIV (OR 0.10 [0.01–0.81]) was protective and HFNC (OR 5.75 [1.15–28.75]) was predictive of desaturation occurrence.

**Table 12:** See text for description

	Bag valve mask N = 157	NIV N = 71	High flow nasal cannula N = 20	Non-re-breather mask N = 71	*P* value
Age	62 ± 16.4	65 ± 12.2	57 ± 17.7	63 ± 17.6	0.29
Gender male (%)	96 (61.1%)	45 (63.4%)	15 (75%)	43 (60.6%)	0.66
SAPS II score	60 ± 22	57 ± 22	51 ± 20	57 ± 21	0.38
PaO_2_/FiO_2_	156 ± 178	102 ± 65	107.5 ± 52	170 ± 157	*0.01*
SpO_2_	95 ± 6	95 ± 6	96 ± 5	96 ± 4	0.54
Successful first-pass laryngoscopy	107 (68%)	46 (65%)	16 (80%)	54 (76%)	0.34
At least one SpO_2_ < 90%	16 (11%)	2 (3%)	4 (20%)	4 (6%)	*0.04*
At least one mild to moderate complication	12 (8%)	3 (4%)	1 (5%)	2 (3%)	0.51
At least one severe complication	18 (12%)	7 (10%)	5 (25%)	6 (9%)	0.27

**Conclusion** All methods of preoxygenation were not equal for occurrence of desaturation below 90% during intubation process. Ours results suggest than NIV could be first line device during preoxygenation for intubation in the ICU. Further analysis and research are warranted.

**Competing interests** None.

**Reference**

1. Bailly A, Lascarrou JB, et al. BMJ Open. 2015;5(12):e009855.

#### S13 Cardiac arrest and mortality related to intubation procedure in critically ill adult patients

##### Amélie Rolle^1^, Audrey De Jong^1^, Gérald Chanques^1^, Samir Jaber^1^

###### ^1^DAR B, Hôpital Saint Eloi, Montpellier, France

####### **Correspondence:** Amélie Rolle - melie9712@hotmail.com

*Annals of Intensive Care* 2017, **7**(**Suppl 1**):S13

**Introduction** Intubation procedure is a challenging issue in intensive care unit (ICU) [1]. Cardiac arrest related to intubation in critically ill adult patients has been poorly studied. The studies were not powered to conclude on this rare outcome [2]. The main objective of our study was to establish the incidence of cardiac arrest and to assess the risk factors of cardiac arrest in a large prospective database of intubation procedures performed in ICU.

**Patients and methods** Five prospective studies were included, with similar data collected before, during and after intubation procedures using the same methodology. The primary outcome was the incidence of cardiac arrest related to intubation. The secondary outcomes were the death (cardiac arrest without return of spontaneous circulation (ROSC)), the cardiac arrests with ROSC, the complications related to intubation, the length of ICU stay and the 28-day mortality. The factors associated with cardiac arrest related to intubation procedures were assessed by univariate and multivariate analysis based on patient, provider and practice characteristics.

**Results** Among the 1847 intubation procedures included, 49 cardiac arrests (2.7%) occurred, including 35 with ROSC (71.4%) and 14 without ROSC (28.6%). Main patient, provider, procedure characteristics and outcomes according to cardiac arrest related to intubation are presented in Table 13. In multivariate analysis, the independent predictors of cardiac arrest related to intubation were low systolic blood pressure prior to intubation, hypoxemia prior to intubation, no preoxygenation, overweight or obesity and age >75 years. Mortality rate at day 28 was significantly lower in patients intubated without cardiac arrest (30.1%, 541 of 1798) than with cardiac arrests overall (73.5%, 36 patients of 49, *p* < 0.001) and cardiac arrest with ROSC (63%, 22 patients of 35, *p* < 0.001).

**Table 13:** Main patient, provider, procedures characteristics and outcomes according to cardiac arrest related to intubation

	Overall (n = 1847)	Cardiac arrest (n = 49)	No cardiac arrest (n = 1798)	*p* value
Age >75 years, n (%)	257 (14)	12 (24)	245 (14)	0.03
SOFA score	6 (3–8)	8 (5–10)	5 (3–8)	*0.039*
BMI (kg/m^2^)	25 (22–29)	27 (23–29)	25 (21–29)	*0.039*
Sp0_2_ < 80%, n (%)	411 (22)	22 (45)	389 (22)	<*0.001*
Lowest SBP before intubation	98 (78–120)	87 (65–105)	110 (89–130)	<*0.001*
SBP < 90 mmHg, n (%)	855 (46)	41 (84)	814 (45)	<*0.001*
MACOCHA score ≥3, n (%)	183/1108	10/28 (35)	173/1080 (16)	*0.005*
Preoxygenation, n (%)	1517 (82)	30/36 (83)	1487/1797 (96)	*0.003*
Severe complications during intubation procedure	329 (18)	49 (100)	280 (16)	<*0.001*
Severe hypoxemia	415 (22)	26 (53)	389 (22)	<*0.001*
Severe collapse	536 (29)	49 (100)	487 (27)	*0.002*
Cardiac arrest	49 (2.7)	49 (100)	0 (0)	<*0.001*
Death	14 (0.7)	14 (29)	0 (0)	<*0.001*
Moderate complications during intubation procedure	346 (19)	24 (49)	322 (18)	<*0.001*
Cardiac arrhythmia	58 (3)	15 (31)	43 (2)	<*0.001*
Inhalation	39 (2)	2 (4)	37 (2)	0.331
Esophageal intubation	86 (5)	4 (8)	82 (5)	0.232
Dental injury	15 (0.8)	0 (0)	15 (0.8)	0.522
Difficult intubation	200 (11)	7 (14)	193 (11)	0.430
Agitation	28 (2)	0 (0)	28 (2)	0.378
Length of ICU stay	15 (7–30)	2 (1–10)	16 (7–30)	*0.001*
Mortality 28 days	576 (31)	36 (73)	541 (30)	<*0.001*

Data expressed as median (25–75% IQR) or proportion (%)

**Conclusion** Cardiac arrest related to intubation in adult ICU is not a rare event occurring in 2.7% of cases with high immediate mortality of 28.6% and at day 28 of 73.5%. We identified five independent risk factors to cardiac arrest which 3 of them could be modifiable. Optimal preparation to intubation procedure could help to prevent those cardiac arrests.

**Competing interests** None.

**References**

1. De Jong A, Molinari N, Terzi N, Mongardon N, Arnal JM, Guitton C, et al. Early identification of patients at risk for difficult intubation in the intensive care unit: development and validation of the MACOCHA score in a multicenter cohort study. Am J Respir Crit Care Med. 2013;187(8):832–9.
2. Jaber S, Amraoui J, Lefrant JY, Arich C, Cohendy R, Landreau L, et al. Clinical practice and risk factors for immediate complications of endotracheal intubation in the intensive care unit: a prospective, multiple-center study. Crit Care Med. 2006;34(9):2355–61.

#### S14 Orotracheal versus nasotracheal intubation: a prospective observational study

##### Geoffroy Hariri^1^, Jean-Luc Baudel^1^, Vincent Dubée^1^, Gabriel Preda^1^, Simon Bourcier^1^, Jeremie Joffre,^2^, Naïke Bigé^1^, Hafid Ait-Oufella^1^, Eric Maury^1^

###### ^1^Réanimation médicale, Hôpital Saint-Antoine, Paris, France; ^2^Service de reanimation médicale, Hôpital Saint-Antoine, Paris, France

####### **Correspondence:** Eric Maury - ejhmaury@gmail.com

*Annals of Intensive Care* 2017, **7**(**Suppl 1**):S14

**Introduction** Naasotracheal intubation (NTI) has been progressively given up in favour of the orotracheal intubation (OTI) in intensive care unit (ICU). This could be explained by more frequent infectious (sinusitis and ventilator associated pneumonia) and non-infectious (epistaxis, turbinates bones injury) complications the former being thought to be more frequent with NTI. However, whereas infectious sinusitis is a risk factor for VAP, no study has yet demonstrated that OTI decreases the infectious sinusitis rate compared with NTI. Furthermore, nasal route could improve patient comfort and decrease auto-extubation. Finally NTI can be performed without laryngoscopy with less risk of lips and dental injury. In this prospective study, we aimed to compare the complication of NTI and OTI and to assess the comfort of the patient.

**Patients and methods** We performed a prospective observational study in a 12-bed medical ICU including patients requiring endotracheal intubation. The intubation route was let at the discretion of the physician in care of the patient, however OTI was compulsory in case of cardiac arrest, severe hypoxemia (P/F < 150 when available) and clotting perturbation. For each patient, age, sex, SAPSII, mechanical ventilation duration. intubation route were recorded as well as complications during the placement of endotracheal tube. Infectious and non infectious complications during invasive ventilation period were also recorded. In patients who were successfully extubated, pain, burning feeling, dryness and the wish of tube removal were assessed using visual analogic scales (VAS).Results are expressed as mean ± SD. Qualitative data were compared using Chi-2 or Fisher’s exact test while quantitative data comparisons used Student t Test or Mann–Whitney as appropriate.

**Results** One hundred and thirty-seven patients were included (60 women, age: 65 ± 17 years, SAPSII: 49 ± 18, mechanical ventilation duration 4.2 ± 3 days). Among them, 90 received OTI and 47 NTI. No statistical difference was found between initial characteristics of the two groups.

During the placement of endotracheal tube, the NTI group received less sedatives (66 vs 91%; p < 0.0006) and less frequently succinyl choline (6 vs 71%; p = 0.0001). NTI was performed without laryngoscopy in 77% of patients. Selective intubations and desaturation during the procedure were observed with a similar frequency in the two groups (13.6% OTI vs 4 NTI p = 0.13).Lips injury were more frequent after OTI (7 vs 0%, p = 0.09). Epistaxis occurred in 15% of patients in the NTI group vs none in the OTI group (p < 0.05) and one required a blood transfusion.

While the endotracheal tube was in place, ulcerations caused by the tube or the fastening system were more frequent in the OTI group [lips ulcerations, n = 27 (30%) versus nostrils ulcerations in the NTI group, n = 6 (13%) (p < 0.05)]. Tube repositioning was required in 22% of patients in the OTI group versus 11% in the NTI group (p = 0, 1) while self extubation was observed in 3 patients in each group (p = 0.4). Ventilator-associated pneumonia occurred in 4% in OTI and 7.5% NTI (p = 0.69). Other infectious complications were one sinusitis in the NTI group (p = 0.34) and 3 labial herpes in the OTI group (p = 0.55). Guedel cannula was used only in the OTI group (9 vs 0%, p = .05).

Among the 58 patients successfully extubated, pain caused by the tube was similar (3, 4/10 in the OTI group vs 3/10 in the NTI, p = 0.6). Burning feeling was similar (2, 3/10 in the OTI vs) 3, 2/10 in the NTI, p = 0.3), while dryness feeling was more important in the OTI (VAS 5.4 vs 3.8 in the NTI, p = 0.05). The wish of tube removing was greater in the OTI group (5/10) and 4/10 in the NTI (p = 0.3).

**Discussion** In this pilot study, patients in the NTI group required less frequently sedative and succinyl-choline use during tracheal intubation. During mechanical ventilation, ulcerations were more frequent in the OTI group. No difference between the two routes was found concerning the infectious complications while the endotracheal tube was in place.

**Conclusion** Despite its small size, and the absence of randomization, the present study suggests that nasotracheal intubation improves the comfort and the tolerance of tracheal intubation and is not associated to higher rates of VAP.

**Competing interests** None.

#### S15 Effect of mode of hydrocortisone administration in patients with septic shock: a prospective randomized trial

##### Oussama Jaoued^1^, Rim Gharbi^1^, Najla Tilouch^1^, Ben Sik Ali Habiba^2^, Houda Mater^1^, Mohamed Fekih Hassen^1^, Souheil Elatrous^1^

###### ^1^Réanimation Médicale, EPS Taher Sfar Mahdia, Mahdia, Tunisia; ^2^Réanimation médicale, EP taher sfar, Mahdia, Tunisia

####### **Correspondence:** Mohamed Fekih Hassen - mohamed.fekihhassen@rns.tn

*Annals of Intensive Care* 2017, **7**(**Suppl 1**):S15

**Introduction** There is a strong biological rationale to support the use of low to moderate doses of corticoids for the treatment of vasopressor-dependent septic shock. The question of whether corticosteroids should be given to septic shock patients by continuous or by bolus infusion is still unanswered.

**Objective**: to evaluate the effect on morbidity and mortality of mode of hydrocortisone administration in patients with septic shock.

**Patients and methods** We conducted a prospective randomized and monocentric study in the medical intensive care of the teaching Hospital of Taher Sfar in Mahdia between April 2013 and June 2016. We assigned patients with septic shock to receive hydrocortisone treatment either by bolus or by continuous infusion with equivalent dose (200 mg/day). Corticosteroids were continued as long as vasopressors were needed. The primary endpoint was all cause of ICU mortality. Secondary endpoints included length of stay, number of vasopressor free days, change in SOFA score, leukocyte count, temperature, plasma C-reactive protein, and the occurrence of adverse events (Secondary sepsis, hyperglycemia, hypokalemia, peripheral neuropathy).

**Results** The trial included 54 patients, of whom 27 were assigned to continuous infusion of hydrocortisone and 27 to discontinuous mode. The baseline characteristics of patients were similar between the two groups. Sepsis was secondary to community-acquired infection in 54% of cases. There was no difference in mortality between groups (74% in continuous groups and 59% in discontinuous group). SOFA score was significantly higher at days 1, 2 and 5 in discontinuous group. Length of stay, duration of mechanical ventilation, number of day without vasopressors, and the occurrence of adverse events were similar in the two groups.

**Conclusion** The mode of hydrocortisone administration in patients with septic shock has no influence on morbidity or mortality. The occurrence of adverse events was similar.

**Competing interests** None.

#### S16 Changes in platelet counts are not associated with the development of ICU-acquired infections in patients with severe sepsis and septic shock

##### Hamid Merdji^1^, David Grimaldi^1^, Julien Charpentier^1^, Christophe Rousseau^2^, Jean-Paul Mira^1^, Jean-Daniel Chiche^1^, Frédéric Pène^1^

###### ^1^Réanimation médicale, Hôpital Cochin, Paris, France; ^2^Unité 1016, 22 rue méchain, 75014, paris, Institut National de la Santé et de la Recherche Médicale, Paris, France

####### **Correspondence:** Hamid Merdji - hamidmerdji@hotmail.fr

*Annals of Intensive Care* 2017, **7**(**Suppl 1**):S16

**Introduction** Widespread activation of coagulation with platelet consumption is a pathophysiological feature of severe sepsis and septic shock. Thrombocytopenia, either defined by platelet count below 150 G/L or by a significant relative 20–40-percent decrease in platelet count is a potent poor prognostic factor in sepsis. Besides their role in hemostasis, platelets also carry various immune and inflammatory functions that are likely to impact on host defense against infections. We aimed to assess whether changes in the platelet count induced by sepsis is associated with the development of subsequent nosocomial infections.

**Patients and methods** Patients were obtained from two prospective studies about immuno monitoring of dendritic cells and innate-like lymphocytes in critically ill septic patients (1, 2). Adult patients with severe sepsis and septic shock were included. Exclusion criteria were any immunosuppressive condition (hematological malignancy, HIV infection at any stage, bone marrow or solid organ transplantation, daily corticosteroid therapy >0.5 mg/kg prednisone-equivalent, chemotherapy or any other immunosuppressive treatments), pregnancy, do-not-resuscitate orders on admission. In addition patients who died or who received platelet transfusion during the first week after ICU admission were also excluded. Platelet counts were collected on the day of sepsis diagnosis (D1) and then on D3, D5 and D7. The relative variation in platelet count at day n compared to day 1 was calculated as follows: (count at day n − count at day 1) × 100/(count at day 1). Patients were screened daily for the development of ICU-acquired infections diagnosed according to usual guidelines (for ventilator acquired pneumonia, urinary tract infection, intravascular catheter-related infection).

**Results** 94 patients were included in the study (65 years old, 68% of men, APACHE 2 = 28). The overall in-ICU mortality rate was 15%. 21 (22%) patients developed ICU-acquired infections after a median of 8 [7–13] days following ICU admission. Patients with nosocomial infections were older (68 [62–73] vs. 64 [60–68] years, p = 0.34, had higher severity at admission (APACHEII 31 [28–34] vs. 27 [25–29], p = 0.09) and finally had higher mortality rate (29 vs. 10%, p < 0.05).

**Fig. 12:**
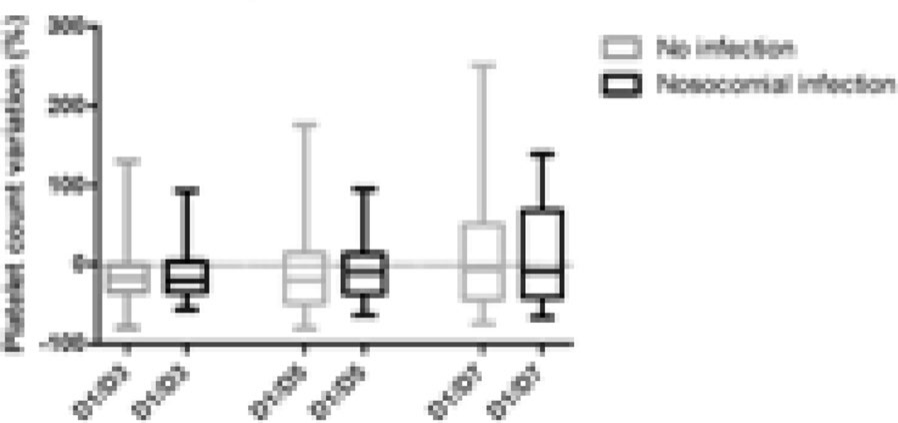
Trends in platelets count over time in patients with and without ICU acquired infection

The average admission platelet count was 239 [210–269] G/L and non significantly dropped to 185 [158–212] G/L at 3 days, 182 [159–205] G/L at 5 days and 222 [199–246] G/L at 7 days. Platelet counts at D1, D3, D5 and D7 appeared similar in patients without and with further nosocomial infections. The relative variations in platelet counts between between D1 and D3, between D1 and D5 and between D1 and D7 were also similar between patients with and without ICU-acquired infections (Fig. 12).

**Discussion** In this preliminary study from selected cohorts of non-immunocompromised patients, sepsis resulted in mild alterations in platelet counts, making it unlikely to become associated with the development of nosocomial infections. It would be relevant to address this question in larger cohorts of non-selected patients, as well as the impact of platelet transfusions in this setting.

**Conclusion** Changes in platelet counts were not associated with an increased susceptibility towards ICU-acquired infections in non-immunocompromised patients with severe sepsis and septic shock.

**Competing interests** None.

**References**

1. Grimaldi, et al. Profound and persistent decrease of circulating dendritic cells is associated with ICU-acquired infection in patients with septic shock Intensive Care Med. 2011;37(9):1438–46.
2. Grimaldi, et al. Specific MAIT cell behaviour among innate-like T lymphocytes in critically ill patients with severe infections. Intensive Care Med. 2014;40(2):192–201.

#### S17 Sepsis and septic shock: prognostic value of lymphocytopenia

##### Ines Sedghiani^1^, A Benabderrahim, Dhekra Hamdi, Asma Jendoubi, Mohamed Ali Cherif, Youssef Zied El Hechmi, Jerbi Zouheir

###### ^1^Emergency and intensive care department, Hôpital Babib Thameur, Tunis, Tunisia

####### **Correspondence:** Ines Sedghiani - sedghiani.ines@gmail.com

*Annals of Intensive Care* 2017, **7**(**Suppl 1**):S17

**Introduction** Sepsis is the leading cause of mortality in the intensive care unit (ICU) patients despite the progress regarding their care. The immunodeficiency due to sepsis with the consequent profound lymphocyte alterations is now well proven. The objective of this work was to determine the prognostic impact of lymphocytopenia in septic patients in ICU.

**Patients and methods** Retrospective study including all patients hospitalized for sepsis or septic shock between 01/01/2013 and 31/05/2015. The sepsis and septic shock definitions were adjusted with the third international consensus definitions for sepsis and septic shock. Were excluded from the study patients of onco-hematology.

Lymphocytopenia was defined as an absolute lymphocyte count less than level of 1500/mm^3^ during the first 24 h of hospitalization. The prognostic factors analyzed for the lymphopenic and non lymphopenic patients were in hospital mortality, the occurrence of nosocomial infections and hospital length of stay.

**Results** Among the 52 patients, aged 58 ± 17 years, 23 patients were with septic shock and 29 patients with sepsis. IGSII score and SOFA score were respectively 42 ± 19 and 5 ± 3. Four patients were immunocompromised due to HIV infection in one case and an immunosuppressive therapy in 3 cases. Lymphocytopenia was observed in 41 patients (78%). Twenty-eight patients (53%) died within an average of 11 ± 13 days. It was noted the occurrence of 10 nosocomial infections. The median length of stay was 7 days with extremes of one and 74 days. The lymphopenic patients were comparable to non lymphopenic patients in terms of medical history and severity scores. Mortality was comparable between the 2 groups with a rate of 51% (n = 21) in lymphopenic patients and 64% (n = 7) in non-lymphopenic patients (*p* = 0.51). The earliness of death was correlated with the duration of lymphopenia (*R* ^2^ = 0.32*, p* = 0.007*)*. The occurrence of nosocomial infections was not different between the two groups: 17% (n = 7) for lymphopenic and 27% (n = 3) for non lymphopenic patients. The hospital length of stay was not different between the two groups but was correlated with the duration of lymphocytopenia (*R* ^2^ = 0.29, *p* = 0.000).

**Conclusion** Lymphocytopenia is frequently found in sepsis. Lymphocytopenia was not associated with excess of mortality nor with the subsequent occurrence of infectious complications during the ICU stay. His persistence was associated with an earlier death and a more prolonged hospitalization.

**Competing interests** None.

#### S19 Role of relative adrenal insufficiency in septic cardiomyopathy: a prospective cohort study

##### François Bagate^1^, Keyvan Razazi^1^, Guillaume Carteaux^1^, Nicolas de Prost^1^, Armand Mekontso Dessap^1^

###### ^1^Réanimation médicale, Hôpital Henri Mondor, Créteil, France

####### **Correspondence:** François Bagate - francois.bagate@aphp.fr

*Annals of Intensive Care* 2017, **7**(**Suppl 1**):S19

**Introduction** Relative adrenal insufficiency (RAI) is common in ICU patients, particularly during septic shock (1). It has been shown that the RAI also occurs during cardiogenic shock (2). Septic cardiomyopathy occurs in a significant proportion of septic shock patients. The aim of this study was to evaluate the role of RAI on septic cardiomyopathy.

**Patients and methods** Prospective observational study conducted in the intensive care in one university hospital in France. Patients meeting the criteria for septic shock without prior corticosteroid therapy and without chronic heart disease were included. Total blood cortisol levels were assessed immediately before (T0) a short corticotropin stimulation test (0.25 mg iv of tetracosactrin) and 30 and 60 min afterward. Δmax was defined as the difference between the maximal value after the test and T0. RAI was defined as an inappropriate adrenal response with Δmax < 9 µg/dl and septic cardiomyopathy as the appearance of cardiac systolic dysfunction (left ventricle ejection fraction <45%) within the first 3 days of septic shock. We performed a multivariable analysis using backward stepwise logistic regression to identify independent predictors of septic cardiomyopathy.

**Results** Of the 52 patients enrolled, 19 (37%) died during ICU stay and 22 (42%) during hospitalization. Septic cardiomyopathy was revealed in 18 patients (35%). Overall, RAI occurred in 32 patients (62%) and this prevalence was significantly higher in patients with septic cardiomyopathy as compared to their counterparts (83 vs. 50%, p = 0.03) and in non-survivors as compared to survivors (82 vs. 47% p = 0.02). In the multivariable analysis, RAI was an independent predictor of septic cardiomyopathy (OR 4.74, p = 0.03), along with SAPS2 (OR 1.06, p < 0.01). Results were similar when the use of etomidate was forced into the model as a potential confounder.

**Discussion** Although the definition of RAI is not consensual, a threshold of Δmax at 9 µg/dl has been widely used in septic shock, with or without the use of T0 (1). The usefulness of substitutive doses of steroids in septic shock is controversial, but many authors assume this treatment has a potential in reversing overt vasoplegia. Our data suggest an implication of RAI in septic cardiomyopathy.

**Conclusion** We found RAI to be an independent predictor of septic cardiomyopathy. These findings may suggest a new role for substitutive doses of steroids in the hemodynamic management of septic shock.

**Competing interests** None.

**References**

1. Annane D, Sebille V, Troche G et al. A 3-level prognostic classification in septic shock based on cortisol levels and cortisol response to corticotropin. JAMA. 2000;283:1038–45.
2. Bagate F, Lellouche N, Lim P et al. Prognostic value of relative adrenal insufficiency during cardiogenic shock: a prospective cohort study with long-term follow-up. Shock. 2016.

#### S20 Effects of continuous haemofiltration versus intermittent haemodialysis on microcirculatory parameters in septic shock: a muscle microdialysis study

##### Zied Hajjej^1^, Walid Sellami^1^, Walid Samoud^1^, Radhwen Bousselmi^1^, Iheb Labbene^1^, Mustapha Ferjani^1^

###### ^1^Department of Critical Care Medicine and Anesthesiology, Military Hospital of Tunis, Tunisia, Tunis, Tunisia

####### **Correspondence:** Zied Hajjej - hajjej_zied@hotmail.com

*Annals of Intensive Care* 2017, **7**(**Suppl 1**):S20

**Introduction** Regional perfusion parameters, like lactate, pyruvate and glycerol, may predict outcome in septic shock patients. Continuous venovenous haemofiltration (CVVH) has been considered beneficial in septic shock patients. The aim of our study was to investigate whether CVVH, in comparison to intermittent haemodialysis (IHD), is able to improve regional perfusion in septic shock patients studied by muscle microdialysis.

**Patients and methods** It was a prospective, randomized, clinical study, including septic shock patients with acute renal failure, aged over 16 years. Patients were randomized to receive either CVVH (n = 10) or IHD (n = 10) for renal replacement therapy. Intermittent haemodialysis was carried out during the first 4 h of the 24 h study period. Systemic haemodynamics and interstitial tissue concentrations of lactate, pyruvate, glucose and glycerol were obtained at baseline, 6, 12, 18 and 24 h after initiation of renal replacement by using muscle microdialysis.

**Results** Regarding systemic haemodynamics parameters, CVVH caused a decrease in heart rate in contrast to IHD after 6 h (−4 ± 2 vs +6 ± 3/mn). There were no changes in vasopressor support throughout the 24-h study period and so systolic blood pressure remained stable in both groups.

During the 24 h of all renal replacement therapies there was no significant change in muscle pyruvate and glucose levels.

**Fig. 13:**
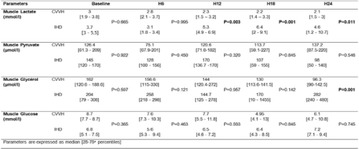
Muscle biological data measured by microdialysis during study period

During CVVH muscle lactate decreased constantly, as did muscle glycerol levels. This decrease reaches a significant levels at H12 for muscle lactate and at H24 for muscle glycerol (Fig. 13).

**Conclusion** Our results suggest that among septic shok patients, CVVH may improves regional perfusion in comparison with IHD.

**Competing interests** None.

#### S21 Hypernatremia as a risk of death during hypertonic fluid resuscitation in septic shock: post hoc analysis of the “Hyper2S” study

##### Frédérique Schortgen^1^, Pierre Asfar^2^, Boisramé-Helms Julie^3^, Julien Charpentier^4^, Emmanuel Guérot^5^, Bruno Megarbane^6^, David Grimaldi^7^, Grelon Fabien^8^, Nadia Anguel^9^, Lasocki Sigismond^10^, Henry-Lagarrigue Matthieu^11^, Frédéric Gonzalez^12^, Legay François^13^, Christophe Guitton^14^, Maleka Schenck^3^, Doise Jean-Marc^15^, Didier Dreyfuss^16^, Peter Radermacher^17^, for the HYPER2S Investigators and REVA research network

###### ^1^Réanimation médicale, Hôpital Henri Mondor, Créteil, France; ^2^Réanimation, C.H.U. d’Angers, Angers, France; ^3^Réanimation médicale, C.H.R.U. Hôpitaux Universitaires Strasbourg, Strasbourg, France; ^4^Réanimation Médicale, Hôpital Cochin, Paris, France; ^5^Réanimation médicale, Hopital Europeen Georges-Pompidou, Paris, France; ^6^Service de Réanimation Médicale et Toxicologique, CHU Lariboisière, Paris, France; ^7^Service de réanimation polyvalente, Centre Hospitalier de Versailles, Le Chesnay, France; ^8^Réanimation, C.H. - Le Mans, Le Mans, France; ^9^Réanimation médicale, CHU de Bicêtre, Le Kremlin Bicêtre, France; ^10^Réanimation chirurgicale, C.H.U. d’Angers, Angers, France; ^11^Réanimation polyvalente, Hospital Center Departmental De Vendée, La Roche-sur-Yon, France; ^12^Réanimation medico-chirurgicale, hopital avicenne, Bobigny, France; ^13^Réanimation, C.H. de Saint Brieuc, Saint-Brieuc, France; ^14^Réanimation médicale, C.H.U. Hôtel Dieu, Nantes, France; ^15^Réanimation, C.H. Chalon sur Saône William Morey, Chalon-sur-Saône, France; ^16^Réanimation polyvalente, Hôpital Louis-Mourier (AP-HP), Colombes cedex, France; ^17^Institut für anästhesiologische pathophysiologie und verfahrensentwicklung, Universitätsklinikum Ulm, Ulm, Germany

####### **Correspondence:** Frédérique Schortgen - frederique.schortgen@aphp.fr

*Annals of Intensive Care* 2017, **7**(**Suppl 1**):S21

**Introduction** Acquired hypernatremia (H-Na) is an independent risk of death among ICU patients (1). In the RCT “Hyper2S” study, we compared normal to 3% hypertonic saline during the first 72 h in patients with septic shock with normal serum Na concentration (SNa) at baseline. The study was prematurely stopped for potential harmful effect associated with more frequent H-Na. We assessed the role of H-Na on mortality.

**Patients and methods** Data are a post hoc analysis of the “Hyper2S” study database including 434 patients. SNa was measured at H0, every 12 h for 3 days and then daily until D7. Study fluids were stopped if SNa >155 or >12 mmol/l increase over 24 h. Mild, moderate, and severe H-Na were defined as SNa >145 mmol/L, >150 mmol/L and >155 mmol/L, respectively. SNa profiles were compared between D28 survivors and non-survivors. Acute kidney injury (AKI) was defined by doubling serum creatinine and/or need for dialysis.

**Results** 431 patients with available data were analysed. 234 (54%) developed H-Na (mild: 28%, moderate: 16%, severe: 10%). No matter the absence or presence and its severity, H-Na did not affect mortality (43, 34, 41, and 35%, respectively without, with mild, moderate, and severe H-Na, p = 0.41). SNa profiles were similar between survivors and non-survivors (Table 14). A sensitivity analysis performed among survivors at D3 did not change the results. Compared to patients without H-Na, AKI occurred in 42% of patients with H-N vs 40% (p = 0.58), atelectasis in 8 versus 10% (p = 0.58) and ICU acquired weakness in 7 versus 10% (p = 0.23).

**Table 14:** Serum Na profile in D28 survivors and non-survivors (results are presented as number and % of patients and medians IQR)

	Survivors N = 262	Non-survivors N = 169	OR 95% CI, p
Hypernatremia, n (%)			
No	113 (43)	84 (50)	1
Yes	149 (57)	85 (50)	0.77 (0.52–1.13), 0.18
Hypernatremia, n (%)			
No	113 (43)	84 (50)	1
Mild	80 (31)	41 (24)	0.69 (0.43–1.10), 0.12
Moderate	41 (16)	29 (17)	0.95 (0.55–1.65), 0.86
Severe	28 (11)	15 (9)	0.76 (0.34–1.66), 0.49
Max SNa (mmol/L)	146 (143–151)	145 (141–149)	0.98 (0.95–1.01), 0.22
Mean SNa (mmol/L)	141 (138–144)	141 (138–145)	1.00 (0.96–1.06), 0.85
Max increase: SNa Max-SNa H0 (mmol/L)	7 (3–13)	6 (1–12)	0.98 (0.95–1.01), 0.15
Max SNa increase rate (mmol/L/h)	0.33 (0.23–0.58)	0.33 (0.17–0.75)	1.31 (0.84–2.07), 0.23

**Conclusion** Hypernatremia occurrence is not associated with an increased risk of morbidity and mortality during hypertonic fluid resuscitation in septic shock.

**Competing interests** None.

**Reference**

1. Darmon M, et al. Nephrol Dial Transplant 2010;25:2510–5.

#### S22 Management of moderate hypokalemia in a medico-chirurgical intensive care unit

##### Antoine Frère^1^, Laurent Martin-Lefèvre^1^, Aurélie Le Thuaut^2^, Gwenhaël Colin^1^, Maud Fiancette^1^, Matthieu Henry-Laguarrigue^1^, Jean-Claude Lacherade^1^, Jean-Baptiste Lascarrou^1^, Christine Lebert^1^, Isabelle Vinatier^1^, Aihem Yehia^1^

###### ^1^Réanimation polyvalente, Centre Hospitalier Départemental - site de La Roche-sur-Yon, La Roche-sur-Yon, France; ^2^Unité de recherche clinique, Centre Hospitalier Départemental - site de La Roche-sur-Yon, La Roche-sur-Yon, France

####### **Correspondence:** Antoine Frère - antfrere@live.fr

*Annals of Intensive Care* 2017, **7**(**Suppl 1**):S22

**Introduction** Guidelines about the moderate hypokalemia treatment (between 2.5 mmol/l and 2.9 mmol/l) are based on experts estimations, and non-specific ones for patients in the intensive care units (ICU). The aim of this study was to evaluate the correction of the hypokalemia in an ICU and the compliance of recommendations.

**Materials and methods** An observational epidemiological, retrospective and monocentric trial has been realized during a period of 13 months (from January 2015 to February 2016). The study population included hospitalized patients in the ICU who have shown a first moderate hypokalemia episode, all cause considered. Patients who have presented an acute renal failure with a KDIGO (Kidney Disease: Improving Global Outcomes) score of three the day of their inclusion were excluded. The main primary study endpoint was percent correction of the serum potassium after 24 h. The secondary study endpoints were the incidence rate of moderate hypokalemia and the efficacy about the hypokalemia correction in accordance with the achieved treatment consistent or not with recommendations.

**Results** 140 patients had at least one episode of hypokalemia. The incidence rate of the hypokalemia first episode was 1.8%. The study population included 106 patients. IGS2 score was 47.1 (± 16). 48 patients required mechanical ventilation at the inclusion. The serum potassium was greater than or equal to 3.5 mmol/l after 24 h about 34 patients (32.1%) (corrected group). At 24 h one patient had a serum potassium higher than 5 mmol/l. The average total potassium was respectively 72.3 mmol/L (±57) in the corrected group and at 75.4 mmol/l (±53.67) in the non-corrected group (p = 0.96). Among 75 patients without contraindication to the enteral administration, this one was used in 46.7% (n = 35) of cases. 47/75 (62.7%) of these patients have received continuous intravenous infusion of potassium and 7 (6.6%) patients have been a management compatible with the most common recommendations (input potassium chloride of 100 mmol, use of the enteral administration and lack of continuous intravenous infusion). The percent correction of the hypokalemia after 24 h was respectively of 2/7 (28.6%) in the group in which recommendations had been respected and of 32/99 (32.3%) in the other one (p = 1.00).

**Discussion** In our knowledge there are no previous studies that have specifically focused on the correction of the moderate hypokalemia in critically ill patients. In our study the incidence rate of the moderate hypokalemia was lower than data from the literature because we have only considered the first episode of the hypokalemia [1]. Among patients without contraindication to the enteral administration, this one was used in less than half of the cases. 62% of these patients received potassium with a continuous intravenous infusion and only 7 patients received medical care conform to the guidelines. The medium potassium quantity provided was very lower to the guidelines. Only 32% of the patients have been corrected after 24 h without any difference in the medium potassium quantity which has been provided in relation to the uncorrected group.

**Conclusion** Only 32.1% of moderate hypokalemia in ICU are corrected after 24 h. The intravenous way is considerably used (in 72% of cases) with a poor return. A wide-ranging study is necessary to determine the best correction modes.

**Competing interests** None.

**Reference**

1. Hessels L, Hoeskstra M, Mijzen LJ, Vogelzang M, Dieperink W, Lansink A, et al. The relationship between serum potassium, potassium variability and in-hospital mortality in critically ill patients and a before-after analysis on the impact of computer-assisted potassium control. Crit. Care. 2015;19(1):4.

#### S23 Nephrotoxic drug prescription in intensive care units: 1028 patient prospective cohort

##### Aurélie Joret^1^, Julie Boisramé-Helms^2^, Laurent Martin-Lefèvre^3^, Nicolas Menunier-Beillard^4^, Jean-Etienne Herbrecht^5^, Dalila Benzekri-Lefevre^6^, René Robert^7^, Arnaud Desachy^8^, Fréderic Bellec^9^, Gaëtan Plantefève^10^, Jean-Pierre Quenot^4^, Jean-Claude Lacherade^3^, Ferhat Meziani^2^, Elsa Tavernier^11^, Stephan Ehrmann^1^, Clinical Research in Intensive Care and Sepsis (CRICS network)

###### ^1^Réanimation polyvalente, CHRU de Tours, Tours, France; ^2^ Réanimation médicale, CHU de Strasbourg, Strasbourg, France; ^3^Réanimation polyvalente, CHD de Vendée, La Roche-sur-Yon, France; ^4^Réanimation médicale, CHU de Dijon, Dijon, France; ^5^Réanimation médicale, CHRU de Strasbourg, Strasbourg, France; ^6^Réanimation médicale polyvalente, CHR d’Orléans, Orléans, France; ^7^Réanimation médicale, CHU de Poitiers, Poitiers, France; ^8^Réanimation polyvalente, CH D’Angoulême, Angoulême, France; ^9^Réanimation, CH de Montauban, Montauban, France; ^10^Réanimation polyvalente, CH Victor Dupouy, Argenteuil, France; ^11^Inserm cic 1415, CHRU de Tours, Tours, France

####### **Correspondence:** Aurélie Joret - aureliejoret@yahoo.fr

*Annals of Intensive Care* 2017, **7**(**Suppl 1**):S23

**Introduction** Acute Kidney Injury (AKI) is frequent in intensive care unit (ICU) patients and associated with poor prognosis. Among the various contributing factors, administration of nephrotoxic drugs is potentially avoidable. A better knowledge of the nephrotoxic prescription pattern and its contribution to renal injury in ICU patients, may enable to identify opportunities for improvement. The objective of this study was to describe the frequency and modality of nephrotoxic drug prescription during the first 7 days in the ICU.

**Patients and methods** Prospective observational study including all patients admitted during two 4 weeks periods, between 2013 and 2015, in 10 ICUs in France. Patients undergoing chronic dialysis, patients with renal replacement therapy within the preceding week of ICU admission as well as patients admitted for benign psychotropic poisoning without organ failure were not included. Nephrotoxic drugs received by the patients were collected daily. AKI was defined according to the KDIGO (Kidney Disease: Improving Global Outcomes) criteria.

**Results** 1028 patients were included. Mean ± SD age was 65 ± 16 years, 64% were male, mean ± SD SAPS II was 47 ± 20. ICU length of stay was 7 ± 11 days and ICU mortality rate was 23%. During the first 7 days in the ICU, 60% of patients received at least one nephrotoxic drug. 31% of patients received one, 18% received two, 9% received three and 3% received more than three nephrotoxic medications. Diuretics, antibiotics and iodinated contrast media were the nephrotoxic drugs most frequently administered to, respectively, 35, 22 and 15% of patients. AKI (KDIGO stage 1 or higher) occurred in 58% of patients during the first 7 days in ICU. The proportion of patients with AKI increased with the number of nephrotoxic drugs received: 189/410 (46%) of the patients not exposed to nephrotoxic drugs developed AKI whereas, respectively, 189/315 (60%), 136/184 (74%), 62/91 (68%) and 24/28 (85%) of the patients receiving one, two, three, and more than three nephrotoxic drugs developed AKI. The univariate association between the number of nephrotoxic medication and AKI persisted in the multivariate analysis adjusted on baseline SAPS II score (p < 0.001).

**Conclusion** The significant proportion of patients exposed to nephrotoxic drugs and the observed association with AKI warrants further investigation. Statistical adjustments for multiple potential confounders is needed in order to assess a potential causal relationship which would lay foundations for interventional studies.

**Competing interests** None.

#### S24 Relationship between ionized calcium and its determinants in septic shock

##### Nicolas Chudeau^1^, Tommy Raveau^2^, Valérie Moal^3^, Alain Mercat^4^, Pascal Houillier^5^, Nicolas Lerolle^2^

###### ^1^Réanimation médico-chirurgicale, C.H. - Le Mans, Le Mans, France; ^2^Réanimation médicale, Centre Hospitalier Universitaire d’Angers, Angers, France; ^3^Laboratoire de biochimie, C.H.U. d’Angers, Angers, France; ^4^Service de réanimation médicale et médecine hyperbare, Centre Hospitalier Universitaire d’Angers, Angers, France; ^5^Département de physiologie et radio-isotopes, Hôpital Européen Georges-Pompidou (AP-HP), Paris, France

####### **Correspondence:** Nicolas Chudeau - nico.chudeau@gmail.com

*Annals of Intensive Care* 2017, **7**(**Suppl 1**):S24

**Introduction** Hypocalcemia is a common disorder in ICU, especially in septic patients and it is associated with mortality. The mechanisms underlying this disorder are unknown. The aim of this study was to investigate the relationship between serum ionized calcium concentration and its determinants in septic shock patients and notably to study the kidney response to hypocalcemia.

**Patients and methods** This prospective, single-center, observational study was conducted in the medical ICU of the University Hospital of Angers, from May 2013 to September 2015. Two groups of patients were evaluated: a group of septic patients with [Ca^2+^] <1.10 mmol/L (hypocalcemia group) in whom physician in charge considered appropriate as routine care to perfuse calcium chloride (CaCl2) to treat hypocalcemia and a group of non-septic and normocalcemic patients hospitalized for acute respiratory failure (normocalcemia group). Exclusion criteria were: age <18 years old, pregnancy and lactation, chronic disorder of calcium or phosphate homeostasis, major hemodynamic instability, use of diuretics in the 24 h before inclusion, anuria or renal replacement therapy and refusal to participate. In the 24 h following ICU admission, calcium metabolism was compared in the two groups of patients (serum [Ca2 +], parathyroid hormone (PTH), 25 OH vitamin D, calcitriol, phosphorus, magnesium, creatinine and urinary calcium concentration). These assays were repeated in hypocalcemic patients after an intravenous infusion of 2 g of CaCl2 over 2 h. Action of PTH on the kidney was assessed by evaluating nephrogenous cyclic AMP.

**Results** 32 patients were included in the study, 19 in the hypocalcemia group and 13 in the normocalcemia group. Hypocalcemic patients had lower [Ca^2+^] than normocalcemic patients (1.03 [0.95–1.05] vs. 1.15 [1.12–1.20] mmol/L, p < 0.0001). In hypocalcemic patients, [Ca^2+^] was significantly increased by the end of the intravenous infusion of CaCl_2_ (1.19 [1.07–1.28] mmol/L, p < 0.001 vs. baseline). PTH was significantly increased in hypocalcemic in comparison to normocalcemic patients (60 [36–130] vs. 43 [25–59] pg/mL, p = 0.02) and significantly decreased after CaCl_2_ infusion (25 [10–60] pg/mL, p < 0.01 vs. baseline). No other calcium determinant (25 OH vitamin D, calcitriol, pH, phosphorus, magnesium) significantly differed between the two groups. PTH was negatively correlated with [Ca^2+^] (Rho = −0.6, p < 0.001). Urinary calcium excretion by 100 mL of glomerular filtrate (GF) was lower in hypocalcemic versus normocalcemic patients (0 [0–0] vs. 1.4 [0.15–5.85] µmol/100 mL GF, p < 0.001) reflecting an effective tubular reabsorption of calcium, but remained unchanged after CaCl_2_ load. Nephrogenous cAMP, although above upper limit range of normal values, was not significantly different in hypocalcemia group and normocalcemia group (3.09 [0.91–11.48] vs. 2.96 [1.51–11.54] nmol/100 mL GF, p = 0.63). Nephrogenous cAMP did not decrease significantly after CaCl_2_ load (1.7 [0.14–6.7] nmol/100 mL GF, p = 0.15 vs baseline).

**Conclusion** In septic shock patients with hypocalcemia, we observed an adequate PTH response to calcium variation and an efficient tubular calcium reabsorption. In the absence of increased nephrogenous cAMP, persisting tubular calcium reabsorption despite Ca load and PTH decrease, may suggest that the usual physiological link between PTH, nephrogenous cAMP and tubular reabsorption is altered in ICU patients. Thus, non-PTH factors drive tubular Ca reabsorption. Finally, our results show that hypocalcemia in septic patients is not explained by altered renal tubular calcium transport and a flux of calcium from serum to soft tissue may be hypothesized.

**Competing interests** None.

**References**

1. Collage RD, Howell GM, Zhang X, Stripay JL, Lee JS, Angus DC, and al. Calcium supplementation during sepsis exacerbates organ failure and mortality via calcium/calmodulin-dependent protein kinase signaling. Crit Care Med. 2003;41(11):e352–60.
2. Zaloga GP. Hypocalcemia in critically ill patients. Crit Care Med. 1992;20(2):251–62.

#### S25 Kidney cell-cycle arrest biomarkers change after iodinated contrast media infusion in critically ill patients

##### Emmanuelle Rouve^1^, Karim Lakhal^2^, Charlotte Salmon Gandonnière^3^, Youenn Jouan^3^, Laetitia Bodet-Contentin^3^, Stephan Ehrmann^3^

###### ^1^Réanimation polyvalente, CHU Bretonneau, Tours, France; ^2^Service de réanimation chirurgicale, Hôpital Guillaume et René Laënnec, CHU de Nantes, Nantes, France; ^3^réanimation polyvalente, CHRU Hôpitaux de Tours, Tours, France

####### **Correspondence:** Emmanuelle Rouve - emarouve@gmail.com

*Annals of Intensive Care* 2017, **7**(**Suppl 1**):S25

**Introduction** Iodinated contrast media is considered to be a frequent contributor to acute kidney injury (AKI) in hospitalized patients (contrast-associated AKI). Recently, the urinary G1 cell-cycle arrest proteins TIMP2 and IGFBP7 have been identified as potential key diagnostic markers of AKI. However, the impact of iodinated contrast media, frequently administered to intensive care unit (ICU) patients on those kidney biomarkers has not been evaluated. The aim of this study was to evaluate TIMP-2 and IGFBP-7 urinary concentrations variations before and after iodinated contrast media infusion for a computed tomographic scan in ICU patients. This setting represents a unique situation of a calibrated kidney aggression in a population of patients at increased risk of AKI.

**Materials and methods** We prospectively enrolled patients undergoing their first contrast media-enhanced computed tomographic scan during their stay in two ICUs. Measurements of urinary [TIMP-2]*[IGFBP7] was performed before and 6 h after iodinated contrast infusion. Patients were followed 5 days for occurrence of AKI (KDIGO classification).

**Results** We included 77 patients. Before contrast administration, 42 (54.5%) had no AKI, whereas 20 (26%) had stage 1 and 15 (19%) stage 2 or 3 AKI. Median [TIMP-2]*[IGFBP7] before contrast was 0.07 [interquartile range (IQR) 0.04–0.27] and individual change 6 h later was −0.01 [−0.11; 0.11] (ng/ml)^2^/1000, p > 0.1. Fourteen (18%) patients increased their serum creatinine of more than 26.4 µmol/L and 30 (41%) increased their KDIGO classification within 72 h after contrast infusion, indicating possible contrast-associated AKI. [TIMP-2]*[IGFBP7] change over 6 h was not significantly different among patients developing or no contrast-associated AKI, whatever the definition. Sixteen (21%) patients had a significant increase (≥0.2 (ng/ml)^2^/1000) in [TIMP-2]*[IGFBP7] 6 h after contrast. Among them, 11 (69%) increased their KDIGO score within 72 h of contrast administration, whereas only 26 (43%) of patients with lower values of [TIMP-2]*[IGFBP7] did so.

**Conclusion** Changes in urinary concentration of [TIMP-2]*[IGFBP7] within 6 h of contrast infusion for a computed tomographic scan were modest. Hypotheses advanced for this finding would be (1) the minimal kidney aggression by current monomeric nonionic low-osmolar contrast media, late serum creatinine increase being explained by the occurrence of later (between the 6th and the 72nd hour) kidney injury due to critical illness or its therapy or (2) insufficient sensitivity of early (6 h) measurements of this biomarker to detect contrast-associated AKI.

**Competing interests** partial financial support, no implication in data analysis and interpretation.

#### S26 Ketoacidosis: a prognosis and therapeutic study

##### Adrien Balmier^1^, Jonathan Messika^2^, Etienne De Montmollin^3^, Victorine Pouyet^4^, Benjamin Sztrymf^5^, Bruno Megarbane^6^, Abirami Thiagarajah,^7^, Jean-Damien Ricard^8^, Didier Dreyfuss^9^, Damien Roux^1^

###### ^1^Réanimation médico-chirurgicale, Hôpital Louis-Mourier - APHP, Colombes, France; ^2^Service de réanimation médico-chirurgicale, CHU Louis Mourier, Colombes, Colombes, France; ^3^Réanimation, C.H. Général Saint Denis hôpital Delafontaine, Saint-Denis, France; ^4^Réanimation médico-chirurgicale, René Dubos, Pontoise, France; ^5^Réanimation polyvalente, Hôpital Antoine Béclère, Clamart, France; ^6^Service de Réanimation Médicale et Toxicologique, CHU Lariboisière, Paris, France; ^7^Réanimation médico-chirurgicale, René Dobus, Pontoise, France; ^8^Service de Réanimation Médico-Chirurgicale, CHU Louis Mourier, Colombes, France; ^9^Inserm, iame, umr 1137, Université Paris Diderot, Sorbonne Paris Cité, Paris, France

####### **Correspondence:** Damien Roux - damien.roux@aphp.fr

*Annals of Intensive Care* 2017, **7**(**Suppl 1**):S26

**Introduction** Diabetic ketoacidosis, generally resulting from an absolute deficiency of insulin, is a frequent cause of hospitalization in intensive care unit. Recommendations for diagnosis of diabetic ketoacidosis, care and site of admission have been published by the English society of diabetology. ICU admission are recommended if one of the following criteria is present: GCS < 12, systolic arterial pressure (SAP) <90 mmHg, SpO_2_ < 92%, ketosis >6 mmol/L, HCO_3_ < 5 mmol/L, pH < 7.1, potassium level < 3.5 mmol/L or anion gap >16 mmol/L. However, it is suspected that adhesion to recommendations remains low.

In this study, we aimed at describing patients admitted for diabetic ketoacidosis in ICU. We looked at adhesion to published recommendations regarding admission and care. We also described metabolic complications and looked for an association between complications and dose of initial insulin therapy.

**Patients and methods** We performed a 2-years retrospective analysis of patients admitted for diabetic ketoacidosis within five ICU in Paris area. Clinical and biological characteristics at admission, treatment and complications during ICU stay were recorded. Categorical data are presented as % and continuous data as median [IQ25–IQ75]. Correlation between hypokalemia and hypoglycemia with insulin dosage was performed using a Spearman test.

**Results** Among the 118 patients admitted in 2013–2014 for diabetic ketoacidosis, 111 (94%) were admitted from home. Median age was 42 years old. Discontinuation of insulin therapy was the main cause of ketoacidosis (37%), whereas this condition revealed diabetes in 30% of patients. Sepsis was responsible for 19% of the cases. One death (0.8%) was observed over the 2 years study.

Despite convincing data on reliability of venous blood gas for ketoacidosis evaluation, arterial blood gas was performed before or at ICU admission in 92% of patients without oxygen desaturation.

At admission, 111/118 patients presented at least a criterion for ICU admission based on British recommendations. A GCS < 12 was observed in 11% of patients, SAP < 90 mmHg in 13%, SpO2 < 92% in 9%, HCO3 < 5 mmol/L in 34%, pH < 7.1 in 37%, K + < 3.5 mmol/L in 9% and anion gap >16 in 93%. Among the 7 patients that did not meet the criteria, four were admitted in ICU for other medical conditions such as hematemesis, acute kidney injury or pneumonia. One single patient, pregnant and suffering from hyperemesis gravidarum had a glycemia below 11 mmol/L at admission.

**Treatment** At diagnosis of ketoacidosis, 11 patients received sodium bicarbonate, in which 7 received hypertonic sodium bicarbonate; 9 of them had either a pH < 7.0, EKG signs of hyperkalemia or hyperkalemia >5.0. Patients received a median of 3000 mL of NaCl 0.9% in the first 24 h. Initial insulin bolus of 10 UI [8–10] has been injected in 24% of patients. This was slightly correlated to hyperglycemia (Spearman test, p = 0.035) but not to pH or kalemia. A continuous infusion of intravenous insulin was started in all patients at a median of 7 UI/h [6–10].

**Complications** Hypoglycemia (<3.9 mmol/L) was observed in 48% of patients within the first 24 h in which 19% were <2.9 mmol/L. This was 47 and 15% of patients between 24 and 48 h of ICU stay. Hypokalemia below 3.5 mmol/L happened in 44% of patients within the first 24 h and in 52% between 24 and 48 h. Neither hypoglycemia nor hypokalemia were correlated with initial insulin bolus or initial dosage of continuous intravenous insulin. Hypophosphatemia <0.30 mmol/L was observed in 15% of patients.

**Discussion** In this study, admission to ICU was consistent with British recommendations since most patients presented at least one clinical or biological criterion indicating ICU admission. Arterial blood gas were sampled in the large majority of patients despite consistent data showing that venous blood gas might be sufficient in non-hypoxemic patients. Also, initial insulin bolus and sodium bicarbonate perfusion were performed in a significant subset of patients despite absence of convincing data or recommendations supporting their use.

Finally, significant hypokalemia and hypoglycemia were frequent in these patients. These complications are in theory favored by insulin therapy but we did not observe a correlation between administration of an insulin bolus or the dose of continuous intravenous insulin perfusion.

**Conclusion** In this retrospective multicentre study, patients admitted in ICU for diabetic ketoacidosis were correctly oriented regarding the British recommendations. Metabolic complications (hypoglycemia and hypokalemia) were frequent but not correlated with initial dose of insulin.

**Competing interests** None.

**References**

1. Ayed S, Bouguerba A, Ahmed P, Barchazs J, Boukari M, Goldgran-Toledano D, et al. Diabetic ketoacidosis traps. Réanimation. 2015;24(6):668–87.
2. Savage MW, Dhatariya KK, Kilvert A, et al. Joint British Diabetes Societies guideline for the management of diabetic ketoacidosis. Diabet Med. 2011;28:508–15.

#### S27 Correction rate of acute severe hypernatremia and outcome in septic shock patients resuscitated with crystalloids

##### Frédérique Schortgen^1^, Pierre Asfar^2^, Boisramé-Helms Julie^3^, Julien Charpentier^4^, Emmanuel Guérot^5^, Bruno Megarbane^6^, David Grimaldi^7^, Grelon Fabien^8^, Nadia Anguel^9^, Lasocki Sigismond^10^, Henry-Lagarrigue Matthieu^11^, Frédéric Gonzalez^12^, Legay François^13^, Christophe Guitton^14^, Maleka Schenck^15^, Doise Jean-Marc^16^, Didier Dreyfuss^17^, Peter Radermacher^18^, for the HYPER2S Investigators and REVA research network

###### ^1^Réanimation médicale, Hôpital Henri Mondor, Créteil, France; ^2^Réanimation, C.H.U. d’Angers, Angers, France; ^3^Réanimation médicale, Nouvel Hôpital Civil, Hôpitaux Universitaires de Strasbourg, Strasbourg, France; ^4^Réanimation Médicale, Hôpital Cochin, Paris, France; ^5^Réanimation médicale, Hopital Europeen Georges-Pompidou, Paris, France; ^6^Service de Réanimation Médicale et Toxicologique, CHU Lariboisière, Paris, France; ^7^Service de réanimation polyvalente, Centre Hospitalier de Versailles, Le Chesnay, France; ^8^Réanimation, C.H. - Le Mans, Le Mans, France; ^9^Réanimation médicale, CHU de Bicêtre, Le Kremlin Bicêtre, France; ^10^Réanimation chirurgicale, C.H.U. d’Angers, Angers, France; ^11^Réanimation polyvalente, Hospital Center Departmental De Vendée, La Roche-sur-Yon, France; ^12^Réanimation medico-chirurgicale, hopital avicenne, Bobigny, France; ^13^Réanimation, C.H. de Saint Brieuc, Saint-Brieuc, France; ^14^Réanimation médicale, C.H.U. Hôtel Dieu, Nantes, France; ^15^Réanimation médicale, C.H.R.U. Hôpitaux Universitaires Strasbourg, Strasbourg, France; ^16^Réanimation, C.H. Chalon sur Saône William Morey, Chalon-sur-Saône, France; ^17^Réanimation polyvalente, Hôpital Louis-Mourier (AP-HP), Colombes cedex, France; ^18^Institut für anästhesiologische pathophysiologie und verfahrensentwicklung, Universitätsklinikum Ulm, Ulm, Germany

####### **Correspondence:** Frédérique Schortgen - frederique.schortgen@aphp.fr

*Annals of Intensive Care* 2017, **7**(**Suppl 1**):S27

**Introduction** The appropriate rate for hypernatremia (H-Na) correction is unknown. Under-correction could be associated with worse outcome. Experts recommend a rapid correction of acute (<2 days) and sever (> 150 mmol/l) H-Na with a rate of −2 mmol/l/h until Na < 146 mmol/l (1). Correction should be, therefore, obtained within 24 h. In patients with septic shock resuscitated with iso- or hypertonic saline and who acquired acute severe H-Na, we assessed if the correction rate was associated with mortality.

**Patients and methods** Data are a post hoc analysis of the RCT “Hyper2S” database comparing normal to 3% saline for 72 h in septic shock. Serum Na (SNa) was measured at H0, every 12 h for 3 days and then daily until D7. Patients with severe H-Na and available SNa control within 24 h were included. SNa evolution was compared between survivors and non-survivors. Correction and over-correction were defined as SNa <146 and <135 respectively.

**Results** 94 patients were included with a median SNa of 153 (152–156) mmol/l. H-Na was corrected within 24 h in 17 (18%) patients with a correction rate of −0.17 (−0.29 to 0) mmol/l/h. H-Na corrected within 24 h was higher in non-survivors (24 vs 15%, p = 0.31) (table). H-Na correction rate was more rapid in non-survivors, p = 0.05 (Table 15). Over-correction occurred similarly in survivors (15%) and non-survivors (10%). The time to reach SNa normalization was shorter in non-survivors (p = 0.05). After adjustment for SAPSII and MacCabe scores, more rapid correction rate remained significantly associated with mortality: OR 0.16; 95% CI (0.03–0.86), p = 0.048.

**Table 15:** Characteristics and evolution of H-Na between D28 survivors and non-survivors (results are number and % of patients and median IQR)

	Survivors n = 65	Non-survivors n = 29	OR 95% CI, p
*Characteristics of hypernatremia*			
3% HT saline gp, n (%)	54 (83)	26 (89)	1.77 (0.45–6.88), 0.41
Max SNa, mmol/L	155 (152–157)	155 (152–157)	1.04 (0.94–1.15), 0.40
Max SNa gap, mmol/L	15 (12–20)	18 (12–22)	0.93 (0.86–1.01), 0.10
*Evolution of hypernatremia within 24* *h*			
H-Na corrected, n (%)	10 (15)	7 (24)	1.75 (0.59–5.2), 0.31
Correction rate, mmol/L/h	−0.17 (–0.25/0)	−0.25 (−0.42/− 0.13)	0.17 (0.03–0.99), 0.05
Lowest SNa, mmol/L	150 (147–153)	147 (146–142)	0.95 (0.87–1.03), 0.22
Over-corrected, n (%)	0	0	
*Final evolution of hypernatremia*			
Finally corrected, n (%)	57 (88)	20 (69)	0.31 (0.11–0.92), 0.03
Time to SNa <146, h	60 (36–84)	36 (24–48)	0.98 (0.96–1.00), 0.05
Over-corrected, n (%)	10 (15)	3 (10)	0.63 (0.16–2.50), 0.52

**Conclusion** In the context of acute severe H-Na induced by fluid resuscitation, a rapid correction rate might be associated with even aggravated rather than improved mortality.

**Competing interests** None.

**Reference**

1. Sterns RH. N Engl J Med. 2015;372:55–65.

#### S28 Myocardial dysfunction in severe attacks of Systemic Capillary Leak Syndrome

##### Marc Pineton De Chambrun^1^, Charles-Edouard Luyt^1^, François Beloncle^2^, Nathalie Zapella^3^, Jean-Paul Mira^4^, Stanislas Ledochowsky^5^, Nicolas Terzi^6^, Jean-Marc Mazou^7^, Romain Sonneville^8^, Sylvie Paulus^9^, Yannick Fedun^10^, Anne-Sophie Moreau^11^, Mickael Landais^12^, Antoine Vieillard-Baron^13^, Jean-Herlé Raphalen^14^, Laurent Argaud^15^, Alain Combes^1^, Zahir Amoura^16^, for the EurêClark study group

###### ^1^Service de réanimation médicale, Groupe Hospitalier La Pitié-Salpêtrière, Institut de Cardiométabolisme et Nutrition, Assistance Publique Hôpitaux de Paris, Paris, France; ^2^Département de réanimation médicale et de médecine hyperbare, C.H.U. d’Angers, Angers, France; ^3^Service de réanimation médico-chirurgicale, Centre Hospitalier de Versailles, Le Chesnay, France; ^4^Service de réanimation médicale, Hôpital Cochin, Assistance Publique Hôpitaux de Paris, Paris, France; ^5^Service de réanimation médicale, C.H. Lyon Sud, Pierre-Bénite, France; ^6^Service de réanimation médicale, Clinique de Réanimation Médicale, Grenoble, France; ^7^Service de réanimation polyvalente, C.H. de Dax - Côte d’Argent, Dax, France; ^8^Service de réanimation médicale et infectieuse, Hôpital Bichat-Claude Bernard, Assistance Publique Hôpitaux de Paris, Paris, France; ^9^Service de réanimation chirurgicale, Hopital Louis Pradel, Bron, France; ^10^Service de réanimation polyvalente, Centre Hospitalier Bretagne Atlantique, Vannes, France; ^11^Service de réanimation médicale, C.H. Régional Universitaire de Lille, Lille, France; ^12^Service de réanimation médicale, CHU Hôtel-Dieu Nantes, Nantes, France; ^13^Réanimation médico-chirurgicale, Assistance Publique - Hôpitaux de Paris, Hôpital Ambroise Paré, Boulogne-Billancourt, France; ^14^Service de réanimation adulte, Hôpital Necker, Assistance Publique Hôpitaux de Paris, France; ^15^Service de réanimation médicale, Hospices Civils de Lyon - Groupement Hospitalier Edouard Herriot, Lyon, France; ^16^Service de médecine interne, Groupe Hospitalier La Pitié-Salpêtrière, Institut IE3 M, Assistance Publique Hôpitaux de Paris, Paris, France

####### **Correspondence:** Marc Pineton De Chambrun - marc.dechambrun@gmail.com

*Annals of Intensive Care* 2017, **7**(**Suppl 1**):S28

**Introduction** Systemic Capillary Leak Syndrome (SLCS) is a rare disease characterized by recurrent life-threatening attacks of capillary hyper permeability in the presence of a monoclonal gammopathy (MG). During acute episodes, the leak of fluid and proteins from the intravascular compartment to the interstitium results in clinical signs of both acute hypovolemia and interstitial edema. Biological profile is pathognomonic with marked hemoconcentration and paradoxal hypoproteinemia. Hypovolemic shock is the classical feature of severe SCLS attacks. However, beside this typical hemodynamic profile, several case report described myocardial dysfunction during SCLS attacks. The objectives of this study were to assess frequency, characteristics and outcome of myocardial involvement during severe SCLS attacks.

**Patients and methods** Multicenter retrospective analysis of data from the European Clarkson registry (EurêClark). Criteria to retain SCLS diagnostic were; presence of a MG; ≥1 typical attack with clinical manifestations of hypovolemia and capillary leak; hemoconcentration with paradoxal hypo protidemia; exclusion of secondary capillary leak syndrome causes. Patients with severe attacks admitted in ICU were identified in EurêClark registry. Physician were contacted and offered to include the attack using a pre-established case report form. Categorical variables are expressed: n (%) and continuous variable: mean ± SD or median [IQR].

**Results** Between May 1992 and February 2016, 64 attacks in 39 patients have been included. In 11 (28%) patients, and 14 (22%) attacks, a myocardial involvement was reported. Sex ratio was 1.2 with an age of 48.2 ± 13 years. Ten (91%) patients had an IgG MG with Kappa light chain in 7 (70%) patients. Eight (57%) patients were admitted in ICU for cardiogenic shock. SAPS II score at admission was 62 [49.2–73] and SOFA score 9.5 [5.7–13]. Admission hemoglobin, protidemia and serum creatinine were 19.1 [17.3–21.5] g/dL, 44 [28.7–52.5] g/L and 228 [120–294] µmol/L, respectively. Echocardiography (TTE) was performed in all 14 patients. TTE reveled global hypokinesia in 10 (71%) patients, focal hypokinesia or dyskinesia in 3 (21%) and ventricular hypertrophy without hypokinesia in 1 (7%). Left ventricular myocardial dysfunction was present in 13 (93%) patients with median left ventricular ejection fraction (LVEF): 15 [5–40] %. Pericardial effusion was reported in 7 (50%) patients. Transient ventricular hypertrophy mimicking hypertrophic cardiomyopathy was reported in 3 (21%) patients. LVEF returned to normal in all survivors on follow-up TTE. Troponin was elevated in 12/13 (92%) patients (one missing data). Twelve patients (85%) required vasopressive amines, 9 (64%) mechanical ventilation, 6 (43%) renal replacement therapy, 5 (36%) veno-arterial extracorporeal membrane oxygenation, 1 (9%) intra-aortic balloon pump and 1 (9%) an IMPELLA. Compartment syndrome occurred in 5 (45%) patients and 4 (36%) died in ICU. We then compared the 11 patients with myocardial involvement to the 28 without Clinical and biological manifestations were similar in between groups. However, chest pain (54 vs 11%, p = 0.008), dyspnea (82 vs 39%, p = 0.03) and respiratory failure (54 vs 18%, p = 0.044) were more frequent in patients with myocardial involvement than in others. There was no difference between groups regarding treatment received in ICU, complication and outcome except for the use of VA-ECMO (36.4 vs 0%, p = 0.004).

**Conclusion** Myocardial involvement seems frequent in patients with severe SCLS attack, occurring in 22% of the cases. Such patients exhibited classical features of SCLS attacks. Myocardial involvement was responsible for altered LVEF or transient ventricular hypertrophy. Myocardial dysfunction could be severe, even requiring mechanical circulatory support. SCLS attacks should be known as a cause of severe reversible myocardial dysfunction and hypertrophy.

**Competing interests** None.

#### S29 Activated Clotting Time (ACT) versus AntiXa activity for monitoring anticoagulation in ECMO patients: concordance study

##### Aemilia Jacquemin^1^, Jean-Marie Conil^1^, Felipe Guerrero^2^, Bertrand Marcheix^3^, Nicolas Hernandez^1^, Olivier Fourcade^4^, Bernard Georges^5^, Clément Delmas^6^

###### ^1^Réanimation polyvalente, Hopital Rangueil, Toulouse, France; ^2^Laboratoire d’hémostase, Hopital Rangueil, Toulouse, France; ^3^Chirurgie cardio-vasculaire, Hopital Rangueil, Toulouse, France; ^4^Réanimation purpan, CHU Toulouse, Toulouse, France; ^5^Réanimation polyvalente, Hopital Rangueil, Avenue du Professeur Jean Poulhes, Toulouse, France, Toulouse, France; ^6^Cardiologie, Hopital Rangueil, Toulouse, France

####### **Correspondence:** Aemilia Jacquemin - aemilia.j@gmail.com

*Annals of Intensive Care* 2017, **7**(**Suppl 1**):S29

**Introduction** In refractory cardiorespiratory emergencies, ECMO appears a good alternative to conventional treatment. Its extracorporeal circuit justifies curative anticoagulation explaining haemorrhagic and thrombotic complications. Activated Clotting Time (ACT) is empirically and commonly used to assess anticoagulation but with large inter and intra-individual variabilities. In practice, antiXa activity dosage is available to approach anticoagulant effect of heparin and is less expensive, but data during ECMO are missing. We sought to demonstrate the lack of correlation between antiXa and ACT in patients under ECMO support.

**Patients and methods** We prospectively include patients supported by ECMO in CHU Toulouse, France, between 01/2014 and 04/2015 for circulatory/respiratory support. Anticoagulation was achieved by unfractionated heparin: initial bolus then continuous intravenous infusion (800–1200 IU/h), for antiXa target of 0.2–0.4. Concomitant dosing of antiXa (laboratory) and ACT (Hemocron^®^) was conducted two times a day on the same sample throughout the ECMO period. Relationship between ACT and antiXa was analyzed by Spearman correlation (Rho). After transformation into categorical variables (obtained target = 1; outside the target = 0), analyzes were completed by a concordance study (Kappa). As recognized on literature ACT’s targets were between 180 and 220.

**Results** 65 patients were included: 46 men (72%), median age 55 yo (53–57). Indications were veno-arterial (n = 42) and veno-venous ECMO (n = 23). ECMO median duration was 5 days (hours to 30 days). Spearman correlation test found low and inconsistent correlation between antiXa and ACT (Rho Spearman < 0.4). This correlation lack present from the day one, worsens over time. Analyzed Kappa showed no discrepancy between the areas “targets” of ACT and AntiXa confirming the results (Table 16).

**Table 16:** ACT—AntiXa concordance analysis by Kappa coefficient study for the first 5 days of ECMO

Morning	J1	J2	J3	J4	J5
ACT	192	196	194	196	194
Median [IC95%]	[181 to 200]	[191 to 204]	[186 to 201]	[183 to 210]	[187 to 207]
Anti Xa	0.21	0.25	0.18	0.21	0.21
Median [IC95%]	[0.17 to 0.34]	[0.20 to 0.32]	[0.14 to 0.23]	[0.17 to 0.25]	[0.16 to 0.24]
Kappa	0.085	0.082	0.069	−0.238	0.115
[IC95%]	[−0.149 to 0.320]	[−0.150 to 0.315]	[0.172 to 0.310]	[−0.530 to 0.054]	[−0.189 to 0.419]
Evening					
ACT	195	193	195	196	195
Median [IC95%]	[190 to 205]	[188 to 200]	[189 to 197]	[185 to 204]	[180 to 199]
Anti Xa	0.23	0.19	0.17	0.22	0.19
Median [IC95%]	[0.21 to 0.30]	[0.13 to 0.26]	[0.13 to 0.21]	[0.19 to 0.26]	[0.14 to 0.25]
Kappa	0.034	0.093	0.154	0.208	0.062
[IC95%]	[−0.198 to 0.267]	[−0.113 to 0.301]	[−0.075 to 0.383]	[−0.093 to 0.509]	[−0.283 to 0.408]

**Conclusion** Use of ACT for ECMO anticoagulation monitoring doesn’t seem appropriate and high price probably justifies preferential use of antiXa in clinical practice. Analyzes of relationships between antiXa and bleeding/thrombotic events are needed to confirm the antiXa place and its target in these indications.

**Competing interests** None.

**References**

1. Chu DC, Abu-Samra AG, Baird GL, Devers C, Sweeney J, Levy MM, et al. Quantitative measurement of heparin in comparison with conventional anticoagulation monitoring and the risk of thrombotic events in adults on extracorporeal membrane oxygenation. Intensive Care Med. 2015;41(2):369–70.
2. ELSO guidelines for cardiopulmonary extracorporeal life support, version 1.3. Ann Arbor: Extracorporeal Life Support Organization; 2013.

#### S30 Outcome, long-term quality-of-life, physical and psychological assessment of patients supported by extracorporeal life support (ECLS) for postcardiotomy refractory cardiogenic shock

##### Sarah Makoudi^1^, Audrey Genton^1^, Rémy Bernard^1^, Adrien Bouglé^1^, Guillaume Lebreton^2^, Julien Amour^1^

###### ^1^Département d’anesthésie et de réanimation, Hôpital Universitaire La Pitié-Salpêtrière, Paris, France;^2^ Service de chirurgie thoracique et cardiovasculaire, Groupe Hospitalier Pitié Salpêtrière, Paris, France

####### **Correspondence:** Sarah Makoudi - smmakoudi@gmail.com

*Annals of Intensive Care* 2017, **7**(**Suppl 1**):S30

*Annals of Intensive Care* 2017, **7**(**Suppl 1**):S20

**Introduction** Postcardiotomy cardiogenic shock (CS) has an incidence of 2% to 6% after routine adult cardiac surgery. In 0.5–1.5% of cases, an venoarterial extracorporeal life support (VA-ECLS) is requested. The 6-month survival rate is 17.6% (1). Survivors may suffer of physical and psychological impairments as well as an alteration of quality of life. This study was designed to assess the outcomes, long-term health-related quality-of-life (HRQL) and occurrence of anxiety, depression and post-traumatic disorder symptoms in patients treated by VA-ECLS in postcardiotomy refractory shock.

**Patients and methods** All patients treated with VA-ECLS in postcardiotomy refractory CS between June 2014 and December 2015 are included in this study. Baseline patient characteristics and ICU events were retrospectively analyzed based on patient’s medical records. Long-term outcomes were assessed prospectively by the mean of a telephone-based questionnaire including French SF-36 quality-of-life assessment, Post-Traumatic Stress Scale 10, Hospital Anxiety and Depression Scale and assessment of function, morbidity, resource use, return to work and usual activities.

**Results** At all, 78 patients were included. At all, 35 patients (45%) survived to hospital discharge., 5 died after hospital discharge and 26 answered the telephone-based questionnaire between July and September 2016 (median follow-up: 512 days). SF-36 evaluation revealed impaired HRQL compared to the French general population tested by Leplège (2) especially concerning items related to physical health and social functioning. Patients had significantly lower mean scores for Physical functioning (p = 0.0004), Role physical (p = 0.0005), General Health (p = 0.02) and Social functioning (0.01) items. Mean physical and mental scores were at 57 ± 27 and 64 ± 23 respectively. Anxiety, depression and/or post-traumatic stress disorder symptoms occur in 30, 15 and 19% of the patients, respectively. Since ICU discharge, 65% of patients reported physical sequelae., ECLS-related limb pain occurs in 38% of patients while paresthesia occurs in 23% and chronic-tiredness in 69%. Mean Karnofsky score was 68% (Table 17).

**Table 17:** See text for description

Scores: mean ± SD	Survivors	Control (2)	p
SF-36: Physical score	57.5 ± 27.5		
PF (Physical Functioning)	61.3 ± 31.3	86.5 ± 19.7	<0.0004
RP (Role-Physical)	42.3 ± 48.8	80.6 ± 33.6	<0.0005
BP (Bodily Pain)	69.1 ± 19.7	74.3 ± 25.2	0.1926
GH (General Health)	58.7 ± 20.6	69 ± 20.9	<0.0178
SF-36: Mental score	64.4 ± 22.7		
VT (Vitality)	51.9 ± 21.5	61.4 ± 20.3	<0.0333
SF (Social Functioning)	68.8 ± 23.3	80.7 ± 22.5	<0.0153
RE (Role-Emotional)	66.6 ± 47.1	80.9 ± 34.1	0.1368
MH (Mental Health)	70.2 ± 13.2	67.8 ± 19.7	0.3627
PTSS-10 (N < 35)	22 ± 9		
HAD-Anxiety Score (N < 7)	6 ± 3		
HAD-Depression Score (N < 7)	4 ± 3		

**Conclusion** After VA-ECLS for postcardiotomy cardiogenic shock long-term physical and psychological sequelae are frequent in survivor patients, impacting their long term quality-of-life. These results support the necessity to develop and organise a systematic post-ICU health care.

**Competing interests** None.

**References**

1. Rastan AJ. Early and late outcomes of 517 consecutive adult patients treated with extracorporeal membrane oxygenation for refractory postcardiotomy cardiogenic shock. J Thorac Cardiovasc Surg. 2010;139:302–11.
2. Leplège A. The French SF-36 Health Survey: translation, cultural adaptation and preliminary psychometric evaluation. J Clin Epidemiol. 1998;51:1013–23.

#### S31 How to predict weaning success during ECLS support?

##### Charlotte Mazet^1^, Jean-Marie Conil^2^, Bernard Georges^3^, Nicolas Hernandez^2^, Fanny Bounes^3^, Gurbuz Murat^4^, Laure Cronier^2^, Guillaume Robin^2^, Caroline Biendel^2^, Stein Silva^5^, Clément Delmas^4^

###### ^1^CHU Toulouse, Toulouse, France, Toulouse, France; ^2^Réanimation polyvalente, Hopital Rangueil, Toulouse, France; ^3^Réanimation polyvalente, Hopital Rangueil, Avenue du Professeur Jean Poulhes, Toulouse, France, Toulouse, France; ^4^Cardiology, Hopital Rangueil, Toulouse, France; ^5^Réanimation, Centre Hospitalier Universitaire Toulouse, Toulouse, France

####### **Correspondence:** Charlotte Mazet - charlottemazet@hotmail.com

*Annals of Intensive Care* 2017, **15**(**Suppl 1**):S31

**Introduction** Datas concerning extracorporeal life support (ECLS) weaning are scarce. Nevertheless, early weaning or unjustified maintenance of such support can lead to own morbidity and mortality. This study would like to determine clinical, biological and echocardiographic predictive criteria for weaning success.

**Patients and methods** The pump flow of patients, considered as stabilized, was decreased to less than 2 L/min during 60 min. A transthoracic echocardiography and a biological test were made before and after the weaning test. Weaning decision was under the responsibility of the intensivist in charge of the patient. Objective of the study was to determine echocardiographic, biological and clinical prognostic value compare to clinical decision.

**Results** 39 tests have been performed on 31 patients between 05.2014 and 07.2016. Patients were essentially men (n = 21, 68%), median age of 46 years old (35–57) with classical cardiovascular risk factors. Indications of ECLS were shared between cardiogenic shock (n = 22, 71%) and cardiac arrest (n = 9, 29%). Five parameters allowed to distinguish patients able to be weaned or not (see Table 18 below): age (threshold: 51 years old), BMI (threshold: 28.6), lactate clearance between ECLS cannulation and test (threshold: 77%), inotrope time perfusion during weaning (threshold: 7 days) and LVEF stability or slightly rising for patients able to support weaning (threshold −2%).

**Table 18:** Area under the curve of continuous variables distinguished patients able to be weaned or not

	AUC	CI 95%	p	Threshold	Se	Sp	PPV	NPV
Age (years)	0.738	0.57–0.87	0.0037	≤51	66.67	86.7	88.9	61.9
BMI	0.855	0.7–0.95	<0.0001	≤28.6	80.9	85	89.5	75
Inotrope time perfusion during weaning (days)	0.896	0.75–0.97	<0.0001	≤7	82.6	80	86.4	75
Lactate clearance between ECLS cannulation and test (%)	0.857	0.67–0.96	<0.0001	>76.9	73.7	88.9	93.3	61.5
LVEF after test–LVEF before test (%)	0.753	0.55–0.89	0.0086	>−2	83.33	55.56	78.9	62.5

*AUC* area under the curve, *CI 95%* confidence interval 95%, *Se* sensitivity, *Sp* specificity, *PPV* positive predictive value (probability of death at 1 month when the test is positive), *NPV* negative predictive value (probability of survival at 1 month when the test is negative)

Furthermore, we developed a clinical and biological model for weaning. Patients with less than 7 days inotrope perfusion during weaning and with LVEF stabilized during test had 88.2% probability to be weaned of ECMO after the test.

**Conclusion** A simple multi-parametric result of the weaning trial could help the difficult ECLS weaning decision. To confirm these data, complementary multi-centric studies should be performed.

**Competing interests** None.

**References**

1. Cavarocchi NC, Pitcher HT, Yang Q, Karbowski P, Miessau J, Hastings HM, Hirose H. Weaning of extracorporeal membrane oxygenation using continuous hemodynamic transesophageal echocardiography. J Thorac Cardiovasc Surg. 2013;146:1474–79.
2. Aissaoui N, Luyt CE, Leprince P, Trouillet JL, Léger P, Pavie A, Diebold B, Chastre J, Combes A. Predictors of successful extracorporeal membrane oxygenation weaning after assistance for refractory cardiogenic shock. Intensive Care Med. 2011;37:1738–45.

#### S32 Impact of fluid balance on mortality of patients treated with veno-arterial extra corporeal membrane oxygenation

##### Samia Boubeche^1^, Caroline Abriou^1^, Véronique Wurtz^1^, Vincent Scherrer^1^, Nathalie Rey^1^, Gioia Gastaldi^2^, Benoit Veber^3^, Fabienne Tamion^4^, Fabien Doguet^5^, Arnaud Gay^5^, Bertrand Dureuil^6^, Emmanuel Besnier^1^

###### ^1^Department of anesthesia and critical care, Chu-Hôpitaux De Rouen, Rouen, France; ^2^Department of anesthesia and critical care, Centre Hospitalier Universitaire Rouen, Rouen, France; ^3^Reanimation chirurgicale, Centre Hospitalier Universitaire Rouen, Rouen, France; ^4^Réanimation médicale, Hospital Center University Rouen, Rouen, France; ^5^Department of cardiac surgery, Chu-Hôpitaux De Rouen, Rouen, France; ^6^Anesthésie, Centre Hospitalier Universitaire Rouen, Rouen, France

####### **Correspondence:** Samia Boubeche - samia.boubeche@hotmail.fr

*Annals of Intensive Care* 2017, **7**(**Suppl 1**):S32

**Introduction** Veno-Arterial Extra Corporeal Membrane Oxygenation (VA ECMO) is a mechanical circulatory support used for critical patients with cardiogenic shock refractory to conventional therapies. Nevertheless, mortality observed for these patients remains very high. Contrary to others critical situations, such as sepsis or ARDS, the impact of fluid balance is little studied, whereas these patients also present and intense inflammatory stress and often required large amount of fluid resuscitation. The purpose of this study was to identify fluid balance as a predictor for mortality in patients under VA ECMO.

**Patients and methods** We conducted a single-center, retrospective and observational investigation in the cardiac surgical ICU of Rouen University Hospital. Patients requiring VA ECMO between March 2013 and May 2016 were included. Pregnant women, patient under 16 years old, patients deceased before 24 h of admission in ICU and ECMO for ARDS were excluded. The primary outcome was the relationship between 1-day fluid balance and 28-day mortality. Secondary outcomes were the association between 28-day mortality and other biological and clinical characteristics after 1, 3 and 7 days in ICU: fluid balance, weight gain, RIFLE (Risk, Injury, Failure, Loss of kidney function, and End-stage kidney disease) score, use of Renal Replacement Therapy (RRT), troponins, bilirubin and transminases values. Univariate analysis was performed through a Chi-2 or fisher exact test for qualitative values and a Student t test for continuous variables. Statistically significant data were then included for multivariate time to death analysis (Cox model). P < 0.05 was consider as significant.

**Results** 88 patients were included (52 ± 14 years, 70% male). Overall 28-day mortality was 53%. Univariate analysis indicated that fluid balance and weight gain at 1-day were significantly higher for deceased patients (1099 ± 2608 vs 3419 ± 3216 mL, p < 0.0001 and 0.07 ± 2.21 vs 1.78 ± 2.85 kg, p = 0.0033, respectively). Troponins at admission were five fold higher in deceased patients (11,469 vs 2098 ng/L, p = 0.002). IGS-2 score was higher in deceased patients (79.1 ± 15.2 vs 60.5 ± 15.1, p < 0.0001). Deceased patients presented worse renal function with higher rifle score (93% of failure or injury status vs 73%, p = 0.009), lower MDRD score at day 1 (38 ± 20.8 vs 50 ± 25 mL/min/m^2^, p = 0.019) but no difference regarding need for RRT. Multivariate analysis wit Cox regression only identified IGS-2 score as an independent factor associated with 28-days mortality (HR 1.04 [1.017–1.063]).

**Discussion** Interest for fluid management is growing in critical patients. Nevertheless, no study has yet investigated its impact in selected patients with cardiogenic shock treated with VA ECMO. Our study suggested a possible association between fluid overload and mortality but lack the power to confirm these results with multivariate analysis.

**Conclusion** Fluid management is a key therapy during VA ECMO but fluid overload could be associated with worsen outcomes. Further studies with larger population are warranted before considering fluid restriction trials.

**Competing interests** None.

**References**

1. Esper SA, Levy JH, Waters JH, Welsby IJ. Extracorporeal membrane oxygenation in the adult: a review of anticoagulation monitoring and transfusion. Anesth Analg. 2014;118(4):731–43.
2. Massetti M, Tasle M, Le Page O, Deredec R, Babatasi G, Buklas D, et al. Back from irreversibility: extracorporeal life support for prolonged cardiac arrest. Ann Thorac Surg. 2005;79(1):178–83; discussion 183–4.
3. Koning NJ, Vonk ABA, Meesters MI, Oomens T, Verkaik M, Jansen EK, et al. Microcirculatory perfusion is preserved during off-pump but not on-pump cardiac surgery. J Cardiothorac Vasc Anesth. 2014;28(2):336–41.
4. Kurundkar AR, Killingsworth CR, McIlwain RB, Timpa JG, Hartman YE, He D, et al. Extracorporeal membrane oxygenation causes loss of intestinal epithelial barrier in the newborn piglet. Pediatr Res. 2010;68(2):128–33.
5. Hoefeijzers MP, ter Horst LH, Koning N, Vonk AB, Boer C, Elbers PWG. The pulsatile perfusion debate in cardiac surgery: answers from the microcirculation? J Cardiothorac Vasc Anesth. 2015;29(3):761–7.
6. Zangrillo A, Landoni G, Biondi-Zoccai G, Greco M, Greco T, Frati G, et al. A meta-analysis of complications and mortality of extracorporeal membrane oxygenation. Crit Care Resusc J Australas Acad Crit Care Med. 2013;15(3):172–8.
7. Schmidt M, Bailey M, Kelly J, Hodgson C, Cooper DJ, Scheinkestel C, et al. Impact of fluid balance on outcome of adult patients treated with extracorporeal membrane oxygenation. Intensive Care Med. 2014;40(9):1256–66.
8. Neyra JA, Li X, Canepa-Escaro F, Adams-Huet B, Toto RD, Yee J, et al. Cumulative fluid balance and mortality in septic patients with or without acute kidney injury and chronic kidney disease. Crit Care Med. 2016.
9. National Heart, Lung, and Blood Institute Acute Respiratory Distress Syndrome (ARDS) Clinical Trials Network, Wiedemann HP, Wheeler AP, Bernard GR, Thompson BT, Hayden D, et al. Comparison of two fluid-management strategies in acute lung injury. N Engl J Med. 2006;354(24):2564–75.
10. Kielstein JT, Heiden AM, Beutel G, Gottlieb J, Wiesner O, Hafer C, et al. Renal function and survival in 200 patients undergoing ECMO therapy. Nephrol Dial Transplant Off Publ Eur Dial Transpl Assoc Eur Ren Assoc. 2013;28(1):86–90.
11. Villa G, Katz N, Ronco C. Extracorporeal Membrane Oxygenation and the Kidney. Cardiorenal Med. 2015;6(1):50–60.
12. McILwain RB, Timpa JG, Kurundkar AR, Holt DW, Kelly DR, Hartman YE, et al. Plasma concentrations of inflammatory cytokines rise rapidly during ECMO-related SIRS due to the release of preformed stores in the intestine. Lab Investig J Tech Methods Pathol. 2010;90(1):128–39.
13. Aso S, Matsui H, Fushimi K, Yasunaga H. In-hospital mortality and successful weaning from venoarterial extracorporeal membrane oxygenation: analysis of 5263 patients using a national inpatient database in Japan. Crit Care Lond Engl. 2016;20:80.
14. Shimizu K, Ogura H. Is the 77.1% rate of in-hospital mortality in patients receiving venoarterial extracorporeal membrane oxygenation really that high? Crit Care Lond Engl. 2016;20(1):202.
15. Schmidt M, Burrell A, Roberts L, Bailey M, Sheldrake J, Rycus PT, et al. Predicting survival after ECMO for refractory cardiogenic shock: the survival after veno-arterial-ECMO (SAVE)-score. Eur Heart J. 2015;36(33):2246–56.
16. Combes A, Leprince P, Luyt C-E, Bonnet N, Trouillet J-L, Léger P, et al. Outcomes and long-term quality-of-life of patients supported by extracorporeal membrane oxygenation for refractory cardiogenic shock. Crit Care Med. 2008;36(5):1404–11.
17. Luyt C-E, Landivier A, Leprince P, Bernard M, Pavie A, Chastre J, et al. Usefulness of cardiac biomarkers to predict cardiac recovery in patients on extracorporeal membrane oxygenation support for refractory cardiogenic shock. J Crit Care. 2012;27(5):524.e7–14.
18. Lim W, Whitlock R, Khera V, Devereaux PJ, Tkaczyk A, Heels-Ansdell D, et al. Etiology of troponin elevation in critically ill patients. J Crit Care. 2010;25(2):322–8.

#### S33 Should we stop ECLS for futility for some patients after 24 h of support?

##### Nicolas Hernandez^1^, Jean-Marie Conil^1^, Bernard Georges^1^, Fanny Bounes^1^, Antoine Rouget^1^, Gurbuz Murat^2^, Stein Silva^3^, Clément Delmas^2^

###### ^1^Réanimation polyvalente, Hopital Rangueil, Toulouse, France; ^2^Cardiologie, Hopital Rangueil, Toulouse, France; ^3^Réanimation, Centre Hospitalier Universitaire Toulouse, Toulouse, France

####### **Correspondence:** Nicolas Hernandez - herni9@gmail.com

*Annals of Intensive Care* 2017, **7**(**Suppl 1**):S33

**Introduction** Extracorporeal life support (ECLS) has taken an important place in the treatment of cardiogenic shock (CS) or refractory cardiac arrest (CA). However, ECLS deplore a high mortality rate in the first days raising important ethic and economic consequences. In this context, continuation of support should be reassessed precociously. The aim of this study was the research of prognostic factors of 30-days mortality, 24 h after ECLS implantation for CS or CA.

**Materials and methods** All patients undergoing ECLS in our tertiary center during a 2-year period were prospectively included. The ECLS were managed with a multidisciplinary protocol based on consensus. Clinico-biological data were collected just before and 24 h after ECLS implantation. These data were compared between survivors and deceased at 1 month.

**Fig. 14:**
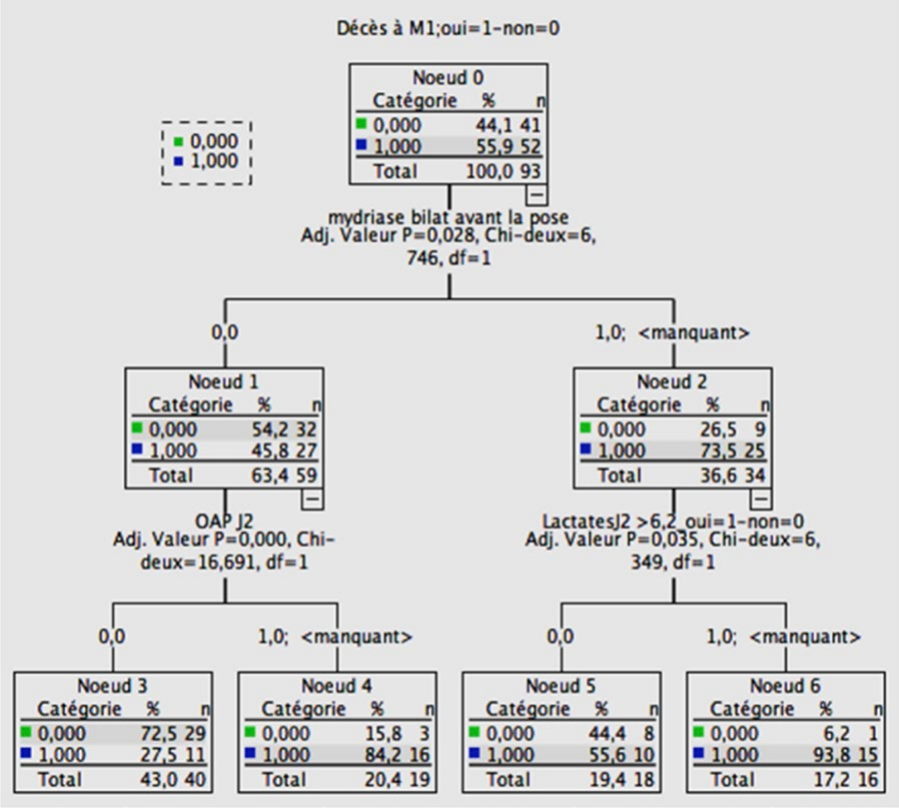
Representation of 30-days patients’ survival by the CHAID’s segmentation method using the variables of interest

**Results** 94 patients were included with a males’ predominance (66%) and a median age of 53 years. ECLS was implanted for CA under resuscitation (29.8%), acute CS (37.2%), CS after CA (22.3%) and CS due to end-stage heart failure (10.6%). 30-days mortality was 56.4%. Significant data were selected in univariate analysis. Thanks to a Cox model (survival model at 30 days), multivariate analysis showed the influence of bilateral mydriasis before ECLS (RR = 2.87, IC95% [1.34–6.07]; p = 0.006), arterial lactate rate >6.2 mmol/l 24 h after ECLS (RR = 4.04, IC95% [1.96–8.32]; p < 0.001) as well as acute pulmonary edema occurrence in the first 24 h after ECLS (RR = 2.37, IC95% [1.20–4.70]; p = 0.013). CHAID’s segmentation method represented the patients’ survival using these covariables with a prevision of 75.3% and may help to stop or maintain ECLS after 24 h of support (Fig. 14).

**Conclusion** Simple biological and clinical markers as initial mydriasis, lactate level at H24 and the occurrence of an acute pulmonary edema during the first 24 h could serve to predict 1-month mortality for patients under ECLS for CA or CS. At 24 h, simple parameters could raise the question to stop support.

**Competing interests** None.

**References**

1. Ponikowski P, Voors AA, Anker SD et al. ESC guidelines for the diagnosis and treatment of acute and chronic heart failure: The Task Force for the diagnosis and treatment of acute and chronic heart failure of the European Society of Cardiology (ESC). Developed with the special contribution of the Heart Failure Association (HFA) of the ESC. Eur Heart J. 2016.
2. Yancy CW, Jessup M, Wilkoff BL et al. ACCF/AHA guideline for the management of heart failure: a report of the American College of Cardiology Foundation/American Heart Association Task Force on practice guidelines. J Am Coll Cardiol. 2013;62(16):147–239.

#### S34 Prognosis at 6 and 12 months of patients treated by hyperbaric oxygenotherapy after self-attempted hanging

##### Guillaume Gantois^1^, Julien Poissy^1^, Erika Parmentier-Decrucq^1^, Raphaël Favory^1^, Daniel Mathieu^1^

###### ^1^Pôle de réanimation, hôpital salengro, C.H.R.U. - Lille, Avenue Oscar Lambret, Lille, France, Lille, France

####### **Correspondence:** Julien Poissy - julien_poissy@hotmail.fr

*Annals of Intensive Care* 2017, **7**(**Suppl 1**):S34

**Introduction** Patients surviving a self-attempted hanging present an anoxic encephalopathy which is recognized as an optional indication for hyperbaric oxygen (HBOT). Previous studies have shown a total neurological recovery in 57–77% of patients. Mortality risk factors of post-hanging patients have been identified, but long term morbidity prognostic factors have not. A follow-up study with neurological evaluation at 6 and 12 months have been undertaken to determinate those factors.

**Patients and methods** In this observational study, all patients hospitalized for post-hanging in ICU in a 5-year period were included. Prehospital and ICU data were collected. Neurological evaluation at 6 and 12 months was performed according to CPC scores. Factors associated with neurological recovery were determined by comparing CPC 2 + 3+4 (bad recovery) versus CPC 1 (good recovery). Qualitative variables were compared by X^2^ tests, and quantitative ones by Mann–Whitney. A p value less than 0.05 was considered as statistically significant.

**Results** 231 patients with a median age of 40.3 years [IQR 30–50.2] were included. 104 patients (47%) were found in cardiac arrest (CA). HBOT was performed in 95% of patients, with a median delay of 120 min [IQR 90–180]. 95 patients died in ICU (41%), 93 (89%) in CA group and 2 (1.6%) in the group without CA. Neurological evaluation at 6 and 12 months was obtained in 97 of the 136 alive patients. At 6 months, in the CA group (n = 9), CPC score was respectively 1 for 6 patients, 2 for 2, 4 for 1. At 12 months, CPC score changed only for the 2 patients with a CPC score at 2 (one died after another suicide attempt, one changed his CPC score to 3). In the group without CA (n = 88), 79 had normal neurological status at 6 months and 78 at 12 months (one patient died because of a cancer). Among these patients, 96% returned at home and 77% returned to work. 16 (18%) patients re-attempted suicide in the year. The major risk factor of mortality is the presence of a cardiac arrest on hanging site. All the other factors found to be related to mortality are well known risk factors in cardiac arrest of other origin. In univariate analysis, risk factors of neurological sequelae at 6 months were a cardiac arrest on hanging site (p = 0.045) an elevated diastolic blood pressure (87 vs 70 mmHg; p = 0.04), a lower initial Glasgow score (4 vs 5; p = 0.04), and an elevated blood glucose (1.39 vs 1.13 g/L p < 0.001) at admission in ICU.

**Discussion** Our cohort of self-hanging patients can be divided in two parts: a) patients with CA in the pre-hospital period with a high mortality and a good neurological recovery in 2/3 surviving patient, but with a small group; b) patients without CA with a very low mortality and a very good neurological recovery. These results seem to be better than in the most important cohort [1] published until now in self-hanging patients without CA and not treated by HBOT (mortality at 9.5% and 3.5% of poor neurological recovery).

**Conclusion** Patients surviving a self-attempted hanging who have not presented CA and treated by HBOT have mainly a good neurological outcome. Randomized control study should be undertaken to confirm HBOT effectiveness in that indication.

**Competing interests** None.

**Reference**

1. Salim A, Martin M, Sangthong B, Brown C, Rhee P, Demetriades D. Near-hanging injuries: a 10-year experience. Injury. 2006;37(5):435–39.

#### S35 Prognostic value of early intermittent electroencephalography in patients supported by venoarterial ECMO

##### Eric Magalhaes^1^, Ruben Wanono^2^, Roland Smonig^1^, Mathilde Lermuzeaux^1^, Jordane Lebut^1^, Andremont Olivier^1^, Claire Dupuis^1^, Aguila Radjou^1^, Bruno Mourvillier^1^, Mathilde Neuville^1^, Marie Pia D’ortho^2^, Lila Bouadma^1^, Anny Rouvel-Tallec^2^, Jean-François Timsit^1^, Romain Sonneville^1^

###### ^1^Department of intensive care medicine and infectious diseases, Hôpital Bichat-Claude Bernard-APHP, Paris, France; ^2^Physiology, Hôpital Bichat-Claude Bernard-AP-HP, Paris, France

####### **Correspondence:** Romain Sonneville - romain.sonneville@aphp.fr

*Annals of Intensive Care* 2017, **7**(**Suppl 1**):S35

**Introduction** Venoarterial extracorporeal membrane oxygenation (VA-ECMO) is increasingly used to treat refractory cardiogenic shock or cardiac arrest. Acute brain injury (i.e. ischemic stroke, haemorrhage and/or failure to awaken because of diffuse brain injury) may occur in up to 15% of patients on VA-ECMO and is associated with increased mortality and poor functional outcome in survivors. However, early indicators of neurological outcome are lacking in this population. We aimed to assess the prognostic value of early electroencephalography (EEG) alterations during VA-ECMO.

**Patients and methods** We conducted a prospective single-center study in the medical ICU of a university hospital on consecutive patients cannulated to VA-ECMO. A standardized clinical neurological evaluation including the RASS score, the GCS score, the Full Outline of UnResponsiveness (FOUR) score and brainstem reflexes was coupled to an intermittent EEG. EEG was recorded as soon as possible within the first 72 h after VA-ECMO cannulation. EEG characteristics were analyzed by a neurophysiologist who was blinded to the patient’s condition. A severely altered EEG pattern was defined as a predominant delta frequency, discontinuous, unreactive and/or an isoelectric background. The primary endpoint was poor neurological outcome, defined as the composite of death or acute brain injury on neuroimaging within 28 days. Data are presented as median (interquartile range) or number (percentage). False-positive rates (FPRs, corresponding to 1-specificity) of poor neurological outcome were calculated for each significant predictor, using an exact binomial 95% confidence interval (CI).

**Results** Sixty-nine (age 58 (50–67) years) patients with a SOFA score of 14 (13–17) were included. Main indications for ECMO were: post cardiac surgery (n = 25, 36%), terminal dilated cardiomyopathy (n = 12, 17%), and acute myocardial infarction (n = 11, 16%). Cardiac arrest before ECMO cannulation was noted in 20 (29%) patients. EEG was recorded 1 (1–2) days after VA-ECMO cannulation and 62 (90%) patients were sedated at time of EEG. At day 28, 46 (67%) had a poor outcome (n = 37 deaths and n = 9 patients alive with acute brain injury). In univariate analysis, a lower RASS score (p = 0.003), a lower FOUR score (p = 0.001), a lower score on the motor component of the Glasgow coma scale (p = 0.001), and a lack of cough reflex (p = 0.033) at the time of EEG were significantly associated with a poor outcome. A severely impaired EEG pattern or presence of a discontinuous background activity were also associated with a poor outcome (p = 0.015 and p = 0.002, respectively). Indicators of poor neurologic outcome are presented in the Table 19. Among all parameters, a discontinuous background activity was the only variable that constantly predicted poor outcome (false-positive poor outcome prediction rate of 0%, 95% CI 0–15%).

**Table 19:** Indicators of poor neurologic outcome for patients cannulated to VA-ECMO

Variable	False positive poor outcome prediction rate (%)	95% CI
Motor component of the GCS < 4	19	9–34
Absence of cough reflex	16	5–36
FOUR score < 6	12	4–26
Severely impaired EEG	13	3–34
Discontinuous EEG background	0	0–15

**Conclusion** Early intermittent EEG has a strong prognostic value for sedated patients on VA-ECMO. Presence of a discontinuous EEG background activity seems to be more accurate than clinical alterations to predict a bad neurologic outcome at 28 days.

**Competing interests** None.

#### S36 Transjugular intra hepatic porto systemic shunt (TIPS) placement induces modifications of cerebral multimodal MRI in cCirrhotic patients, even in the absence of development of hepatic encephalopathy

##### Marika Rudler^1^, Nicolas Weiss^2^, Vincent Perlbarg^3^, Damien Galanaud,^4^, Dominique Thabut^5^, Brain Liver Pitié-Salpêtrière Study Group (BLIPS)

###### ^1,^Hepatology and gastroenterology, Pitié-Salpêtrière Hospital, Paris, France; ^2^Unité de réanimation neurologique, Hôpital Pitié-Salpêtrière, Paris, France; ^3^Bioinformatics and biostatistics platform, ICM Institut du Cerveau et de la Moelle épinière, Paris, France; ^4^Neurological icu, Pitié-Salpêtrière Hospital, Paris, France; ^5^Brain Liver Pitié-Salpêtrière Study Group (BLIPS), Hôpital Pitié-Salpêtrière, Paris, France

####### **Correspondence:** Marika Rudler - marika_rudler@yahoo.fr

*Annals of Intensive Care* 2017, **7**(**Suppl 1**):S36

**Introduction** Hepatic encephalopathy (HE) may occur after transjugular intrahepatic porto systemic shunt (TIPS) placement. Multimodal MRI, combining morphologic sequences, diffusion tensor imaging (DTI), and H proton magnetic resonance spectroscopy (MRS), is modified in cirrhotic patients even in the absence of patent HE. Our aims were: (1) To assess if TIPS induces changes in multimodal MRI; (2) To correlate changes to the development of HE after TIPS.

**Patients and methods** All consecutive patients with cirrhosis and an indication for TIPS were prospectively screened. Exclusion criteria were: common counter-indication to MRI, active drinking, overt HE. Neurocognitive evaluation using Psychometric HE test score (PHES), were assessed at baseline at inclusion, at the day of MRI, and 3 months after TIPS placement. MRI combined morphologic sequences, DTI, and MRS. DTI data were processed with standard tools of FSL5 including eddy current correction and the tensor metrics (Fractional Anisotropy, FA and Mean Diffusivity, MD) computation. Averaged measures of FA and MD were calculated within the 48 regions of the ICBM-DTI-81 white-matter labels atlas.

**Results** 25 consecutive patients were prospectively analysed (age 57 ± 8, male gender 68%, Child-Pugh score 7.8 ± 1.6, MELD score 12 ± 4, cause of cirrhosis: OH/virus/others 16/4/5, indication for TIPS placement: Ascites/Varices secondary prophylaxis/other 20/3/2), HE status: no HE/minimal HE (MHE)/patent HE: 20/5/0. 8/25 patients developed HE after TIPS (1 MHE, 7 patent HE). Baseline metabolites were significantly different in patients with or without MHE before TIPS: decrease in myoinositol, choline and increase in glutamate/glutamine. MD and FA were similar according to the presence of MHE or not before TIPS. TIPS induced significant changes in MRS metabolites as compared to baseline: decrease in myoinositol, glycerophosphocholine, *N*-acetylasparte, increase in glutamate/glutamine. TIPS did neither modify FA nor MD. Baseline metabolites were not predictive of the development of HE after TIPS. However, baseline FA was significantly lower in patients who developed HE: in cingulate gyrus, in external capsule and in fronto-occipital regions.

**Conclusion** TIPS induced significant changes in cerebral metabolites, even in the absence of the development of HE. DTI findings before TIPS could help predicting the development of HE after TIPS. This finding could lead to a targeted prevention therapy in some patient that undergo TIPS placement.

**Competing interests** None.

#### S37 Septic cerebral thrombophlebitis: risk factors and outcome

##### Emna Rachdi^1^, Ghada Mhamdi^2^, Ahlem Trifi^1^, Rim Abdelmalek^2^, Sami Abdellatif^1^, Foued Daly^1^, Rochdi Nasri^1^, Hanene Tiouiri^2^, Salah Ben Lakhal^1^

###### ^1^Réanimation médicale, Centre hospitalier universitaire la Rabta, Tunis, Tunisia; ^2^Maladies infectieuses, centre Hospitalier universitaire la Rabta, Tunis, Tunisia

####### **Correspondence:** Emna Rachdi - e.rachdi@yahoo.fr

*Annals of Intensive Care* 2017, **7**(**Suppl 1**):S37

**Introduction** Cerebral thrombophlebitis (CTP) is a dramatic and potentially lethal disease. It occurs mostly in children and young adults. Septic thrombosis of the dural venous sinuses is a rare accounting for 0.5–1% of all strokes but often catastrophic complication of a variety of infectious processes. Bacterial meningitis and paranasal sinusitis may be complicated by superior sagittal sinus thrombosis, an entity associated with a mortality rate of nearly 80%. Although there is often no direct evidence that the thrombus itself is infected, cases of cerebral sinus thrombosis are presumed to be “septic” when the primary process is an infection.

Here in we are interested in bacterial meningitis with or without locoregional infection complicated by CTP. Our objectives were to determine the risk factors predisposing to the occurrence of CTP in case of bacterial meningitis.

**Patients and methods** A retrospective case/control study performed in 2 units (intensive care and infectious diseases) over 44 months. Were recorded all patients who suffered from bacterial meningitis. Exclusion criteria were tuberculosis meningitis and meningitis on immunocompromised. We performed a multivariate logistic regression analysis to determine firstly the association between risk factors and occurrence of CTP and secondly between CTP and mortality. Analyse risk factors were: demographic factors, locoregional infection, craniofacial trauma, pregnancy, co morbidities (Thrombophilia, vascularitis, and cancer), clinical features (coma, seizures, cephalalgia), scannographic data, cytobacteriological and biochemical data of cerebro-spinal fluid (CSF) analysis, delayed diagnosis and/or management.

**Results** 100 patients were hospitalized with meningitis between Jan 2013 and Aug 2016. CTP occurred in 13 cases (13%). Age, sex-ratio, SAPS II and SOFA scores did not differ between patients with meningitis complicated by CTP versus those with meningitis not complicated by CTP: (39 vs 45 years, p = 0.22), (2.25 vs 1.71, p = 0.76), (28 vs 23, p = 0.25) and (4.3 vs 2.7, p = 0.08) respectively. Coma, cerebral oedema, cerebral commitment and positive cerebral spinal fluid (CSF) culture were significantly associated with the occurrence of TPC. SOFA >3 and contraception were near to significance (joined Table 20). It was not found a significant association of CTP to mortality (46% in the case group and 30% in control group, p = 0.08). Other factors that increased mortality were coma, seizures, shock, oedema, cellularity in CSF >1200 units/mm^3^. Otherwise, the ventilation length was prolonged with CTP group (8.7 vs 3.4 days, p = 0.034) and neurological sequels namely the epilepsy was more frequent with the group CTP: (23 vs 5%, p = 0.028).

**Table 20:** Factors associated with CTP complicating meningitis by stepwise logistic regression

variables	OR	(95% CI)	P value
Coma	3.73	(1.33–10.48)	0.019
Cerebral oedema	1.82	(1.07–3.39)	0.003
CEREBRAL commitment	1.45	(1.14–1.97)	0.018
Positive CSF culture	1.27	(0.99–1.64)	0.017
SOFA > 3	1.12	(0.95–1.33)	0.11
Oral contraception	1.32	(0.75–2.34)	0.17

**Conclusion** The occurrence of CTP on bacterial meningitis was significantly associated with CT scan lesions which seems to be an association be in both directions. Also, the positive culture predisposed more to the CTP. Mortality was higher with the presence of CTP but without real significance. The CTP was a factor that extends the ventilation time and exposed to the post infectious epilepsy.

**Competing interests** None.

**Reference**

1. Med Clin N Am. 2012;96:1107–26.

#### S38 Rapid diagnosis of bacterial meningitis using a point-of-care glucometer

##### Geoffroy Rousseau^1^, Romain Asmolov^2^, Leslie Grammatico-Guillon^3^, Adrien Auvet^2^, Said Laribi^1^, Denis Garot^2^, Youenn Jouan^2^, Pierre François Dequin^2^, Antoine Guillon^2^

###### ^1^Département de médecine d’urgence, Hospital Trousseau, Chambray-lès-Tours, France; ^2^Réanimation polyvalente, CHRU Hôpitaux de Tours, Tours, France; ^3^Service d’information médicale, epidémiologie et economie de la santé, CHRU Hôpitaux de Tours, Tours, France

####### **Correspondence:** Geoffroy Rousseau - grousseau.tours@gmail.com

*Annals of Intensive Care* 2017, **7**(**Suppl 1**):S38

**Introduction** Acute bacterial meningitis requires rapid triage and therapeutic decision-making. The aim of this study was to assess the overall ability of a point-of-care glucometer to determine bacterial infection in cerebrospinal fluid (CSF).

**Materials and methods** We performed a prospective, observational study. We included patients for whom an analysis of CSF was indicated by the physician in charge with blood sampling performed for glucose concentration measurement within 1 h. We simultaneously measured the glucose concentrations in CSF and blood using a central laboratory and point-of-care glucometer. The diagnosis of bacterial meningitis was determined by two physicians after reviewing the complete medical chart. We compared CSF and blood glucose concentrations and CSF/blood glucose ratios obtained at the bed-side with a glucometer versus those obtained by the central laboratory. We determined the performance characteristics of the CSF/blood glucose ratio provided by a glucometer to detect bacterial infection in the CSF immediately after CSF sampling.

**Results** We screened 201 CSF collection procedures during the study period and included 172 samples for analysis. Acute bacterial meningitis was diagnosed in 17/172 (9.9%) of CSF samples. The median turnaround time for glucose concentration analysis with a point-of-care glucometer was shorter than for the central laboratory: 5 [IQR 2–10] min versus 112 [IQR 86–154] min (p < 0.0001), respectively. The optimal cut off for the CSF/blood glucose ratio from central laboratory measurements to identify bacterial meningitis was 0.44 with a sensitivity of 94.1% [95% CI 71.3–99.9%], a specificity of 92.3% [95% CI 86.9–95.9%], and a positive likelihood ratio of 12.

The optimal cut off of CSF/blood glucose ratio calculated from a bed-side glucometer was 0.46 with a sensibility of 94.1% [95% CI 71.3–99.9%], a specificity of 91% [95% CI 85.3–95%] and a positive likelihood ratio of 10.

**Conclusion** We demonstrated that the CSF/blood glucose ratio measured by a glucometer can serve as a clinical decision support tool for the early detection of CSF with a high probability of bacterial infection. This costless point-of-care method has the potential to expedite medical decision-making for the triage of adult patients with suspected meningitis in the emergency department immediately after lumbar puncture.

**Competing interests** None.

#### S39 Guillain–Barré syndrome outbreak associated with Zika virus in Martinique

##### Jean-Louis Fergé^1^, Gwénolé Abgrall^1^, Ronan Hinault^1^, Shazima Vally^1^, Benoit Roze^2^, Agathe Chaplain^1^, Cyrille Chabartier^1^, Anne-Charlotte Savidan^1^, Sabia Marie^1^, Andre Cabie^2^, Dabor Resiere^1^, Ruddy Valentino^1^, Hossein Mehdaoui^1^

###### ^1^Intensive care unit, C.H.U La Meynard, Fort de France, Martinique; ^2^Department of infectious & tropical diseases, C.H.U La Meynard, Fort de France, Martinique

####### **Correspondence:** Jean-Louis Fergé - jean.ferge@gmail.com

*Annals of Intensive Care* 2017, **7**(**Suppl 1**):S39

**Introduction** An emergence of Zika virus (ZIKV) infections occured in Martinique, an 400,000 people island of the French West Indies, with more than 35,000 estimated cases between December 2015 and September 2016. The disease is usually benign and self-limiting if symptomatic (fever, headache, retro-orbital pain, non-purulent conjunctivitis, maculopapular rash, arthralgia, and myalgia). ZIKV is strongly suspected to be a cause of Guillain–Barré syndrome (GBS) based on case–control study describing French Polynesia outbreak in 2014 (1). Virological investigations were essentially both microsphere immunofluorescent and seroneutralisation assays for Zika virus and Dengue virus because of cross reactivity. Since then, numerous publications help to understand time of detection of ZIKV in biological human samples as blood or urine by Reverse-Transcriptase Polymerase-Chain-Reaction (RT-PCR) to diagnose ZIKV infection unambiguously. We aimed to asses the role of ZIKV in GBS outbreak in Martinique.

**Patients and methods** In this prospective cohort study, cases were patients with GBS at the French university hospital of Martinique during the outbreak period. Demographic, clinical, biological and electrophysiologic data, times of endotracheal intubation, and mechanical ventilation (MV) weaning were collected for all patients. At least 4 early predictors for MV were reached before endotracheal intubation (2). Weaning was usually decided when maximal minute ventilation was twice the minute ventilation after a 3–6 h trial of T-tube ventilation. Virological investigations included RT-PCR in blood and urine, and serological tests for ZIKV. Certainty ZIKV infection diagnosis was definite by RNA Zika detection by RT-PCR in blood or urine and by a negative screening for the others common etiologies of GBS (Cytomegalovirus, Epstein-Barr Virus, Human Immunodeficiency Virus and Human T-cell Lymphotropic Virus 1).

**Results** 30 patients were diagnosed for SGB during the study period. 21 had a certainty diagnosis for ZIKV infection. 13 patients were male. Median age was 65.4 years (range 56.7–71.2). 14 patients had clinical manifestations for ZIKV in a median preceding days of 5 (range 2–7). Immune therapy using immunoglobulins was realized in most of cases except for two patients who received plasma exchange. The median time before plateau phase was 8 days (range 6–12). 13 patients were hospitalized in ICU, 10 requiring MV for a median duration of 11.5 days (range 6–27). 19 patients underwent an electromyography in a median delay of 14 days (range 10–22). Acute inflammatory demyelinating polyradiculoneuropathy was diagnosed in 18 cases. 3 of them had axonal involvement.

**Discussion** Half of the patients had a need for MV. Temporality of events, certainty of ZIKV infection diagnosis by RT-PCR and exclusion of alternative explanations provide evidence for ZIKV causability in SGB. Annual incidence of SGB had already double comparing 10 previous years. SGB incidence backward have to be observe after the ZIKV outbreak.

**Conclusion** This study provides evidence for Zika virus infection causing Guillain–Barré syndrome. Consistency (same association found in different studies and populations) is strengthened by our study. At risk countries need to prepare for adequate intensive care beds capacity to manage patients with Guillain–Barré syndrome.

**Competing interests** None.

**References**

1. Cao-Lormeau VM. Guillain–Barré syndrome outbreak associated with Zika virus infection in French Polynesia: a case–control study. Lancet. 2016;387:1531–9.
2. Sharshar T. Early predictors of mechanical ventilation in Guillain–Barré syndrome. Crit Care Med. 2003;31:278–83.

#### S40 Prognostic value of standardized EEG features in post-anoxic coma after resuscitated cardiac arrest

##### Lucas Benarous^1^, Marième Soda-Diop^2^, Fouad Bouzana^1^, Gilles Perrin^1^, Jeremy Bourenne^1^, Béatrice Eon^1^, Dominique Lambert^1^, Agnes Trebuchon^2^, Marc Gainnier^1^

###### ^1^Service de réanimation des urgences et médicale, Hôpital Timone Adulte, Marseille, France; ^2^Service de neurophysiologie clinique, Hôpital Timone Adulte, Marseille, France

####### **Correspondence:** Lucas Benarous - lucasbenarous@gmail.com

*Annals of Intensive Care* 2017, **7**(**Suppl 1**):S40

**Introduction** Cardiac arrest remains a frequent cause of admission in intensive care unit. A majority of patients will die during their hospital stay mainly from consequences of hypoxic-ischemic brain injury after a decision of withdrawal of life sustaining therapy support by a prediction of poor outcome. The reliability of prognostication is crucial, but is still a difficult and uncertain exercise. EEG is the most widely used prognostic tool to support a clinical examination and is accessible in most hospitals. It is recommended for both prognostication and ruling out subclinical seizures. There is no high-level evidence for predicting poor prognosis using EEG because of the wide variety of classification systems used and the interrater variability. Our objective is to assess the prognostic value of simple EEG features based on the recent American Clinical Neurophysiology Society (ACNS) standardized classification and to study the interrater variability.

**Patients and methods** We conducted a retrospective monocentric observational study in a 12 bed medical intensive care unit of the university Hospital la Timone, Marseille, France. All patients aged of more than 18 year-old admitted for a resuscitated cardiac arrest between November 2012 and July 2014 who underwent therapeutic hypothermia and a full multimodal prognostic evaluation including a EEG were included in the study. Outcome was classified according to the Cerebral Performance Category Score measured at day 28. Unfavorable outcome was defined as death (CPC 5), persistent vegetative state (CPC 4), or severe neurological disability (CPC 3). Favorable outcome was defined as moderate neurological disability (CPC 2), or no disability (CPC 1). EEG was performed in all patients still comatose after rewarming between 48 and 72 h after admission and after discontinuation of sedation. EEG interpretation was made by 2 independent senior neurophysiologists, blind to the outcome. EEG features are based on the latest ACNS classification. For each EEG feature, sensitivity, specificity, positive predictive value (PPV), negative predictive value (NPV) for predicting an unfavorable outcome were calculated.

**Results** During the study period, 122 cardiac arrest were admitted of which 48 patients went through a full neurologic evaluation and were finally included in the study. According to neurological outcome, 19% had a favorable evolution, and 81% had an unfavorable outcome. The presence of burst suppression, and epileptiform activity was constantly associated with an unfavorable prognostic with a 100% specificity and 0% false positive. A non-reactive EEG is strongly associated with an unfavorable evolution with a 89% specificity and 3% false positive. Other features including periodic or rhythmic patterns and low voltage were inconstantly associated with unfavorable outcome. Kappa score for all EEG feature was slight or fair and always under 0.4.

**Discussion** This study allowed us to identify a homogenous cohort of comatose patient after cardiac arrest who underwent therapeutic hypothermia. We identified simple EEG features based on the new classification of the ACNS constantly associated with unfavorable outcome. These features must be known by intensivists to better integrate EEG in the multimodal evaluation of neurological prognostic. There is important interrater variability that must lead to caution and to always use multimodal approach to prognostic an unfavorable outcome.

**Conclusion** Bedside EEG is an excellent tool for predicting outcome of post-anoxic coma through simple EEG features. Burst suppression, epileptiform activity and non-reactive EEG are strongly associated to neurological outcome after cardiac arrest. However, the interrater variability emphasize the need of being well trained for the standardized methods of evaluating EEG parameters.

**Competing interests** None.

**References**

1. Westhall E. Neurology. 2016;86(16):1482–90.
2. Ben-Hamouda N. Chest. 2014;146(5):1375–86.

#### S41 The air-leak test revisited in childhood: a prospective observational study of feasibility in a PICU

##### Géraldine Poncelet^1^, Fleur Le Bourgeois^2^, Levy Michael^2^, Guillot Camille^2^, Jérôme Naudin^3^, Anna Deho^4^, Stéphane Dauger^3^

###### ^1^Pédiatrie, Hôpital Robert-Debré (AP-HP), Paris, France; ^2^Réanimation et surveillance continue pédiatriques, Hôpital Robert Debré, Paris, France; ^3^Réanimation et surveillance continue pédiatriques, CHU Robert Debré, Paris, France; ^4^Réanimation et surveillance continue pédiatriques, CHU Robert Debré, Paris, France, France

####### **Correspondence:** Géraldine Poncelet - gege.ponce@orange.fr

*Annals of Intensive Care* 2017, **7**(**Suppl 1**):S41

**Introduction** Emergent reintubation is a well-known risk of laryngo-tracheal trauma and of ventilatory acquired pneumonia. To precisely define its risk before extubation for each patient is a part of quality of care in intensive care units. The air-leak test (ALT) around the endotracheal tube (ETT) has been validated in adults to predict post-extubation adverse events such as stridor, upper airway obstruction or reintubation. Although the only six studies performed in childhood have shown highly variable results, this test is often used in pediatric intensive care units (PICU) where cuffed ETTs are now strongly recommended even in young infants. We report here the feasibility of different kinds of ALT in critically ill pediatric patients.

**Patients and methods** We designed a prospective, monocentric, observational study in our 12 beds-PICU of a University Hospital. From May 2016 to September 2016, we included all patients aged from 2 days to 18 years and intubated with cuffed ETT. We excluded patients intubated with uncuffed ETT, who were transferred still intubated to another PICU or who died during their stay in PICU. ALT was performed in all patients as soon as they were considered weanable from the ventilator by the intensivist in charge of the PICU. Thirty minutes before extubation, the intensivist successively tested two methods, before and after cuff deflation, with patients ventilated under CPAP (+5 cms H_2_O) with the FiO_2_ needed to obtain a saturation above 94%: (i) a qualitative test based on detection of an audible air-leak around the ETT with and without the use of a stethoscope placed on the larynx, (ii) a quantitative test known as the percent of cuff leak either calculated with expiratory tidal volume (VtE) or inspiratory tidal volume (VtI) (PCL: VtE inflated cuff - VtE deflated cuff/VtE inflated cuff × 100 or VtI inflated cuff - VtI deflated cuff/VtI inflated × 100). ALT was considered positive when no air-leak was audible and/or the PCL is less than 10%. Data are reported as medians [first-third quartiles].

**Results** During 4 months, 65 patients were mechanically ventilated in the PICU. 21 patients were excluded (11 deaths, 3 transferred still intubated and 7 intubations with uncuffed ETT). Of 44 patients with inclusion criteria, 43 (98%) were included (male/female: 30/13; age: 42 [6; 146] months; weight: 16.6 [5.6; 37.1] kg; PIM2 score: 3 [1.4; 5.6]; PELOD score at Day#1: 10 [1; 11]; medical/surgical: 28/15; duration of mechanical ventilation: 3 [2; 5] days; length of stay in PICU: 4.5 [3; 7] days). Numbers of cuffed ETT of each diameter were as follow: 1 × 2.5 cm, 8 × 3.0 cm, 7 × 3.5 cm, 5 × 4.0 cm, 7 × 4.5 cm, 3 × 5.0 cm, 3 × 5.5 cm, 3 × 6.0 cm, 4 × 6.5 cm, and 2 × 7.0 cm.

Every 10 senior pediatric intensivists working day or night in the PICU reported that performing all forms of ALT before extubation was easy, fast and safe. Clinical detection of an audible air-leak with or without a stethoscope was in agreement in 84% of cases. During the study period, no patients have been reintubated because of untractable inspiratory distress resisting to anti-inflammatory treatment (aerosol of epinephrine and systemic steroids) and non-invasive ventilation. 29 (67%) ALT were negative (presence of air-leak around ETT) and 14 (33%) were positive (absence of air-leak), with 67% concordance between qualitative and quantitative test. This concordance may depend on age (56% if <1 year, 76% if 1–7 years, and 78% if >7 years). With a cut-off of 10%, the result of the PCL calculation was identical using VtE or VtI.

**Discussion** Quite all patients have been included during the study period. ALT is fast and easy to safely perform at the bedside in PICU. It doesn’t take too much time for the intensivist. Audible ALT could be done without the use of a stethoscope which is difficult to place in front of the larynx on the anterior part of the neck in small infants. Comparing VtE, a usual parameter systematically measured by all PICU ventilators, before and after cuff ETT deflation, was the simplest ALT to use in this population. An average of six consecutive measurements, as performed in adults, could maybe enhance the test’s specificity. None of these 43 consecutive children representative of PICU activity has been reintubated. The coming prospective muticentric study which aims to validate ALT in childhood must precisely define this criteria of evaluation.

**Conclusion** The different methods of ALT are feasible in real clinical conditions in PICU. Because of the increasing use of cuffed ETTs in a wide variation of patients with different body weight, the best ALT to use at the bedside must be definitively validated in this population.

**Competing interests** None.

#### S42 Prognosis for newborns with prolonged mechanical ventilation: preliminary results of a retrospective single center cohort study

##### Michaël Sauthier^1^, Krystale Bergeron-Gallant^1^, Guillaume Emeriaud^1^, Philippe Jouvet^1^

###### ^1^Paediatric Intensive Care Unit, CHU Sainte-Justine, Chemin de la Côte-Sainte-Catherine, Montreal, QC, Canada, Montréal, Canada

####### **Correspondence:** Michaël Sauthier - michael.sauthier@umontreal.ca

*Annals of Intensive Care* 2017, **7**(**Suppl 1**):S42

**Introduction** Prolonged mechanical ventilation (PMV) and chronic mechanical ventilation (CMV) in neonates is associated with a high morbidity and mortality. The objective of the study is to identify, among the patients with PMV, those that evolved to CMV, as well as the adverse respiratory, neurological and feeding sequelae.

**Patients and methods** We conducted a retrospective study of the last 10 years at the CHU Sainte-Justine (Montreal, Canada). Chart review included patients with PMV (≥21 days) using the paediatric definition adapted from the 2005 NAMDRC consensus conference (1). Demographic and clinical data, including follow-up at 6 and 18 months corrected age, was collected for each included patient. The evolution of PMV neonates with CMV (≥125 days) and without (21–125 days) was compared.

**Results** We identified 174 neonates that met criteria for PMV. Patients born between 2004 and 2011 (n = 110, 63% of the cohort) were analyzed. Around half of the patients (5–10 patients a year) are transferred from the neonatal unit to the paediatric intensive care unit. In our center, they represent around 1% of total admissions, but their length of stay is among the longest. Among these 110 newborns, 79% were preterm (n = 87) with 45% (n = 50) born before 29 weeks gestation. Of all patients with a malformation (52%, n = 57), 21 had a thoracoabdominal anomaly and 24 had congenital heart disease. Thirty-six patients had CMV with mean ventilation time of 239 days (range 125–753 days). Survival at 18 months corrected age was 66% (49/74) in the PMV group and 72% (26/36) in the CMV group. At 18 months corrected age, 30% of patients were dependent on artificial enteral feeding (nasogastric tube or gastrostomy), with 18% in the PMV group and 54% in the CMV group. Nine percent of patients had oxygen supplementation (2 patients in the PMV group and 4 in the CMV group), and 7% were mechanically ventilated. Ten percent of patients had a tracheostomy (5 patients in the PMV group and 2 in the CMV group).

**Discussion** Neonates with CMV have more sequelae. Their rapid identification (at 21 days of ventilation) is essential to implement multidisciplinary development care in order to minimize neurodevelopment impairment.

**Conclusion** Most newborns in our PMV cohort have a congenital malformation. Survival at 18 months corrected age appears equivalent in both PMV and CMV group. Artificial enteral feeding is more frequent in the CMV group and most patients have no respiratory support at 18 months corrected age.

**Competing interests** None.

**Reference**

1. Sauthier M, Rose L, P Jouvet. Paediatric prolonged mechanical ventilation: Considerations for definitional criteria. Resp Care; 2016 (In Press).

#### S43 Relative value of pressures and volumes in assessing fluid responsiveness in children: the role of systolic cardiac function

##### Nicolas Tiebergien^1^, Matthias Jacquet-Lagrèze^1^, Jean-Luc Fellahi^1^

###### ^1^Anesthésie réanimation, Hôpital Louis Pradel, Bron, France

####### **Correspondence:** Matthias Jacquet-Lagrèze - matthias.jl@gmail.com

*Annals of Intensive Care* 2017, **7**(**Suppl 1**):S43

**Introduction** The value of pressures and volumes in assessing the fluid responsiveness depend on the systolic cardiac function in adult (1). We have studied the relative value of static filling volume and pressure to predict the fluid responsiveness, according to systolic cardiac function in children during acute circulatory failure.

**Patients and methods** Patients under 8 years old with an acute circulatory failure of two intensive care units during a 1 year period of inclusion were analyzed. An exhaustive cardiac echography was performed initially (indexed End-diastolic volume (EDVi) and E/e’ from transmitral and tissue Doppler were recorded), and the stroke volume index (SVI) was measured before and after a fluid challenge (a 10 ml/kg of crystalloid over 10 min). SVI was computed as the left ventricular outflow tract velocity time integer multiplied by the left ventricular outflow tract surface. Patients were responders to fluid loading if their SVI increased of at least 15%. Patients were considered to have a low systolic function if their Left Ventricular Ejection Fraction (LVEF) was under 50% or normal if it was over 50%. R software with pROC package was used to performed descriptive and analytic statistic. Receiver operative characteristic curves were built. Bootstrap technic was used to compute the confidence intervals. p < 0.05 was considered significant. Local institutional review boards approved this study (CPP Lyon Sud Est II). Written informed consent was obtained from parents.

**Results** Twenty-five children with acute circulatory failure were included. Fluid responsiveness occurred in 6 of the 13 fluid loading events with low LVEF, and in 6 of the 12 fluid loading events with normal LVEF. Pressure approach: For low and normal LVEF, the AUC-ROC for fluid responsiveness was respectively 0.83 (CI 0.56–1)/0.73 (0.39–1) for a E/e’.The best thresholds of E/e’ in low LVEF was 7.9 with a sensitivity of 87 (CI 62–100) % and a specificity of 67 (CI 33–100) %. For low and normal LVEF AUC ROC was respectively 0.6 (CI 0.21–0.99)/0.52 (CI 0.14–0.9) for the PVC.

Volume approach: For low and normal LVEF, the AUC-ROC for fluid responsiveness was respectively 0.69 (CI 37–1) and 0.74 (0.4–1). The best thresholds in normal LVEF was an EDVi below 29 ml/m2 wit a Specificity of 83 (CI 50–100) and a sensitivity of 71 (CI 42–100) %.

**Discussion** Our study shows a variation of the diagnostic value of E/e’ and EDVi according to the left ventricular systolic function. Therefore, the systolic function should be taken into account to analysed the E/e’ and EDVi value. Few preload dependency markers are validated in Children and none for children in spontaneous ventilation (2). Our study suffers from a lack of power that calls into question the validity of our results. Another limitation is that both approaches with volume and pressure are not very discriminant as it is known for static value in adults. Our study illustrates that, on a pressure–volume curve, when the cardiac inotropism is reduced, the filling of the Left ventricle is moved to the up and right of the curvilinear diastolic function curve. Therefore, pressure variations are larger than volume variations. These values should be monitored on a larger scale to define their exact diagnostic value.

**Conclusion** Static PVC value is a low preload-dependency surrogate. When LVEF is low a pressure evaluation based approach seems more accurate. When LVEF is normal a volume evaluation based approach seems informative as predicted by the slope of the end diastolic pressure volume curve. Those both static approaches remain of poor diagnosis accuracy.

**Competing interests** None.

**References**

1. Trof RJ. Cardiac filling volumes versus pressures for predicting fluid responsiveness after cardiovascular surgery: the role of systolic cardiac function. Crit. Care. 2011.
2. Desgranges F-P. Respiratory variation in aortic blood flow peak velocity to predict fluid responsiveness in mechanically ventilated children: a systematic review and meta-analysis. Pediatr Anesth. 2015.

#### S44 Prone position reduces work of breathing in children with severe bronchiolitis

##### Florent Baudin^1^, Guillaume Emeriaud^2^, Sandrine Essouri^2^, Jennifer Beck^3^, Etienne Javouhey^4^, Claude Guérin^5^

###### ^1^Réanimation pédiatrique, Hôpital Femme Mère Enfant, Bron - Lyon, France; ^2^Soins intensifs pédiatriques, CHU Sainte-Justine, Montréal, Canada; ^3^Keenan research center, St Michael’s Hospital, Toronto, Canada; ^4^Réanimation pédiatrique hfme, Hospices civils de Lyon, Lyon, France; ^5^Réanimation médicale, Hôpital de la Croix-Rousse, Lyon, France

####### **Correspondence:** Florent Baudin - florent.baudin@chu-lyon.fr

*Annals of Intensive Care* 2017, **7**(**Suppl 1**):S44

**Introduction** Acute viral bronchiolitis is a primary cause of respiratory distress in paediatric intensive care unit (ICU). Prone position (PP) is commonly used in neonates to improve respiratory mechanics and has been found beneficial to adult patients with acute respiratory distress syndrome. We aimed to evaluate the effect of PP on work of breathing as compared to supine position (SP) in children with severe bronchiolitis requiring non-invasive ventilation.

**Fig. 15:**
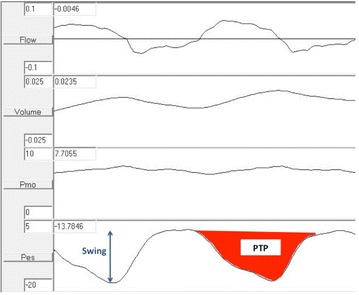
Example of recording and main analysis (*PTP* pressure time product)

**Patients and methods** The protocol was approved by our IRB (2015- A01200–49). Fourteen infants (9 boys) with median age 33 days [first-third quartiles 25–58] with severe bronchiolitis requiring CPAP were included after written informed consent. Children were investigated in PP and SP each applied for 1 h in a random order with a washout period of 10 min between them. Level of CPAP was set at 7 cmH_2_O in both conditions. Oesophageal pressure probe was inserted orally (CTO-2 pressure transducer, Gaeltec, Scotland) to measure oesophageal pressure. Flow and airway pressure (Pmo in Fig. 15) were simultanuously recorded using a Neurovent data acquisition system (Neurovent Inc, Toronto, Canada). One hundred breaths were analyzed in each condition, in which work of breathing was estimated from oesophageal pressure–time product (PTPes) and oesophageal swings (Fig. 15). Data were expressed as median (first-third quartiles) and compared by using the Wilcoxon two-sample paired sign test. A p-value below 0.05 was considered significant.

**Results** Respiratory rate was not different in PP and SP position (66 [46–78] vs 59 [52–77] breaths/minute, p = 0.40). Between PP and SP, the oesophageal PTP per breath was 3.5 [2.9–4.2] vs 4.6 [3.4–5.1] cmH_2_O s, respectively, p = 0.048 and the swings of oesophageal pressure amounted to 9.3 [8.3–12.8] vs 14.9 [11.0–16.2] cmH_2_O, respectively, p = 0.035.

**Discussion** Work of breathing estimated by using the oesophageal PTP and oesophageal swing was lower in PP than in SP in children who required non-invasive ventilation for severe bronchiolitis. This physiologic study suggests that PP may improve respiratory condition in children with severe bronchiolitis.

**Conclusion** In children with severe bronchiolitis, the prone position decreased significantly the work of breathing compared to the supine position during non-invasive ventilation.

**Competing interests** F. Baudin: has received speaking fees from Maquet critical care. J. Beck: through Neurovent Research, serves as a consultant to Maquet Critical Care. She has made inventions related to neural control of mechanical ventilation that are patented. The license for these patents belongs to Maquet Critical Care. Future commercial uses of this technology may provide financial benefit to Dr. Beck through royalties.

**References**

1. Numa AH, et al. Effect of prone and supine positions on functional residual capacity, oxygenation, and respiratory mechanics in ventilated infants and children. Am J Respir Crit Care Med. 1997;156:1185–89.
2. Gouna, et al. Positioning effects on lung function and breathing pattern in premature newborns. J Pediatr. 2013;162:1133–7.

#### S45 Characteristics and initial prognostic factors of abusive head trauma in pediatric intensive care unit

##### Marie Lampin^1^, Ouardia Mamouri,^2^, Patrick Devos^3^, Yasemin Karaca-Altintas^1^, Matthieu Vinchon,^4^, Stéphane Leteurtre^1^

###### ^1^Service de réanimation pédiatrique, CHRU de Lille, Lille, France; ^2^Service de pédiatrie, CH d’Armentières, Armentières, France; ^3^Service de biostatistiques, CHRU Lille, Lille, France; ^4^Service de neurochirurgie pédiatrique, CHRU de Lille, Lille, France

####### **Correspondence:** Marie Lampin - marie-emilie.lampin@chru-lille.fr

*Annals of Intensive Care* 2017, **7**(**Suppl 1**):S45

**Introduction** Abusive head trauma assigned a frequency to high morbidity and mortality and it is not uncommon in pediatric intensive care unit. The aim of this study was to describe the frequency and the characteristics of abusive head trauma from all head trauma in children under 2 years hospitalized in a pediatric intensive care unit and define initial prognostic factors of mortality.

**Patients and methods** We performed a retrospective observational cohort study, including all patients under 2 years, admitted in pediatric intensive care unit of Lille, France for head trauma from January 2004 to December 2014. Outcome for initial prognostic factors was mortality.

**Results** One hundred forty patients were included, 64% were abusive head trauma and 36% were accidental head trauma. The main differences between the 2 groups were respectively age (3 vs 12 months), retinal hemorrhages (81 vs 8%), subdural hematoma (94 vs 40%), location of fractures (long bones: 61 vs 0%; ribs: 46 vs 3%) and location of ecchymosis (face: 84 vs 8%; skull: 32 vs 96%) (all p < 10–4). Seizures were more common in abusive head trauma (74%) versus accidental head trauma (24%) (p < 10–4). Patients with abusive head trauma were mostly boys (sex ratio: 1.4). Mortality rate was higher in abusive head trauma (26%) versus accidental head trauma (8%). Prognostic factors of mortality were younger age (p = 0.03), a threshold probability of death PIM 2 of 8% (Se 92%, Sp 77%) and 12% for the PRISM III score (Se 89% Sp 77%), Glasgow score ≤8 (OR [CI95%]: 7 [53; 424]), lactate level greater than 2 mmol/l and initial hypoxic-ischemic injuries in cerebral tomodensitometry (CT) (p < 10–4). Only subdural-peritoneal shunt placement was a protective factor (OR 0.007 [95% CI 0.2, 0.6]; p = 0.003).

**Conclusion** In our study, abusive head trauma is a frequent cause of head trauma in children under 2 years (68%) and is more lethal than other head injuries. Young age, PIM2 or PRISM III score, Glasgow score, lactatemia and hypoxic-ischemic brain injuries were associated to mortality.

**Competing interests** None.

#### S46 High quality electronic database in paediatric intensive care unit: where are we after 1 year!

##### David Brossier^1^, Redha Eltaani^1^, Michaël Sauthier^2^, Guillaume Emeriaud^1^, Philippe Jouvet^1^

###### ^1^Soins intensifs pédiatriques, CHU Sainte-Justine, Montréal, Canada; ^2^Paediatric Intensive Care Unit, CHU Sainte-Justine, Chemin de la Côte-Sainte-Catherine, Montreal, QC, Canada, Montréal, Canada

####### **Correspondence:** David Brossier david_brossier@yahoo.fr

*Annals of Intensive Care* 2017, **7**(**Suppl 1**):S46

**Introduction** Since May 2015, we have been able to collect several physiological and therapeutic data in an electronic database (eDTB) implemented in our paediatric intensive care unit (PICU) of CHU Sainte Justine using all medical devices at bedside and informatics network of the hospital. The purpose of this abstract is to present the data after 1 year of exploitation.

**Materials and methods** The eDTB was implemented after the CHU Sainte Justine ethical committee approval. Data are collected from admission to discharge, every 5 s from monitors and every 30 s from mechanical ventilator and infusion pumps. All these data are linked to medical information retrieved from electronic medical chart.

**Results** Between May 21th 2015 and July 31st 2016, 993 patients were hospitalised in our PICU, among them 43 weren’t included in the eDTB for technical and age (over 18 years old) reasons. Eventually, after 20 exclusions, 930 patients remained in the eDTB. Mortality was 4.3% (n = 40). The eDTB contained 1063 PICU stays (1048 complete and 15 partial).The median age was 2 years old [0–17], median weight was 12.9 kg [2.3–104.0]. The median ICU stay was 53 h [3–11,720] or 2 days [0–488]. The first motive for admission was respiratory causes (308, 28.9%) Other principal motives were post-surgery (186, 17.5%) post cardiac surgery procedures (160, 15.1%), neurologic causes (116, 10.9%). Other motives were under 10% (hemodynamic causes, traumatic injuries, infectious diseases, intoxications, metabolic causes, cancer, organ transplant, cardiac arrest). The eDTB contains data from ventilated patients (invasively and non-invasively) and details concerning ionotropic and sedative treatment during PICU courses.

**Discussion** As far as we know, this eDTB is currently the only one as exhaustive available in PICU worldwide. After almost 3 years of multidisciplinary collaboration, we are able to collect many useful physiological, therapeutic and medical data in an ongoing eDTB. Although many concerns remain concerning data validation, organisation and exploitation, this eDTB already contribute to the development of clinical decision support systems and virtual patient validation and we create international collaborations to further develop these tools. Three research protocols using the database are ongoing including: validation of a neuromonitoring clinical decision support system, validation of a cardio-respiratory simulator, developement and validation of the automatic diagnosis of pediatric acute respiratory distress syndrome and development of SpO2 forecast using artificial neuronal network.

**Conclusion** Thanks to informatics and electronic devices improvement, data gathering in intensive care units has empowered. We hope that our work in PICU will encourage other teams on the way of data gathering, in order to build an international PICU eDTB in a close future.

**Competing interests** None.

#### S47 Pre-hospital control of secondary systemic cerebral insults in children referred to a pediatric trauma center with traumatic brain injury

##### Sonia Teyssedre^1^, Meyet Sabine^2^, Jean-Christophe Bouchut^3^, Olivier Peguet^3^, Etienne Javouhey^4^

###### ^1^69, Hôpital Femme Mère Enfant, Bron, France; ^2^01, Hospital Center Fleyriat, Bourg-en-Bresse, France; ^3^69, Edouard Herriot Hospital, Lyon, France; ^4^Réanimation pédiatrique hfme, Hospices civils de Lyon, Lyon, France

####### **Correspondence:** Sonia Teyssedre - sonia.teyssedre@chu-lyon.fr

*Annals of Intensive Care* 2017, **7**(**Suppl 1**):S47

**Introduction** Severe trauma is rare in the pediatric setting (15% of all trauma in France). However, its morbidity and mortality remain high, in relation to brain injury. Pediatric traumatic brain injury (TBI) pre-hospital care is challenging for non-pediatric retrieval teams. Though, we disseminated pediatric TBI pre-hospital care regional guidelines and thereafter intended to assess severe pediatric trauma pre-hospital care and secondary cerebral insults control.

**Materials and methods** We conducted a retrospective study in a single pediatric trauma center. Children admitted in emergency room with severe trauma and moderate to severe TBI (Glasgow coma scale ≤12) from June 2014 to March 2016 were included. Pre-hospital and hospital data regarding primary care, equipment, medications and secondary cerebral insults control (i.e. blood pressure, oxygenation, CO_2_ level, temperature, glycemia) were collected from medical files. Two pediatric transport team experts assessed the quality of pre-hospital care, based on two major endpoints.

**Results** Twenty-nine files were analyzed. Median ISS was 34 [9–75]. All the children had been referred directly from the trauma scene to the pediatric trauma center. They were all intubated in the pre-hospital setting, 4 (13.7%) presented with SpO2 < 90% before or at emergency room admission, and 17 (58.6%) presented with a pCO2 >45 mmHg at admission. At least one peripheral catheter was inserted in all the children. Mean total fluid bolus was 26.9 mL/kg (± 14). Nor-epinephrine was administered in 16 (55%) children. Mean blood pressure was below age threshold in 18 (62%) children during transport or at admission. An intracranial hypertension treatment (apart from sedation) was delivered in 17 (55%) children before admission. Body temperature was monitored in 10 patients and 17 were hypothermic at emergency room admission. Experts concluded on sub-optimal care in 17 children: major endpoint was “respiratory care”, “hemodynamic care” and “neurologic care” in 13, 12 and 9 patients respectively.

**Discussion** On this small series, we showed pre-hospital sub-optimal care regarding secondary cerebral insults control, especially regarding CO_2_ level, blood pressure and body temperature. Our results will help to design new care improvement strategies (e.g. sedation, fluid bolus and ventilation optimization, early use of vasoactive drugs, systematic body temperature monitoring…).

**Conclusion** Data on pre-hospital secondary cerebral insults care are rare in the pediatric setting. Based on our results, we aim to improve quality of care of children presenting with traumatic brain injury, and to reduce its morbidity and mortality.

**Competing interests** None.

#### S48 Failure extubation in intensive care unit: risk factors, incidence and evaluation of a mechanical ventilator weaning protocol

##### Lucie Petitdemange^1^, Anne Sophie Guilbert^1^

###### ^1^67, University De Strasbourg - Campus Médecine, Strasbourg, France

####### **Correspondence:** Lucie Petitdemange - lucie.petitdemange@outlook.fr

*Annals of Intensive Care* 2017, **7**(**Suppl 1**):S48

**Introduction** Unsuccessful extubation from mechanical ventilation increases mortality and morbidity. To reduce the extubation failures in our Intensive Care Unit we used a mechanical ventilator weaning protocol, based on published data. During the first part of the study, risk factors and incidence of extubation failure were first described. Afterwards in the second part, our mechanical ventilator weaning protocol was tested to determined its efficiency regarding the extubation failure.

**Patients and methods** A monocentric and observational study, was first conducted. We included 245 children aged from birth to 18 old, during a period of 15 months and collected for each patient their medical history, intubation and extubation parameters, and existing events of extubation failure or extubation complication. The second part of the study was prospective, we include 70 patients extubated by applying our mechanical ventilator weaning protocol.

**Results** Average duration of mechanical ventilation was 51.6 h in the first part of the study. Using a univariate analysis, duration of mechanical ventilation was a risk factor of extubation failure with an average duration of 131.6 h in the group with an extubation failure versus 40.8 h in the group without failure (p = 0.000005). Chronic respiratory affection (OR 2.46 [1.04; 5.57] p = 0.049), and history of previous intubation (OR 2.46 [1.26; 7.41] p = 0.005) were found to be failure extubation failure risk factors. SaO_2_ was statistically lower for a higher FiO_2_ in the group with failure extubation. Use of sedation did not change the risk of extubation failure except for midazolam. Administration of benzodiazepine at the time of extubation significantly increased extubation failure (OR 3.06 [1.07; 8.33]; p = 0.02).

In children intubated for respiratory or neurologic distress we observed more extubation failures compared to children intubated for hemodynamic distress or surgical reasons (p = 0.02).

Using a multivariate analysis, each mechanical ventilation additional hour increased of 1.004 the risk of failure extubation [1.001; 1.0081] (p = 0.02). Extubation complications increase by 10.86 the failure extubation risk [3.947; 29.907] p = 0.000004.

The mechanical ventilator weaning protocol in ICU strongly reduced the risk of extubation failure (OR 4.55 [1.10; 40.35] p = 0.026). The number needed to treat with the protocol to avoid one extubation failure is 12 patients. Our protocol did not change the average duration of mechanical ventilation. (51.6 vs 48 h p = 0.07) neither the incidence of extubation complications (17.1 vs 10% p = 0.19).

**Discussion** Our study confirms published data about extubation failure risk factor like duration of intubation, chronic respiratory affection, history of previous intubation, and the administration of benzodiazepine. It is the first pediatric study that shows a reduction of extubation failure by using a specific mechanical ventilator weaning protocol. The mean bias of our its retrospective and prospective character.

**Conclusion** Our study shows the interest of a mechanical ventilator weaning protocol to reduce the incidence of extubation failure. We currently continue the apply our protocol to include more patients in order to confirm our results.

**Competing interests** None.

#### S49 Problem diagnosis and management of stroke in children in the PICU of the hospital establishment of canastel ORAN

##### Amel Zerhouni^1^, Djamila-Djahida Batouche^2^, Nadia Benatta^3^, Nabil Tabet Aoul^4^, Zakaria Addou^5^, Nabil Aouffen^6^

###### ^1^Reanimation, EHS CANASTEL, oran, Algeria; ^2^Réanimation pédiatrique, Centre Hospitalier et Universitaire d’Oran, Oran, Algeria; ^3^cardiologie, Centre Hospitalier et Universitaire d’Oran, Oran, Algeria; ^4^Réanimation pédiatrique canastel, Faculté de médecine d’Oran, Oran, Algeria; ^5^Réanimation pédiatrique de Canastel d’oran, Departement de medecine d’Oran Algerie, Oran, Algeria; ^6^Anesthésie réanimation pédiatrique, Etablissement hospitalier spécialisé en pédiatrie Canastel, Oran, Algeria

####### **Correspondence:** Djamila-Djahida Batouche - khedidjabatouche@yahoo.fr

*Annals of Intensive Care* 2017, **7**(**Suppl 1**):S49

**Introduction** Stroke of the child is formidable though it is ten times rarer than in adults, but this scarcity can have adverse consequences on the speed and quality of the management and the consequences on later psychomotor development. Our goal is to describe the clinical and therapeutic aspects of these pediatric stroke while bringing our experience.

**Patients and methods** Retrospective study of cases of children hospitalized in general intensive care unit to the pediatric hospital Canastel Oran for stroke during the period from January 2014 to January 2016. The clinical, etiological, para clinical, and scalable were studied and transcribed on a standard electronic form.All patients had a brain CT. Magnetic resonance imaging(MRI) was possible in 02 patients for lack of availability of the technical facilities during the study.

**Results** Ten cases were selected. The mean age was 64 months (1 month to 15 years), 66% are male, 2 patients had a history of CHD like tetralogy of Fallot and complicated bronchiolitis myocarditis, one patient had a history of petechial purpura, 1 other was a factor 7 deficiency, headache history was noted in 02 patients, and 04 patients with no particular antecedent was found.

All patients arrived comatose 11/15 score on the scale of glasgow, isochores reactive pupils with a motor deficit of hémicorps, 03 patients have degraded their neurological score with onset of clinical signs of hypertension intra cranial namely anisocoria and hypertension requiring osmotherapy, sedation and mechanical ventilation with an average duration of 2–7 day. O1 child arrived brain dead, 03 patients had generalized tonic–clonic seizures which yielded after taking a benzodiazepine (Diazepam) and phenobarbital (like gardenal). Cerebral CT was performed in all cases and could we revealed the nature of the stroke hemorrhagic in 07 cases and ischemic stroke in 03 cases. Two patients have benefited from an MRI that found a thrombosis of the artery internal carotid right Sylvian. Besides symptomatic treatment, treatment was initiated based on the type of stroke, 03 patients received low molecular weight heparin (LMWH) at 0.1 ml/kg in addition to symptomatic treatment, 02 patients received vitamin K. Four patients died in an array of autonomic disorders and evolved favorably and six patients were transferred to a pediatric unit.

The average length of stay in ICU was 5.5 days (2–10 days).

**Discussion** The mortality rate is important since no specialized center for children, and difficulty especially in the diagnostic imaging field while Suspected stroke should be confirmed by imaging and the diagnostic delay. Which is due to a poor assessment of the initial situation in half of the cases by the parents, the other half by the Swiss magazine consulté.une doctor showed that in a study in 42% of children with stroke, this diagnosis was not primarily discussed and that in 11% of cases the cause of the stroke was poorly evaluated [1]. Heart disease certainly represent the second most important risk factor. A collaboration of a team must be multidisciplinary, death has affected mostly older children whose age is between 11 and 15 years, who have a hemorrhagic stroke against by infants who have an ischemic stroke have evolved and oriented they exceed the acute phase to pediatric services for further investigation and monitoring.

**Conclusion** The child may also be having a stroke, which usually reaches the elderly. This justifies a good knowledge of this disease, and multiply the initial management efforts to reduce mortality and improve prognosis.

**Competing interests** None.

**Reference**

1. Steinlin M, Wehrli E. Berne. L’accident vasculaire ischémique en pédiatrie. Quand y penser – quoi faire. Pediatrica. 20(2); 2009

#### S50 Bacterial nosocomial infections incidence in medicosurgical pediatric intensive care unit

##### Anwar Armel^1^, Benqqa Anas^1^, Samira Kalouch^2^, Khalid Yaqini^2^, Aziz Chlilek^2^

###### ^1^Anesthésie réanimation, CHU Ibn Rochd, Casablanca, Morocco; ^2^Service de réanimation pédiatrique, Chu Ibn Rochd, Casablanca, Morocco

####### **Correspondence:** Anwar Armel - armelanwar@gmail.com

*Annals of Intensive Care* 2017, **7**(**Suppl 1**):S50

**Introduction** Nosocomial infections are a main problem for public health for their cost as well as for the morbidity and mortality they generate. They are particularly common in intensive care units due to patient’s lower defenses and of invasive procedures proliferation.

Work’s purpose:

- Determine the epidemiology of bacterial noso-Comiales infections (IBN) in the medico-surgical pediatric intensive care department of Children’s University Hospital of Casablanca.
- To identify factors associated with these infections.

**Patients and methods** We led a retrospective study of hospitalized patients, spending more than 48 h in medical-surgical pediatric intensive care department, at the University Hospital Ibn Rochd of Casablanca, over a period of 12 months from 1 January 2015 to 31 December 2015.

**Results** During the studied period, 420 patients were admitted at intensive care with a stay of more than 48 h.

Thirty episodes of INB were recorded. The incidence rate was 7.1% and the incidence density was 20.6% per 1000 hospitalization’s days.

The admission average age was 4.6 ± 22-month starting from 1 month to 12 years with a male predominance (60%).

Most of admissions (80%) was related to medical background, 50.3% received from other hospital department.

Furthermore, 40% of the patients received prior antibiotics, usually prescribed before ICU admission.

Invasive procedures (intubation, central catheterization) were used in 93.3% of patients, VVP only in 6.66%, tracheotomy in 33.3 and 6.66% had received surgery.

Gram-negative bacilli (BGN) were isolated for a lot of patients, dominated by *Acinetobacter baumannii*. These bacteria were isolated throughout the study year. Risk factors Analysis underlined that the presence of invasive procedures enhances IN risk, that is central venous catheter and the need for mechanical ventilation.

**Conclusion** Nosocomial bacterial infections are dominated by pneumonia and central catheter infections, and are mainly due to BGN. The factors associated with these infections were identified.

**Competing interests** None.

#### S51 Guillain–Barré syndrome mortality factors in pediatric intensive care

##### Anwar Armel^1^, Rchi Abdou^2^, Samira Kalouch^3^, Khalid Yaqini^3^, Aziz Chlilek^3^

###### ^1^Département d’anesthésie réanimation, CHU Ibn Rochd Casa, Casablanca, Morocco; ^2^Anesthésie réanimation, CHU Ibn Rochd, Casablanca, Morocco; ^3^Service de réanimation pédiatrique, Chu Ibn Rochd, Casablanca, Morocco

####### **Correspondence:** armelanwar@gmail.com (Anwar Armel)

*Annals of Intensive Care* 2017, **7**(**Suppl 1**):S51

**Introduction** The Guillain–Barré syndrome (GBS) is the most common cause of acute flaccid paralysis in children since the acute anterior poliomyelitis eradication.

Few studies have been held on the topic and knowledge of GBS in children, although it is recognized that the etiologic mechanisms, and clinicobiological background, are the same as in adults, prognosis remains different.

Our work’s aim is to study this disease’s mortality factors of children hospitalized in pediatric intensive care.

**Patients and methods** It is a retrospective, descriptive, mono centric study to review 35 patients with GBS between January 2009 and December 2015 and hospitalized at pediatric intensive care department of AbderrahimHarouchi hospital of Casablanca.

The used software is SPSS 16.0 to compare the bivariate variables, we used the khi2 test, and to compare quantitative variables, the ANOVA to 1 factor test was used. The level of significance was fixed at 5% with 95% confidence interval.

**Results** The disease was predominant in male with a sex ratio of 1.7 men/women. After a prodromal event, usually infectious (95.7%) and a free interval of 15 days on average to start motor disorders.

These are of two types: either a hypo or areflectic flaccid paralysis of the lower limbs (25.7%) of ascending evolution in 91.4% of the cases. Either flaccid tetraplegia or hypo areflectic, (74.3%).

Ventilation was required in 65.7% of the cases, and specific treatments based on immunoglobulins were administered in 88.6% of the cases.

Death’s rate is still high (22.9%) and mainly due to hospitalization complications.

In our study respiratory disease was noted in 65.7% of the cases, also other signs of serious illness such as swallowing disorders (71.4%) and autonomic disorders (14.3%) also noted what led to management in intensive care for all our patients.

These patients study allowed to identify some mortality prognosis factors of the disease in intensive care units (such as male gender, Ig administration duration, the occurrence of autonomic disorders like blood pressure instability), the most discriminating remains the occurrence of nosocomial infections.

**Conclusion** It must be underlined, that in view of our strict inclusion criteria, focusing only on patients admitted at intensive care and of the relatively small sample size (35 cases), our results must be qualified and must be enhanced by additional and more varied studies to better understand this disease in children.

**Competing interests** None.

#### S52 Management of infantile refractory intracranial hypertension: decompressive craniectomy versus medical therapy alone

##### Perrine Gravellier^1^, Julie Chantreuil^1^, Nadine Travers^2^, Antoine Listrat^2^, Claire Le Reun^1^, Geraldine Favrais^1^

###### ^1^Réanimation pédiatrique, Hôpital Clocheville, Centre Hospitalier Universitaire Tours, Tours, France; ^2^Chirurgie pédiatrique de la tête et du cou, Hôpital Clocheville, Centre Hospitalier Universitaire Tours, Tours, France

####### **Correspondence:** Julie Chantreuil - j.chantreuil@chu-tours.fr

*Annals of Intensive Care* 2017, **7**(**Suppl 1**):S52

**Introduction** Early surgical treatment is recommended for refractory intracranial hypertension (HTIC) in children to improve vital and functional prognoses, whether traumatic or vascular cause. The main objective of this study was to compare the mortality and morbidity of children with severe intracranial hypertension after severe head trauma (TC) or due to vascular cause after decompressive craniectomy (DC) or medical therapy alone. The secondary objective was to identify the initial severity factors associated with higher mortality.

**Patients and methods** A retrospective study was performed with data collected from patients aged under 18 years-old admitted to our pediatric intensive care unit for severe intracranial hypertension of traumatic or vascular cause, between January 2000 and January 2016. They were divided into 2 groups: patients who received medical therapy alone and those treated with decompressive craniectomy after optimal medical management.

**Results** A total of 83 children were included. Among them, 17 were treated with DC (6 HTIC of vascular cause and 11 HTIC of traumatic cause), and 66 were supported by medical means only (6 HTIC of vascular cause and 60 HTIC of traumatic cause).

In the population “traumatic intracranial hypertension”, we note that children in the “DC” subgroup are more often in mydriasis upon arrival (p = 0.017) than in the subgroup treated medically. In this same population, children in the “DC” subgroup received higher doses of MIDAZOLAM (p = 0.015), of MANNITOL (p = 0.0041) and hypertonic saline (p = 0.0006) than in the other subgroup. In the population “vascular intracranial hypertension” the two subgroups were comparable.

In the case of traumatic intracranial hypertension, mortality rate in the “DC” subgroup was 54.5% against 13.34% for children treated medically (p = 0.0015); “DC” children had more metabolic complications such as hypernatremia than “not DC” children, p = 0.043. Mortality rate in the «vascular intracranial hypertension» group was 0% for children treated with decompressive craniectomy, and 83.3% for children treated medically alone (p = 0.015). Patients treated surgically in the «vascular intracranial hypertension» group had longer overall stays (p = 0.009) and longer ICU stays (p = 0.016). POPC Score (Pediatric Overall Performance Category) upon discharge for children with intracranial hypertension of traumatic cause treated with decompressive craniectomy was 4.91 ± 1.37 against 3.75 ± 1.31 among children treated medically, p = 0.08. In “DC” children with intracranial hypertension of vascular cause, POPC upon hospital discharge was 3.66 ± 0.51 against 5.66 ± 0.81 among non-operated children, p = 0.007. The schooling rate was higher among children treated medically for intracranial hypertension of traumatic cause, p = 0.012. The severity factors related with higher mortality identified in the population “traumatic intracranial hypertension” were mydriasis upon admission, a PIM 2 score higher and a lower temperature (<35.5°); the latter being the only factor identified for HTIC of vascular cause. In the case of traumatic intracranial hypertension, ICP monitoring in survivors was 40.35% against 28.6% in children died, with no significant difference. In the population “vascular intracranial hypertension”, all the patients who died had not been monitoring PIC.

**Discussion** The severity factors related with higher mortality identified in the population “traumatic intracranial hypertension” were mydriasis upon admission, a PIM 2 score higher and a lower temperature (<35.5°); the latter being the only factor identified for HTIC of vascular cause. Other studies have related other severity factors as initial Glasgow scale, tardive decompressive craniectomy.

**Conclusion** Decompressive craniectomy doesn’t seem to improve the mortality rate or the outcome in patients with hypertension of traumatic cause in our study but the DC traumatic subgroup was more serious than the subgroup treated medically. In children with refractory intracranial hypertension of vascular cause DC significantly improves survival and outcome. Further studies are needed to clarify the role of decompressive craniectomy and its timing in the therapeutic management of refractory intracranial hypertension.

**Competing interests** None.

**Reference**

1. Wang R, Li M, Gao W–W, Guo Y, Chen J, Tian H–L. Outcomes of early decompressive craniectomy versus conventional medical management after severe traumatic brain injury: a systematic review and meta-analysis. Medicine (Baltimore). 2015.

#### S53 Myocardial dysfunction evaluation in pediatric brain death donor

##### Zoe Coppere^1^, Stéphane Blanot^2^, Juliette Montmayeur^2^, Régis Bronchard^3^, Stephane Rolando^4^, Gilles Orliaguet^2^

###### ^1^Fondation Ophtalmologique Adolphe de Rothschild, Paris, France; ^2^Réanimation neurochirurgicale pédiatrique, Hospital Necker, Paris, France; ^3^Agence de Biomédecine, Saint-Denis, France; ^4^Service de régulation et d’appui, Agence biomedecine, Malakoff, France

####### **Correspondence:** Zoe Coppere - zozocop@hotmail.com

*Annals of Intensive Care* 2017, **7**(**Suppl 1**):S53

**Introduction** Shortage of heart grafts is a major problem, leading to a significant mortality rate in the national waiting list, essentially for young children with low weight. The potential paediatric brain-dead donors often have myocardial dysfunction (MD), which seems to be reversible.

The aim of this study is to assess prevalence, causes and consequences of MD when the potential paediatric donors are taken over, up to multi-organ retrieval, and the evolution after cardiac transplantation.

**Materials and methods** This observational, monocentric, retrospective study included all brain-dead children aged 0–18 years old, who had their myocardial function assessed through a cardiac ultrasound performed by a cardiologist and identified from 2004 to 2016. All adult patients and those who didn’t undergo a cardiac ultrasound were excluded.

MD was defined as an LVEF ≤50% with or without abnormal segmented cinetic parameters.

The main evaluation criteria was the prevalence of MD in potential identified donors. The secondary evaluation criteria were the causes and consequences of MD on heart retrieval and the origin of this MD.

**Results** Out of 40 included patients, 11 had MD. Prevalence of MD was of 30%.

There was no significant difference between 2 groups regarding aetiology of brain death nor administration of catecholamines. Having a cardiopulmonary arrest during intensive care unit stay was associated with a significant risk of presenting a MD (p = 0.034). Having a MD had no consequences on organ retrieval in general (p = 0.25), but was significantly associated with a decrease in heart retrieval opportunities (p = 0.036). The cause of heart grafts refusal was a poor ventricular function in 50% of cases (3 cases out of 6). The cause for non-retrieval was parental refusal in one-third of cases. Evolution of the cardiac grafts was favorable in 16 cases on 18, one transplanted patient died (from a non-cardiac cause) and 1 patient was lost to follow up.

**Conclusion** MD in paediatric brain-dead patients has direct consequences on heart retrieval and transplantation, and otherwise, organ shortage is a major ongoing problem. A better transplant management regarding hemodynamics (with the use of a protocol) could increase the number of heart transplants, especially in small children, and reduce mortality rate in national waiting list.

**Competing interests** None.

#### S54 Practice survey on prone positioning in French-speaking pediatric intensive care units (medical part)

##### Pierre-Louis Leger^1^, Jérôme Rambaud^1^, Emilie Thueux^1^, Alexandra De Larrard^1^, Véronique Berthelot^1^, Julien Denot^1^, Marie Reymond^1^, Alain Amblard^1^

###### ^1^réanimation néonatale et pédiatrique, Hopital pour enfants Trousseau, Paris, France

####### **Correspondence:** Pierre-Louis Leger - leger.pierrelouis@gmail.com

*Annals of Intensive Care* 2017, **7**(**Suppl 1**):S54

**Introduction** The prone positioning (PP) is a strategy widely used in the treatment of severe forms of acute respiratory distress syndrome (ARDS) in adults. Its early use significantly reduces mortality (1). However, the studies do not strongly demonstrate its prognostic impact in pediatric ARDS. The aim of this study was to describe the prone positioning practices in the French-speaking pediatric intensive care units (PICU).

**Patients and methods** This survey was conducted by email questionnaire to pediatric intensivists belonging to the French Society of Intensive Care Medicine and the French-speaking Group of Pediatric Intensive Care and Emergency Medicine. It was conducted From February to May 2016. The survey was addressed to doctors, nurses, physiotherapists practicing in PICU. It included 29 questions about indications, contraindications, techniques and medical devices used, and complications.

**Fig. 16:**
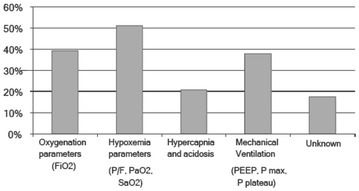
Criteria used to stop Prone positionin in the French-speaking Pediatric Intensive Care Units

**Results** One hundred and three persons answered (69 doctors and 33 nurses) which work in 28 french hospitals and 1 canadian hospital. Sixty-eight percent of interviewed persons have more than 5 years experience and 57% of them treat each year more than 10 children ARDS. Only 10% of the PICU have a PP medical protocol. Fifty percent of interviewed persons frequently use PP for the medical care of ARDS and 30% systematically use it. Thirty-six percent begin PP at the early phase of ARDS during conventional ventilation, while 42% before the introduction of unconventional ventilatory strategies (OHF); only 12% use it after the respiratory failure unless unconventional ventilatory strategies. Seventy-three percent report that PP is used with prolonged periods (>12 h/day), 22% with short periods (<12 h/day) and 14% with very long periods (>20 h/day). Regarding the weaning criteria, most of interviewed persons seem to use multiple and combinated criteria: 51% use hypoxemia severity parameters (PaO_2_/FiO_2_, PaO_2_, SaO_2_), 39% use the oxygen level (FiO_2_) and 38% use the mechanical ventilation parameters (PEEP, P max, P plate). Finally, despite a low level of scientific evidence in children, 87% of the persons gave a strong recommendation for PP as standard care in severe pediatric ARDS. See Fig. 16.

**Discussion** The survey confirmed the widely use of PP in pediatric ARDS. However, no specific protocol is avalaible in most of the PICU. The timing of the PP beginning can be different according to children, early and prior to use of the conventional ventilation strategy in most cases. The duration of PP seems more consensual. Most of the centers use extended periods longer than 12 h/day. These results are close to Guérin et al. advocating a duration >16 h/day. Finally, the weaning is a great issue and depends on multiple criteria. In Guerin et al. (2) PP was interrupted if one of the following criteria were present: PaO_2_/FiO_2_ ≥ 150 mmHg, with PEEP of ≤10 cm of water and a FiO_2_ of ≤0.6; decreased PaO_2_/FiO_2_ than 20%, compared to compared to the supine position, or the occurrence of complications. No study has validated PP weaning criteria during pediatric ARDS.

**Conclusion** The prone positioning is a strategy commonly used in pediatric intensive care units for the severe pediatric ARDS. The criterias of implementation and timing are variable, as well as the weaning criterias. More pediatric multicenter randomized studies will be necessary to confirm the benefits of PP in pediatric ARDS and to define clear weaning criteria.

**Competing interests** None.

**References**

1. Bloomfield R, Noble DW, Sudlow A. Prone position for acute respiratory failure in adults. Cochrane Database Syst Rev. 11:CD008095.
2. Guerin C, Reignier J, Richard JC, Beuret P, Gacouin A, Boulain T, et al. Prone positioning in severe acute respiratory distress syndrome. N Engl J Med. 368(23):2159–68.

#### S56 Severe opportunistic infections in critically ill allogeneic bone marrow transplantation recipients

##### Sarah Morin-Zorman^1^, Etienne Lengliné^2^, Claire Pichereau^3^, Eric Mariotte^4^, Canet Emmanuel^5^, Virginie Lemiale^6^, Frédéric Pène^7^, Elie Azoulay^6^

###### ^1^SAINT LOUIS, Paris, France; ^2^Hématologie, Hôpital Saint Louis, Paris, France; ^3^Réanimation médicale, Hôpital Saint-Antoine, Paris, France; ^4^Service de réanimation médicale et infectieuse, Hôpital Bichat-Claude Bernard-APHP, Paris, France; ^5^Réanimation médicale, SAINT LOUIS, Paris, France; ^6^Réanimation médicale, Hôpital Saint-Louis, Paris, France; ^7^Réanimation Médicale, Hôpital Cochin, Paris, France

####### **Correspondence:** Sarah Morin-Zorman - smorinzorman@gmail.com

*Annals of Intensive Care* 2017, **7**(**Suppl 1**):S56

**Introduction** Allogeneic Hematopoietic Stem Cell Transplantation (HSCT) recipients have profound defects in every immunity compartments that can lead to severe opportunistic infections (OI). 20% of HSCT patients require admission to the ICU because of diverse infectious or non-infectious complications with dismal outcomes. OI specific course in this population has not been described previously and the management of these infections may be a concern. The aim of this study was to investigate risk factors, management and outcomes of IO in HSCT recipients admitted to the ICU.

**Patients and methods** This was a retrospective (2007–2016) single center study of patients admitted to ICU after an allogeneic HSCT. Patients provided written informed consent according to Helsinki declaration. Data regarding the transplant, infections and life sustaining therapy use were analyzed. OI were considered if present at the time or during ICU admission.

**Results** Hundred and ninety-four patients (Pt) were included. Median age was 43 [28; 58] years, 63.2% were males. Reason for transplantation was acute leukemia in 84 (42%) Pt and the hematological condition was still in complete remission at ICU admission in 83% of patients. 78 (41%) and 53 (28%) had received a myeloablative conditioning regimen and anti-thymoglobulin serum respectively. 40% had acute graft versus host disease over grade 2 at ICU admission. OI was documented in 38 patients (20%). An invasive fungal infection (IFI) was found in 19 Pt owing to 3 mucormucosis, 1 trichosporon septicemia and 15 invasive aspergillosis (5 possible, 9 probable and 1 proven according to EORTC criteria). Serum Galactomannane antigen was positive in 10 (67%). Median time from transplantation and ICU admission to IFI diagnosis was respectively 86 [18; 529] and −3 [−8; 0] days. Lung was involved in 90% and patients with aspergillosis were admitted to the ICU for acute respiratory failure in 71% (vs. 33% for others p = 0.2). They did not required invasive ventilation more frequently (36 vs. 31% p = 0.8). 32 and 10% required vasopressors and renal replacement therapy with no difference as compared to others. Median ICU length was 3 [2; 6] days. Demographic, stem cell source, and donor type were not associated with IFI occurrence in this population. However 15/19 had received a total body irradiation (78 vs. 47% p = 0.01). IFI occurrence was not associated with ICU or Day 90 mortality (33 vs. 26% p = 0.7 and 53 vs. 41% p = 0.3 respectively). A viral infection was found in 17 Pt owing to 11 CMV, 1 adenovirus, 1 HSV and 1 VRS infections. Analyses were focused on CMV reactivation. Median time from transplantation and ICU admission to CMV reactivation was respectively 111 [50; 151] and −23 [−38; −10] days. Reactivation was mainly positive blood PCR but 1 pt had CMV colitis. A preemptive treatment was started on the same day in median and lasts 34 [21; 71] days. Patients with CMV reactivation had more frequently multiple organ failure (50 vs. 22% p = 0.06) and higher ICU admission SOFA score (7 [5; 12] vs. 6 [3–8] p = 0.05). They trend to have higher admission creatinine serum level (132 [72; 157] vs. 81 [60; 120] umol/L, p = 0.2) and more frequently required emergency renal replacement therapy (36 vs. 7% p = 0.01) mechanical ventilation (64 vs. 30% p = 0.04) and vasopressors (73 vs. 37% p = 0.02). Median ICU length was 4 [2; 11] days and comparable to others. Demographic, stem cell source, conditioning regimen and donor type were not associated with CMV occurrence. CMV reactivation was not significantly associated with ICU or Day 90 mortality (36 vs. 26% p = 0.5 and 60 vs. 41% p = 0.3 respectively).

**Conclusion** OI was found in 20% of allogeneic HSCT recipients admitted to the ICU. IFI were mainly responsible for respiratory distress and CMV associated to multiple organ failure. Non-invasive diagnostic tests were positives in a majority of these patients. In this cohort, IO treatment was started quickly after the diagnostic and we did not find an association with mortality. Intensivists should always consider OI in their diagnostic panel in this specific population.

**Competing interests** None.

#### S57 Opportunistic infections in patients with solid tumors: a systematic review

##### Julien Poujade^1^, Elie Azoulay^2^

###### ^1^Service de réanimation médicale, Hôpital Saint-Louis (AP-HP), Paris, France; ^2^Réanimation médicale, Hôpital Saint-Louis, Paris, France

####### **Correspondence:** Julien Poujade - julien.poujade236@gmail.com

*Annals of Intensive Care* 2017, **7**(**Suppl 1**):S57

**Introduction** Over the last two decades, targeted therapies in patients with solid tumors have both increased their length of survival and significantly altered their immune functions. However, data on opportunistic infections in this setting remain scarce. In this systematic review, we sought to identify published cases of opportunistic infections in patients with solid tumors, with a special interest on clinical findings, trends over time and outcomes.

**Materials and methods** We performed a search of Medical Subject Headings (MeSH) on PubMed using the words pneumonia pneumocystis (PCP), invasive aspergillosis (IA), histoplasma, mucor, geotrichum, cryptococcus, coccidioidomycosis combined with the MeSH term neoplasms (breast, lung, ovarian, urologic gastrointestinal, digestive system, abdominal, brain, carcinoid tumor, sarcoma, testicular, seminoma). We identify published cases of opportunistic infections in non HIV patients with solid tumors between 01/01/1966 and 05/01/2016 included.

**Results** Regarding *Pneumocystis jirovecii* pneumonia, 94 cases could be identified. There were 32 men and 62 women, aged of 57.5 (19–80) years. Underlying tumors were chiefly brain neoplasms (n = 31, 33%), lung neoplasms (n = 24, 26%) and breast neoplasms (n = 16, 17%). At the time of Pneumocystis pneumonia onset, 52 patients (55%) had a history of chemotherapy, 58 (62%) had received long term or high dose steroids, and 21 (22%) had an history of biotherapy targeting the malignancy. Of note, 18 patients (19%) had received only chemotherapy, 17 (18%) had received steroids alone, 4 (4%) everolimus therapy alone and 3 (3%) received none of these treatments. Regarding invasive aspergillosis 64 cases could be identified. Mean age was 66.7 (37–82) and 46 (72%) were men. Solid tumors associated with invasive aspergillosis were primarily lung neoplasms (n = 31, 48%) and brain neoplasms (n = 24, 38%). At aspergillosis onset, 38 (59%) patients had a history of chemotherapy, 29 (45%) were receiving long term or high dose steroids and 3 (5%) had received targeted therapy. Fourteen (22%) patients had received only chemotherapy, 2 (3%) only steroids, and 1 (1.5%) had received targeted therapy alone.

For both infection, there was a trend for a higher number of reported cases throughout the studied period.

**Conclusion** This systematic review provides objective data showing that an increased proportion of patients with solid tumors present with opportunistic infections. We are convinced that it is a clinically relevant but still neglected problem. Selected oncologic population may be becoming eligible for antimicrobial prophylaxis against Pneumocystis or Aspergillus.

**Competing interests** None.

#### S58 Invasive aspergillosis in non-immunocompromised patients hospitalized intensive care unit

##### Guillaume Trumpff^1^, Max Guillot^2^, Thierry Braun^2^, Ralf Janssen-Langenstein^2^, Marie-Line Harlay^2^, Jean-Etienne Herbrecht^2^, Quentin Maestraggi^3^, Baptiste Michard^4^, Maleka Schenck^2^, Francis Schneider^2^, Vincent Castelain^5^

###### ^1^Anesthésie-réanimation, C.H.R.U. Hôpitaux Universitaires Strasbourg, Strasbourg, France; ^2^Réanimation médicale, C.H.R.U. Hôpitaux Universitaires Strasbourg, Strasbourg, France; ^3^Service de réanimation médicale, Hôpital de Hautepierre du C.H.R.U, Avenue Molière, Strasbourg, France, Strasbourg, France; ^4^Service de réanimation médicale, Hôpital de Hautepierre, Hôpitaux Universitaires de Strasbourg, Strasbourg, France; ^5^Hautepierre réanimation médicale, C.H.R.U. Hôpitaux Universitaires Strasbourg, Strasbourg, France

####### **Correspondence:** Guillaume Trumpff - trumpffguillaume@gmail.com

*Annals of Intensive Care* 2017, **7**(**Suppl 1**):S58

**Introduction** Characteristics and outcomes of adult patients with invasive aspergillosis in intensive care unit have rarely been described.

**Materials and methods** We performed a retrospective study on consecutive adult patients with invasive aspergillosis who were admitted form January 2010 through January 2016 to the intensive care unit of Strasbourg in France. Patients were included only if they are non-immunocompromised according to the European Organisation for Research and Treatment of Cancer (EORTC). Invasive aspergillosis was defined as an association of microbiological evidence, a radiological imaging and a clinical context.

**Results** Eighteen patients (13 males) were identified during the study period. The median of IGS II was 60.5 (interquartile range (IRQ), 49.25–78.75). Ninety-four percent was under mechanical ventilation. Fourteen (78%) patients were suffering from liver failure. Among liver failure, twelve (86%) were beforehand suffering from cirrhosis. The median MELD score was 42 (interquartile range (IRQ), 30–53). Sixty-four percent of aspergillosis were due to *Aspergillosis fumigatus*. Hundred percent were pulmonary aspergillosis. Fifty-six percent of aspergillosis were associated with bacterial pneumonia. The mortality rate at the date of the latest news (an average of 2 years) was seventy-two percent.

**Discussion** Invasive aspergillosis is not exceptional in the non-immunocompromised patient especially in patient developing liver failure. An active research of colonization/infection with aspergillus in these patients remain to be discussed.

**Conclusion** Invasive aspergillosis in ICU has a poor prognosis. The liver failure seems to be the most important risk factor in non-immunocompromised patients according EORCT criteria.

**Competing interests** None.

#### S60 Invasive fungal infection after liver transplantation: diagnostic value of 1,3-beta-d-glucan (BG)

##### Noorah Zaid^1^, Nawel Ait-Ammar^2^, Christine Bonnal^2^, Jean-Claude Merle^1^, Francoise Botterel^2^, Eric Levesque^1^

###### ^1^Anesthesia and intensive care medicine, CHU Henri Mondor, Créteil, France; ^2^Unité de parasitologie-mycologie, département de virologie, bactériologie-hygiène, parasitologie, hopital henri mondor, Créteil, France

####### **Correspondence:** Eric Levesque - eric.levesque@aphp.fr

*Annals of Intensive Care* 2017, **7**(**Suppl 1**):S60

**Introduction** Liver transplant recipients have high rate of Invasive Fungal Disease (IFD) with high morbidity and mortality, in part due to its delayed diagnosis. The fungal cell wall component (1,3)-beta-d-glucan (BG) is a biomarker for fungal infection but its utility remains uncertain. This prospective study was designed to review our experience in IFD and to evaluate the impact of BG in the diagnosis of IFD.

**Patients and methods** From January 2013 to May 2016, 271 liver transplantation were performed in our institution. Serum samples were tested for BG (Fungitell; Cape Cod Inc., USA) least weekly between liver transplantation and their discharge from hospital. IFD was defined as proposed by the European Organization for research and treatment of Cancer/mycoses study group.

**Results** Nineteen patients (7%) were diagnosed with IFD including 13 cases of Candidiasis infection (CI) in eleven out of 271 patients, 8 invasive pulmonary aspergillosis (including one who had previously CI) and one case of septic arthritis of the hip caused by *Scedosporium* spp. IFD was associated with significantly high mortality (log-rank p = 0.003). The area under the ROC curves, for BG to predict IFD, was 0.78 (95% CI 0.73–0.83). Using a cutoff of 139 pg/ml, the most discriminative cut-off point from the ROC curve, The sensitivity, specificity, Positive Predictive Value (PPV) and Negative Predictive Value (NPV) values of BG for overall IFD was 68% (95% CI, 59–77), 79% (95% CI, 76–78), 24% (95% CI, 19–30) and 96% (95% CI, 95–97).

**Conclusion** Based on its high NPV, BG value appears to be a good biomarker to rule out the diagnosis of IFD when the value is below 139 pg/ml. A single point BG may guide the investigation and the decision to start antifungal therapy in patients at risk for IFD.

**Competing interests** None.

#### S61 Monitoring of changes in lung and chest wall mechanics in the supine, lateral and prone positions during the prone positioning maneuver in ARDS patients

##### Zakaria Riad^1^, Mehdi Mezidi^1^, Hodane Yonis^1^, Mylène Aublanc,^1^, Sophie Perinel-Ragey,^1^, Floriane Lissonde^1^, Aurore Louf-Durier,^1^, Romain Tapponnier^1^, Jean-Christophe Richard^1^, Bruno Louis,^2^, Claude Guérin^1^, PLUG working group

###### ^1^Réanimation médicale, Hôpital de la Croix-Rousse, Lyon, France; ^2^Inserm, u955, equipe 13, équipe biomécanique cellulaire et respiratoire, Université Paris-Est Créteil - Faculté de médecine, Créteil, France

####### **Correspondence:** Zakaria Riad - zakaria.riad@icloud.com

*Annals of Intensive Care* 2017, **7**(**Suppl 1**):S60

**Introduction** Chest wall elastance (Ecw) has been found to increase in prone (PP) as compared to supine position (SP) in ARDS patients [1]. This makes respiratory system elastance (Ers) not reflecting lung elastance (El). Little is known about the changes of Ecw, El and lung resistance (Rl) when moving the patient from the SP to the PP via the lateral position (LP). The goal of present study was to measure Ecw, El and Rl in ARDS patients in SP, LP and PP during the proning procedure.

**Patients and methods** It was a prospective, single-center, controlled study. ARDS patients intubated, sedated and paralyzed with PaO_2_/FiO_2_ ratio < 150 mmHg, PEEP ≥ 5 cmH_2_0 and an indication of PP were included. Mechanical ventilation was delivered in volume controlled mode with constant flow inflation and end-inspiratory pause 0.100 s included into the inspiratory time. Ventilator settings were unaltered during the procedure. An esophageal balloon catheter (Nutrivent device) was used for esophageal pressure (Pes) measurement. Pressure at the airway opening (Pao) and airflow were measured by Fleish 2 pneumotachograph proximal to endotracheal tube and upstream Heat and Moisture exchanger. Pao, Pes and airflow were continuously measured during 30 min in SP, then during 1 min in LP and 30 min in PP. The side for the lateralization was that selected by routine practice (in the opposite side from central venous line). Ers and resistance of the respiratory system (Rrs) were obtained by fitting flow and Pao signals breath by breath to the first order equation. Ecw and resistance of the chest wall (Rcw) were similarly obtained by fitting flow and Pes signals breath by breath to the first order equation pertaining to the chest wall. El and lung resistance (Rl) were obtained by subtracting Ers and Rrs from Ecw and Rcw, respectively. Our ethical committee approved the protocol. Data are shown as median (first and third quartiles). Comparisons between positions were made by using paired-t-test.

**Results** Twenty-nine patients, 19 males, of 68 (62–74) years, SAPS 2 45 (34–55) and SOFA score 7 (3–9) were included 1 (0–2) days after ARDS criteria were met. The ARDS severity was moderate in 25 cases (86%) and severe in 4 (14%). Tidal volume averaged 5.9 (5.6–6) ml/kg predicted body weight, PEEP 10 (10–10) cmH_2_O, FiO_2_ 60 (50–70) %, PaO_2_/FiO_2_ 122 (108–139) mmHg. The cause of ARDS was pulmonary in 21 cases (72%), extra pulmonary in 3 (10%) and undetermined in 6 (18%). Lateral positioning was on the right side in 14 (48.3%) and on the left side in 15 patients (51.7%). The results are shown in the Table 21.

**Table 21:** Respiratory mechanics during the proning procedure

	SP	LP	PP
Ecw (cmH_2_O/L)	9.6 (6.5–12.5)	10.5 (6.9–13.3)	11.3 (7.3–13.3)**
El (cmH_2_O/L)	27.7 (21.7–43.9)	30.8 (23.5–44.5)	31.3 (23.0–47.0)
Rl (cmH_2_O/L/s)	13.6 (11.2–16.0)	14.2 (12.2–17.5)**	14.7 (13.2–17.0)

** p < 0.01 versus supine

**Conclusion** During prone positioning in ARDS patients, as compared to SP we observed a higher RL in LP and an increased Ecw in PP.

**Competing interests** None.

**Reference**

1. Pelosi P, et al. Effects of the prone position on respiratory mechanics and gas exchange during acute lung injury. Am J Respir Crit Care Med. 1998;157(2):387–93.

#### S62 Effects of neuromuscular blockers on trans pulmonary pressures in moderate-to-severe acute respiratory distress syndrome

##### Christophe Guervilly^1^, Jean-Marie Forel^1^, Magali Bisbal^2^, Samuel Lehingue^1^, Malika Mechati^3^, Jeremy Bourenne^4^, Gilles Perrin^4^, Romain Rambaud^1^, Mélanie Adda^1^, Sami Hraiech^1^, Elisa Marchi^1^, Antoine Roch^1^, Marc Gainnier^4^, Laurent Papazian^1^

###### ^1^Réanimation des détresses respiratoires et infections sévères, Hôpital Nord APHM, Marseille, France; ^2^Reanimation, Institute Paoli-Calmettes, Marseille, France; ^3^Réanimation des détresses respiratoires et infections sévères, Hôpital Nord APHM, Toulon, France; ^4^Reanimation des urgences et medicale, CHU la Timone 2, Marseille, France

####### **Correspondence:** Christophe Guervilly - christophe.guervilly@ap-hm.fr

*Annals of Intensive Care* 2017, **7**(**Suppl 1**):S62

**Introduction** Neuromuscular Blocking Agents (NMBA) could exert beneficial effects in Acute Respiratory Distress Syndrome (ARDS) through properties on respiratory mechanics and particularly in modifying transpulmonary pressures (P*L*).

**Patients and methods** Prospective randomized control study in moderate to severe ARDS patients within the first 48 h of the onset of ARDS. All patients were monitored by an esophageal catheter and followed during 48 h. Moderate ARDS patients were randomized in two groups according to the systematic administration of a 48 h continuous infusion of cisatracurium besylate or not (control group). The severe ARDS patients group received a 48 h continuous infusion of cisatracurium besylate. The evolution during the 48 h of the study of the oxygenation and the respiratory mechanics including inspiratory and expiratory transpulmonary pressures and driving pressure were assessed and compared. Delta transpulmonary pressure (∆P*L*) was defined as inspiratory P*L* minus expiratory P*L*.

**Fig. 17:**
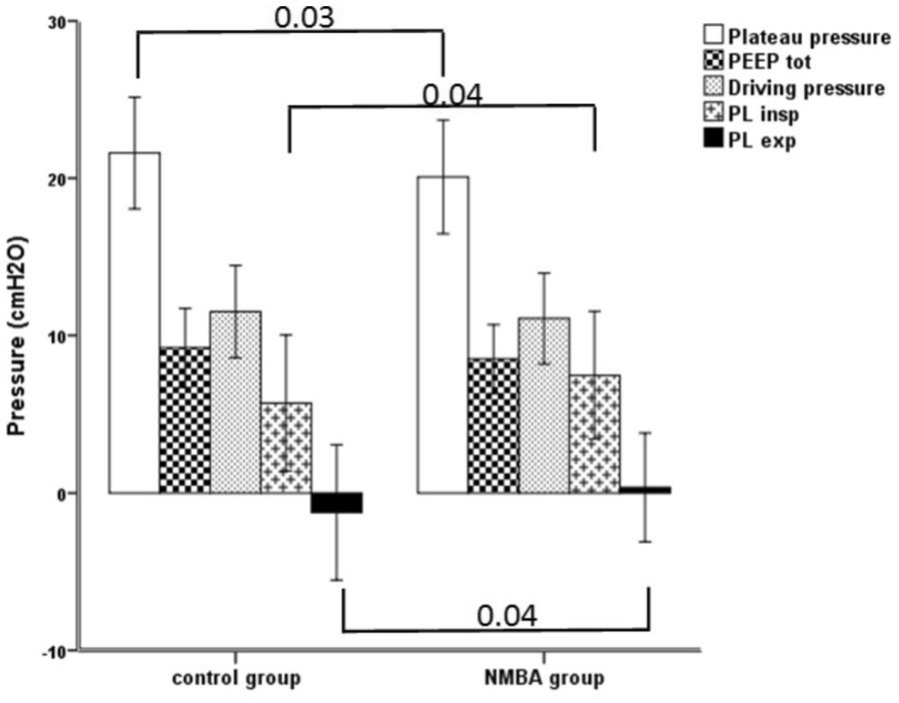
Mean pooled values of Pplat, PEEP tot, ∆P, P*L insp*, P*L exp* in moderate ARDS, excluding baseline values, shown as mean and standard deviation

**Results** Thirty patients were included, 24 in the moderate ARDS group and 6 in the severe ARDS group. NMBA infusion was associated with an improvement in oxygenation both the moderate and the severe ARDS patients group accompanied by a decrease in both the plateau pressure and the total positive end expiratory pressure. The mean inspiratory and expiratory P*L* were higher in the moderate ARDS patients group receiving NMBA as compared with the control group (Fig. 17). In contrast, there was no modification of both the driving pressure and the ∆P*L* related to NMBA administration.

**Conclusion** NMBA could exert beneficial effects in moderate ARDS patients through higher observed inspiratory and expiratory transpulmonary pressures.

**Competing interests** None.

#### S63 Prone position: impact on intracranial pressure and cerebral perfusion pressure in ARDS patients with brain injury

##### Jeremy Bourenne^1^, Vincent Guerin^1^, Gilles Perrin^2^, Lucas Benarous^3^, Fouad Bouzana^1^, Dominique Lambert^1^, Sami Hraiech^4^, Laurent Papazian^5^, Marc Gainnier^1^

###### ^1^Service de réanimation des urgences et médicale, Hôpital Timone Adulte, Marseille, France; ^2^Réanimation urgence et médicale pr auffray, Hôpital de la Timone, Marseille, France; ^3^13, Hôpital Timone adulte, marseille, France; ^4^Réanimation DRIS, Hôpital Nord APHM, Marseille, France; ^5^Service de réanimation-détresses respiratoires et infections sévères, Hôpital Nord, Marseille, France

####### **Correspondence:** Jeremy Bourenne - jeremy.bourenne@ap-hm.fr

*Annals of Intensive Care* 2017, **7**(**Suppl 1**):S63

**Introduction** Prone position (PP) is a major treatment in management of acute respiratory distress syndrome (ARDS). The use of PP in patients with severe ARDS associated with brain injury is at high risk of intracranial hypertension.

The aim of this study is to analyze the effect of PP on intracranial pressure (ICP) and cerebral perfusion pressure (CPP) in patients with ARDS and acute neurological condition requiring monitoring of ICP.

**Patients and methods** It is a retrospective descriptive study including sixteen patients with acute brain injury (subarachnoid hemorrhage, severe head trauma, and hemorrhagic stroke) and continuous monitoring of ICP who developed a severe ARDS during ICU stay from January 2012 to December 2015 and for which PP was performed. 29 PP sessions were analyzed. Hemodynamic and respiratory parameters, blood oxygenation, PIC and PPC were studied in supine, before PP and after PP. The study was approved by FICS ethic comity.

**Fig. 18:**
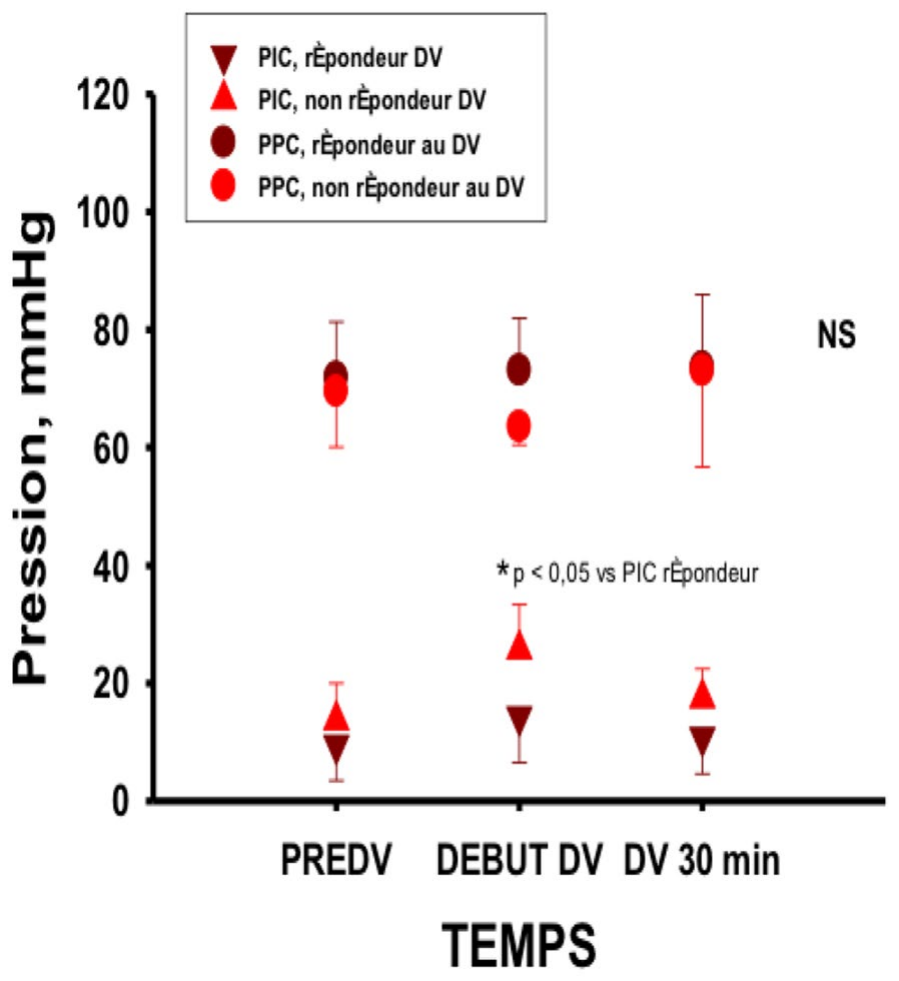
Evolution from ICP and CPP during the first PP session, according to the response

**Results** A significant increase in PaO_2_/FiO_2_ ratio was observed in PP, from 112 ± 34 to 200 ± 84 (p < 0.01). In PP, the ICP was increased 11 ± 1.1–17 ± 1.3 mmHg (p < 0.05) while the CPP was stable 74 ± 2 versus 76 ± 2 mmHg (NS). Median duration of PP session was 16 h (12–20). Increasing of ICP during PP required medical treatment in 8 sessions (27%). PP session was interrupted in 6 sessions (20%). In subgroup of patients who respond to PP in terms of oxygenation, the increase of ICP was lower than in non-responders (43 vs 118%) (p < 0.05). CPP was not modified whatever the nature of the response to PP (76 ± 15–77 ± 11 in non-responders and from 73 ± 10 to 76 ± 10 in responders (NS)) (Fig. 18).

**Discussion** Our study shows an improvement of oxygenation during PP in severe ARDS patient with acute brain injury. We observe a constant increment of PIC during PP sessions. The increment of ICP is less in responders to PP.

Significant increased ICP requiring an enhancement in the medical treatment was observed in 30% of the cases, and lead in most cases to a discontinuation of the session. Our data underlined the absolute necessity to monitor ICP during PP session in patients with acute brain injury and ARDS, even if ICP is controlled previously in supine.

Only 3 prospective (1, 2) and one retrospective studies evaluate the effects of PP on ICP in patients with acute brain injury and acute respiratory failure (ARF). They results are similar to ours. In all these studies, the severity of ARF was often not well specified. Roth and al. (2) had included only 10% of ARDS in a population of patient with ICP not controlled. In others studies, monitoring of ICP during PP was not systematic.

Despite the retrospective nature of the study and the small number of patients, it is the only work studying the effects of PP on intracranial pressure in patients with acute brain injury at risk for intracranial hypertension and severe ARDS according to the Berlin’s definition.

**Conclusion** Our work suggest that PP is a quite secure technique for use for the treatment of severe ARDS even patients at risk of intracranial hypertension with a benefit in terms of oxygenation without major increase of ICP particularly in PP responders.

**Competing interests** None.

**References**

1. Roth C, Ferbert A, Deinsberger W, Kleffmann J, Kästner S, Godau J, et al. Does prone positioning increase intracranial pressure? A retrospective analysis of patients with acute brain injury and acute respiratory failure. Neurocrit Care. 2014;21(2):186–91.
2. Thelandersson A, Cider A, Nellgård B. Prone position in mechanically ventilated patients with reduced intracranial compliance. Acta Anaesthesiol Scand. 2006;50(8):937–41.

#### S64 Bacterial co-infection in influenza-associated ARDS supported by ECMO

##### Sacha Rozencwajg^1^, Matthieu Schmidt^1^, Guillaume Hekimian^1^, Nicolas Bréchot^1^, Jean Louis Trouillet^1^, Guillaume Lebreton^2^, Sébastien Besset,^1^, Guillaume Franchineau^1^, Ania Nieszkowska^1^, Leprince Pascal^2^, Alain Combes^1^, Charles-Edouard Luyt^1^

###### ^1^Service de réanimation médicale, Groupe Hospitalier Pitié-Salpêtrière, Institut de Cardiométabolisme et Nutrition, Paris, France; ^2^Service de chirurgie thoracique et cardiovasculaire, Groupe Hospitalier Pitié Salpêtrière, Paris, France

####### **Correspondence:** Sacha Rozencwajg - sacha.rozencwajg@gmail.com

*Annals of Intensive Care* 2017, **7**(**Suppl 1**):S64

**Introduction** Influenza-associated acute respiratory distress syndrome (ARDS) requiring extracorporeal membrane oxygenation (ECMO) support is known to have a good prognosis (1). However, the incidence and impact of co-infection in this setting remain unknown.

**Patients and methods** We conducted a retrospective, observational analysis of data prospectively collected from all patients admitted to our medical ICU who received ECMO support for influenza-associated ARDS between 2009 and 2016. Co-infection was defined as isolation of a pathogen in the lower respiratory tract at a significant level or in the blood during the 48 h following hospital admission. When no pathogen was identified in a patient receiving antibiotics prior to bacteriological sampling, an independent adjudication committee reviewed all charts to assess if the patient had a “high probability” or “low probability” for bacterial co-infection, based on clinical, radiological and biological results available. Results are presented as median [IQR].

**Results** Among the 116 patients hospitalized for an influenza-associated infection in our ICU, 77 had an ARDS requiring support by either veno-venous- (VV, n = 58), venoarterial (VA, n = 15) or venoarterio-venous- (VAV; n = 4) ECMO. Co-infection occurred in 39 (51%) patients, *Staphylococcus aureus* (18 patients [46%]) being the most prevalent pathogen. Panton-Valentin leucocidin (PVL) was present in 10 (56%) of *S. aureus* co-infection, representing 26% of all co-infections. Compared to primary viral pneumonia, co-infected patients had a lower BMI (26 [24–30] vs 31 [8–38] kg/m^2^; p < 0.0001), a higher Sequential Organ Failure Assessment (SOFA) score before ECMO start (16 [14–18] vs 12 [10–16]; p = 0.001), and received less antibiotics before hospital admission (26 vs 51%; p = 0.03). No differences were observed in age, comorbidities, ARDS management before and after ECMO, influenza characteristics (genotype, sub-type, neuraminidase inhibitors use, delay between symptoms onset and hospital or ICU admission), ECMO management (timing of initiation, VV-, VA or VAV- configuration). Co-infection was associated with increased ICU mortality (62 vs 29%; OR 3.93; 95% CI [1.51–10.18], p = 0.0059), lower ECMO free days at day 60 (0 [0–23] vs 23 [0–46]; p = 0.006) and lower mechanical ventilation free days at 60 days (0 [0–0] vs 6 [0–35]; p = 0.003). Multivariable analysis retained age >49 years (OR 8.2; 95% CI 2.3–29.9), pre-ECMO SOFA score >14 (OR 6.2; 95% CI 1.9–19.8) as independent predictors of hospital mortality, but not co-infection (OR 2.5, 95% CI 0.8–7.9). In a second analysis, patients with proven co-infection and high probability of co-infection were grouped and compared to patients with no co-infection and low probability of co-infection; and results were similar. As compared to others co-infected patients, those co-infected with a PVL-positive *S. aureus* had same characteristics and similar mortality rate, but all received a treatment active against PVL production.

**Conclusion** Co-infection is frequent in patients with influenza-associated ARDS supported by ECMO, occurring in roughly 50% of the cases. Mortality of patients with co-infection is higher than those without, but seems mainly due to the severity of the disease. *S. aureus* was the most frequently identified pathogen, with a high prevalence of PVL-positive *S. aureus*, Infection with a PVL-positive strain was not associated with a poorer outcome as compared to other co-infections. Whether a treatment active against PVL production should be given in those patients remains to be determined.

**Competing interests** None.

#### S65 Acute respiratory failure (ARF) in patients with systemic rheumatic disease

##### Maud Loiselle^1^, Guillaume Dumas^2^, Guillaume Geri^3^, Chemam Sarah^4^, Dangers Laurence^5^, Claire Pichereau^6^, Julien Mayaux^7^, Maleka Schenck^8^, Boisramé-Helms Julie^8^, Thomas Guillemette^9^, Claude Guérin^10^, Sylvie Chevret^11^, Jean-Daniel Chiche^3^, Elie Azoulay^2^

###### ^1^Réanimation médicale, Hôpital Saint-Louis (AP-HP), Paris, France; ^2^Réanimation médicale, Hôpital Saint-Louis, Paris, France; ^3^Réanimation Médicale, Hôpital Cochin, Paris, France; ^4^Réanimation médicale, Hôpital Bichat-Claude Bernard (AP-HP), Paris, France; ^5^Réanimation médicale, Pitié-Salpêtrière Hospital, Paris, France; ^6^Réanimation médicale, Hôpital Saint-Antoine, Paris, France; ^7^Réanimation médicale, Hôpital Pitié-Salpêtrière, Paris, France; ^8^Réanimation médicale, C.H.R.U. Hôpitaux Universitaires Strasbourg, Strasbourg, France; ^9^Service de réanimation-détresses respiratoires et infections sévères, Hôpital Nord, Marseille, France; ^10^Réanimation médicale, Hôpital de la Croix-Rousse, Lyon, France; ^11^Service de biostatistique et information médicale, Hôpital Saint-Louis, Paris, France

####### **Correspondence:** Maud Loiselle - maud.loiselle@outlook.fr

*Annals of Intensive Care* 2017, **7**(**Suppl 1**):S65

**Introduction** Systemic rheumatic diseases (SRD) are autoimmune diseases that are rare but cause substantial morbidity and mortality. SRDs chiefly affect the lungs, however, data on critically ill patients with SRD admitted for ARF are scarce.

**Patients and methods** Retrospective cohort conducted in 10 French ICUs (2009–2013) and prospectively completed in the coordinating center until June 2016. Demographic, clinical and therapeutic data were collected. Predictors of hospital mortality were identified by multivariable logistic regression.

**Results** 226 patients totalizing 237 ICU admissions were included (median age 61 [47–71] years, female gender 62%). SRDs were connective tissue diseases in 63% of cases (Systemic sclerosis 19.5%, Systemic lupus erythematous 18%) and systemic vasculitis in 31% of cases. Nearly 5% of patients had antiphospholipid syndrome. Time since SRD onset was 7.5 [2.0–15.8] years with median involvement of two organs [1.0–4.0]. The major comorbidities were cardiovascular (49%), tobacco exposure (25%), chronic kidney disease (18%) and neoplasia (15%). Two-thirds of patients were on systemic corticosteroids at admission, the median dose of 10 (IQR) mg per day. SRD diagnosis was made in the ICU in 11.5% of patients.

Clinically or microbiologically documented bacterial pneumonia was the leading ARF etiology (40.9%). In 32% of cases, ARF was related to an opportunistic infection (mainly Aspergillus (n = 8) and Pneumocystis (n = 7)). Others ARF etiologies included specific lung involvement (23.2%) and cardiac pulmonary edema (20.7%).

SOFA on day one was 5 [3–9]. Associated organ dysfunctions were mainly hemodynamic (42%) and renal (45%). Mechanical ventilation was needed in 68% of patients (non invasive only in 17.3% or invasive in 82.7%), 40% needed vasopressors, and 30% renal replacement therapy. Systemic corticosteroids were started in 20% of patients and 23% of patients received pulse steroids. Cyclophosphamide and plasma exchange were required in 10 and 11% of patients, respectively.

Length of ICU stay was 7 [4–14] days. ICU-acquired infection occurs in 25% of cases. In total, 59 patients (24.9%) died throughout the ICU stay. ARF etiology was not associated with mortality. By multivariate analysis, shock on admission (OR 6.25 [2.71–14.40], p < 0.001) and the use of invasive mechanical ventilation (OR 5.06 [1.84–13.94], p = 0.02) were independently associated with mortality, whereas non-invasive ventilation was associated with decreased mortality (OR 0.31 [0.12–0.81], p = 0.02). By considering among the connective tissue diseases, the groups of myositis and scleroderma (n = 61), these diseases were associated with a trend for a higher mortality (OR 2.08 [0.95–4.55], p = 0.06).

**Conclusion** In patients with SRD, ARF is associated with a high case fatality, primarily when mechanical ventilation is needed. Particular attention must be given to specific SRD-sub groups for which pulmonary flare may require intensive immunosuppression.

**Competing interests** None.

#### S67 Pneumonia during pregnancy and the post partum period: a reappraisal of etiologies requiring intermediate or intensive care—a retrospective study through the CUBREA network

##### Alice Jacquens^1^, Sebastien Kerever^2^, Bertrand Guidet^3^, Philippe Aegerter^4^, Vincent Das^5^, Alain Cariou^6^, Muriel Fartoukh^7^, Jan Hayon^8^, Alain Combes^9^, Etienne De Montmollin^10^, Christian Richard^11^, Mathieu Desmard^12^, Bruno Megarbane^13^, Jean-Pierre Fulgencio^7^, Benjamin Zuber^14^, Benjamin Sztrymf^15^, Jean-Damien Ricard^16^, Jonathan Messika^17^, Cubrea network

###### ^1^Réanimation médico-chirurgicale, Hôpital Louis-Mourier - APHP, Colombes, France; ^2^Département d’anesthésie réanimation, GH St Louis/Lariboisière AP-HP, Paris, France; ^3^Réanimation Médicale, Hôpital Saint-Antoine, Paris, France; ^4^Urc, Hospital Ambroise Paré, Boulogne-Billancourt, France; ^5^Réanimation polyvalente adulte, Centre Hospitalier Intercommunal André Grégoire, Montreuil, France; ^6^Réanimation Médicale, Hôpital Cochin, Paris, France; ^7^Réanimation médico-chirurgicale, Hôpital Tenon, Paris, France; ^8^Réanimation polyvalente, CHI Poissy-St Germain, Poissy, France; ^9^Service de Réanimation Médicale, Groupe Hospitalier Pitié Salpêtrière, Paris, France; ^10^Réanimation, C.H. Général Saint Denis hôpital Delafontaine, Saint-Denis, France; ^11^Réanimation médicale, CHU de Bicêtre, Le Kremlin Bicêtre, France; ^12^Réanimation polyvalente, Centre Hospitalier Sud Francilien, Boulevard Jean Jaurès, Corbeil-Essonnes, France, Corbeil-Essonnes, France; ^13^Service de Réanimation Médicale et Toxicologique, CHU Lariboisière, Paris, France; ^14^Intensive care unit, Hospital Center De Versailles, Le Chesnay, France; ^15^Réanimation polyvalente, Hôpital Antoine Béclère, Clamart, France; ^16^Service de Réanimation Médico-Chirurgicale, CHU Louis Mourier, Colombes, France; ^17^Service de réanimation médico-chirurgicale, CHU Louis Mourier, Colombes, Colombes, France

####### **Correspondence:** Jonathan Messika - jonathan.messika@aphp.fr

*Annals of Intensive Care* 2017, **7**(**Suppl 1**):S67

**Introduction** Infections are among the main causes of non-obstetric morbidity and mortality during pregnancy and the postpartum period, and the second causes of intensive care unit (ICU) admissions for non-obstetric reasons. Among those, pneumonia is of particular interest, as pregnancy impairs the ventilatory function, and blunts the immune response. In addition, pneumonia might increase fetal morbi-mortality. We, therefore, sought to investigate the epidemiology of community-acquired pneumonia (CAP) occurring during pregnancy or postpartum (until 6 weeks postpartum) requiring ICU or intermediate care unit admission.

**Patients and methods** We conducted a retrospective multicenter observational study in 41 Paris and suburban area ICUs participating in the CUBREA database (January 2005–December 2014). Women were identified through the ICD-10 database with an obstetric code (O00–O99 or Z35–Z39) and an acute respiratory failure (J96) or pneumonia code (J10–J22). Charts were reviewed. The primary outcome was to determine the rate of CAP diagnoses within this population admitted for acute respiratory failure. The secondary outcomes were to compare CAP and non-CAP women, regarding comorbid conditions, ICU course, therapies and fetal outcome; and finally to describe the etiologies of CAP episodes.

**Results** Our search retrieved 367 women pregnant or during the postpartum period. Seventy-one of those (22.5%) had a CAP, and had been admitted in the intermediate (11; 15.5%) or intensive (60; 84.5%) care units. CAP women had a median age of 32 years [28–37] (vs. 32 [27–36] for non-CAP), and a median SAPSII score of 21 [13–29] (vs. 23 [16–41] for non-CAP). Thirty-four harbored at least one comorbid condition (47.9 vs. 36.9%; p = 0.10), and they were more frequently active smokers (14.7 vs. 7%; p = 0.044) than non-CAP women. Fifty-one CAP women were admitted during prepartum (71.8 vs. 36.21% for non-CAP; p < 0.0001), 7 in intermediate care unit, and 44 in ICU, at 31 weeks [26–33] of gestational age, while 20 (4 in intermediate care unit, and 16 in ICU) were admitted during the postpartum period (28.2 vs. 63.8% for non-CAP). Thirty (42.3 vs. 54.3% for non-CAP; p = 0.08) underwent mechanical ventilation, 12 (16.9 vs. 11.5% for non-CAP; p = 0.23) non-invasive ventilation and 7 (9.9 vs. 2.9% for non-CAP; p = 0.001) high-flow nasal cannula oxygen therapy. Compared to non-CAP, CAP women had longer duration of mechanical ventilation (7 days [4–13] vs. 2 [1–5]; p < 0.0001). Acute respiratory distress syndrome occurred in 20 CAP women (28.2 vs. 3.7% for non-CAP; p < 0.0001), and 6 required extra-corporeal membrane oxygenation (8.4 vs. 4.5% for non-CAP; p = 0.23). Preterm extraction occurred for maternal reasons in 15 CAP women (21.1 vs. 7.8% for non-CAP; p = 0.004), and more frequently by cesarean section (84.2 vs. 53% for non-CAP; p = 0.0003). Fetal survival did not differ significantly, as live births occurred in 85 vs. 77.4% for non-CAP (p = 0.39). Length of ICU and hospital stays were significantly longer in CAP compared to non-CAP women (respectively 4 [2–8] vs. 3 [1–5] days; p = 0.001), and 12 [7–22] vs. 9 [4–15] days; p = 0.001). ICU and hospital mortality were null for CAP versus 5.8 and 6.6% for non-CAP (respectively p = 0.045 and p = 0.027). Regarding CAP microbial etiologies, no causative pathogen was found in 25 CAP subjects (39.7%); Influenza was identified alone in 18 (25.7%), *Streptococcus pneumoniae* in 6 (8.6%), *Haemophilus sp.* in 3 (4.3%), methicilin-resistant *Staphylococcus aureus* in 2 (2.3%) and methicillin-susceptible *S. aureus* in one (1.4%). Finally, another respiratory virus was diagnosed in 3 patients (4.3%).

**Discussion** In this series, CAP was the cause of nearly a quarter of pregnancy or postpartum respiratory failure requiring intermediate or intensive care unit admission. Despite a longer ICU and hospital stay, no woman died. As in the non-pregnant population, the absence of any causative pathogen is frequent. Influenza was the most frequent pathogen identified.

**Conclusion** Pneumonia during pregnancy and postpartum requiring intermediate or intensive care unit has important maternal and fetal implications. As such, a rigorous multidisciplinary approach is warranted.

**Competing interests** None.

#### S68 Postoperative morbidity of duodeno pancreatectomy in surgical intensive care unit

##### M. T. Slaoui^1^, M. A. Bouhouri, A. Soufi, K. Khaleq, D. Hamoudi, A. Nsiri, R. Harrar

###### ^1^Reanimation des urgences chirurgicale, chu ibn rochd, Casablanca, Morocco

####### **Correspondence:** Mohamed Taoufik Slaoui - dr.t.slaoui@gmail.com

*Annals of Intensive Care* 2017, **7**(**Suppl 1**):S68

**Introduction** The pancreaticoduodenectomy (PD) is major surgery in visceral surgery. This technique performed for the first time in 1935 by Whipple has seen much progress and development over the years that have enabled a significant reduction in mortality, while the morbidity remains high. The aim of this study was to analyze postoperative morbidity pancreaticoduodenectomies.

**Patients and methods** We retrospectively studied 34 cases of cephalic duodenopancreatectomy at the department of surgical emergencies resuscitation (wing 33) spanning 6 years, between January 2010 and December 2015.

**Results** The average age of patients was 58.2 years with 43% of females and 57% of males, the frequence of pancreatic resections was 5 years.

The indications of cephalic duodenopancreatectomy were: tumors of pancreatic head (65%), Ampulla Vater (23%), duodenum tumors (12%).

The restoration of continuity after cephalic duodenopancreatectomy was realized with a rate of 41% for pancreaticogastrostomy and 59% for pancreaticojejunostomy.

The average hospital stay was 11, 7 days, with extreme lengths of 6–27 days.

The postoperative course was marked by the occurrence of 8 deaths (24%), the morbidity rate was 29, 25% after PJ and 28% after PG; the most frequent complications were the pancreatic fistula (40%), the postoperative peritonitis (40%), the digestive bleeding (20%), the gastroparesis (20%).

**Conclusion** Advances in the overall care of patients by surgical teams, anesthesiologists and intensivists, the DPC mortality is currently low in experienced centers. The multidisciplinary, involving surgeons, radiologists and especially intensive care, to manage more effectively the complications of this surgery remains burdened with high morbidity.

**Competing interests** None.

#### S69 Severe acute pancreatitis in ICU: management and outcomes of infected pancreatic necrosis

##### Charlotte Garret^1^, Matthieu Peron^2^, Emmanuel Coron^2^, Cédric Bretonnière^1^, Jean Reignier^1^, Christophe Guitton^1^

###### ^1^Réanimation médicale, CHU Hôtel-Dieu Nantes, Nantes, France; ^2^Hépato-gastroentérologie, CHU Hôtel-Dieu Nantes, Nantes, France

####### **Correspondence:** Charlotte Garret - charlottegarret83@gmail.com

*Annals of Intensive Care* 2017, **7**(**Suppl 1**):S69

**Introduction** Severe Acute Pancreatitis (SAP) is a common but potentially lethal pathology due to the multiplicity and severity of complications that can occur at all stages of evolution. In the last decade, mini-invasive interventional treatments of infected pancreatic necrosis (IPN) have been developed. The aim of the present study was to assess the management and outcomes of SAP patients, as well as to identify the role of IPN.

**Materials and methods** This was a retrospective study of prospectively collected data from all consecutive patients admitted in Intensive Care Unit (ICU) in a single French center (Hospital of Nantes) from 2012 to 2015. Using logistic regression, we evaluated the association between IPN and patients characteristics at baseline and the outcomes.

**Fig. 19:**
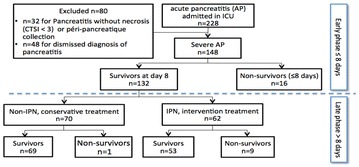
Flow chart of the study and details from mortality during the early phase and details from mortality of the two groups: infected pancreatic necrosis (IPN) and non-IPN patients during the late phase of the onset of severe acute pancreatitis

**Results** In total, 148 patients were included in this study. Overall mortality was 17% and was significantly associated in multivariate analysis with BMI, Computed Tomography Severity Index (CTSI) and the need for life sustaining support. 16 patients (11%) died during the early phase ≤8 days. During the late phase of the onset of SAP (>8 days), IPN was present in 62 patients, all requiring an intervention (i.e. radiologic, endoscopic, and surgical). In contrast, 70 patients with no suspicion or confirmed IPN were managed conservatively. The flow chart of the study and details from mortality during the early and the late phase are details Fig. 19. Complications such as haemorrhage, perforation of visceral organ, ischemia of enteric tube or colon and portosplenomesenteric venous thrombosis (PSMVT) occurred more frequently in the IPN group. The late phase mortality and hospitalisation length of stay were significantly higher in the IPN group (14.3 vs 3.6%, p = 0.01 and 65.5 vs 15 days, p < 0.001, respectively). In multivariate analysis, factors associated with IPN were PSMVT (OR 6.0 [2.42–14.90], p < 0.001), and life sustaining support (OR 5.72 [1.06–30.9], p = 0.04).

**Discussion** This retrospective cohort reports an overall mortality slightly lower than previously reported. This may be related to the development of novel life support techniques and mini-invasive interventional treatments of IPN. The outcomes in term of late phase mortality, length of stay and complications (i.e. haemorrhage, perforation of visceral organ and mesenteric ischemia) are all strongly associated with IPN. Therefore, to determine predictive factors of IPN may help identify patients with a high morbidity and mortality. In multivariate analysis, organ failure during the first week and PSMVT were strongly associated with IPN. The radiological findings in term of PSMVT could be important to assess the degree of severity of patients with AP. Further prospective studies are needed to confirm the association between these factors and IPN.

**Conclusion** Our study is the first to focus on mortality and outcomes of IPN in real life since the development of mini-invasive interventional treatments. 43% of patients admitted with severe SAP in ICU developed IPN and required intervention. IPN was managed with different drainage and necrosectomy’s modalities. The late phase mortality, length of stays and complications such as PSMVT were all significantly higher in the IPN group.

**Competing interests** None.

#### S70 Severe acute pancreatitis in intensive care unit

##### M. A. Bouhouri^1^, M. T. Slaoui^1^, A. Soufi, K. Khaleq, D. Hamoudi, A. Nsiri, R. Harrar

###### ^1^Reanimation des urgences chirurgicale, chu ibn rochd, Casablanca, Morocco

####### **Correspondence:** Mohamed Taoufik Slaoui - dr.t.slaoui@gmail.com

*Annals of Intensive Care* 2017, **7**(**Suppl 1**):S70

**Introduction** The acute pancreatitis appears as a pathology that we can define with difficulty because of its clinical presentation or prognosis.

**Patients and methods** In our study, we analysed 102 cases of acute necrotic and hemorrhagic acute pancreatitis, hospitalized at the department of resuscitation of the surgical emergencies (P33) of the UHC Ibn Rochd Casablanca during the period (2009–2015).

The purpose of this study is to do a descriptive analysis of the epidemiologic, clinic, radiological, therapeutic and evolutive data of the acute necrotic pancreatitits, we included in our study 102 patients with epidemiologic, clinic, radiologic, biologic criteria of acute necrotic pancreatitits diagnosis whatever is the biliary or alcoholic etiology.

The valuation gravity of the pancreatitis has been based on:

- Ranson bioclinical score >3/APACHE II >8;
- Visceral failure.
- Spreading of the necrosis.

**Results** The analysis of the results shows that: About the epidemiologic aspect: mean age (52 year old), the biliary etiology predominates (81%).

About the clinical aspect: pain (95%) vomiting (77%), stop of the transit (18%), the visceral distresses are: the shock (34%), respiratory distress (40%), and neurological distress (28%).

About the radiological aspect: pleural effusion (42%), abdominal echography: vesicular lithiasis (60%), dilated principal biliary duct (13%), abdominal computerized tomography: stage E (49%).

About the biological aspect: hyperglycemia (80%), hyper-amylasemia (68%).

The indexes of gravity that have been appreciated in this study are: Ranson score >3 (58%), Imrie score >3 (66%), IGS score ≥6 (47%), OSF score ≥1 (70%).

The treatment of the ANHP has been symptomatic in particular and the evolution has been characterized by mortality about 38%, the cause was particularly infectious.

The prognostic factors predetermined in this study are:

- Female type (p = 0.0004).
- Hemodynamic distress (p = 0.00005).
- Respiratory distress (p = 0.0000001).
- Scores of gravity:
  - Ranson >3 (p = 0.0000001).
  - Imrie >3 (p = 0.02).
  - OSF ≥ 1 (p = 00001).
- Infection (p = 0.0000001).
- Duration of the hospitalization (p = 0.02).
- Rate of C-reagent protein (p = 0.001).

**Conclusion** In conclusion, the mortality is still high in the ANHP, considerable effort of search is necessary to prevent the infectious complications of mortality.

**Competing interests** None.

#### S71 Predicting 90-day mortality following liver transplantation in patients with Acute-On-Chronic Liver Failure: a decision-tree model from the French national liver transplantation system, the OPTIMATCH study, 2008–2014

##### Eric Levesque^1^, Etienne Audureau^2^, Winters Audrey^3^, Duvoux Christophe^4^, Jacquelinet Christian^5^, Azoulay Daniel^6^, Feray Cyrille^4^, Optimatch group

###### ^1^Anesthesia and intensive care medicine, CHU Henri Mondor, Créteil, France; ^2^Service de santé publique, ap-hp hôpital henri mondor; ea7376 cepia, université paris est crétei, ap-hp hôpital henri mondor, Créteil, France; ^3^Upres ea 2415 département de biostatistiques, Université de Montpellier, Montpellier, France; ^4^Service d’hépatologie, Hôpital Henri Mondor, Créteil, France; ^5^Aence de la biomédecine, Agence de la biomédecine, Saint-Denis, France; ^6^Service de chirurgie digestive et transplantation hépatique, Hôpital Henri Mondor, Créteil, France

####### **Correspondence:** Eric Levesque - eric.levesque@aphp.fr

*Annals of Intensive Care* 2017, **7**(**Suppl 1**):S71

**Introduction** Cirrhotic patients undergoing an initial liver transplantation (LT) have a 1-year survival exceeding 85%. Some cirrhotic patients present organ failures at the time of LT defining Acute-On-Chronic Liver Failure (ACLF). The aim of this study was to build a decision-tree algorithm for predicting 90-day mortality in transplanted patients, by assessing the independent prognostic contribution of ACLF.

**Patients and methods** All patients transplanted between 2008 and 2014 (N = 4789) were included as part of the OPTIMATCH study, yielding 4010 patients with complete data for the present analysis. Data collected comprised clinical and biological features at the time of LT for recipients and their donors, assessing ACLF status according to ACLF/CANONIC criteria to categorize patients. A survival Classification and Regression Analysis (CART) algorithm was applied to build the prognostic model for 90-day mortality.

**Fig. 20:**
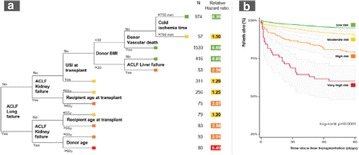
Decision tree freom CART survival analysis (**a**) and corresponding Kaplan–Meier curves (**b**)

**Results** 1657 patients (41%) met the CANONIC criteria of ACLF with at least one organ failure (1: 20.3%; 2: 12.6%; 3 and more: 8.4%). Overall 90-day mortality rate was 7.6%, with corresponding rates of 5.4, 7.2, 10.2 and 20.1% in patients with 0, 1, 2, 3 and more organ failures, respectively. Decision-tree modelling identified 12 subgroups further classified in 4 increasing risk classes (Fig. 20), highlighting the prognostic importance of respiratory failure and acute renal failure at the time of LT, as well as complex interactions between donor and recipient features.

**Discussion**

**Conclusion** Ventilator support and/or acute renal failure at the time of LT are major predictors of mortality but complex recipients/donors relationships may moderate these associations, as demonstrated by our CART analysis.

**Competing interests** None.

#### S72 Morbidity and mortality of gastrectomy for cancer in surgical ICU

##### Chaigar Mohammed Cheikh^1^, Wissal Aissaoui^1^, Kawtar Rghioui^1^, Wafae Haddad^1^, Rachid Cherkab^1^, Houcine Barrou^1^

###### ^1^Anesthésie réanimation, Chu Ibn Rochd, Casablanca, Morocco

####### **Correspondence:** Chaigar Mohammed Cheikh - chaigarmed@gmail.com

*Annals of Intensive Care* 2017, **7**(**Suppl 1**):S72

**Introduction** Evaluation of morbidity and mortality in ICU is crucial to determine risk factors and improve clinical outcome. The aim of our study was to identify risk factors of postoperative morbidity and mortality of gastrectomy for cancer.

**Patients and methods** We conducted a retrospective monocentric study in surgical ICU of UH Ibn Rochd of Casablanca. Were included all patients admitted for gastrectomy for cancer between January 2014 and December 2015. Clinical, radiological and biological data was collected. Patients were categorized into 2 groups: Group A including patients who developed postoperative complications and Group B including patients who didn’t. The qualitative data were expressed as absolute value and percentage. Quantitative data were expressed as mean. Categorical variables were compared by a Chi2 test or Fischer test. Comparing the quantitative variables was performed by a Student’s t test. Odds ratios were calculated with 95% confidence interval and a difference was considered significant if p < 0.05. Multivariate analysis was performed by a test logistic regression. Statistical analysis was performed using the Epi-Info software 3.5.4.

**Results** Sixty patients were enrolled. The sex-ratio was 1.7. The mean age was 59.8 ± 11.7 years. The mean ASA score was 2 (± 0.7). Eight patients (13.3%) had a medical history of diabetes. Nineteen patients (31.7%) had cardiovascular diseases (high blood pressure, arythmia, heart failure). Seven patients (11.7%) had at least one family history of gastrointestinal cancer in a related. The postoperative body mass index (BMI) was 23.2 ± 3.2 kg/m^2^. The mean albumin level was 31 +/9.3 g/l. Twenty-two patients (35%) suffered from malnutrition and had received preoperative nutritional rehabilitation. The most frequently reported tumor location was the gastric antrum (40%). Five patients (8.3%) had a pan-gastric extension. Preoperative chemotherapy was performed in 18.3% of patients. Total gastrectomy was performed in 28 patients (46.7%). Twenty-three had partial gastrectomy (2/3), and 9 subtotal gastrectomy (4/5). Enlarged gastrectomy was performed in 3 patients (5%). The mean operative time was 244.3 ± 58 min. Per-operative transfusion was required in 14 patients (23.3%). The average length of stay in ICU was 8.2 ± 5 days. Postoperative mortality was 6.7%. In our series, 13 patients (21.7%) had at least one postoperative complication: An anastomotic fistula diagnosed in 7 patients (11.7%), 12 patients (11.3%) had postoperative peritonitis and 6 patients had ventilator associated pneumonia. Reoperation was necessary for 13 patients (21.7%), it was performed after 7.2 days (2–18 days). In univariate analysis, risk factors for postoperative morbidity after gastrectomy was hypoalbuminemia (p = 0.0145), anemia (p = 0.0001), BMI (p = 0.001) and malnutrition (p = 0.007). Age, sex, neoadjuvant chemotherapy, extended lymphadenectomy, splenectomy or pancreatosplenectomy, total gastrectomy and operative time were not significantly associated with higher postoperative morbidity. In multivariate analysis, malnutrition (p = 0.01) and BMI (p = 0.001) were significantly associated with the occurrence of postoperative complications.

**Conclusion** The results of our study are similar to those reported in medical literature. Preoperative evaluation and nutritional rehabilitation are crucial to improve patient’s outcome and reduce morbidity and mortality after gastrectomy for cancer.

**Competing interests** None.

#### S73 Assessment of the management of acute mesenteric ischemia in intensive care unit

##### M. T. Slaoui^1^, M. A. Bouhouri, A. Soufi, K. Khaleq, D. Hamoudi, A. Nsiri, R. Harrar

###### ^1^Reanimation des urgences chirurgicale, chu ibn rochd, Casablanca, Morocco

####### **Correspondence:** Mohamed Taoufik Slaoui - dr.t.slaoui@gmail.com

*Annals of Intensive Care* 2017, **7**(**Suppl 1**):S73

**Introduction** The mesenteric ischemia is a condition relatively rarely. It is marked by high mortality. Mortality is primarily related to the land on which ischemia occurs and especially the time taken to diagnose. This delay is due to the low specificity of clinical signs and the absence of diagnostic laboratory test. The mesenteric ischemia remains a diagnostic and therapeutic challenge.

**Patients and methods** Twenty cases of acute mesenteric ischemia have been collected at the surgical resuscitation (resuscitation 33) at the hospital center IBN ROCHD of Casablanca from January 2010 to December 2015.

**Results** The mean age of our patients is 50 year old. It is about a disease that the incidence increases these last years, particularly because of the waxing number of old patients and/or suffers from advanced cardiovascular diseases.

The cardiovascular risk factor has been present in 35% of our patients.

The abdominal pain has been present in all the patients. It is a sudden, intensive pain localized the most often at the level of the epigastria, becomes diffuse in few hours or even few days. Other clinical signs have been described as the bilious vomiting that becomes fecaloid after few days. The digestive hemorrhages as the moelena and the hematemeses. A stop of the matter and the gazes was noticed in 25% of our patients.

The absence of specificity of the clinical signs forced the realization of complementary examinations.

The scanner becomes the reference imaging. It permits a differential diagnosis, the search of direct signs of vascular obstruction and the emphasis of intestinal pain.

Four etiologies are noticed: The arterial occlusion by emboli (40%), the arterial thrombosis (20%), the venous thrombosis (25%) and the “non occlusive” form (15%).

The strategy of management of the acute mesenteric ischemia is multidisciplinary, based on the equips of radiology, vascular surgery and/or visceral surgery and resuscitation.

The treatment consists in measures of general resuscitation, the techniques of endoluminal vascular disobstruction and techniques of surgical revascularization.

In spite of the improvements in the diagnosis and the therapeutic procedure of the IMA, the disease still know a rate of mortality between 60 and 97% according the studies.

In our study, we noticed 8 cases of death (40%), 9 cases of good recovery (45%), 3 cases are unknown evolution (15%).

**Conclusion** It is a vital emergency that the evolution still knows great mortality. It is very important to remind the acute mesenteric ischemia in the case of any acute abdominal symptom in order to anticipate about the natural evolution and to act in a reversible stage of the ischemia.

**Competing interests** None.

#### S74 Emergency digestive surgery in critically ill patients under Extracorporeal Membrane Oxygenation: a cohort study

##### Anna Carteaux-Taeib^1^, Romain Deransy^2^, Renato Lupinacci^1^, Gilles Manceau^3^, Florence Jeune^3^, Alexandre Demoule^4^, Christophe Tresallet^1^, Alain Combes^5^, Nicolas Bréchot^5^

###### ^1^Chirurgie digestive, Groupe Hospitalier Pitié-Salpêtrière, Paris, France; ^2^Ranimation chirurgicale polyvalente, Groupe Hospitalier Pitié-Salpêtrière, Paris, France; ^3^Chirurgie digestive et hépato-bilaire, Groupe Hospitalier Pitié-Salpêtrière, Paris, France; ^4^Service de pneumologie et réanimation médicale, Groupe Hospitalier Pitié-Salpêtrière, Paris, France; ^5^Service de réanimation médicale, Groupe Hospitalier Pitié-Salpêtrière, Institut de Cardiométabolisme et Nutrition, Paris, France

####### **Correspondence:** Nicolas Bréchot - nicolas.brechot@aphp.fr

*Annals of Intensive Care* 2017, **7**(**Suppl 1**):S74

**Introduction** Extracorporeal Membrane Oxygenation (ECMO) is responsible for hemostatic disorders, increasing the risk of bleeding during invasive procedures. Particularly, outcome of patients undergoing digestive surgery while on ECMO has been poorly reported to date. This study aimed to describe the outcome of critically ill ECMO patients undergoing emergent digestive surgery, in comparison to a population of non-ECMO patients operated in the same unit.

**Materials and methods** Retrospective single-center study.

**Results** Data are expressed as % or median (IQR).

Between 2006 and 2014, 35 patients with ECMO and 42 patients without ECMO underwent emergency digestive surgery. The main indications were intestinal ischemia (43% ECMO vs. 41% non-ECMO, ns) and cholecystectomy (23 vs. 26%, ns). Patients on ECMO were younger [59 (43–64) vs. 64 (54–74) years; p = 0.02] but had a higher preoperative SAPS II score [79 (70–90) vs. 71 (44–83); p = 0.03]. There were significantly more reinterventions for bleeding in the ECMO group (20 vs. 2%, p = 0.02) and a greater need for blood transfusion within 72 h after surgery (13 packed red blood cells (6–22) vs. 3 (0–5); p < 0.0001). In multivariate analysis, ECMO remained strongly associated with the occurrence of bleeding (OR 5.6; CI [0.2–15.4]; p = 0.001). ICU mortality was significantly higher in patients under ECMO (69 vs. 33%; p = 0.003) but perioperative mortality remains comparable between groups (14 vs. 12%, ns). These results were confirmed in a sensitivity analysis in 36 ECMO and non-ECMO patients matched on the pre-operative SAPS-II score, age, and type of digestive surgery.

**Conclusion** In critically ill patients undergoing emergent digestive surgery, ECMO is associated with an increased risk of bleeding but not with perioperative mortality.

**Competing interests** None.

#### S75 Burnout risk factors in emergency: multicentric investigation

##### Sahar Habacha^1^, Ines Fathallah^1^, Aymen Zoubli^1^, Rafaa Aloui^1^, Nadia Kouraichi^1^

###### ^1^Intensive care unit, regional hospital of Ben Arous, Tunis, Tunisia

####### **Correspondence:** Ines Fathallah - ines822004@yahoo.fr

*Annals of Intensive Care* 2017, **7**(**Suppl 1**):S75

**Introduction** Emergency Departments staff are frequently exposed to many complex stressful situations and consequently burnout syndrome. Our study aimed to describe epidemiological particularities and determine the risk factors of burnout syndrome in different categories of emergency.

**Patients and methods** We studied five academics and four regional hospitals. The level of burnout was assessed using the “Maslach Burn Out Inventory” score and the degree of depression with Major Depression Inventory (MDI) test.

**Results** One hundred and forty-three correctly completed questionnaires were collected. The mean age of study population was 32 ± 7 years. Sex-ratio was at 0.63. Fifty-one per cent of the care staff were married. Physicians represented 40% and paramedical 60%. The general frequency of Burnout syndrome was 85% (n = 127). Low level Burnout was present in 32%, moderate level in 45% and high level in 23%. The depression frequency was 52%.

A statistically significant correlation was found between burnout and depression firstly (*p* = 0.006) and between burnout and lack of equipment (*p* = 0.04). Their relative risk was 4.7 [1.5, 15] and 9.14 [2.2, 37.6] respectively).

Main risk factors associated with high level burnout are detailed in Table 22.

**Table 22:** Factors associated with high level burnout

Factors	High level bunout (N = 29)	No high level burnout (N = 114)	P	RR	95% CI
Conflictual situations	29 (100%)	85 (74%)	0.19	–	–
*Civil state: married*	21 (72%)	55 (48%)	*0.036*	*2.245*	*[1.067, 4.724]*
*Working in Academic hospital*	20 (68%)	44 (38%)	*0.06*	*9.708*	*[1.326, 5.530]*
Medical staff	8 (27%)	51 (44%)	*0.8*	**–**	**–**
Heavy workload	21 (72%)	114 (100%)	0.4	–	–
Empathy regard to patients	9 (31%)	72 (63%)	0.14	–	–

**Conclusion** Burnout syndrome frequency in our emergency departments is alarming. Helping to resolve social and psychological problems and improving work conditions may help to decrease it.

**Competing interests** None.

#### S76 Let us resuscitate our waste

##### Emilie Jouet^1^, Julie Badin, Brice Fermier, Marc Feller, Mathieu Serie

###### ^1^41, C.H. de Blois, Blois, France

####### **Correspondence:** Emilie Jouet - emjouet@hotmail.com

*Annals of Intensive Care* 2017, **7**(**Suppl 1**):S76

**Introduction** The healthcare activity is recognized as a major polluting activity. In France, it generates 800,000 tons of waste cremated each year, and represents 12% of the tertiary energy consumptions. In the United States, it generates 7000 tons of waste per day and 7% of total CO_2_ emissions in 2007 were attributed to him. Ultimately, such waste production is associated with adverse environmental and health effects. Nevertheless, near half of the hospital waste would be recyclable, particularly in our intensive care units (ICU) [1]. Furthermore, sustainable development solutions generate profits.

The aim of this study is to make an overview of waste produced in a ICU and offer solutions to conserve natural resources and reduce the carbon footprint bound to the healthcare activity.

**Materials and methods** Experimental study, single-center, concerning a period of 6 months in an ICU—high surveillance unit compound of 16 beds. We have identified all waste generated. Our packaging were given to the recycling company in connection with the hospital. Then we have studied the impact of the implementation of sustainable development solutions.

**Results** Firstly, we have studied the non-recycled waste and the quantity produced over a period of 1 month. Approximately 8 kg of waste is produced per patient per day with 45% of infectious waste and 55% of general waste. These results were linked with a bad distribution of garbage bags in the rooms (130 L of infectious waste versus 50 L of general waste).

Secondly, we have improved our way to sort and consume and we have created recycling dies without compromising patient safety. All these measures have not increased workload.

Changing bags in the rooms (20 L of infectious waste and 2 bags of 50 L of general waste) allowed to reach the normal goals of 2 sectors with a net benefit estimated at 4500 euros per year.

The medical broken glass containing drugs was thrown into plastic containers of 5 L for infectious waste to prevent the risk of cuts. By creating a specific die intended to the general waste, we could quantify the production of this glass to 10 kg per week and to spare the use and the incineration of 350 containers of 5 L per year (global economy of 1000 euros).

Plastic packaging represented an important proportion of the cremated waste. We have created 3 sectors of recycling including the polypropylene (80–200 kg per month), the polyethylene colorless and colored polyethylene. This plastic is sold to be recycled without additional cost for the hospital.

The linerboards was cremated. We have created a recycling die (50 kg per month). This sector was subsequently extended to the entire hospital structure, particularly the pharmacy that produces 12 containers of 400 L per month. They are now sold without additional cost.

Many unnecessary plastic waste is generated daily. We have removed using mild soap plastic bottles of 30 ml by using the same mild soap in pump of 500 ml (economy of 1000 euros). The use of 100 L plastic bags for the transitional deposit of linen has been deleted (economy of 450 euros).

Concerning the paper: 100% of the impressions were made in simplex. Printers were parametrized on both sides by default allowing the economy of 60 reams per year (30,000 sheets), several thousand liters of water and the reduction of CO_2_ emissions.

**Discussion** Recycling is only one component of the sustainable development in health. Other avenues that could be considered to improve ICU sustainability would include examining water use (for linen), electricity use (reducing non-essential use at night…). Beyond these actions, we need to encourage our suppliers to turn to sustainable and recyclable packages to reduce the use of polluting and depletable fossil fuels such as oil. But also to develop with them circular economies where waste is returned to them to be reused.

**Conclusion** We must ask the question also resuscitate our tons of waste. Our ICU produce large quantities of waste (over 2 tons per year per bed). However, a significant proportion, especially plastic, is recyclable with a significant environmental and financial benefit. Waste management also requires an optimal and rational use of supplies because “the best waste is that which is not produced” and that excess is not a guarantee of quality.

As already said St Exupéry in 1939: “We do not inherit the Earth from our parents, we borrow it from our children.” So do not expect tomorrow to reduce major adverse ecological impact paradoxically generated by a great profession whose ultimate goal is to cure people.

**Competing interests** None.

**Reference**

1. McGain F, Story D, Hendel S. An audit of intensive care unit recyclable waste. Anesthésia. 2009;64:1299–302.

#### S77 Point of care ultrasonography: is there a place for pocket size ultrasonography devices?

##### Gabriel Preda^1^, Vincent Dubée^1^, Naïke Bigé^1^, Jeremie Joffre^2^, Jean-Luc Baudel^1^, Hafid Ait-Oufella^1^, Eric Maury^1^

###### ^1^Réanimation médicale, Hôpital Saint-Antoine, Paris, France; ^2^ Service de reanimation médicale, Hôpital Saint-Antoine, Paris, France

####### **Correspondence:** Eric Maury - ejhmaury@gmail.com

*Annals of Intensive Care* 2017, **7**(**Suppl 1**):S77

**Introduction** Point of care ultrasonography (US) is more are more used in Intensive Care Unit. It should be performed to answer questions with a dichotomous response (yes or no or I don’t know). Whereas hand held US (HHUS) devices are well fitted to this practice, the place of new available pocket size US (PSUS) devices has still to be delineated. The aim of the present study was to compare quality of portable and pocket size US devices and to assess the agreement between the two modalities.

**Patients and methods** During a 8-month period every cardiac, pulmonary or abdominal point of care US exam deemed to be performed between 08.30 am and 07.00 PM with a HHUS device was eligible. Vascular exam were excluded. Every US exam was performed twice, with our regular HHUS device (MTurbo^®^, Sonosite, Bothwell, MA, USA) and with a PSUS (Vscan^®^; GEHealthcare, Wauwatosa, WI, USA) equipped with a phased-array probe 1—5 MHz and 1.7—3.8 MHz respectively. HHUS and PSUS were performed at random by intensivists skilled in general ultrasonography and mastering at least a basic level of echocardiography. Exam duration as soon as US device was working, imaging quality assessed using a semi quantitative scale (0 nothing is seen to 4 excellent) were recorded. Several items were assessed: left ventricular ejection fraction (normal, moderately or severely depressed), right ventricular dilation (RV < 0.6LV, RV = LV, RV > LV), IVC variation (present or not), Pleural effusion (present or not, centesis feasible), B-lines (less than 3 or coalescent), lung condensation (present or not), diphragmatic excursion ascites (present or not, centesis feasible), kidney visualization (yes or no), obstructive uropathy (present or not), intrabiliary duct dilation (present or not). Demographic and descriptive data are expressed as mean ± 1 SD or median IC25%–75%. Procedure duration ultrasonographic were compared using the Student t test. Answers to the questions were yes, no, or do not know. Agreement between the answers to clinical questions was assessed using the Kappa Cohen coefficient.

**Results** During the study period, 71 US exams were performed in 38 patients (71 years [51–82], 28 male, SAPSII:45 (30–55), height 172 cm [168–177], weight 74 kgs [65–90], 50% of them receiving mechanical ventilation). Duration of exams were shorter with (PSUS (181 ± 97 vs 225 ± 146, p = .006). Global imaging quality was better with HHUS (2.95 ± 0.87 vs 2.73 ± 0.84, p = .004) and was better for cardiac, abdominal and lung exams even difference did not reach significancy. Simultaneous visualisation of both ventricles on an apical view was more frequently impaired with PSUS (27 vs 4.5%, p = 0.04). Among the 690 items assessed by both US devices, inability to answer to a question was more frequently observed with PSUS than HHUS (6.7 vs 4.2%, p = 0.05).

Agreement between the two devices for some of the items assessed are depicted in the Table 23.

**Table 23:** Agreement between HHUS and PSUS for some of the items tested

Question	Case	Discrepant results	K coefficient
Pleural effusion (38)	19	5	0.71 [0.56–0.87]
B-lines (38)	29	5	0.69 [0.55–0.83]
Lung consolidation (38)	12	3	0.71 [0.52–0.90)
Obstructive uropathy (25)	3	0	1
LVEF (23)	7	3	0.7 [0.51–0.89]
VD (23)	1	3	0.61 [0.42–0.79]

**Conclusion** Despite a weaker imaging quality and greater unconclusive exam rate, this study suggests that pocket size US device is an acceptable alternative to hand held US device for basic point of care ultrasonography.

**Competing interests** None.

#### S78 Six-months outcome of patients aged 80 and over following admission to an intensive care unit

##### Jérôme Pillot^1^, William Marie^1^, Chloé Gisbert-Mora^1^, Camille Vinclair^1^, Pierre Lesbordes^1^, Pascal Mathieu^1^, Fabienne De Brabant^1^, Emmanuel Muller^1^, Marie-Aline Robaux^1^

###### ^1^Réanimation polyvalente - usc, Centre Hospitalier de la Côte Basque, Bayonne, France

####### **Correspondence:** Jérôme Pillot - jeromewilliam.pillot@gmail.com

*Annals of Intensive Care* 2017, **7**(**Suppl 1**):S78

**Introduction** There is a growing interest in long-term outcomes of patients aged 80 and over. Survival rate ranges from 20 to 30% 1 to 2 years after intensive care unit (ICU) admission according to different publications. A better knowledge of patient’s short-term prognosis and the inherent risks of ICU care impeding recovery would provide intensivists with management guidelines for ICU admissions and procedures for clinical practices. Physicians could adapt medical care to outcomes which could be expected rather than limiting to age. This study aims to analyse 6-month mortality in patients aged 80 and over following admission to an ICU, to investigate withholding/withdrawing (Wh/Wd) life sustaining treatment decisions and to identify predictors of 6-month mortality.

**Patients and methods** We conducted a retrospective, observational, cohort study including all elderly patients (≥80 years) admitted from 1st January 2012 to 31st December 2013 in a medical-surgical ICU in a French district general hospital.

**Results** 109 patients (13%) who were at least 80 years old were selected for the study out of the 814 patients admitted to the ICU during the study. 76 (70%) had medical conditions and 29 (27%) required unscheduled surgery. The mean SAPS II score was 57 ± 21 and the mean APACHE II score was 19 ± 8. During the ICU stay, 85 (78%) required mechanical ventilation, 74 (68%) catecholamines, 32 (30%) blood transfusions, 22 (20%) needed additional renal support and 15 (14%) neuromuscular blockade. More than 80% needed antibiotics.

Six-month mortality was 58% (63/109), with no significative difference between age, sex or reasons for admission. Two-thirds (41/63) of deaths occurred in ICU with a mean length of ICU stay of 9 ± 9 days. ICU-mortality of our study population was significatively higher than ICU-mortality of the 705 younger patients (<80 years) admitted during the same period (38 vs 22%, OR 2.1, 95% CI [1.4–3.2], p = 0.001). This was particularly significant for male mortality (41 vs 20%, OR 2.8, 95% CI [1.7–4.8], p < 0.0001), but not female (32 vs 26%, p = 0.453). Two-thirds of patients were alive 6-month after their ICU visit, although 19% were to die during their hospital stay. Less than 6% of patients followed up with a specific geriatric care after discharge the ICU.

The SAPS II score, the APACHE II score, severe sepsis and Wh/Wd decisions were independently associated with 6-month mortality (SAPS II ≥ 60, OR 3, CI95% [1.1–8.5], p = 0.034; APACHE II ≥19, OR 3, CI95% [1.1–8], p = 0.029; severe sepsis, OR 6.2, CI95% [1.1–33], p = 0.034; Wh/Wd decisions, OR 14.7, CI95% [4.5–47.8], p < 0.0001). 75% (31/41) of patients died in ICU were identified for Wh/Wd treatment, this represented 63% of 6-month deaths. More than 88% (40/45) of patients who had Wh/Wd decisions during ICU stay died within 6 months. In contrast, 89% of survivors had no Wh/Wd treatment during their ICU stay.

**Conclusion** This study confirms that physicians should not reject ICU admission simply on the basis of 80 years old or over. Initial clinical severity measured by usual prognostic scores, severe sepsis, as well as withholding/withdrawing decisions made during the ICU stay, are independent risk factors of 6-month mortality. Six-month survival in these patients is far from negligible. Future research on patient’s prognosis after an ICU stay should clearly concentrate on the existence of a comprehensive geriatric system of care and on Wh/Wd treatment decisions. Special attention should be paid to the Wh/Wd decision-making process so as to ensure that patient’s death is not the result of a self-fulfilling prophecy.

**Competing interests** None.

#### S79 Intensive Care Unit (ICU) non-readmission decisions: a survey of practices

##### Mikhael Giabicani^1^, Antoine Marchalot^1^, Stéphanie Gelinotte^1^, Pierre Louis Declercq^1^, Jean-Pierre Eraldi^1^, François Bougerol^1^, Nicolas Meunier-Beillard^2^, Hervé Devilliers^3^, Jean-Pierre Quenot^4^, Jean-Philippe Rigaud^1^

###### ^1^Intensive care, Dieppe General Hospital, Dieppe, France; ^2^Umr 7366 cnrs, University of Burgundy, Dijon, France; ^3^Internal medicine, Chu Dijon, Dijon, France; ^4^ Intensive care, Chu Dijon, Dijon, France

####### **Correspondence:** Mikhael Giabicani - mikhael.giabicani@gmail.com

*Annals of Intensive Care* 2017, **7**(**Suppl 1**):S79

**Introduction** In-ICU admission of critically ill patients depends partly on benefits expected. Then, some patients are not admitted in ICU because of a high risk of mortality and/or a poor expected quality of life after ICU hospitalization. While ICU readmissions increase morbidity and mortality, decisions of “non-readmissions” are taken for patients who had underwent very difficult ICU hospitalizations. But «non-admission» and «non-readmission» decisions lead to limit initiation of life-sustaining therapy for (potential) life-threatening patients. Then, these are decisions to forgo life-sustaining treatment as defined by the French law enacted in April 2005 (the so-called Leonetti’s law). The aim of this study is to evaluate practices concerning ICU «non-readmission» decisions.

**Materials and methods** We performed a survey among French intensive care physicians by electronic questionnaire between December 2015 and January 2016 to evaluate «non-readmission» practices. The questionnaire consisted of 27 items relating to various aspects of the decision process. We also recorded responders’ socio-demographic data (age, sex, length of practice, type of ICU in which they practiced). Qualitative variables were expressed in percentages and were compared according socio-demographic data using the Chi-2 test or Fisher exact test.

**Results** Among 653 physicians contacted, 165 (25.3%) completed the questionnaire. Responders were mostly male (sex ratio 3:1), aged between 35 and 50 years and were experienced for years in intensive care. «Non-readmission» decisions are mainly taken at the end of ICU hospitalization (87%) following a collegial discussion (89%). However, patients, relatives and/or family, external consultant or general practitioner are marginally involved in the decision process (respectively 10, 34, 14 and 29% cases). If 73% of the relatives are informed of the «non-readmission» decision, less than a third of patients receive this information. Once decision made, the latter is notified in the patient’s medical record in 93% cases. However, 96% of responders consider having the right not into account this decision as appropriate. In addition, 92% of doctors consider clearly a difference between readmission’s request for a new acute episode and the same request for a worsening of a chronic disease. Otherwise, palliative care program is decided jointly to the «non-readmission» decision in only 41% of cases. Finally, 91% of responders consider the «non-readmission» decisions as fully parts of the law of April 2005. Secondary analyzes have not identified any difference according to socio-demographic groups.

**Conclusion** Evaluation of «non-readmission» decisions shows a defective process regarding French legal and ethical aspects. Particularly, patients themselves are insufficiently involved in discussion and/or informed of decisions about their own health. Moreover, an external consultant is rarely applied and palliative cares are insufficiently developed after «non-readmission» decisions. For providing corrective measures, this study lead to propose a «non-readmission» process by integrating the discussion for a real «patient’s care project» at the end of the ICU hospitalization. This process would lead to collect patient’s opinion through advance directives, to ensure a collegial discussion including an external consultant and to allow reevaluation of global patient’s clinical status and one or more organ failure(s). Then, «non-readmission» decisions would be integrated in a therapeutic project which would promote the initiation of a palliative care program if necessary. The purpose of this process is well to respect patient’s autonomy and dignity as required by French law and medical ethics.

**Competing interests** None.

#### S80 Withholding decision in elderly patients: who share the decision?

##### Sidonie Hubert,^1^, Camille Verrière,^1^, Marc Tran,^2^, Kelly Tiercelet^2^, Mélanie Cherin^3^, Cédric Bruel^2^, Francois Philippart^2^

###### ^1^Médecine interne, Groupe Hospitalier Paris Saint-Joseph, Paris, France; ^2^Réanimation, Groupe Hospitalier Paris Saint-Joseph, Paris, France; ^3^Réanimation polyvalente adulte, Centre Hospitalier Intercommunal André Grégoire, Montreuil, France

####### **Correspondence:** Francois Philippart - fphilippart@gmail.com

*Annals of Intensive Care* 2017, **7**(**Suppl 1**):S80

**Introduction** The proportion of elderly patients is steadily increasing. Due to the growth of this part of the population who suffer from multiple pathologies, the need for hospitalization in intensive care increases. According to the simulations, the proportion of octogenarian patients in ICU will increase reaching the third of ICU patients. While chronological age is not a significant factor of poor prognosis in the ICU (1), many factors should be taken into account to evaluate the relevance of ICU admission in the senior population and withholding such intensification should be consensually discussed between clinicians and obviously as often as possible with the patient himself (2).

The aim of the study was to assess the role of stakeholders (ward physicians, intensivists, family doctor and patient himself) in the decision of withholding ICU admission for elderly patients in our internal medicine department.

**Patients and methods** We made a prospective observational monocentric study, including all the elderly patients (defined as older than 75) admitted in the internal medicine department from January 2012 to June 2012. The only non-inclusion criterion was patient’s refusal to participate to the survey.

Collected data involve physiological (cognitive, autonomy, nutritional status), morbidities (acute and chronic diseases) and social parameters (marital status, relatives). And evaluation of quality of life by the patient himself using an analog visual scale was also obtained.

Internal medicine physicians were asked to report any ICU withholds decision for their patients. In absence of notification, every physician was questioned again the day of the concerned patient’s discharge.

**Results** One hundred ninety-one patients were included between January and June 2012. The average age of the study population was 85.7 ± 6.01 years. A decision of therapeutic intensity limitations was made for 96 patients (50.2%). Lack of decision concerning potential ICU transfer was noted for 7 patients (3.6% of cases).

In 60.4% (58 of 96 patients) of cases withholding ICU decision was taken by the attending physician, alone. Family physicians was involved in limitation decision in only 77 (40.3%) of cases. During the period of the study, ICU physician were involved in only 3 decisions about potential intensification of therapeutic interventions in patients. Concerning patient for whom a therapeutic limitation was taken, 46% did not want to take an active part in decision affecting their potential intensity of care. On the other hand, patient’s opinion was not sought in 30.5% of cases.

Factors associated with an ICU withholding decision were the presence of advanced cognitive impairment (26% of limitation decisions), dependency (Katz scale ≤4; 22.9%), high comorbidities and age (86.9 vs 82.8 years; p = 0.002).

**Discussion** The absence information regarding refusal decision actually made by intensivist can be considered as a weakness of the study. Nevertheless, systematic prospective information of limitation decisions before potential ICU proposal of critical patients show the weak involvement of intensivist about intensification decisions in our hospital.

**Conclusion** ICU withholding is relatively frequent in internal medicine department, notably due to the importance of elderly and comorbid admitted patients. Despite medical societies and law recommendations, lonesome limitation decision remains surprisingly frequent. Patients’ participation in this reflection is made difficult both because their desire not to be involved and due to the lack of offered opportunities by clinicians.

**Competing interests** None.

**References**

1. Montuclard L, Garrouste-Orgeas M, Timsit JF, Misset B, De Jonghe B, Carlet J. Outcome, functional autonomy, and quality of life of elderly patients with a long-term intensive care unit stay. Crit Care Med. 2000;28(10):3389–95.
2. Philippart F, Garrouste-Orgeas M. Le refus d’admission en réanimation, première limitation des thérapeutiques actives. In: Enjeux ethiques en réanimation. Berlin: Springler; 2010.

#### S81 ICU nurses’ perception of end-of-life decision making: a French multicenter survey

##### Fanny Ardisson^1^, Nancy Kentish-Barnes^2^, Gwenaëlle Jacq,^3^, Christine Lebert^4^, Akli Chermak^5^, Alexandre Lautrette^6^, Matthieu Legrand^7^, Frédéric Pène^8^, Nicolas de Prost^9^, François Vincent^10^, Amélie Seguin^11^, Alexis Soummer^12^, Vincent Peigne^13^, Guillaume Thiery^14^, Alice Cottereau^15^, Julien Mayaux^16^, Elie Azoulay^17^, Emmanuel Canet^17^

###### ^1^Hôpital Saint-Louis (AP-HP), Paris, France; ^2^Réanimation médicale, Assistance Publique Hôpitaux de Paris, Hôpital Saint Louis, Paris, France; ^3^Réanimation, C.H. de Versailles, Le Chesnay, France; ^4^Réanimation, Centre Hospitalier Départemental - site de La Roche-sur-Yon, La Roche-sur-Yon, France; ^5^Réanimation polyvalente, CH sud Essonne, Paris, France; ^6^Réanimation médicale, CHU Gabriel-Montpied, Clermont-Ferrand, France; ^7^Anesthésie réanimation et traitement chirurgical des grands brûlés, APHP - Hôpital Saint-Louis, Lariboisière, Fernand-Widal, Paris, France; ^8^Réanimation Médicale, Hôpital Cochin, Paris, France; ^9^Réanimation Médicale, Hôpital Henri Mondor, Créteil, France; ^10^Réanimation polyvalente, Groupe Hospitalier Intercommunal Le Raincy-Montfermeil, Montfermeil, France; ^11^Réanimation médicale, Centre Hospitalier Universitaire de Caen, Caen, France; ^12^Réanimation, Hospital Foch, Suresnes, France; ^13^Réanimation, Hôpital d’Instruction des Armées Percy, Clamart, France; ^14^Réanimation - Grands Brulés, CHU Pointe à Pitre - Abymes, POINTE A PITRE, France; ^15^ Réanimation polyvalente adulte, Centre Hospitalier Intercommunal André Grégoire, Montreuil, France; ^16^ Réanimation médicale, Hôpital Pitié-Salpêtrière, Paris, France; ^17^ Réanimation médicale, Hôpital Saint-Louis, Paris, France

####### **Correspondence:** fanny.ardisson@gmail.com (Fanny Ardisson)

*Annals of Intensive Care* 2017, **7**(**Suppl 1**):S81

**Introduction** The French law and recent expert opinions have emphasized the need for a multidisciplinary approach in decisions to forgo life sustaining therapies for the critically ill. We sought to assess how ICU nurses actually rank their involvement and perceive this process.

**Materials and methods** We conducted a cross sectional survey using a web-based questionnaire between June and September 2016.

**Results** Of the 38 ICUs invited to participate, 22 (58%) agreed. A total of 541 ICU participants completed the survey of whom 73% were nurses and 26% assistant nurses. Median age was 31 (Inter Quartile Range 27–38) years and 84% were female. Median work experience was 7 (4–12) years and time in the ICU was 5 (3–8) years. Eighty-five percent of the participants have been involved at least once in a multidisciplinary end-of-life discussion. Less than half of the participants reported a good (4%) or partial (44%) knowledge of the current end-of-life legal framework.

The decision to start a discussion about Withdraw Life-Sustaining Therapy (WLST) was initiated by a senior intensivist in 83% of the cases, by a nurse in 5% and an assistant nurse in 0.6%. This decision was approved by 97% of the participants. The decision-making process was considered to be initiated at the right time for 49% of the participants, too late for 48%, and too early for 3%. The discussion occurred mostly in the afternoon (38%) or during the medical staff (22%), in a dedicated place in 63% of the cases. A median of 7 (5–10) health-care professionals attended the WLST discussion.

Half the respondents reported being reluctant to talk during the discussions and 11% never expressed their own opinion. Indeed, although the length of the discussion was 30 (15–45) minutes, participants estimated to talk during only 3 (1–5) minutes. The following reasons were mentioned by the participants to explain these facts: having cared for the patient for too short time (62%), lack of medical knowledge (35%), decision of WLST already taken by the medical staff (31%), their opinion not really taken into account (19%), reluctant to talk during meetings in general (18%), consider that the discussion is limited to a medical expertise (15%), limited professional experience (14%), and fear to express a different opinion (12%).

Nevertheless, 84% of the participants were partially (76%) or totally (8%) satisfied by the way the decision making process was conducted, 81% considered that collegiality was applied, and 93% agreed with the final decisions.

**Conclusion** ICU nurses rank favorably multidisciplinary WLST discussions. Nevertheless their involvement in the discussion remains limited. Beyond factors related to work organization and professional experience, efforts should be made to recognize their role and value, and to encourage them to share their own opinions with the other members of the ICU team.

**Competing interests** None.

#### S82 Determinants and prognosis of elevation of high-sensitivity cardiac troponin T in patients hospitalized with vasodilatatory shock

##### Marie Caujolle^1^, Jérôme Allyn^1^, Dorothée Valance^1^, Caroline Brulliard^1^, Olivier Martinet^1^, Julien Jabot^1^, Thomas Gallas^1^, David Vandroux^1^, Nicolas Allou^1^

###### ^1^Réanimation polyvalente, CHU La Réunion, Saint-Denis, France

####### **Correspondence:** Marie Caujolle - caujollemarie@hotmail.com

*Annals of Intensive Care* 2017, **7**(**Suppl 1**):S82

**Introduction** The aim of this study was to assess determinants and the prognostic value of high-sensitivity cardiac Troponin T (hsTnT) elevation in intensive care unit (ICU) patients with vasodilatatory shock (VS).

**Materials and methods** A prospective observational cohort study conducted in an ICU. Patients hospitalized for VS were consecutively included between October 2014 and December 2015. This observational study was approved by the Ethics Committee of Félix Guyon University Hospital (R14008). Clinical, laboratory, electrocardiography and echocardiography data were collected within 24 h of admission. Established criteria for the diagnosis of VS were as follows: (1) vasopressors required despite adequate fluid loading to achieve a mean blood pressure >65 mmHg; (2) absence of elevated left-ventricular filling pressures at transthoracic echocardiography; (3) signs of impaired organ perfusion and (4) absence of positive inotropes.

Values of hs-cTnT with exponential distribution were log10 transformed for linear analyses. Univariate (t-test and Pearson correlation test) and multivariate analysis were used to examine correlations of variables with log10(hs − cTnT). Predictive factors of in-hospital mortality in bivariate analysis with P < 0.05 were entered into a multivariate logistic regression analysis using backward selection with P < 0.05. Results are expressed as median [25th–75th percentiles], mean ± standard deviation or number (%) as appropriate.

**Results** During the study period 206 patients were hospitalized in ICU for VS (71.8% with septic shock) with a mean simplified acute physiology score 2 of 58 ± 17. Among them 185 (89.8%) had hsTnT > 14 pg/mL and the median peak of hsTnT was 55.1 [24.5–136] pg/mL.

Combining all variables following multivariate analysis, high body mass index (t = 2.52, P = 0.01), low left ventricular systolic function (t = −2.73, p = 0.007), high white blood cell count (t = 3.72, P = 0.0001), low creatinine clearance (t = −2.84, P = 0.0005), high lactate level (t = 2.62, P = 0.01) and ST-segment depression (t = 3.98, P = 0.0001) best correlated with elevated log10 transformed hsTnT concentration. Left ventricular diastolic dysfunction defined by the presence of a lateral E′ maximal velocity <10 cm/s did not significantly correlate with log10 (hsTnT) (T = 1.41, P = 0.16).

Seventy-seven patients (37.4%) died in-hospital. Following univariate analysis peak of hsTnT was higher in non-survivors (67.4 [32.5–167] pgmL) than in survivors (50.1 [20.9–121.3], P = 0.027). After multivariate analysis, peak of hsTnT was not associated with a significant reduction of in hospital mortality (aOR 0.99 [95% CI 0.93–1.02]). Factors associated with a significant reduction of in hospital mortality were higher age (P = 0.002), higher lactate level (P = 0.004), chronic obstructive pulmonary disease (P = 0.04), diabetes mellitus (P = 0.03), immunodepression (P = 0.05) and respiratory failure (P = 0.02).

**Conclusion** In patients hospitalized for VS high body mass index, low left ventricular systolic function, high white blood cell count, low creatinine clearance, high lactate level and ST-segment depression are the variables correlating significantly with high-sensitivity troponin-T concentrations. Peak of hsTnT was not significantly associated with in-hospital mortality in this setting.

**Competing interests** None.

#### S83 Free plasmatic mitochondrial DNA-receptor for advanced glycation end-products: a new signaling pathway of critical illness-induced endothelial dysfunction

##### Arthur Durand^1^, Rémi Nevière^2^, Florian Delguste^2^, Eric Boulanger,^2^, Raphaël Favory^3^, Sebastien Preau^4^

###### ^1^Nord, Hôpital Roger Salengro, Lille, France; ^2^Inserm u995 equipe 4, Université Lille 2, Lille, France; ^3^Pôle de réanimation, hôpital salengro, Centre Hospitalier Régional Universitaire de Lille, Lille, France; ^4^Réanimation médicale, Centre Hospitalier Régional Universitaire de Lille, Lille, France

####### **Correspondence:** Arthur Durand - durand.arthur@gmail.com

*Annals of Intensive Care* 2017, **7**(**Suppl 1**):S83

**Introduction** Mitochondria are evolutionary endosymbionts that are derived from ancestral aerobic bacteria and so might bear and release bacterial molecular motifs supporting the role of mitochondria in danger signal regulations. Free circulating mitochondrial DNA (mtDNA) is elevated in a wild range of critical illness observed in intensive care units, and is associated with bad outcomes and mortality. The mtDNA is a molecular pattern that belongs to mitochondrial damage associated molecular patterns (mtDAMPs), and can interact with pattern recognition receptors (PRR) to induce self defense reaction. Free mtDNA activates inflammatory signaling pathways through Toll-like endosomal receptor 9 (TLR9) interactions. Nevertheless, new evidence advocates a role of the receptor for advanced glycation end-products (RAGE) in mtDNA signaling. Experimental data suggest a role of mtDNA-PRR interaction in systemic inflammation and organ dysfunctions as septic acute kidney injury or pulmonary inflammation. Impact of free circulating mtDNA on endothelial cell is not known.

The main purpose of this study was to test whether mtDAMPs and mtDNA can induce endothelial dysfunction. We also evaluated the role of mtDNA-RAGE axis in mtDAMPs induced endothelial dysfunction.

**Materials and methods** Mitochondria were isolated from livers of wild type C57B6 mice. Isolated mitochondria were sonicated on ice to obtain mtDAMP preparations. Semi quantitative evaluation of mtDAMP content was tested by qPCR, with specific markers of mtDNA (cytochrome B (cytb), NADPH oxidase (ND4)). Intraperitoneal injection of 1 mg of mtDAMPs was used as experimental model in wild type and RAGE KO mice, as previously described [1]. The mtDAMPs were also administrated after ex vivo DNAse preparation. Endothelial function was assessed with a Mulvany-Halpern style myograph, 4 h after mtDAMP administrations on aorta (conductive vessel) and on 2d division of mesenteric artery (resistive vessel). Endothelial-dependent relaxation was studied by cumulative expositions of the vessels to acetylcholine (1.10-10–1.10-5 M). Endothelial-independent relaxation was studied by sodium nitroprussiate exposition.

**Results** The mtDAMPs preparation contains a high quantity of mtDNA with a 1/cycle threshold (Ct) ratio of 0.062 for cytb expression. Intraperitoneal administrations of mtDAMPs induced a decrease of endothelial-dependent relaxation mainly on conductive vessel (p = 0.0079, n = 5 per group) and to a lesser extent on resistive vessel (p = 0.22, n = 5 per group). RAGE-KO mice were protected from mtDAMPs-induced aorta dysfunction (p = 0.53, n = 5 per group). The ex vivo exposition of mtDAMPs to a DNAse preparation decreased mtDNA content in mtDAMPs solution with a 1/Ct ratio of 0.038 for cytb expression. Eventually, the pretreatment of mtDAMPs with a DNAse preparation prevented the mtDAMPs-induced aorta dysfunction (p = 0.85, n = 5).

**Discussion** More than prognostic markers, mtDAMPs particularly mtDNA seems implicated in endothelial dysfunction in critically ill patient. New evidence suggest RAGE interaction in endosomal TLR9 pro-inflammatory and pro-oxidant response to mtDNA [2]. Also in sepsis, physiological clearance of circulating DNA might be impaired, this results comfort the possibility of therapeutic regulation of free circulating mtDNA to prevent septic organ dysfunction related to mtDAMPs accumulations.

**Conclusion** Exogenous mtDAMPs can induce endothelial dysfunction in mice. The mtDNA-RAGE axis is a key component of the signaling pathway involved in this dysfunction.

**Competing interests** None.

**References**

1. Zhang Q, Raoof M, Chen Y, Sumi Y, Sursal T, Junger W, et al. Circulating mitochondrial DAMPs cause inflammatory responses to injury. Nature. 2010;464:104–7.
2. Sirois CM, Jin T, Miller AL, Bertheloot D, Nakamura H, Horvath GL, et al. RAGE is a nucleic acid receptor that promotes inflammatory responses to DNA. J. Exp. Med. 2013;210:2447–63.

#### S84 Prediction of fluid responsiveness using carbon dioxide gap

##### Najla Tilouch^1^, Oussama Jaoued^1^, Ben Sik Ali Habiba^1^, Houda Mater^1^, Rim Gharbi^1^, Mohamed Fekih Hassen^1^, Souheil Elatrous^1^

###### ^1^Réanimation Médicale, EPS Taher Sfar Mahdia, Mahdia, Tunisia

####### **Correspondence:** Mohamed Fekih Hassen - mohamed.fekihhassen@rns.tn

*Annals of Intensive Care* 2017, **7**(**Suppl 1**):S84

**Introduction** The use of dynamic parameters to assess fluid responsiveness was supported by cyclic changes in stroke volume induced by mechanical ventilation. However, these parameters have several limits. Venous to arterial carbon dioxide difference inversely related to cardiac index. Consequently, fluid administration would be beneficial if carbon dioxide gap increases.

**Objective** To investigate whether carbon dioxide gap predicts fluid responsiveness in patients with acute circulatory failure.

**Patients and methods** We conducted a prospective study in the medical intensive care unit of Hospital Taher Sfar at Mahdia, between March 2013 and April 2016. Patients with circulatory failure and who required mechanical ventilation were included. We measured the variation of cardiac index between baseline and after volume expansion of 500 ml of saline fluid. The PiCCO2 was used to measure cardiac index. Response to fluid challenge was defined as a 15% increase in cardiac index. Before and after fluid administration, we recorded carbon dioxide difference and hemodynamic parameters.

**Results** Among 68 included patients, 33 (49%) were responders. The causes of acute circulatory failure were septic shock (*n* = 45), cardiogenic shock (*n* = 11), and Hypovolemia (*n* = 12). Carbone dioxide gap was significantly higher in responders group (8 ± 7 vs 4 ± 4 mmHg, p = 0.019). The area under the ROC curve for carbon dioxide gap was 0. 68 (95% CI 0.55–0.80). The best cutoff value was 6 mmHg (Sensibility = 46%, specificity = 80%, positive predictive value = 52% and negative predictive value = 60%). The area under the ROC curve for delta carbon dioxide was 0.54 (95% CI 0.4–0.68).

**Conclusion** In this study, baseline carbon dioxide gap was not universal indicator to predict the fluid responsiveness in patient with circulatory failure.

**Competing interests** None.

#### S85 Incidence and prognostic impact of new onset supraventricular arrhythmia in patient with septic shock

##### Walid Sellami^1^, Zied Hajjej^1^, Soumaya Ben Yedder^1^, Walid Samoud^1^, Iheb Labbene^1^, Mustapha Ferjani^1^

###### ^1^Department of critical care medicine and anesthesiology, Military Hospital of Tunis, Tunisia, Tunis, Tunisia

####### **Correspondence:** Walid Sellami - drsellamiwalid@yahoo.fr

*Annals of Intensive Care* 2017, **7**(**Suppl 1**):S85

**Introduction** Supraventricular arrhythmia (SVA) is commun in intensive care unit (ICU). Its incidence seems to be higher in patients with sepstic shock. Sepsis-associated myocardial dysfunction promote the occurrence of SVA by constituting an arrythmogenic substrate or under the effect of inotropic drugs.

The aim of this study is to assess the incidence and prognostic impact of SVA in patients with septic shock.

**Patients and methods** We retrospectively studied all patients with new onset SVA suffering from septic shock in non cardiac surgical ICU. Myocardial dysfunction was evaluated by transthoracic echography (TTE) after an adequate cardiac resuscitation using intravenous fluids expansion and adjunctive vasoactive agents. SVA was detected by the electrocardiogram scope. During the study period clinical and biologic characteristics, hemodynamic tolerance (vasopressors doses, arterial pressure changes), current treatment (such as corticoid), duration of mechanical ventilation, duration of vasopressor requirement and hospital mortality were collected.

**Results** Sixty patients were included in the study. The SVA occurred in 28 patients, with an incidence of 46%. The median time to onset was 2 days. Cardioversion was performed for 15 patients with an effectiveness of 80%. Clinical and biological characteristics were similar between the groups with and without SVA: SAPS 2 and SOFA score at the beginning of septic shock, the existence of ARDS and cardiac biomarkers (NT-proBNP, troponin). However, renal failure and the use of corticoid in septic shock were more frequent in the group with SVA. The maximum doses of vasopressor agent were not significantly different between the groups with or without SVA. Myocardial dysfunction in sepsis defined by the left ventricle ejection fraction (LVEF) less than 45% (or the need for inotropic drug for LVEF > 45%) was not associated with the occurrence of SVA (+SVA group: n = 8; −SVA group: n = 6; p: 0.57). SVA was poorly-tolerated, observed by a significant decrease in mean arterial pressure and a significant increase in norepinephrine doses within 1 h of the start of SVA. The occurrence of SVA was associated with longer duration of use of vasopressor agent and a longer duration stay in ICU (+SVA group: 21 days, −SVA group: 14 days; p = 0.03). There was no difference in duration of mechanical ventilation and hospital mortality between the two groups.

**Conclusion** The occurrence of SVA is common in septic shock, poorly tolerated hemodynamically and associated with longer duration stay in the ICU and vasopressor need. Sepsis myocardial dysfunction isn’t necessarily associated to the occurrence of SVA.

**Competing interests** None.

#### S86 Long term hemodynamic effects of prone position in ARDS patients

##### Ruste Martin^1^, Laurent Bitker^1^, Hodane Yonis^1^, Mylène Aublanc,^1^, Aurore Louf-Durier,^1^, Floriane Lissonde^1^, Sophie Perinel-Ragey^1^, Claude Guérin^1^, Jean-Christophe Richard^1^

###### ^1^Réanimation médicale, Hôpital de la Croix-Rousse, Lyon, France

####### **Correspondence:** Jean-Christophe Richard - j-christophe.richard@chu-lyon.fr

*Annals of Intensive Care* 2017, **7**(**Suppl 1**):S86

**Introduction** A short term beneficial effect of prone position on cardiac index has been shown in 50% of 18 ARDS patients, and was related to an increase in cardiac preload in preload responsive patients (1). The aim of this study was to evaluate the long term hemodynamic response to prone position in a larger series of ARDS patients.

**Patients and methods** Single center retrospective observational study performed on ARDS patients hospitalized in a medical ICU between July 2012 and March 2016. Patients included were adults fulfilling the Berlin definition for ARDS, undergoing at least one prone position session, under hemodynamic monitoring by the Picco^®^ device, with availability of hemodynamic measurements performed before (T1), at the end (T2), and after the prone position session (T3). Prone position sessions were excluded if they were performed >7 days after ARDS onset. The following variables were recorded: demographic, SAPSII, ARDS severity and risk factor, SOFA score and cumulative fluid balance at PP onset, delay between ARDS session and PP session, hemodynamic, arterial blood gas, ventilatory settings, plateau pressure, catecholamine dose and additional treatments. Statistical analyses were performed using prone position session as statistical unit and mixed models taking into account both multiple prone position sessions by patient and multiple measurements during a prone position session. p < 0.05 was chosen for statistical significance. Data are expressed as mean ± standard deviation.

**Results** 85 patients fulfilled the inclusion criteria over the study period, totalizing 149 prone position sessions (2 ± 1 sessions per patient). Patients’ age was 65 ± 11 y, 67% were male, 75% fulfilled the criteria for severe ARDS, and SAPSII at ICU admission was 64 ± 17. ARDS risk factors were pneumonia in 63 (74%), aspiration pneumonia in 26 (31%), and sepsis in 7 (8%) patients. Duration of prone position sessions was 16 ± 3 h. Hemodynamic measurements were performed in PP 13 ± 3 h after PP session onset. At session onset, SOFA score was 15 ± 4, and cumulated fluid balance was 2.5 ± 7.1 L. Vasopressor were used in 87%, inhaled nitric oxide in 22%, and neuromuscular blocking agents in 91% of the sessions. Hemodynamic and respiratory parameters before, during and after the prone position sessions are reported in Table 24.

**Table 24:** Hemodynamic and respiratory measurements at T1, T2 and T3

	Before PP (T1)	End of PP (T2)	After PP (T3)
Cardiac index (L min^−^1 m^−2^)	3.4 ± 1.2	3.4 ± 1.0	3.2 ± 1.0^†,‡^
Extravascular lung water (mL kg^−1^ PBW)	13.9 ± 4.7	13.5 ± 4.5	13.1 ± 4.0^†^
Pulmonary vascular permeability index	2.6 ± 1.1	2.3 ± 0.8^†^	2.4 ± 0.8^†^
Global end-diastolic volume (mL m^−2^)	729 ± 204	757 ± 203^†,^*	723 ± 208
dPmax (mmHg s^−1^)	1207 ± 496	1177 ± 478	1132 ± 458
Global ejection fraction (%)	21 ± 7	21 ± 7	20 ± 7
Cardiac function index (min^−1^)	4.9 ± 1.5	4.7 ± 1.5	4.6 ± 1.6^†^
Vasopressor dose (µg kg^−1^ min^−1^)	0.94 ± 1.53	0.90 ± 1.82^†^	0.97 ± 1.94^†^
Dobutamine dose (µg kg^−1^ min^−1^)	3 ± 5	3 ± 6	3 ± 6
Lactates (mmol L^−1^)	3.7 ± 3.3	3.3 ± 2.9^†^	3.2 ± 2.9^†^
PEEP (cmH_2_O)	9 ± 3	8 ± 2^†^	9 ± 3^†^
Plateau pressure (cmH_2_O)	22 ± 4	21 ± 4^†^	21 ± 4^†^
VT (ml kg^−1^ PBW)	6 ± 1	6 ± 1	6 ± 1
PaO_2_/FiO_2_ (mmHg)	110 ± 25	176 ± 58^†,^*	150 ± 56^†^

^†^p < 0.05 versus before PP; ^‡^ p < 0.05 versus end of PP; * p < 0.05 versus after PP

Cardiac index increased by at least 15%, decreased by at least 15% or remained stable in 35 (24%), 36 (34%), and 78 (52%) of the sessions, respectively. As compared to both other groups, PP sessions with significant increase in cardiac index had the following significant differences at T1 by univariate analysis: lower cardiac index, lower global end-diastolic volume, lower cardiac function index, and lower vasopressor dose. Multivariate analysis is under investigation.

**Conclusion** Prone position is associated with an increase in global end-diastolic volume, reversible after return in supine position that may explain the positive effect of PP on cardiac index observed in ¼ of the PP sessions.

**Competing interests** None.

**Reference**

1. Jozwiak M. Beneficial hemodynamic effects of prone positioning in patients with acute respiratory distress syndrome. Am J Respir Crit Care Med 2013;188:1428–33.

#### S87 Is the shape of the inferior vena cava a good tool to assess fluid responsiveness? A pilot study

##### Hélène Cochet^1^, Jean Pierre Ponthus^1^, Virginie Amilien^1^, Martial Tchir^1^, Elise Barsam^1^, Mohsen Ayoub^1^, Jean Francois Georger^1^

###### ^1^Réanimation polyvalente - surveillance continue, Ctre Hospitalier Intercommunal de Villeneuve Saint Georges, Lucie et Raymond AUBRAC, Villeneuve-Saint-Georges, France

####### **Correspondence:** Hélène Cochet - herrpotok@gmail.com

*Annals of Intensive Care* 2017, **7**(**Suppl 1**):S87

**Introduction** Make sure that our patient have a good circulatory condition is a daily challenge for the intensivist. One of the therapeutics is fluid and one of his purpose is to increase venous return and then cardiac output. In order to examine that, There are several tools as the transthoracic echocardiogram wich allows the visualisation and the study of the respiratory variability from the inferior vena cava (IVC). Unfortunately there are some situations where the IVC visualisation is difficult (obesity, gut surgery, emphysema). The IVC is easily seen by a transhepatic ultrasound in her retrohepatic section. We make the hypothesis that the shape of the IVC could be predictive of fluid responsiveness.

**Patients and methods** We have performed fluid challenge in patients under mechanical ventilation. The need for fluid therapy is the intensivist in charge decision. We performed a echocardiogram and we take two measures of the ICV: major axis and minor axis, the ICV is measured avec the sus hepatic vena. A elastometry index (EI) is determined which is the ratio of minor axis to minor axis. The fluid challenge is 250 ml of isotonic saline then we perform a new echocardiogram. A Tag is written on the patient to take the same ultrasound slice. We retain one increase of 15% of the cardiac index (IC) as a success of the filling. We exclude the presenting patients A right cardiac insufficiency, an arrhythmia and/or a HTAP. The statistical analysis is realized with the software R.

**Fig. 21:**
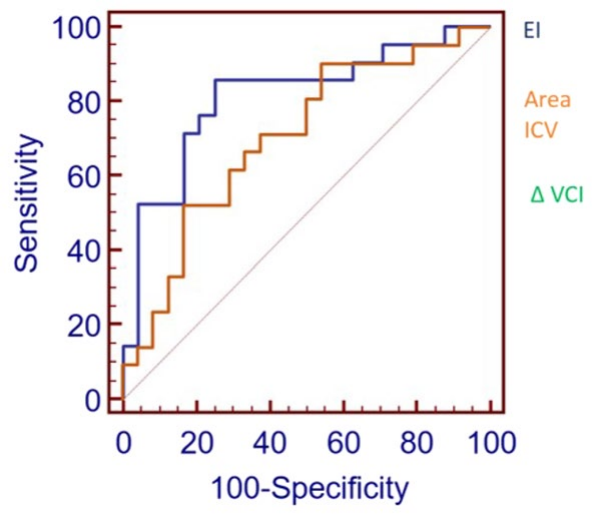
See text for description

**Results** Between August, 2015 and January, 2016 we included 49 patients. The average age is of 59 years (24–81), IGS2 of 48 (20–84), Ejectionnal Fraction of 48% 35–75) and the S wave tricuspid is 14 (9–21). The causes of the filling were an oliguria (31%), a low blood pressure (20%), a low cardiac output (30%), a hyperlactatémia (9%) and an other cause in 10% of the cases. We find a positive correlation between the EI and the increase of the IC, also for the area of the VCI and the respiratory variations of the VCI (p0.05) the other variables are not predictive (BP, E/e’, E/A). The data are summarized in the picture 1. ROC curves has been established (Table 25; Fig. 21).

**Table 25:** Relevant parameters

	R	IC	Value
EI	0.64	0.38–0.77	0.83 (0.69–093)
ICV Area	0.21	0.15–0.89	12 (9–17)
ICV respiratory variation (%)	0.61	0.34–0.85	12 (8–17)

	ACS (IC95)	Se/Sp	Threshold
*Courbes ROC*			
EI	0.81 (0.67–0.91)	85.7–75	0.85
ICV Area	0.70 (0.55–0.83	90–45	16.37
ICV respiratory variation			
(%)	0.79 (0.65–0.87)	80–77	16

**Conclusion** It is an alternative and simple technique once acquired. The limits are essentially established by technical conditions such as the necessity of being very perpendicular at the risk of falsifying the EI. For now it is just a static index but an ovoïd vena cava (EI: 0.85) may be predicative of fluid responsiveness. The strangely big size of the VCI can be attributed to a deformation of the in-depth image because of the probe but this should not modify in theory the report of EI. It is a question in the future of testing this parameter at the spontaneous breathing patient, study his variability with respiration and strengthening its external validity.

**Competing interests** None.

#### S88 Quality of reporting of fluid responsiveness evaluation studies: a five year systematic review

##### Izaute Guillame^1^, Matthias Jacquet-Lagrèze^1^, Jean-Luc Fellahi^1^

###### ^1^Anesthésie réanimation, Hopital Louis Pradel, Bron, France

####### **Correspondence:** Matthias Jacquet-Lagrèze - matthias.jl@gmail.com

*Annals of Intensive Care* 2017, **7**(**Suppl 1**):S88

**Introduction** The Standards for the Reporting of Diagnostic Accuracy Studies (STARD) statement was developed in 2003 to improve the quality of reporting of diagnosis studies (1). STARD is a list of 25 essential items, it was updated in 2015 (2). Fluid responsiveness evaluation is a cornerstone of hemodynamic management of patient with circulatory failure. Numerous studies have evaluated tools for assessing fluid responsiveness. The purpose of this work was to evaluate the quality of reporting of studies assessing fluid responsiveness, using the STARD criteria.

**Materials and methods** We used MEDLINE via PubMed to search all publication of studies assessing the ability to predict or diagnose fluid responsiveness in a perioperative or circulatory failure context between the 1st of January 2004 and the 1st of January 2014. We have presented herein the last 5 years (2010–2014). Paediatric studies, publication with no abstract available or in another language than English, review, meta-analysis, and journals with impact factor less than 2 were excluded. We have checked these studies for all the 25 STARD criteria. The rating methodology of each item was discussed and double checked (GI and MJL). The 2003 and 2015 STARD quality score (SQS) was the integer of the present item. Univariate and multivariate analysis were conducted to find out if some characteristic of the studies were linked with a better SQS. Following items were studied: year of publication, positive or negative study, location of study, expert team, funding source, conflict of interest, the use of a new device, instruction to the author including STARD statement, impact factor of the journal. Every covariate associated with a *p*-value lower than 0.20 in the univariate analysis were used in the multivariate analysis. Statistical analysis was performed with R software. All the test were two-sided, *p* < 0.05 was considered statistically significant.

**Results** After a double screening (GI, MJL) of 430 articles (2010–2014), 97 were selected, then 46 were excluded because of an IF < 2. Fifty-one studies were selected and reviewed. The mean 2003 overall quality score was 11.85 on a scale of 0–25, whereas the mean 2015 overall quality score was 15.34 on a scale of 0–30. The 2003 SQS remained stable between 2010 and 2014 (*p* = 0.173). Correlation between 2003 and 2015 SQS was good (r = 0.83; *p* < 0.001). Some items are insufficiently reported, especially: participant sampling (adequately reported in 19.6% of studies), executing and reading the tests (11.7%), blindness (11.8%) and, the description of test reproducibility (19.1%). Some items are better reported: technical specifications and/or references to tests (65.1%), methods for calculating or comparing (90.2%), clinical and demographic characteristics (82.3%), diagnostic accuracy (90.2%). Only 16% of the journals studied required authors to use STARD. A high Impact factor and the year of the study were the items associated with a better SQS the presence of a conflict of interest was associated with a lower SQS in univariate analysis. A higher impact factor (> 5), was the only independent factors statistically significantly (*p* = 0.037) associated with higher SQS in a multivariate regression model.

**Discussion** Our study showed that the SQS were very low. Assessment of a study depends on quality of reporting. Blindness and participant sampling are the cornerstone to evaluate such bias as spectrum, verification, review and selection bias of a study, and were unfortunately scarcely reported compared to existing data in diagnosis accuracy reporting. One of the limitation is the 5 years sample of the study. We have planned to continue the analysis for a 10-year review starting just after the 2003 STARD publication.

**Conclusion** Our study showed that several items remain poorly reported. We recommend systematic use of STARD criteria in the elaboration and reporting of future studies that evaluates the preload dependence.

**Competing interests** None.

**References**

1. Bossuyt PM. STARD 2015: an updated list of essential items for reporting diagnostic accuracy studies. BMJ. 2015;351:h5527.
2. Bossuyt PM. Towards complete and accurate reporting of studies of diagnostic accuracy: The STARD initiative. Ann Intern Med. 2003;138(1):40–4.

#### S89 Neurological impairment in cirrhotic patients admitted to ICU: hepatic versus drug-induced encephalopathy

##### Julie Assaraf^1^, Simona Tripon^2^, Maxime Mallet^3^, Marika Rudler^4^, Julien Mayaux^5^, Dominique Thabut^6^, Nicolas Weiss^7^

###### ^1^Unité de soins intensifs d’hépatogastroentérologie, Pitié-Salpêtrière Hospital, Paris, France; ^2^Unité de soins intensifs d’hépatogastroentérologie, Hôpital Pitié-Salpêtrière, Paris, France; ^3^Unité de Soins Intensifs. Service d’Hépato-gastro-entérologie du Pr Poynard, CHU Pitié Salpétrière, Paris, France; ^4^Hepatology and gastroenterology, Pitié-Salpêtrière Hospital, Paris, France; ^5^Réanimation médicale, Hôpital Pitié-Salpêtrière, Paris, France; ^6^Brain Liver Pitié-Salpêtrière Study Group (BLIPS), Hôpital Pitié-Salpêtrière, Paris, France; ^7^Unité de réanimation neurologique, Hôpital Pitié-Salpêtrière, Paris, France

####### **Correspondence:** Julie Assaraf - julie.assaraf@gmail.com

*Annals of Intensive Care* 2017, **7**(**Suppl 1**):S89

**Introduction** Neurological impairment, i.e. encephalopathy, is commonly observed in patients with decompensated cirrhosis and/or portosystemic shunts admitted in ICU. Often ascribed to high plasmatic levels of ammonia, encephalopathy could also be induced by drugs or infection, due to altered blood–brain barrier (BBB) permeability. This latter setting is often underdiagnosed and encephalopathy related to hyperammonemia (so called hepatic encephalopathy-HE) being pointed out as the culpit of all neurological symptoms in cirrhotic patients. Quinolones and betalactamins were recently found in the cerebrospinal fluid of HE patients and it has been shown that the expression of efflux pumps, responsible for drugs passing through the BBB, was altered in animal models of HE. The purpose of this study was to assess the incidence of neurological impairment, i.e. encephalopathy, in cirrhotic patients hospitalized in ICU and to analyse its promoting factors, especially ammonia levels, infection and drug intake.

**Patients and methods** Between February 2015 and September 2016, all cirrhotic patients admitted in ICU were prospectively included. We collected patient’s clinical and biological parameters, especially ammonia levels, all the ongoing treatments, and seeked for the presence of evolutive infection defined as an infection considered as clinically relevant. Cirrhotic patients having encephalopathy and ammonia levels above 75 μmol/L were considered as having HE. We performed univariate and multivariate analysis to estimate the association between ammonia level, infection or drugs intake and the presence of encephalopathy.

**Results** 202 cirrhotic patients were included, they were 58 ± 11 years-old, 71% were men. Etiology of cirrhosis was as followed: alcohol 110 (54%), virus 27 (13%), NASH 19 (10%), mixed 30 (15%), others 16 (8%). 122 patients (60%) displayed neurological impairment, i.e. encephalopathy at admission, 84 (42%) had ammonia levels above 75 μmol/L, 80 (40%) had evolutive infection and the following drugs were found: furosemide, hydrochlorothiazide, betablockers, lactulose, rifaximine, betalactamins, proton pump inhibitors (PPIs), quinolones, benzodiazepines, morphine derivatives, insulin, anti-epileptic drugs, corticosteroids and immunosuppressants. In univariate analysis, patients with encephalopathy had significantly higher Child and MELD scores, higher ammonia, bilirubin and leukocytes levels, more evolutive infections, and received more frequently PPIs, lactulose and quinolones as compared to those who had no encephalopathy. Patients with encephalopathy took also more frequently an association of agonist of efflux pumps (e.g. PPIs, betalactamins or quinolones) (p = 0.0005). In the group of patients displaying low ammonia levels (less than 75 μmol/L), encephalopathy was significantly increased in patients having infection (p < 0.0001), taking quinolones (p = 0.0086), PPIs (p = 0.0196), and betalactamins (p = 0.0438) whereas ursodeoxycholic acid treatment had a protective effect (p = 0.0299). Multivariate analysis showed that high ammonia levels (p < 0.0001), presence of infection (p < 0.0001), and taking quinolones (p = 0.0403) were independently associated with the presence of encephalopathy.

**Conclusion** These results highlight the involvement of drugs in the physiopathology of neurological impairment in cirrhotic patients. Several drugs that were implicated can modify the activity of efflux pumps expressed on the BBB. Besides HE, cirrhotic patients are susceptible to develop drugs-induced encephalopathy regardless of ammonia level, underlying the necessity to carefully assess every treatment indication, given their potential neurotoxicity.

**Competing interests** None.

#### S90 Functional outcome of prolonged hypoglycemic encephalopathy

##### Guilaume Barbara^1^, Bruno Megarbane^2^, Laurent Argaud^3^, Guillaume Louis^4^, Nicolas Lerolle^5^, Francis Schneider^6^, Stéphane Gaudry^7^, Nicolas Barbarot^8^, Angéline Jamet,^9^, Hervé Outin^10^, Sébastien Gibot^1^, Pierre-Edouard Bollaert^1^

###### ^1^Réanimation médicale, hôpital central, C.H.U. de Nancy, Nancy, France; ^2^Service de Réanimation Médicale et Toxicologique, CHU Lariboisière, Paris, France; ^3^Réanimation Médicale, Hospices Civils de Lyon - Groupement Hospitalier Edouard Herriot, Lyon, France; ^4^Réanimation polyvalente, Hôpital de Mercy, Ars Laquenexy, France; ^5^Réanimation médicale, Centre Hospitalier Universitaire d’Angers, Angers, France; ^6^Réanimation médicale, C.H.R.U. Hôpitaux Universitaires Strasbourg, Strasbourg, France; ^7^Service de réanimation médico-chirurgicale, CHU Louis Mourier, Colombes, Colombes, France; ^8^Service de réanimation polyvalente, Centre hospitalier, Saint Brieuc, France; ^9^Réanimation médicale, CHU de Poitiers, Poitiers, France; ^10^Service de Réanimation Médico-Chirurgicale, CHI de Poissy/Saint-Germain-en-Laye - Site Poissy, Poissy, France

####### **Correspondence:** Pierre-Edouard Bollaert - pe.bollaert@chu-nancy.fr

*Annals of Intensive Care* 2017, **7**(**Suppl 1**):S90

**Introduction** Little is known about the causes, clinical course and long-term outcome of prolonged hypoglycemic encephalopathy. We investigated functional long-term prognosis and identify prognosis factors of patients admitted in an Intensive Care Unit (ICU) with prolonged neurological manifestations related to obvious hypoglycemia.

**Patients and methods** This was a retrospective study conducted in patients hospitalized from July 1, 2004, to July 1, 2014, in 9 polyvalent intensive care units in French university and general hospitals. Participants were adults admitted to the ICU with a Glasgow Coma Score <8 due to hypoglycemia <0.5 g/l and persistent consciousness disorders after normalizing blood glucose levels. Patients with possible other causes of consciousness disorders, previous cognitive disorders, hypothermia <35 °C or circulatory arrest within 24 h after ICU admission, were excluded. Follow-up phone call was used to determine the functional outcome using modified Rankin Scale at 1-year minimum (mRS) with mRS 0–3 defining good and mRS 4–6 poor outcomes.

**Results** Forty-nine patients were included. Median age was 55 years. Causes of hypoglycemia were various, mainly including insulin or oral antidiabetic drugs abuse (65%) and neuroendocrine carcinoma (16%). Twenty (41%) patients died in the ICU, and a total of thirty-one (63%) patients had a poor outcome at 1-year follow-up. Twenty-two (45%) patients underwent therapeutic limitation, primarily due to no hope expected for improvement (86%). Among survivors with poor outcome at ICU discharge (n = 15), one died 10 months later and six improved their outcome at 1-year follow-up with a median decrease in mRS of −2 [IQR −2.5 to −1.75], including five patients who finally reached the good outcome group. Among the patients with good outcome on ICU discharge, one died, seven further improved their outcome at 1 year with a median decrease in mRS of −2 [IQR −2.0 to −1.0], whereas one patient deteriorated while remaining in the good outcome group (from mRS 1–2). On multivariate analysis, only low mRS prior to ICU admission (OR 2.6; 95% CI = 1.1–6.3; *P* = .03) and normal brain imaging (OR 7.1; 95% CI 1.1–44; *P* = .03) were significantly predictive of outcome. All patients (n = 15) who stayed hypoglycemic 480 min or more evolved poorly.

**Conclusion** Poor outcome was observed in about 60% of this population of prolonged hypoglycemic encephalopathy. Some patients can recover satisfactorily over time though we did not identify any robust factor of good outcome.

**Competing interests** None.

**Reference**

1. Witsch J, Neugebauer H, Flechsenhar J, Jüttler E. Hypoglycemic encephalopathy: a case series and literature review on outcome determination. J Neurol. 2012;259(10):2172–81.

#### S91 Cerebrospinal fluid characteristics during status epilepticus: a descriptive and prognostic study

##### Mathilde Holleville^1^, Stéphane Legriel^1^, Anne Laure Chateauneuf^1^, Sébastien Cavelot^1^, Gwenaëlle Jacq,^1^, Jean-Denis Moyer^1^, Jean Pierre Bedos^1^, Fabrice Bruneel^1^

###### ^1^Réanimation médico-chirurgicale, C.H. de Versailles, Le Chesnay, France

####### **Correspondence:** Mathilde Holleville - mathildeholleville@gmail.com

*Annals of Intensive Care* 2017, **7**(**Suppl 1**):S91

**Introduction** The lumbar puncture is an important tool of the etiologic investigations during status epilepticus. Scarce data are available regarding cerebrospinal fluid pleocytosis directly induced by seizures. We aimed to describe the cerebrospinal fluid characteristics of the patients admitted to the intensive care unit for status epilepticus, and to report the clinical, biological and radiological data of these patients. Furthermore, to determine whether status epilepticus *per se* increases the cerebrospinal fluid pleocytosis, we specifically studied cerebrospinal fluid parameters after status epilepticus in adult patients after exclusion of the cases of status epilepticus induced by acute infection or neoplastic disease of the central nervous system.

**Patients and methods** This monocentric retrospective study (2005–2012) included patients of more than 18 years old admitted to the intensive care unit for status epilepticus and who had a lumbar puncture. Status epilepticus was defined by a single seizure lasting more than 5 min or multiple seizures without neurological recovery in between. A cerebrospinal fluid pleocytosis was defined by a cerebrospinal fluid cell count of more than 4/mm^3^. We analyzed demographic, medical, biological data and outcomes at 3 months and 1 year. The groups were compared using univariate and multivariate analysis. Cerebrospinal fluid pleocytosis was considered directly linked to seizures when etiologic investigations identified causes of status epilepticus which were not known to induce cerebrospinal fluid pleocytosis.

**Results** Among 191 patients admitted to the intensive care unit for status epilepticus, 124 (65%) had a lumbar puncture and were included. Eight of them had a cerebrospinal fluid pleocytosis of infectious or neoplastic origin. Among the 116 remaining patients, 97 (84% = group B2) had normal cerebrospinal fluid parameters, and 19 (16% = group B1) had a cerebrospinal fluid pleocytosis considered as directly linked to seizures, with a median [Q1–Q3] cerebrospinal fluid cell count of 8/mm3 [7–20]. No other cerebrospinal fluid parameters changes were observed in the two groups, unless a slight increase in the cerebrospinal fluid lactate (mmol/l), but without significant difference between the two groups (3.1 [2.7–3.4] group B1 vs 2.9 [2.5–3.5] group B2). In the multivariate analysis, only myoclonic status epilepticus (OR 55.1; CI 95% 6.02–1103.88; p = 0.001) and leukocytosis at the admission (OR 1.1 per 103/mm3 increment; CI95% 1.01–1.21; p = 0.01) were associated to pleocytosis directly linked to status epilepticus. The intensive care unit mortality was significantly increased in patients with pleocytosis directly linked to status epilepticus (5 (26%) vs 7 (7%), p = 0.02), but hospital mortality was not significantly different between the 2 groups.

**Discussion** Overall, we reported a higher rate of lumbar puncture than those reporting in others studies concerning status epilepticus. Furthermore the rate of 16% of pleocytosis directly linked to status epilepticus is slightly higher than in most studies. Unfortunately we didn’t realize a second lumbar puncture to assess the pleocytosis normalization during the days following the first lumbar puncture. The pathophysiological hypothesis of this phenomenon may be that prolonged/repeated seizures during status epilepticus would induce a blood–brain barrier dysfunction thereby favoring a cerebrospinal pleocytosis.

**Conclusion** In our study, 16% of status epilepticus without infectious or neoplastic origin had a cerebrospinal pleocytosis directly linked to status epilepticus. This pleocytosis was significantly associated with myoclonic seizures and blood leukocytosis. These data may help to interpretation of cerebrospinal fluid pleocytosis during status epilepticus.

**Competing interests** None.

**References**

1. Chatzikonstantinou A, Ebert AD, Hennerici MG. Cerebrospinal fluid findings after epileptic seizures. Epileptic Disord. 2015;17(4):453–9.
2. Tumani H, Jobs C, Brettschneider J, Hoppner AC, Kerling F, Fauser S. Effect of epileptic seizures on the cerebrospinal fluid—a systematic retrospective analysis. Epilepsy Res. 2015;114:23–31.

#### S92 Synek score and NSE to predict poor neurological outcome after cerebral anoxia and therapeutic hypothermia

##### Dimitri Titeca Beauport^1^, Magalie Joris^1^, Philippe Merle^2^, Loay Kontar^1^, Antoine Riviere^1^, Bertand De Cagny^1^, Thierry Soupison^1^, Michel Slama^1^, Julien Maizel^1^

###### ^1^Réanimation médicale, Centre Hospitalier Universitaire, Amiens, France; ^2^Explorations fonctionelles du système nerveux adulte, Centre Hospitalier Universitaire, Amiens, France

####### **Correspondence:** Dimitri Titeca Beauport - titeca.dimitri@chu-amiens.fr

*Annals of Intensive Care* 2017, **7**(**Suppl 1**):S92

**Introduction** Neurological prognostication from cardiac arrest survivor is a current concern. EEG patterns and NSE dosage are two important prognostic factors. NSE threshold for prediction of poor outcome appear controversial, in part, because of variability in dosage timing and measurement techniques. Synek Score is routinely used in our center to classify comatose patients in post cardiac arrest. The aim of this study was to assess the prognostic value of NSE and Synek classification to predict poor neurological outcome.

**Materials and methods** We conducted a retrospective monocentric study in our medical intensive care unit between November 01, 2013 and August 31, 2016. All patient having at least one EEG and H48-72 NSE dosage available were included. Patients dead from post cardiac arrest shock, stroke, moribund, spontaneous awaking, or missing data were excluded. Electroencephalograms were prospectively classified by electrophysiologist according to the Synek score and NSE were performed within H48-72 post cardiac arrest.

**Results** Among 162 cardiac arrest survivors, 70 were retrospectively analyzed. All patients received sedation with midazolam and sufentanil and mild therapeutic hypothermia for 24 h. Forty-eight were classified as poor outcome (CPC ≥ 3) and 22 as good outcome (CPC 1–2). Serum NSE levels above 46 µg/l was associated with a poor neurological outcome with sensitivity of 75%, specificity of 100% and AUROC at 0.95. The Synek score ≥3 was also strongly associated with poor neurological outcome with sensitivity of 91.5%, specificity of 90.9% and AUROC at 0.95.

**Conclusion** In the limits of this study, Synek score ≥3 on first EEG and H48-72 NSE > 46 µg/l are two strong predictor of poor neurologic outcome. The prognostic value of the association of the NSE and the Synek score to predict a poor neurological outcome remain to be determined.

**Competing interests** None.

#### S93 Influence of PbtO2 with severe TBI patients outcome at 6 months

##### Aurelie Laine^1^, De Sa Natalie^2^

###### ^1^Nord, C.H. Régional Universitaire de Lille (CHRU de Lille), Lille, France; ^2^Nord, C.H. de Valenciennes, Valenciennes, France

####### **Correspondence:** Aurelie Laine - aurelie-laine@hotmail.fr

*Annals of Intensive Care* 2017, **7**(**Suppl 1**):S93

**Introduction** Traumatic Brain Injury (TBI) is a major public health problem. It is the leading cause of death and disability in young subjects. One of the principles of the TBI management is prevention of secondary cerebral insults including maintaining perfusion and cerebral oxygenation, control of intracranial pressure (ICP). An increase in ICP above 20 mmHg is associated with poor outcome. Cerebral hypoxia can occur with normal level of ICP and cerebral perfusion pressure (CPP).Monitoring of regional partial pressure of brain tissue oxygen (PbtO2) is a safe and reliable method for measuring cerebral oxygenation.

**Patients and methods** A retrospective single-center observational study was conducted between January 2012 and December 2013, aimed to study the influence of PbtO2 with severe TBI patients outcome at 6 months through Glasgow Outcome Scale (GOS). The hourly values of ICP, PbtO2 and CPP were recovered on daily monitoring sheets. We compared two groups according to their GOS. During the study period, 66 patients underwent a monitoring ICP and PbtO2.

**Results** The mean age was 33.7 ± 14.1 years. 78.8% were men. The initial Glasgow score was 6.6 ± 3.7. The mean Simplified Acute Physiology Score (SAPS II) was 43.4 ± 11.2 and Injury Severity Score (ISS) 30.5 ± 9.7. At 6 months, 7 patients had died (GOS1). Forty patients had a good outcome: GOS 4–5 (Group 1). Sixteen patients had poor outcome: GOS 2–3 (Group 2). In Group 2, there are significantly more PbtO2 hourly values below 10 mmHg at Day0 (5.5 ± 6.4 vs 2.3 ± 3, 2 in Group 1, p = 0.025); and more PbtO2 hourly values greater than 20 mmHg at Day0 (8.4 ± 7.0 vs 4.8 ± 4.9, p = 0.045).

**Conclusion** PbtO2 less than 10 mmHg or greater than 20 mmHg at Day0 is associated with poor outcome at 6 months in the severe TBI. The PbtO2 allows a more individual approach of monitored TBI.

**Competing interests** None.

**References**

1. Brain Trauma Foundation, American Association of Neurological Surgeons, Congress of Neurological Surgeons, Joint Section on Neurotrauma and Critical Care, AANS/CNS, Bratton SL, Chestnut RM, et al. Guidelines for the management of severe traumatic brain injury. VI. Indications for intracranial pressure monitoring. J Neurotrauma. 2007;24(Suppl 1):S37–44.
2. Stiefel MF, Spiotta A, Gracias VH, Garuffe AM, Guillamondegui O, Maloney-Wilensky E, et al. Reduced mortality rate in patients with severe traumatic brain injury treated with brain tissue oxygen monitoring. J Neurosurg. 2005;103(5):805–11.

#### S95 Organ procurement and kidney transplantation under Maastricht 3 condition (M3): update on 1 year of activity

##### Mathieu Cornuault^1^, Jérome Libot^1^, Jean Reignier^2^, Karim Asehnoune^3^, Bertrand Rozec^4^, Jacques Dantal^5^, Michel Videcoq^1^

###### ^1^Coordination prélèvements organes, CHU Hôtel-Dieu Nantes, Nantes, France; ^2^Réanimation, Centre Hospitalier Départemental - site de La Roche-sur-Yon, La Roche-sur-Yon, France; ^3^Réanimation chirurgicale, C.H.U. Hôtel Dieu, Nantes, France; ^4^Réanimation CTCV Transplantation thoracique, CHU de Nantes - Hôpital Nord Laennec, Saint-Herblain, France; ^5^Néphrologie-transplantation rénale, CHU Hôtel-Dieu Nantes, Nantes, France

####### **Correspondence:** Mathieu Cornuault - mathieu.cornuault@chu-nantes.fr

*Annals of Intensive Care* 2017, **7**(**Suppl 1**):S95

**Introduction** Organ donation in patients after a decision to withdraw life-supportive therapies (WLST) (Maastricht 3 condition: M3) have been performed in our hospital since May 2015. We report here main characteristics of donors, data on M3 procedure and results on renal transplant recipients.

**Patients and methods** All potential donors were included in a survey from May 2015 to June 2016, according to the French national M3 protocol defined by the French Organ Procurement Agency (Agence de la Biomédecine:ABM) [1].The demographical, clinical and biological characteristics of the donors, the different deadlines and times of the protocol and data of renal transplantation were collected and analyzed.

**Results** 28 patients had inclusion criteria. Patients were admitted in intensive care unit for cardiac arrest (68%), strokes (14%), traumatic brain injury (14%), ARDS (4%). Of them, 15 procedures (54%) were stopped (6 refusals of organ donation, 4 medical contra-indications discovered with additional exams, 1 failure of vessel cannulation, 4 deaths more than 3 h after extubation). 28 kidneys were harvested and 26 transplantations performed (1 renal cancer discovered during procurement surgery).The characteristics of the donors, deadlines of the protocol and transplant recipients are reported in the Table 26.

**Table 26:** Characteristics of the donors, deadlines of the protocol and transplant recipients

*Characteristics of potential donors (n* *=* *28)*	
Age in years, mean [range]	47.9 [21–59]
Sexe, male (%)	24 (86%)
Serum creatinine, mean (μmol/L) [range]	68.96 [31–154]
CrCl by Cockroft-Gault, mean (ml/mn) [range]	139.27 [53–255]
Length of stay in ICU, mean (day) [range]	7.85 [2–23]
*Deadlines of the protocol (n* *=* *13)*	
Time from extubation to death, mean (min) [range]	37.2 [7–160]
Warm ischemia time, mean ‘min) [range]	36.8 [21–51]
Duration of vessel cannulation, mean (min) [range]	27.2 [12–45]
Duration of normothermic regional perfusion, mean (min) [range]	171.2 [94–222]
Duration of cold storage, mean (min)	591
*Characteristics of transplant recipients (n* *=* *26)*	
Age in years, mean [range]	56.4 [35–71]
Sexe, male, n (%)	16 (61.5%)
Length of stay in transplant unit, mean (day) [range]	12.8 [6–41]
Duration before creatinine <250 μmol/L after transplantation, mean (day) [range]	7.8 [1–34]
Serum creatinine (μmol/L) at the hospital discharge, mean [range]	157.5 [52–276]
Delayed Graft Function, n (%)	4 (15.3%)

**Conclusion** The French programm Maastricht 3 offered a new possibility of organ donation in our hospital. Thanks to these donors, the number of renal grafts increases and the preliminary results on transplant recipients are encouraging in line with the preliminary report of the ABM. Nevertheless, it is necessary to follow the transplant recipients and extend the procedure to new centres.

**Competing interests** None.

**Reference**

1. Conditions à respecter pour réaliser des prélèvements d’organes sur les donneurs décédés après arrêt circulatoire de la catégorie III de Maastricht dans un établissement de santé. Agence de la biomédecine. Version n°6 mai 2016 [consulté le 26/08/2016]. http://www.agence-biomedecine.fr/IMG/pdf/v6_guide_procedures_ddac_miii_052016.pdf.

#### S96 Is chronic obstructive pulmonary disease a risk factor for microaspiration in intubated critically-ill patients?

##### Thècle Degroote^1^, Emmanuelle Jaillette^1^, Farid Zerimech^2^, Balduyck Malika^2^, Saad Nseir^1^

###### ^1^Centre de réanimation, Centre Hospitalier Régional Universitaire de Lille, Lille, France; ^2^Laboratoire de biochimie et biologie moléculaire, Centre Hospitalier Régional Universitaire de Lille, Lille, France

####### **Correspondence:** Thècle Degroote - thecle.degroote@gmail.com

*Annals of Intensive Care* 2017, **7**(**Suppl 1**):S96

**Introduction** Microaspiration of gastric and oropharyngeal contaminated secretions occurs frequently in intubated critically-ill patients, and plays a major role in the pathogenesis of ventilator-associated pneumonia (VAP). At basic state, patients with chronic obstructive pulmonary disease (COPD) have an increased risk of microaspiration (due to gastro-esophageal reflux disease, pharyngo-laryngeal dysfunction…), this risk may even be more important under mechanical ventilation. The main purpose of this study is to determine if COPD is a risk factor for global abundant microaspiration (GAM) in intubated critically-ill patients.

**Materials and methods** We gathered data about two prospective multicentric randomized trials focused on microaspiration in intubated patients. Data about COPD were retrospectively collected in order to complete previous data. Microaspiration of gastric and oropharyngeal secretions was respectively determined by quantitative measurements of pepsin and salivary amylase in all tracheal aspirates during the first 48 h after intubation. GAM was defined as the presence at significant level of pepsin (>200 ng/ml) and/or salivary amylase (>1685 UI/L) in at least 30% of the tracheal aspirates. In order to find GAM independent risk factors, we realized an univariate and multivariate analysis of the variables collected.

**Results** Out of 448 patients included in the studies, 415 were analyzed among which 95 patients with COPD. 360 patients (87%) had GAM. Neither COPD diagnosis, nor spirometric severity nor specific therapeutics were associated with GAM. Risk factors for GAM in univariate analysis were the age, diabetes, low score in Glasgow Coma Scale (GCS), and no recourse to paralytic agents or vasopressors. After multivariate analysis, age was identified as independant risk factor for GAM (OR [IC 95%] = 1.03 [1.01–1.05], p < 0.001); whereas high score in GCS (OR [IC 95%] = 0.93 [0.86–0.99], p = 0.03) and use of paralytic agents (OR [IC95%] = 0.46 [0.23–0.90], p = 0.02) were associated with less occurrence in GAM. GAM was not associated with any increase of length of mechanical ventilation, length of ICU stay, VAP incidence or surmortailty.

**Discussion** In this study, we found some relevant risk factors for microaspiration (age, low score at GCS) consistent with literature on the subject. Patients with paralytic agents had less GAM which may be due to higher PEEP, higher cuff pressure and less enteral nutrition because of the severity of the underlying diseases.

**Conclusion** This study did not show any increased risk of microaspiration in intubated COPD patients, whatever stage of COPD.

**Competing interests** None.

#### S97 Microscopic examination and quantitative culture of protected brush specimen in suspected episodes of ventilator acquired pneumonia: effect of prior antimicrobial treatment

##### Jean-François Llitjos^1^, Marlène Amara^2^, Guillaume Lacave^3^, Jean Pierre Bedos^3^, Béatrice Pangon^2^

###### ^1^Réanimation Médicale, Hôpital Cochin, Paris, France; ^2^78150, Hospital Center De Versailles, Le Chesnay, France; ^3^Réanimation médico-chirurgicale, Centre Hospitalier de Versailles, Le Chesnay, France

####### **Correspondence:** Jean-François Llitjos - jllitjos@gmail.com

*Annals of Intensive Care* 2017, **7**(**Suppl 1**):S97

**Introduction** Protected specimen brush (PSB) is considered to be one of the standard methods for the diagnosis of ventilator-associated pneumonia (VAP). To our knowledge, there is no study assessing effect of prior antibiotherapy on direct examination, bacteriological culture and concordance of direct microscopy and culture.

**Patients and methods** All consecutive episodes of suspected VAP were retrospectively evaluated between January 2014 and December 2014 in a 20-bed intensive care unit. Patient’s characteristics and preexisting conditions were abstracted from the medical charts. After assessment of VAP probability using the clinical pulmonary infection score (CPIS), PSB were performed in patients with a CPIS of 6 or more. Based on antibiotic treatment in patients when bacteriological specimens were obtained, two groups were defined: no antibiotic group and antibiotic treatment started before PSB group. Two independent bacteriologists retrospectively reviewed direct examination and culture of PSB to assess bacteriological concordance, defined as non-concordant when direct examination and culture were different, concordant when direct examination and culture were similar and partially concordant when either direct examination or culture were comparable but with other microorganisms lacking in one or the other method.

**Results** During this 12-months period, among 73 mechanically ventilated patients, 116 episodes of suspected VAP with PSB were evaluated. We found 60% of PSB (n = 70) performed without antibiotic treatment and 40% of PSB (n = 46) performed under antibiotherapy. We found no significant differences in patient’s demographics, characteristics, and severity between both groups. Patients received antibiotics for the following reasons: aspiration pneumonia (n = 12), peritonitis (n = 8), VAP (n = 7), community-acquired pneumonia (n = 4), septic shock of unknown origin (n = 4), pyelonephritis (n = 3), meningitis (n = 2), acute pancreatitis (n = 2) and others (n = 4). The median duration of mechanical ventilation in the antibiotic receiving group and in the group without antibiotics was 7.5 days (IQR; 5–12 days) and 9 days (IQR: 5–22), respectively. When PSB was performed under antibiotic treatment, direct examination was positive in 26% (n = 12), culture was positive in 28% (n = 13) and those methods were concordant, non concordant and partially concordant in 93% (n = 43), 4% (n = 2) and 3% (n = 1), respectively. On the other hand, when PSB was performed without antibiotics, direct examination was positive in 65% (n = 46), culture was positive in 62% (n = 43) and those methods were concordant, non concordant and partially concordant in 71% (n = 50), 18% (n = 12) and 11% (n = 8), respectively. In univariate analysis, we found a significantly higher proportion of negative direct examination and negative culture in the antibiotic group (p > 0.001). Moreover, these methods were significantly more frequently concordant (p = 0.01), with a higher rate of both negative microscopic exam and culture when compared to the no antibiotic group (76%, n = 33 vs 38%, n = 18). Surprisingly, among the 13 patients previously treated with antibiotics with positive culture, 38% (n = 5) of the microorganisms showed antibiotics sensitivity.

**Discussion** Whether prior antibiotic treatment may induce false negative of false positive treatment is a well-recognized phenomenon, the precise effect of antibiotics on direct examination and quantitative culture is not well assessed in VAP. Moreover, despite recent development of clinico-radiological score, diagnosis of VAP remains difficult, with no gold-standard. Therefore, bacteriological guided therapy is of particular importance. We found PSB realization under antibiotic treatment is associated with a lower rate of positive direct examination and culture and suggest performing these bacteriological samples without antibiotherapy. Some authors have suggested lowering the diagnostic threshold point of this bacteriological technique in order to preserve its accuracy. However, we can postulate that microorganisms responsible of superinfection in mechanically ventilated patients treated with antibiotics may be resistant and therefore the PSB could be positive.

**Conclusion** In patients with a high pre-test probability of ventilator-acquired pneumonia, recent introduction of antibiotics significantly reduced the diagnostic accuracy of protected brush specimen by reducing rates of positive direct examination and culture. Further studies should evaluate if antibiotic discontinuation may revert this effect.

**Competing interests** None.

#### S98 Implementation and impact assessment of a “ventilator-bundle” at the university clinics of Kinshasa: before and after study

##### José Mavinga^1^, Joseph Nsiala Makunza^2^, M E Mafuta^3^, Yves Yanga^1^, Amisi Eric^1^, Jp Ilunga^1^, Ma Kilembe^1^

###### ^1^Anesthesia and Reanimation, Cliniques universitaires de kinshasa, Kinshasa, Democratic Republic of the Congo; ^2^Anesthésie-Réanimation, Hôpital Privé d’Athis-Mons - Site Caron, Athis-Mons, France; ^3^Scool of public health, Université de Kinshasa, Batiment administratif, Kinshasa, Democratic Republic of the Congo

####### **Correspondence:** José Mavinga - joicemav@yahoo.fr

*Annals of Intensive Care* 2017, **7**(**Suppl 1**):S98

**Introduction** In 2005, an international consensus conference took stock of the various measures to be implemented for the prevention of ventilator acquired pneumonia (VAP) [1]. These measures are often gathered in groups of 3 or 5 under the term of “ventilator-bundle.” The effectiveness of these “bundles” was poorly evaluated in African environment.

**Objective** To establish a VAP prevention program and assess its impact on morbidity and mortality of patients under mechanical ventilation in our service.

**Patients and methods** Prospective, mono centric, quasi-experimental before-after study. It took place in the intensive care unit of the University Clinics of Kinshasa in the Democratic Republic of Congo (DRC). This service is equipped with 8 beds and a respirator for two beds. The observational period (phase1) was carried out from February 1st to December 31st, 2014 and the intervention period (phase2) from February 15st, 2015 to February 15st, 2016. All consecutive patients intubated and mechanically ventilated for more than 48 h were included. Five preventive measures were held: hand hygiene, the elevation of the head of the bed at 30°–45°, the daily lifting of sedation, oral decontamination with chlorhexidine and control cuff pressure of the endotracheal tube. Compliance with this bundle was assessed by direct observation without the knowledge of caregivers. The diagnosis of “VAP” was held before a clinically modified sore (m CPIS) >6. The main outcomes were the incidence of VAP and mortality. The protocol for this study was approved by the Ethics Committee of the School of Public Health of the University of Kinshasa, under the approval number: ESP/EC/015/2015.

We have had non conflict of interest in this study.

**Results** We included 44 patients in the phase1 and 58 patients in the phase2. Baseline characteristics of patients were similar in both groups. Compliance with all the measures has been improved between the two period from 0 to 32.75%. The incidence density decreased from 33.74 to 18.05 VAP per 1000 ventilator days between observational and interventional period, but the all-cause mortality was almost equal in the 2 groups (88.6 vs. 86%).

**Discussion** With the implementation of our bundle, observance of the team were improved in the second group, compared to the first and the incidence density decreased from 33.74 to 18.05 VAP per 1000 ventilator days between both period. This result is consistent with the littérature. Sure enough, many studies show the same effect of VAP prevention with a decrease of nearly 50% of the incidence density of VAP, after implementation of a «ventilator –bundle [2].

**Conclusion** The implementation of a “ventilator bundle,” has significantly reduced the incidence of VAP in our service. In the contrary, our study failed to demonstrate a reduction in mortality.

**Competing interests** None.

**References**

1. American Thoracic Society, Infectious Diseases Society of America. Guidelines for the management of adults with hospital acquired, ventilator-associated, and healthcare-associated pneumonia. Am J Respir Crit Care Med. 2005;171:388.
2. Morris AC, Hay AW, Swann DG and all. Reducing ventilator-associated pneumonia in intensive care: impact of implementing a care bundle. Crit Care Med. 2011;39(10):2218–24.

#### S99 Histone deacetylases inhibition reverses sepsis-induced susceptibility to *Pseudomonas aeruginosa* pneumonia

##### Fanny Alby-Laurent^1^, Julie Toubiana^1^, Christophe Rousseau^1^, Hamid Merdji^1^, Jean-François Llitjos^1^, Jean-Paul Mira^2^, Frédéric Pène^2^, Jean-Daniel Chiche^2^

###### ^1^U1016, 22 rue méchain, 75014, paris, Institut National de la Santé et de la Recherche Médicale, Paris, France; ^2^Réanimation Médicale, Hôpital Cochin, Paris, France

####### **Correspondence:** Jean-Daniel Chiche - jean-daniel.chiche@aphp.fr

*Annals of Intensive Care* 2017, **7**(**Suppl 1**):S99

**Introduction** With an increasing incidence and high mortality rates, sepsis is a public health issue. There is growing evidence that sepsis induces long lasting alterations of transcriptional programs through epigenetic mechanisms that may lead to protracted inflammation, organ failure, sepsis-induced immune suppression (SIIS), secondary infections and death. We hypothesized that epigenetic changes contribute to the pathophysiology of SIIS. To test this hypothesis, we studied the effects of histone deacetylases (HDAC) inhibition with trichostatin A (TSA) in a double-hit murine model of SIIS and secondary pneumonia.

**Materials and methods** C57BL/6 mice were treated with TSA (2 mg/kg ip) or saline serum (CTL) 30 min before induction of sepsis by cecal ligation and puncture (CLP). Surviving mice underwent intratracheal instillation of 1.5 × 10^6^ CFU of *Pseudomonas aeruginosa* 8 days after CLP. We evaluated the effect of TSA on survival and cellular responses to the primary and secondary infections. Cellular responses in the blood, spleen and BAL were assessed by flow cytometry after CLP (Days 1, 3 & 8) and after pneumonia (4 & 12 h). We also studied lymphocyte apoptosis and dendritic cells (DC) expression of CD40, CD86, and MHCII. Bacterial clearance was assessed in the BAL and in the blood 4 and 12 h after pneumonia. Continuous variables represented as mean ± SD were compared using Student t test. Kaplan–Meier curves were compared by the log rank test. P < 0.05 indicated statistically significant differences.

**Fig. 22:**
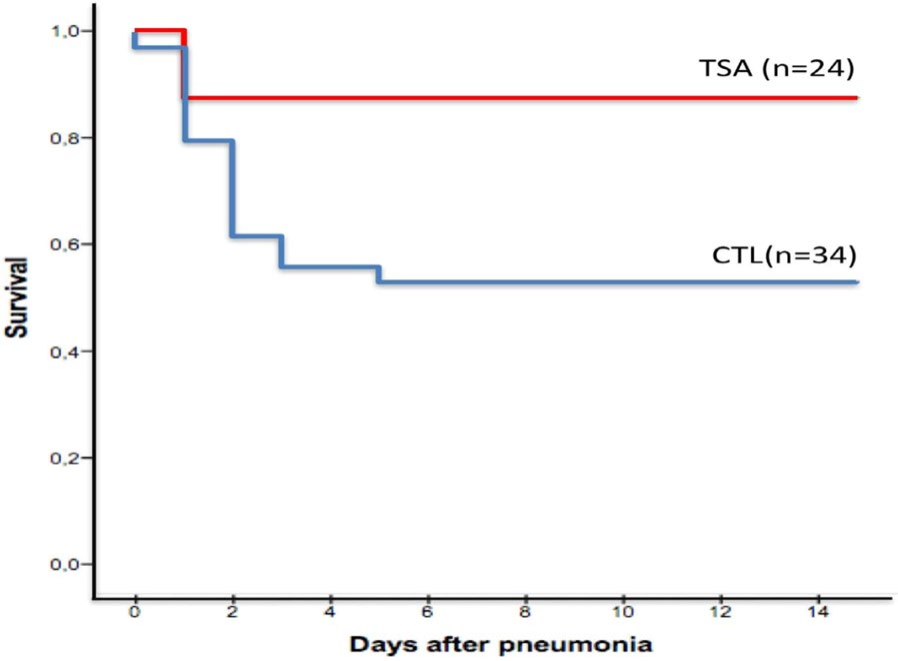
See text for description

**Results** Whereas treatment with TSA did not change survival after CLP, TSA improved survival after tracheal instillation of *P. aeruginosa* (P = 0.009, Fig. 22).

TSA-treated mice had significantly higher absolute DC, T and B-lymphocytes counts with reduced lymphocyte apoptosis after CLP. Four hours after secondary pneumonia, TSA-treated mice had significantly higher DC counts and improved bacterial clearance in the BAL, with reduced systemic dissemination of P. aeruginosa.

**Conclusion** HDAC inhibition with TSA improves survival in our murine model of secondary pneumonia, improves bacterial clearance and attenuate cellular features of SIIS. These results suggest that sepsis-induced epigenetic changes contribute to the advent of SIIS. Comprehensive characterization of epigenetic changes associated with SIIS might allow us to identify new therapeutic targets to reprogram immune cells in sepsis and avoid SIIS.

**Competing interests** None.

#### S100 Nosocomial infections in intensive burn care unit

##### Amel Mokline^1^, Achraf Laajili^1^, Helmi Amri^1^, Imene Rahmani^1^, Nidhal Mensi^1^, Lazheri Gharsallah^1^, Sofiene Tlaili^1^, Bahija Gasri^1^, Rym Hammouda^1^, Amen Allah Messadi^1^

###### ^1^Burn care department, Trauma and burn center, Tunis, Tunisia

####### **Correspondence:** Amel Mokline - dr.amelmokline@gmail.com

*Annals of Intensive Care* 2017, **7**(**Suppl 1**):S100

**Introduction** Nosocomial infections (NI) are common in burn patients due to the loss of the first line of defense against microbial invasion, immunocompromising effects of burn injury, and invasive diagnostic and therapeutic procedures. The objective of this study was to identify the incidence of nosocomial infection (NI), the pathogens and their antibacterial patterns, and prognosis of these burn patients.

**Patients and methods** A retrospective study was conducted in a 20 bed intensive burn care unit during 6 months. Patients were eligible for the study, if they met the following criteria: total burn surface area (TBSA) > 10%, length of ICU stay ≥48 h, and infected in accordance with the criteria of the National Nosocomial Infections Surveillance (NNIS) and the criteria of the SFETB [1–2]. In this study, NIs were classified into four main groups: pneumonias, bloodstream infections (BSI), catheter related infections (CRI), and urinary tract infections (UTI). For included patients, skin levy, blood cultures, urine and sputum cultures were drawn during fever or clinical features of sepsis.

**Results** During the 6-month study period, 190 patients were admitted to the ICU, 47 patients were included (24.7%). 32 were male and 15 female. The mean age was 41 ± 19 yr. The mean TBSA was 41 ± 24%. 63% were admitted from another hospital. Burn injuries were due to domestic accidents in 53% and self immolation in 25%. The mean length of ICU stay was 16 ± 14 days. 47 patients acquired 58 NIs (36.2% BSI, 20.5% pneumonia, 10.3% CRI and 10.3% UTI. There was no bacteriological documentation of NI in 22.5% of cases. NIs occured 4 days post burns. The most three isolated pathogens were: *Acinetobacter* spp. (31%), P. aeruginosa (22.5%) and extended spectrum beta-lactamase-producing Enterobacteriaceae (17%). The most frequently administered antibiotics were polymyxin/carbapenem/teicoplanin combination (21%), polymyxin/carbapenem combination (15%) and carbapenem/tigecycline combination (15%). In our study, mortality rate was 50%.

**Conclusion** Nosocomial infection occured in 24.7% of cases in burn patients, caused by *Acinetobacter* spp, *P. aeruginosa* and Enterobacteriaceae BLSE. So, eradication of infection in burn patients require effective surveillance and infection control in order to reduce mortality rates, length of hospitalization and associated costs.

**Competing interests** None.

**References**

1. National Nosocomial Infections Surveillance System. National Nosocomial Infections Surveillance (NNIS) System Report, data summary from January 1992-June 2002, issued August 2002. Am J Infect Control. 2002;30:458–75.
2. Critères d’infection chez les brulés. SFETB-2006.

#### S101 Predictors of treatment failure for patients with pneumonia caused by *Staphylococcus aureus* methicillin-sensitive in a intensive care unit: a retrospective study

##### Pierre-Antoine Allain^1^, Nathallie Gault^2^, Catherine Paugam-Burtz^1^, Arnaud Foucrier^1^

###### ^1^Anesthesiology and critical care, Hospital Center University Beaujon (AP-HP), Clichy, France; ^2^Unité d’épidémiologie et recherche clinique, Hospital Center University Beaujon (AP-HP), Clichy, France

####### **Correspondence:** Arnaud Foucrier - arnaud.foucrier@aphp.fr

*Annals of Intensive Care* 2017, **7**(**Suppl 1**):S101

**Introduction** Infection of the lower respiratory tract is the most common cause of infection in intensive care unit (ICU) (1). Although the attributable mortality of ventilator associated pneumonia remains debated, the recurrence of these infections is always associated with a significant morbidity (2). *Staphylococcus aureus* methicillin-sensitive (SAMS) is one of the most frequently germs involved in ICU pneumonia especially in trauma patients. The aim of the study was to establish the risk factors associated with microbiological treatment failure of pneumonia, caused by SAMS.

**Fig. 23:**
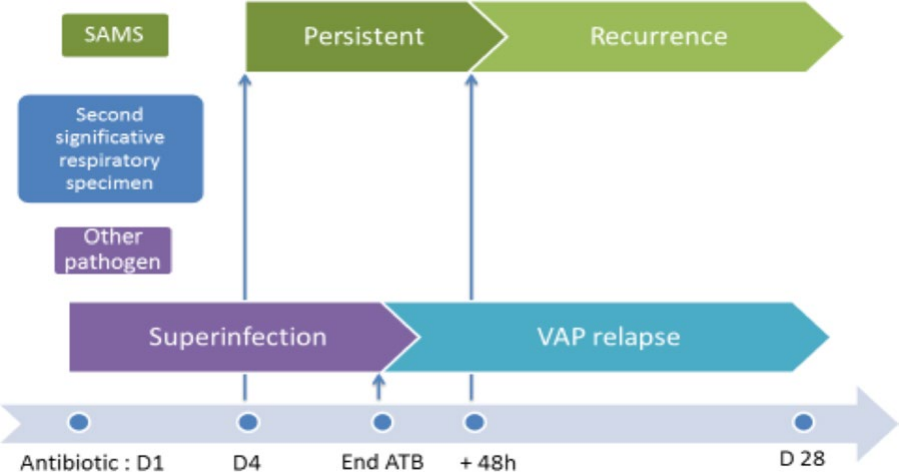
See text for description

**Materials and methods** we retrospectively identified 185 patients who developed a first episode of ventilator associated pneumonia caused by SAMS during a 6 years-period (2009–2014). The primary end point was the microbiological treatment failure defined as a second episode of pneumonia caused by SAMS corresponding to either a persistent or a recurrence of the pneumonia (Fig. 23). The primary aim of the study was to identify factors associated with a treatment failure, the secondary objective was to identify factors associated with the occurrence of second episode (i.e. persistent, recurrence, superinfection and/or relapse of pneumonia caused by any bacteria) during or after treatment of the first episode caused by SAMS. Definition of outcomes was based after analysis of current concepts available in the literature. Factors associated with primary and secondary objectives in univariate analysis (p-value < 0.20), or clinically relevant ones, were entered in a multivariate logistic regression. The final selection was performed using the stepwise selection based on the Akaike criterion.

**Results** Fifty-nine patients (33.3%) developed a second episode of pneumonia and among them, 30 (16.6%) were considered as a microbiological failure. In a multivariate analysis, the association of oropharyngeal flora (FOP) with the SAMS (OR, 3.86; 95% CI, 1.33–11.11; p = 0.011) and the need of emergency surgery (OR, 5.26; 95% CI, 1.39–35.2; p = 0.035) were predictive of a microbiological failure. Empirical antibiotic therapy with amoxicillin-clavulanic acid (OR, 2.28; 95% CI, 1.15–4.51; p = 0.017) and performing emergency surgery (OR, 3.60; 95% CI, 1.34–9.65; p = .011) were predictors of a second episode of pneumonia caused by any bacteria.

**Conclusion** In this retrospective, monocentric study, the co presence of orophryngeal flora and the need of emergency surgery were associated with microbiological failure of pneumonia caused by SAMS in ICU.

**Competing interests** None.

**References**

1. Vincent J-L. International study of the prevalence and outcomes of infection in intensive care units. JAMA. 2009;302(21):2323–9.
2. Cavalcanti M. A. Risk and prognostic factors of ventilator-associated pneumonia in trauma patients. Crit Care Med. 2006;34(4):1067–72.

#### S102 Ventilator-associated pneumonia: never enough, never give up!

##### Sahar Habacha^1^, Bassem Chatbri^1^, Aymen M’rad^1^, Youssef Blel^1^, Nozha Brahmi^1^, Yousra Bourbiaa^2^, Lamia Thabet^2^

###### ^1^Departement of intensive care and toxicology, Centre d’Assistance Médicale Urgente, Tunis, Tunisia; ^2^Biology departement, Trauma Center, Ben Arous, Tunisia

####### **Correspondence:** Sahar Habacha - sahar.habacha@gmail.com

*Annals of Intensive Care* 2017, **7**(**Suppl 1**):S102

**Introduction** Ventilator-associated pneumonia is a major iatrogenic problem since it is a cause of hospital morbidity, mortality and increase of health care costs. It has been studied many times, but data’s revision is always necessary. Our study aimed to describe epidemiology of ventilator-associated pneumonia and identify local causative pathogens.

**Materials and methods** We carried out a prospective study in an intensive care unit. Were included patients intubated for more than 48 h, from April 2015 to May 2016, and presenting signs of ventilator-associated pneumonia (fever, abundant and purulent secretion, increase of FiO_2_ greater than 0.2, signs on chest-X ray) with positive culture of endotracheal aspirate. Were excluded patients with germ colonization.

**Results** A total of 268 patients were ventilated for more than 48 h. Among them thirty-four patients aged of 39 ± 18.5 years presented 54 episodes of ventilator-associated pneumonia (that is 1.58 ± 0.85 episodes per patient). The mean SOFA score was 5.2 ± 1.8. The main reasons of mechanical ventilation were loss of consciousness secondary to poisoning (20%), respiratory distress (20%) and status epilepticus (12%). The mean duration of stay was 62.5 days with extremes at 7 and 180 days. The average time between hospitalization and suspicion of ventilator-associated pneumonia was 5.9 ± 2.7 days. The average value of the Clinical Pulmonary Infection Score at suspicion was 5 ± 1.26. The average time between recurrences was 16.8 days with extremes at 4 and 39 days. The culture of endotracheal aspirate identified two pathogens in 11%. It reveled *Acinetobacter baumanii* in 35% in which 63% were imipenem resistant, *Pseudomonas aeroginosa* in 33%, *Klebsielle pneumoniae* in 22%, *Staphylococcus aureus* methicillin resistant in 7%. Extended spectrum β-lactamases bacteria were found in 10% and carbapenemases producers in 12%. Empirical antibiotherapy was always association of imipenem and colistin. It was necessary to adapt it to antibiograms in 33/54.

Ventilator-associated pneumonia was complicated by septic shock in 28% and acute respiratory distress syndrome in 24%. Patients evolved to healing in 63% of episodes (n = 34), to superinfection in 26% (n = 14) and to death in 9% (n = 5). *Pseudomonas aeruginosa* was the most frequent germ in superinfection (7/14), *Acinétobacter baumanii* was the most pathogen associated to death (3/5).

**Conclusion** Ventilator-associated pneumonia is an iatrogenic disease that threatens lives. It’s in part avoidable. Preventive measures have to be implemented to reduce its frequency, consequences and costs.

**Competing interests** None.

#### S103 Comparative performance of different automated weaning modes: a network meta-analysis

##### Arthur Neuschwander^1^, Looten Vincent^2^, Chhor Vibol^1^, Yavchitz Amelie^1^, Matthieu Resche-Rigon^2^, Jean Mantz^1^, Romain Pirracchio^1^

###### ^1^Anesthésie réanimation, Hôpital Européen Georges-Pompidou (AP-HP), Paris, France; ^2^Biostatistiques, Hôpital Saint-Louis (AP-HP), Paris, France

####### **Correspondence:** Arthur Neuschwander - arthur.neuschwander@gmail.com

*Annals of Intensive Care* 2017, **7**(**Suppl 1**):S103

**Introduction** Mechanical ventilation (MV) weaning is a crucial step in critically ill patients. MV duration is associated with an increased risk of ventilator associated events, even though its specific impact on mortality has never been clearly demonstrated (1). Automated closed loop systems might help the weaning process. A recently published meta-analysis has reported a reduction in MV duration when using an automated weaning mode as compared to non-automated mode (2). However, the different automated modes have not been compared to each other. The objective of this network meta-analysis was to compare the performance of the three major automated weaning modes, i.e. the Automode°, the Smartcare° and the Adaptative Support Ventilation (ASV°) for MV weaning in critically ill and post-operative adult patients.

**Materials and methods** We included all randomised control trials that compared automated closed loop weaning applications either to another automated application or standard care, including weaning according to a written weaning protocol or nurse driven protocols. The three modes of automated modes included in the study were ASV°, Smartcare° and Automode°. The primary outcome was the duration of MV weaning, defined as the time between randomization and a successful extubation. We also planned subgroup analyses in the ICU and the post-operative populations. The quality of the studies was assessed independently by two blinded investigators, using the evaluation recommended by the Cochrane Collaboration. A network Bayesian meta-analysis using random effect models and based on aggregate data from the included studies was performed using the gemtc package (R project, Vienna). This trial was declared in PROSPERO in August 2015 (CRD42015024742).

**Fig. 24:**
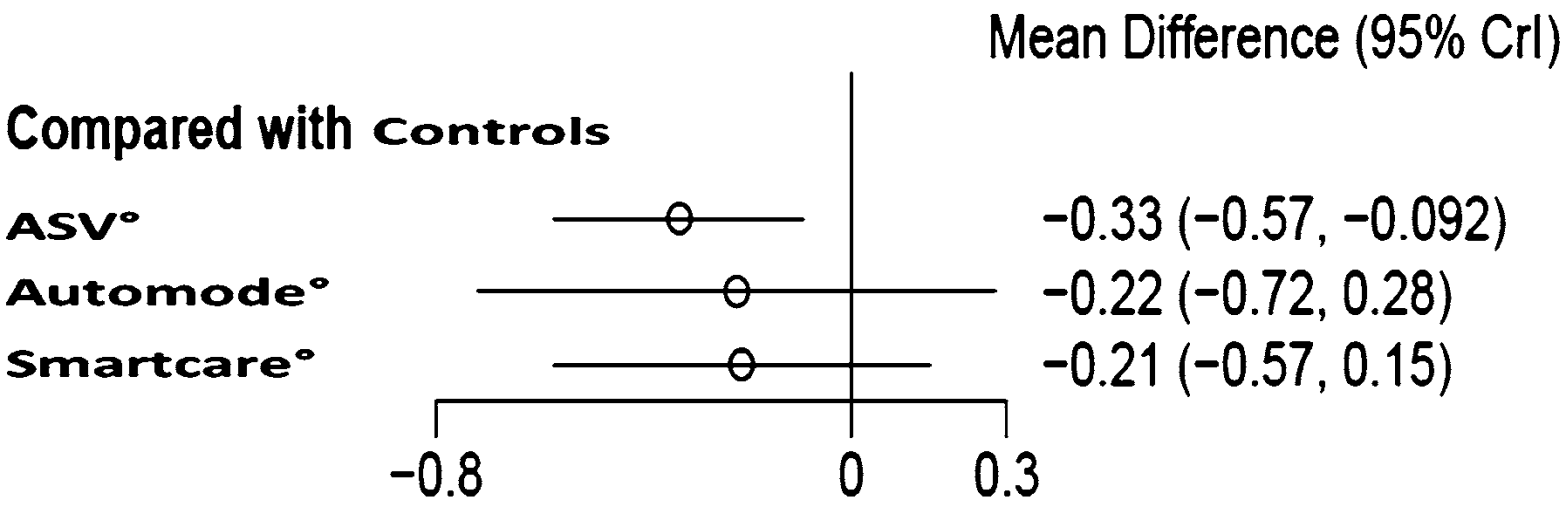
Bayesian NMA with random effect

**Results** *S*earch of databased identified 776 articles; 233 were screened for eligibility after removal of duplicates. Abstract analysis led to the exclusion of 191 articles with a final full text analysis of 42 randomised control trials. Ultimately, 15 trials were included in the analysis, representing 1424 ventilated patients. Nine studies included patients in the post-operative period while six were conducted in ICU. The automated mode was ASV° (A) in 9 studies, Smartcare° (C) in 4 studies and Automode° (B) in 2 studies. All studies reported the duration of MV weaning as defined in our protocol. In all studies, the control group was standard care with a weaning process driven either by nurses or physicians. In 12 studies (75%) a written weaning protocol was used in the control group. All ICU studies used sedation protocols based on sedation scores, none of them including systematic daily sedation interruption. Each one of the automated application was associated with a significant reduction in the duration of MV as compared to the control. When comparing all different modes using the network meta-analysis framework, ASV° appeared to be the best automated mode when it pertains to reducing the duration of mechanical ventilation weaning (Fig. 24). Subgroup analysis showed similar results in the post-operative and the ICU populations.

**Conclusion** Compared to standard weaning practice, the 3 major automated weaning modes significantly reduced the duration of MV weaning in critically ill and post-operative adult patients. ASV° was associated with the most significant effect when compared to the two other automated modes (Smartcare°, Automode°). Further physiological respiratory studies would help to understand the underlying mechanisms accounting for the superiority of ASV.

**Competing interests** None.

**References**

1. McConville JF, Kress JP. Weaning patients from the ventilator. N Engl J Med. 2012;367(23):2233–9.
2. Rose L, Schultz MJ, Cardwell CR, Jouvet P, McAuley DF, Blackwood B. Automated versus non-automated weaning for reducing the duration of mechanical ventilation for critically ill adults and children: a cochrane systematic review and meta-analysis. Crit Care Lond Engl. 2015;19:48.

#### S104 Impact of proportional assisted ventilation on dyspnea and asynchrony in mechanically ventilated patients

##### Côme Bureau^1^, Maxens Decavèle^1^, Sébastien Campion^2^, Roukia Ainsouya^2^, Marie-Cécile Niérat^2^, Julien Mayaux^3^, Hélène Prodanovic^3^, Mathieu Raux^4^, Thomas Similowski^1^, Alexandre Demoule^1^

###### ^1^Réanimation médicale, inserm umr_s 1158 neurophysiologie respiratoire expérimentale et clinique, Pitié-Salpêtrière Hospital, Paris, France; ^2^Inserm umr_s 1158 “neurophysiologie respiratoire expérimentale et clinique”, Pitié-Salpêtrière Hospital, Paris, France; ^3^Unité de réanimation et de surveillance continue, service de pneumologie et réanimation médicale, Pitié-Salpêtrière Hospital, Paris, France; ^4^Département d’anesthésie-réanimation et urm_s 1158, Hôpital Universitaire Pitié-Salpêtrière, Paris, France

####### **Correspondence:** Côme Bureau - come.bureau@gmail.com

*Annals of Intensive Care* 2017, **7**(**Suppl 1**):S104

**Introduction** During mechanical ventilation, mismatch between respiratory muscles activity and the assistance delivered by the ventilator results in dyspnea and asynchrony and is commonly observed in intensive care unit (ICU) patients. Proportional Assisted Ventilation (PAV) is a ventilatory mode that adjusts the level of ventilator assistance to the activity of respiratory muscles estimated by an algorithm. To date, PAV has been mostly studied in patients without severe dyspnea or asynchrony. We hypothesized that, compared to pressure support ventilation (PSV), PAV will prevent severe dyspnea or asynchrony.

**Patients and methods** Were included ICU mechanically ventilated patient exhibiting severe dyspnea or asynchrony with PSV. Three conditions were successively studied: 1) PSV on inclusion (Baseline), 2) PSV after optimisation of ventilator settings in order to minimize dyspnoea and asynchrony (Optimisation), and 3) PAV. Ten-minutes recording were performed with each condition. The intensity of dyspnea was assessed by the Visual Analogic State (VAS, only in patients able to communicate) and by the Intensive Care Respiratory Distress Operating Scale (IC-RDOS) for all the patients. The electrical activity (EMG) of extradiaphragmatic inspiratory muscles was measured. The prevalence of asynchrony was quantified by the visual inspection of the airway flow and pressure traces.

**Fig. 25:**
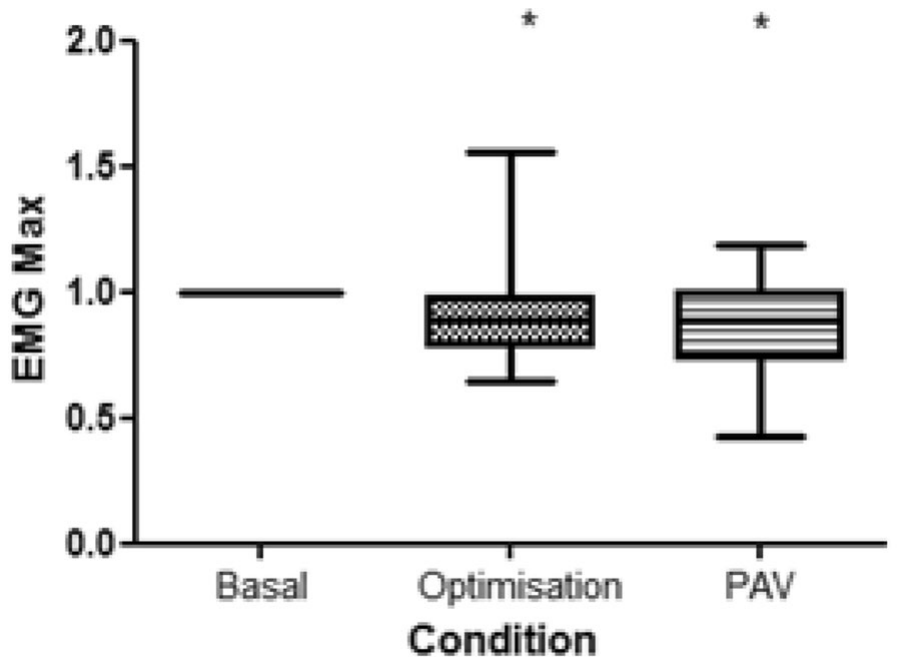
Electromyographic of activity parasternal muscles. *p < 0. 05 compared to basal condition

**Results** 34 patients were included, 74% male, aged 66 [58–78] years, SAPS2 57 [39–66], mechanically ventilated for 6 [4–9] days. The tidal volume (Tv) was higher in the Optimisation and PAV than in the Basal condition (Table 27). The respiratory rate(RR) was lower with PAV than in the other conditions. The dyspnea-VAS was lower with Optimisation and PAV than with the Basal conditions. The IC-RDOS was lower with PAV than with the two other conditions. The asynchrony index was lower with PAV than with the two other conditions. Parasternal EMG activity was lower with PAV and Optimisation (Fig. 25).

**Table 27:** Breathing pattern, dyspnea score and prevalence of asynchrony according to condition

Variables	Basal	Optimisation	PAV	*p*
RR (min^−1^)	27 [22–32]	24 [20–30] *	21 [17–28] *^,§^	<0.0001
Tidal volume (ml)	489 [373–624]	532 [446–726]*	629 [452–733]*	<0.0001
VAS for Dyspnea. (mm)	62 [28–76]	37 [20–55] *	31 [14–45] *	<0.001
IC-RDOS	4.6 [4.1–6.5]	4.3 [2.3–4.8]	4.2 [2.4–4.7] *	0.002
Asynchrony index (%)	0.68 [0–2.28]	0.6 [0.31–1.4]	0 [0–0.55] *^,§^	0.005

* *p* < 0. 05 compared to basal condition; ^§^ *p* < 0. 05 compared to optimisation condition

**Conclusion** In ICU patients receiving mechanical ventilation with PSV and exhibiting severe dyspnea or asynchrony, the optimisation of ventilator settings with PSV and the PAV mode decrease in the simiar way the severity of dyspnea and the prevalence of patient-ventilator asynchrony.

**Competing interests** None.

#### S105 Validation of ultrasound to assess diaphragm function in mechanically ventilated patients

##### Alexandre Demoule^1^, Bruno-Pierre Dubé^1^, Julien Mayaux^1^, Suela Demiri^1^, Thomas Similowski^1^, Martin Dres^1^

###### ^1^Service de pneumologie et réanimation médicale, Hôpital Pitié-Salpêtrière, Paris, France

####### **Correspondence:** Alexandre Demoule - alexandre.demoule@aphp.fr

*Annals of Intensive Care* 2017, **7**(**Suppl 1**):S105

**Introduction** In intensive care unit (ICU) patients, diaphragm dysfunction is associated with adverse clinical outcomes. Ultrasound measurements of diaphragm thickness (Tdi), excursion (EXdi) and thickening fraction (TFdi) have been proposed as estimators of diaphragm function, but have never been compared to phrenic nerve stimulation.

Our aim was to describe the relationship between Tdi, EXdi, TFdi and diaphragm function evaluated using the change in endotracheal pressure after phrenic nerve stimulation (Ptr,stim), and to compare their prognostic value.

**Patients and methods** Ptr,stim and ultrasound variables were measured in mechanically ventilated (MV) patients <24 h after intubation (“initiation of MV”, under assist-control ventilation, ACV) and at the time of switch to pressure-support ventilation (“switch to PSV”). Diaphragm dysfunction was defined as Ptr,stim <11 cmH_2_O.

**Results** 112 patients were included. At initiation of MV, Ptr,stim was not correlated to Tdi (rho = −0·13, p = 0·28), EXdi (rho = 0·10, p = 0·66) or TFdi (rho = −0·03, p = 0·80). At switch to PSV, TFdi and EXdi were correlated to Ptr,stim, (rho = 0·87, p < 0.001 and 0·45, p = 0·001, respectively), but Tdi was not (rho = −0·09, p = 0·45). At switch to PSV, a TFdi <29% could reliably identify diaphragm dysfunction (sensitivity and specificity of 85 and 88%, respectively), but Tdi and EXdi could not. This value was associated with increased duration of ICU stay and MV, and mortality.

**Conclusion** Under ACV, neither Tdi, EXdi nor TFdi were related to Ptr,stim. Under PSV, TFdi was strongly correlated to diaphragm strength and, when decreased, was associated with poorer outcome.

**Competing interests** Alexandre Demoule has signed research contracts with Covidien, Maquet and Philips; he has also received personal fees from Covidien and MSD.

#### S107 End-tidal carbon dioxide during spontaneous breathing trial to predict extubation failure: a single center prospective observational study

##### Faten May^1^, Keyvan Razazi^1^, François Bagate^1^, Guillaume Carteaux^1^, Nicolas de Prost^1^, Armand Mekontso Dessap^1^

###### ^1^Réanimation médicale, Hôpital Henri Mondor, Créteil, France

####### **Correspondence:** Faten May - m_faten2001@yahoo.fr

*Annals of Intensive Care* 2017, **7**(**Suppl 1**):S107

**Introduction** In spite of recent research and progress in weaning protocols, extubation failure still occurs in 10–20% of patients and is associated with poor outcomes, with a mortality rate of 25–50%. Many risk factors for planned extubation failure have been suggested, including hypercapnia at end of spontaneous breathing trial (SBT). However, performing arterial blood gases at the end of SBT is not routinely recommended whereas EtCO2 may be routinely monitored during a low pressure support SBT. The aim of this prospective observational study was to determine the clinical usefulness of EtCO2 to predict extubation failure.

**Patients and methods** We recorded clinical data and EtCO2 during a successful 1 h low level pressure support SBT (at the beginning, after 5 min and at the end of the trial). Patients ventilated through tracheostomy and unplanned extubations were excluded. Extubation failure was defined as death or the need for reintubation within 72 h (1) after extubation; this delay was prolonged to 7 days (2) in case of noninvasive ventilation after extubation, which was systematic in older patients or those with cardiorespiratory disease, as per our weaning protocol. Multivariable logistic regression analysis was performed to identify independent variables associated with extubation failure.

**Results** One hundred and fifteen ventilated patients were enrolled in our study from July 2015 to June 2016. The median age of these patients was 63 [52–75] years, their median Simplified Acute Physiology Score (SAPS) II was 48 [38–61] points and 42.5% (n = 49) were female. Seventeen (15%) patients had chronic obstructive pulmonary disease. Reintubation rate was 15% (n = 18). EtCO2 at the end of SBT was similar between patients with failed and successful extubation: 38 [29–41] vs. 35 [30–40] mmHg, p = 0.9. EtCO2 at other time points as well as its changes during the SBT were also similar between groups. The three variables predicting extubation failure in the multivariable logistic regression model were a past medical history of cirrhosis, acute respiratory distress syndrome before weaning and lower minute ventilation at the end of SBT.

**Conclusion** EtCO2 during a successful SBT seems useless to predict outcome of extubation.

**Competing interests** None.

**References**

1. Girault C, Bubenheim M, Abroug F, Diehl JL, Elatrous S, Beuret P, et al. Noninvasive ventilation and weaning in patients with chronic hypercapnic respiratory failure: a randomized multicenter trial. Am J Respir Crit Care Med. 2011;184(6):672–9.
2. Frutos-Vivar F, Ferguson ND, Esteban A, Epstein SK, Arabi Y, Apezteguia C, et al. Risk factors for extubation failure in patients following a successful spontaneous breathing trial. Chest. 2006;130(6):1664–71.

#### S109 A multicenter prospective observational study of 1514 extubation procedures in 26 intensive care units: the FREE-REA study

##### Samir Jaber^1^, Hervé Quintard^2^, Audrey De Jong^1^, for the FREE-REA study group

###### ^1^DAR B, Hôpital Saint Eloi, Montpellier, France; ^2^Réanimation polyvalente, Hôpital Saint-Roch, Nice, France

####### **Correspondence:** Audrey De Jong - audreydejong@hotmail.fr

*Annals of Intensive Care* 2017, **7**(**Suppl 1**):S109

**Introduction** Airway management in intensive care unit (ICU) patients is challenging [1]. “Airway failure”, defined as the inability to breathe without endotracheal tube, differs from “weaning failure”, defined as the inability to breathe without an invasive mechanical ventilation. However, most of the studies assessing predictive factors of extubation failure did not separate airway from weaning failure. We aimed to describe incidence of extubation failure in critically ill patients, separating for the first time airway from weaning failure, in a prospective multicenter observational study.

**Patients and methods** A prospective, observational, multicenter study was conducted in 26 French ICUs. All adult patients consecutively extubated in ICU were included. An ethics committee approved the study design (code UF: 9242, register: 2013-A01402-43). The study was registered on clinicaltrials.gov (identifier no.NCT 02450669). Clinical parameters were prospectively assessed before, during and after extubation procedure. Extubation failure was defined as the need to reintubate less than 48 h after extubation. Extubation failure could be due to airway failure, weaning failure or mixed airway and weaning failure.

**Fig. 26:**
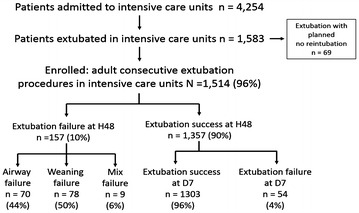
Flow chart of the FREE-REA study

**Results** From December 2013 to May 2015, 1514 intubation-procedures were studied in 1465 patients from 26 centers. 49 patients (3.2%) were intubated twice. The median number of intubation-procedures included by center was 27 (11–72). The flow chart of the study is shown in Fig. 26. Incidence of extubation failure was 10.4% (157 of 1514 intubation-procedures). Incidence of airway failure, weaning failure and mixed failure were respectively 4.6% (70 of 1514), 5.2% (78 of 1514) and 0.6% (9 of 1514).

**Conclusion** Extubation failure at 48 h occurred in 10.4% of the 1514 extubation procedures recorded, 44% due to airway failure, 50% to weaning failure and 6% to mixed airway and weaning failure. Specific risk factors will be determined using this multicenter database.

**Competing interests** None.

**Reference**

1. De Jong A, Molinari N, Terzi N, Mongardon N, Arnal JM, Guitton C, Allaouchiche B, Paugam-Burtz C, Constantin JM, Lefrant JY, et al. Early identification of patients at risk for difficult intubation in the intensive care unit: development and validation of the MACOCHA score in a multicenter cohort study. Am J Respir Crit Care Med 2013;187(8):832–39.

#### S110 Efficiency and safety of total plasma exchange in critically ill cirrhotic patients with acute on chronic liver failure admitted to the ICU

##### Ilias Kounis^1^, Faouzi Saliba^1^, Stephane André^1^, Marc Boudon,^1^, Philippe Ichai^1^, Aline Younes^1^, Lionel Nakad^1^, Audrey Coilly^1^, Teresa Antonini^1^, Rodolphe Sobesky^1^, Eleonora De Martin^1^, Didier Samuel^1^

###### ^1^Centre hépato-billiaire, APHP Hôpital Paul-Brousse, Villejuif, France

####### **Correspondence:** Faouzi Saliba - faouzi.saliba@pbr.aphp.fr

*Annals of Intensive Care* 2017, **7**(**Suppl 1**):S110

**Introduction** Acute on chronic liver failure (ACLF) have been recently defined by an acute decompensation of a chronic liver disease associated to organ failure and a high mortality rate. Few authors reported on the use of total plasma exchange (TPE) in patients with the current definition of ACLF. The aim of this pilot study was to evaluate the efficiency and safety of TPE in critically ill cirrhotic patients admitted with ACLF in the ICU.

**Patients and methods** A prospective cohort of cirrhotic patients admitted to the ICU between February 2015 and February 2016. TPE was performed using a plasma filter (TPE2000, Hospal^®^) on a CVVHDF machine (Prismaflex^®^, Baxter^®^) connected to the patient with a femoral double lumen 13F catheter. The plasma volume exchanged per session was 1.3–1.5 of the total plasma volume. Ratio and type of fluid replacement were 50% with 5% albumin solution followed by 50% with fresh frozen plasma. Clinical and biological parameters, and the following scores MELD, SOFA, CLIF-SOFA, CLIF-OF and Child Pugh were evaluated prior, after TPE session and 7 days distant of treatment.

**Results** Seven male patients with a mean age of 50.6 ± 7.7 years comprised the study and had a total of 20 TPE sessions. The etiology of cirrhosis was alcoholic (n = 6) or post-HCV (n = 1). The reasons of ACLF were acute alcoholic hepatitis (n = 2), variceal bleeding (n = 2) and sepsis (n = 3). Prior to TPE, the mean scores of SOFA, CLIF-SOFA, CLIF-OF, MELD and Child-Pugh were respectively 9.4, 12, 11.7, 37.6 and C12.5. Mean total bilirubin prior and after TPE sessions was reduced from 392.3 ± 117.8 µmol/l to 244.9 ± 93.2 µmol/l (reduction of 37.5%; p = 3.81E−6); at day 7, mean total bilirubin was still lower at 333 ± 132 µmol/l (p = 0.1). Mean INR prior and after TPE improved from 5.54 ± 3.38 to 2.32 ± 0.80 (reduction of INR of 58.1%, p = 4.76E−5) and at day 7 of treatment at 4 ± 1.64 (reduction of 43%, p = 0.125). Mean GGT levels reduced by 28.5% (p = 0.01). Mean platelet counts (50.8 ± 19.5 G/L) reduced by 10.2% (p = NS). The probability of survival at 10, 28 and 90 days was 42.8, 28.6 and 14.3%. One patient was transplanted and still alive. Tolerance during sessions was good similar to CVVHDF. Two side effects related to the femoral catheter were observed (bacteremia and hemorrhagic shock post catheter ablation).

**Conclusion** This preliminary study of TPE in ACLF showed a marked reduction of liver enzymes and improvement in coagulation parameters with a relative good safety. A specific caution should be undertaken regarding catheter related complications. TPE worth to be evaluated in large trials in ACLF’ patients, with a liver transplant project, and a lesser degree of organ failure.

**Competing interests** None.

#### S111 Management of enteral feeding during extubation in the intensive care unit: a multi-center retrospective study in 11 French intensive care units

##### Mickael Landais^1^, Noemie Hubert^2^, Mai-Anh Nay^3^, Johann Auchabie^4^, Bruno Giraudeau^5^, Reignier Jean^6^, Arnaud W Thille^7^, Stephan Ehrmann^8^

###### ^1^Service de réanimation médicale, CHU Hôtel-Dieu Nantes, Nantes, France; ^2^Réanimation, C.H.U de Caen, Caen, France; ^3^Réanimation médicale polyvalente, Hôpital de La Source, CHR Orléans, Orléans, France; ^4^Réanimation médicale, Centre Hospitalier Universitaire d’Angers, Angers, France; ^5^Unité de biostatistiques, Hôpital Bretonneau, Tours, France; ^6^Réanimation médicale, CHU Hôtel-Dieu Nantes, Nantes, France; ^7^Réanimation Médicale, CHU de Poitiers, Poitiers, France; ^8^Réanimation polyvalente, CHRU Hôpitaux de Tours, Tours, France

####### **Correspondence:** Mickael Landais - mickaelandais@gmail.com

*Annals of Intensive Care* 2017, **7**(**Suppl 1**):S111

**Introduction** Extubation is a key moment for the patient on his way to recovery. Extubation failure concerns 10–20% of ICU patients and is closely linked to nosocomial pneumonia. The practice concerning enteral feeding interruption at time of extubation has not been investigated. Fasting before extubation may prevent aspiration and development of nosocomial pneumonia. Thus, fasting and gastric content suctioning before extubation may be reasonably considered as a mean to reduce this burden. Fasting before extubation may prevent aspiration and development of nosocomial pneumonia. Thus, fasting and gastric content suctioning before extubation may be reasonably considered as a mean to reduce this burden. However, fasting, as recommended before elective general anesthesia is likely to be ineffective in the setting of extubation in the ICU, due to patients’ gastroparesis and prolonged gastric stasis. Beyond the potentially unnecessary burden in terms of paramedical workload, fasting may have some side effects such as caloric deficit, hypoglycemia, or delayed extubation. Given the current lack of objective data concerning the clinical practice of feeding/fasting and gastric tube suctioning before extubation in the ICU, we undertook this descriptive study to assess current practice.

**Materials and methods** We conducted a retrospective, multicenter study in eleven intensive care units in the west of France over a 1 month timespan. All patients extubated were included and data about enteral feeding during the peri-extubation period as well as extubation failure and nosocomial that pneumonia occured within 7 days were recorded. Data observed in the eleven participating centers were completed with a short email survey concerning declarative practice performed among 43 intensive care units.

**Results** During the study period, 162 patients were included. Overall, 25 patients (15%) failed extubation and needed reintubation within the 7 days following planned extubation. Pneumonia was significantly more frequent reintubated patients than the other (36 vs. 4%, p < 0.01). Hundred patients (62%) received enteral feeding at the time of extubation. Compared to patients who did not receive enteral feeding, those patients had a higher disease severity (SAPSII score 50, [41; 63] vs. 45 [35; 52], p < 0.01; longer duration of mechanical ventilation 7 [4; 13] vs. 1.5 [1; 3] days, p < 0.01). Accordingly, those patients had a higher rate of extubation failure (21 vs. 7%, p = 0.01) and pneumonia (13 vs. 3%, p = 0.05). Among the 100 patients receiving enteral feeding, fasting was implemented before extubation for 64 patients (64%). Median fasting duration before extubation was 6 h [2; 13]. Despite a higher severity of disease of patients undergoing fasting before extubation (SAPS II of 50 [42; 66] vs. 48 [31; 60], p = 0.05). The rate of extubation failure was similar between the fasting patients and the others: 15 of 64 patients (23%) versus 6 of 36 (17%) of the patient (p = 0.60). Similarly, the incidence of pneumonia was not different between groups (n = 9 (14%) vs. n = 4 (11%), p = 0.76). After extubation, the fasting patients experienced a longer delay until feeding resumption as compared to non-fasting patients (21 h [6; 42] vs. 8 [5; 22]), but this difference did not reach statistical significance. Overall gastric content suctioning before extubation was not commonly performed; before extubation: 30% of the fasting patients and 26% of the non fasting patients.

Among the 11 participating centers, while some centers imposed a fasting period before extubation to all their patients, some did it infrequently. However, no center never imposed fasting, illustrating between and within center heterogeneity.

This heterogeneity was confirmed on the larger scale declarative email survey (88% response rate amont 43 units) which showed that only 44% of the units had a written standardized operational procedure for extubation. Survey respondents reported to practice fasting before extubation “Always”, *“Frequently”* and *“Never or Rarely”* in respectively 71, 21 and 8% of cases.

**Conclusion** Both practices, fasting as well as pursued nutrition until extubation are commonly performed in ICUs, with little standardization of practice. Safety seems equivalent, as no clinically significant difference in terms of reintubation rate and pneumonia were observed. Thus, the equipoise condition appears met to undertake a trial evaluating feeding strategies in the peri-extubation period.

**Competing interests** None.

**References**

1. Smith I, Kranke P, Murat I, Smith A, O’Sullivan G, Søreide E, Spies C, in’t Veld B; European Society of Anaesthesiology. Perioperative fasting in adults and children: guidelines from the European Society of Anaesthesiology. Eur J Anaesthesiol. 2011;28:556–69.
2. Thille AW, Richard JC, Brochard L. The decision to extubate in the intensive care unit. Am J Respir Crit Care Med. 2013;187:1294–1302.

#### S112 More than half the patients receiving non-invasive ventilation are fasting

##### Nicolas Terzi^1^, Michaël Darmon^2^, Jean Reignier^3^, Stephane Ruckly^4^, Maïté Garrouste-Orgeas^5^, Elisabeth Gratia^1^, Alexandre Lautrette^6^, Elie Azoulay^7^, Bruno Mourvillier^8^, Laurent Argaud^9^, Laurent Papazian^10^, Marc Gainnier^11^, Dany Goldgran-Toledano^12^, Samir Jamali^13^, Anne Sylvie Dumenil^14^, Carole Schwebel^15^, Jean-François Timsit^16^, OUTCOMEREA study group

###### ^1^Service de réanimation médicale, Clinique de Réanimation Médicale, Grenoble, France; ^2^Réanimation Médicale, CHU Saint-Etienne - Hôpital Nord, Saint-Étienne, France; ^3^Réanimation médicale, CHU Hôtel-Dieu Nantes, Nantes, France; ^4^Reanimation, Hôpital Bichat-Claude Bernard (AP-HP), Paris, France; ^5^Réanimation, Fondation Hopital Saint Joseph, Paris, France; ^6^Réanimation médicale, CHU Gabriel-Montpied, Clermont-Ferrand, France; ^7^Réanimation médicale, Hôpital Saint-Louis, Paris, France; ^8^Réanimation Médicale et Infectieuse, GH Bichat Claude Bernard, Paris, France; ^9^Réanimation Médicale, Hospices Civils de Lyon - Groupement Hospitalier Edouard Herriot, Lyon, France; ^10^Service de réanimation-détresses respiratoires et infections sévères, Hôpital Nord, Marseille, France; ^11^Réanimation des urgences médicales, Hôpital de la Timone, Marseille, France; ^12^Réanimation polyvalente, Centre Hospitalier Général, Gonesse, France; ^13^Réanimation médicale, Centre Hospitalier Sud Essonne, Dourdan, France; ^14^Réanimation chirurgicale, Hôpital Antoine Béclère, Clamart, France; ^15^Réanimation médicale, C.H.U. Grenoble, Grenoble, France; ^16^Réanimation médicale et infectieuse, Hôpital Bichat-Claude Bernard, Paris, France

####### **Correspondence:** Nicolas Terzi - terzinicolas@gmail.com

*Annals of Intensive Care* 2017, **7**(**Suppl 1**):S112

**Introduction** Noninvasive ventilation (NIV) has become a cornerstone for the supportive therapy of acute respiratory failure (ARF). Survival benefits in chronic obstructive pulmonary disease (COPD) and cardiac patients have been demonstrated. Although ARF and COPD patients are at risk of malnutrition that adversely affects patient outcomes, few data are available regarding the management of nutritional support in non-invasively ventilated patients. We sought to describe nutritional management in patients receiving NIV as the first line therapy for ARF. Secondary objectives were to assess the impact of early nutrition use on the need for invasive mechanical ventilation, occurrence of ICU-acquired pneumonia, length of stay, and death.

**Patients and methods** We conducted an observational study from the multicenter French database fed by 20 French ICUs. Our institutional review board approved this study. Adult medical patients admitted to the ICU and receiving NIV for more than 2 days were included. Exclusion criteria were patients admitted after surgery, readmitted in ICU, patients with neuromuscular disease and treatment-limitation decisions on admission. Four groups of patients were defined according to nutrition received during the first 2 days of NIV: (1) No nutrition; (2) Enteral nutrition: patients who received enteral nutrition with or without parenteral nutrition; (3) Parenteral nutrition only (3) Oral nutrition only.

The impact of nutrition on day-28 mortality was assessed through the use of a Cox model adjusted on clinically relevant covariates. The impact of nutrition on other secondary end-point i.e. ICU-acquired pneumonia occurrence, need for invasive mechanical ventilation were assessed using a Fine & Gray models. Patients were censored after 28 days of follow-up. Choice among collinear variables was performed considering clinical relevance, rate of missing variables and reproducibility of definitions. Results were given as hazard ratio (HR) for Cox models and subdistribution hazard ratios (sHR) and 95% confidence intervals (CI). The impact on duration of stay was estimated by a multivariate Poisson regression. P values less than 0.05 were considered as significant. Statistical analysis was performed using SAS 9.4 (Cary, NC).

**Results** During the study period, 16,734 patients were included in the database and 1075 met inclusion criteria. Among them, 622 received no nutrition; 28 received enteral nutrition, 74 received parenteral nutrition only, and 351 received oral nutrition only. Overall, 86 patients developed ICU-acquired pneumonia (8%), 158 required invasive mechanical ventilation (14.7%) and 161 died before day-28 (15%). Median length of stay was 6 days [4; 9].

After adjustment for confounders, type of nutrition support was associated with an increase day-28 mortality (P = 0.02). Compared to *oral* nutrition, enteral nutrition was associated with an increase day-28 mortality [sHR 2.91, 95% CI 1.44–5.89; P = 0.003] whereas parenteral nutrition and no nutrition did not influence this outcome. The type of nutrition was not associated with the occurrence of ICU-acquired pneumonia (P = 0.18). However, patients who received enteral nutrition experienced more frequently ICU-acquired pneumonia [sHR = 3.00, 95% CI 1.08–8.37; P = 0.036] as compared to oral nutrition patients. Ventilator free days within the 28 days were negatively associated with the type of nutrition (P < 0.0001). Compared to oral nutrition, parenteral and enteral nutrition were negatively associated with ventilator free days within the 28 days [RR per day = 1.48, 95% CI 1.22–1.78; P < 0.0001 and RR per day = 1.77, 95% CI 1.37–2.30; P < 0.0001]. Delta PaCO_2_ measured between the first 2 days was not associated with any type of nutrition.

**Conclusion** More than half the patients receiving NIV were fasting within the first two NIV days. Oral nutrition was prescribed for one-third of them and was well tolerated. Lack of feeding or underfeeding had no impact on mortality and ventilator free days within the 28 days. However, enteral nutrition was associated with an increased occurrence of ICU-aquired pneumonia and a higher mortality rate.

**Competing interests** None.

#### S113 Nutritional support in patients receiving temporary extracorporeal life support: a retrospective cohort study

##### Arthur Bailly^1^, Laurent Brisard^1^, Philippe Bizouarn^1^, Thierry Lepoivre^1^, Johanna Nicolet^1^, Jean Christophe Rigal^1^, Jean Christian Roussel^2^, Bertrand Rozec^1^

###### ^1^Réanimation ctcv transplantation thoracique, CHU de Nantes - Hôpital Nord Laennec, Saint-Herblain, France; ^2^Chirurgie ctcv transplantation thoracique, CHU de Nantes - Hôpital Nord Laennec, Saint-Herblain, France

####### **Correspondence:** Laurent Brisard - laurent.brisard@chu-nantes.fr

*Annals of Intensive Care* 2017, **7**(**Suppl 1**):S113

**Introduction** The optimal nutritional intake in patients receiving temporary extracorporeal life support (ECLS), including extracorporeal membrane oxygenation (ECMO) venovenous (VV) or venoarterial (VA), remains controversial. Enteral nutrition (EN) is suspect to increase risk of gastrointestinal (GI) intolerance and intestinal ischemia. So, total parenteral nutrition (TPN) is often preferred. The purpose of this study is to describe the nutrition practices for critically ill patients receiving ECLS and identify opportunities for improving nutrition therapy in this population.

**Patients and methods** Retrospective analysis of patients requiring ECMO-VA or ECMO-VV between 2010 and 2014 in the cardiac surgery intensive care unit of the University Hospital of Nantes. Nutritional support was daily monitored with parenteral intake (glucose, lipid and propofol, protein and albumin, parenteral nutrition) and enteral nutrition until ECLS weaning. Two groups were compared during ECLS period: no enteral nutrition delivered (none or TPN) (ANEC, n = 73) and at least once enteral nutrition delivered (NEC, n = 50) including EN alone and supplemental parenteral nutrition (SPN). Primary outcome was incidence of GI intolerance and risk factors. Secondary outcomes were nutritional adequacy (calculated as overall of calories and protein delivered divided by the theoretical amount requirements: 20 kcal/kg/d and 1.2 g/kg/d) and clinical outcome. Data are reported as median (25th and 75th percentiles) or number (%), and analyzed with student’s *t* test for continuous variables and χ2 test for categorical variables. P < 0.05 was considered as significant.

**Fig. 27:**
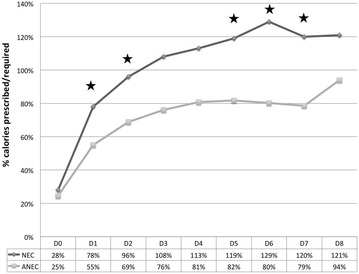
Daily proportion of target energy delivered on first 8 days

**Results** 123 patients were enrolled [age 49 years (38–59), IGS-II 51 (35–67), IMC 24 (22–29)], represented 1009 nutrition days [ANEC = 428 (42), NEC = 581 (58)] with duration of 7 days (5–10) [ANEC = 6 (5–8), NEC = 9 (6–13); P < 0.001] including 96 ECMO-VA and 27 ECMO-VV. None nutrition support and TPN were respectively received on 42% (n = 428) and 32% (n = 321) of patient days (NEC = 105, SPN = 155). Patients received median intake of 1188 kcal (751–1784) [NEC = 1458 kcal (952–2016), ANEC = 990 kcal (605–1389); P = 0.001]. The median ratio of prescribed/required calories per day was 83% (50–127) [NEC = 105% (61–144), ANEC = 70% (42–101); P = 0.001]. Evolution of daily energy balance is reported in Fig. 27. Digestive intolerance was reported in 27 patients (54%) including gastric residual volume considered high for physician in 15 cases (30%). Motility agents were used in 18 patients(36%). No serious GI complications or clinical signs of mesenteric ischemia were reported. Risk factors for GI intolerance were low weight [66 kg (56–75) vs. 80 kg (68.5–86); P = 0.034] and long duration of circulatory support [8 days (5–9.5) vs. 11 days (6.5–14.5); P = 0.018]. Clinical outcome was similar between the groups (NEC vs. ANEC; ECMO-VA vs. ECMO-VV).

**Discussion** TPN was preferably used in this specific cohort of critically ill patients. Although, the number of patients receiving no nutrition was high, caloric debt during temporary ECLS was low in comparison with previous results [1]. Overnutrition was frequent in the NEC group and would justify implementation of nutrition protocol. Incidence of GI intolerance remains frequent and could justify systematic used of motility agents with introduction of EN.

**Conclusion** Enteral nutrition in patients treated with temporary extracorporeal life support is feasible and may be improve with systematic motility agents and implementation of nutritional protocol.

**Competing interests** None.

**Reference**

1. Ferrie, et al. Intensive Care Med 2013;39:1989–94.

#### S114 Refeeding hypophosphoremia in a medical critical care unit: 3-month observational study

##### Gioia Gastaldi^1^, Cherifa Cheurfa^2^, Julien Abily^1^, Steven Grange^1^, Dorothée Carpentier^1^, Christophe Girault^1^, Gaetan Beduneau^1^, Thomas Lescot^3^, Fabienne Tamion^1^

###### ^1^Réanimation médicale, Centre Hospitalier Universitaire Rouen, Rouen, France; ^2^Anesthésie réanimation, Centre Hospitalier Universitaire Rouen, Rouen, France; ^3^Réanimation chirurgicale digestive, Hôpital Saint-Antoine, Paris, France

####### **Correspondence:** Gioia Gastaldi - gioia.gastaldi@chu-rouen.fr

*Annals of Intensive Care* 2017, **7**(**Suppl 1**):S114

**Introduction** Refeeding syndrome (RS) is a potentially lethal condition that remains underdiagnosed. It is characterized by severe electrolyte and fluid shifts associated with metabolic abnormalities in malnourished patients undergoing refeeding orally, enterally, or parenterally. Clinical criteria have been proposed for determination of its risk and reported in the National Institute for Clinical Excellence (NICE) Clinical Guidelines. Hypophosphoremia (hP) is a prominent feature of the RS and seems to be the earliest abnormality. Phosphorus is a vital component of nucleic acids, enzyme systems, and various metabolic pathways.

**Objective** To determine the incidence of refeeding hypophosphoremia (RH) < 0.9 mmol/L, and severe RH < 0.6 mmol/L in a medical critical care unit.

**Patients and methods** Monocentric, retrospective and observational study with patients from FRench-speAking icu Nutritional Survey study FRANS. Critically ill adults (more than 18 yo) were enrolled if they were hospitalized for more than 3 days during a 3-month period and had an artificial nutritional support. Refeeding hypophosphoremia is defined by the occurrence of hypophosphoremia after refeeding. We studied the incidence of HR, risk factors, and prognosis.

**Fig. 28:**
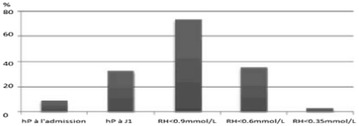
Incidence of hypophosphoremia at admission, the first day, and refeeding hypophosphoremia

**Results** 34 patients were enrolled between 03/01/2015 and 05/31/2015. RH appears in 73.5% and severe RH < 0.6 mmol/L in 35.3% (Fig. 28). There is no correlation between RS risk factors and RH in our study. Logistic regression did not permit to identify neither risk factor nor prognostic modification. There is a lack in phosphoremia measuring (27.6%), and overfeeding during the first 3 days occurs in 29.4%.

**Discussion** We define that an hypophosphoremia appearing after refeeding is a refeeding hypophosphoremia, and we do not consider others etiologies, such as mechanical ventilation, alkalosis, sepsis, alcoholism, malabsorptive states, poor intake, some medication. Our cohort is too small to find some possible correlations with risk factors or prognosis.

**Conclusion** Refeeding hypophosphoremia is common in our population. Hypophosphoremia is not an independent predictor of ICU or in-hospital mortality in critically ill patients. The knowledge of the SRI requires the follow-up of the phosphoremia during nutrition after critical illness in particular in the undernourished patients.

**Competing interests** None.

**References**

1. Marik PE. Refeeding hypophosphatemia in critically ill patients in an intensive care unit. A prospective study. Arch Surg 1996;131:1043–1047.
2. Crook MA. Refeeding syndrome: problems with definition and management. Nutrition 2014;30:1448–55.

#### S115 Impact of the cumulated proteins and energy deficits on ICU patient’s outcome

##### Isaline Page^1^, Stéphanie Warnier^1^, Monique Nys^1^, Anne-Françoise Rousseau^1^, Pierre Damas^1^

###### ^1^Intensive care, CHU Sart-Tilman, Liège, Belgium

####### **Correspondence:** Pierre Damas - pdamas@chu.ulg.ac.be

*Annals of Intensive Care* 2017, **7**(**Suppl 1**):S115

**Introduction** To determine the possible relationship between 10 days cumulated proteins (10 days CPD) and energy deficits (10 days CED) observed in ventilated patients and ICU length of stay, duration of ventilator support, incidence of infections and 28 days mortality.

**Patients and methods** Mixed medical or surgical ventilated for at least 2 days adult patients from 5 ICUs from CHU Liège Belgium were enrolled into the study. They were fed by enteral route with a target of 25 kcal and 1.25 g of proteins by corrected kg of bodyweight and by day. If 50% of the target was not reached on day seven, parenteral nutrition was added with the same target. CED and CPD were calculated for 10 days, taking into account all the sources of nutrition, and was defined as the difference between the amount of energy or protein intake and the target.

**Results** From 10/12/2014 till 30/5/2015, 99 patients were followed. Data from 62 patients could be cumulated on the first 10 days. There were 45 males, mean BMI was 26.9 ± 6.3; SAPS II score on day 1 was 51.3 ± 15.8, SOFA score at day 1 was 9.1 ± 3.5. They were ventilated for a median of 18 days (IQR 13–26), median ICU length of stay was 27 days (IQR 19–35). Mean SOFA max calculated for the first 10 days was 12.1 ± 4.2 and the 28 day mortality was 32.3%. On day 5, only 42% reached the target of 25 kcal/kg and 27% the target of 1.25 g of protein/kg. Mean 10 days CED was −5555.2 ± 4639.9 kcal and mean 10 days CPD was −350.8 ± 252.3 g. There was a significant negative relationship between both deficits and the SOFA max (p = 0.0064 for CED and p = 0.0053 for CPD). However, there were no correlations between any of the deficits and ICU length of stay, duration of mechanical ventilation, occurrence of infections and 28 days mortality.

**Discussion** Saps II level, SOFA max level, ICU length of stay, all these parameters emphasize the high severity of this cohort of patients. It could indeed been thought that it is in this group of critically ill patients that the impact of nutrition could be easily demonstrated. Clear relationships between SOFAmax on day 10 and the 10 days CED and CPD could be seen. However, both the deficit and the level of organ dysfunctions could be cause or consequence. Unlike previous studies, usually performed in less severely ill patients, we did not find any relationship between CED or CPD and patient’s outcome.

**Conclusion** Contrary to some recent studies, we found no relationship between CED and CPE and outcome of patients. Future studies are needed.

**Competing interests** None.

#### S116 Cardiopulmonary bypass induces lymphopenia and decreases lymphocyte proliferation ability: IL-10 and PD-L1 as potential therapeutic targets to reduce postoperative infection

##### Fabrice Uhel^1^, Mathieu Lesouhaitier^1^, Murielle Grégoire^2^, Baptiste Gaudriot^3^, Arnaud Gacouin^1^, Yves Le Tulzo^1^, Erwan Flecher^4^, Karin Tarte^5^, Jean-Marc Tadié^1^

###### ^1^Réanimation médicale, Centre Hospitalier Universitaire de Rennes, Rennes, France; ^2^Biosit and inserm u917, faculte de medecine, université rennes 1, Centre Hospitalier Universitaire de Rennes, Rennes, France; ^3^Anesthésie-réanimation, Centre Hospitalier Universitaire de Rennes, Rennes, France; ^4^Service de chirurgie thoracique, cardiaque et vasculaire, Centre Hospitalier Universitaire, Rennes, France; ^5^Biosit and inserm u917, faculte de medecine, universite rennes 1, Centre Hospitalier Universitaire de Rennes, Rennes, France

####### **Correspondence:** Jean-Marc Tadié - jeanmarc.tadie@chu-rennes.fr

*Annals of Intensive Care* 2017, **7**(**Suppl 1**):S116

**Introduction** Cardiac surgery with cardiopulmonary bypass (CPB) is associated with a generalized inflammatory response with concomitant immune paresis which predisposes to the development of postoperative infections and sepsis (1). Lymphocytes are essential agents of innate and adaptive immune responses during infections or inflammation processes. Lymphopenia has been associated with immune dysfunction during septic shock, and it has been shown that low absolute lymphocyte count was predictive of postoperative sepsis (2). Furthermore, impaired lymphocyte function probably occurs after CPB. Thus, we investigated mechanisms involved in postoperative lymphopenia and impaired lymphocyte function after CPB. The aims of this study were: 1) To describe a potential relationship between lymphopenia and occurrence of postoperative infections. 2) To demonstrate that CPB induces lymphocytes apoptosis. 3) To demonstrate that CPB impaired lymphocyte function (ability to proliferate). 4) To demonstrate that IL-10, PD-L1 (programmed cell death 1 ligand 1) and Indoleamine 2,3-dioxygenase (IDO) could be interesting targets to restore lymphocyte ability to proliferate after CPB.

**Patients and methods** Blood cell counts with differentials obtained within the first postoperative week were analyzed in 828 patients undergoing cardiac surgery in 2015. Postoperative lymphopenia was defined as a lymphocyte count <1.3 × 109 cells L^−1^. Postoperative infections were defined following CDC criteria.

Study procedures: The following analysis were performed before (T0) and 24 h after (T1) cardiac surgery with CPB: Lymphocyte apoptosis; T-cell proliferation ability following polyclonal stimulation; HLA-DR and PD-L1 expression on monocytes; plasma IDO activity and IL-10 levels; and the ability of lymphocytes to undergo a clonal proliferation when stimulated using specific inhibitors of IL-10 and IDO.

The study was approved by our local ethics committee. Patients were informed of the observational nature of the study and gave their consent.

**Results**

1. Early lymphopenia after CPB was associated with the occurrence of postoperative infection: Postoperative infections occured with a median delay of 6 days. Patients who developed postoperative infections had a significantly lower lymphocyte count at Day 4, Day 5 and Day 6 than patients without postoperative infections.
2. CPB induced lymphocyte apoptosis and decreased T-cell proliferation ability.
3. CPB during cardiac surgery decreased mHLA-DR expression.
4. CPB increased IDO activity, PD-L1 expression and IL-10 plasma levels.
5. IL-10 or PD-L1 inhibition of inhibition could restore ability of lymphocytes to proliferate, although IDO inhibitors did not show any effect.

**Conclusion** We provided new evidences that CPB induces immunosuppression. We also demonstrated that IL-10 and PD-L1 could be interesting targets to restore ability of lymphocytes to proliferate. As maintaining MV during CPB decreased plasmatic levels of IL-10, our study brings new evidences that ventilator strategies could be of interest to decrease postoperative infections.

**Competing interests** None.

**References**

1. Gaudriot B, Uhel F, Gregoire M, Gacouin A, Biedermann S, Roisne A, et al. Immune dysfunction after cardiac surgery with cardiopulmonary bypass: beneficial effects of maintaining mechanical ventilation. Shock. 2015;44(3):228–33.
2. Edwards MR, Sultan P, del Arroyo AG, Whittle J, Karmali SN, Moonesinghe SR, et al. Metabolic dysfunction in lymphocytes promotes postoperative morbidity. Clin Sci. 2015;129(5):423–37.

#### S117 Influence of neutropenia on mortality of critically ill cancer patients: results of a systematic review on individual data

##### Quentin Georges^1^, Elie Azoulay^2^, Djamel Mokart^3^, M Soares^4^, Kyeongman Jeon^5^, Sandra Oeyen^6^, Chin Kook Rhee^7^, Pascale Gruber^8^, Marlies Ostermann^9^, Quentin Hill^10^, Peter Depuydt^11^, Christelle Ferra^12^, Alice Muller^13^, Virginie Lemiale^2^, Bourmaud Aurelie^14^, Michaël Darmon^1^

###### ^1^Réanimation médicale, CHU Saint-Etienne - Hôpital Nord, Saint-Étienne, France; ^2^Réanimation médicale, Hôpital Saint-Louis, Paris, France; ^3^Réanimation, Institut Paoli-Calmettes, Marseille, France; ^4^Post-graduation program, D’Or Institute for Research and Education, Rio de Janeiro, Brazil; ^5^Department of critical care medicine and division of pulmonary and critical care medicine, Sungkyunkwan University, Seoul, Republic of Korea; ^6^Department of intensive care, Ghent University hospital, Gent, Belgium; ^7^Division of pulmonary, allergy and critical care medicine, Seoul St. Mary’s Hospital, Seoul, Republic of Korea; ^8^Intensive care, anaesthesia, and surgery, The Royal Marsden Hospital, London, United Kingdom; ^9^Nephrology and intensive care, St Thomas’ Hospital, London, United Kingdom; ^10^Hematology, Leeds Teaching Hospital, Leeds, United Kingdom; ^11^Department of intensive care, Ghent University Hospital, Ghent, Belgium; ^12^Hematology, Catalan Institute of Oncology, Barcelona, Spain; ^13^Department of critical care medicine and pulmonary medicine, hOSPITAL DE CLINICAS DE PORTO ALEGRE, Porto Alegre, Brazil; ^14^Département de santé publique, Institut de Cancérologie de la Loire Lucien Neuwirth, Saint-Priest-en-Jarez, France

####### **Correspondence:** Michaël Darmon - michael.darmon@chu-st-etienne.fr

*Annals of Intensive Care* 2017, **7**(**Suppl 1**):S117

**Introduction** The prognostic impact of neutropenia in critically-ill cancer patients remains controversial. Hence, several studies in critically ill cancer patients failed to demonstrate the impact of neutropenia on outcome [1]. This lack of statistical association might however, reflect a lack of statistical power.

A previous meta-analysis of aggregated data suggested 11% (95% CI 9–14%) raw increase in mortality in neutropenic patients. The available data were, however insufficient to allow adjustment with confounders [2].

The aim of this study was to assess the influence of neutropenia on mortality of critically ill cancer patients using individual data obtained from studies identified by our systematic review. Secondary objectives were to assess the influence of neutropenia on mortality of critically ill patients while taking into account underlying malignancy, use of G-CSF or changes related to period of admission.

**Patients and methods** This systematic review and meta-analysis was performed according to the PRISMA statements. Public-domain databases including PubMed and the Cochrane database were searched by using predefined keywords. The research was restricted to articles published in English and studies focusing on critically ill adult patients from May 2005 to May 2015.

The methods and objectives of this systematic review were reported in the PROSPERO database (CRD42015026347).

Selected manuscripts’ authors were then contacted to obtained part of their dataset.

Mortality was defined as either hospital or day-28 mortality.

This preliminary analysis reports results from the whole dataset before and after adjustment using logistic regression. Period of admission and use of G-CSF were then assessed and were a pre-planned analysis.

**Results** Our initial search yielded 1528 citations and 131 studies were retained for further analysis. Overall, 9 studies were excluded for redundancy with other included studies, 5 as containing only neutropenic patients, and two as containing only palliative patients. Finally 30 datasets (26%) containing sufficient data to allow comparison were obtained from authors.

Overall, 7356 patients were included in this study, including 1666 patients with neutropenia at ICU admission. Median age was of 60 years (IQR 49–69). Median SAPSII score at ICU admission was 42 (IQR 28–57). Respectively 4101 and 3255 patients had underlying haematological malignancy and solid tumours, and 438 patients underwent allogeneic stem cell transplantation. Mechanical ventilation, vasopressors, and renal replacement therapy were required in respectively 50.7% (n = 3729), 41.1% (n = 3024) and 16.1% (n = 1174) of the included patients.

Mortality was of 47.4% in the overall population (n = 3483) and was higher in neutropenic patients (60.3 vs. 43.6% in non-neutropenic patients; P < 0.001). Neutropenia was independently associated with poor outcome when adjusted for underlying malignancy, allogeneic stem cell transplantation and severity as assessed by organ support (OR 1.45; 95% CI 1.27–1.65).

Mortality decreased progressively over time in both non-neutropenic (from 54 to 44%; P < 0.0001) and in neutropenic patients (from 72 to 57%; P < 0.0001). When adjusted for confounders, admission during a more recent period was independently associated with favourable outcome and did not change the final model.

**Conclusion** This preliminary analysis suggests a meaningful survival in neutropenic critically ill cancer patients despite an independent association between neutropenia and mortality.

Additional analyses are on-going in order to adjust for study weight, heterogeneity across studies, assess the influence of neutropenia duration or G-CSF use, and confirm the influence of neutropenia in a predefined subgroup of patients.

**Competing interests** None.

**References**

1. Azoulay, et al. Blood Rev. 2015.
2. Bouteloup, et al. Oncotarget. 2016.

#### S118 Cytokinic profiles kinetic in response to Candida bloodstream infections

##### Christopher Niles^1^, Fabien Herbert^2^, Sylviane Pied^2^, Séverine Loridant,^3^, Nadine François^3^, Anne Bignon^4^, Boualem Sendid^5^, Julien Poissy^1^

###### ^1^Pôle de réanimation, hôpital salengro, C.H.R.U. - Lille, Avenue Oscar Lambret, Lille, France, Lille, France; ^2^Centre d’infection et d’immunité de lille equipe 4 - basic and clinical immunity of parasitic di, Institut Pasteur de Lille, Rue du Professeur Calmette, Lille, France, Lille, France; ^3^Centre de biologie et pathologie génétique, laboratoire de mycologie et parasitologie, Centre Hospitalier Régional Universitaire de Lille, Lille, France; ^4^Service de réanimation chirurgicale, hôpital huriez, Centre Hospitalier Régional Universitaire de Lille, Lille, France; ^5^Inserm u995-2, Universite Lille 2 - Droit et Santé, Lille, France

####### **Correspondence:** Julien Poissy - julien_poissy@hotmail.fr

*Annals of Intensive Care* 2017, **7**(**Suppl 1**):S118

**Introduction** *Candida* bloodstream infections (CBI) are frequent and increasing in hospitalized patients, especially in intensive care units. Considering the results of some experimental in vitro and animal studies, it seems that yeasts belonging to *Candida* genus are able, so as to survive, to modulate the immune response of the host by guiding T cells polarization to Th2 profile. Th1 and Th17 cytokines are known to be involved in host defense against CBI. However, these data are mainly experimental or collected after candidemia. The aim of this study is to precise kinetic of cytokines network during human CBI.

**Patients and methods** This was an ancillary study of an institutional project dedicated to pathophysiology of candidiasis. We have included 32 patients with candidemia and 54 controls (27 matched hospitalized controls and 27 healthy subjects). The sera of cases were gathered before (almost 5 days before), during and after the isolation of yeasts from blood culture, defined as day 0 (D0). Quantitative analysis of 28 cytokines by Luminex^®^ technology and of (1,3)-β-d-glucans by Fungitell^®^ test were performed on 132 samples. The amplitude of Th profile response was expressed by summing the amount of the most relevant cytokines for Th1, Th2 and Th17 profiles, in pg/mL. For each patient, the highest level of response was considered as 100%. Results are expressed for the population by means of the results. We then performed univariate analysis (Fischer exact test for qualitative variables, Mann–Whitney and Wilcoxon test for quantitative variables, Spearman for correlation; GraphPad Prism V6 software) and a multidimensional analysis by principal component analysis (PCA; IgorPro software).

**Results** Patients with candidemia exhibited an increase in pro-inflammatory cytokines (IFNγ, TNFα and IL-12), in comparison with the anti-inflammatory cytokines (IL-4 and IL-10) before D0 (p = 0.034) in univariate analysis. The ratio between mean values reverses at D2 and D3 (p = 0.02) and the increase of Th2 response level from D0 to D4 is correlated to the decrease of Th1 response (r = −0.885; p = 0.033) in univariate analysis and PCA. A pro-inflammatory response (Th1) is associated with a reduced mortality (RR = 0.58 [0.34; 0.98]) and with a lower β-D-glucans levels (p < 0.0001).

**Discussion** We describe here a dynamic cytokine profiles in response to candidemia. Pro-inflammatory response predominates before D0 and reverses after. This is contradictory to the postulate that an anti-inflammatory background could predispose to invasive candidiasis in ICU patients and exhibiting a “Post-infectious immune suppression conditions”. But the relative deficiency in Th1 response compared to simultaneous anti-inflammatory cytokines secretion observed after CBI is in accordance with experimental data, suggesting the modulation of the immune response by *Candida*. The link between cytokinic profile and mortality can also raise the hypothesis of an influence by genetic factors on the regulation and direction of the immune response and so, the existence of a high-risk population.

**Conclusion** These data suggest a relation between *Candida* and the orientation of the immune response towards a pattern deleterious for the infected host. This could allow to determine the most relevant cytokines varying during CBI. They could be used as biomarkers to identify the patients who could benefit from an early treatment in a preemptive targeted therapeutic strategy. These data will be paralleled to genetic background and to circulating *Candida* derived molecules to precise the relative part of the host and the pathogen in this complex interaction.

**Competing interests** None.

#### S120 Neutrophil-to-lymphocyte ratio as an independent predictor of mortality in critically ill cirrhotic patients

##### Mikhael Giabicani^1^, Caroline Lemaitre^1^, Emmanuel Weiss^2^, Steven Grange^1^, Dorothée Carpentier^1^, Gaetan Beduneau^1^, Christophe Girault^1^, Catherine Paugam-Burtz^2^, Fabienne Tamion^1^

###### ^1^Intensive care, Hospital Center University Rouen, Rouen, France; ^2^Anesthesiology and critical care, Hospital Center university Beaujon (AP-HP), Clichy, France

####### **Correspondence:** Mikhael Giabicani - mikhael.giabicani@gmail.com

*Annals of Intensive Care* 2017, **7**(**Suppl 1**):S120

**Introduction** Prognosis of cirrhotic patients hospitalized in intensive care unit (ICU) remains poor. Many studies suggested a negative impact of systemic inflammation on organ failure and outcome in cirrhosis(1). In ICUs, cirrhotic patients are widely admitted and revalued after receiving optimal treatments for 3 days. However, little is known about how manage these patients after day 3 according to their prognosis. The blood neutrophil-to-lymphocyte ratio (NLR) as a novel inflammation index biomarker has been reported to be a predictor of clinical outcomes in various malignancies and in unselected critically ill patients(2). NLR has also been identified as a predictor of mortality in patients with stable liver cirrhosis. To our knowledge, the ability of NLR to predict outcome in critically ill cirrhotic patients has never been studied. The aim of this study was to evaluate the usefulness of inflammatory marker such as NLR for diagnosis of infection and predicting the outcome in hospitalized critically ill cirrhotic patients.

**Patients and methods** We performed a retrospective monocentric study including consecutively cirrhotic patients hospitalized in a medical ICU from 2010 to 2014. For each patient, clinical and biological data at admission and day 3 were collected. NLR at admission (“NLRD0”), at day 3 (“NLRD3”) and the variation of NLR between admission and D3 (“delta NLR”) were calculated. Statistical analysis used appropriate non parametric tests and Cox regression for survival analysis. The ability of the variables to discriminate survivors from non-survivors was determined using ROC curves. Results are expressed as median (IQR).

**Fig. 29:**
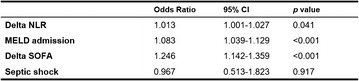
Multivariate analysis of factors associated with mortality

**Results** During the study period, 149 cirrhotic patients were admitted in ICU. The etiologies of liver cirrhosis were alcoholic in 87% of cases with severe score: median Child-Pugh score = 9 [7–11], median MELD score = 25 [18.3–30.8]. Main reasons for ICU admission were sepsis (40.9%), gastrointestinal bleeding (23.5%) and renal failure (19.5%). NLRD0 was higher for patients hospitalized for septic shock (p < 0.001). Patients were followed up for 23 days [5.5–289.5]. 90 (59.6%) patients died including 57 (37.7%) deaths in ICU and 18 (12.1%) deaths after ICU discharge during the same hospitalization. NLR decreased for survivors between D0 and D3 (−4.5 [−9.8 to 0.0]) whereas it increased for non-survivors (+1.2 [−3.1 to +10.1]). In univariate analysis, for predicting survival, higher values of NLRD3, delta NLR, MELD score at admission, SOFA score at admission and at day 3 and delta SOFAD0-D3 were significant factors. Predictors of death in multivariate analysis are shown in Fig. 29. Area under delta NLR ROC curve was 0.72 (CI = 0.61–0.83).

**Conclusion** The blood NLR is a novel inflammation index that has been shown to independently predict poor clinical outcomes. We have demonstrated that delta NLR is an independent predictor of mortality in critically ill cirrhotic patients. Delta NLR could help to identify cirrhotic patients at risk of unfavorable outcome.

**Competing interests** None.

**References**

1. Salciccioli JD, et al. The association between the neutrophil-to-lymphocyte ratio and mortality in critical illness: an observational cohort study. Crit Care. 2015;19:13.
2. Gandoura S, et al. Gene- and exon-expression profiling reveals an extensive LPS-induced response in immune cells in patients with cirrhosis. J Hepatol. 2013;58(5):936–48.

#### S121 Serum procalcitonin is a good diagnostic marker of bacterial infection in patients with hematologic malignancies in the Intensive Care Unit

##### Celine Dupre^1^, Saad Nseir^2^, Anne-Sophie Moreau^1^

###### ^1^Centre de réanimation, C.H. Régional Universitaire de Lille (CHRU de Lille), Lille, France; ^2^Centre de Réanimation, Centre Hospitalier Régional Universitaire de Lille, Lille, France

####### **Correspondence:** Celine Dupre - duprecece@gmail.com

*Annals of Intensive Care* 2017, **7**(**Suppl 1**):S121

**Introduction** Diagnosis of infection in immunocompromised patients can be difficult. However, diagnosing infection is very important, particularly in critically ill. This study aims to evaluate the benefit of procalcitonin (PCT) blood level as a diagnostic marker for bacterial infection in patients with hematological malignancies admitted to the Intensive Care Unit (ICU).

**Patients and methods** This retrospective single-center study included all consecutive patients with acute myeloid leukemia or high grade lymphoid malignancy admitted to the ICU. Patients were sorted into three subgroups, according to clinical and microbiological data: «Infectious disease», «no infectious disease» and «Unknown». Initial serum PCT and when available at day 3 and day 5 were recorded. Receiver Operating Characteristic (ROC) curve, sensitivity and specificity were calculated. Serum PCT was considered as decreasing when the decrease was ≥25% at day 3 and/or ≥50% at day 5. Mortality rates in the ICU and at day-90 were also studied.

**Fig. 30:**
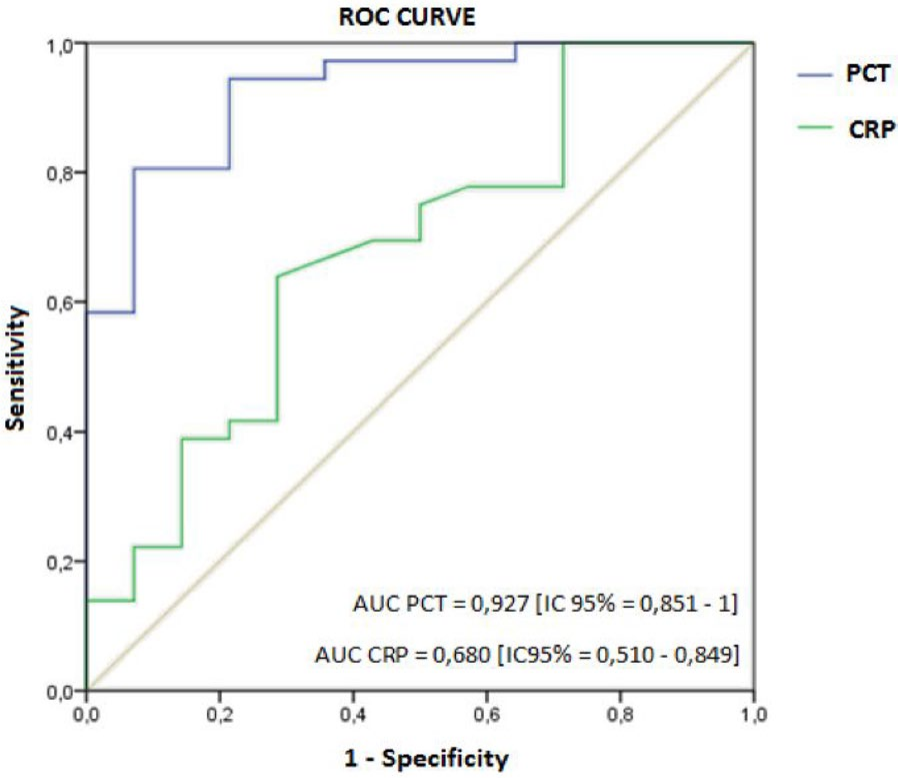
ROC Curve of initial blood level of PCT and CRP as markers of bacterial infection

**Results** Fifty-four patients were included in the study. At diagnosis, PCT levels were significantly different between the “Infection disease” group and the “No infection disease” group (p = 0.002). There was no difference between the “Infection disease” group and the “Unknown” group (p = 0.052). For the diagnosis of bacterial infection, best initial serum PCT threshold was 0.65 ng per milliliter. For that threshold, sensitivity was 94.6% and specificity was 78.6%. PCT area under the ROC curve was 0.93 [CI 95% = 0.855–1]. Youden’s J statistic was 0.73. PCT levels weren’t different between groups according to the presence of neutropenia or in case of inaugural disease. There was a significant difference in PCT values between groups according to the SOFA score (p = 0.027), but not the SAPS2 score. Mortality rate in the ICU and at day-90 were significantly lower for the patients with decreasing PCT (p < 0.001 and p < 0.001, respectively). When comparing serum PCT and CRP predictive values, PCT was significantly a better marker of bacterial infection (Fig. 30).

**Discussion** We found that serum PCT, with a threshold of 0.66 ng/mL, is a reliable marker of bacterial infection disease in patients with aggressive hematological malignancy admitted to the ICU. Our study confirms the results of a previous study in unselected immunocompromised patients admitted to the ICU, showing a 100% sensitivity, a 63% specificity and an area under ROC curve of 0.851 [0.78–0.92] for a threshold of 0.5 ng/mL (1).

The main limitations of our study are its retrospective design and the small number of included patients.

**Conclusion** PCT is a reliable marker of bacterial infection in patients with hematological malignancies admitted to the ICU. PCT kinetic seems to be an interesting prognostic marker in this population.

**Competing interests** None.

**Reference**

1. Bele N, Darmon M, Coquet I, Feugeas J-P, Legriel S, Adaoui N, Schlemmer B, Azoulay É. Diagnostic accuracy of procalcitonin in critically ill immunocompromised patients. BMC Infect Dis. 2011;11:224.

#### S122 Could Endocan plasmatic level predict the nature of pathogenic microorganisms in sepsis?

##### Aymen Zayene^1^, Lucie Portier^2^, Nathalie De Freitas Caires^2^, Philippe Lassalle^3^

###### ^1^59, C.H. Régional Universitaire de Lille (CHRU de Lille), Lille, France; ^2^Inserm u1019 e13, Institute Pasteur De Lille, Lille, France; ^3^59, LUNGINNOV, Lille, France

####### **Correspondence:** Aymen Zayene - aymen.z@hotmail.com

*Annals of Intensive Care* 2017, **7**(**Suppl 1**):S122

**Introduction** Endocan is a proteogycan secreted by pulmonary and renal endothelial cells (Lassalle et al.). Its synthesis is increased in sepsis and is regulated by Lipopolysaccharide (LPS) and cytokines.

Toll Like Receptors (TLRs) are receptors of innate immunity and recognize diverse exogenous and endogenous patterns.

In this study, we have compared the effect of 2 TLRs agonists on regulation of secretion and expression of Endocan by Human Umbilical Veinous Endothelial Cells (HUVECs).

**Materials and methods** The stimulation of HUVECs was performed by 2 TLRs agonists: LPS and PolyInossinic Polycytidylic acid (Poly (I:C)).

The secretion of Endocan was evaluated by Enzyme Linked Immuno Sorbant Assay (ELISA) in the supernatant of stimulated HUVECs.

Real Time Polymerase Chain Reaction (qPCR) study compared the expression of Endocan in the two groups.

**Results** The secretion of Endocan by HUVECs is upregulated by LPS and poly (I:C).

Kinetics of secretion of Endocan was faster with LPS than Poly (I:C).

q PCR study confirmed that Endocan is overexpressed in HUVECs stimulated with LPS than Poly (I:C).

**Discussion** TLR 4 is involved in the recognition of LPS (Kawai et al.), a component of the outer membrane of Gram Negative Bacteria, respiratory syncytial virus fusion proteins, *Steptococcus pneumoniae* pneumolysin.

TLR 3 was originally identified as recognizing a synthetic analog of double stranded Ribonucleic Acid (dsRNA), poly(I:c) which mimics viral infections.

In this study, we have found that kinetics of secretion and expression of Endocan is faster with HUVECs stimlated by TLR 4 agonist than TLR 3 agonist.

This results could suggest that Endocan may be not only a marker of septic shock but could be also a specific marker to recognize the nature of pathogenic microorganisms in septic shock.

Furthermore, other studies with more TLR agonists could be useful to confirm these results.

**Conclusion** Studying the effects of diverse TLRs agonists could make the plasmatic dosage of Endocan more specific and helpful to recognize the nature of pathogenic microorganisms in septic shock.

**Competing interests** None.

**References**

1. Kawai T, Akira S. The role of pattern-recognition receptors in innate immunity: update on Toll-like receptors. Nat Immunol. 2010;21(8):373–82.
2. Lassalle P, Molet S, Janin A. ESM-1 is a novel human endothelial cell specific molecule expressed in lung and regulated by cytokines. J Biol Chem. 1996;271(34):20458–64.

## Physiotherapists abstracts

### Oral communications

#### O79 Lung ultrasound: help to the diagnostic and the monitoring of response to physiotherapy. A case report of pneumonia

##### Aymeric Le Neindre^1^, Pascal Selot^1^, Daniel Ferreiro,^1^, Maria Bonarek,^1^, Stépahen Henriot,^1^, Julie Rodriguez,^1^

###### ^1^Physiotherapy, Hôpital Forcilles - Fondation Cognacq-Jay, Férolles-Attilly, France

####### **Correspondence:** Aymeric Le Neindre - aymeric.leneindre@gmail.com

*Annals of Intensive Care* 2017, **7**(**Suppl 1**):O79

**Introduction** Lung ultrasound is widely used in intensive care, ermergency and pneumology medicine, for assessing acute respiratory pathologies. It is noninvasive, radiation free and rapidly available at the patient’s bedside and provides an excellent accuracy. So, lung ultrasound may be an interesting tool for the physiotherapist as it allows to assess with more accuracy the patient improving the chest physiotherapy indication and monitoring (1).

As far as we are aware, no study has evaluated the impact of lung ultrasound on clinical-decision making by physiotherapists in the use of chest physiotherapy.

This case report highlights the lung ultrasound interest in chest physiotherapy in patient with lung consolidation.

**Patients and methods** This was a case report written following the recommendations of the CARE guideline (2).

The case was a 68-years-old female patient, non intubated, hospitalized in a respiratory ICU. She was hypoxemic (PaO_2_ = 59 mmHg and SaO_2_ = 89%), with dyspnoea at rest and an increasing radiological opacity at the right lung base. Hypoxemia was the indication for physiotherapist referral.

At the clinical examination, the physiotherapist’s findings were: decreased mobility, dullness and abolished vesicular sound at the base of right hemithorax.

This clinical examination and chest x-rays analysis allowed the physiotherapist to propose several clinical hypotheses: pleural effusion, obstructive atelectasis or pneumonia.

The chest physiotherapy treatment differs according to the type of lung deficiencies. For example, the physiotherapist must to refer the patient to the medical staff in case of pleural effusion or may implement hyperinflation technique in case of obstructive atelectasis. Determining the nature of lung deficiencies is essential to provide the more suitable therapeutic strategy. So, the physiotherapist decided to perform a lung ultrasound examination to retain the more likely hypothesis.

**Results** Ultrasound examination performed by the physiotherapist highlighted the presence of a lung consolidation at the infero-lateral and posterior parts of the right lung with a pneumonia pattern: presence of tissue-like sign, shred sign, dynamic air bronchogram and fluid bronchogram.

The medical staff implemented antibiotic treatment. The ultrasound findings guided the physiotherapist to choose chest physiotherapy technique improving the alveolar recruitment: nearly prone position (left side down) and continuous positive airway pressure during 45 min.

The patient response to the treatment was monitored by ultrasound and showed a decrease of the lung consolidation size and apparition of B lines, meaning a gain of lung aeration. These findings were associated with SpO_2_ improvement but without decrease of dyspnoea.

**Discussion** Lung ultrasound allowed the physiotherapist to precise the nature of the radiological lung opacity. As it is more accurate than clinical examination or chest x-ray, this suggests a more suitable choice of chest physiotherapy techniques than conventional clinical decision-making process.

Ultrasound findings suggested a positive response to the chest physiotherapy treatment. The apparition of re-aeration signs (B lines, decreased consolidation size) showed a short-term efficacy of the chest physiotherapy treatment. This allowed the physiotherapist to continue the treatment during 1 week and obtain a substantial clinical improvement.

**Conclusion** The use of lung ultrasound in the clinical decision-making process may help the physiotherapist to choose with more accuracy the therapeutic strategy. Moreover, it allows to monitor the treatment in real-time and assess the patient’s response. The use of this tool may allow the physiotherapist to determine the optimal indications for chest physiotherapy and thus avoid unnecessary or inappropriate treatments.

**Competing interests** None.

**References**

1. Le Neindre A, Mongodi S, Philippart F, Bouhemad B. Thoracic ultrasound: potential new tool for physiotherapists in respiratory management. A narrative review. J Crit Care. 2016;31(1):101–9.
2. Gagnier JJ, Kienle G, Altman DG, Moher D, Sox H, Riley D. The CARE guidelines: consensus-based clinical case reporting guideline development. J Med Case Rep. 2013;7(223):1–6.

#### O80 Chronic critical illness: a prospective observational study in Florence, Italy

##### Mara Taddei^1^, Mauro Di Bari^2^

###### ^1^Department of healthy profession, intensive care unit, division of cardiology, Careggi University Hospital, Firenze, Italy; ^2^Division of geriatric cardiology and medicine, research unit of medicine of ageing, Careggi University Hospital, Firenze, Italy

####### **Correspondence:** Mara Taddei - marataddei@libero.it

*Annals of Intensive Care* 2017, **7**(**Suppl 1**):O80

**Introduction** Chronic Critical Illness (CCI) syndrome is a new condition affecting an increasing number of patients, who survived an acute critical illness but have persistent severe organ dysfunction, requiring prolonged specialized care. CCI is a iatrogenic process, reflecting the efficacy of modern life support technologies(1), and encompasses multiple organ failure, need for prolonged mechanical ventilation (MV), organ support, and palsy due to polineuromyopathy. The transition from acute to CCI is gradual: definitions are based on duration of MV, with cut-offs of 7, 14 or 21 consecutive days of MV for ≥6 h/day. CCI patients may come from either medical or surgical ICU; their health status fluctuates between improvements and deteriorations implying recurrent transitions between different levels of care (1).The risk of death is reported to be as high as 50%. Despite a relatively young age (65 years on average), functional status of CCI patients discharged is seriously impaired, thus CCI patients require long-term rehabilitation.

AIM: To estimate the frequency of CCI Syndrome in Careggi, a large academic, tertiary care hospital; to describe the clinical course of CCI patients through discharge, and their functional status at discharge.

**Patients and methods** Administrative data on admission, transfer, death and discharge of all CCI patients, consecutively admitted in one of the 56 ICU beds at Careggi Hospital from January 1 to December 31, 2014, were collected. CCI was defined with the cut off of ≥21 days of ICU stay, representing the index event (IE) without contribution of previous or subsequent hospitalization in other hospitals. Reasons for admission were grouped into the 4 broad categories of medical causes, surgery, major trauma and cardio-respiratory arrest. Patients discharged were evaluated in daily living, cognitive status, and mobility using Barthel Index.

**Results** We identified 123 subjects who developed CCI (71 males; age 61.7 ± 1.5 years, mean ± SEM); 36 of them came from an external ICU, 87 began their CCI course within Careggi hospital (60 from the Emergency Room, 27 from a regular ward). Average duration of the IE was 36.1 ± 2.1 days. These sample developed accumulative length of ICU stay of 4440 days, corresponding to a 22% ICU bed occupation over the theoretical total of 20,440. When days of sub-intensive care and regular ward were separately added, 5500 days of highly specialized care and 6266 days of total acute hospital stay were reached. Surgical patients had longer hospitalizations (p = 0.009).CCI patients confirmed to be highly erratic: a total of 302 transitions across different services were recorded in the 123 patients, with a maximum of 9 in 6 of them. Mean age was comparable between the 27 patients who died (22%) and the remaining 95 who were discharged alive (66.9 ± 2.5 vs. 60.3 ± 1.7 years; p = 0.058).Fourteen subjects continued their ICU stay out of hospital. Only 6, whose age was lower (37.7 ± 3.7 years), were discharged home; half of the participants (n = 68, 55.2%) were admitted to a residential rehabilitation facility. Younger subjects scored better in the domains of self care (p = 0.018) and cognitive status (p = 0.008) but not in the domain of mobility, including walking ability: 45 patients required maximal assistance in performing activities of daily living and transfers, other 12 required medium/maximal assistance, with no statistical difference between DG group.

**Conclusion** CCI is a relevant clinical condition that need to be assessed and possibly prevented, as it causes severe morbidity, long-term functional impairment and exceeding healthcare costs.

**Competing interests** None.

**Reference**

1. MacIntyre NR, Fan E, et al. Journal Conference on Chronic Critical Illness. Resp. Care 2012;57(6).

#### O81 Physical therapy during the early course of sepsis is safe and preserves skeletal muscle mass

##### Cheryl Hickmann^1^, Diego Castanares-Zapatero^1^, Louise Deldicque^2^, Peter Van Den Bergh^3^, Gilles Caty^4^, Jean Roeseler^1^, Marc Francaux^2^, Pierre-François Laterre^1^

###### ^1^Intensive care unit, Cliniques universitaires Saint-Luc, Université catholique de Louvain, Bruxelles, Belgium; ^2^Institute of neuroscience, Université catholique de Louvain, Louvain-la-Neuve, Belgium; ^3^Neuromuscular reference centrer, Cliniques universitaires Saint-Luc, Université catholique de Louvain, Brussels, Belgium; ^4^Department of physical medicine and rehabilitation, Cliniques universitaires Saint-Luc, Université catholique de Louvain, Brussels, Belgium

####### **Correspondence:** Cheryl Hickmann - cheryl.hickmann@uclouvain.be

*Annals of Intensive Care* 2017, **7**(**Suppl 1**):O81

**Introduction** Critical illness together with immobilization have deleterious effects on patients outcome, especially in the presence of sepsis. Increased muscle catabolism and membrane inexcitability reduce muscular mass and impair function within the first days after sepsis onset (1). Early mobilization could potentially limit muscle wasting and functional impairment in this population. The purpose of this study was to test whether exercise during the early phase of sepsis is safe and beneficial and to which extent it can limit skeletal muscle protein catabolism and preserve function.

**Patients and methods** Adult patients admitted with the diagnosis of severe sepsis were included and randomly allocated to two groups; 1) Control group (Ctrl-G): manual passive/active manual mobilization twice a day or 2) Experimental group (Exp-G): additional two times 30 min of passive/active cycling exercise. Both groups benefited from a reduced sedation, adjusted nutritional intake and bed to chair transfer as soon as possible.

Skeletal muscle biopsy and electrophysiological testing were realized at day-1 and day-7. Muscle histology, biochemical and molecular analyses of anabolic/catabolic and inflammatory signalling pathways were performed. A group of four healthy subjects was used to obtain non pathological values.

Hemodynamic parameters and patients perception were collected during each session.

**Results** Twenty-one patients were included, however 3 died before the second muscle biopsy. Ten patients in Ctrl-G and nine in Exp-G were finally analysed. Muscle fibre cross sectional area (µm^2^) was significantly preserved by exercise (relative changes were Ctrl-G: −45 ± 41% vs Exp-G:12 ± 19%, p = 0.001). Markers of catabolic systems were highly increased during sepsis compared to healthy subjects and reduced in both groups 7 days after admission. However the reduction in mRNA (relative change) tended to be more important in Exp-G: MURF-1 (Ctrl-G: −31 ± 67% vs Exp-G: −63 ± 45%, p = 0.15), MAFbx (Ctrl-G: −7 ± 138% vs Exp-G: −56 ± 37%, p = 0.23), LC3b (Ctrl-G: 5 ± 47% vs Exp-G: −21 ± 18%, p = 0.18) and Bnip3 (Ctrl-G: 27 ± 198% vs Exp-G: −59 ± 23%, p = 0.02). Anabolic and inflammatory markers were not affected by exercise.

Electrophysiological testing, including direct muscular stimulation, was abnormal on Day-1 in 10 of 13 evaluated patients. Since only a limited number of patients could be reassessed a second time, comparison between groups was not possible.

In general, all activities were well tolerated by patients with no adverse events.

**Conclusion** Early mobilization during the first week of the sepsis onset was safe and preserved muscle fibre cross sectional area.

**Competing interests** None.

**Reference**

1. Kress JP, Jesse BH. ICU-Acquired weakness and recovery from critical illness. N Engl J Med. 2014;(370):1626–35.

#### O82 Where should we place the stethoscope’s chestpiece to hear the noise of the primary bronchi?

##### Frédéric Duprez^1^, Bastien Dupuis^2^, Grégory Cuvelier^2^, Thierry Bonus^1^, Sandra Ollieuz^1^, Sharam Machayeckhi^1^, Gregory Reychler^3^

###### ^1^Icu, C.H. Epicura, Hornu, Belgium; ^2^Laboratoire de l’effort et du mouvement, Condorcet, Tournai, Belgium; ^3^Service de pneumologie, Cliniques Universitaires Saint Luc, Bruxelles, Belgium

####### **Correspondence:** Frédéric Duprez - dtamedical@hotmail.com

*Annals of Intensive Care* 2017, **7**(**Suppl 1**):O82

**Introduction** The pulmonary auscultation is used by respiratory therapist (RT) to evaluate the efficiency of a treatment. Listen to the noises coming from the primary bronchi (PB) is important because it is the place where secretions can be accumulated. Therefore, it is crucial to know exactly where to place the stethoscope’s chestpiece on the chest. Few studies have analyzed the chest area where the PB were located. Our hypothesis is that PB are localized on a line that joins axillary fossa (Bi-Axillary line: BAL). The aim of our study is to evaluate the probability to find the primary bronchi by analysis of chest radiography.

**Patients and methods** A retrospective study was performed by analysis of chest X-Ray using the software: TM reception^®^, which allows precise measures to the tenth of millimeter. All the X-Rays were made on confined to bed patients hospitalized within intensive care unit, internal medicine and abdominal surgery rooms.

The following measures (in mm) were made between:

- Lowered perpendicular (LP) of:
- Bi-Axillary Line (BAL) and the sternal carina (SC)
- BAL’s and the position of right and left PB
- Middle of the body-sternum (BS) and the perpendicular middle of right and left PB.
- Hyoid bone and the SC

The exclusion criteria were: BMI < 18.5 kg/m^2^ and BMI > 30 kg/m^2^, scoliosis, minor patient, lack of visibility of one of the axillary fossa, lack of visibility of PB, clavicular asymmetry, kyphosis, lack of symmetry in the shot, atelectasis and pneumothorax.

Statistics: Normality test: KS. Mean values are expressed with their SD and 95% CI.

**Results**

**Discussion** In this study, we performed analysis of chest x-Rays of bedridden patients and we demonstrated that it is possible to localize easily, on either side of the BS, the right and left PB at ± 25 mm distance (LP) above a line joining axillary fossa. This study constitutes a new tool for the RT who, by using stethoscope with a chestpiece of 10 cm^2^ surface area, will be able to listen to noise coming from PB.

**Conclusion** The data presented herein (Fig. 31) show that right and left PB are located at a mean distance of 25 (±5) mm and 27 (±6) mm above the BAL, on both sides of the BS. The BAL represents thus an easy and precise mode to detect right and left PB by bedridden. Finally, the distance between the hyoid bone and the SC is about 12 cm. As the PB are located after the bifurcation, this information constitutes another useful way for the localization the right and left PB by bedridden patient.

**Fig. 31:**
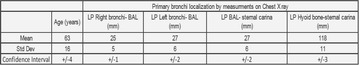
50 X-Rays (Men = 26, women = 24) have been analyzed. Normality test passed. *LP* lower perpendicular, *BAL* bi-axillary line

**Competing interests** None.

#### O83 Study of efficacy on ICU acquired weakness of early standing with the assistance of a tilt table in critically ill patients

##### Celine Sarfati^1^, Alex Moore^1^, Paula Mendialdua^1^, Emilie Rodet^1^, Catherine Pilorge^2^, Francois Stephan^2^, Saida Rezaiguia-Delclaux^2^

###### ^1^Physiotherapy department, Surgical Center Marie Lannelongue, Le Plessis-Robinson, France; ^2^Réanimation adulte, Surgical Center Marie Lannelongue, Le Plessis-Robinson, France

####### **Correspondence:** Celine Sarfati - celine.sarfati@yahoo.com

*Annals of Intensive Care* 2017, **7**(**Suppl 1**):O83

**Introduction** Critically ill patients frequently develop muscle weakness, which is associated with prolonged intensive care unit and hospital stay (1). This randomized controlled trial (Clinical Trials NCT02047617) was designed to investigate whether a daily training session using a tilt table, started early in stable critically ill patients with an expected prolonged ICU stay, could improve strength at ICU and hospital discharge compared to a standard physiotherapy program.

**Patients and methods** The study protocol was approved by an ethics committee and informed consent was obtained from all patients. Patients admitted in adult ICU of Marie Lannelongue hospital, France, who were mechanically ventilated for at least 3 days were included. Exclusion criteria were cerebral or spinal injury, pelvic or lower limb fracture. Patients were assessed each day for temporary contraindications for mobilization out of bed (RASS score <−2 or >1; hemodynamic instability; a continuous intravenous dose of epinephrine/norepinephrine >2 mg/h; continuous renal replacement; ECMO). Interventions for patients assigned to the standard physiotherapy group (Std) included sitting in armchair for at least 2 h per day. In addition to the standard physiotherapy mobilization protocol, patients assigned to the Tilt table physiotherapy group (Tilt) were positioned on a tilt table for 1 h per day. The primary outcome was the muscles strength evaluated using the Medical Research Council (MRC) score (range 0 = no muscle contraction, to 5 = normal strength), scoring 3 muscle groups in each limb, at ICU and hospital discharge, compared to MRC score evaluated in ICU before the implementation of the physiotherapy program. Secondary outcome variables included ICU and hospital length of stay.

**Results** The flow chart of the study, conducted between October 2013 and October 2014, is presented in the Fig. 32.

**Fig. 32:**
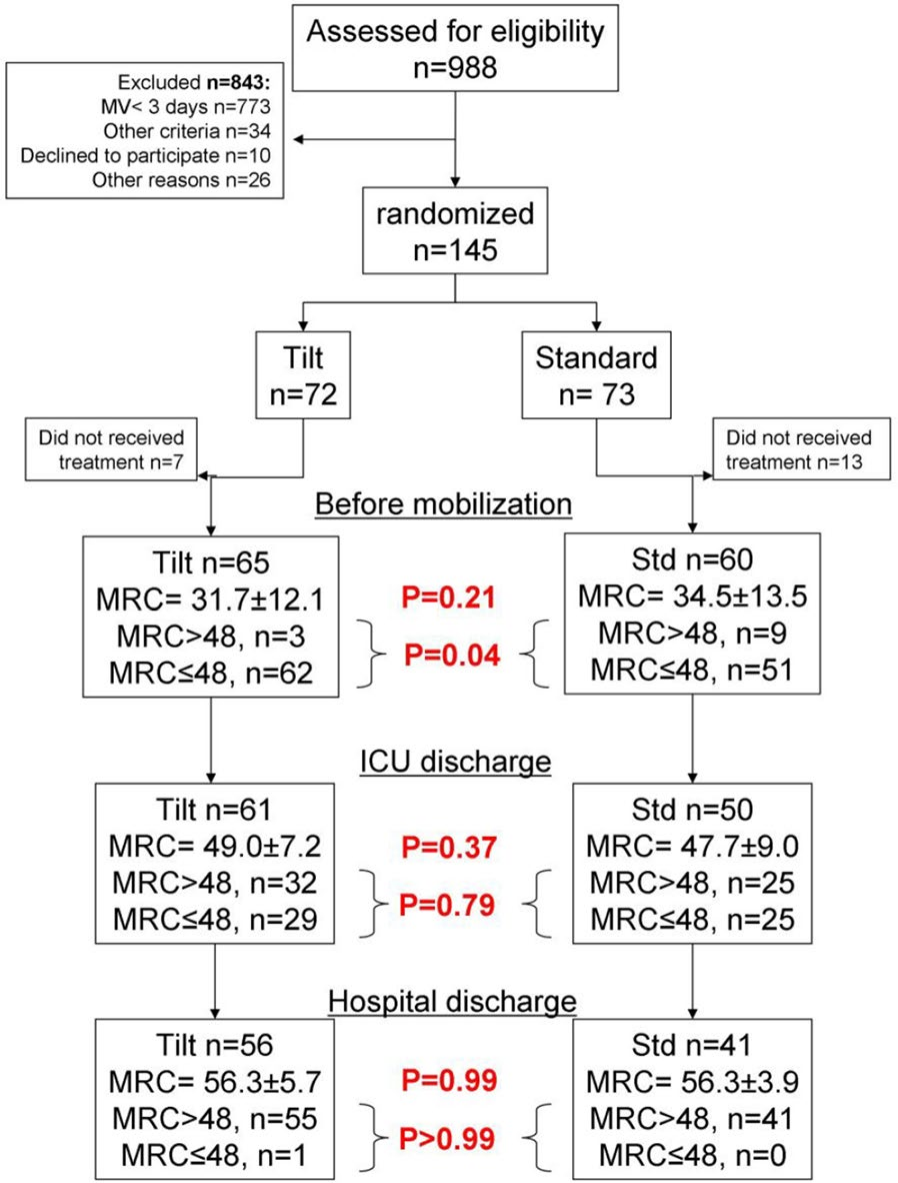
See text for description

Both groups (Std vs Tilt) are comparable for age (63 ± 16 vs 60 ± 15, p = 0.37), gender (21 Females vs 26 F and 52 Males vs 46 M, p = 0.34) and the SAPSII (43 ± 13 vs 42 ± 12, p = 0.65). No significant difference was observed in terms of MRC score or in terms of pts with or without weakness (MRC > 48) at ICU or hospital discharge. However, the number of pts with weakness was significantly higher in the group before Tilt mobilization, suggesting a more rapid improvement in the tilt group. The ICU and hospital lengths of stay were not different between groups.

**Discussion** The prevalence of muscle weakness in our population is high before mobilization (90.6%, 95% CI 85.2–95.6), is still 48.6% at ICU discharge but represents only ~ 1% at hospital discharge. This low hospital discharge prevalence is probably related to the early and intense physiotherapy in both groups, which may explain our inability to demonstrate superiority of the addition of tilt table positioning, although a faster recovery is suggested.

**Conclusion** Training sessions using a tilt table, in addition to early and intense physiotherapy did not improve muscle strength evaluated using MRC score in surgical ICU patients with muscle weakness.

**Competing interests** None.

**Reference**

1. Am J Respir Crit Care Med. 2007;175:480–9.

#### O84 Aerosol delivery using two nebulizers through high flow nasal cannula: a randomized cross-over SPECT-CT study

##### Jonathan Dugernier^1^, Michel Hesse^2^, Thibaud Jumetz^1^, Emilie Bialais^1^, Jean Roeseler^1^, Virginie Depoortere^2^, Jean Bernard Michotte^3^, Xavier Wittebole^1^, Pierre-François Laterre^1^, François Jamar^4^, Gregory Reychler^5^

###### ^1^Sevice des soins intensifs, Cliniques Universitaires Saint-Luc, Bruxelles, Belgium; ^2^ Médecine nucléaire, Cliniques Universitaires Saint-Luc, Bruxelles, Belgium; ^3^Respiratory, Haute École de Santé Vaud, Lausanne, Switzerland; ^4^Service de médecine nucléaire, Cliniques Universitaires Saint-Luc, Woluwe-Saint-Lambert, Belgium; ^5^Service de pneumologie, Cliniques Universitaires Saint Luc, Bruxelles, Belgium

####### **Correspondence:** Jonathan Dugernier - jonathan.dugernier@uclouvain.be

*Annals of Intensive Care* 2017, **7**(**Suppl 1**):O84

**Introduction** Patients with high flow nasal cannula may benefit from combined aerosol therapy. Clinical efficacy depends on pulmonary deposition which is related to the type of nebulizer. All new nebulizers or delivery methods require rigorous evaluation. The aim of this study was to compare lung deposition between two nebulizers (jet nebulizer vs vibrating-mesh nebulizer) through high flow nasal cannula in healthy subjects.

**Patients and methods** Aerosol delivery of diethylenetriaminepentaacetic acid labelled with technetium-99m (99mTc-DTPA, 4 mCi/4 mL) to the lungs using a vibrating-mesh nebulizer (Aerogen Solo^®^, Aerogen Ltd., Galway, Ireland) and a constant-output jet nebulizer (Opti-Mist Plus Nebulizer^®^, ConvaTec, Bridgewater, NJ) through high flow nasal cannula (Optiflow^®^, Fisher & Paykel, New Zealand) was compared in 6 healthy subjects. Flow rate was set at 30 L/min through the heated humidified circuit. Pulmonary and extrapulmonary deposition were measured by single photon emission computed tomography combined with a low dose CT-scan (SPECT-CT) and by planar scintigraphy.

**Results** Lung deposition was only 3.3 ± 1.3 and 1.2 ± 0.8% of the nominal dose with the vibrating-mesh nebulizer and the jet nebulizer, respectively (p < 0.05). Dose lost in the high flow circuit, humidification chamber and nasal cannula was higher with the vibrating-mesh nebulizer as compared to the jet nebulizer (75.2 ± 8.2 vs 45.0 ± 6.2% of the nominal dose, p = 0.001). Expressed as percentage of emitted dose, lung deposition was similar with both nebulizers.

**Conclusion** This study demonstrated that aerosol delivery through HFNC is poor in the specific conditions of the study despite the higher efficiency of the vibrating-mesh nebulizer as compared to the jet nebulizer. Placing the nebulizer on the HFNC circuit at 30 L/min induces high aerosol loss on the circuit and the oropharynx.

**Competing interests** J. Dugernier: unrestricted grant by Aerogen Ltd. (Galway, Ireland).

